# Author Correction: Glycopeptide database search and de novo sequencing with PEAKS GlycanFinder enable highly sensitive glycoproteomics

**DOI:** 10.1038/s41467-024-45153-x

**Published:** 2024-01-24

**Authors:** Weiping Sun, Qianqiu Zhang, Xiyue Zhang, Ngoc Hieu Tran, M. Ziaur Rahman, Zheng Chen, Chao Peng, Jun Ma, Ming Li, Lei Xin, Baozhen Shan

**Affiliations:** 1 Bioinformatics Solutions Inc., Waterloo, ON Canada; 2 David R. Cheriton School of Computer Science, University of Waterloo, Waterloo, ON Canada; 3 BaizhenBio Inc., Wuhan, China; 4 Wuhan BioBank, Wuhan, China; 5 Henan Academy of Sciences, Zhengzhou, Henan China

**Keywords:** Proteomics, Glycobiology, Glycomics, Proteome informatics, Software

Correction to: *Nature Communications* 10.1038/s41467-023-39699-5, published online 08 July 2023

The original version of this article contained an error in the Code Availability Statement, which incorrectly omitted from the end the following: ‘Our glycan de novo sequencing tool GlycoNovo uses the Python glypy library [50]’. This has been corrected in both the PDF and HTML versions of the article.

The original version of this article omitted a reference to previous work in ‘Klein, J. & Zaia, J. glypy - An open source glycoinformatics library. *J. Proteome Res*.**18**, 3532–3537 (2019).’. This has been added as reference [50] in the Code Availability Statement: ‘Our glycan de novo sequencing tool GlycoNovo uses the Python glypy library [50]’. This has been corrected in the PDF and HTML versions of the article.

