# Supplementary material for: Glycopeptide database search and de novo sequencing with PEAKS GlycanFinder enable highly sensitive glycoproteomics
Source: Nat Commun. 2023 Jul 8;14:4046. doi: 10.1038/s41467-023-39699-5 (PMC10329677; doi:10.1038/s41467-023-39699-5)
Supplement: Supplementary file 1 — Supplementary Information [file 41467_2023_39699_MOESM1_ESM.pdf]

Glycopeptide database search and de novo sequencing with PEAKS GlycanFinder enable highly sensitive glycoproteomics

Weiping Sun[1,*], Qianqiu Zhang[2,*], Xiyue Zhang[1,*], Ngoc Hieu Tran[1,2,*], M Ziaur Rahman[1], Zheng Chen[1], Chao Peng[3], Jun Ma[1], Ming Li[2,**], Lei Xin[1,**], Baozhen Shan[1,**]

[1] Bioinformatics Solutions Inc., Waterloo, Ontario, Canada.

[2] David R. Cheriton School of Computer Science, University of Waterloo, Ontario, Canada.

[3] Wuhan BioBank, Wuhan, China.

* Contributed equally to this work.

** Corresponding authors. Emails: mli@uwaterloo.ca, lxin@bioinfor.com, bshan@bioinfor.com

Supplementary Information

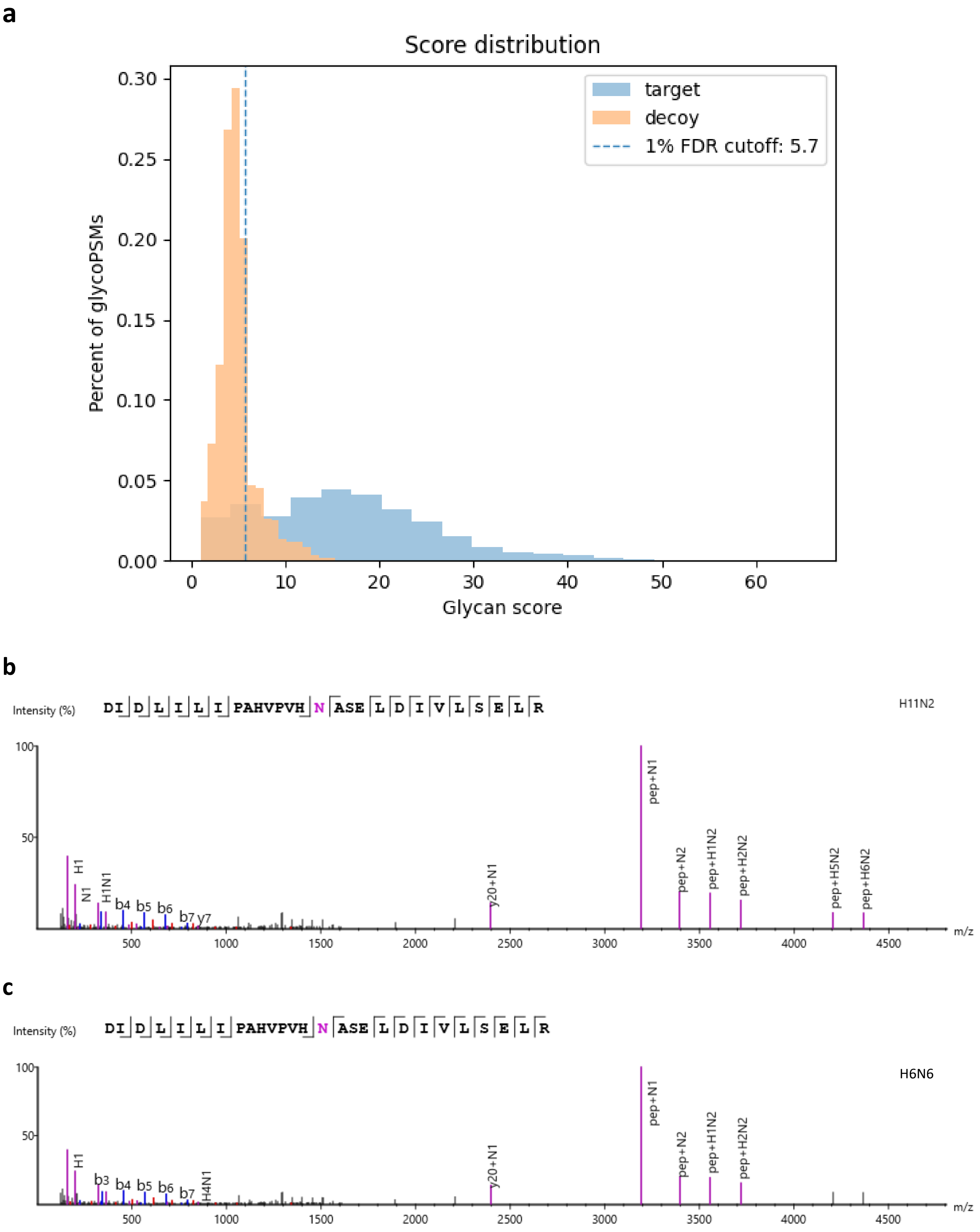

Supplementary Figure 1. (a) Score distributions of target and decoy glycoPSMs for FDR calculation on the mouse brain dataset (PXD005411) from Liu et al.[27]. (b, c) Two glycoPSMs identified by GlycanFinder and pGlyco3 from the same spectrum scan 56805, sample 2 (cwq_mix2-2_726.raw) of the fission yeast dataset (PXD005565) from Liu et al.[27]. The glycan identified by GlycanFinder, (HexNAc)2(Hex)11, has more supporting glycopeptide ions and has a high-mannose (HexNAc)2(Hex)n structure, which is commonly observed in the fission yeast species. Peptide-backbone b/y ions are highlighted in blue and red, respectively. Glycopeptide B/Y ions are highlighted in purple. (H: Hex; N: HexNAc).

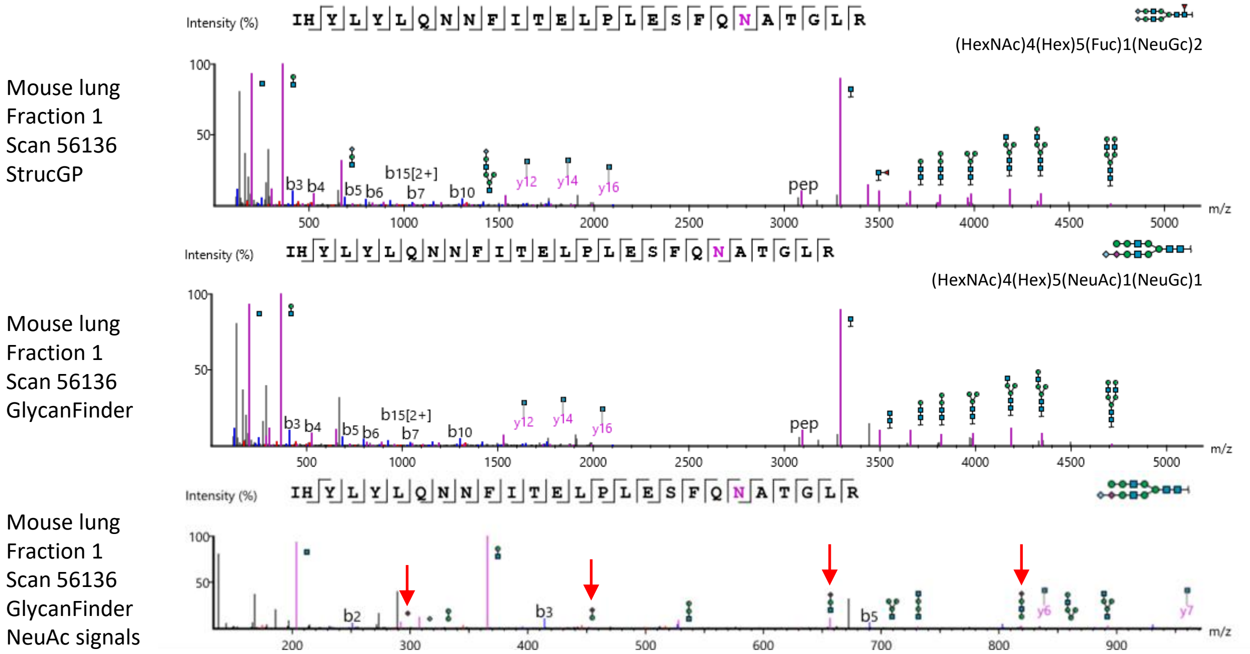

Supplementary Figure 2. Example glycoPSMs of different de novo glycans predicted by StrucGP and GlycanFinder on the same spectrum. StrucGP failed to detect NeuAc signals in the spectrum (red arrows).

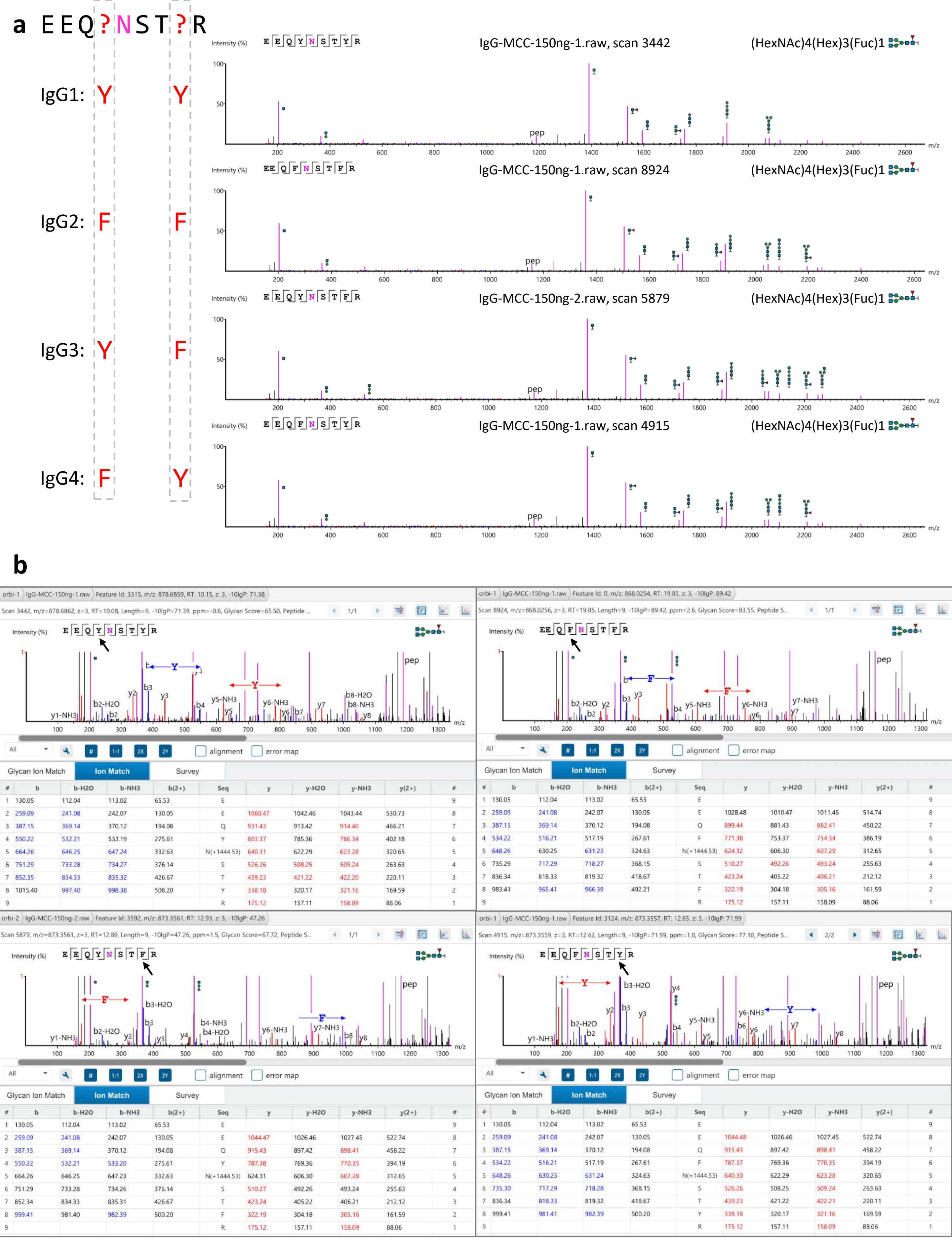

Supplementary Figure 3. N-linked glycopeptide analysis by GlycanFinder on the IgG Orbitrap dataset. (a) Example glycopeptide-spectrum matches of the four isomeric glycopeptides EEQ[Y/F]N(HexNAc4Hex3Fuc1)ST[Y/F]R on IgG1-4. (b) Zoom-in to the peptide backbone b/y ions of the glycopeptide-spectrum matches that could distinguish the four isomeric glycopeptides EEQ[Y/F]N(HexNAc4Hex3Fuc1)ST[Y/F]R on IgG1-4. The key ions b3/b4, y5/y6, b7/b8, and y1/y2 corresponding to Tyr (Y) and Phe (F) amino acids at the 4th and 8th positions are highlighted. The tables below the annotated spectra show the masses of the fragment ions and the matched b/y ions are highlighted in blue and red, respectively.

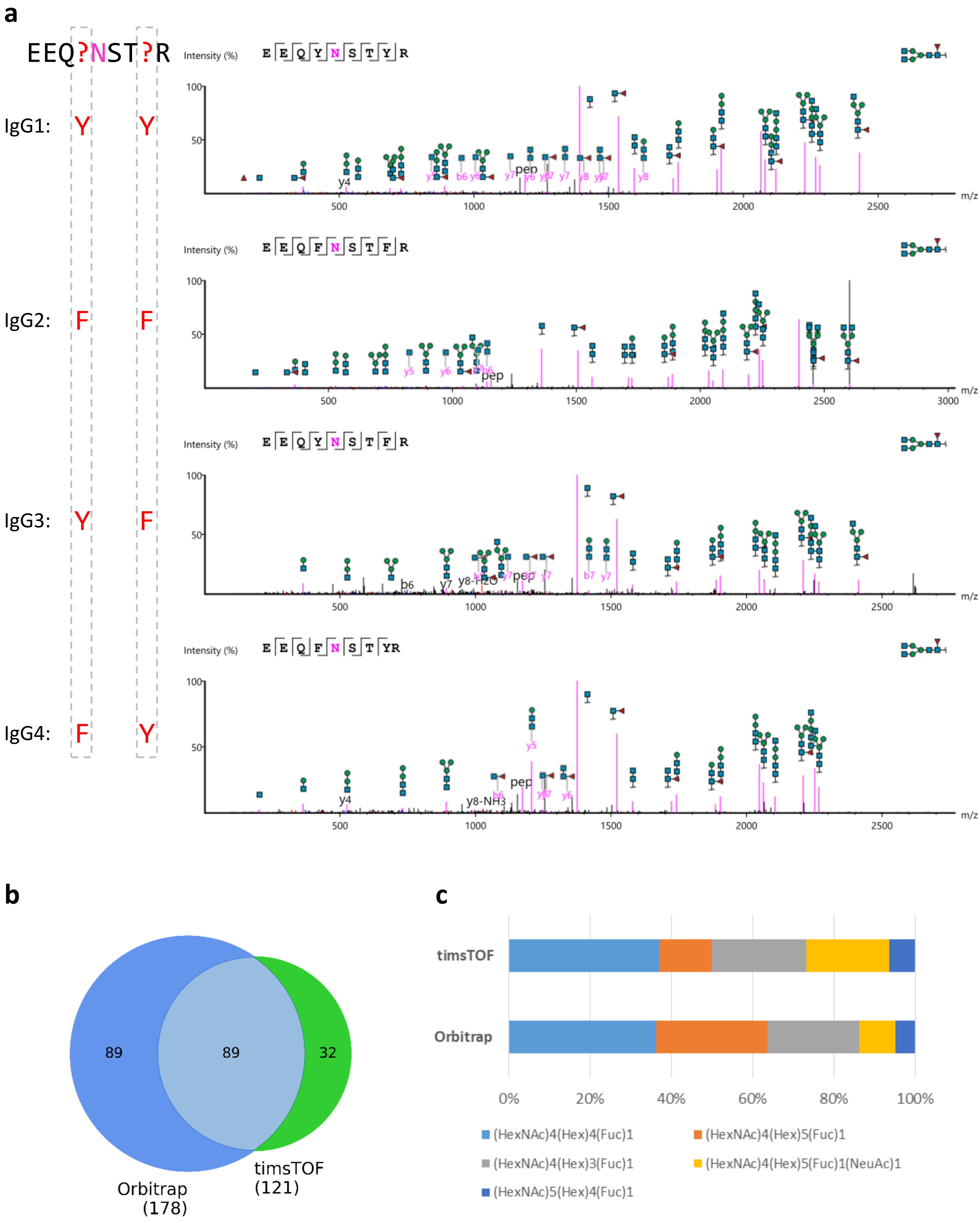

Supplementary Figure 4. N-linked glycopeptide analysis by GlycanFinder on the IgG timsTOF dataset. (a) Example g lycopeptide-spectrum matches that could distinguish isomeric peptides and glycans on four IgG1-4 subclasses. (b) Venn diagram of unique N-linked glycopeptides identified from the IgG Orbitrap and timsTOF datasets. (c) Relative quantification results of the top five most abundant N-linked glycans on the protein IgG2.

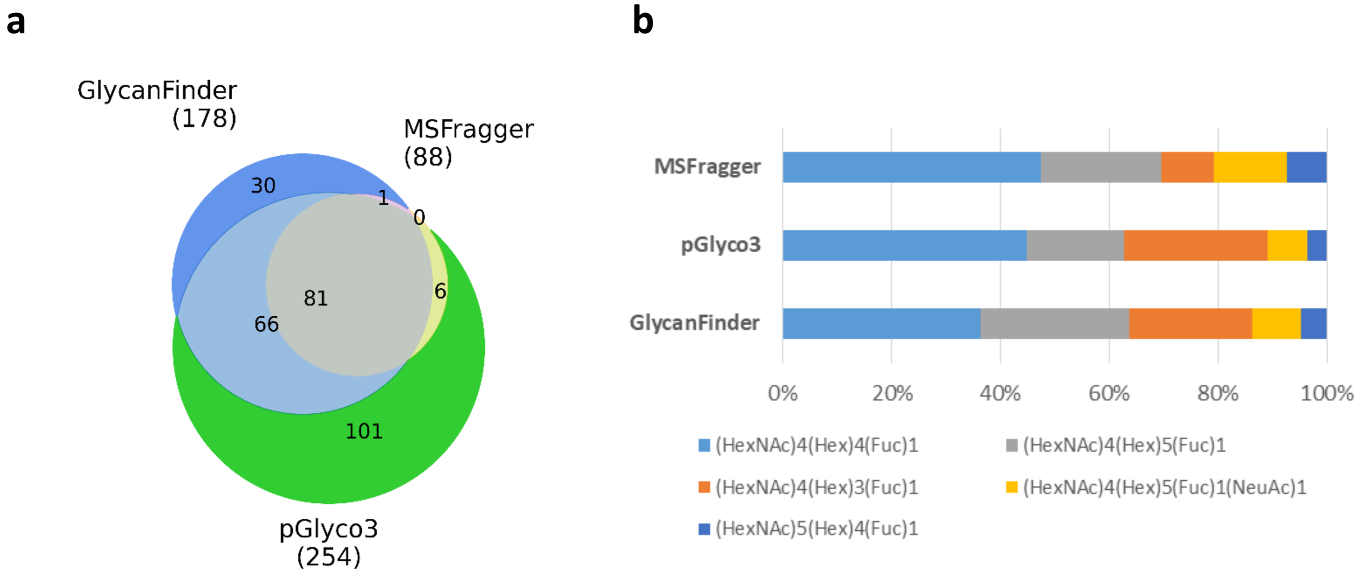

Supplementary Figure 5. Comparison of N-linked glycopeptide analysis results by GlycanFinder, pGlyco3, and MSFragger on the IgG Orbitrap dataset. (a) Venn diagram of unique N-linked glycopeptides identified by the three search engines. (b) Relative quantification results of the top five most abundant N-linked glycans on the protein IgG2.

a

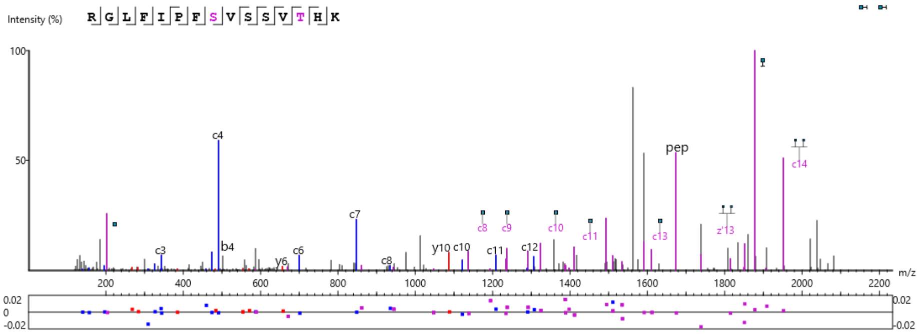

#	Theoretical m/z	Observed m/z	Moiety
1	204.09	204.09	60001_HexNAc1
2	588.30	588.30	y3_60001_HexNAc1
3	671.35	671.34	z'4_60001_HexNAc1
4	861.43	861.44	y6_60001_HexNAc1
5	944.48	944.48	z'7_60001_HexNAc1
6	1047.53	1047.53	y8_60001_HexNAc1
7	1138.63	1138.62	c8_60001_HexNAc1
8	1194.60	1194.61	y9_60001_HexNAc1
9	1234.59	1234.59	z'8_60001_HexNAc1_60001_HexN...
10	1237.69	1237.70	c9_60001_HexNAc1
11	1291.65	1291.66	y10_60001_HexNAc1
12	1324.73	1324.73	c10_60001_HexNAc1
13	1387.71	1387.72	z11_60001_HexNAc1
14	1388.72	1388.72	z'11_60001_HexNAc1
15	1397.68	1397.68	y9_60001_HexNAc1_60001_HexN...
16	1411.76	1411.76	[illegible]

b

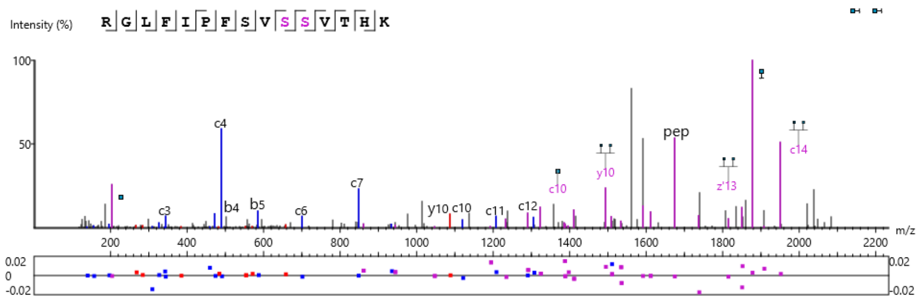

#	Theoretical m/z	Observed m/z	Moiety
1	204.09	204.09	1_HexNAc1
2	861.43	861.44	y6_1_HexNAc1
3	944.48	944.48	z'7_1_HexNAc1
4	1047.53	1047.53	y8_1_HexNAc1
5	1194.60	1194.61	y9_1_HexNAc1
6	1234.59	1234.59	z'8_1_HexNAc1_2_HexNAc1
7	1291.65	1291.66	y10_1_HexNAc1
8	1324.73	1324.73	c10_1_HexNAc1
9	1387.71	1387.72	z11_1_HexNAc1
10	1388.72	1388.72	z'11_1_HexNAc1
11	1397.68	1397.68	y9_1_HexNAc1_2_HexNAc1
12	1411.76	1411.76	[illegible]

Supplementary Figure 6. As there are often more than one O-glycosylation sites in a peptide sequence. GlycanFinder allows at most two O-linked glycans per peptide and considers internal fragment ions to determine the best glycosylation sites. In this example, the best candidate (a) has 4 more matched ions, y3-HexNAc1, z’4-HexNAc1, c8-HexNAc1 and c9-HexNAc1, than the candidate in (b).

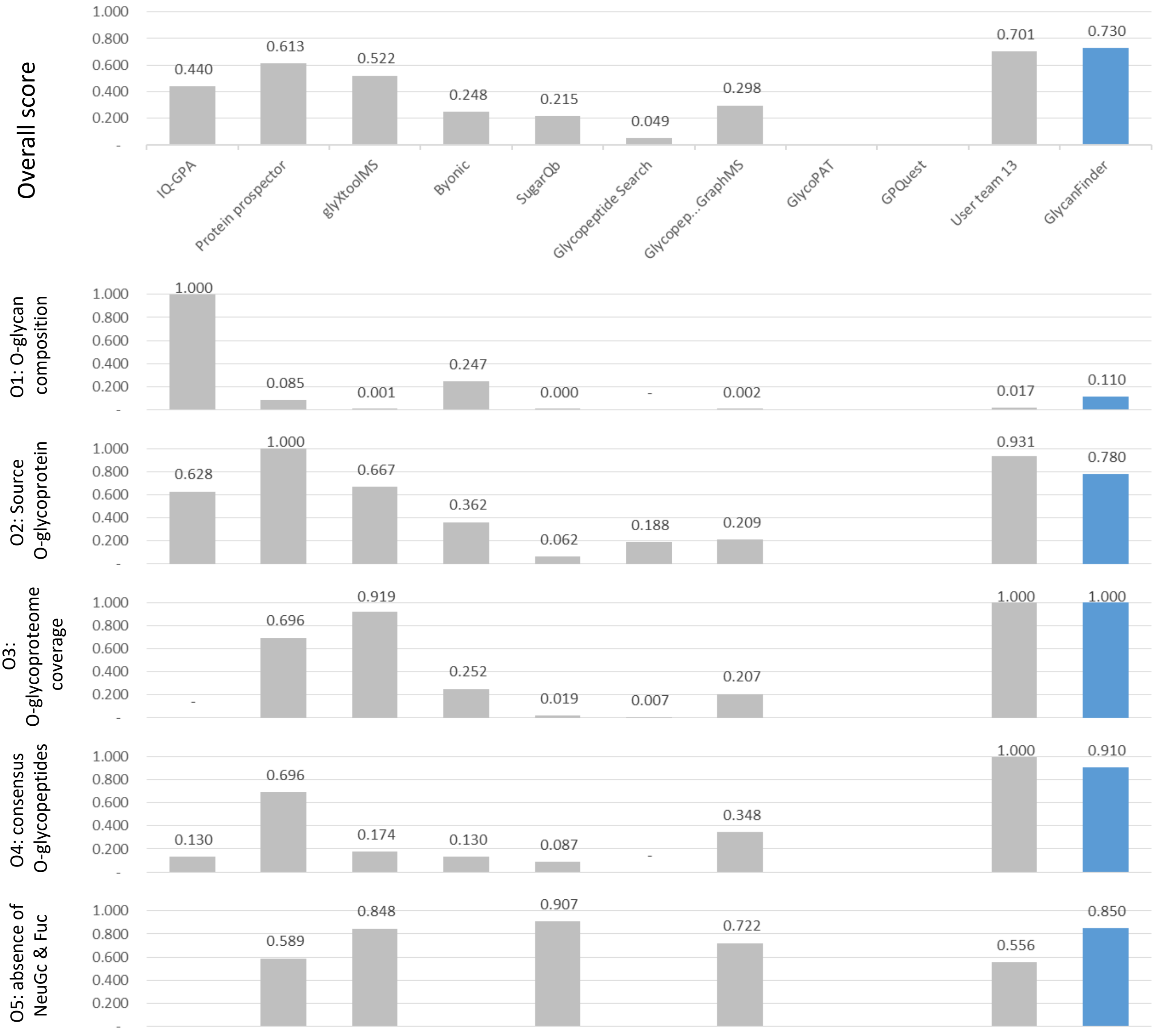

Supplementary Figure 7. Performance of O-linked glycopeptide database search engines on community-based evaluation benchmarks proposed in Kawahara et al.[3].

a

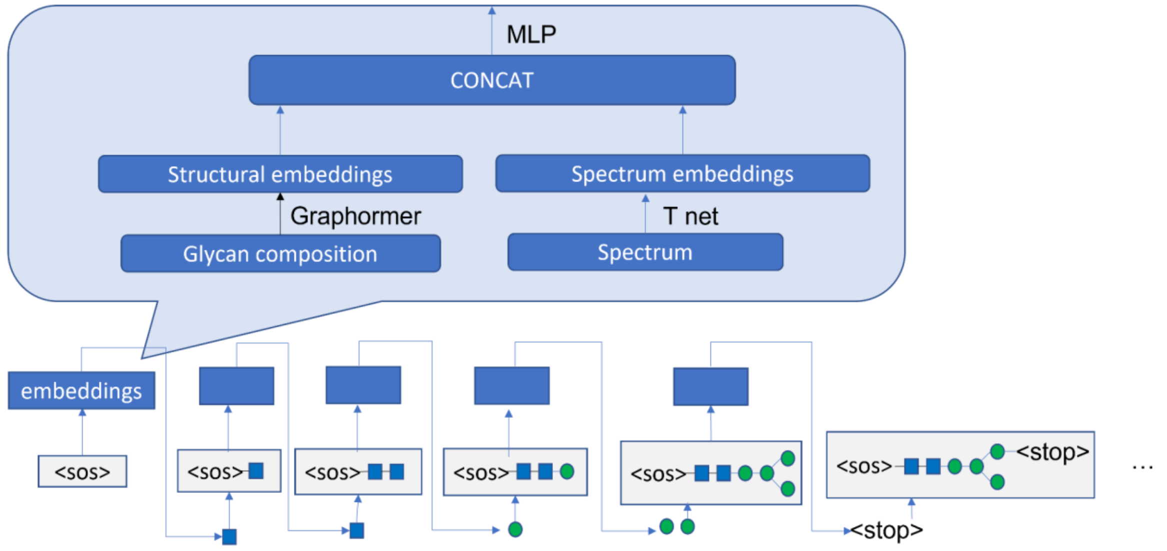

b

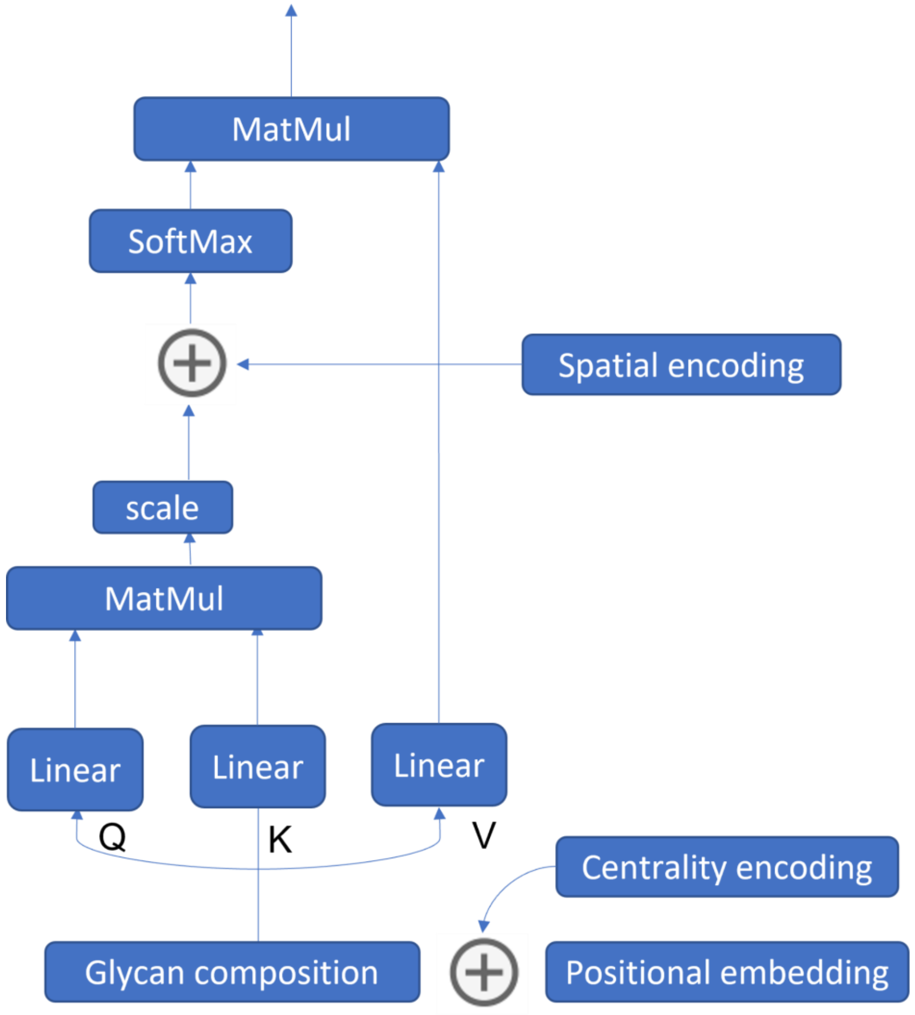

Supplementary Figure 8. Deep learning model for glycan de novo sequencing. (a) Our approach involves the extraction of information from both the spectrum and the current substructure at each time step. Each node within the substructure represents compositions derived from dynamic programming. For instance, <sos> in the initial time step consists of node features of two Hexes and three HexNAcs, the HexNAc node in the second time step consists of two Hexes and two HexNAcs. These combinations of monosaccharides are integrated into the substructure. The generation process concludes either when all monosaccharides in composition have been utilized or when all child nodes are connected by a <stop> sign. (b) We use Graphormer to encode structure at each time step. Each node in structure is labeled by breadth-first-search order starting from the root attached to the peptide. As a result we use positional embedding introduced in the transformer to encode the label and centrality encoding that encodes the in and out degrees of each node.
