# Supplementary material for: Glycopeptide database search and de novo sequencing with PEAKS GlycanFinder enable highly sensitive glycoproteomics
Source: Nat Commun. 2023 Jul 8;14:4046. doi: 10.1038/s41467-023-39699-5 (PMC10329677; doi:10.1038/s41467-023-39699-5)
Supplement: Supplementary file 3 — Reporting Summary [file 41467_2023_39699_MOESM3_ESM.pdf]

nature portfolio

Corresponding author(s): Ming Li

Last updated by author(s): May 23, 2023

Reporting Summary

Nature Portfolio wishes to improve the reproducibility of the work that we publish. This form provides structure for consistency and transparency in reporting. For further information on Nature Portfolio policies, see our Editorial Policies and the Editorial Policy Checklist.

Statistics

For all statistical analyses, confirm that the following items are present in the figure legend, table legend, main text, or Methods section.

n/a	Confirmed	
☒	☐	The exact sample size (*n*) for each experimental group/condition, given as a discrete number and unit of measurement
☒	☐	A statement on whether measurements were taken from distinct samples or whether the same sample was measured repeatedly
☐	☒	The statistical test(s) used AND whether they are one- or two-sided<br>*Only common tests should be described solely by name; describe more complex techniques in the Methods section.*
☒	☐	A description of all covariates tested
☒	☐	A description of any assumptions or corrections, such as tests of normality and adjustment for multiple comparisons
☒	☐	A full description of the statistical parameters including central tendency (e.g. means) or other basic estimates (e.g. regression coefficient) AND variation (e.g. standard deviation) or associated estimates of uncertainty (e.g. confidence intervals)
☐	☒	For null hypothesis testing, the test statistic (e.g. *F*, *t*, *r*) with confidence intervals, effect sizes, degrees of freedom and *P* value noted<br>*Give P values as exact values whenever suitable.*
☒	☐	For Bayesian analysis, information on the choice of priors and Markov chain Monte Carlo settings
☒	☐	For hierarchical and complex designs, identification of the appropriate level for tests and full reporting of outcomes
☐	☒	Estimates of effect sizes (e.g. Cohen's *d*, Pearson's *r*), indicating how they were calculated

Our web collection on statistics for biologists contains articles on many of the points above.

Software and code

Policy information about availability of computer code

Data collection	No software was used for data collection.
Data analysis	The Python implementation, training and testing datasets of the deep learning model for glycan de novo sequencing is available on our GitHub repository GlycoNovo [49] and can be accessed via the following link: https://github.com/zqq66/GlycoNovo. GlycanFinder is available at https://www.bioinfor.com/peaks-studio/, its documentation is provided in the Supplementary Data 7.

For manuscripts utilizing custom algorithms or software that are central to the research but not yet described in published literature, software must be made available to editors and reviewers. We strongly encourage code deposition in a community repository (e.g. GitHub). See the Nature Portfolio guidelines for submitting code & software for further information.

Data

Policy information about availability of data

All manuscripts must include a data availability statement. This statement should provide the following information, where applicable:

- Accession codes, unique identifiers, or web links for publicly available datasets
- A description of any restrictions on data availability
- For clinical datasets or third party data, please ensure that the statement adheres to our policy

Data Availability

The fission yeast and mouse datasets from Liu et al.[27] were downloaded from the PRIDE[48] repository with accession numbers:
PXD005565 [http://central.proteomexchange.org/cgi/GetDataset?ID=PXD005565] (yeast)
PXD005411 [https://proteomecentral.proteomexchange.org/cgi/GetDataset?ID=PXD005411] (mouse brain)
PXD005412 [https://proteomecentral.proteomexchange.org/cgi/GetDataset?ID=PXD005412] (mouse kidney)
PXD005413 [https://proteomecentral.proteomexchange.org/cgi/GetDataset?ID=PXD005413] (mouse heart)
PXD005553 [https://proteomecentral.proteomexchange.org/cgi/GetDataset?ID=PXD005553] (mouse liver)
PXD005555 [http://central.proteomexchange.org/cgi/GetDataset?ID=PXD005555] (mouse lung)
The HGI dataset HCD-EThCD-CID-MS/MS (file B) from Kawahara et al.[3] was downloaded from the PRIDE repository with accession number
PXD024101 [https://proteomecentral.proteomexchange.org/cgi/GetDataset?ID=PXD024101]
The IgG datasets generated in our study have been deposited to the PRIDE repository with accession number:
PXD039787 [https://www.ebi.ac.uk/pride/archive/projects/PXD039787]

Human research participants

Policy information about studies involving human research participants and Sex and Gender in Research.

Reporting on sex and gender	Not applicable
Population characteristics	Not applicable
Recruitment	Not applicable
Ethics oversight	Not applicable

Note that full information on the approval of the study protocol must also be provided in the manuscript.

Field-specific reporting

Please select the one below that is the best fit for your research. If you are not sure, read the appropriate sections before making your selection.

☒ Life sciences ☐ Behavioural & social sciences ☐ Ecological, evolutionary & environmental sciences

For a reference copy of the document with all sections, see nature.com/documents/nr-reporting-summary-flat.pdf

Life sciences study design

All studies must disclose on these points even when the disclosure is negative.

Sample size	Our study analyzed three previously published datasets (fission yeast, mouse, and HGI), which have been widely used by the research community to benchmark glycoproteomics softwares. For the new IgG dataset that we generated in this study, two biological replicates were used to assess the consistency of glycan quantification. We found that the glycan quantification profiles were consistent across these two replicates. The replicates were not meant to assess biological variation.
Data exclusions	No data were excluded in this study.
Replication	For the new IgG dataset that we generated in this study, two biological replicates were used to assess the consistency of glycan quantification. We found that the glycan quantification profiles were consistent across these two replicates. The replicates were not meant to assess biological variation.
Randomization	For all datasets used in this study, all available data were analyzed and no randomization was needed.
Blinding	There was no group allocation during data collection and analysis. All datasets were analyzed in the same manner. Hence, blinding was not relevant to this study.

Reporting for specific materials, systems and methods

We require information from authors about some types of materials, experimental systems and methods used in many studies. Here, indicate whether each material, system or method listed is relevant to your study. If you are not sure if a list item applies to your research, read the appropriate section before selecting a response.

Materials & experimental systems

n/a	Involved in the study
☒	☐ Antibodies
☒	☐ Eukaryotic cell lines
☒	☐ Palaeontology and archaeology
☒	☐ Animals and other organisms
☒	☐ Clinical data
☒	☐ Dual use research of concern

Methods

n/a	Involved in the study
☒	☐ ChIP-seq
☒	☐ Flow cytometry
☒	☐ MRI-based neuroimaging
