# Supplementary material for: Glycopeptide database search and de novo sequencing with PEAKS GlycanFinder enable highly sensitive glycoproteomics
Source: Nat Commun. 2023 Jul 8;14:4046. doi: 10.1038/s41467-023-39699-5 (PMC10329677; doi:10.1038/s41467-023-39699-5)
Supplement: Supplementary file 4 — Description of Additional Supplementary Files [file 41467_2023_39699_MOESM4_ESM.pdf]

Description of Additional Supplementary Files

File Name: Supplementary Data 1
Description: Running time and glycoPSMs identified by GlycanFinder, pGlyco3, MetaMorpheus, and MSFragger on the fission yeast dataset from Liu et al.[27]

File Name: Supplementary Data 2
Description: Glycan de novo sequencing results by GlycanFinder and StrucGP on the dataset of five mouse tissues from Liu et al.[27]

File Name: Supplementary Data 3
Description:. glycoPSMs identified by GlycanFinder, pGlyco3, and MSFragger on the IgG Orbitrap dataset.

File Name: Supplementary Data 4
Description: glycoPSMs identified by GlycanFinder on the IgG timsTOF dataset.

File Name: Supplementary Data 5
Description: N-linked and O-linked evaluation results and glycoPSMs identified by GlycanFinder from the HCD-EThcD-CID dataset from the community-based evaluation study by Kawahara et al.[3]

File Name: Supplementary Data 6
Description: Protein, glycan databases, and a Python script to calculate the evaluation criteria for the HCD-EThcD-CID dataset from the community-based evaluation study by Kawahara et al.[3]

File Name: Supplementary Data 7
Description: Documentation of GlycanFinder.
