# Supplementary material for: Glycopeptide database search and de novo sequencing with PEAKS GlycanFinder enable highly sensitive glycoproteomics
Source: Nat Commun. 2023 Jul 8;14:4046. doi: 10.1038/s41467-023-39699-5 (PMC10329677; doi:10.1038/s41467-023-39699-5)
Supplement: Supplementary file 11 — Supplementary Data 7 [file 41467_2023_39699_MOESM11_ESM.pdf]

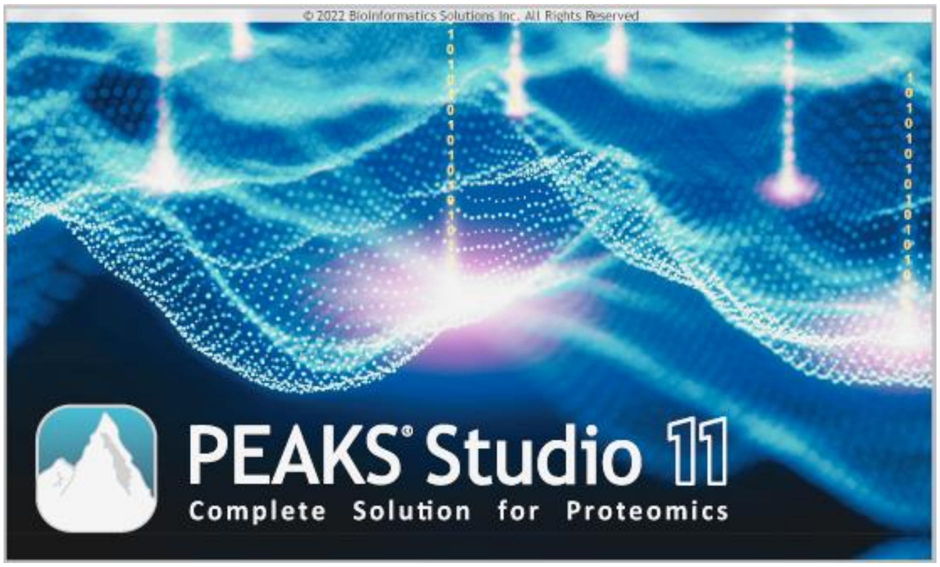

PEAKS STUDIO 11 USER MANUAL

January 4, 2023

Table of Contents

1. Overview 1
  1.1 How to Use This Manual 1
  1.2 What is PEAKS Studio 11? 1
  1.3 Installation and Activation 1
    1.3.1 System Requirements 1
    1.3.2 Installation on a Windows operating system 2
    1.3.3 Activation 2
    1.3.4 License Key descriptions 3
  1.4 Supported Data Formats 5
  1.5 Quick Walkthrough 5
    1.5.1 Start Page and Sample Project 5
    1.5.2 Setting up a new project 6
    1.5.3 Monitoring analysis and task cancellation 11
    1.5.4 Project Tree Hierarchy 14
    1.5.5 Analysis and Sample deletion 15
    1.5.6 Running additional analyses in existing project and modifying analysis 15
    1.5.7 Project opening and closing 16
  1.6 Import PEAKS Online Project 16
2. Configuration 18
  2.1 General Configuration - Default Directories Preferences 18
  2.2 Enzyme Configuration 19
  2.3 Instrument Configuration 20
  2.4 PTM Configuration 21
  2.5 TMT/iTRAQ Q Method Configuration 23
  2.6 SILAC Q Method Configuration 25
  2.7 Database Configuration 26
  2.8 Taxonomy Configuration 28
  2.9 Spectral Library Configuration 29
  2.10 Glycan Database Configuration 30
  2.11 Workflow Configuration - Saved Workflows 31
  2.12 Performance Configuration 32
3. Data View & Data Refine View 33
  3.1 Data Refinement - MS View 33
    3.1.1 Data Refinement - MS View - timsTOF Instruments 34
    3.1.2 Data Refinement - MS View - DIA Acquisition 35
  3.2 Data Refinement - MS/MS View 35
    3.2.1 Data Refinement - MS/MS View - timTOF Instruments 36
  3.3 Data Refinement - LC/MS View 37
    3.3.1 Additional LC/MS Controls 38
      3.3.1.1 Noise Level 38
      3.3.1.2 Show/Hide Features 38
      3.3.1.3 Locate M/Z and Retention Time 39

3.3.1.4 Show/Hide MS/MS Spectra 39
3.3.1.5 Show/Hide Markers 40
3.3.1.6 Intensity View 42
3.3.1.7 Navigation History 43
3.3.1.8 Export LC/MS 43
3.3.2 Data Refinement - LC/MS View - timsTOF Instruments 43
3.3.3 Data Refinement - LC/MS View - DIA Acquisition 44
3.4 Data View 44
3.5 Project Wizard Data Refine 45
3.6 Data Exports 45
4. Denovo peptide sequencing 46
4.1 De Novo Analysis Workflow and Parameter Settings 46
4.2 Understanding PEAKS De Novo Sequencing Results 49
4.3 Denovo result - Summary tab 49
4.4 De novo result - De novo View 50
4.4.1 De novo View - De novo table 50
4.4.2 De novo View - De novo table controls 51
4.4.2.1 De novo table controls - Sample Selection 51
4.4.2.2 De novo table controls - Pagination 51
4.4.2.3 De novo table controls - Mass Tag Filter 51
4.4.2.4 De novo table controls - Search function 51
4.4.2.5 De novo table controls - Table exporting 52
4.4.3 De novo View - De novo All Candidates 52
4.4.4 PSM selection 52
4.4.4.1 Show spectrum in Data View 53
4.4.4.2 Show spectrum in LC/MS View 53
4.4.4.3 Show Raw Spectrum View 53
4.4.5 Annotated Spectrum Chart 53
4.4.5.1 Annotated Spectrum Chart - Chart Navigation 53
4.4.5.2 Annotated Spectrum Chart - Spectrum Annotation Settings 54
4.4.5.3 Annotated Spectrum Chart - Additional Chart Controls 55
4.4.6 Ion Match Table 55
4.4.7 Survey Chart 56
4.4.8 LC/MS Snapshot 56
4.4.9 Precursor Profile 57
4.5. De novo result - Feature View 57
4.5.1 Feature View - Feature table 57
4.5.2 Feature View - Feature table controls 58
4.5.2.1 Feature table controls - Sample Selection 58
4.5.2.2 Feature table controls - Pagination 58
4.5.2.3 Feature table controls - Feature View Filter 58
4.5.2.4 Feature table controls - Search function 59
4.4.2.5 Feature table controls - Table exporting 59
4.4.2.6 Feature table - PSM visualization 59

4.6. De novo result - Exporting 60
5. Deepnovo Peptidome 61
5.1 Deepnovo Peptidome Analysis Workflow and Parameter Settings........ 61
5.2 Understanding PEAKS Deep Novo Results 63
5.3 Deep Novo result - Summary tab........ 64
5.3.1 Deep Novo result - Summary tab - ALC versus precursor mass error scatterplot 64
5.3.2 Deep Novo result - Summary tab - Distribution of local residue confidence 65
5.3.3 Deep Novo result - Summary tab - ALC RT distribution of first fraction........ 65
5.3.4 Deep Novo result - Summary tab - Peptide RT versus Predicted RT scatter plot 65
5.4 Deep Novo result - Deep Novo View 66
5.4.1 Deep Novo View - Deep Novo table 67
5.4.2 Deep Novo View - Deep Novo table controls 67
5.4.2.1 Deep Novo table controls - Sample Selection........ 68
5.4.2.2 Deep Novo table controls - Pagination 68
5.4.2.3 Deep Novo table controls - Mass Tag Filter 68
5.4.2.4 Deep Novo table controls - Search function 68
5.4.2.5 Deep Novo table controls - Table exporting 68
5.4.3 Deep Novo View - Deep Novo All Candidates........ 69
5.4.4 PSM selection 69
5.4.4.1 Show spectrum in Data View 69
5.4.4.2 Show spectrum in LC/MS View 69
5.4.4.3 Show Raw Spectrum View 69
5.4.5 Annotated Spectrum Chart 69
5.4.5.1 Annotated Spectrum Chart - Chart Navigation........ 70
5.4.5.2 Annotated Spectrum Chart - Spectrum Annotation Settings 70
5.4.5.3 Annotated Spectrum Chart - Additional Chart Controls 71
5.4.5.4 Annotated Spectrum Chart - Mirror Plot 72
5.4.6 Ion Match Table 72
5.4.6.1 Ion Match Table - Predicted matching table........ 73
5.4.7 Survey Chart 73
5.4.8 LC/MS Snapshot 73
5.4.9 Precursor Profile 74
5.5. Deep Novo result - Exporting........ 74
5.6 Peptide Search result 75
5.6.1 Peptide Search result - Summary tab 75
5.6.1.1 Peptide Search result - Figure 1. False Discovery Rate (FDR) curve. 76
5.6.1.2 Peptide Search result - Table 1. Statistics of data........ 76
5.6.1.3 Peptide Search result - Figure 2. PSM score distribution 77
5.6.1.4 Peptide Search result - Result filtration parameters 77
5.6.1.5 Peptide Search result - RT Figures 77
5.6.1.6 Peptide Search result - Boxplot........ 78
5.6.1.7 Peptide Search result - Other Information 79
5.6.2 Peptide Search result - Peptide Tab........ 79
5.6.3 Peptide Table controls 81

5.6.3.1 Peptide Table controls - Pagination 81
5.6.3.2 Peptide Table controls - View Filter 81
5.6.3.3 Peptide Table controls - Optional Columns 83
5.6.3.4 Peptide Table controls - Search function 83
5.6.4 Feature selection breadcrumb 83
5.6.5 Peptide View - PSM selection 84
5.6.5.1 Show spectrum in Data View 84
5.6.5.2 Show spectrum in LC/MS View 84
5.6.5.3 Show Raw Spectrum View 84
5.6.6 Peptide View - Annotated Spectrum Chart 85
5.6.6.1 Annotated Spectrum Chart - Chart Navigation 85
5.6.6.2 Annotated Spectrum Chart - Spectrum Annotation Settings 85
5.6.6.3 Annotated Spectrum Chart - Additional Chart Controls 86
5.6.6.4 Annotated Spectrum Chart - Mirror Plot 87
5.6.6.5 Peptide View - Ion Match Table 87
5.6.7 Peptide View - LC/MS Snapshot 88
5.6.8 Peptide View - Precursor Profile 88
5.7 Peptide Search result - Exporting 89
5.7.1 Peptide Search result - Exporting - comma separated values (CSV) format 89
5.7.2 Peptide Search result - Exporting - HTML format 89
6. Peptide, PTM, and Mutation Identification (PEAKS DB, PEAKS PTM, and SPIDER) 90
6.1 Database Search Workflow and Parameter Settings 90
6.2 Understanding PEAKS DB Search Results 94
6.3 PEAKS DB Search result - Summary tab 95
6.3.1 PEAKS DB Search result - Figure 1. False Discovery Rate (FDR) curve. 96
6.3.2 PEAKS DB Search result - Figure 2. PSM score distribution 97
6.3.3 PEAKS DB Search result - Table 1. Statistics of data 97
6.3.4 PEAKS DB Search result - Figure 3. Sample overlap for Proteins and Peptides 98
6.3.5 PEAKS DB Search result - Figures 4 and 5. Distribution of peptide feature detection 99
6.3.6 PEAKS DB Search result - Result filtration parameters 100
6.3.7 PEAKS DB Search result - Experiment Control 101
6.3.8 PEAKS DB Search result - Other Information 102
6.4 PEAKS DB Search result - Proteins View 102
6.4.1 Proteins View - Protein Table controls 103
6.4.1.1 Protein Table controls - Protein View Filters 103
6.4.1.2 Protein Table controls - Protein count 105
6.4.1.3 Protein Table controls - Optional columns 105
6.4.1.4 Protein Table controls - Protein Table Search Function 105
6.4.1.5 Protein Table controls - Protein Table Export 106
6.4.2 Proteins View - Protein Table 106
6.4.3 Proteins View - Coverage Pane 107
6.4.3.1 Protein Sequence Coverage 107
6.4.3.2 Coverage Control Panel 108
6.4.3.3 Proteins View - Protein Tools 109

6.4.3.4 Proteins View - Supporting Peptides 118
6.4.3.5 Proteins View - De novo Tags 118
6.5 PEAKS DB Search result - Peptide View 119
6.5.1 Peptide Table 119
6.5.2 Peptide Table controls 120
6.5.2.1 Peptide Table controls - Pagination 120
6.5.2.2 Peptide Table controls - View Filter 121
6.5.2.3 Peptide Table controls - Optional Columns 121
6.5.2.4 Peptide Table controls - Search function 121
6.5.3 Feature selection breadcrumb 122
6.5.4 PSM selection 123
6.5.4.1 Protein jump button 123
6.5.4.2 Show spectrum in Data View 123
6.5.4.3 Show spectrum in LC/MS View 123
6.5.4.4 Show Raw Spectrum View 123
6.5.5 Annotated Spectrum Chart 123
6.5.5.1 Annotated Spectrum Chart - Chart Navigation 124
6.5.5.2 Annotated Spectrum Chart - Spectrum Annotation Settings 124
6.5.5.3 Annotated Spectrum Chart - Additional Chart Controls 125
6.5.6 Ion Match Table 125
6.5.7 Survey Chart 126
6.5.8 LC/MS Snapshot 126
6.5.9 Precursor Profile 127
6.6 PEAKS DB Search result - De novo only View 128
6.7 PEAKS DB Search result - Feature View 128
6.8 PEAKS DB Search result - Exporting 129
6.8.1 PEAKS DB Search result - Exporting - comma separated values (CSV) format 130
6.8.2 PEAKS DB Search result - Exporting - HTML format 131
6.8.3 PEAKS DB Search result - Exporting - pepXML and mzIdentML 131
6.8.4 PEAKS DB Search result - Exporting - Spectral Library generation 132
6.9 PEAKS PTM Finder 132
6.10 SPIDER 133
7. DIA Workflow (Spectral Library, DB Search, De Novo, Quantification) 134
7.1 DIA Identification Analysis Workflow and Parameter Settings 134
7.1.1 DIA Identification - Spectral Library Search parameters 134
7.1.2 DIA Identification - DIA DB Search parameters 135
7.1.3 DIA Identification - DIA De Novo parameters 137
7.1.4 DIA Quantification (DIA LFQ) Analysis Workflow and Parameter Settings 138
7.2 Understanding PEAKS Spectral Library Search Results 144
7.3 Spectral Library - Summary View 144
7.3.1 Spectral Library result - Figure 1. False Discovery Rate (FDR) curve. 145
7.3.2 Spectral Library result - Figure 2. RT Calibration 146
7.3.3 Spectral Library result - Boxplot for RT change 146
7.3.3 Spectral Library result - Table 1. Statistics of data 147

7.3.4 Spectral Library result - Result filtration parameters 147
7.3.5 Spectral Library result - Experiment Control 148
7.4 Spectral Library result - Proteins View 149
7.4.1 Proteins View - Protein Table controls 149
7.4.1.1 Protein Table controls - Protein View Filters 150
7.4.1.2 Protein Table controls - Protein count 151
7.4.1.3 Protein Table controls - Optional columns 151
7.4.1.4 Protein Table controls - Protein Table Search Function 151
7.4.1.5 Protein Table controls - Protein Table Export 152
7.4.2 Proteins View - Protein Table 152
7.4.3 Proteins View - Coverage Pane 153
7.4.3.1 Coverage Pane - Protein Sequence Coverage 153
7.4.3.2 Coverage Pane - Coverage Control Panel 154
7.4.3.3 Proteins View - Protein Tools 155
7.4.3.4 Proteins View - Supporting Peptides 155
7.5 Spectral Library Result - Peptide View 156
7.5.1 Peptide View - Peptide Table 157
7.5.2 Peptide View - Peptide Table controls 158
7.5.2.1 Peptide Table controls - Pagination 158
7.5.2.2 Peptide Table controls - View Filter 158
7.5.2.3 Peptide Table controls - Optional Columns 159
7.5.2.4 Peptide Table controls - Search function 159
7.5.3 Peptide View - Feature selection breadcrumb 160
7.5.4 Peptide View - PSM selection 160
7.5.4.1 Peptide View - Protein jump button 160
7.5.5 Peptide View - Annotated Spectrum Chart 161
7.5.5.1 Annotated Spectrum Chart - Chart Navigation 161
7.5.5.2 Annotated Spectrum Chart - Spectrum Annotation Settings 161
7.5.5.3 Annotated Spectrum Chart - Additional Chart Controls 162
7.5.5.4 Annotated Spectrum Chart - Mirror Plot 163
7.5.6 Peptide View - Ion Match Table 163
7.5.6.1 Ion Match Table - Library matching table 163
7.5.7 Peptide View - LC/MS Snapshot 164
7.5.8 Peptide View - Precursor Profile 164
7.5.9 Peptide View - Fragment Ion XIC 165
7.6 Spectral Library Search Result - Exporting 166
7.6.1 Spectral Library Search - Exporting - comma separated values (CSV) format 166
7.6.2 Spectral Library Search - Exporting - HTML format 166
7.7 Understanding PEAKS DIA Database Search Results 166
7.8 DIA Database Search - Summary View 167
7.8.1 DIA Database Search - Figure 1. False Discovery Rate (FDR) curve. 168
7.8.2 DIA Database Search result - Figure 2. RT Calibration 169
7.8.3 DIA Database Search result - Boxplot for RT change 169
7.8.4 DIA Database Search result - Table 1. Statistics of data 169

7.8.5 DIA Database Search result - Result filtration parameters 170
7.8.6 DIA Database Search result - Experiment Control 171
7.9 DIA Database Search - Proteins View........ 171
7.9.1 Proteins View - Protein Table controls 172
7.9.1.1 Protein Table controls - Protein View Filters 172
7.9.1.2 Protein Table controls - Protein count........ 174
7.9.1.3 Protein Table controls - Optional columns 174
7.9.1.4 Protein Table controls - Protein Table Search Function 174
7.9.1.5 Protein Table controls - Protein Table Export........ 175
7.9.2 Proteins View - Protein Table........ 175
7.9.3 Proteins View - Coverage Pane 176
7.9.3.1 Coverage Pane - Protein Sequence Coverage........ 176
7.9.3.2 Coverage Pane - Coverage Control Panel 177
7.9.3.3 Proteins View - Protein Tools........ 178
7.9.3.4 Proteins View - Supporting Peptides 178
7.10 DIA Database Search Result - Peptide View........ 179
7.10.1 Peptide View - Peptide Table 179
7.10.2 Peptide View - Peptide Table controls........ 180
7.10.2.1 Peptide Table controls - Pagination 181
7.10.2.2 Peptide Table controls - View Filter 181
7.10.2.3 Peptide Table controls - Optional Columns 182
7.10.2.4 Peptide Table controls - Search function........ 182
7.10.3 Peptide View - Feature selection breadcrumb 182
7.10.4 Peptide View - PSM selection 183
7.10.4.1 Peptide View - Protein jump button 183
7.10.5 Peptide View - Annotated Spectrum Chart........ 184
7.10.5.1 Annotated Spectrum Chart - Chart Navigation........ 184
7.10.5.2 Annotated Spectrum Chart - Spectrum Annotation Settings 184
7.10.5.3 Annotated Spectrum Chart - Additional Chart Controls 185
7.10.5.4 Annotated Spectrum Chart - Mirror Plot 186
7.10.6 Peptide View - Ion Match Table........ 186
7.10.6.1 Ion Match Table - Library matching table 186
7.10.7 Peptide View - LC/MS Snapshot........ 187
7.10.8 Peptide View - Precursor Profile 187
7.10.9 Peptide View - Fragment Ion XIC 188
7.11 DIA Database Search Result - Exporting 189
7.11.1 DIA Database Search - Exporting - comma separated values (CSV) format 189
7.11.2 DIA Database Search - Exporting - HTML format........ 189
7.12 Understanding PEAKS DIA De Novo Results 189
7.13 DIA De Novo - Summary View........ 190
7.14 DIA De Novo result - De Novo View........ 191
7.14.1 De Novo View - De novo table 191
7.14.2 De Novo View - De Novo table controls........ 192
7.14.2.1 De Novo table controls - Sample Selection........ 192

7.14.2.2 De Novo table controls - Pagination ... 192
7.14.2.3 De Novo table controls - Mass Tag Filter ... 192
7.14.2.4 De Novo table controls - Search function ... 192
7.14.2.5 De Novo table controls - Table exporting ... 193
7.14.3 De Novo View - Deep Novo All Candidates ... 193
7.14.4 PSM selection ... 193
7.14.4.1 Show spectrum in Data View ... 194
7.14.4.2 Show spectrum in LC/MS View ... 194
7.14.4.3 Show Raw Spectrum View ... 194
7.14.5 Annotated Spectrum Chart ... 194
7.14.5.1 Annotated Spectrum Chart - Chart Navigation ... 194
7.14.5.2 Annotated Spectrum Chart - Spectrum Annotation Settings ... 195
7.14.5.3 Annotated Spectrum Chart - Additional Chart Controls ... 196
7.14.5.4 Annotated Spectrum Chart - Mirror Plot ... 196
7.14.5.5 Annotated Spectrum Chart - Pseudo Scan ... 197
7.14.6 DIA De Novo Ion Match Table ... 197
7.14.6.1 Ion Match Table - Predicted matching table ... 197
7.14.7 DIA De Novo View - LC/MS Snapshot ... 198
7.14.8 DIA De Novo View - Precursor Profile ... 198
7.14.9 DIA De Novo View - Fragment Ion XIC ... 199
7.15 DIA De Novo result - Exporting ... 199
7.16 DIA Label Free Quantification result ... 200
7.17 Understanding DIA LFQ Results ... 200
7.18. DIA LFQ - Summary View ... 201
7.18.1 Summary View - Protein Filters ... 201
7.18.2 Summary View - Notes ... 201
7.18.3 Summary View - Heat Map ... 203
7.18.4 Summary View - Density-Ratio Plot ... 205
7.18.5 Summary View - Sample Correlation plot ... 206
7.18.6 Summary View - Group Correlation plot ... 207
7.18.7 Summary View - Volcano plot ... 208
7.18.8 Summary View - RT and m/z Shift Distribution ... 210
7.18.9 Summary View - Missing Value percentage chart ... 210
7.18.10 Summary View - Filtration parameters and statistics ... 211
7.18.11 Summary View - Search parameters ... 211
7.19 LFQ - Protein View ... 212
7.19.1 Proteins View - Protein Table controls ... 212
7.19.1.1 Protein Table controls - Protein View Filters ... 212
7.19.1.2 Protein Table controls - Protein count ... 214
7.19.1.3 Protein Table controls - Optional columns ... 214
7.19.1.4 Protein Table controls - Protein Table Search Function ... 214
7.19.1.5 Protein Table controls - Protein Table Export ... 214
7.19.2 Proteins View - Protein Table ... 215
7.19.3 Proteins View - Coverage Pane ... 216

7.19.3.1 Protein Sequence Coverage 216
7.19.3.2 Coverage Control Panel 217
7.19.3.3 Proteins View - Protein Tools 219
7.19.3.4 Proteins View - Supporting Peptides 219
7.20 DIA LFQ result - Peptides View 221
7.20.1 Peptides View - Peptide Table 221
7.20.2 Peptide Table controls 222
7.20.2.1 Peptide Table controls - Pagination 222
7.20.2.2 Peptide Table controls - View Filter 223
7.20.2.3 Peptide Table controls - Optional Columns 224
7.20.2.4 Peptide Table controls - Search function 224
7.20.3 Feature vector selection 224
7.20.4 Protein jump button 225
7.20.5 Feature Details 225
7.21 DIA LFQ Exporting 225
7.21.1 LFQ result - Exporting - comma separated values (CSV) format 226
7.21.2 LFQ result - Exporting - HTML format 226
8. Label Free Quantification (LFQ) 227
8.1 Label Free Quantification Analysis Workflow and Settings 227
8.2 Understanding LFQ Results 235
8.3 LFQ - Summary View 236
8.3.1 Summary View - Protein Filters 236
8.3.2 Summary View - Notes 236
8.3.3 Summary View - Heat Map 238
8.3.4 Summary View - Density-Ratio Plot 240
8.3.5 Summary View - Sample Correlation plot 241
8.3.6 Summary View - Group Correlation plot 242
8.3.7 Summary View - Volcano plot 244
8.3.8 Summary View - RT and m/z Shift Distribution 245
8.3.9 Summary View - Missing Value percentage chart 245
8.3.10 Summary View - Filtration parameters and statistics 246
8.3.11 Summary View - Search parameters 246
8.4 LFQ - Protein View 247
8.4.1 Proteins View - Protein Table controls 247
8.4.1.1 Protein Table controls - Protein View Filters 247
8.4.1.2 Protein Table controls - Protein count 249
8.4.1.3 Protein Table controls - Optional columns 249
8.4.1.4 Protein Table controls - Protein Table Search Function 249
8.4.1.5 Protein Table controls - Protein Table Export 249
8.4.2 Proteins View - Protein Table 250
8.4.3 Proteins View - Coverage Pane 251
8.4.3.1 Protein Sequence Coverage 251
8.4.3.2 Coverage Control Panel 252
8.4.3.3 Proteins View - Protein Tools 254

8.4.3.4 Proteins View - Supporting Peptides ... 258
8.5 LFQ result - Peptides View ... 260
8.5.1 Peptides View - Peptide Table ... 260
8.5.2 Peptide Table controls ... 261
8.5.2.1 Peptide Table controls - Pagination ... 261
8.5.2.2 Peptide Table controls - View Filter ... 262
8.5.2.3 Peptide Table controls - Optional Columns ... 262
8.5.2.4 Peptide Table controls - Search function ... 263
8.5.3 Feature vector selection ... 263
8.5.4 Protein jump button ... 264
8.5.5 Feature Details ... 264
8.5.6 Sample Features ... 264
8.5.7 RT Alignment ... 265
8.6 LFQ Exporting ... 265
8.6.1 LFQ result - Exporting - comma separated values (CSV) format ... 266
8.6.2 LFQ result - Exporting - HTML format ... 266
9. Isobaric Labelling Quantification (TMT / iTRAQ) ... 267
9.1 Isobaric Labelling Quantification Analysis Workflow and Settings ... 267
9.2 Understanding TMT/iTRAQ Results ... 278
9.3. TMT/iTRAQ - Summary View ... 279
9.3.1 Summary View - Protein Filters ... 279
9.3.2 Summary View - Notes ... 279
9.3.3 Summary View - Heat Map ... 281
9.3.4 Summary View - Volcano plot ... 283
9.3.5 Summary View - Filtration parameters and statistics ... 284
9.3.6 Summary View - Search parameters ... 284
9.4 TMT/iTRAQ - Protein View ... 285
9.4.1 Proteins View - Protein Table controls ... 285
9.4.1.1 Protein Table controls - Protein View Filters ... 286
9.4.1.2 Protein Table controls - Protein count ... 287
9.4.1.3 Protein Table controls - Protein Table Search Function ... 287
9.4.1.4 Protein Table controls - Protein Table Export ... 287
9.4.2 Proteins View - Protein Table ... 287
9.4.3 Proteins View - Coverage Pane ... 288
9.4.3.1 Protein Sequence Coverage ... 289
9.4.3.2 Coverage Control Panel ... 290
9.4.3.3 Proteins View - Protein Tools ... 292
9.4.3.4 Proteins View - Supporting Peptides ... 297
9.5 TMT/iTRAQ result - Peptides View ... 298
9.5.1 Peptides View - Peptide Table ... 298
9.5.2 Peptide Table controls ... 299
9.5.2.1 Peptide Table controls - Pagination ... 300
9.5.2.2 Peptide Table controls - View Filter ... 300
9.5.2.3 Peptide Table controls - Optional Columns ... 301

9.5.2.4 Peptide Table controls - Search function 301
9.5.3 Feature selection breadcrumb 302
9.5.4 Protein jump button 302
9.5.5 Annotated Spectrum Chart 302
9.5.5.1 Annotated Spectrum Chart - Chart Navigation 303
9.5.5.2 Annotated Spectrum Chart - Spectrum Annotation Settings 303
9.5.5.3 Annotated Spectrum Chart - Additional Chart Controls 304
9.5.6 Ion Match Table 305
9.5.7 Survey Chart 305
9.5.8 Reporter Ion 306
9.5.9 LC/MS Snapshot 306
9.6 TMT/iTRAQ Exporting 307
9.6.1 TMT/iTRAQ result - Exporting - comma separated values (CSV) format 308
9.6.2 TMT/iTRAQ result - Exporting - HTML format 308
10. Stable Isotope Labeling by Amino acids in Cell culture (SILAC) 309
10.1 Precursor Ion Quantification Analysis Workflow and Settings 309
10.2 Understanding SILAC Results 318
10.3. SILAC - Summary View 319
10.3.1 Summary View - Protein Filters 319
10.3.2 Summary View - Notes 319
10.3.3 Summary View - Heat Map 321
10.3.4 Summary View - Volcano plot 322
10.3.5 Summary View - Ratio Distribution Histogram 323
10.3.6 Summary View - Filtration parameters and statistics 324
10.3.7 Summary View - Search parameters 324
10.4 SILAC - Protein View 325
10.4.1 Proteins View - Protein Table controls 325
10.4.1.1 Protein Table controls - Protein View Filters 326
10.4.1.2 Protein Table controls - Protein count 327
10.4.1.3 Protein Table controls - Protein Table Search Function 327
10.4.1.4 Protein Table controls - Protein Table Export 327
10.4.2 Proteins View - Protein Table 327
10.4.3 Proteins View - Coverage Pane 328
10.4.3.1 Protein Sequence Coverage 329
10.4.3.2 Coverage Control Panel 330
10.4.3.3 Proteins View - Protein Tools 332
10.4.3.4 Proteins View - Supporting Peptides 337
10.5 SILAC result - Peptides View 338
10.5.1 Peptides View - Peptide Table 339
10.5.2 Peptide Table controls 340
10.5.2.1 Peptide Table controls - Pagination 340
10.5.2.2 Peptide Table controls - View Filter 340
10.5.2.3 Peptide Table controls - Search function 342
10.5.3 Feature vector selection breadcrumb 342

10.5.4 Protein jump button ... 343
10.5.5 Annotated Spectrum Chart ... 343
10.5.5.1 Annotated Spectrum Chart - Chart Navigation ... 343
10.5.5.2 Annotated Spectrum Chart - Spectrum Annotation Settings ... 343
10.5.5.3 Annotated Spectrum Chart - Additional Chart Controls ... 344
10.5.6 MS1 Spectrum ... 345
10.5.7 eXtracted Ion Chromatogram (XIC) chart ... 345
10.5.8 LC/MS Snapshot ... 346
10.6 SILAC Exporting ... 347
10.6.1 SILAC - Exporting - comma separated values (CSV) format ... 347
10.6.2 SILAC - Exporting - HTML format ... 348
11. Glycan Search ... 349
11.1 Glycan Search Analysis Workflow and Setting ... 349
11.2 Understanding Glycan Search results ... 354
11.3 Glycan Search Summary View ... 354
11.3.1 Summary View - Notes ... 355
11.3.2 Summary View - Protein Filters ... 356
11.3.3 Summary View - Statistics of Data ... 356
11.3.4 Summary View - Non-glycopeptide Precursor mass error distribution ... 356
11.3.5 Summary View - Glycopeptide Precursor mass error distribution ... 357
11.3.6 Summary View - Number of Glycosylation Sites (top 20) ... 358
11.3.7 Summary View - Result Filtration Parameters. ... 358
11.4 Glycan Proteins View ... 359
11.4.1 Protein Table ... 359
11.4.2 Protein View Filters ... 360
11.4.3 Protein Optional Columns ... 362
11.4.4 Protein Table Search Function ... 362
11.4.5 Protein Coverage ... 363
11.4.5.1 Protein Sequence Coverage ... 363
11.4.5.2 Glycan Profile ... 364
11.4.5.3 Glycan Pie Chart ... 365
11.4.5.4 Coverage Control Panel ... 366
11.4.6 Supporting Glycopeptides Table ... 368
11.4.7 Supporting Native Peptides Table ... 368
11.5 Glycan Search Glycopeptide View ... 369
11.5.1 Glycan Table ... 370
11.5.2 Glycan Table controls ... 372
11.5.2.1 Glycan Table controls - Pagination ... 372
11.5.2.2 Glycan Table controls - View Filter ... 372
11.5.2.3 Glycan Table controls - Optional Columns ... 374
11.5.2.4 Glycan Table controls - Search function ... 374
11.5.3 Feature selection breadcrumb ... 375
11.5.4 PSM selection ... 376
11.5.4.1 Protein jump button ... 376

11.5.4.2 Show spectrum in Data View 376
11.5.4.3 Show spectrum in LC/MS View 376
11.5.4.4 Show Raw Spectrum View 376
11.5.5 Annotated Spectrum Chart 377
11.5.5.1 Annotated Spectrum Chart - Chart Navigation 377
11.5.5.2 Annotated Spectrum Chart - Spectrum Annotation Settings 377
11.5.5.3 Annotated Spectrum Chart - Additional Chart Controls 378
11.5.6 Glycan Ion Match Table 379
11.5.7 Ion Match Table 379
11.5.8 Survey Chart 380
11.5.9 LC/MS Snapshot 380
11.5.10 Precursor Profile 381
11.6 Glycan Search Peptide View 382
11.6.1 Peptide Table 382
11.6.2 Peptide Table controls 384
11.6.2.1 Peptide Table controls - Pagination 384
11.6.2.2 Glycan Table controls - View Filter 384
11.6.2.3 Peptide Table controls - Optional Columns 385
11.6.2.4 Glycan Table controls - Search function 386
11.6.3 Feature selection breadcrumb 386
11.6.4 PSM selection 387
11.6.4.1 Protein jump button 387
11.6.4.2 Show spectrum in Data View 388
11.6.4.3 Show spectrum in LC/MS View 388
11.6.4.4 Show Raw Spectrum View 388
11.6.5 Annotated Spectrum Chart 388
11.6.5.1 Annotated Spectrum Chart - Chart Navigation 388
11.6.5.2 Annotated Spectrum Chart - Spectrum Annotation Settings 389
11.6.5.3 Annotated Spectrum Chart - Additional Chart Controls 390
11.6.6 Ion Match Table 390
11.6.7 Survey Chart 390
11.6.8 LC/MS Snapshot 391
11.6.9 Precursor Profile 392
11.7 Glycan Search Feature View 392
11.7.1 Feature Table 393
11.7.2 Feature Table controls 393
11.7.2.1 Feature Table controls - Pagination 393
11.7.2.2 Feature Table controls - Sample selection 394
11.7.2.3 Feature Table controls - Search function 394
11.7.3 Feature Details 394
11.7.4 PSM selection 394
11.7.4.1 Show spectrum in Data View 395
11.7.4.2 Show spectrum in LC/MS View 395
11.7.4.3 Show Raw Spectrum View 395

11.7.5 Annotated Spectrum Chart 395
11.7.5.1 Annotated Spectrum Chart - Chart Navigation 395
11.7.5.2 Annotated Spectrum Chart - Spectrum Annotation Settings 396
11.7.5.3 Annotated Spectrum Chart - Additional Chart Controls 397
11.7.6 Ion Match Table 397
11.7.7 Survey Chart 398
11.7.8 LC/MS Snapshot 398
11.7.9 Precursor Profile 399
11.8 Glycan Search Partial View 400
11.8.1 Partial Table 400
11.8.2 Partial Table controls 401
11.8.2.1 Partial Table controls - Pagination 401
11.8.2.2 Partial Table controls - Search function 401
11.8.3 Partial Spectrum Chart 402
11.8.3.1 Partial Spectrum Chart - Chart Navigation 402
11.8.3.2 Partial Spectrum Chart - Spectrum Annotation Settings 402
11.8.3.3 Partial Spectrum Chart - Additional Chart Controls 403
11.8.4 Partial Glycan Ion Match Table 404
11.8.5 Partial Ion Match Table 404
11.8.6 Partial Survey Chart 404
11.9 Glycan Search Exporting 405
11.9.1 Export - Glycan Search Protein CSV 406
11.9.2 Export - Glycan Search Support Peptide CSV 406
11.9.3 Export - Glycan Search Support Glycan Peptide CSV 406
11.9.4 Export - Glycan Search Peptide CSV 407
11.9.5 Export - Glycan Search Glycan Peptide CSV 407
11.9.6 Export - Glycan Search PSM CSV 407
11.9.7 Export - Glycan Search Glycan PSM CSV 408
11.9.8 Export - Glycan Search Feature CSV 409
11.9.9 Export - Glycan Search Glycosylation Sites CSV 410
11.9.10 Export - Glycan Search Glycan Partial Spec CSV 410
11.9.11 Export - Glycan Search All Spec CSV 411
11.9.12 Export - Glycan Search HTML 411
12. Tools 412
12.1 Mass Calculator 412
12.2 Spectral Library Viewer 412
12.2.1 Library Viewer - Meta Information 414
12.2.2 Library Viewer - Statistical Distribution 415
12.2.3 Library Viewer - Library Table 417
12.2.3.1 Library Table - Pagination 419
12.2.3.2 Library Table - Search function 419
12.2.3.3 Library Table - Spectrum Chart 420
12.2.3.4 Library Table - Peptide Details 422
12.2.3.5 Library Table - Ion Table 422

12.3 Compare Result 423
12.3.1 Compare Result - Protein Compare Table 423
12.3.1.1 Protein Compare Table - Pagination 424
12.3.1.2 Protein Compare Table - Protein Result Filter 425
12.3.1.3 Protein Compare Table - Search Function 425
12.3.1.4 Protein Compare Table - Exporting 426
12.3.1.5. Protein Compare Table - Venn Diagram 426
12.3.1.6. Protein Compare Table - Score Distribution 426
12.3.2 Compare Result - Peptide Compare Table 427
12.3.2.1 Peptide Compare Table - Pagination 428
12.3.2.2 Peptide Compare Table - Peptide Result Filter 428
12.3.2.3 Peptide Compare Table - Search Function 429
12.3.2.4 Peptide Compare Table - Exporting 429
12.3.2.5. Peptide Compare Table - Venn Diagram 429
12.3.2.6. Peptide Compare Table - Score Distribution 430
12.3.3 Compare Result - Glycan Compare Table 430
12.3.3.1 Glycan Compare Table - Pagination 431
12.3.3.2 Glycan Compare Table - Peptide Result Filter 432
12.3.3.3 Glycan Compare Table - Search Function 433
12.3.3.4 Glycan Compare Table - Exporting 433
12.3.3.5. Glycan Compare Table - Venn Diagram 433
12.3.3.6. Glycan Compare Table - Glycan Score Distribution 433
12.3.3.7. Glycan Compare Table - Peptide Score Distribution 434
12.4 Performance Configuration 434
12.5 Worker Monitor 435

1. Overview

Welcome to PEAKS Studio 11.

1.1 How to Use This Manual

This chapter provides an overview of PEAKS Studio 11's features typical data analysis work flow in PEAKS Studio. It is strongly recommended for new users of PEAKS Studio to read this chapter to get a big picture of what PEAKS Studio provides and how to use PEAKS Studio. For users who are familiar with the PEAKS Studio product line from previous releases, it will be a similar user experience and this chapter may be used as reference.

1.2 What is PEAKS Studio 11?

PEAKS Studio 11 is the first release of PEAKS Studio on the new platform. PEAKS Studio 11 is an all-in-one software solution for data dependent acquisition (DDA) and data independent acquisition (DIA) shotgun proteomic mass spectrometry data analysis. It is a vendor-neutral platform capable of reading in raw mass spectrometry data and public data formats. Intuitive result visualization and validation tools are provided at every stage of analysis and results can be exported. In addition to the various DDA and DIA identification and quantification workflows with ion mobility support provided in previous releases, PEAKS Studio 11 highlights the new **Glycan Search** and **Deep learning** algorithm.

1.3 Installation and Activation

1.3.1 System Requirements

PEAKS 11 is recommended to be installed on 64-bit Windows operating system with Windows 10 or later. PEAKS 11 installer will take ~2.5GB of disk space and PEAKS 11 program files will use ~5GB disk space. The program files folder will take up more disk space over time as protein databases and glycan databases are configured and saved. The amount of disk space required for this purpose depends on the size of the user's datasets. Protein databases may require up to double the file size of the configured .fasta file. It is recommended to allocate a large disk space for the purpose of running projects, and ensure the location of the project has a lot of free space to handle the size of the project.

The two main factors affecting PEAKS 11's performance are CPU and RAM. The minimum, recommended and ideal requirements are as follows:

- Minimum: Quad-core processors and 32 GB of RAM.
- Recommended: 16 threads processors and 64GB of RAM with compatible GPU (described below).
- Ideal: Intel Core i7/i9/Xeon or AMD Ryzen 7/9/threadripper processors with total 32 threads or more, and 64-128 GB of RAM with compatible GPU.

For running DIA Database Search, it is recommended that the machine is equipped with at least 64GB of free memory and a NVIDIA CUDA compute capability >= 4 GPU with at least 8GB of dedicated memory.

1.3.2 Installation on a Windows operating system

Double-click on the PEAKS Studio 11.exe installer.

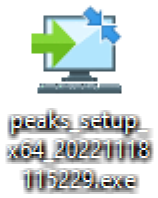

Go through the steps of the Setup Wizard, selecting a directory for the application to be installed in. By default, it will select path **C:\PEAKSStudio11**

1.3.3 Activation

Once set up, double-click on **PEAKSStudio.exe** in the installation folder.

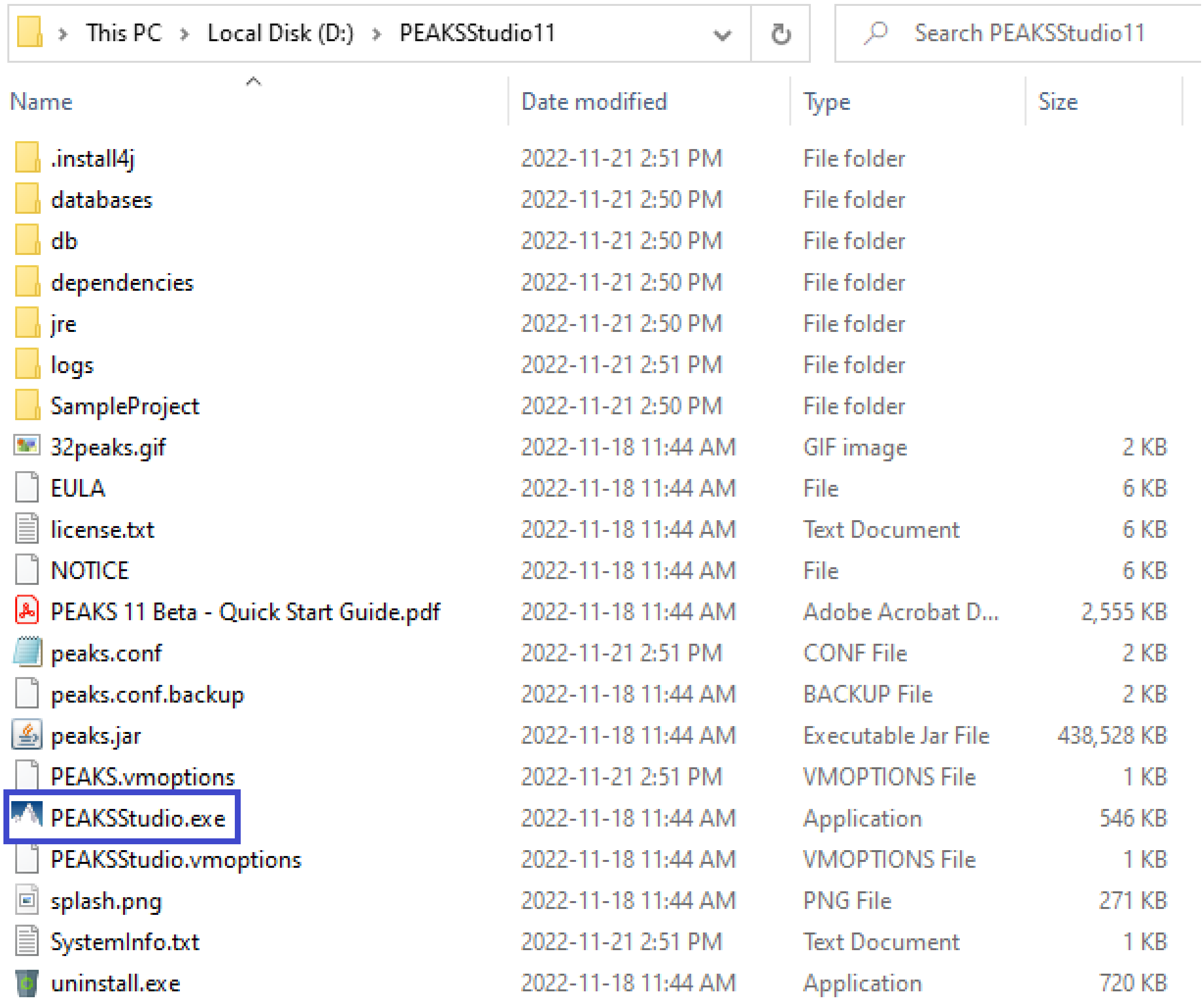

You will be prompted for a license key. For purchasing a license or requesting a trial license key, please contact sales@bioinfor.com

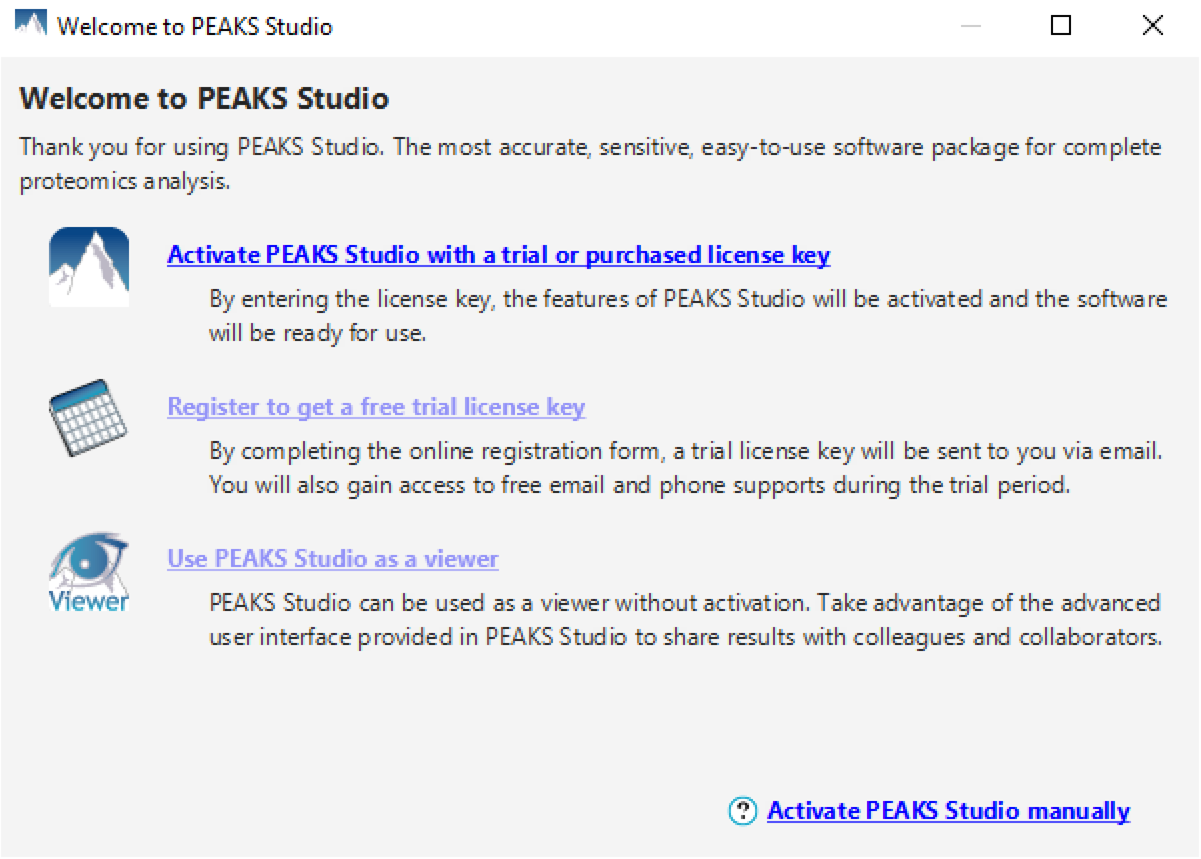

After a successful activation, the application will perform an automatic performance configuration. Double-click on PEAKS.exe again to open the program and you will be greeted with the main interface.

1.3.4 License Key descriptions

To view the current license, click on the icon under the **Help** menu located at the top of the interface. This opens the **About BSI PEAKS Studio** dialog where the current license details are listed.

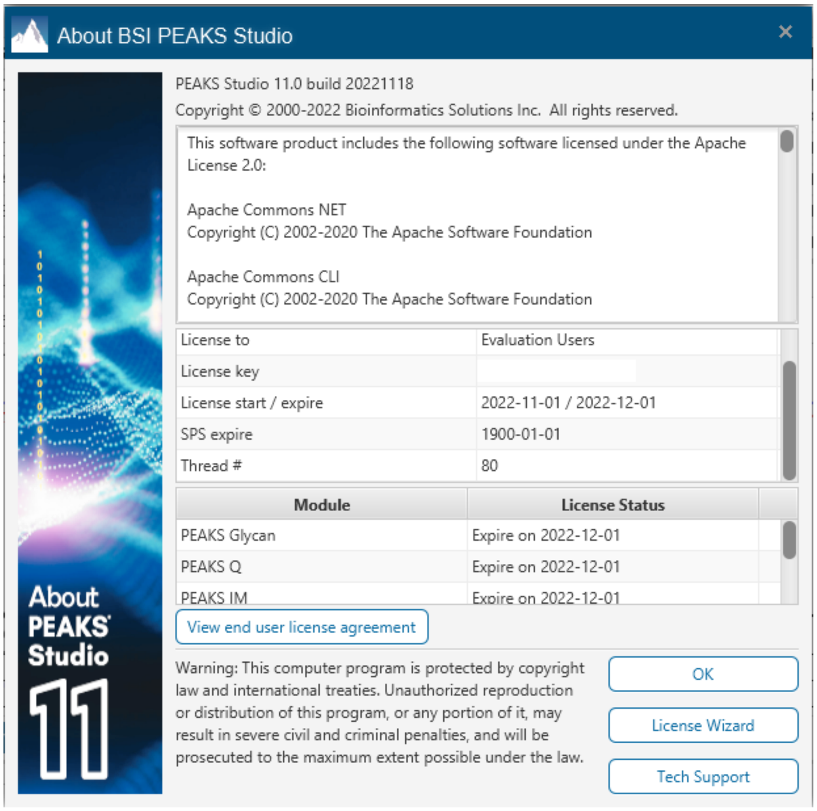

Each license key has a specified start / expire date. Licenses will have activation limits so it is intended to only be applied on the machines designated for PEAKS Studio 11. In the Module section, the following optional modules that are included in the license are listed.

PEAKS Platform: This is the base license without any add-on modules. Both DDA and DIA workflows are available.

PEAKS IM module: Enables running DDA and DIA analysis with ion mobility data, including timsTOF 1/k0 and FAIMS CV with results displayed in a 4D view.

PEAKS Q module: Enables running quantification workflows including Precursor ion quantification (SILAC/iTRAQ), Reporter ion quantification (TMT/iTRAQ), Label-free quantification for DDA and Label-free quantification for DIA.

PEAKS Glycan module: Enables running Glycan Search workflows.

1.4 Supported Data Formats

PEAKS 11 currently supports vendors with the following data formats:

1) Thermo - .raw, FAIMS, DIA
2) Bruker - timsTOF, tdf/baf/yep/fid, diaPASEF
3) Waters - MSe (data loading only), .raw
4) SCIEX - .wiff, .wiff2, DIA, ZenoTOF
5) Agilent - .d
6) Shimadzu - .lcd
7) .mzml and .mzXML

1.5 Quick Walkthrough

This chapter will go over the basic functions in creating projects and running analyses, as well as provide an overview of the different options available for deleting samples and analysis, modifying and running analysis, and other navigation options.

1.5.1 Start Page and Sample Project

Upon opening PEAKS 11 interface for the first time, you are presented with the Start Page seen below.

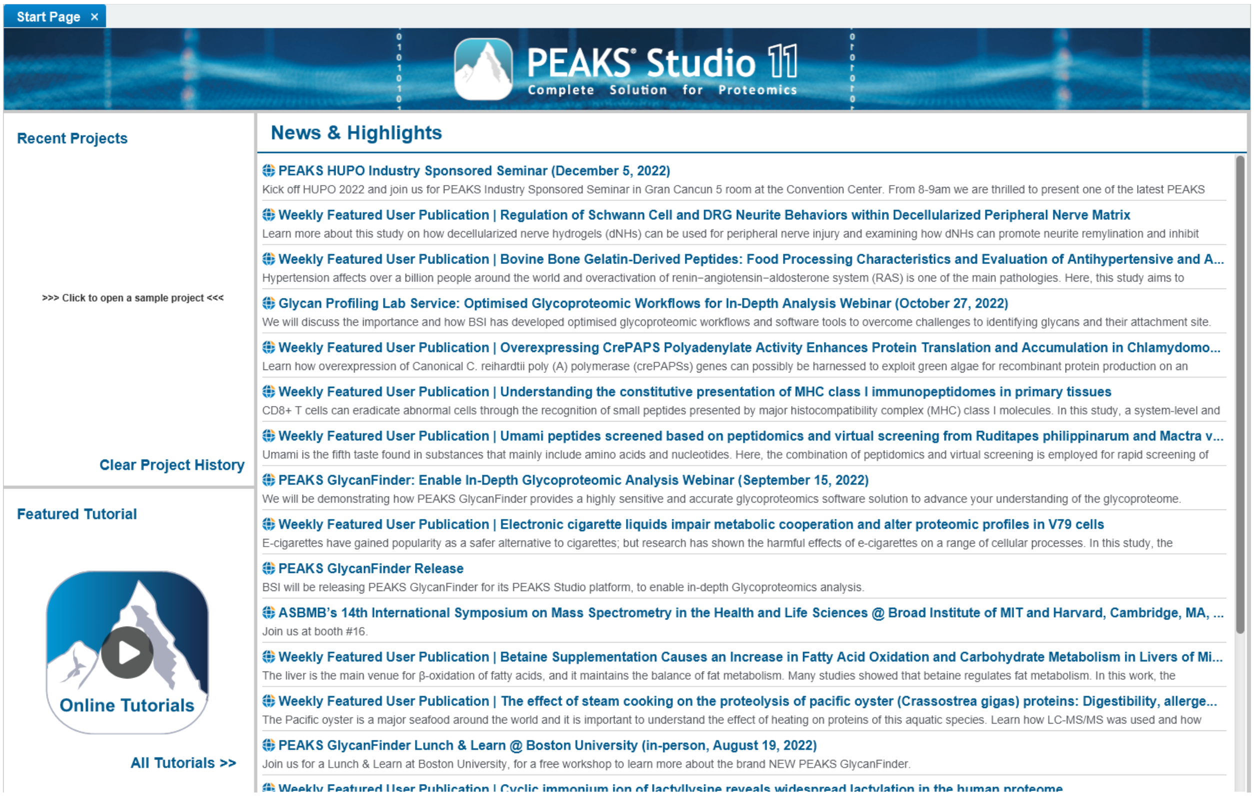

The Start Page lists recent news and highlights related to PEAKS Software line. Clicking on any of the titles will open the web browser showing interesting reads about recent developments.

In the top left "Recent Projects" section, there is an option to open the **Sample Project**. If you do not see the option, click on "Clear Project History". The **Sample Project** is meant for users to have a quick view of what PEAKS 11 has to offer. This project cannot be modified or deleted in any way.

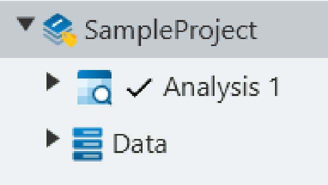

Once open, click on the arrow next to **Analysis 1** and double-click on the **DB Search** result node to open the sample result. This is the **Database Search** result which you can learn more about in later sections.

1.5.2 Setting up a new project

This section is created for those not familiar with previous PEAKS Studio or PEAKS Online platforms. If you have experience with PEAKS products, the workflow settings and user interface (UI) for PEAKS Studio 11 will be straightforward.

To get started with a new project, in the toolbar, click on [icon] to create a new project.

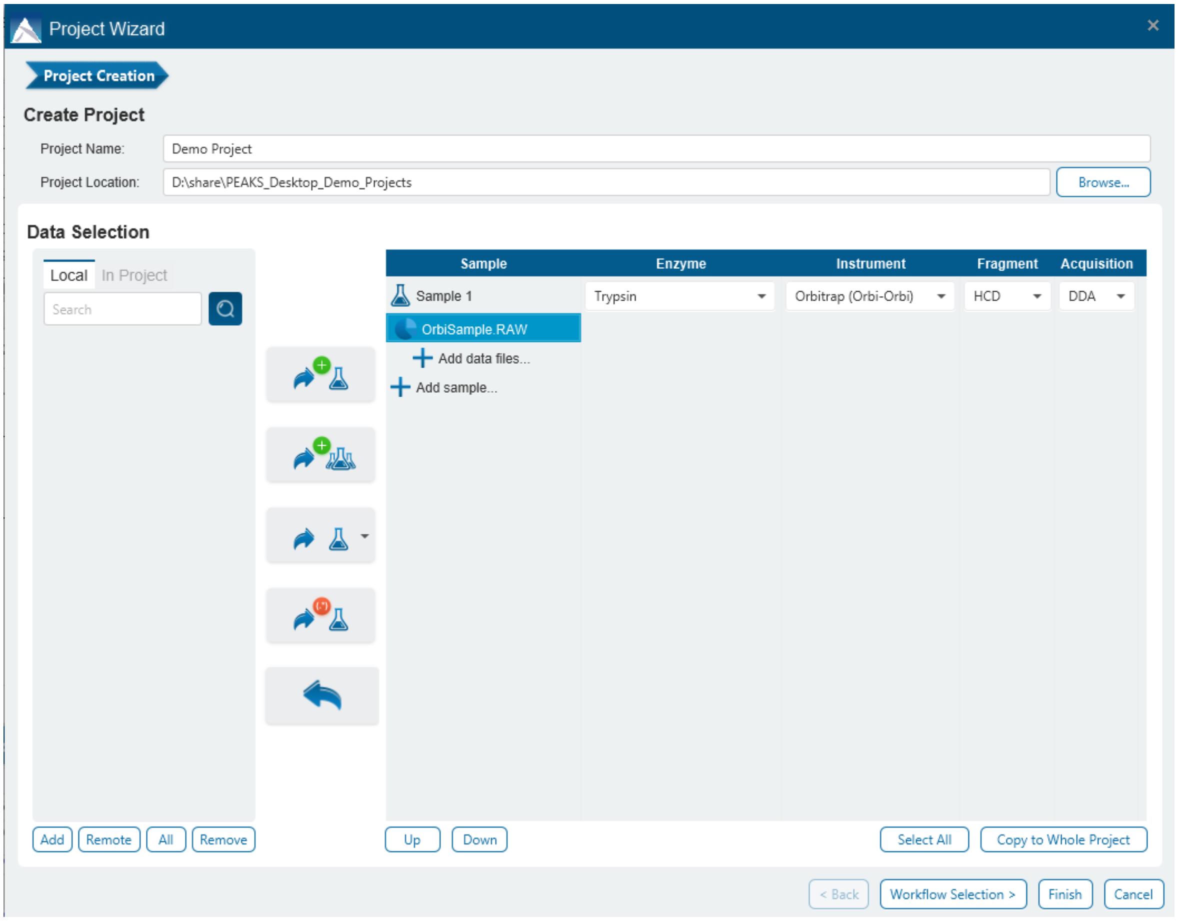

The Project Wizard opens at the Project Creation step. Here, you first add the raw mass spectrometric data files into the project. Add in a demo file from orbitrap instrument if you have one available. The data files selected need to be added into samples, and we provide an option to quickly configure a large number of samples by name using the 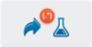 button. Adding samples by **Delimiter** is a great feature for quickly grouping and naming samples for large experiments.

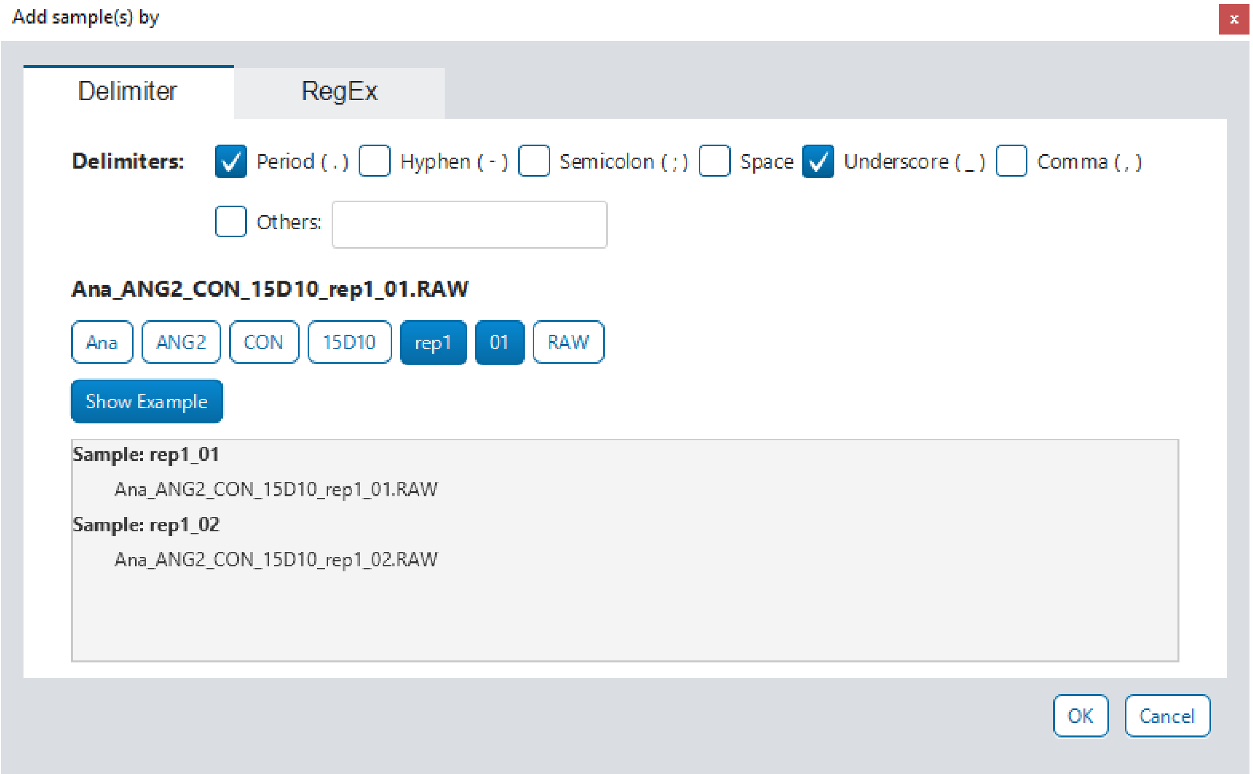

After selecting supported data files and adding them into samples, make sure to set the correct Instrument, Fragmentation, and Acquisition mode. Use the **“Copy to Whole Project”** button to copy the settings from the selected sample to all other samples.

Next, you may click “Finish” to load the data files only or you may proceed with setting up of your analysis by clicking on “Workflow Selection >”.

For raw data acquired in Data Dependent Acquisition (DDA) mode, five Workflow options are available:

- De Novo
- PEAKS DB Search (optional PEAKS PTM Finder and SPIDER)
- PEAKS Q (LFQ, SILAC, TMT/iTRAQ)
- Glycan Search
- DeepNovo Peptidome

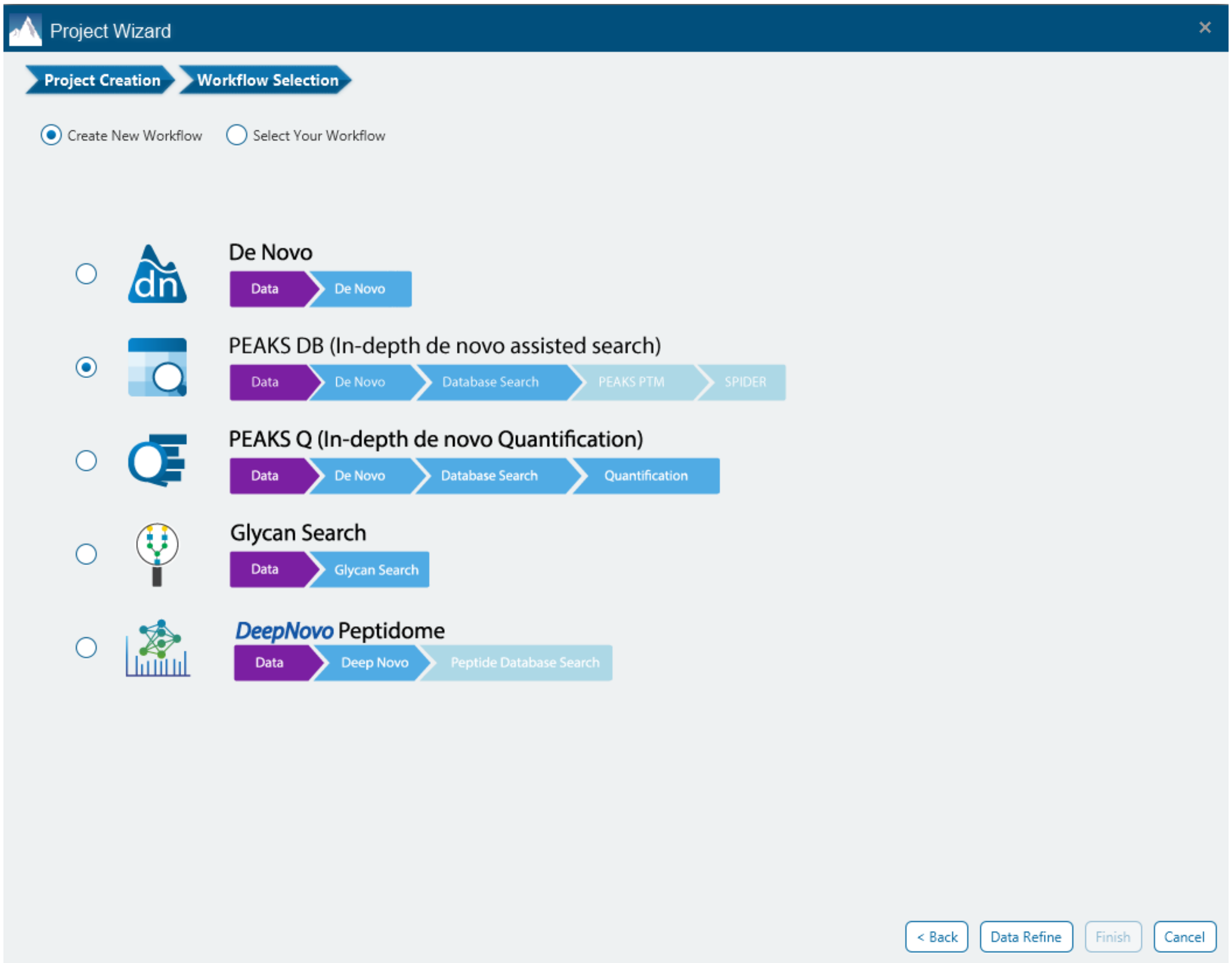

For raw data acquired in Data Independent Acquisition (DIA) mode, two Workflow options are available:

- DIA Identification (Spectral Library Search, Database Search, De Novo)
- DIA Quantification (Spectral Library Search, Database Search, LFQ)

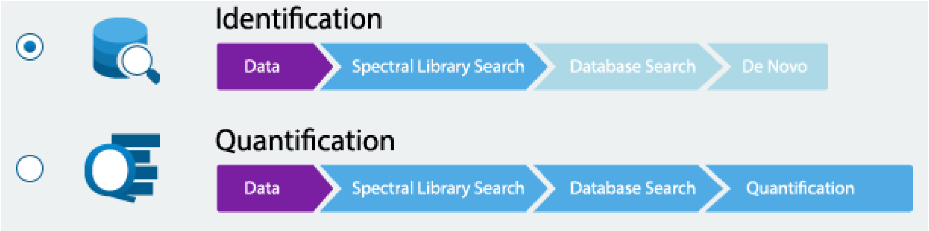

Finally, you may create a new workflow or use a saved workflow from previous analysis, where saved parameters are automatically loaded into your new analysis. Click on the “Select Your Workflow” option to see a list of saved workflows. Any workflows you save later will appear here.

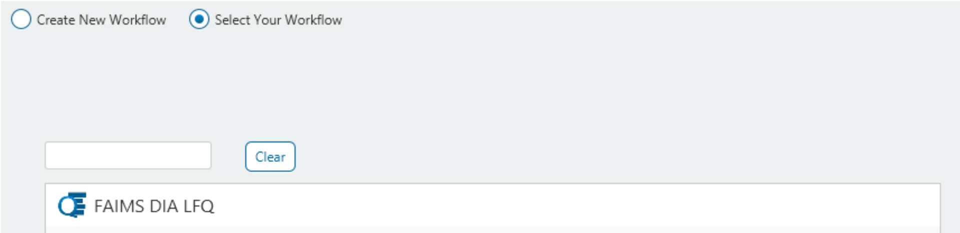

For the purpose of this demo, select the DDA PEAKS DB Workflow indicated below.

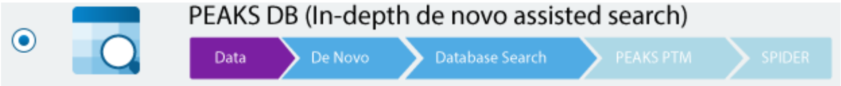

Click on “Data Refine” to begin setting up the PEAKS DB workflow.

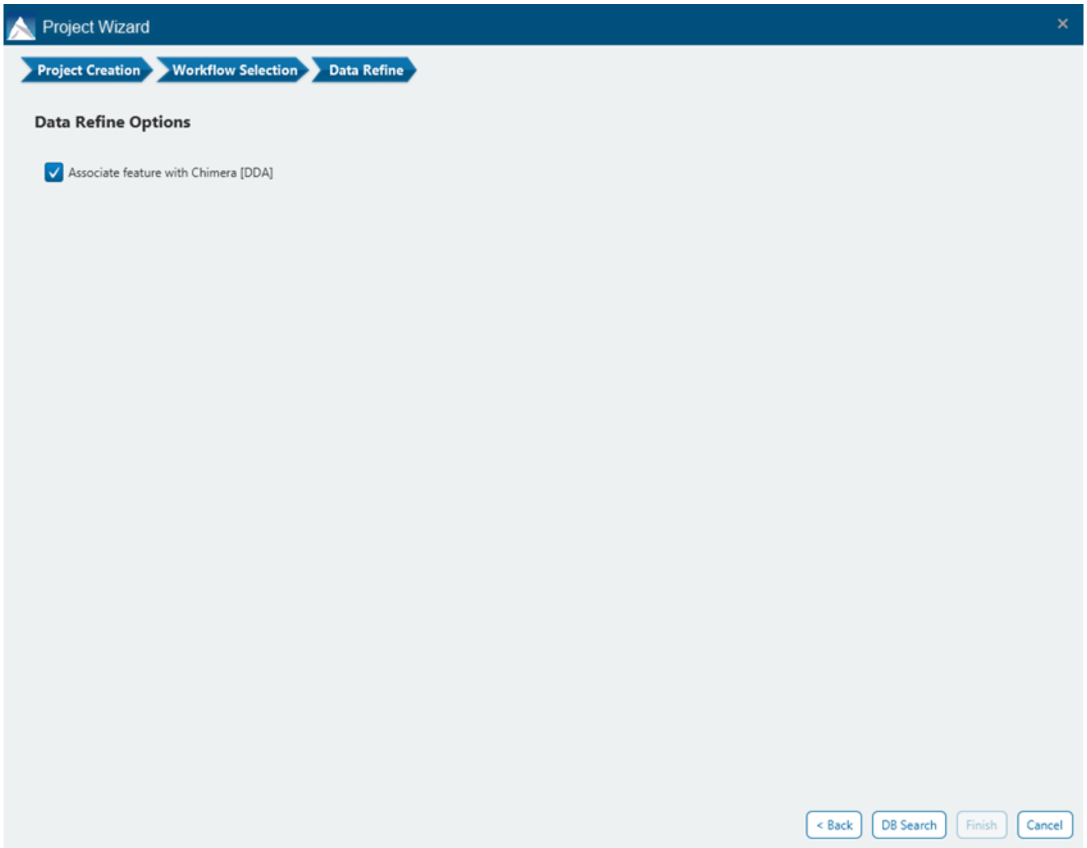

The first step in PEAKS DB workflow is Data Refine. This step prompts to enable/disable “Associate feature with Chimera”. If enabled, PEAKS will assume that a tandem scan may contain two or more peptides, which will allow PEAKS to identify co-eluting peptides found within the acquisition window of the scan. It does this by searching for peptides that match the monoisotopic mass of any peptide feature in the acquisition window.

Once selected, in the bottom right corner, click on “DB Search” to continue to the next step.

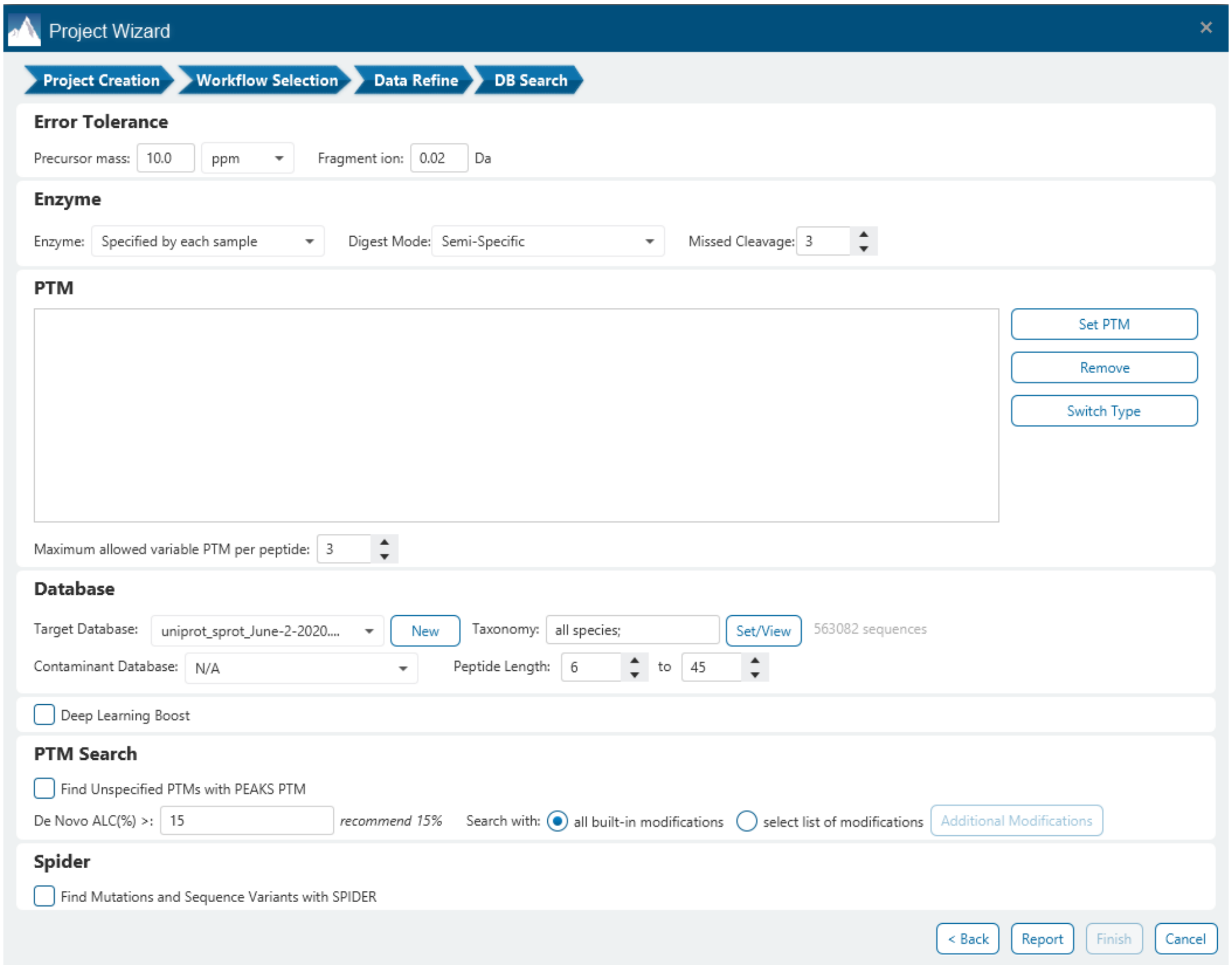

The DB Search setup contains parameters such as mass tolerance, enzyme and digest mode, missed cleavages, modifications (fixed or variable), database information, as well as advanced search options such as PTM Finder (search for additional modifications) and SPIDER (search for sequence variations).

Existing configured protein database may be selected, or a new database can be added and configured by clicking on "New" option. Note that for the Uniprot database, the taxonomy files (taxdmp and speclist) are already preloaded into the databases folder in the installation folder.

Once finished, click on Report to proceed to the next step.

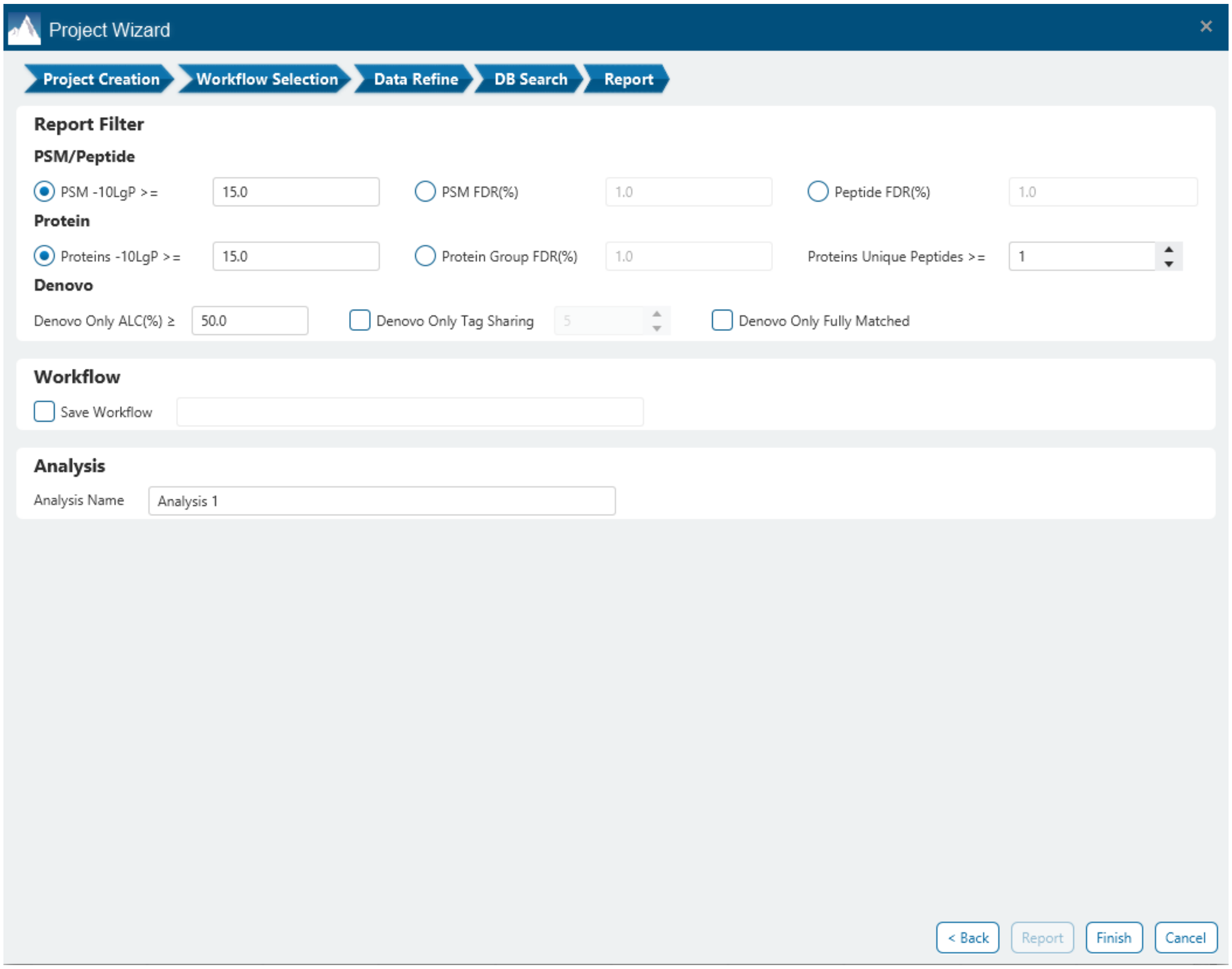

Report Filter is the final step in the workflow setup. Here, pre-selected scoring for PSM/peptide, protein, and De novo tag will determine the reported results. Important to note is that changing these filters and re-running the same analysis will take less time given that these filters are applied to the final output.

At this point, you may also choose to save your workflow parameters, which will allow you to re-select this workflow in the future. Lastly, the analysis can be given a name, or it can be renamed later in the project view.

Clicking on Finish will start loading the data files and running the analysis.

1.5.3 Monitoring analysis and task cancellation

Click on the **Progress** tab on the bottom left (below the project tree) to see progress status of projects or analyses being run. The statuses are as follows:

- RUNNING: The tasks in this step are running and in progress.
- PENDING: The tasks in this step are queued.
- DONE: All tasks in this step are complete.
- FAILED: An issue has prevented the task to complete.
- CANCELLED: The analysis was cancelled while in progress.

The progress bar is also colour-coded. Green indicates completed tasks in the step, blue indicates tasks in progress, orange indicates pending tasks in the step, red is an encountered failure during a task, and light blue is analysis cancellation.

In progress and pending tasks can be cancelled by right-clicking on the analysis.

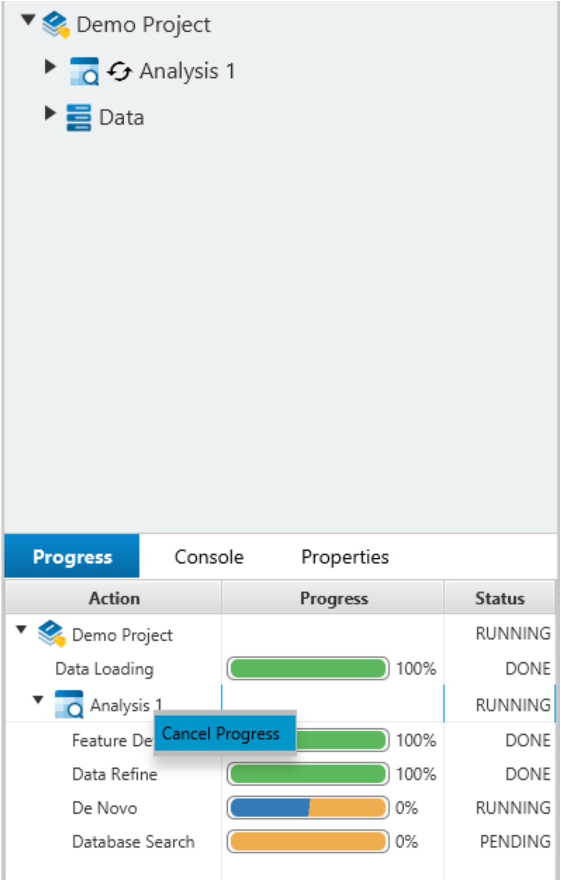

A bar at the bottom left will also indicate if PEAKS Studio is currently busy running tasks.

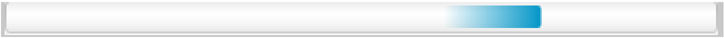

Clicking on the **Console** tab will provides more details on the current tasks being run and may give more insight on how far the analysis progressed.

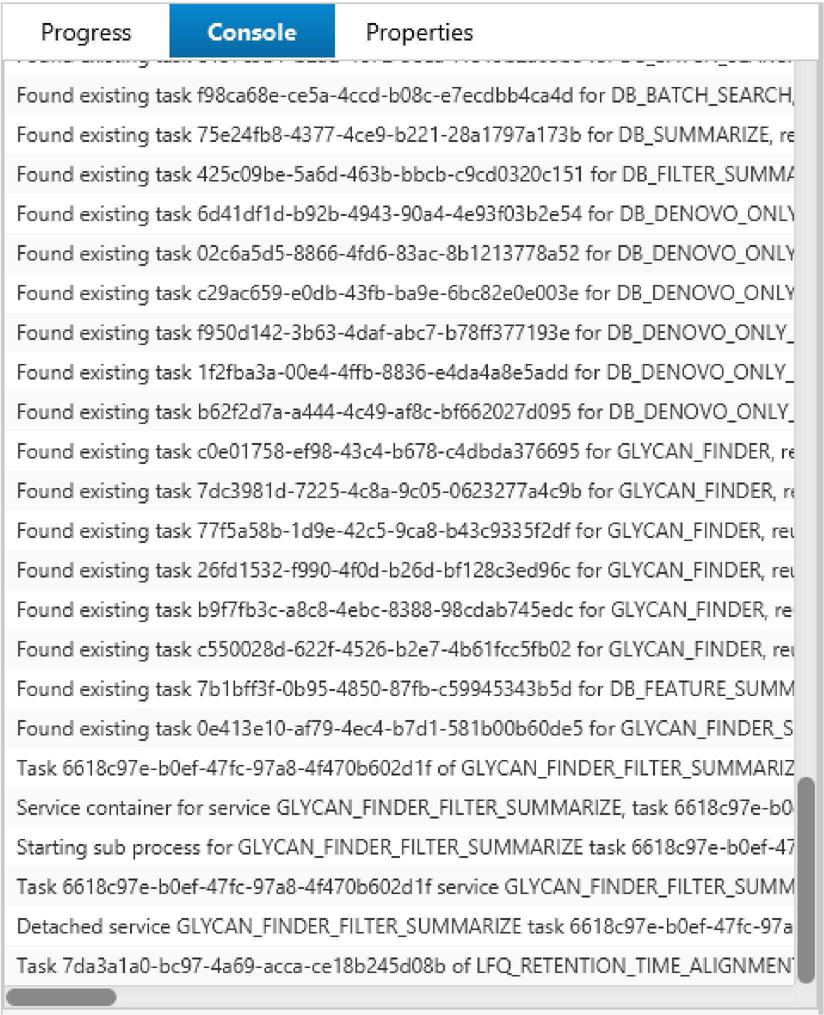

In the build directory there is a **logs** folder which also logs this running info. A tasks folder saves all the logs for each individual task ran.

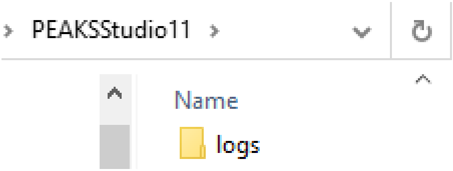

When encountering issues, the user is encouraged to submit the **logs** folder to the PEAKS support team so that we can better assist you with the problem.

Clicking on the Properties tab will show details on each of the selected result nodes. For example, selecting a Glycan Search result node will display the parameters used in this setup in the Properties tab.

In addition, selecting either the Project node or Analysis node in the project tree will display the time that the project or analysis was created.

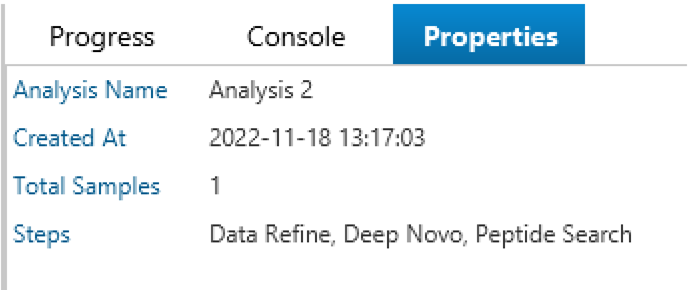

1.5.4 Project Tree Hierarchy

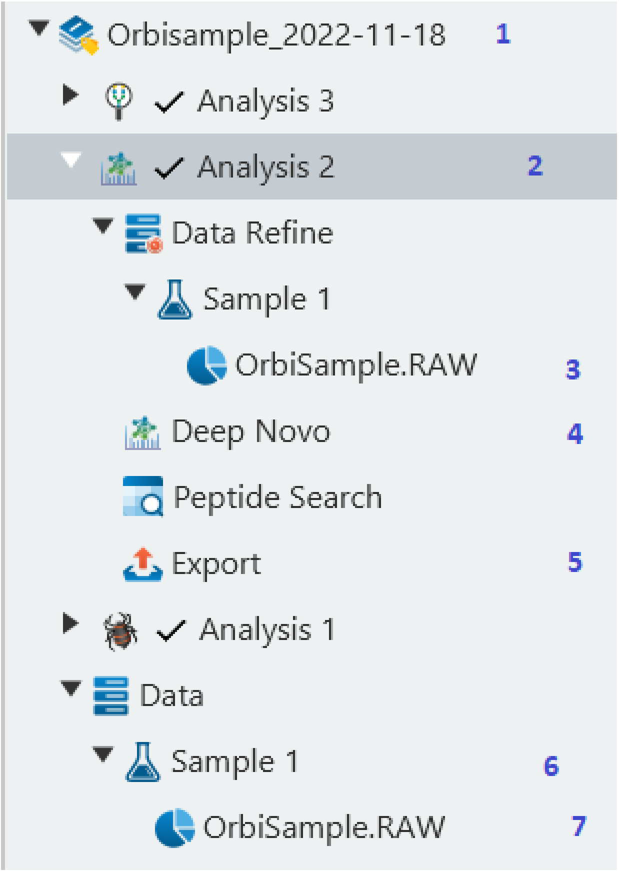

In the top left of the interface is the Project View. Project View will display any opened projects that are complete or in progress. The typical project tree has the following nodes:

1. Project node (top level): Displays the project name. It can collapse/expand the project. Right clicking will allow user to close the project or run another analysis in the project.
2. Analysis node (second level): Each analysis run will have its analysis node under the project. Each analysis can collapse/expand. Right clicking on the analysis node provides the option to change the name of the Analysis. There is also an option to delete the analysis from the project.
3. Data Refine node (third level): Expand to see the samples included in the analysis. Expand each sample to see the fractions or data files added to the sample. Double-click to open the data refine view. For more information on the Data Refine view, see 5. Data Refinement.

4. Result node (third level): The main result node. Double-click to open the result node. Later sections will cover details of the result view.
5. Export node (third level): The Export result node provides several options for exporting. This is particularly useful for exporting the results tables into a comma separated text file (csv). There are several other export options available such as mgf export, html export, and spectral library generation. Double-click to open the Export node and see the options available. For more details on each of the exports, refer to the **Export** sections in each of the result node chapters.
6. Sample node (second level): Displays the samples in the project. Click on the dropdown arrow to expand and reveal the data files in that sample. Right-click on the sample to delete it from the project. This can only be done if no analysis is using that sample and the project is not currently running an analysis.
7. Data File node (third level): Similar to the Data Refine Node but with unrefined (raw) data view. Double-click to open any of the raw data files.

1.5.5 Analysis and Sample deletion

Once the project analysis run is completed, the result nodes will appear in the project view (top left). Here, completed analyses can be renamed or deleted by right-clicking on them.

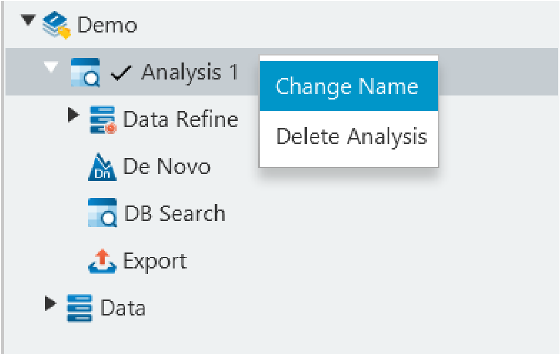

Samples can also be deleted by right-clicking on the Sample under 'Data' dropdown. Important to note that the Data can only be deleted if it is currently not a part of any analysis, and the project, that the sample is loaded into, is not running any analysis at that moment. Additionally, all analyses containing the sample to be deleted, must be deleted first. This is to prevent unwanted issues related to removal of the data from the project.

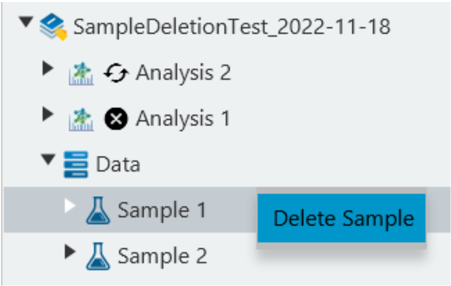

1.5.6 Running additional analyses in existing project and modifying analysis

In the Projects tab, click on 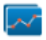 to run a new analysis in the current project. Within Data Selection, In Project tab will allow selecting pre-loaded samples.

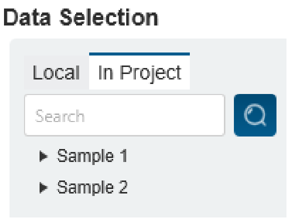

Another useful feature for running additional analyses is “Modify Analysis” function. Clicking on will prepare a new analysis with the same parameters as the existing one, which makes it a fast and convenient way to modify only specific parameters or report new filters without having to go through each of the settings again.

If the analysis is run with the same settings as the existing analysis in the project, the project should finish instantaneously, creating a duplicate of that analysis.

1.5.7 Project opening and closing

While the Projects tab is selected, the projects currently opened are at the top. Select the tab to switch between each project. Clicking ‘X’ on the tab will close the project.

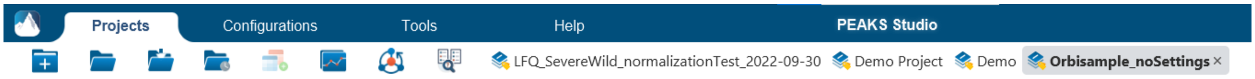

Projects can also be closed by right-clicking on the project and selecting **“Close Project”** in the dropdown. It is recommended to close unused projects to preserve memory.

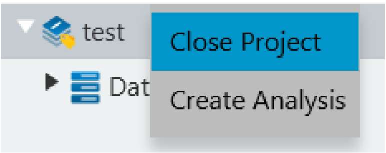

To open an existing project, click on the icon. You can also open from the list of recently opened projects by clicking on . Ten latest projects are listed here. Recent projects can also be opened from the **Start Page** Recent Projects lists.

1.6 Import PEAKS Online Project

PEAKS Studio 11 also supports importing archived PEAKS Online projects. Click on to select an archived Online project to view in PEAKS Studio 11.

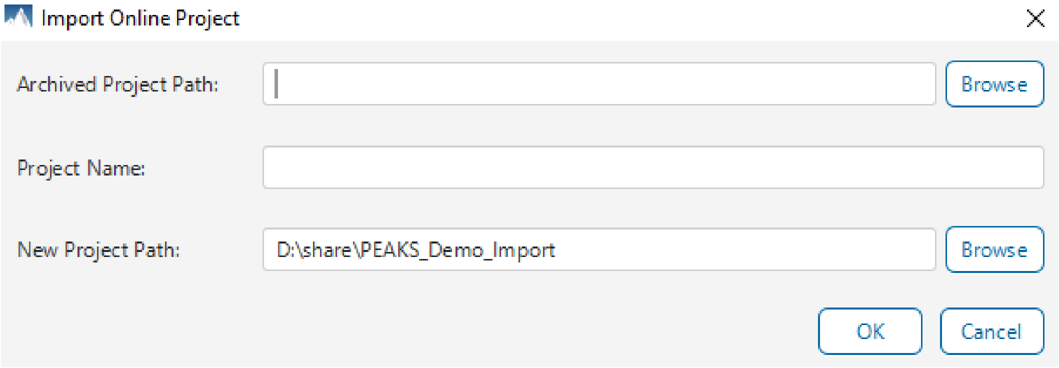

Any result nodes that are available in both PEAKS Online and PEAKS Studio can be imported successfully into PEAKS Studio. Certain figures and tables will not be displayed since they are not available in the PEAKS Online project. Due to different project structures, running an analysis on an imported PEAKS Online project is prevented.

2. Configuration

Click on the button to open the configuration dialog. We provide a number of configuration options which will be explored in the following sections.

2.1 General Configuration - Default Directories Preferences

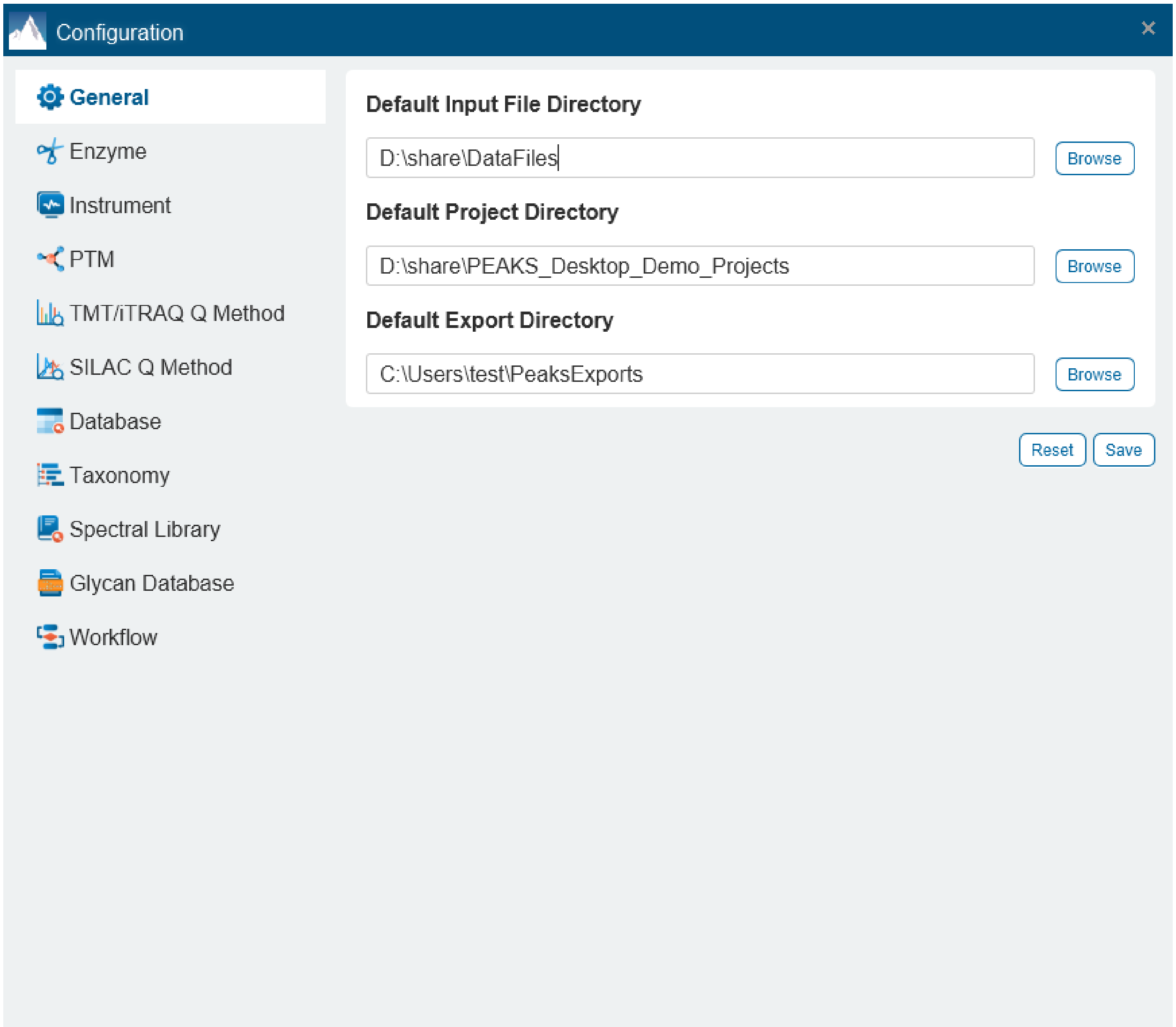

Default directories can be set in Configuration > General. Each of these directories are used as follows:

- Default Input File Directory: This is the folder that is selected initially when adding input data files. Choose the location of your source files for easy access when creating projects or analyses.
- Default Project Directory: This folder specifies the default location that projects will be created in. When opening the Project Wizard, this will be the path set in 'Project Location'.
- Default Export Directory: This is the folder that is selected by default for all exports of PEAKS Studio. This includes exports of .csv and .html files from the **Export** node as well as any image exports provided.

Clicking on ‘Save’ will save the selected directories.

Clicking on ‘Reset’ will set the directories to default. A pop-up will prompt the user to confirm this action.

2.2 Enzyme Configuration

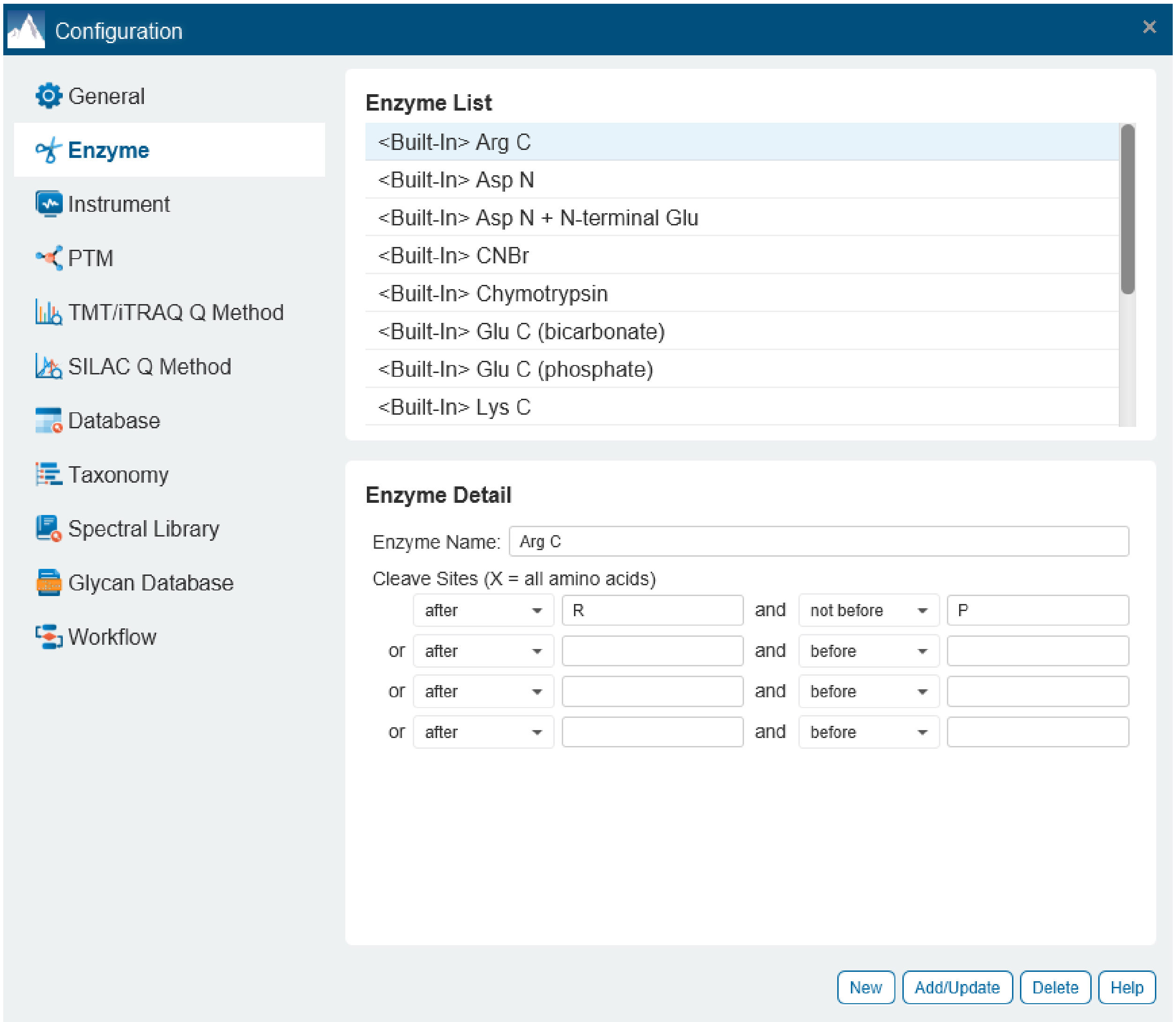

All of the built-in enzymes are listed here. A built-in enzyme cannot be modified or deleted.

Custom Enzymes can be created and updated. Provide the name of the new enzyme in the “Enzyme Name” field and specify how the custom enzyme will cleave the protein between two amino acids to create peptides in the “Enzyme Detail” panel.

The letter, X, denotes *any amino acid at this position*, while amino acids within the { and } brackets indicate any amino acid except for the one in the brackets. Choose where the cleavage sites are by selecting *after* or *not after* and *before* or *not before* to specify the range. Add multiple amino acids to indicate that cleavage happens before or after any of the stated amino acids. For example, after RK means after R or K not after R and K.

Click the Add/Update button to save the changes. The new enzyme will now appear in the “Enzyme List”, where it can be accessed later. To delete a customized enzyme, select the appropriate enzyme and click the Delete button.

2.3 Instrument Configuration

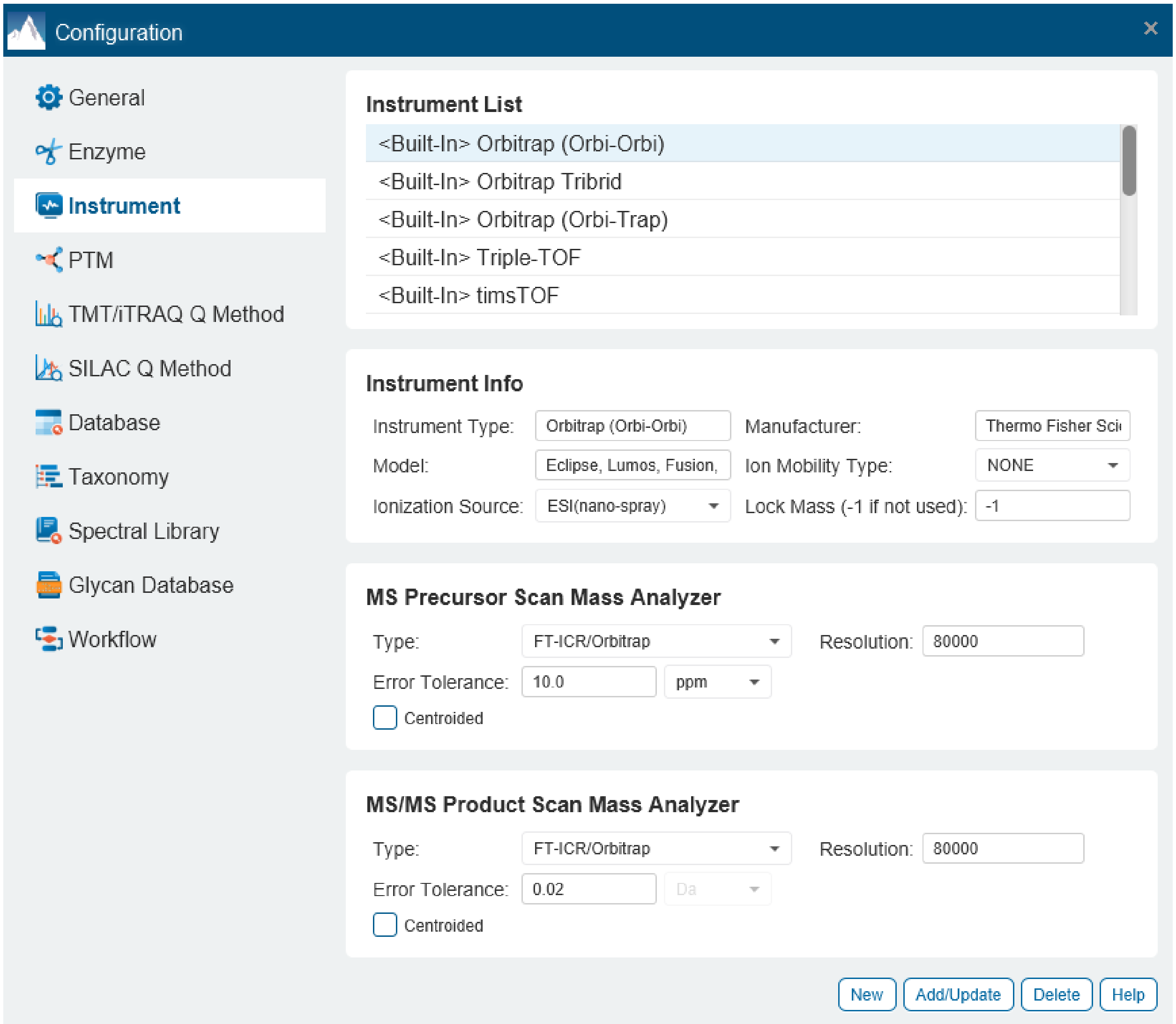

The names of the built-in instruments are provided in the “Instrument List”. Select an instrument to view the detailed instrument information in the “Instrument Detail” panel below.

The details of a built-in instrument cannot be deleted or edited.

Create a new instrument.
Click the New button and provide a name for the instrument in the “Instrument Details” panel.

Use the “Ion Source” drop-down menu to select the ion source that was used: MALDI/SELDI or ESI (nano-spray). This will help the PEAKS Data Refine tool to determine the charge of the ions.

Use the “MS Precursor Scan” drop-down menu to select the type of MS scan that was performed. This selection will tell the PEAKS Data Refine tool whether the survey scan is of sufficient resolution to determine the charge and the monoisotopic peak during examination of the survey scan.

Use the “MS/MS Product Scan” drop-down menu to select the type of MS/MS scan that was performed. This selection will help PEAKS decide which internal parameters (for weighing fragments and amount of noise) to use during PEAKS auto de novo sequencing and a PEAKS DB search. Select LIT/FT if alternating high-res/low-res modes were used. This will allow the algorithm to determine the mass analyzer from the scan header.

Specify the values for “Parent mass error tolerance” and “Fragment mass error tolerance” in Daltons or ppm.

Click the Add/Update button to save the changes. The new instrument will appear in the “Instrument List” where it can be accessed when creating a new project file. To delete a customized instrument, select the appropriate instrument from the “Instrument List” and click the Delete button.

2.4 PTM Configuration

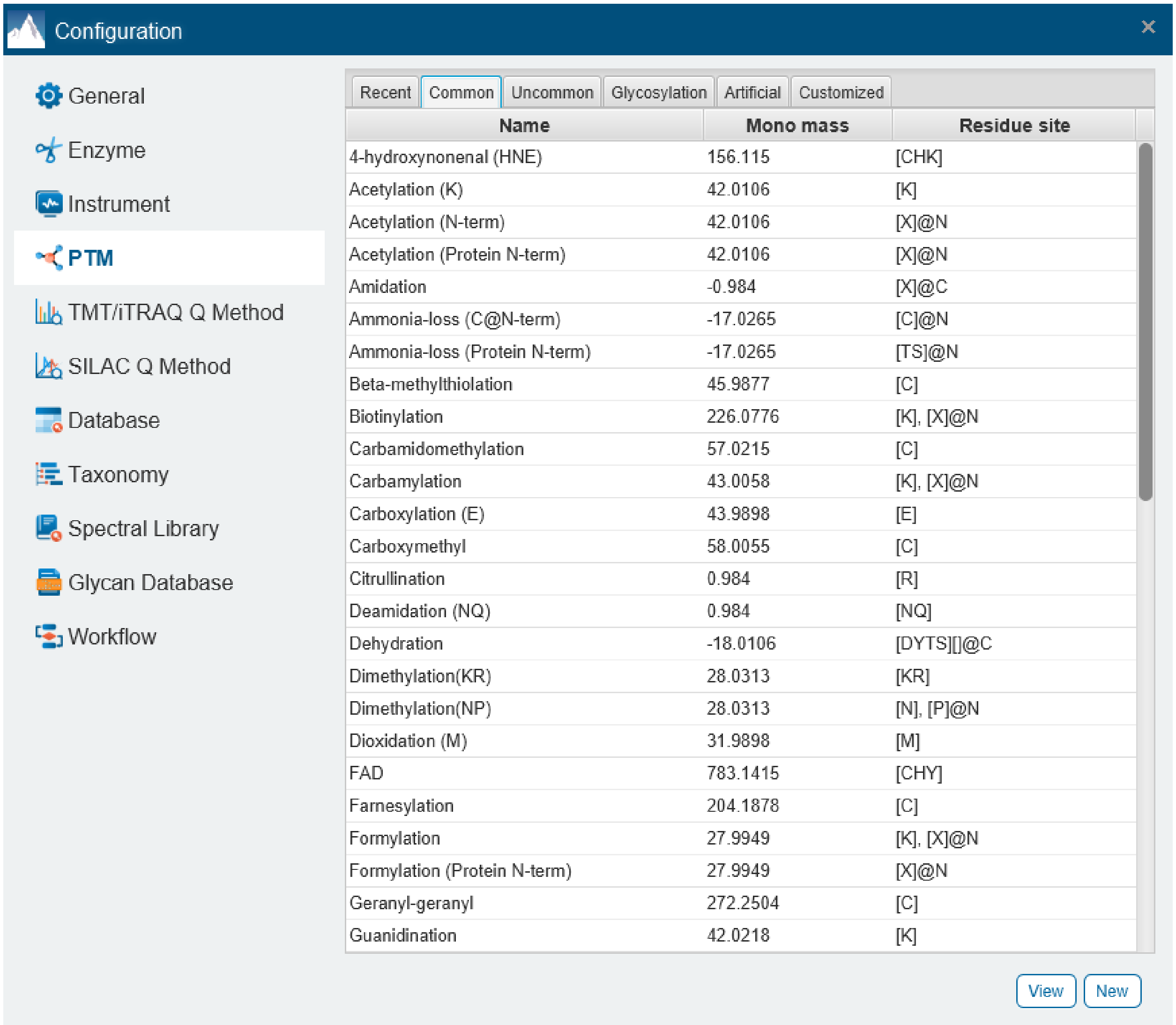

Create a new PTM. To create a new PTM, click on the New button in the bottom right-hand corner of the PTM Configuration window to display the "New PTM" dialogue. Provide the necessary information about the new PTM.

- PTM Name: This name will appear in the PTM list for future use after it is saved.
- PTM abbreviation: The PTM name expressed in a shortened form.
- Mass (Monoisotopic): The mass that the residue gains or loses as a result of the PTM.
- Residues that can be modified: Enter residues that can be modified anywhere, or residues that can only be modified if they are at the N- or C-terminus. User will also need to specify if these residues can be modified on the peptide-level or protein-level.
- Formula: The chemical formula of the PTM. This should correspond to the mass listed above.
- Rule: This field is used for the user's reference only. Users can use the field to enter a comment about the PTM.

Click the OK button to save the changes. The new PTM will now appear in the "Customized" PTM list, where it can be accessed later. To delete a customized PTM, select the appropriate PTM from the list and click the Delete button.

The "Recent" tab lists all the PTMs recently used in analyses.

2.5 TMT/iTRAQ Q Method Configuration

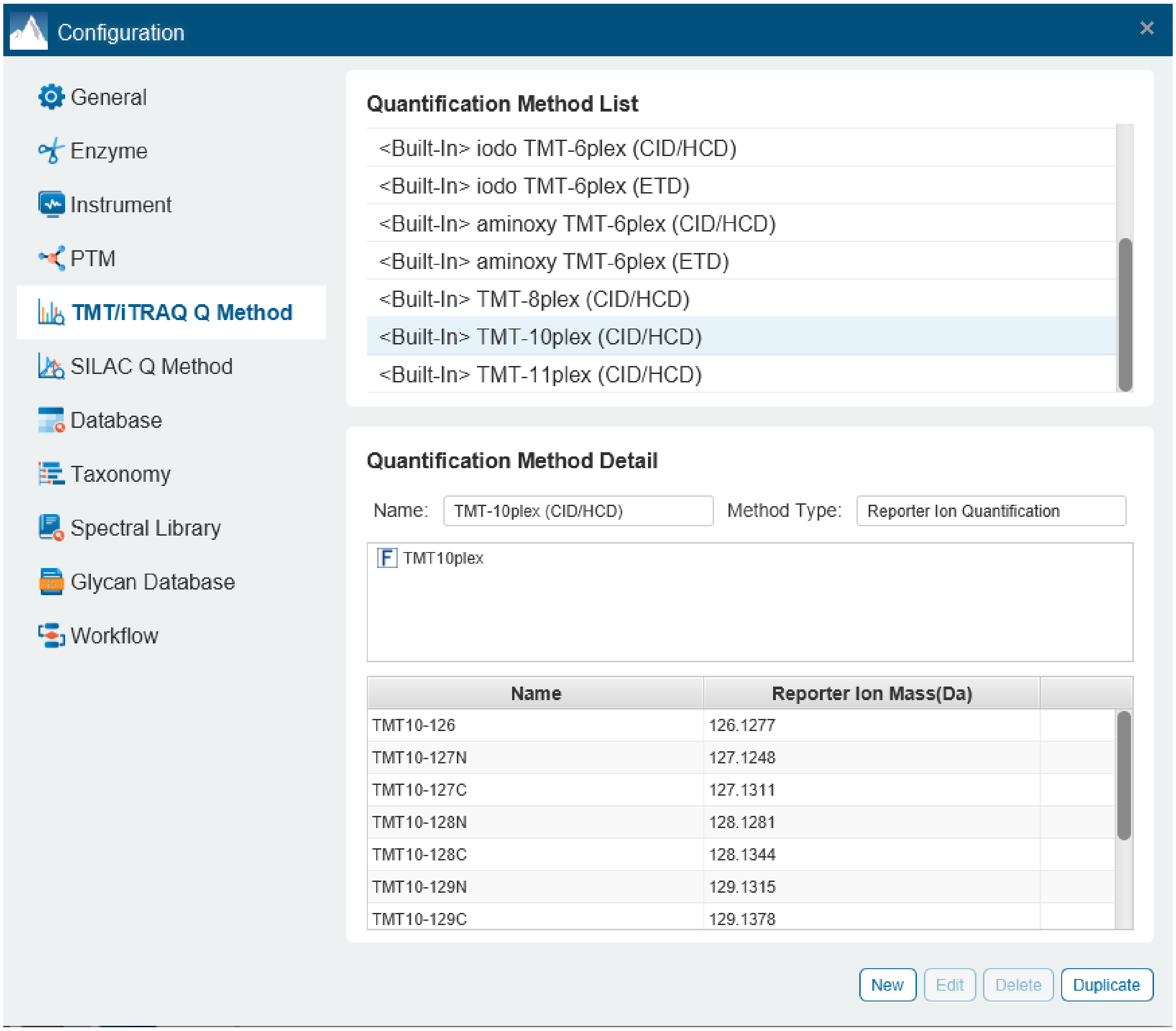

PEAKS Studio comes with a number of predefined labelled quantification methods for TMT and iTRAQ quantification. Custom label Q methods can be defined by users.

Built-in Label Q Methods. The built-in label Q methods within PEAKS Studio come standard with all installations and can not be modified. To modify a built-in label Q method click the “Duplicate” button which will create a new label Q method (it will not modify the original method) with the same settings as the built-in method, which can then be modified.

Create a new Label Q Method. Click the **New** button to create a new label Q method.

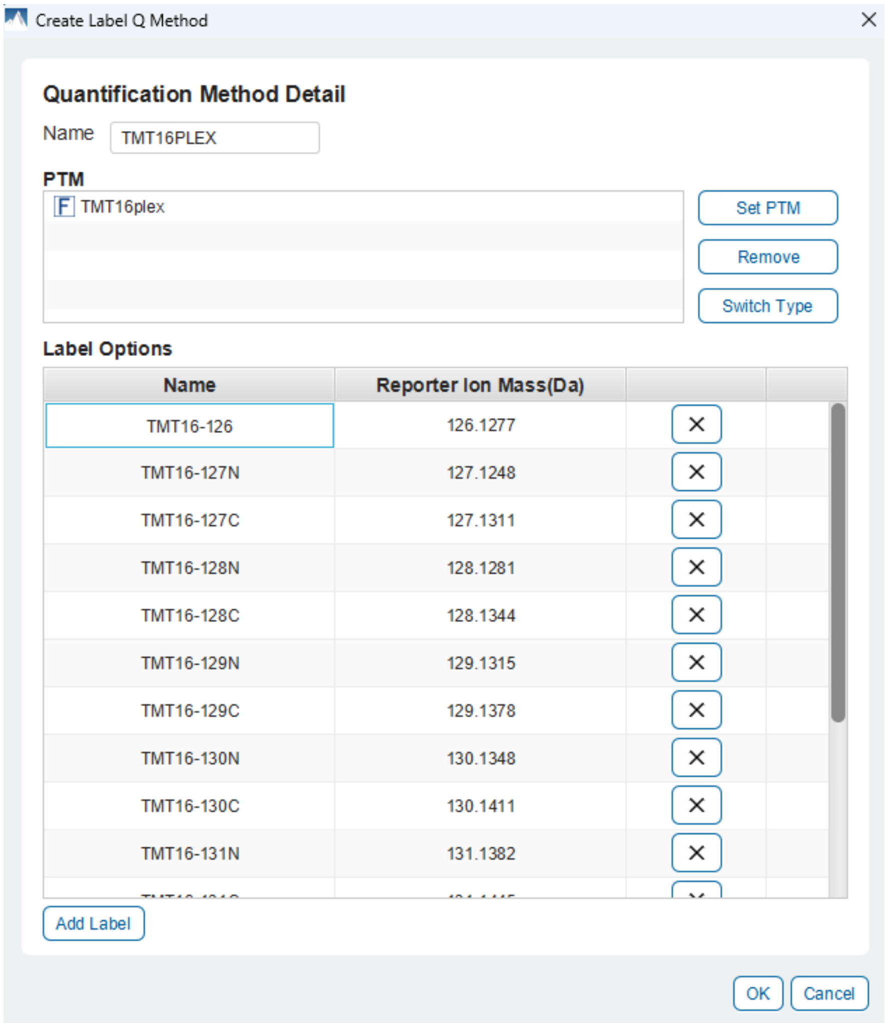

PTM: The modifications used as labels for this label Q method. Click the Set PTM button to open the PTM options and set the modifications for this label Q method.

Label Options: The labels used by this label Q method. Each label must include a:

• Name: (e.g.: iTRAQ-114). This will be the name associated with that channel in the labelled Q parameter page and on labelled Q result pages.

• Reporter Ion Mass: (e.g.: 114.11 for iTRAQ-114). The mass for this channel's reporter ion.

Click OK to save this method into the configured reporter ion quantification list.

Edit a custom Label Q Method. To edit a label Q method, click the "Edit" button to edit any fields.

Delete a custom Label Q Method. The user can delete the custom label Q Methods, but not built-in label Q methods. Click on the "Delete" button to remove the custom method.

2.6 SILAC Q Method Configuration

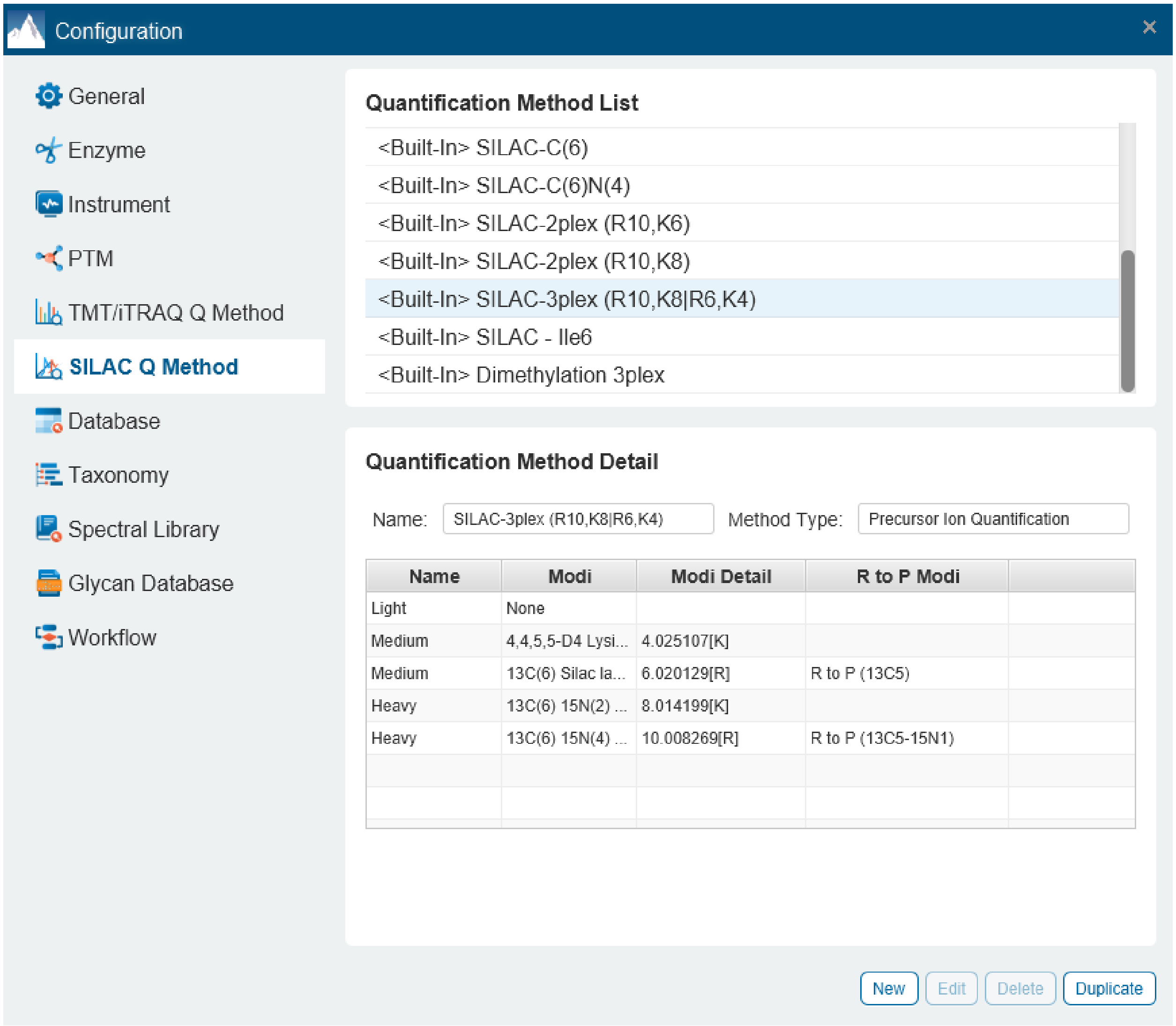

PEAKS Studio comes with a number of predefined SILAC quantification methods. Custom SILAC Q methods can be defined by users.

Built-in SILAC Q Methods. The built-in SILAC Q methods within PEAKS Studio come standard with all installations and can not be modified. To modify a built-in SILAC Q method click the **"Duplicate"** button in the SILAC Q method's actions column. This will create a new SILAC Q method (it will not modify the original method) with the same settings as the built-in method, which can then be modified.

Create a new SILAC Q Method. Click the "**New**" to create a new SILAC Q method. Fill in the settings below and click **"OK"** to save this custom method.

Quantification Method Detail Name: This is the name that will appear in the SILAC Q methods list for future use after it is saved. If selecting an existing name, it will replace that method in the list. Names with built-in methods cannot be used and will not replace built-in methods.

Click on Add Row to add additional labels and set the Label Options as follows:

• Name: (e.g.: Light). This will be the name associated with condition in the SILAC Q parameter page and on SILAC Q result pages.

• Modi: The modifications used as a condition for this SILAC Q method. Click the button to open the PTM Options dialog in the Artificial tab and set the modifications for this SILAC Q method.

• Modi Detail: Shows the selected modification's monoisotopic mass and residue site.

Delete a custom SILAC Q Method. The user can delete the custom SILAC Q Methods, but not built-in SILAC Q methods. To delete a SILAC Q method find it in the Quantification Method List and click on the "**Delete**" button.

Edit a custom SILAC Q Method. To edit a SILAC Q method find it in the list and click the **"Edit"** button. All fields are editable, and if the method name is changed, it will save a separate custom SILAC Q method and keep the original as well.

2.7 Database Configuration

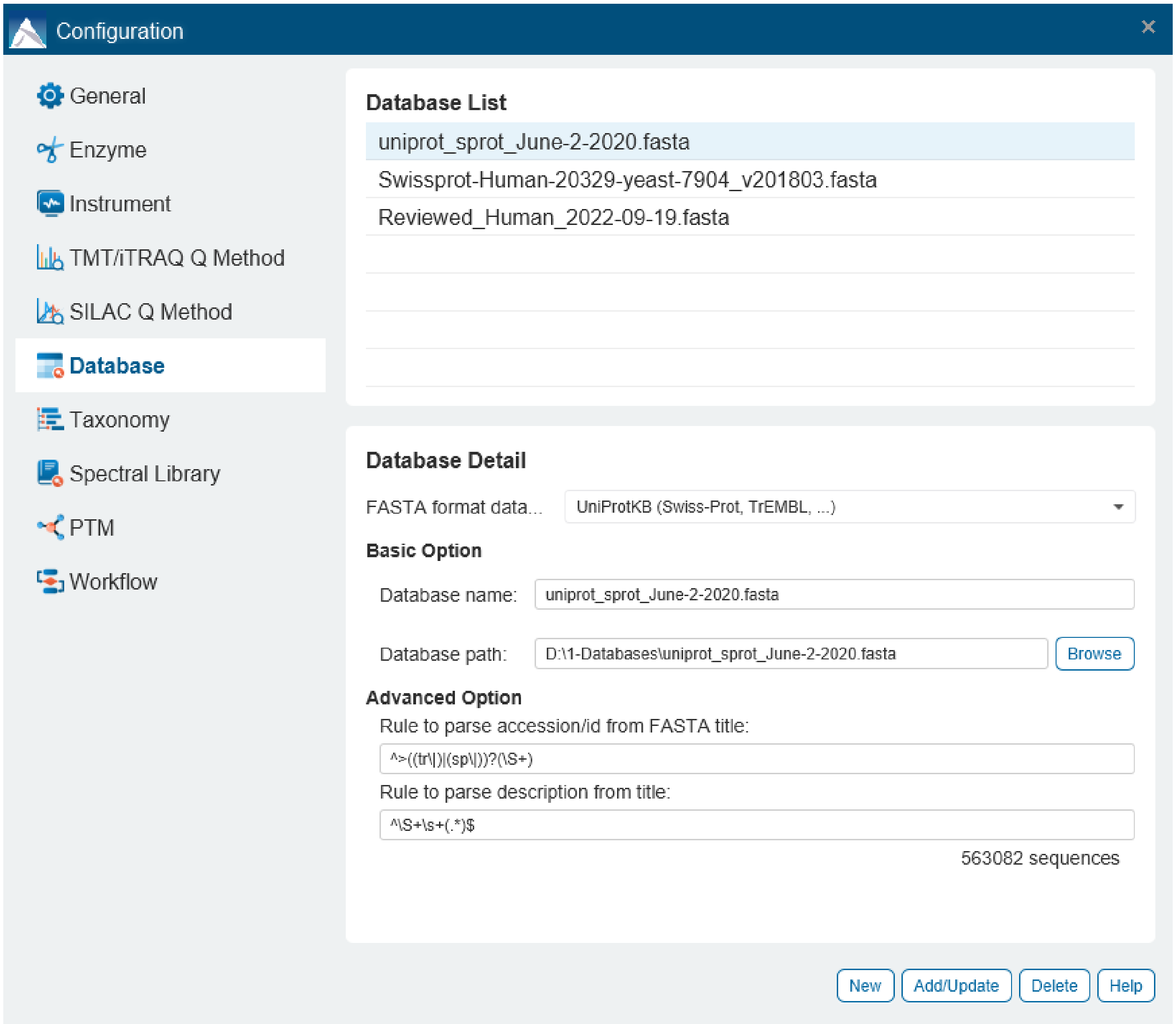

The “Database List” on the top shows all the configured databases in the system. Select a database from the list to view the detailed information about the database in the “Database Details” panel.

Create a new database. Click the New button to create a new sequence database entry or select a database from the “Database List”. The database can be configured in the “Database Details” area below. Click the Add/Update button to save any changes.

Note: Databases can also be configured while creating a project in the Project Wizard.

Moving/Updating a database. To move a database to another directory, the location must be updated in PEAKS. Select the database and specify the new location using the Browse button next to the “Database path” field. Then, click Add/Update to save the new settings. If the database location is invalid, an error pop-up will be shown and the change will not be saved.

Delete a previously configured database. To delete a database file, select the database to be deleted from the “Database List” and click the Delete button at the bottom.

Note: A copy of the fasta database is copied into the installation folder ***db*** when configured. This is the main reason PEAKS Studio installation directory will occupy more disk space over time as each database file configured can be roughly two times the size of the original database.

Note: NCBI Database configuration can take a very long time depending on the size of the database. Please prepare for PEAKS Studio to be busy during that time and it is recommended to configure NCBI Databases when not actively using PEAKS Studio for other purposes.

2.8 Taxonomy Configuration

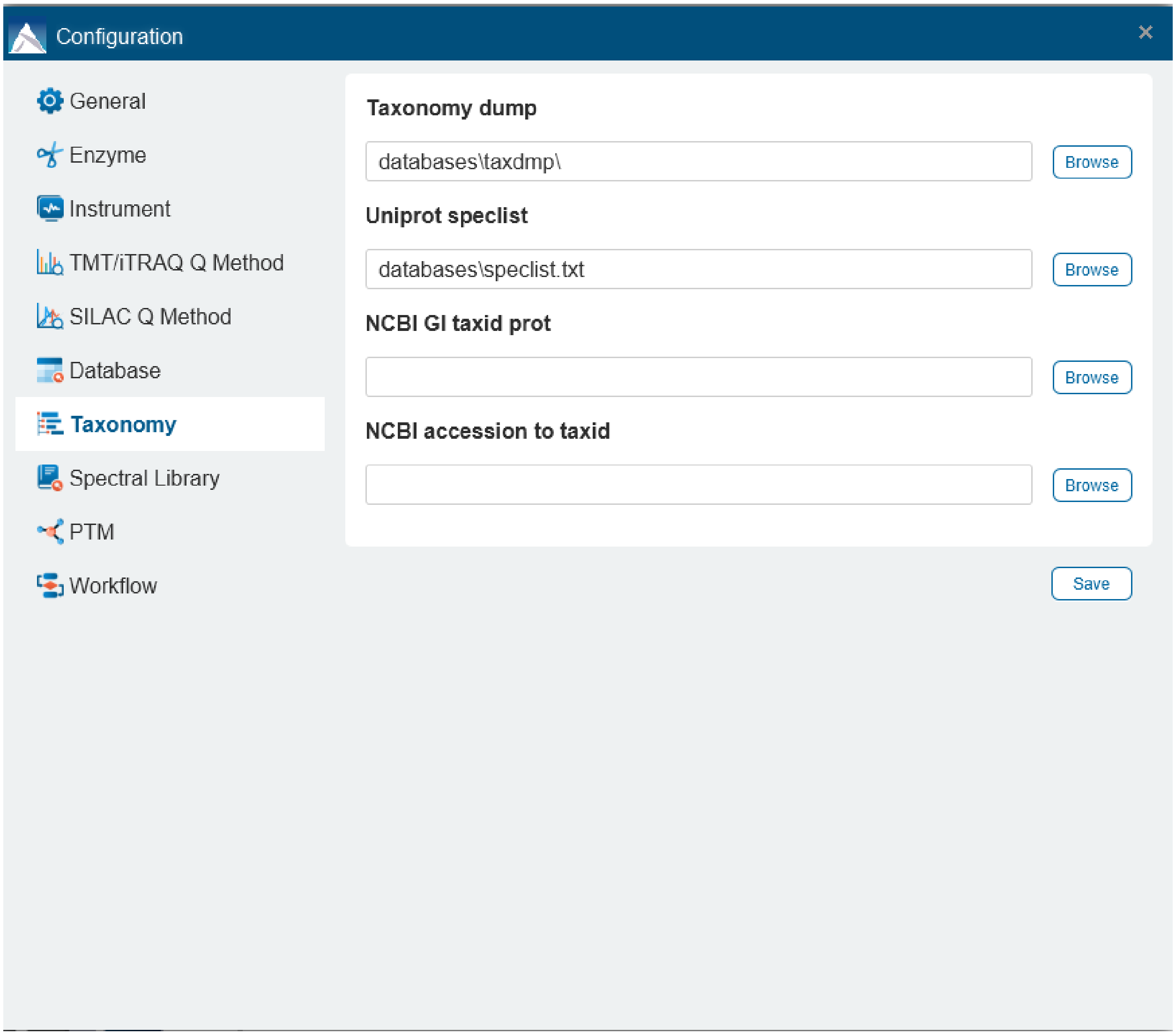

The PEAKS Studio build bundles a taxdmp and speclist in the ***databases*** folder of the installed directory. Clicking on the **“Browse”** button next to the text field will allow user to specify the path to a different taxdmp folder or speclist.txt file. For NCBI databases, set gi_taxid_prot.dmp file for the NCBI GI taxid prot and set prot.accesssion2taxid.gz file for NCBI accession to taxid.

2.9 Spectral Library Configuration

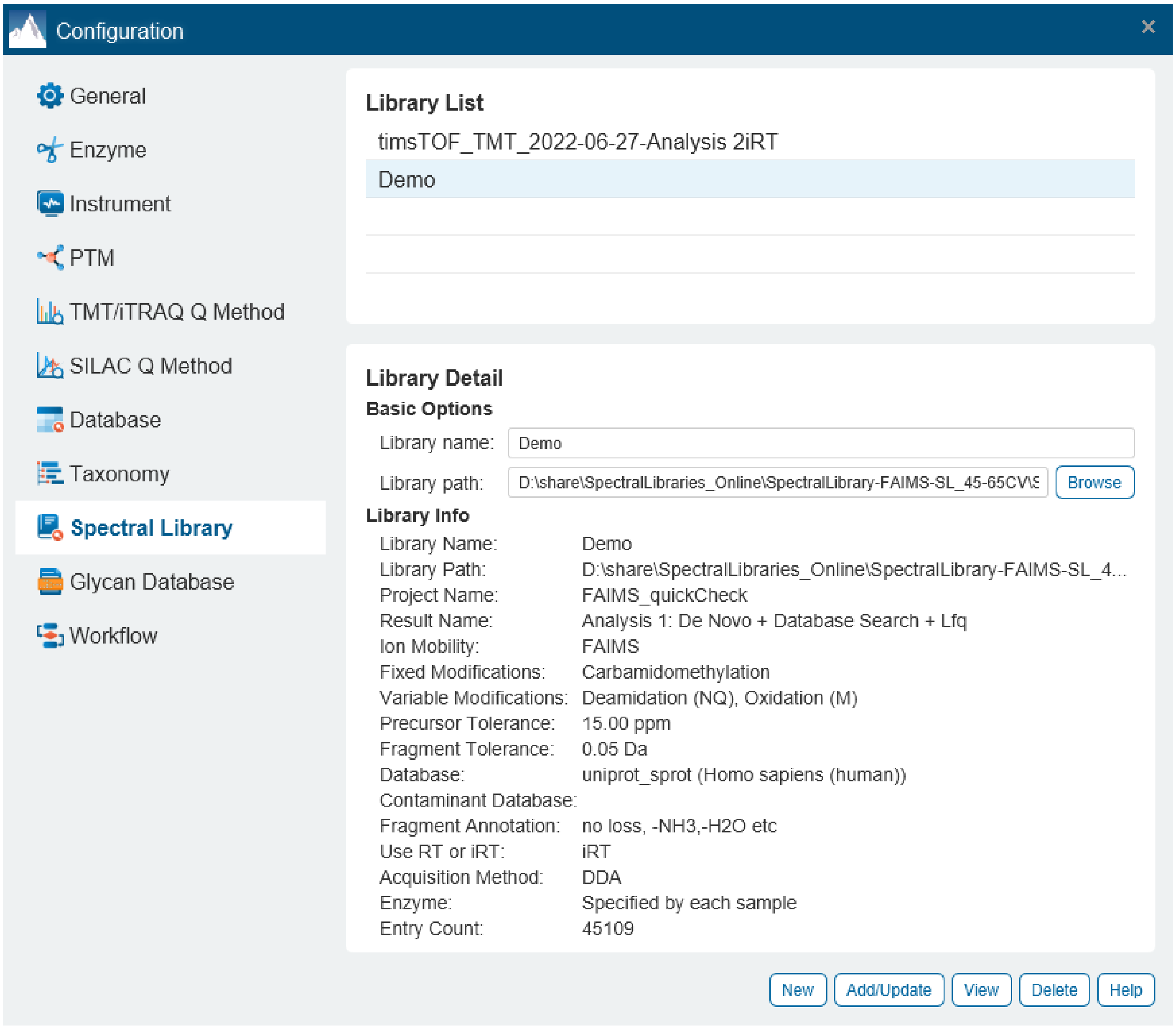

The “Library List” shows currently configured spectral libraries that can be used in PEAKS Studio DIA library search. Click on **“Browse”** Library path to select a valid library file and give it a name in Library name. Clicking “**Add/Update”** will configure this library with the name set, which will replace an entry if that name already exists.

Important to note that certain library files compatible with PEAKS Studio 11 can be configured. They are as follows:

- PEAKS Studio generated spectral library, which is exported from the Database Search result. See Section 6.8.4 PEAKS DB Search result - Exporting - Spectral Library generation for how to export this library. Select the **.tsv** file as the Library path, and if there is a **.info** file in the same path, it will also be read and additional information about the library will be shown.
- PEAKS Online generated spectral library, which is downloadable from PEAKS Online. Similar to PEAKS Studio libraries, select the .tsb file as the Library path, and if there is a **.info** file in the same path, it will also be read and more information about the library will be shown.
- PEAKS Xpro generated spectral library, which is exported from the Database Search result from the previous PEAKS Studio release. However, before being able to configure this library, it will have to be

converted into a compatible library format using the Library Viewer tool (see 12.2 Spectral Library Viewer).

Click on **“New”** to clear the Library path and Library name. Clicking on **“View”** will open the Library Viewer tool. More details on this will be covered in 12.2 Spectral Library Viewer

Click on **“Delete”** to remove that library from configured library list.

2.10 Glycan Database Configuration

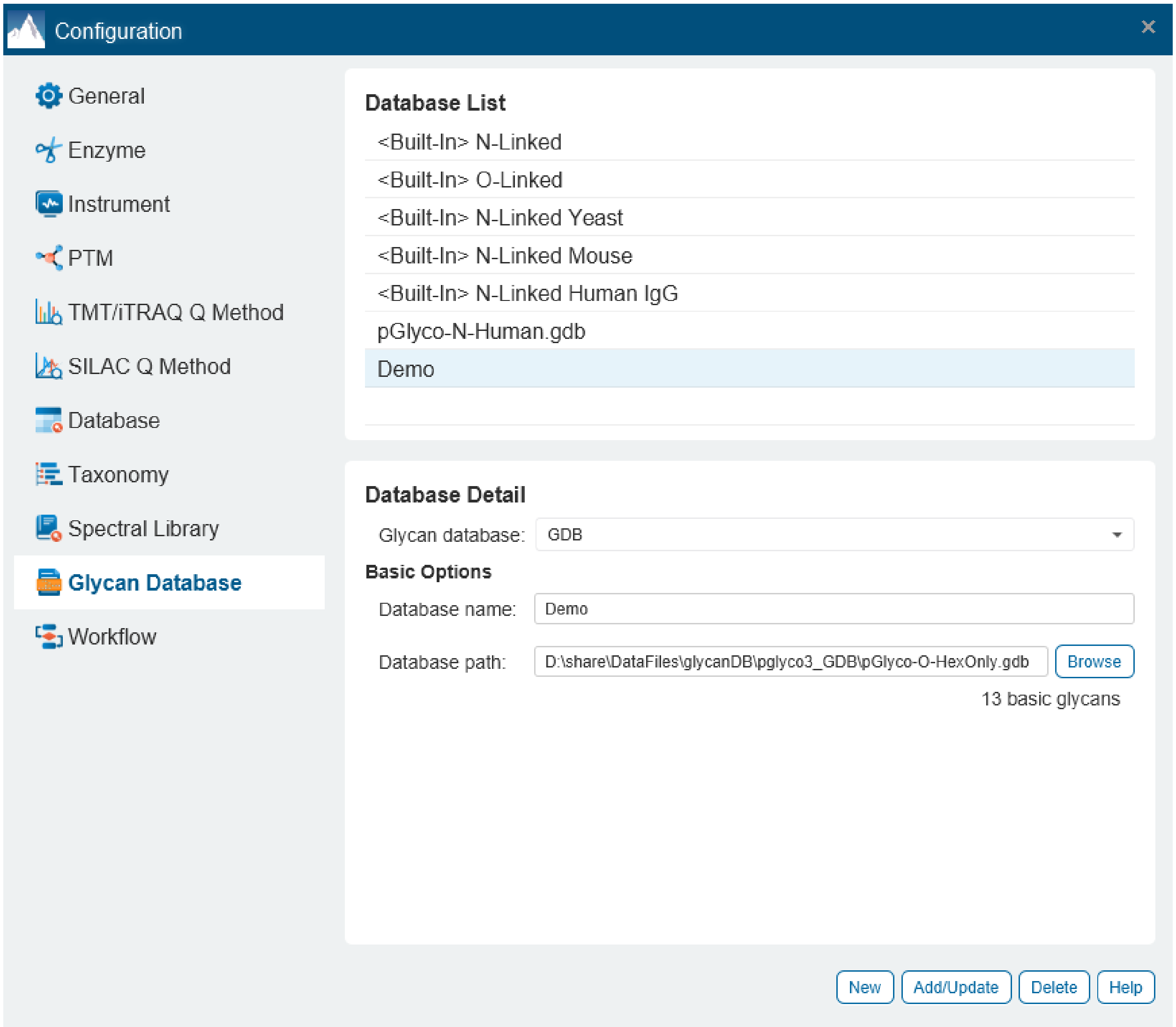

Much like protein databases in section **2.7 Database Configuration**, Glycan databases can also be configured. PEAKS Studio 11 supports **GDB** and **GlycoCT** formats. Select a database by clicking on **“Browse”** next to Database path and give it a name. Click **“Add/Update”** to configure the selected glycan database. If the name is already in the glycan database list, it will replace that configured database. If the name is shared with a built-in database, it cannot be configured as built-in databases cannot be modified or removed.

Click on **“New”** to clear the Database name and Database path for a new entry.

Click on **"Delete"** to remove the configured glycan database. Built-in databases cannot be removed.

2.11 Workflow Configuration - Saved Workflows

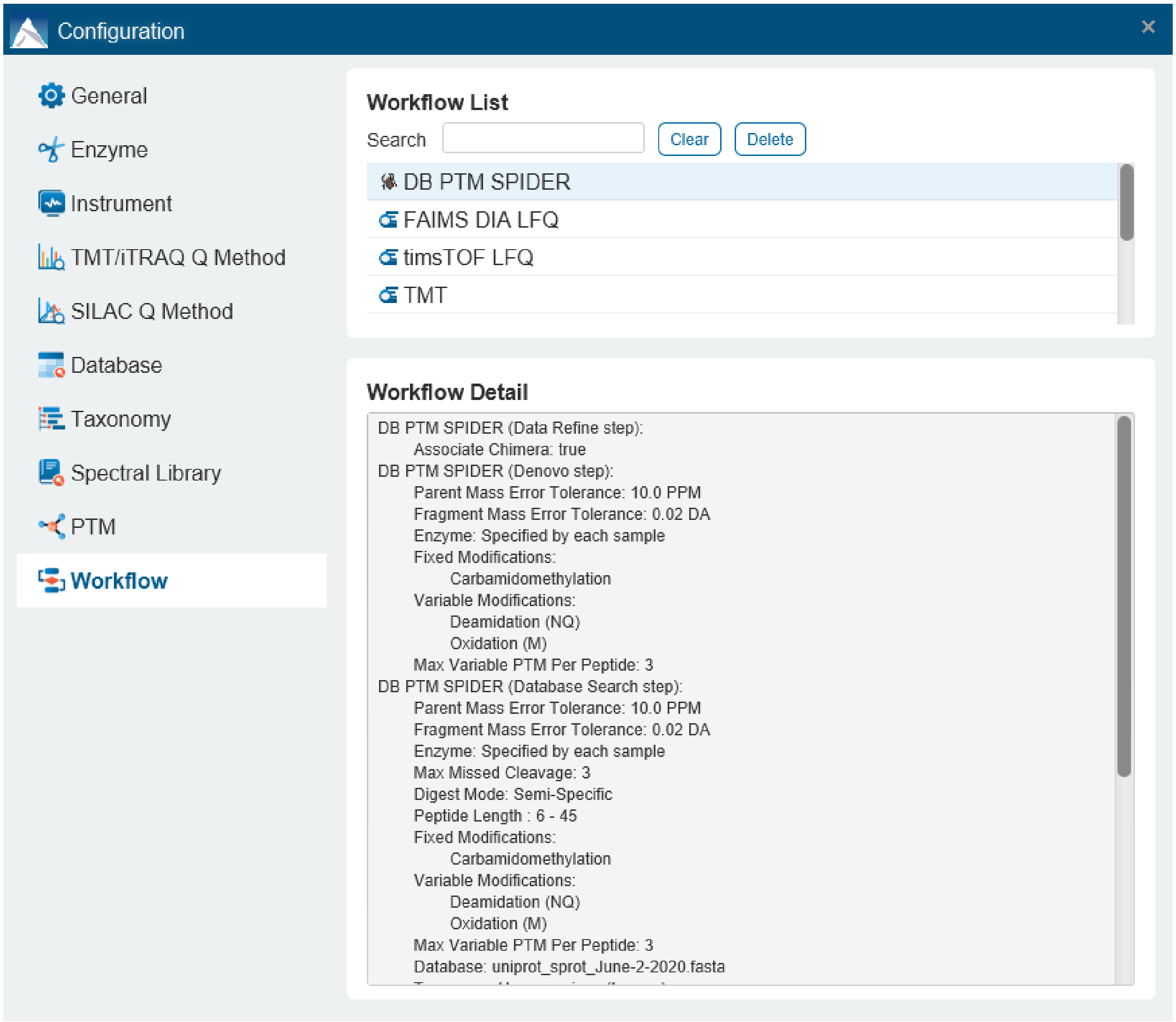

Saved workflows are managed in the Workflow Configuration. To create a new saved workflow, check the Save Workflow option in the "Report" step of any workflow in the Project Wizard.

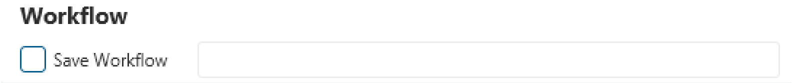

Select a workflow from the "Workflow list" to view more details on the parameters set for that workflow which will be automatically filled in the project wizard. A search box is provided for easy filtering of saved workflow names. Click on **"Clear"** to reset the search box.

Clicking the **"Delete"** button will delete any selected workflows in the Workflow list. A confirmation dialog will appear to ensure this is not done by mistake.

2.12 Performance Configuration

The Performance Configuration is accessed by clicking on the button under **Configurations** tab. It will list your computer resources including total physical memory and number of processors. Allocated resources for PEAKS Studio is automatically calculated based on your computer's specifications and can be modified at any time. Be sure to leave some processors and memory free to allow other programs to function as normal while PEAKS Studio is open. The **Detach Service JVM** checkbox is selected by default. It allocates 2 of the total threads and 8GB of the total allocated memory specifically for the User Interface updates. Unchecking this may have performance issues when using PEAKS Studio, and it is only recommended to be disabled on computers with low thread count (8 or less) and less memory (32GB or less).

Click **"Apply"** to save your preferred settings, and restart PEAKS Studio for the changes to be applied.

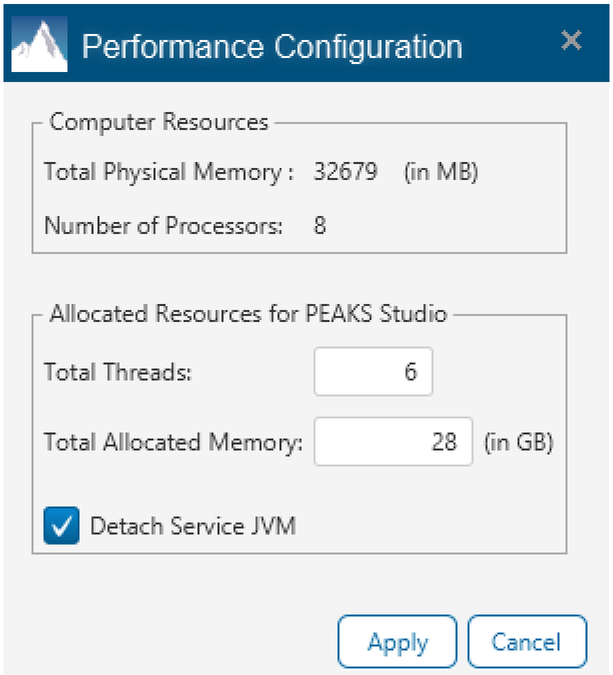

3. Data View & Data Refine View

The data view & data refine view gives a visualization of the mass spectrometric data loaded into the project. It allows for the inspection of LC-MS/MS details in an intuitive way for quality control and troubleshooting issues.

In the Data Refinement View, the information of a typical DDA LC-MS/MS data file is organized into 3 sections:

- MS - This section shows the TIC (total ion chromatogram) plot and all the MS scans. For each MS scan, the corresponding MS/MS scans are also displayed.
- MS/MS - This section lists all the MS/MS scans. For each MS/MS scan, the corresponding features are listed below, and associated number of features is displayed next to the MS/MS scan information header.
- LC/MS - This section provides a bird's eye view of the whole LC-MS dataset with detected features and MS/MS scans marked on refined data.

After opening a data file by double-clicking the data node from the Project View pane, the choice of different views can be made by choosing different tabs in the upper-left corner of the data view window. Hover mouse over an opened Data tab to see a tooltip of which data file it is and from which analysis.

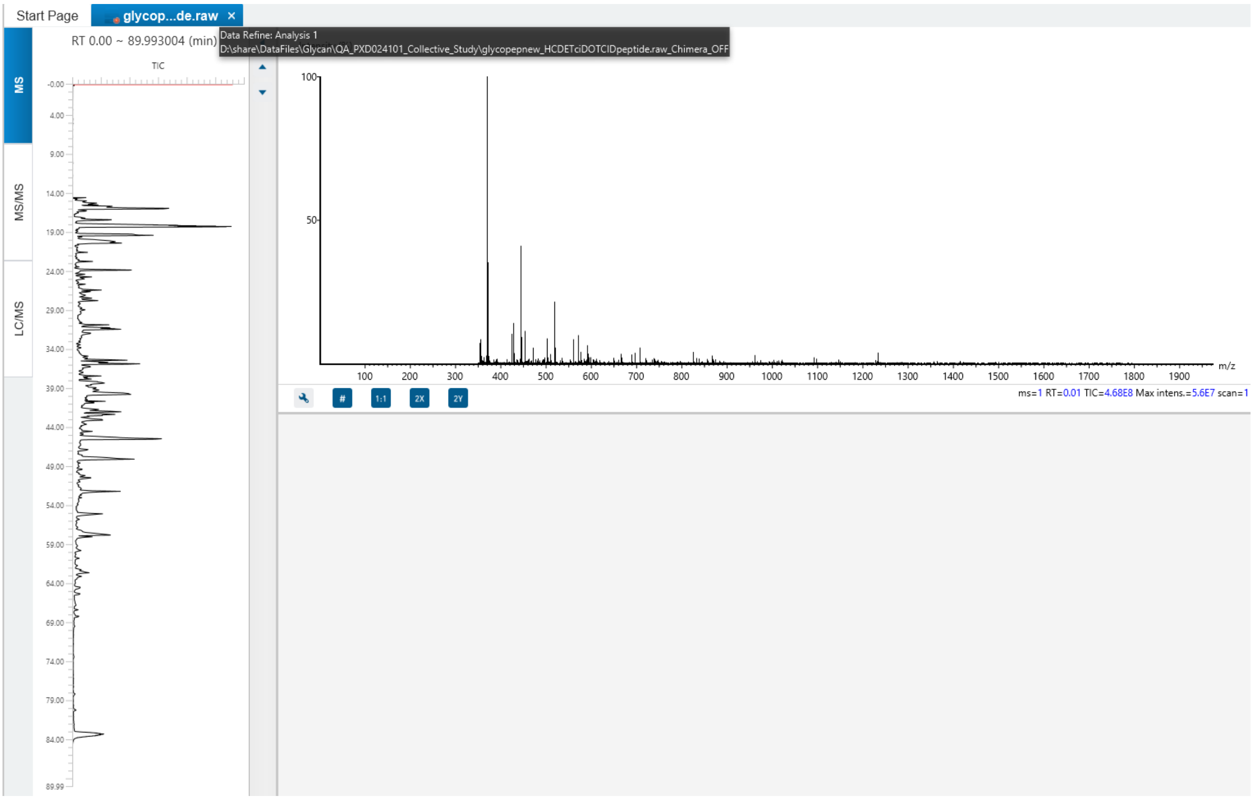

3.1 Data Refinement - MS View

The MS View contains the TIC and all of the scans. The total ion chromatogram (TIC) is displayed on the left of the MS view. The navigation buttons are highlighted in the figure. To collapse the TIC chart, click the left navigation button. Clicking on a specific position in TIC will display the corresponding survey (MS) scan. To navigate the MS scans, use the up and down navigation buttons. The MS scans can also be navigated through using the up and

down arrows of the keyboard. The tandem (MS2) scans associated with the selected MS scan are shown in the panel below, where each can be viewed by selecting the different tabs identified by their "m/z" information.

The survey scans and tandem scans provide a few convenient ways to zoom and navigate the spectrum:

- **Zoom to an m/z region** - Click the desired start m/z and drag horizontally to the desired end m/z, release the mouse button.
- **Zoom in/out smoothly** - Place the mouse pointer at a particular m/z value (right below the x-axis line), scroll the mouse wheel button.
- **Increase/decrease the peak intensity** - Place the mouse pointer on the spectrum, scroll the mouse wheel button.
- **See the whole spectrum** - Double-click on the spectrum or click the **1:1** button.

3.1.1 Data Refinement - MS View - timsTOF Instruments

timsTOF Instruments have a different MS View that includes Ion Mobility information.

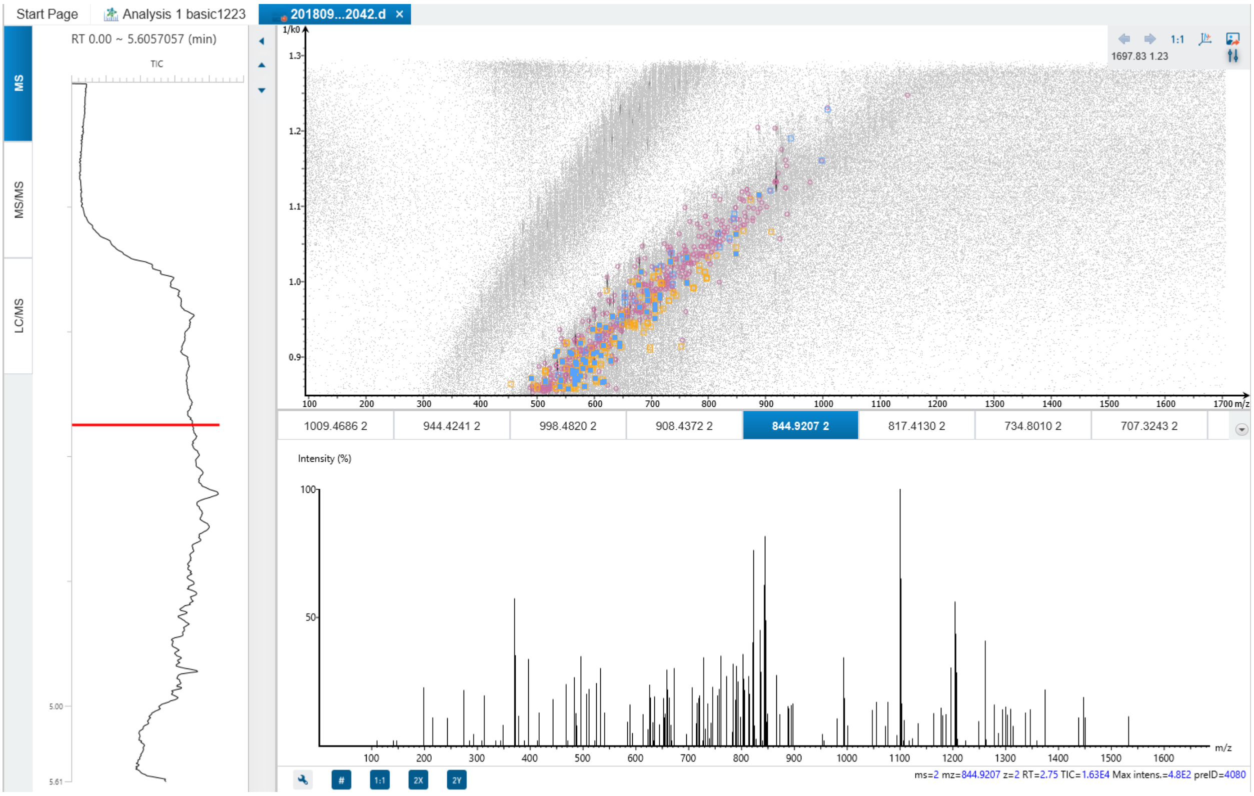

The total ion chromatogram (TIC) is displayed on the left of the MS view. A red bar on TIC curve points the current selected retention time. The top panel at right side shows the MS1 data at the selected retention time as an MS1 Frame - a heat map in m/z-1/k0 dimension.

The precursors associated with the selected MS1 Frame are listed at the bottom panel - one tab per precursor. The MS2 data associated with a precursor are pooled to form a combined MS2 spectrum. Each precursor tab shows the corresponding combined MS2 spectrum.

3.1.2 Data Refinement - MS View - DIA Acquisition

Data Refinement is also available for DIA data. The MS tab is similar to DDA but without any associated MS2 displayed for each MS1 scan. Select both timsTOF instrument and DIA acquisition to run diaPASEF data, which will have a similar MS View.

3.2 Data Refinement - MS/MS View

The MS/MS View shows the list of tandem scans on the left. If data refinement has been performed on the data file, each tandem scan in the list will indicate the total number of associated features at the end of the spectrum header [ex. "695.8360 (RT=26.95 Scan=1348) 2" from the image below indicates that there are 2 peptide features associated with the selected tandem scan]. In addition, when data refinement has been performed, a tandem scan header can be expanded and the associated features and their details (e.g. m/z, RT, charge) are listed below. When a tandem scan is selected from the list, the following information is provided to the right of the list: a summary of the spectral results, the MS/MS spectrum, the zoomed in LC/MS view, the Ion Match Table, and its Survey Scan. Similarly, when an individual peptide feature is instead selected from the list, the result for that feature is displayed. Zoom options are the same as described in the “MS View” section.

- **Summary of Spectral Results:** A table with a summary of the spectral results is displayed here, including the associated feature information, de novo sequence (if de novo sequencing has been performed), and database ID sequence (if a PEAKS search has been performed).
- **MS/MS Spectrum:** A view of the tandem scan will be displayed here. If de novo sequencing and/or a PEAKS search has been performed for the data file, the scan will be annotated. By clicking the sequence results from the Spectral Result table, the corresponding annotation will be displayed. Users will also have the ability to turn on the alignment and error map for the spectrum view when de novo sequencing and/or a PEAKS search is performed.

A search function is provided to search for specific Scan numbers (or precursor ID for timsTOF data files).

- **LC-MS Snapshot:** The LC-MS Snapshot displays a zoomed-in view of the precursor scan in the LC-MS heat map 2-D view. The MS/MS scan is indicated by a red framed square and the span of the yellow line designates the isolation window. If data refinement has been completed, purple circles will also be displayed on the heat map, and selecting an individual feature from the list on the left will highlight the feature in purple.
- **Parent Scan:** The “Survey” tab displays the precursor scan with the best signal within the isolation window. The buttons that appear in this section are identical to those explained in Section 5.2, “MS View”.
- **Ion Match Table:** An "Ion Match" tab will appear when one of the sequence results from the Spectral Results table is selected. The “Ion Match” tab in the bottom pane displays a table with the possible fragment ions for the selected MS/MS scan. Each ion in the table shows the calculated fragment mass, where mass values displayed in color indicate fragment ions present in the spectrum. N-terminal ions are shown in blue and C-terminal ions are shown in red. A fragment ion is found when there is a matching peak within the mass error tolerance, as defined in the de novo sequencing parameters, and when relative intensity of the matching peak is at least 2%. The ion types displayed in the table can be configured in "Spectrum Annotation Settings".
- **Glycan Ion Match Table:** For glycosylated peptides identified using Glycan Search, a Glycan Ion Match table is provided showing the Theoretical m/z and Observed m/z of each Glycan Moiety matched ions.

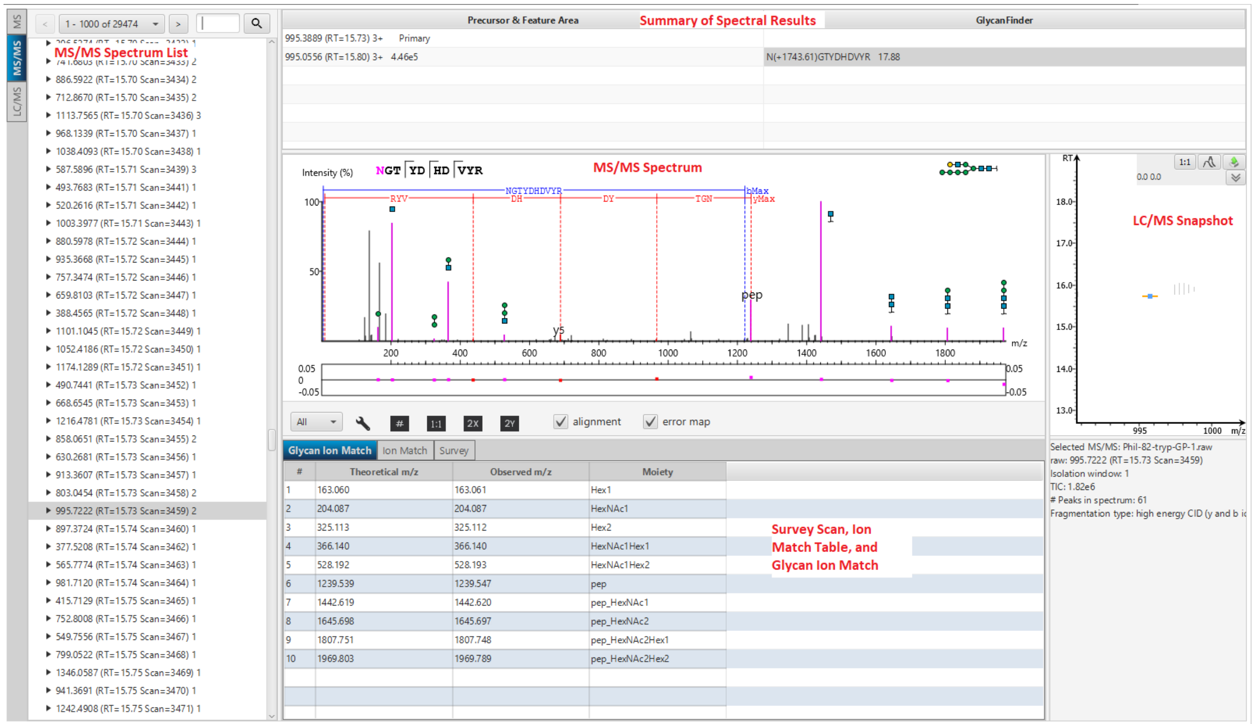

3.2.1 Data Refinement - MS/MS View - timTOF Instruments

MS/MS View for data acquired using a timsTOF instrument includes Ion Mobility information.

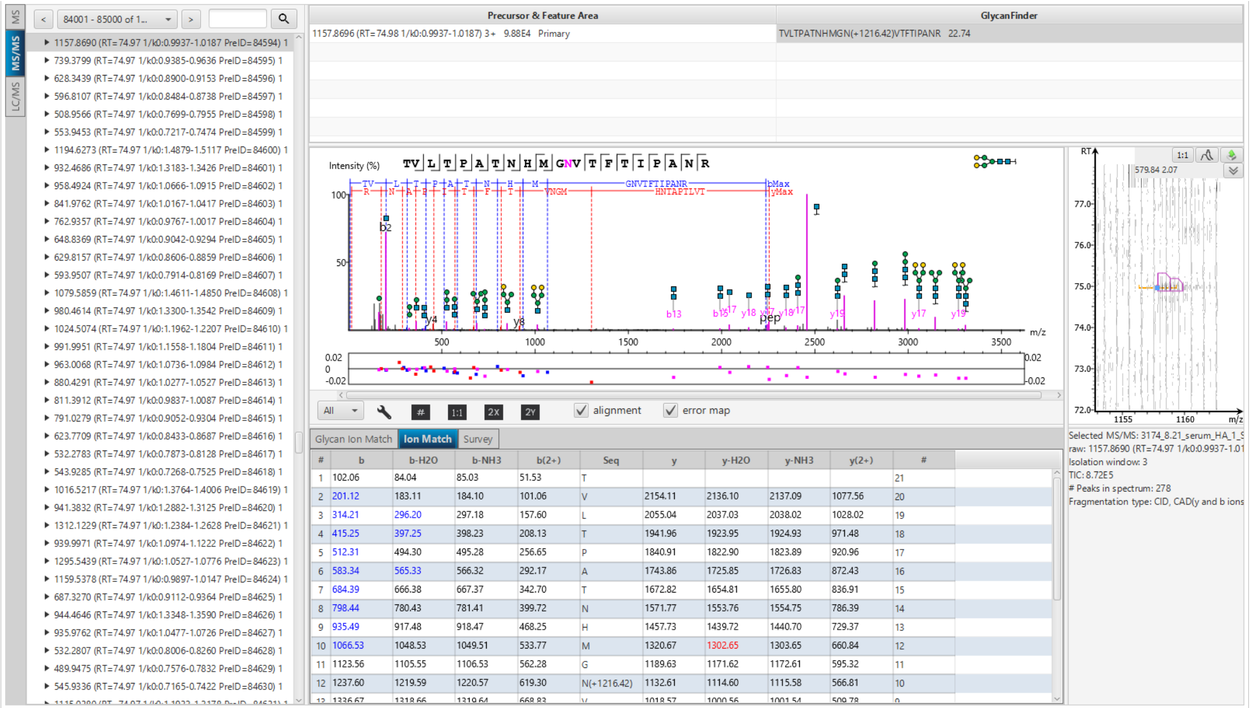

The MS/MS View shows the list of precursors on the left panel. The right panel shows the details associated with the selected precursor. The combined MS2 spectrum is shown in the right middle panel. The MS1 Frame, to whom

the precursor belongs, is displayed at the bottom-right panel zoomed in to show the precursor and the associated feature.

3.3 Data Refinement - LC/MS View

The LC/MS view shows the distribution of LC-MS signals/features as a heat map. Placing the cursor on the heat map will show the m/z value and the retention time of that point.

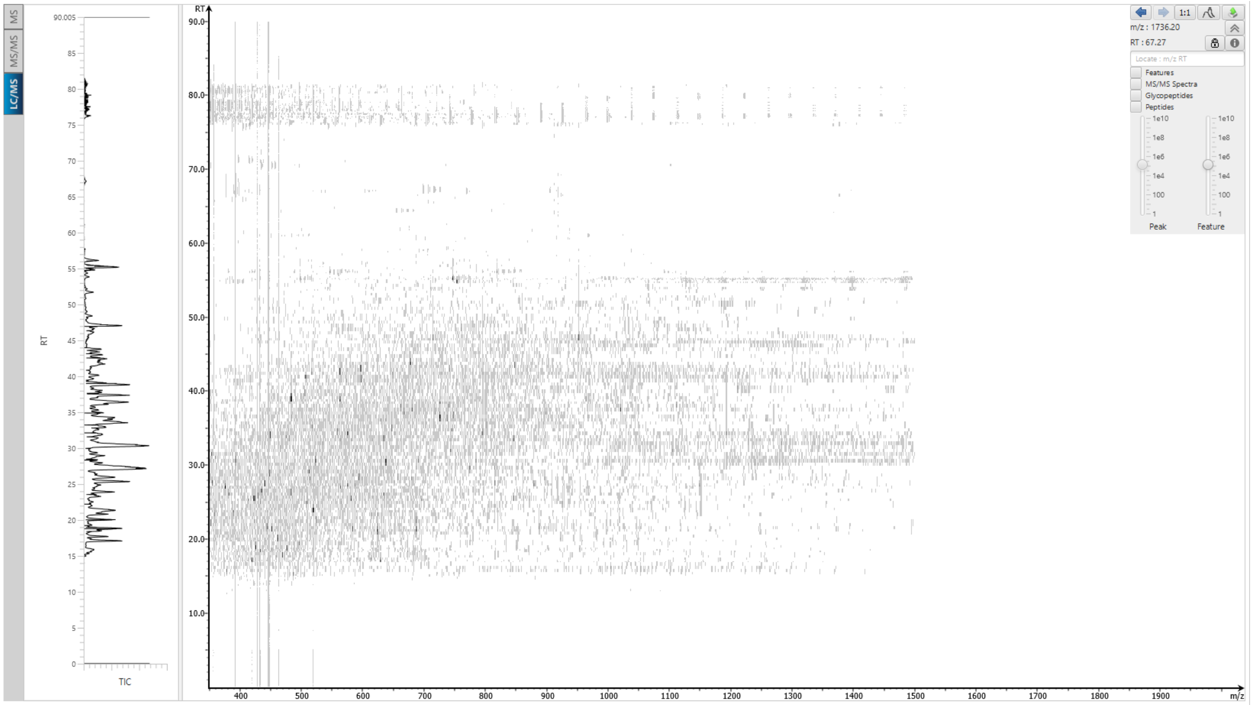

The LC/MS view provides various convenient ways to zoom and navigate the LC-MS features in the data:

- **Move to a specific heat map area:** Click on the heat map, hold and drag the cursor until the desired area of the heat map is reached and then release the mouse button.
- **Zoom to a specific heat map area:** Right-click on the heat map, hold and drag the cursor to select the desired area of the heat map and then release the mouse button.
- **Move to a specific heat pap area from thumbnail:** Current view range of the heat map is shown as a rectangle in the Thumbnail. Click on the thumbnail view range rectangle, hold and drag the cursor until the desired area of the heat map is reached and then release the mouse button.
- **Jump to a specific heat map area:** Provide approximate m/z and RT values separated by a space in the Locate: m/z RT box and then hit the Enter key to zoom in to an area centered at the specified m/z and RT value.
- **Zoom in/out from thumbnail:** Place the cursor on the Thumbnail and then scroll the mouse wheel button.
- **Zoom in/out smoothly:** Place the cursor at a particular (m/z value, RT) position and then scroll the mouse wheel button.

- **Move to a specific RT range:** Click the cursor on the TIC or the RT axis, and then hold and drag the cursor until the desired range is reached.
- **Zoom to a specific RT range:** Place the cursor on the TIC or the RT axis, and then scroll the mouse wheel button.
- **Move to a specific m/z range:** When the m/z axis is zoomed in, click the mouse on the m/z axis, and then hold and drag the cursor until the desired range is reached.
- **Zoom to a specific m/z range:** Place the cursor on m/z axis and then scroll the mouse wheel button.
- **Change contrast of the heat map:** Place the cursor on the heat map, press and hold the Ctrl key, and then scroll the mouse wheel button.
- **Change peak height in the 3D intensity mode:** Place the cursor on the heat map, press and hold the Ctrl key, and then scroll the mouse wheel button.
- **View the whole heat map:** Click the 1:1 button in the control.
- **Move back/forward to previously specified heat map area:** Use the Back and Forward buttons 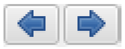 in the control to navigate the previously specified heat map areas.

3.3.1 Additional LC/MS Controls

The heat map view offers various controls to study the LC-MS data features. The following sections describe the control options and their uses in detail.

3.3.1.1 Noise Level

Select the appropriate threshold for noise filtering by peak and/or feature intensities. Once selected, the heat map view will reflect the changes dynamically.

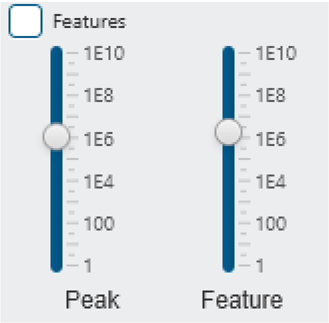

3.3.1.2 Show/Hide Features

To view the detected LC/MS features, mark the Features checkbox in the control. The features detected during the data refinement steps will be highlighted by small, red, diamond-shaped points on the heat map. Placing the cursor over such a feature point will show the boundary of the feature using a red polygon and display a pop-up window that includes the following information: the monoisotopic m/z, the mean RT, the charge state of the feature, and the total area under the XIC curves of the feature.

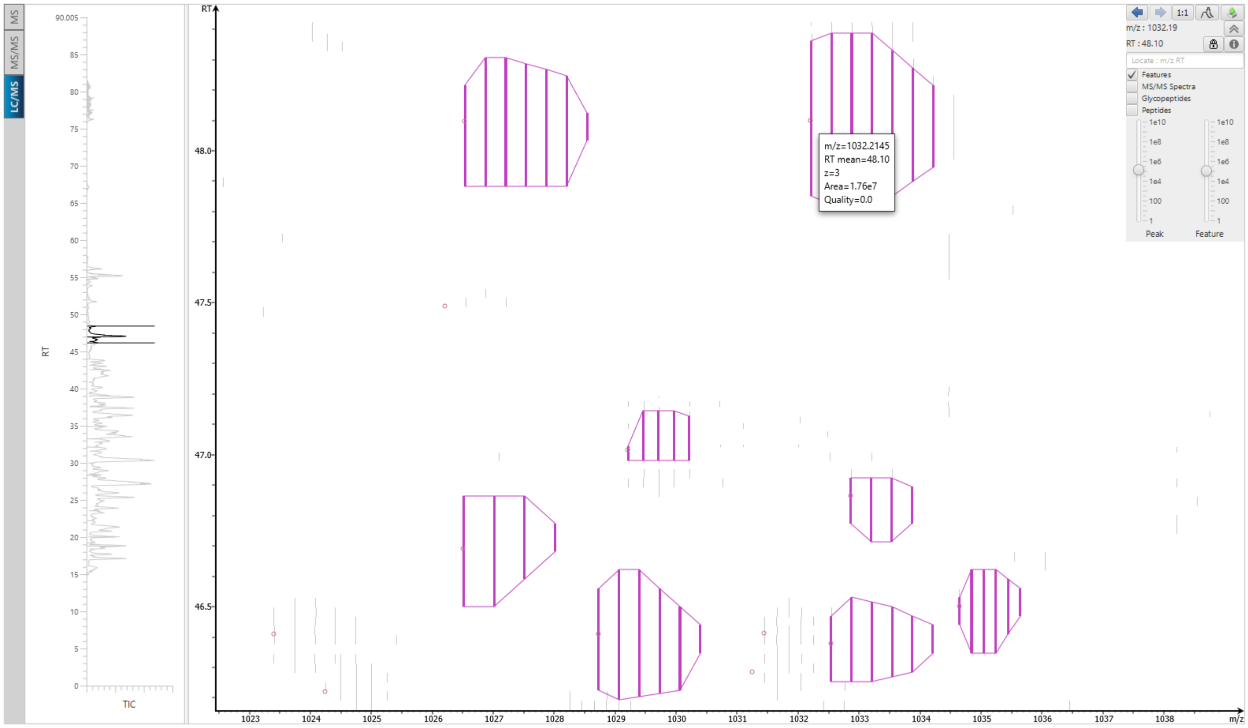

Note: Multiple features can be selected and represented by different colors by holding Ctrl and selecting the feature points.

3.3.1.3 Locate M/Z and Retention Time

Type an m/z and an RT value, such as "618.88 50.5" in the "Locate" text box 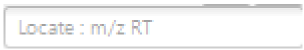

to pinpoint a specific (m/z, RT) position in the LC/MS view.

3.3.1.4 Show/Hide MS/MS Spectra

Select the MS/MS Spectra checkbox to indicate where a tandem scan has been taken. The location will be marked with blue-outlined squares on the heat map. Place the cursor over a square to view the precursor m/z, the RT, the precursor charge state, and the scan number of that spectrum. Click on the square to view the acquisition window (indicated by the yellow line), and the spectrum in a pop-up window. Once selected, the square will become red-outlined.

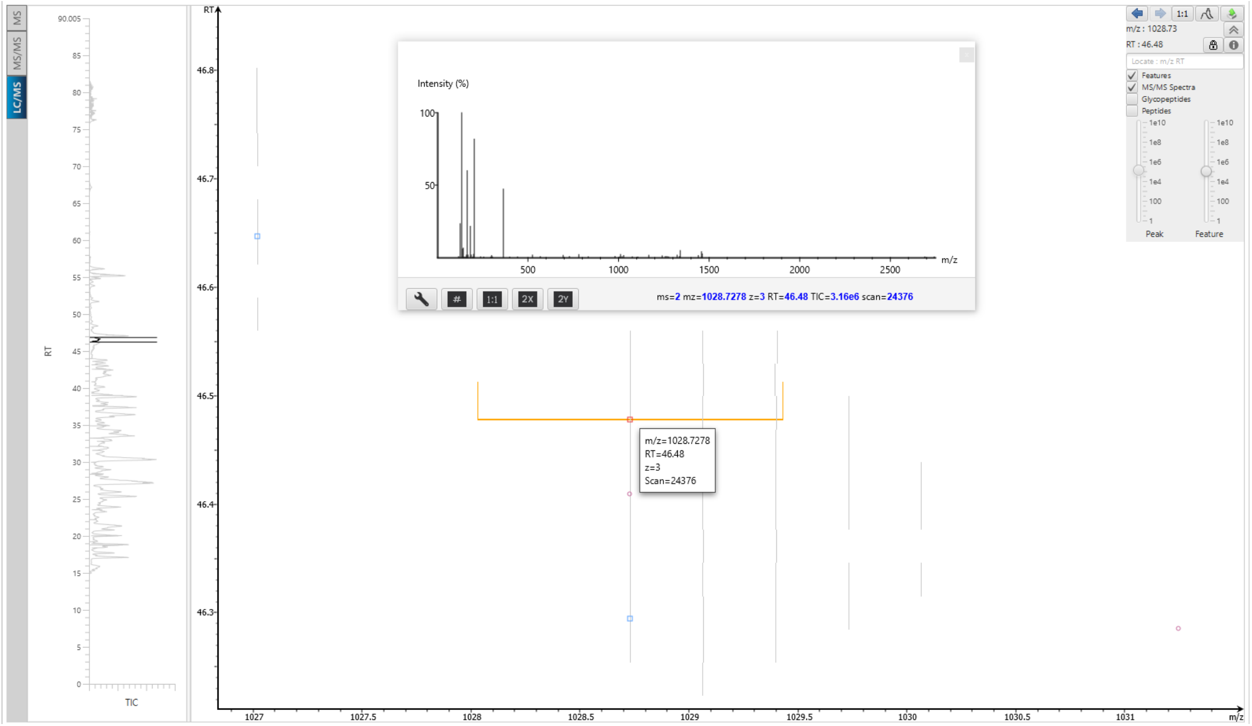

3.3.1.5 Show/Hide Markers

Select the checkboxes to show the markers of where tandem scans are associated with different types of peptides.

- MS/MS Spectra will display all tandem scans. These are marked by empty blue markers.
- De novo tags will display all tandem scans associated with peptides found in De Novo search. These are shown as empty orange markers.
- De novo only tags are associated with the de novo only table in the DB Search result. These are shown as solid orange markers.
- Identified peptides are markers from Database search results such as DB Search, PTM Search, and SPIDER. These are shown as solid blue markers.
- Quantified peptides are markers associated peptides from TMT and SILAC quantification. These are shown as solid green markers.
- Glycopeptides are markers from Glycan Search glycopeptides. The location will be marked with a purple marker for N-linked glycopeptides and yellow for O-linked glycopeptides.

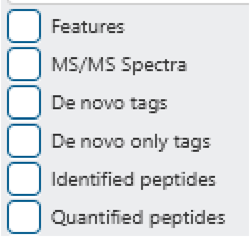

Place the cursor over a square to view more details about that spectrum in the tooltip. Click on the square to view the acquisition window (indicated by the yellow line), and the spectrum in a pop-up window. Once selected, the square will become red-outlined. Note that each of these markers can be jumped to from each of the respective result tables.

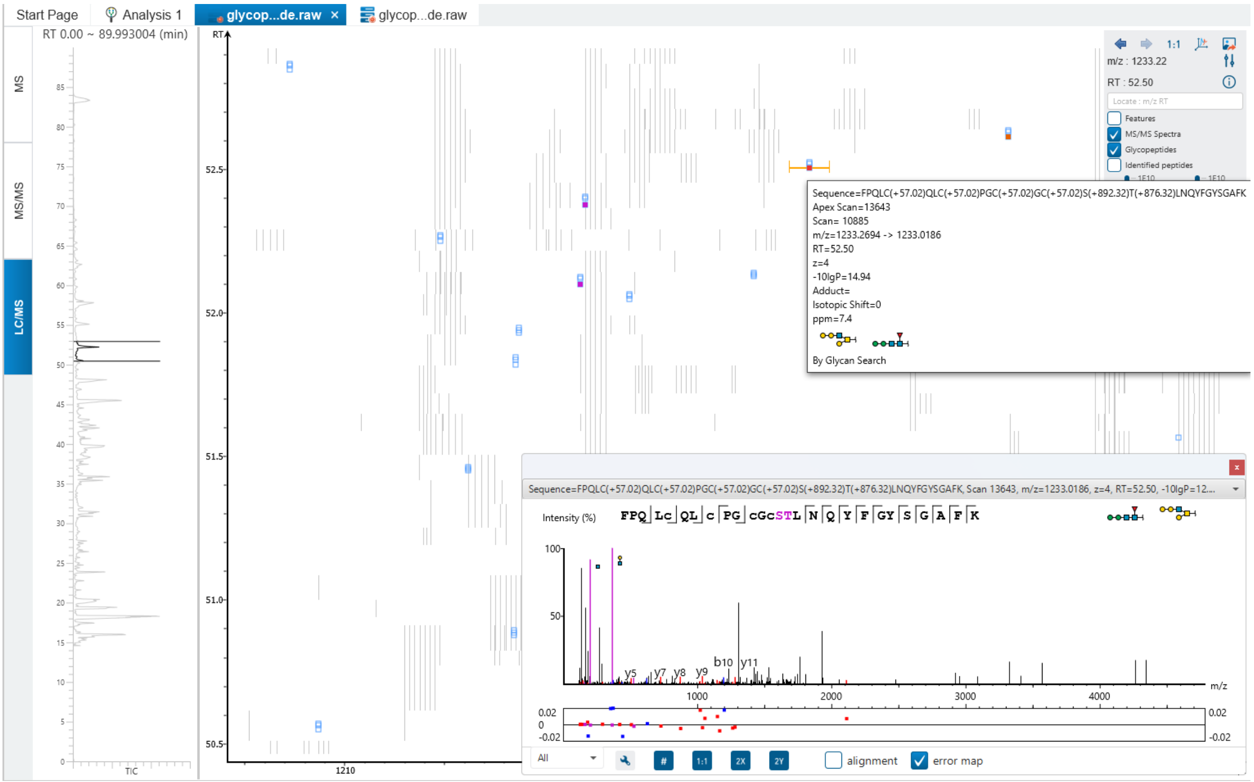

For **FAIMS** data, each marker will have different shades of colour to differentiate between CV values.

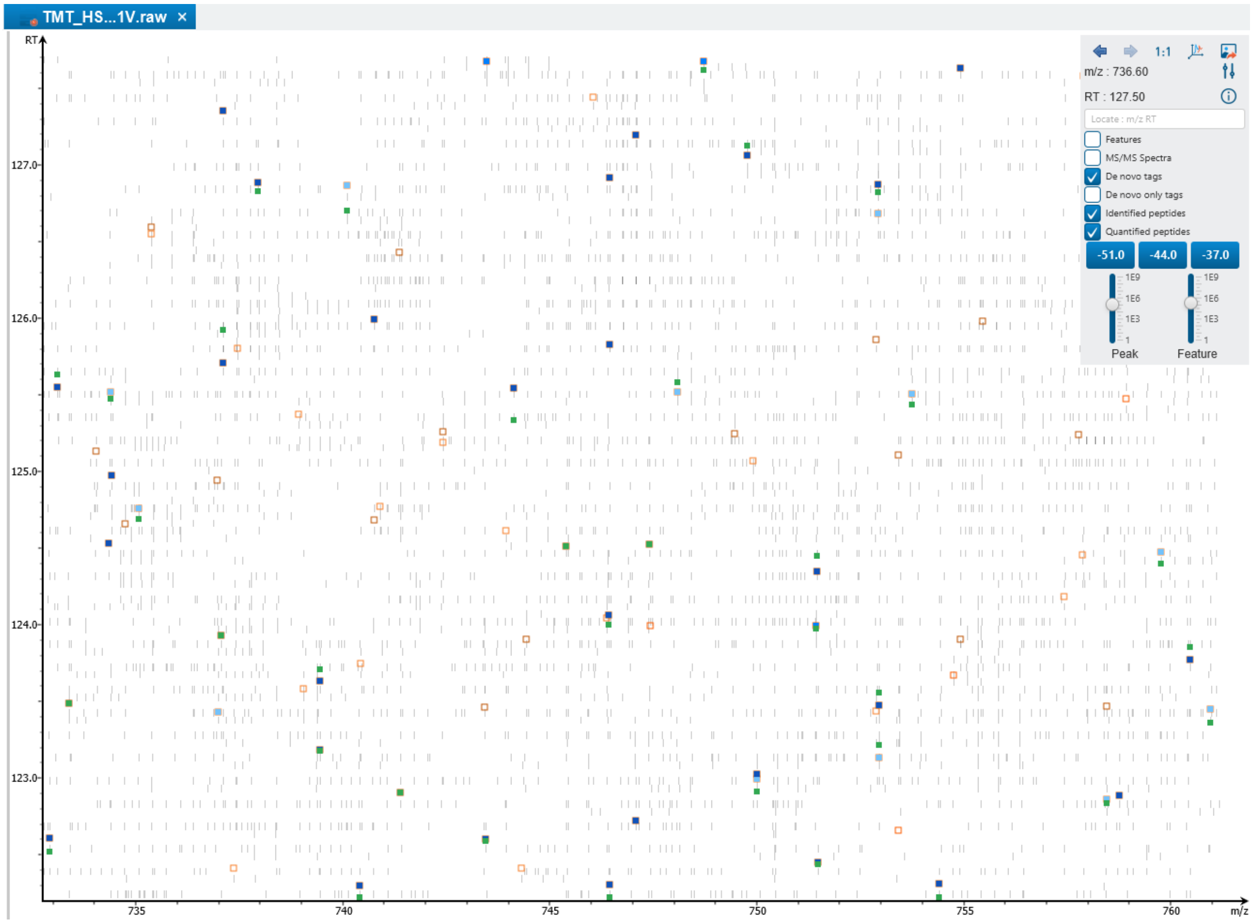

3.3.1.6 Intensity View

In addition to the heat map view, PEAKS also provides a 3D intensity view for visual inspection of the LC/MS data.

To switch between the heat map view and the intensity view, click the 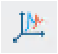 button in the top-right LC/MS control options. The intensity view has the same set of controls to easily zoom and navigate. Click the 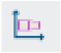 button to switch back to the 2D view.

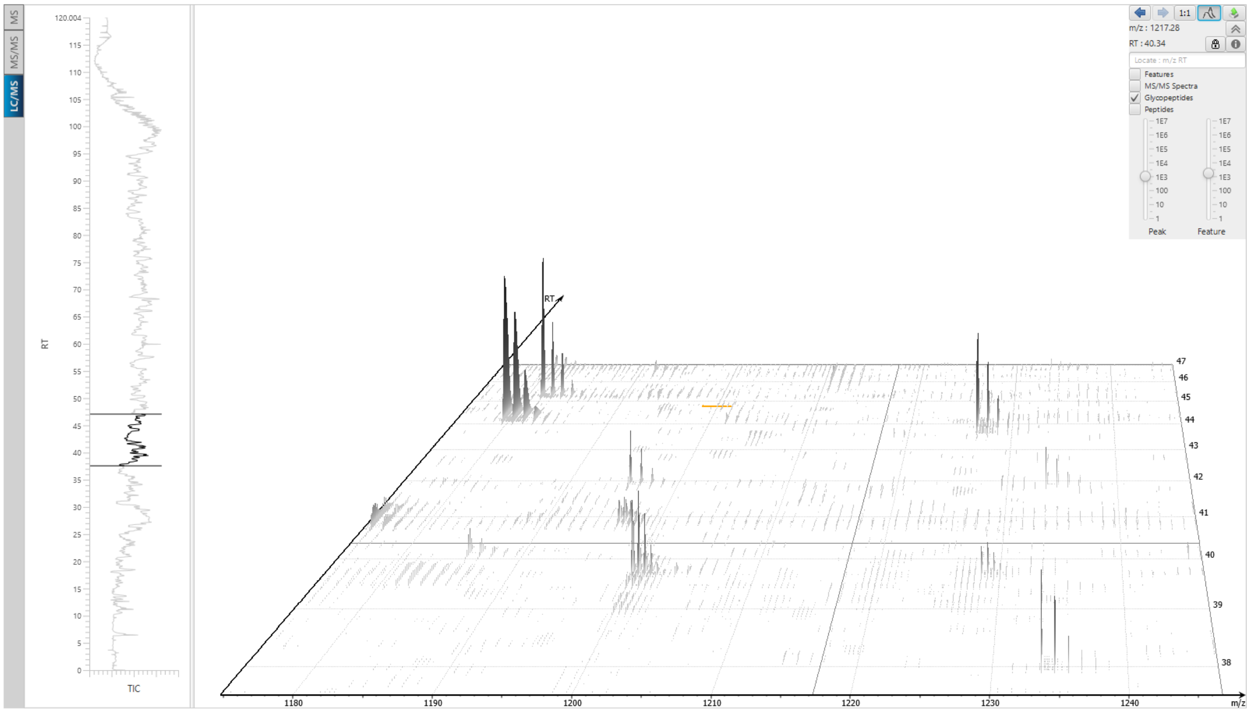

3.3.1.7 Navigation History

The left and arrow buttons in the control panel provides the option to switch back and forth to previous views such as zoom levels, m/z or RT ranges, 3D view, and different noise level filters.

3.3.1.8 Export LC/MS

Click the icon on the LC/MS control options to export the LC/MS heat map or intensity view in PNG format.

3.3.2 Data Refinement - LC/MS View - timsTOF Instruments

The LC/MS view of timsTOF Instruments have an extra IMMS button to view the IM-MS dimension. Clicking on it will show a confirmation pop-up to process data reduction and index creation. This will take some time depending on the size of the data. Once complete, the IMMS pop-up will be shown with 1/k0 on the y-axis, m/z on the x-axis, and the RT range is based on the range in the LC/MS view. Adjust the RT in the LC/MS to adjust the RT range in the IMMS view.

The set of controls and functions in the in IMMS view is the same as in the LC/MS view.

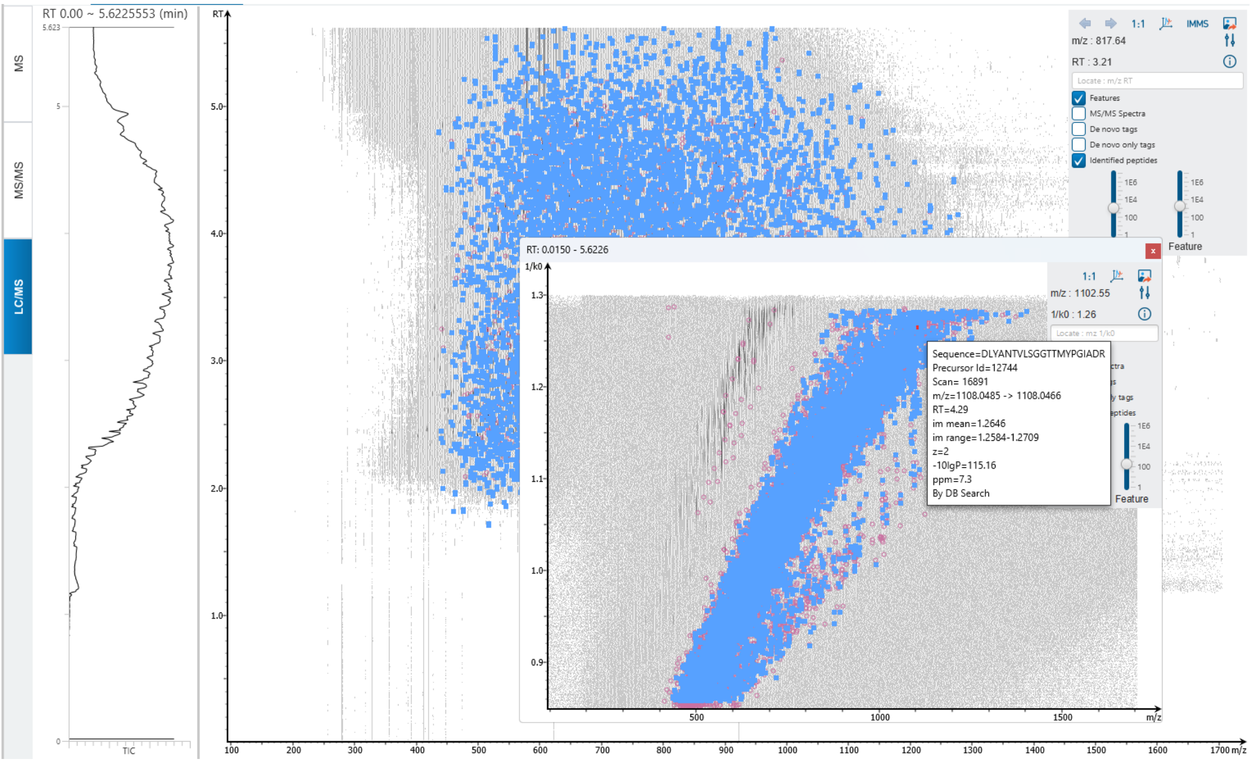

3.3.3 Data Refinement - LC/MS View - DIA Acquisition

The Data Refinement LC/MS view is also provided for DIA data. It only has Features markers and no tandem scan markers. The controls are the same as conventional LC/MS view controls.

3.4 Data View

In the Project View, double-click on a data file under a *Data* node to view the raw data visualisation. This view is similar to the Data Refine view except it is from the raw data. The MS View, MS/MS View, and LC/MS View are all similar to what is shown in the Data Refine result node.

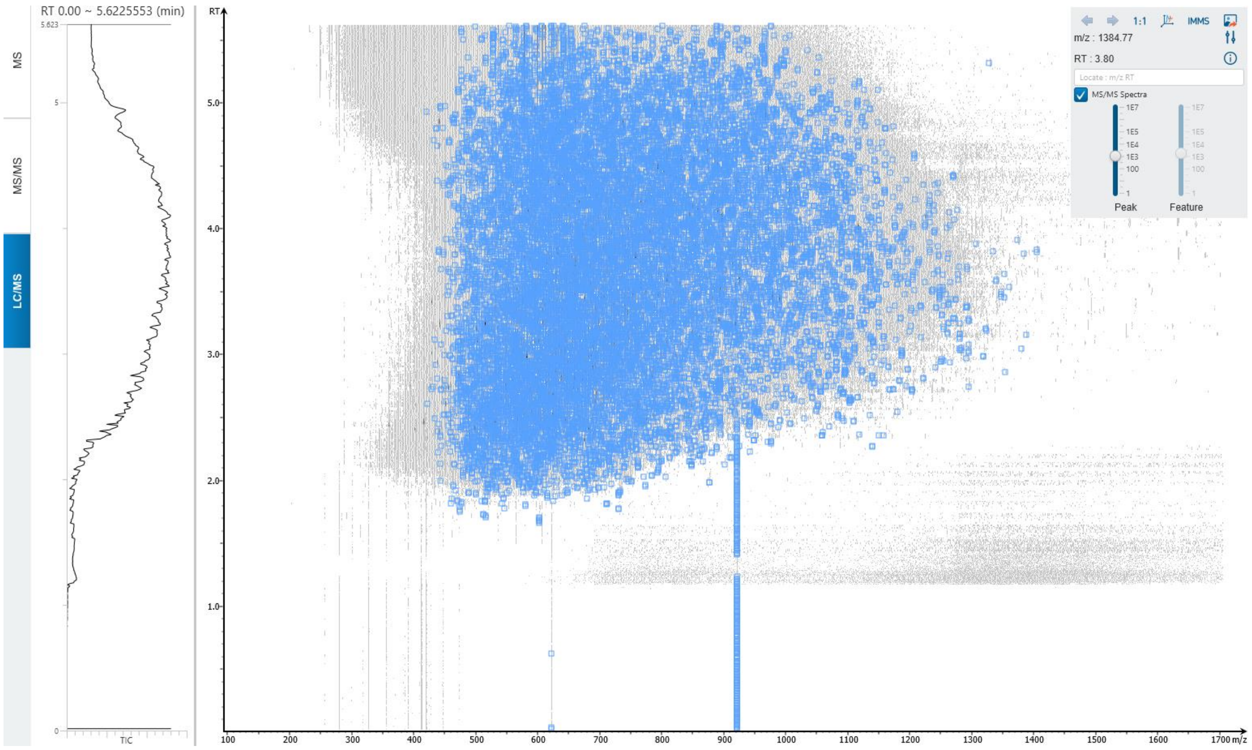

3.5 Project Wizard Data Refine

In the following chapters, each workflow type will be explored. Data Refinement is a mandatory step for every analysis. For DDA workflows, a Data Refine Option "Associate feature with Chimera [DDA]" is provided.

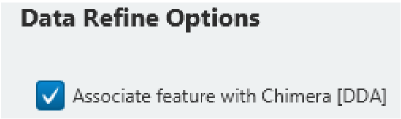

The Data Refinement step has a single option "Associate feature with Chimera [DDA]". If checked, PEAKS will consider a tandem scan can contain two peptides and PEAKS will search for chimera spectra to enable correct inference of co-eluted isobaric peptides.

3.6 Data Exports

When any DDA analysis is completed, an Export node appears with options for Data Exports. In Project View, double-click on 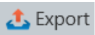 Export to open the Export node. The loaded Data can be exported as Raw MS2 scans or Refined MS2 scans in MGF format.

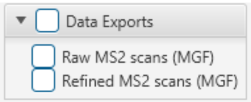

4. Denovo peptide sequencing

De novo sequencing derives a peptide sequence directly from the tandem mass spectrum without the need of a sequence database. It is the preferred method for identifying novel peptides and the study of unsequenced organisms.

PEAKS Studio provides a reliable and comprehensive solution for automated peptide *de novo* sequencing. It

features:

- Accurate de novo sequencing

  The PEAKS Studio *de novo* sequencing algorithm and scoring functions are constantly tuned for each instrument type to ensure optimal accuracy.

- Easy-to-use user interface

  PEAKS Studio generates a comprehensive result summary and provides interactive views of annotated spectra.

- Support of all major instrument types

  PEAKS Studio supports de novo sequencing of spectra generated by various mass analyzers (Ex. Orbitrap, Ion Trap, Time-of-Flight, Quadrupole) and instrument types (Ex. Orbitrap Eclipse with FAIMS, timsTOF, ZenoTOF) in common fragmentation modes (Ex. CID, HCD, ETD, ETHCD).

- Automated result validation

  PEAKS Studio assigns a local confidence score to each amino acid in a de novo sequence. The local confidence score ranges from 0 to 99%, indicating how confident the algorithm considers a particular amino acid residue to be the correct assignment. Moreover, the peptide sequence is evaluated by an ALC (Average of Local Confidence) score, which is the average of the local confidence scores of all the amino acids in the sequence.

Note: For details about the PEAKS *de novo* sequencing algorithm, please refer to the initial publication:

"PEAKS: Powerful Software for Peptide De Novo Sequencing by Tandem Mass Spectrometry", Rapid

Communication in Mass Spectrometry, 17(20): 2337-2342 (2003).

Other PEAKS Studio tools, including PEAKS DB for database search, PEAKS PTM for unspecified PTM search,

and SPIDER for homology search, depend on the *de novo* sequencing result to achieve superior sensitivity and

accuracy.

4.1 De Novo Analysis Workflow and Parameter Settings

To run a De novo Analysis, Select the De Novo option in Workflow Selection

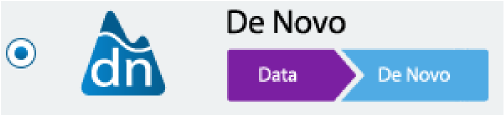

After deciding the Data Refine option, proceed to the next step to see the Denovo parameters.

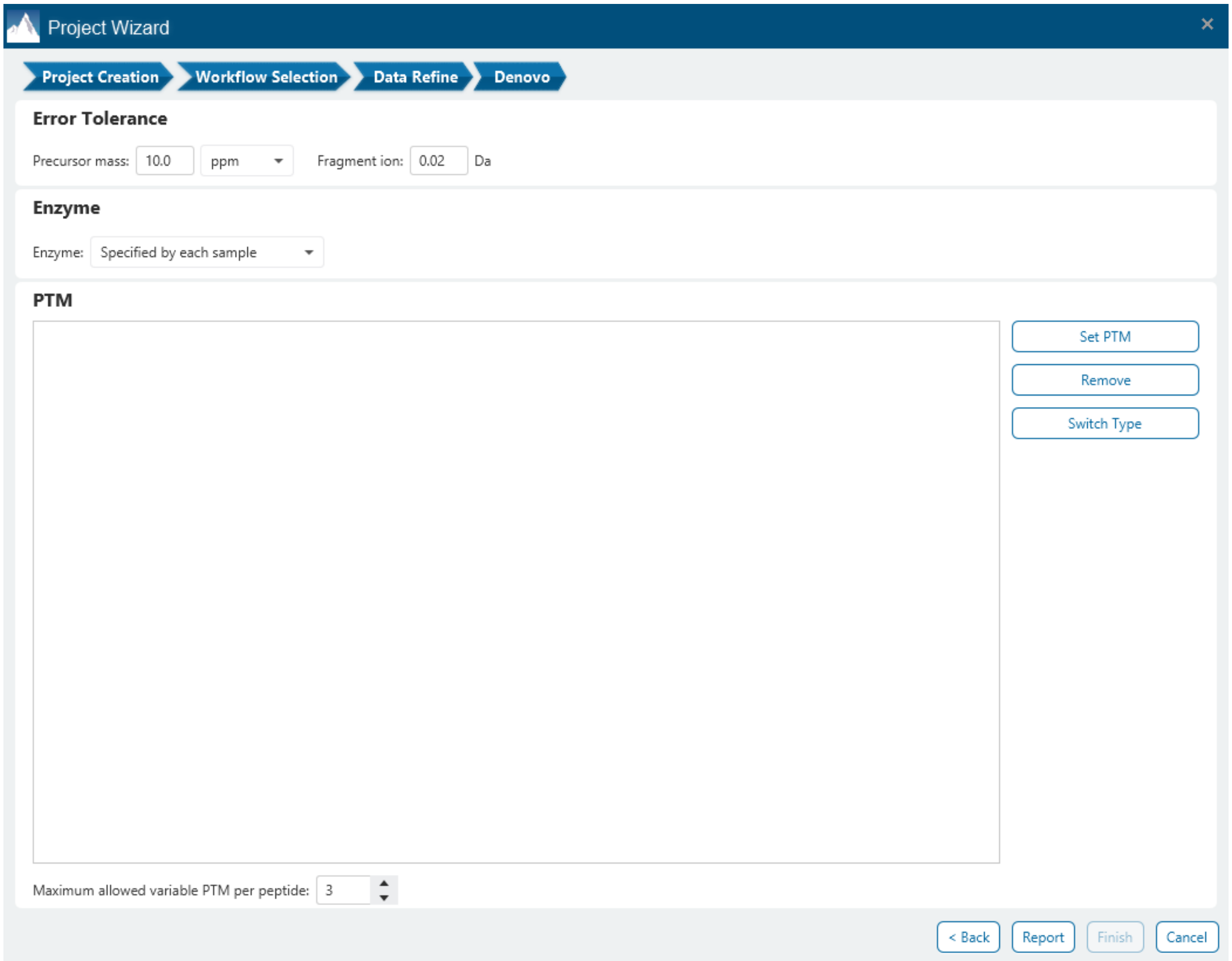

The following parameter settings are provided:

Precursor Mass Error Tolerance: The maximum mass difference between the de novo peptide and the monoisotopic mass of the precursor ion.

Precursor Mass Tolerance unit: PEAKS de novo generates peptides within the precursor mass error tolerance. Precursor mass error tolerance can be specified in either Daltons or ppm.

Fragment Mass Error Tolerance: PEAKS de novo uses this value when scoring de novo peptide sequences. PEAKS Studio considers a fragment ion to be matched if the calculated m/z is within the specified error tolerance. Units are Daltons.

Both precursor and fragment error tolerance parameters need to be set consistently with the mass accuracy of the instrument.

Enzyme Settings: Select the enzyme used for protein digestion from the enzyme drop-down menu. PEAKS de novo respects the enzyme specificity at both ends when generating peptides.

Note: When the selected data node is a project of multiple samples, "Specified by each sample" allows samples to be analyzed separately, using their respective specified enzymes during project creation.

Note: "None" is a special enzyme allowing non-specific cleavage at both ends of the peptide. It is the recommended option when no digestion enzyme was used or when the digestion enzyme exhibits a high degree of non-specificity.

PTM Settings: Click “Set PTM” to open the **PTM Options** dialog and specify the fixed PTMs and variable PTMs expected in the experiment. Highlight PTMs and click on the button to place them as either Fixed or Variable PTM. From there, highlighted PTMs can be removed by clicking the “Remove” button. Clicking “Remove All” will reset the PTM options. Highlighting and clicking “Switch Type” will move the Fixed PTM in into the Variable PTM list and vice-verse. Click on “New” to create a new custom PTM. While a PTM is selected, click on “View” to see more details.

Note: Multiple PTMs can be highlighted by holding down the CTRL key or SHIFT key on the keyboard.

Note: For a specific residue, only one fixed modification is allowed.

Once the PTMs are selected, the maximum number of variable PTMs per peptide can be specified and a value of less than 4 is recommended.

Note: The use of variable modifications increases the size of the computational search space for the de novo sequencing algorithm. It is recommended not to select too many variable modifications in PEAKS de novo. See PEAKS PTM for alternate search options.

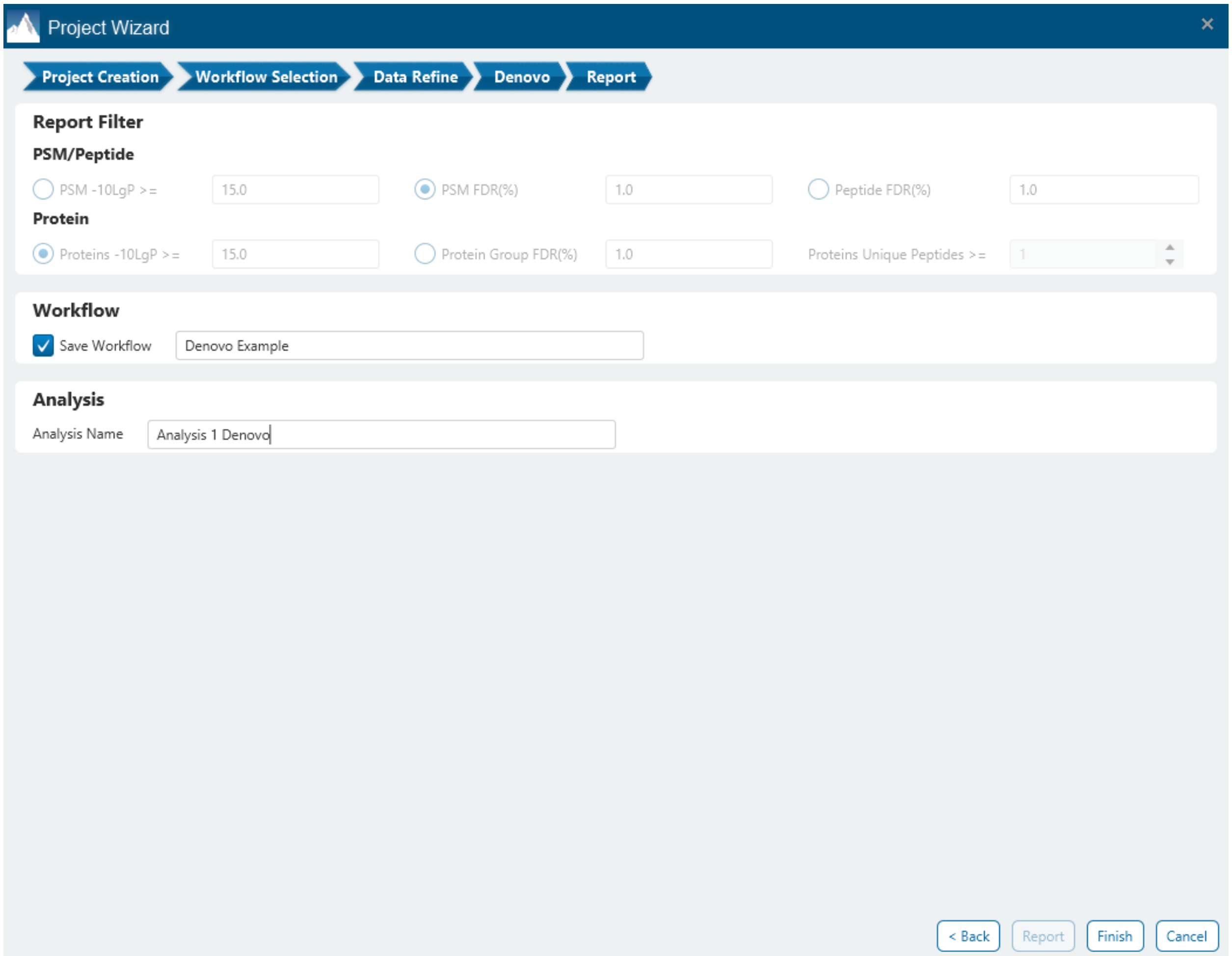

Click on "Report" to proceed to the final step. The Denovo result does not have any Report filters that need to be set. From here, there is the option to specify an Analysis Name (which can be later renamed) and/or decide to save the Workflow to quickly re-use in future analyses.

4.2 Understanding PEAKS De Novo Sequencing Results

When de novo sequencing is done, double-click on the De Novo result node to open the the Denovo result. The result contains three tabs:

- Summary: This shows an outline of the PEAKS de novo search results with key statistics. The overall quality of the experiment can be examined and the filters for de novo ALC can be adjusted at the top of the page.
- De novo: This shows a list of peptide sequences that pass the ALC threshold detected by de novo sequencing.
- Feature: This shows a list of features detected in the refined data. Features associated denovo peptides are easily identified here.

The following sections will explore each of these three tabs in the Denovo result.

4.3 Denovo result - Summary tab

In the summary tab, A PEAKS Studio de novo sequencing result can be filtered using Average Local Confidence (ALC) score. Low quality de novo sequences can be filtered out by specifying a minimal threshold of the ALC score. The purpose of filtering is to remove poor sequences in which residues are incorrectly identified. By default, the ALC threshold is set to 50%. After changing the threshold, click Apply to apply the new filter. The result in the Denovo result view will be updated accordingly.

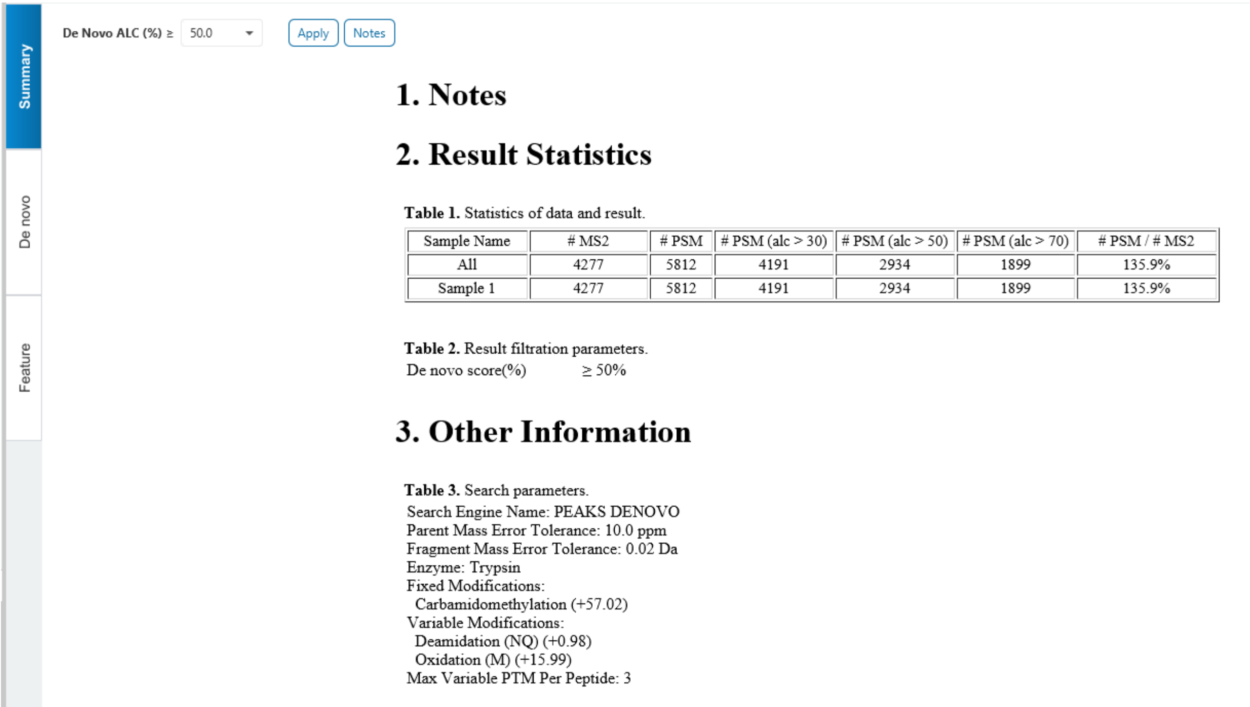

Sample Name	# MS2	# PSM	# PSM (alc > 30)	# PSM (alc > 50)	# PSM (alc > 70)	# PSM / # MS2
All	4277	5812	4191	2934	1899	135.9%
Sample 1	4277	5812	4191	2934	1899	135.9%

Click on Notes to open the Notes dialog to save useful information regarding this Analysis. The Notes will appear in Section 1 of the summary page at the top.

Section 2 Result Statistics gives key statistics in Table 1 on the number of MS2 scans and # PSMs total as well as with different ALC thresholds for each sample. The #PSM / #MS2 provides a de novo sequencing identification rate metric for each sample.

Table 2 simply shows what is the current Denovo ALC filter set. This is placed here so that when the Summary page is exported, the viewer will know what the ALC threshold was at the time.

Section 3 Other Information shows the parameters that were set when the analysis was ran.

4.4 De novo result - De novo View

The "De Novo View" displays the de novo sequencing results in greater detail, as shown in the next figure. The table on the top displays de novo sequences, while the bottom section provides additional information about the peptide-spectrum match.

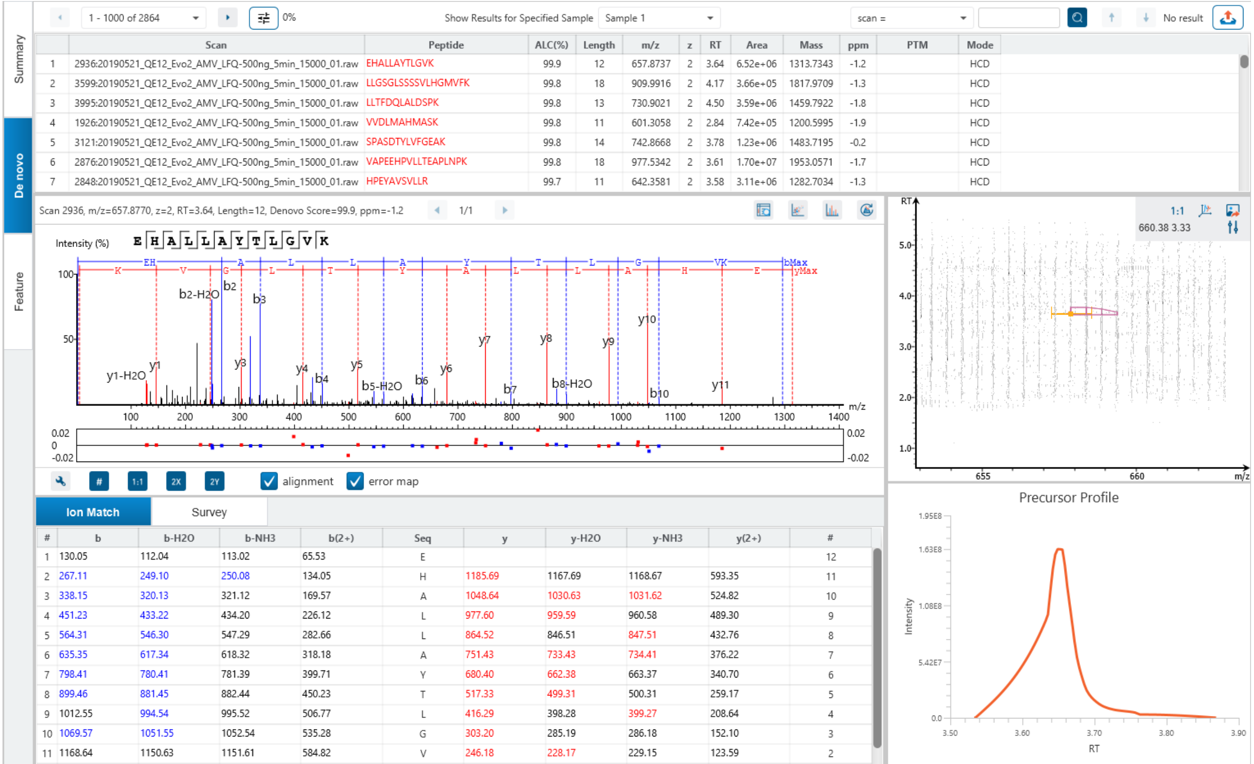

4.4.1 De novo View - De novo table

The following list describes the contents in each of the columns of the Denovo table:

- **Scan:** The scan number and data file it belongs to. The scan number is a unique index for tandem mass spectra in the data.
- **Peptide:** The amino acid sequence of the peptide as determined by de novo sequencing. A modified residue is followed by a pair of parentheses enclosing the mass of that modification.
- **ALC(%):** The average local confidence. ALC is calculated as the total of the residue local confidence scores in the peptide divided by the peptide length. The higher the score the more confident the denovo candidate.
- **Length:** The number of amino acids in the sequence backbone.
- **m/z:** The precursor mass-to-charge ratio.

- **z:** The precursor charge.
- **RT:** The retention time (elution time) of the spectrum as acquired by mass spectrometer.
- **Area:** The area under the curve of the peptide feature found at the same m/z and retention time as the MS/MS scan. This can be used as an indicator of the abundance of the peptide.
- **Mass:** The monoisotopic mass of the peptide.
- **ppm:** The precursor mass error, calculated as 10^6 × (precursor mass - peptide mass) / peptide mass.
- **PTM:** The types and the numbers of modifications present in the peptide shown in color-coded icons.
- **Mode:** The fragmentation mode in which de novo sequencing is performed by the algorithm.

Click on any of the column headers to sort the column.

To copy the sequence, right click on a row (or multiple rows) in the table and select “Copy selected peptide sequence(s)”. Another option is to mouse over any cell in the table and hitting Ctrl + C on your keyboard to copy the contents of those selected cells.

In the table, Amino acids in de novo sequences are color-coded according to their local confidence scores. Red represents a very high confidence (greater than 90%); purple represents a high confidence (80 to 90%); blue represents a medium confidence (60 to 80%); and black represents low confidence (less than 60%). Hover the cursor over the sequence to view local confidence scores of each individual amino acid.

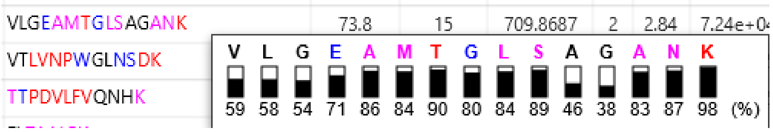

4.4.2 De novo View - De novo table controls

The menu above the Denovo table provides options to scroll through the table of de novo peptides, filter for mass tags, switch between samples, search for specific entries, and export the table.

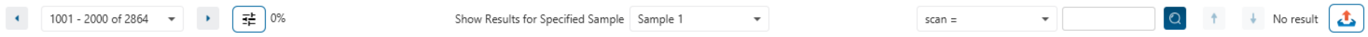

4.4.2.1 De novo table controls - Sample Selection

Result for different samples can be viewed by selecting the sample using the "Show Results for Specified Sample" drop-down menu.

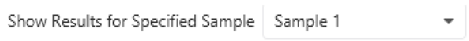

4.4.2.2 De novo table controls - Pagination

1000 rows are visible at any one time in the Denovo table. Use the top left Pagination options to switch between result records.

4.4.2.3 De novo table controls - Mass Tag Filter

Click on 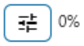

to use the mass tag confidence slider filter which will replace amino acids in the sequence with their mass tag values if their local confidence does not meet the threshold.

4.4.2.4 De novo table controls - Search function

Use the search function to search for scan # or precursor ID, m/z, RT, or sequence contains. Search results can be navigated between using the Up or Down arrows.

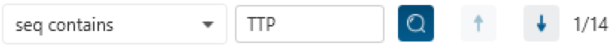

Options for searching include:

- **Scan =:** Search for the Scan number associated with each peptide. One scan can be associated to multiple peptides. For projects with multiple data files, the scan number is searched on every fraction. For timsTOF, there is the option to search for Precursor ID instead.
- **m/z ≈:** Search for the approximate m/z. The search will consider a range of m/z equal to the search value +/- 1 of the last significant digit.

  For example, searching for m/z ≈ 580.8 will find all m/z between [580.7, 580.9]
- **RT ≈:** Search for the approximate RT. Likewise, the search will consider a range of RT equal to the search value +/- 1 of the last significant digit set.

  For example, searching for RT ≈ 64 will find all m/z between [63, 65]
- **Seq contains:** Search for the sequence in the table. If specifying a sequence without glycans or PTMs, the results will include that sequence and any sequences with glycans or PTMs with the same backbone. If specifying a sequence with bracket and some mass value (like in the above image), then only sequence that have a substring with an exact match will be considered.

  Additionally, since the glycan peptide table shows both I/L and they can not be distinguished, then searching for I in the sequence is equivalent to searching for L in the sequence. i.e. Searching for “LVLK” is the same as searching for “LVIK”, “IVLK”, and “IVIK”

4.4.2.5 De novo table controls - Table exporting

Click on the button to export the contents of the current table. This considers sorting applied to the table and is consistent with the view. There is the option to export the De novo table and/or the De novo all candidates table in CSV format.

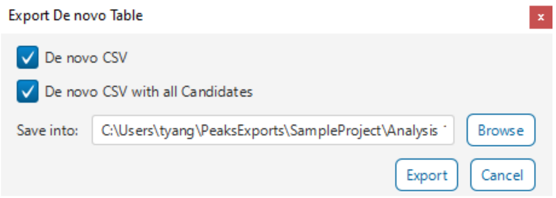

4.4.3 De novo View - De novo All Candidates

Click on the button to view the All de novo candidates for that spectrum. By default, the top 5 candidates are reported. The highest ALC scoring candidate is the one displayed in the De novo table.

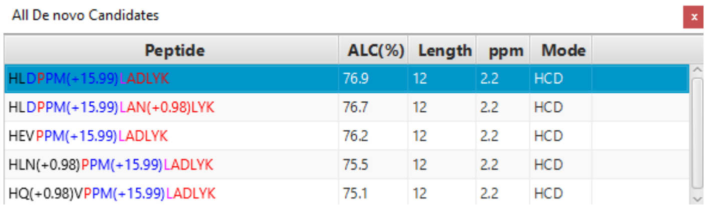
All De novo Candidates

Peptide	ALC(%)	Length	ppm	Mode
HLDPPM(+15.99)LADLYK	76.9	12	2.2	HCD
HLDPPM(+15.99)LAN(+0.98)LYK	76.7	12	2.2	HCD
HEVPPM(+15.99)LADLYK	76.2	12	2.2	HCD
HLN(+0.98)PPM(+15.99)LADLYK	75.5	12	2.2	HCD
HQ(+0.98)VPPM(+15.99)LADLYK	75.1	12	2.2	HCD

4.4.4 PSM selection

Click on the Left and Right arrow buttons to switch between PSMs related to the currently selected feature.

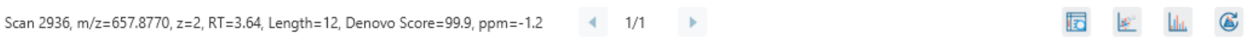

All other components in the De novo View interface will update according to the currently selected PSM.

For timsTOF instruments, Precursor ID is used rather than Scan number.

4.4.4.1 Show spectrum in Data View

Click on to open the Data Refine MS/MS tab (if not already opened) and jump to the corresponding selected Scan.

4.4.4.2 Show spectrum in LC/MS View

Click on to open the Data Refine LC/MS tab (if not already opened) and jump to the corresponding selected Scan, highlighting the De novo marker and the feature if present.

4.4.4.3 Show Raw Spectrum View

Click on to show the raw spectrum chart of the currently selected Scan.

4.4.5 Annotated Spectrum Chart

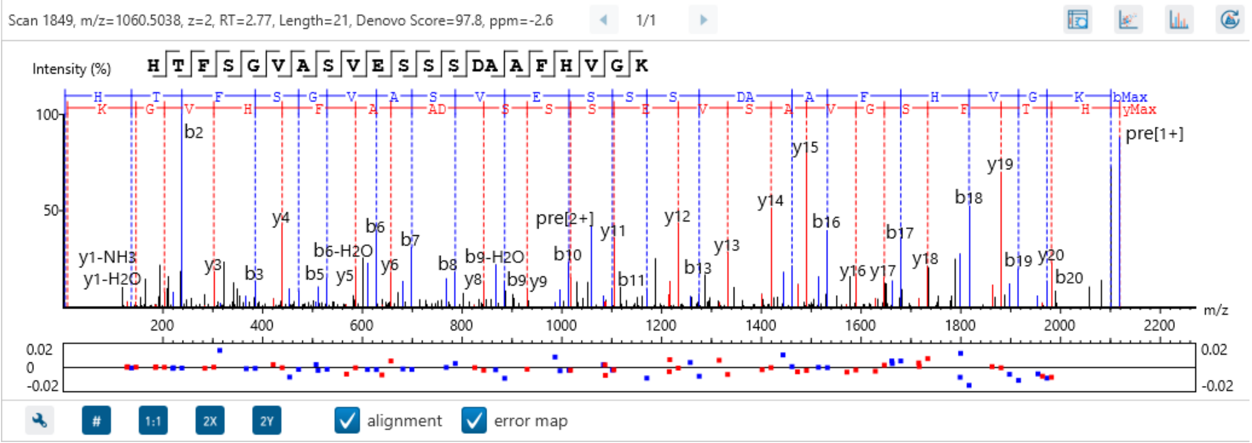

For the currently selected PSM, a graphical representation is shown in the form of an annotated spectrum chart. There are several controls provided for this view which will be explored in the following sections.

4.4.5.1 Annotated Spectrum Chart - Chart Navigation

Moving the cursor over the peptide sequence in the spectrum will show the mass transitions for a particular amino acid residue.

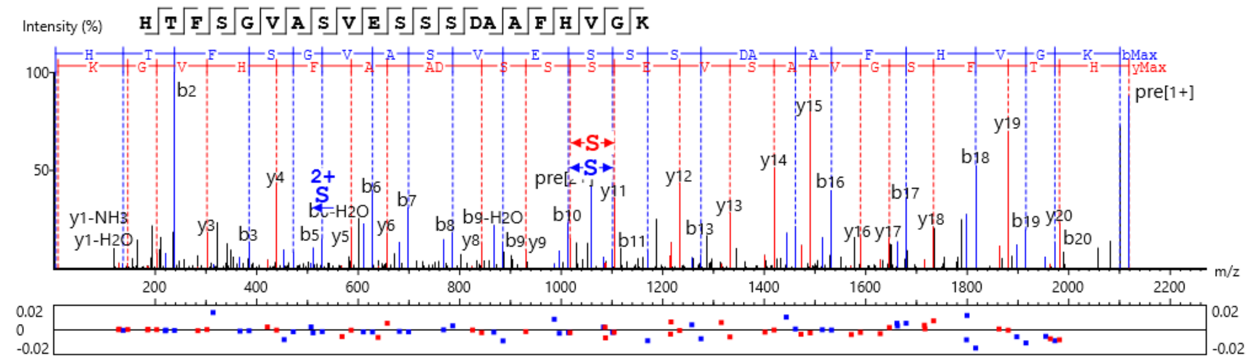

Zoom to a m/z region: Drag horizontally from the start m/z and to the end m/z with left mouse button.

Zoom in/out smoothly: Place the cursor at a particular m/z value (right below the x-axis), zoom in/out by scrolling the mouse wheel.

Increase/Decrease peak intensity: Place the cursor in the spectrum and scroll the mouse wheel.

See the whole spectrum: Double click in the spectrum or click the 1:1 button.

4.4.5.2 Annotated Spectrum Chart - Spectrum Annotation Settings

Click on the button to show the spectrum annotation settings.

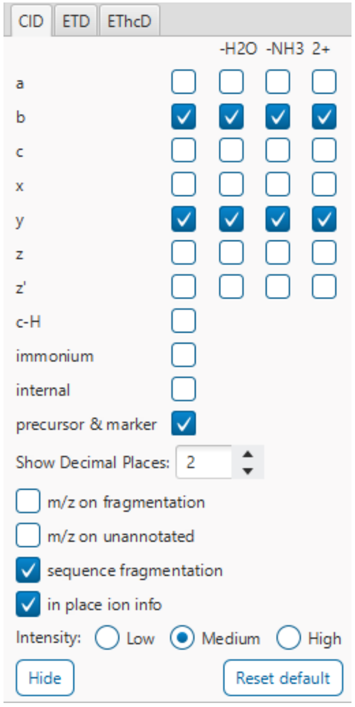

The spectra in the PEAKS results can be annotated by selecting ion types from the thorough collection of ions that PEAKS offers. The selected ion types will be displayed in the “Ion Match” table, as well. It is possible to annotate the spectrum with various ion types for CID, EThcD and ETD. Each of these fragmentation modes can be set with different settings which will be shared with other results with the same fragmentation type.

To reset the defaults, click on the “Reset default” button.

Show Decimal Places: Select the number of decimal places that will appear in the ion table and the spectrum view. The default is set to two decimal places.

m/z on Fragmentation: Select this to display the m/z value on top of the annotated ions.

m/z on Unannotated: Select this to display the m/z value on top of the peaks without ion matches.

Sequence Fragmentation: Select this to display the sequence fragmentation on the top-left corner of the “Spectrum Annotation” view.

In Place Ion Info: Ion information, m/z value, and relative intensity are all displayed in a pop-up within the “Spectrum Annotation” view when this option is checked and the cursor is placed on a peak.

Intensity: Set the intensity threshold for spectrum annotation to low (2%), medium (5%), or high (10%).

4.4.5.3 Annotated Spectrum Chart - Additional Chart Controls

Click on # or % to toggle between absolute and percentage intensity values in the Y axis.

Click on 1:1 to reset the zoom level of the annotated spectrum chart to the default.

Click on 2X to zoom into the X axis by a factor of 2.

Click on 2Y to zoom into the Y axis by a factor of 2.

The "alignment" check box ☑ alignment allows user to display/hide how the fragment ions generated from the peptide align with the spectrum. N-terminal ions are shown in blue and C-terminal ions are shown in red.

The “error map” check box ☑ error map is used to show/hide the error map underneath the chart.

4.4.6 Ion Match Table

The “Ion Match” tab in the bottom pane contains a table with possible fragment ions for the selected MS/MS scan. Each ion in the table shows the calculated mass, where if the fragment ion is present in the spectrum, its mass value is displayed in color. N-terminal ions are shown in blue and C-terminal ions are shown in red. A fragment ion is found when there is a matching peak within the mass error tolerance, as defined in the de novo sequencing parameters, and when relative intensity of the matching peak is at least 2%. The ion types displayed in the table can be configured in "Spectrum Annotation Settings" (see 4.4.5.2 Annotated Spectrum Chart - Spectrum Annotation Settings).

Glycan Ion Match | Ion Match | Survey

#	b	b-H2O	b-NH3	b(2+)	Seq	y	y-H2O	y-NH3	y(2+)	#
1	129.066	111.056	112.039	65.033	Q					21
2	257.125	239.114	240.098	129.062	Q	2387.065	2369.055	2370.039	1194.033	20
3	385.182	367.170	368.157	193.092	Q	2259.007	2240.996	2241.980	1130.004	19
4	522.241	504.232	505.214	261.621	H	2130.948	2112.938	2113.921	1065.988	18
5	635.326	617.317	618.299	318.163	L	1993.890	1975.879	1976.862	997.445	17
6	782.396	764.382	765.370	391.697	F	1880.805	1862.795	1863.778	940.903	16
7	839.416	821.395	822.389	420.208	G	1733.737	1715.726	1716.710	867.369	15
8	926.448	908.455	909.421	463.724	S	1676.716	1658.705	1659.689	838.858	14
9	1040.491	1022.481	1023.464	520.746	N(+1622.58)	1589.683	1571.673	1572.656	795.342	13
10	1139.560	1121.549	1122.516	570.280	V	1475.644	1457.630	1458.614	738.320	12
[illegible]	[illegible]	[illegible]	[illegible]	[illegible]	[illegible]	[illegible]	[illegible]	[illegible]	[illegible]	[illegible]

Note: Uncheck the alignment checkbox in the spectrum annotation view to see the highlighted fragment ions more easily.

4.4.7 Survey Chart

The “Survey” tab displays the precursor ion spectrum. The buttons that appear in this section are identical to those explained above in 4.4.5.3 Annotated Spectrum Chart - Additional Chart Controls.

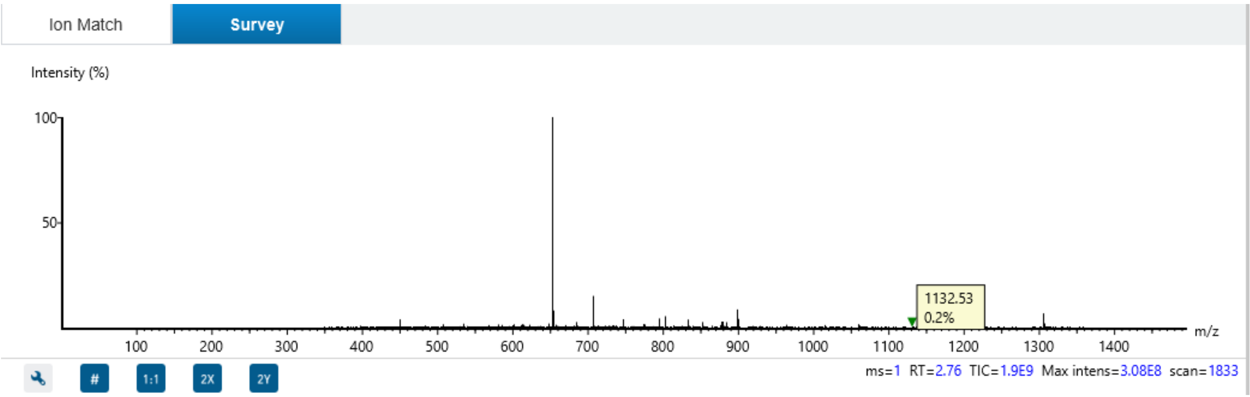

4.4.8 LC/MS Snapshot

The LC-MS Snapshot displays a zoomed-in view of the precursor scan in the LC-MS heat map 2-D view. The span of the orange line in the LC-MS Snapshot designates the isolation window.

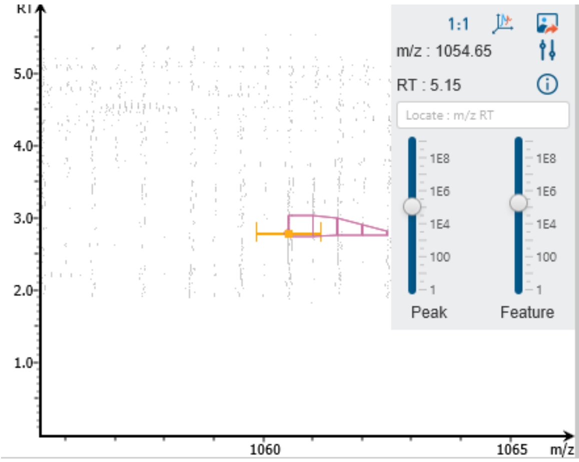

Users can change the view from the 2-D heat map to a 3-D intensity view by clicking the intensity view button in the upper right-hand corner of the LC-MS Snapshot. In addition, users can export the LC-MS heat map by clicking the export image button in the upper right-hand corner of the LC-MS Snapshot.

Expand the options dropdown by clicking to reveal a search option to specify a *m/z RT* (ensure there is a space between these two numbers) location to zoom into. There is also a Peak slider filter to filter out low intensity peaks and a feature area filter to filter out low area features.

4.4.9 Precursor Profile

The Precursor Profile, or eXtracted Ion Chromatogram (XIC) of the precursor ion, graphs the elution profile of the selected peptide feature vector over the retention time range where it was identified. The graph is plotted with the retention time along the x-axis and the intensity along the y-axis.

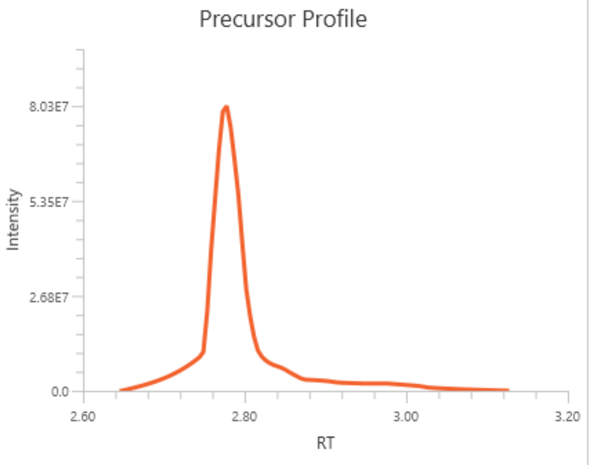

4.5. De novo result - Feature View

The **"Feature View"** displays all the feature information in greater detail, as shown in the next figure. The table on the top displays features, while the bottom section provides additional information about the feature, precursor profile, and if available peptide-spectrum match.

4.5.1 Feature View - Feature table

The following list describes the contents in each of the columns of the Feature table in De novo result:

- **Fraction:** The fraction from which the feature is detected.
- **Feature ID:** An arbitrary number provided by PEAKS during data refinement for each identifiable feature.
- **m/z:** The precursor mass-to-charge ratio.
- **z:** The precursor charge.
- **RT:** The retention time (elution time) for the spectrum as recorded in the data.
- **RT Begin:** The beginning retention time (elution time) for the feature as recorded in the data.
- **RT End:** The ending retention time (elution time) for the feature as recorded in the data.
- **Area:** The area under the curve of the peptide feature found at the same m/z and retention time as the MS/MS scan. This can be used as an indicator of the abundance of the peptide.
- **Denovo Peptide:** The amino acid sequence of the peptide associated with the feature, as determined by de novo sequencing. A modified residue is followed by a pair of parentheses enclosing the mass of that modification. If multiple MS2 are associated with the feature, all de novo peptide results are listed.
- **Denovo Score (%):** The denovo score represents the quality of the peptide spectrum match. Factors that affect this score includes ALC score and RT difference.
- **ppm:** the mass error in ppm of the de novo peptide with the highest ALC score

Click on any of the column headers to sort the column.

To copy the sequence, right click on a row (or multiple rows) in the table and select "Copy selected peptide sequence(s)". Another option is to hover mouse over any cell in the table and hitting Ctrl + C on your keyboard to copy the contents of those selected cells.

In the table, Amino acids in Denovo Peptide are color-coded according to their local confidence scores. Red represents a very high confidence (greater than 90%); purple represents a high confidence (80 to 90%); blue represents a medium confidence (60 to 80%); and black represents low confidence (less than 60%). Hover the cursor over the sequence to view local confidence scores of each individual amino acid.

4.5.2 Feature View - Feature table controls

The menu above the Denovo table provides options to scroll through the table of de novo peptides, filter for mass tags, switch between samples, search for specific entries, and export the table.

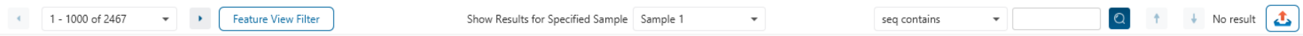

4.5.2.1 Feature table controls - Sample Selection

Result for different samples can be viewed by selecting the sample using the "Show Results for Specified Sample" drop-down menu.

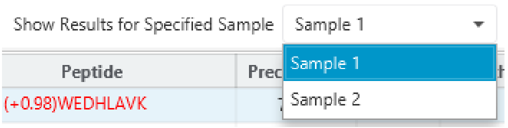

4.5.2.2 Feature table controls - Pagination

1000 rows are visible at any one time in the Denovo table. Use the top left Pagination options to switch between result records.

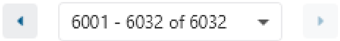

4.5.2.3 Feature table controls - Feature View Filter

Click on 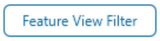

to open the Feature View Filter dialog.

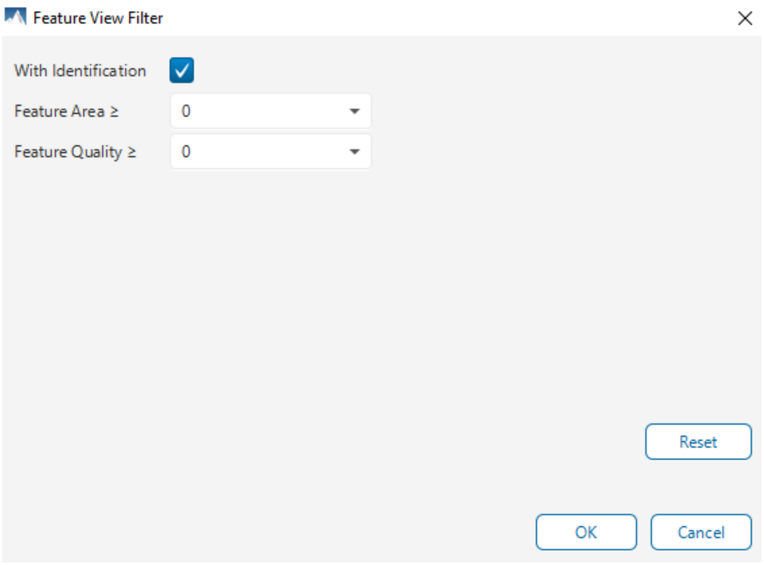

There are three options for filter provided:

- With Identification: When checked, features that are associated with a De novo peptide will be displayed in the table. Uncheck this to see all features detected in the samples.
- Feature Area ≥: Filters out all features with area below this threshold.
- Feature Quality ≥: Filters out all features with quality below this threshold. In PEAKS Studio 11, all features in a certain dataset may have quality score of 0 as there is no scoring associated with the features. It is recommended to not use this filter in that case.

Click on the Reset button to restore the default filters.

Click on OK to apply these filters to the Feature table.

Click on Cancel to discard changes and close the Feature View Filter dialog.

4.5.2.4 Feature table controls - Search function

Use the search function to search for sequence or m/z. Search results can be navigated between using the Up or Down arrows.

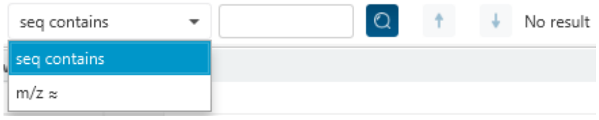

Options for searching include:

- m/z ≈: Search for the approximate m/z. The search will consider a range of m/z equal to the search value +/- 1 of the last significant digit.

  For example, searching for m/z ≈ 580.8 will find all m/z between [580.7, 580.9]

- Seq contains: Search for the sequence in the table. If specifying a sequence without glycans or PTMs, the results will include the bare sequence as well as sequences containing glycans or PTMs with the same peptide backbone. If specifying a sequence and a mass value within a bracket, then only sequence that has a substring with an exact match will be considered.

  Additionally, since the glycan peptide table shows both I/L and they can not be distinguished, then searching for I in the sequence is equivalent to searching for L in the sequence. i.e. Searching for “LVLK” is the same as searching for “LVIK”, “IVLK”, and “IVIK”

4.4.2.5 Feature table controls - Table exporting

Click on the button to export the contents of the current table. This considers sorting applied to the table and is consistent with the view. There is the option to export the De novo table and/or the De novo all candidates table in CSV format.

4.4.2.6 Feature table - PSM visualization

The rest of the components of the Feature View are the same as the De novo View Chapter.

Those sections are:

- 4.4.4 PSM selection

- 4.4.5 Annotated Spectrum Chart
- 4.4.6 Ion Match Table
- 4.4.7 Survey Chart
- 4.4.8 LC/MS Snapshot
- 4.4.9 Precursor Profile

4.6. De novo result - Exporting

In Project View, double-click on Export to open the Export node.

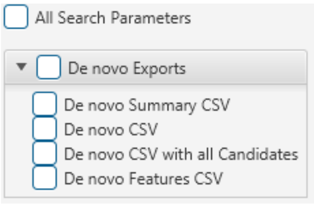

There are several options for exporting the De novo results:

- De novo Summary CSV: Exports the statistics tables from the De novo Summary page into a CSV format.
- De novo CSV: Exports the De novo table. This export does not consider any sorting and will export a separate file for each sample.
- De novo CSV with all Candidates: Exports the De novo all candidates. This export does not consider any sorting and will export a separate file for each sample.
- De novo Features CSV: This export does not consider any Feature view filters or sorting, but will consider the De Novo ALC (%) filter in the Summary tab. Each sample will be in a separate export.

5. Deepnovo Peptidome

5.1 Deepnovo Peptidome Analysis Workflow and Parameter Settings

To run a Deepnovo Peptidome Analysis, Select DeepNovo Peptidome in Workflow Selection

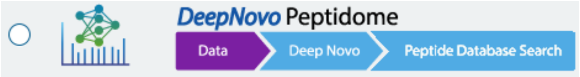

After deciding the Data Refine option, proceed to the next step to see the Deepnovo Peptidome Search parameters.

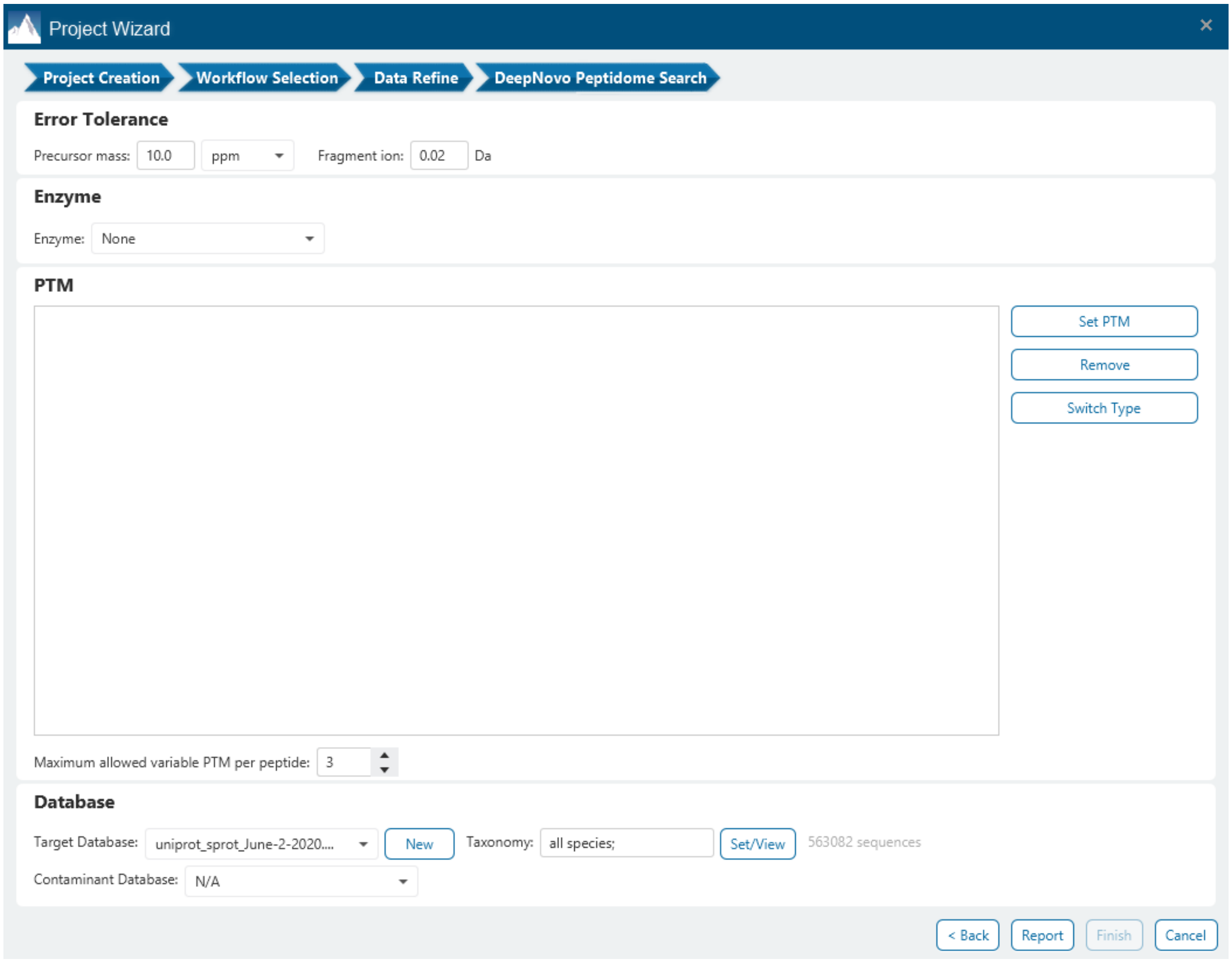

The following parameter settings are provided:

Precursor Mass Error Tolerance: The maximum mass difference between the peptide and the monoisotopic mass of the precursor ion.

Precursor Mass Tolerance unit: PEAKS generates peptides within the precursor mass error tolerance. Precursor mass error tolerance can be specified in either Daltons or ppm.

Fragment Mass Error Tolerance: PEAKS uses this value when scoring peptide sequences. PEAKS Studio considers a fragment ion to be matched if the calculated m/z is within the specified error tolerance. Units are Daltons.

Both precursor and fragment error tolerance parameters need to be consistent with the mass accuracy of the mass analyzers used for MS and MSMS acquisitions respectively.

Enzyme Settings: Select the enzyme used for protein digestion from the enzyme drop-down menu. For Deepnovo Peptide Search, only None and Trypsin are provided as Enzyme options.

Note: "None" is a special enzyme allowing non-specific cleavage at both ends of the peptide. It is the recommended option when no digestion enzyme was used or when the digestion enzyme exhibits a high degree of non-specificity.

PTM Settings: Click “Set PTM” to open the **PTM Options** dialog and specify the fixed PTMs and a few common variable PTMs expected in the experiment. Highlight PTMs and click on the 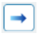 button to place them as either Fixed or Variable PTM. From there, highlighted PTMs can be removed by clicking the “Remove” button. Clicking “Remove All” will reset the PTM options. Highlighting and clicking “Switch Type” will move the Fixed PTM in into the Variable PTM list and vice-verse. Click on “New” to create a new custom PTM. While a PTM is selected, click on “View” to see more details.

Note: Multiple PTMs can be highlighted by holding down the CTRL key or SHIFT key on the keyboard.

Note: For a specific residue, only one fixed modification is allowed.

Once the PTMs are selected, the maximum number of variable PTMs per peptide can be specified and a value of less than 4 is recommended.

Note: The use of variable modifications increases the size of the computational search space for the de novo sequencing algorithm. It is recommended not to select too many variable modifications in PEAKS de novo. See PEAKS PTM for alternate search options.

Target Database: Select a protein sequence database for the search. Select one from the list of databases that have been configured in PEAKS and set the taxonomy by clicking on Set/View, if applicable. To configure a new database, click on New next to the Target Database dropdown to open the Database Configuration dialog.

Contaminant Database: To search for contaminants, select a contaminant database from the list of databases that have been configured in PEAKS. Keep the selection as N/A if no contaminant database is required for the search.

Click on “Report” to proceed to the final step. Report filters can be set here to filter the final result.

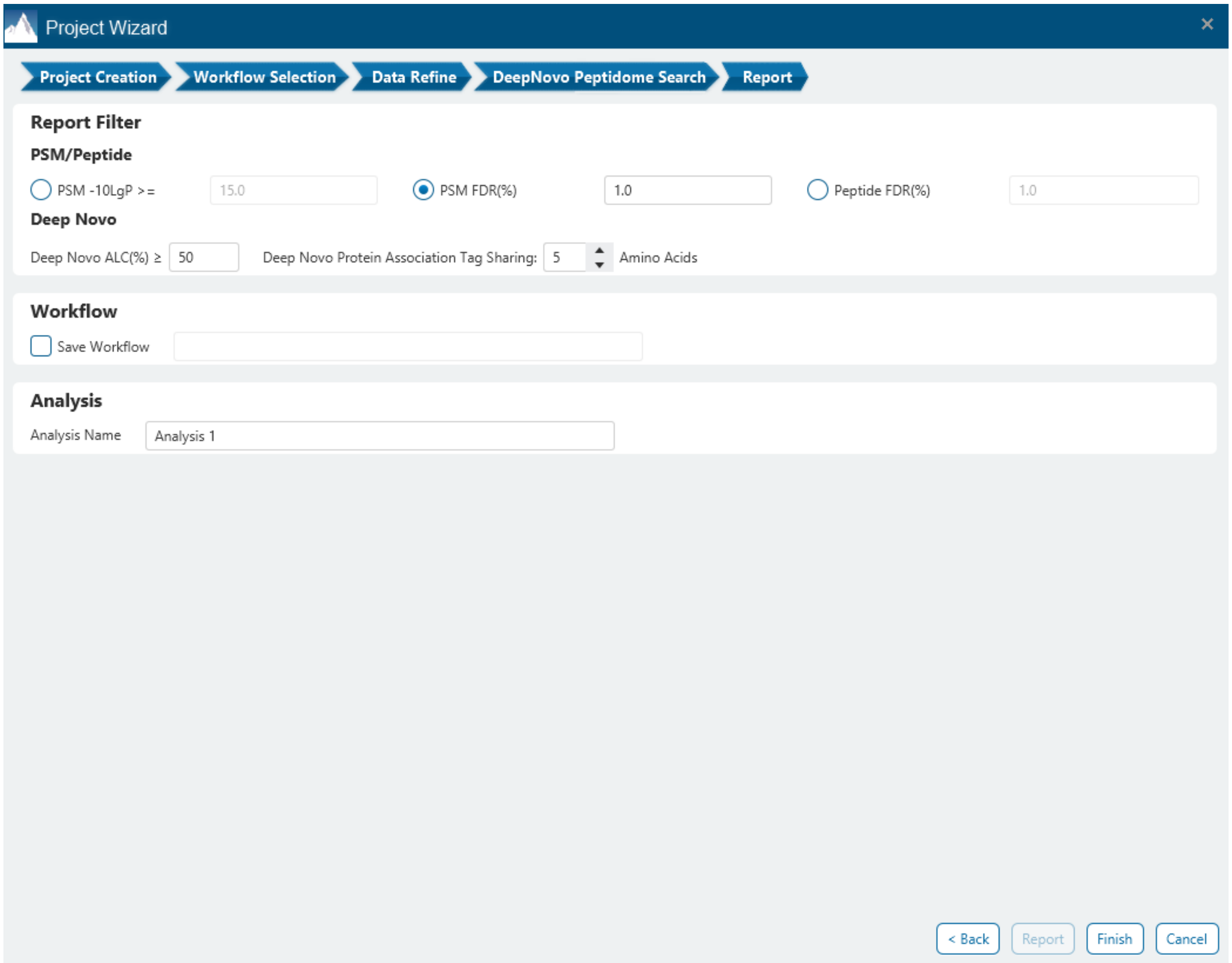

These filters are provided:

PSM/Peptide: Select one of the options to filter the PSMs or Peptides.

Deep Novo: Set a Deep Novo ALC(%) filter to filter the Deep novo peptides. This number can be adjusted in the Deep novo Summary tab to filter the Deep novo table.

Finally, there is an option to specify the Analysis Name (which can also be renamed post-analysis) and/or an option to save the Workflow for a timely re-use in future analyses.

5.2 Understanding PEAKS Deep Novo Results

When Deep Novo sequencing is done, double-click on the Deep Novo result node to open the the Deep novo result. The result contains two tabs:

- Summary: This tab shows an outline of the PEAKS deep novo search results with key statistics and chart visualizations. The overall quality of the experiment can be examined and the filters for deep novo ALC can be adjusted at the top of the page.

- Deep Novo: This tab shows a list of peptide sequences detected by deep novo sequencing, which passed the set ALC score threshold.

The following sections will explore each of these two tabs in the Deep Novo result.

5.3 Deep Novo result - Summary tab

In the summary tab, PEAKS Studio deep novo sequencing result can be filtered using Average Local Confidence (ALC) score. Low quality deep novo sequences can be filtered out by specifying a minimal threshold of the ALC score. The purpose of filtering is to remove low confidence sequences in which residues are likely incorrectly identified. By default, the ALC threshold is set to 50%. When changing this threshold, click Apply to apply the new filter. The result in the Denovo result view will be updated accordingly.

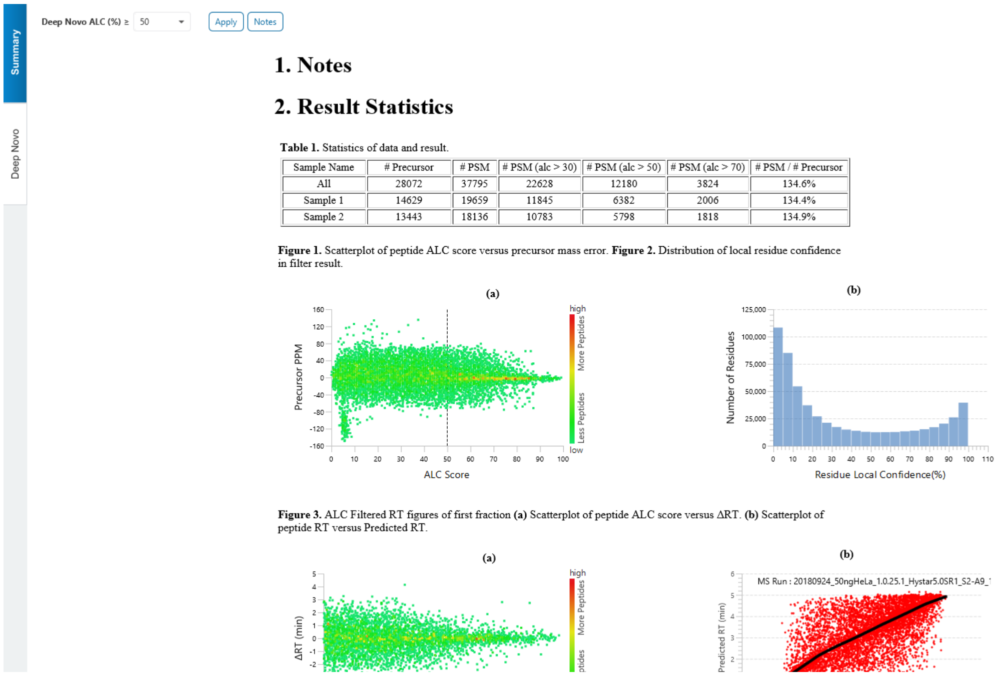

1. Notes

2. Result Statistics

Table 1. Statistics of data and result.

Sample Name	# Precursor	# PSM	# PSM (alc > 30)	# PSM (alc > 50)	# PSM (alc > 70)	# PSM / # Precursor
All	28072	37795	22628	12180	3824	134.6%
Sample 1	14629	19659	11845	6382	2006	134.4%
Sample 2	13443	18136	10783	5798	1818	134.9%

Figure 1. Scatterplot of peptide ALC score versus precursor mass error. **Figure 2.** Distribution of local residue confidence in filter result.

Figure 3. ALC Filtered RT figures of first fraction **(a)** Scatterplot of peptide ALC score versus ΔRT. **(b)** Scatterplot of peptide RT versus Predicted RT.

Click on Notes to open the Notes dialog to save useful information regarding this Analysis. The Notes will appear in Section 1 of the summary page at the top.

Section 2 Result Statistics gives key statistics in Table 1 by providing the total number of MS2 scans and PSMs total as well as the number of MS2 scans and PSMs with different ALC thresholds for each sample. The #PSM / #MS2 provides a de novo sequencing identification rate metric for each sample. For timsTOF data, Table 1 shows # Precursors instead.

5.3.1 Deep Novo result - Summary tab - ALC versus precursor mass error scatterplot

Figure 1. a) Scatterplot of peptide ALC score versus precursor mass error shows the distribution of precursor mass error according to their ALC score. Red indicates a higher concentration of peptides at that mass error and ALC score, and green indicates a lower concentration. A vertical dashed line shows the current Deep Novo ALC(%) filter applied.

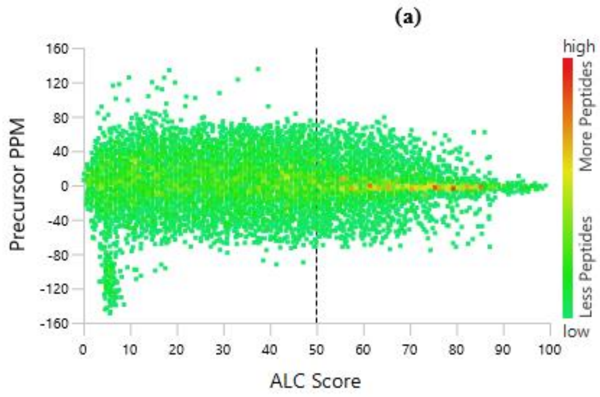

5.3.2 Deep Novo result - Summary tab - Distribution of local residue confidence

Figure 1. b) Distribution of local residue confidence shows the overall distribution of local confidence scores of residues across all peptides.

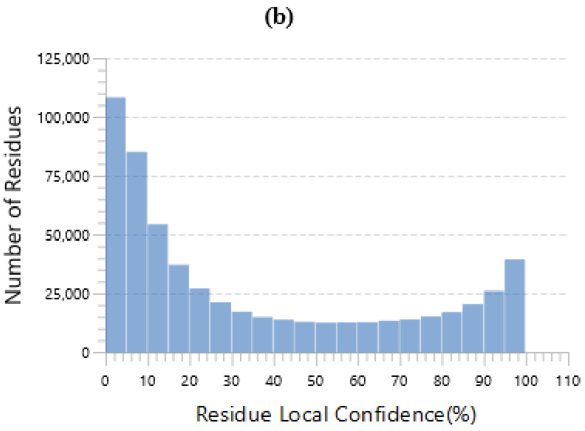

5.3.3 Deep Novo result - Summary tab - ALC RT distribution of first fraction

Similar to Figure 1, Figure 2. a) ALC Filtered RT figures of first fraction shows the distribution of ΔRT (min) according to their ALC score. Red indicates a higher concentration of peptides at that ΔRT and ALC score, and green indicates a lower concentration. A vertical dashed line shows the current Deep Novo ALC(%) filter applied.

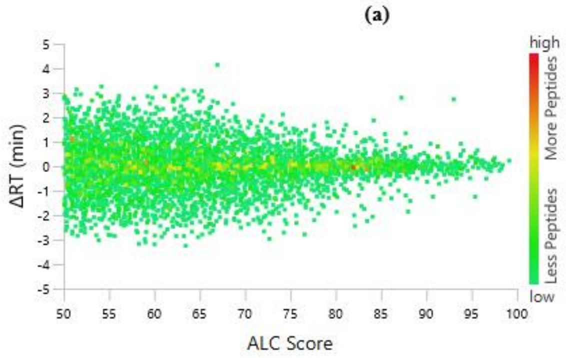

5.3.4 Deep Novo result - Summary tab - Peptide RT versus Predicted RT scatter plot

Figure 2. b) Scatterplot of peptide RT versus Predicted RT figure shows the scatter plot of predicted RT versus actual RT in minutes for the first fraction. A regression function is shown to fit the plot.

(b)

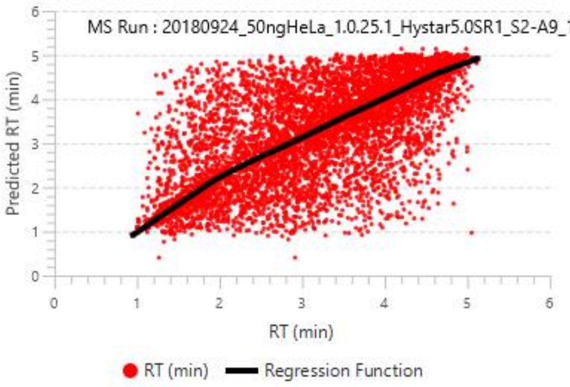

Table 2 simply shows what is the current Deep novo ALC filter set. This is placed here so that when the Summary page is exported, the viewer will know what the ALC threshold was at the time.

Section 3 Other Information shows the parameters that were set when the analysis was ran.

5.4 Deep Novo result - Deep Novo View

The "Deep Novo View" displays the deep novo sequencing results in greater detail, as shown in the next figure. The table on the top displays deep novo sequences, while the bottom section provides additional information about the peptide-spectrum match.

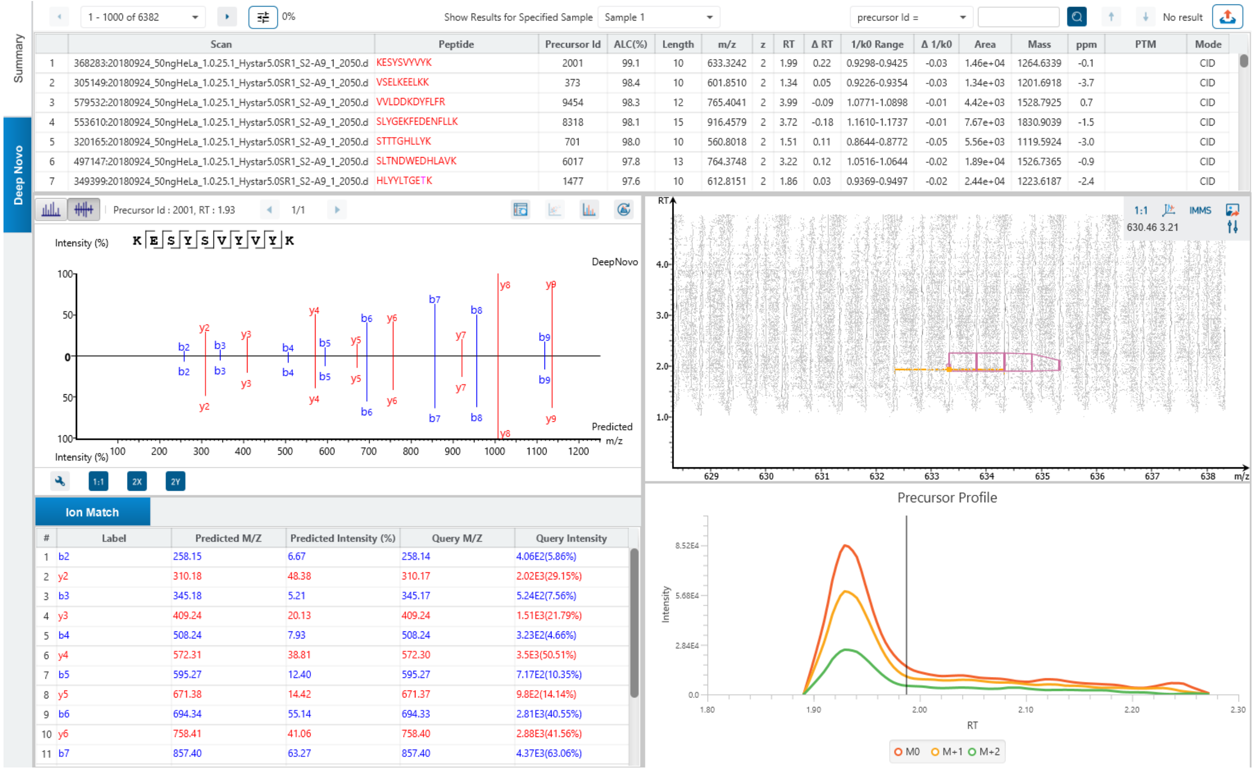

5.4.1 Deep Novo View - Deep Novo table

The following list describes the contents in each of the columns of the Denovo table:

- **Scan:** The scan number and data file it belongs to. The scan number is a unique index for tandem mass spectra in the data.
- **Peptide:** The amino acid sequence of the peptide as determined by de novo sequencing. A modified residue is followed by a pair of parentheses enclosing the mass of that modification.
- **ALC(%):** The average local confidence. ALC is calculated as the total of the residue local confidence scores in the peptide divided by the peptide length. The higher the score the more confident the denovo candidate.
- **Precursor Id:** This column is only displayed for timsTOF data. A unique identifier is assigned for each precursor obtained from the raw data.
- **Length:** The number of amino acids in the sequence backbone.
- **m/z:** The precursor mass-to-charge ratio.
- **z:** The precursor charge.
- **RT:** The retention time (elution time) of the spectrum as recorded in the data.
- **Δ RT:** The difference in retention time from the data and the RT prediction in deep learning algorithm.
- **1/k0 Range:** This shows the ion mobility range associated with precursor.
- **Δ 1/k0:** The difference in 1/k0 from the data and the 1/k0 prediction in deep learning algorithm.
- **Area:** The area under the curve of the peptide feature found at the same m/z and retention time as the MS/MS scan. This can be used as an indicator of the abundance of the peptide.
- **Mass:** The monoisotopic mass of the peptide.
- **ppm:** The precursor mass error, calculated as $10^6 \times$ (precursor mass - peptide mass) / peptide mass.
- **PTM:** The types and the numbers of modifications present in the peptide shown in color-coded icons.
- **Mode:** The fragmentation mode in which de novo sequencing is performed by the algorithm.

Click on any of the column headers to sort the column.

To copy the sequence, right click on a row (or multiple rows) in the table and select "Copy selected peptide sequence(s)". Another option is to mouse over any cell in the table and hitting Ctrl + C on your keyboard to copy the contents of those selected cells.

In the table, Amino acids in deep novo sequences are color-coded according to their local confidence scores. Red represents a very high confidence (greater than 90%); purple represents a high confidence (80 to 90%); blue represents a medium confidence (60 to 80%); and black represents low confidence (less than 60%). Hover the cursor over the sequence to view local confidence scores of each individual amino acid.

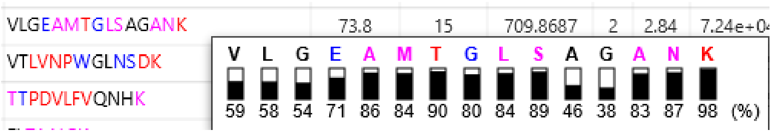

5.4.2 Deep Novo View - Deep Novo table controls

The menu above the Deep Novo table provides options to scroll through the table of deep novo peptides, filter for mass tags, switch between samples, search for specific entries, and export the table.

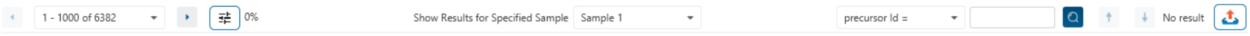

5.4.2.1 Deep Novo table controls - Sample Selection

Result for different samples can be viewed by selecting the sample using the "Show Results for Specified Sample" drop-down menu.

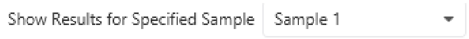

5.4.2.2 Deep Novo table controls - Pagination

1000 rows are visible at any one time in the Deep Novo table. Use the top left Pagination options to switch between result records.

5.4.2.3 Deep Novo table controls - Mass Tag Filter

Click on 0% to use the mass tag confidence slider filter which will replace amino acids in the sequence with their mass tag values if their local confidence does not meet the threshold.

5.4.2.4 Deep Novo table controls - Search function

Use the search function to search for scan # or precursor ID, m/z, RT, or sequence contains. Search results can be navigated between using the Up or Down arrows.

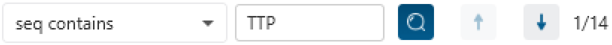

Options for searching include:

- **Scan =:** Search for the Scan number associated with each peptide. One scan can be associated to multiple peptides. For projects with multiple data files, the scan number is searched on every fraction. For timsTOF, there is the option to search for Precursor ID instead.
- **m/z ≈:** Search for the approximate m/z. The search will consider a range of m/z equal to the search value +/- 1 of the last significant digit.

  For example, searching for m/z ≈ 580.8 will find all m/z between [580.7, 580.9]
- **RT ≈:** Search for the approximate RT. Likewise, the search will consider a range of RT equal to the search value +/- 1 of the last significant digit set.

  For example, searching for RT ≈ 64 will find all m/z between [63, 65]
- **Seq contains:** Search for the sequence in the table. If specifying a sequence without glycans or PTMs, the results will include that sequence and any sequences with glycans or PTMs with the same backbone. If specifying a sequence with bracket and some mass value (like in the above image), then only sequence that have a substring with an exact match will be considered.

  Additionally, since the glycan peptide table shows both I/L and they can not be distinguished, then searching for I in the sequence is equivalent to searching for L in the sequence. i.e. Searching for “LVLK” is the same as searching for “LVIK”, “IVLK”, and “IVIK”

5.4.2.5 Deep Novo table controls - Table exporting

Click on the button to export the contents of the current table. This considers sorting applied to the table and is consistent with the view. There is the option to export the Deep Novo table and/or the Deep novo all candidates table in CSV format.

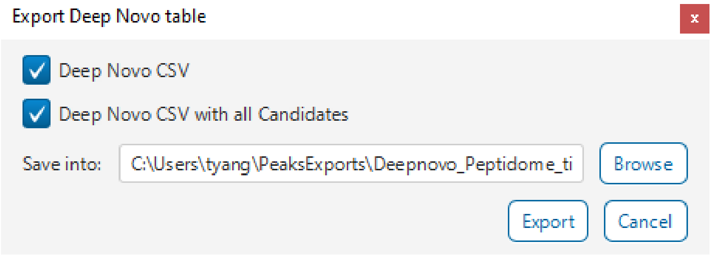

5.4.3 Deep Novo View - Deep Novo All Candidates

Click on the button to view the All deep novo candidates for that spectrum. By default, the top 5 candidates are reported. The highest ALC scoring candidate is the one displayed in the De novo table.

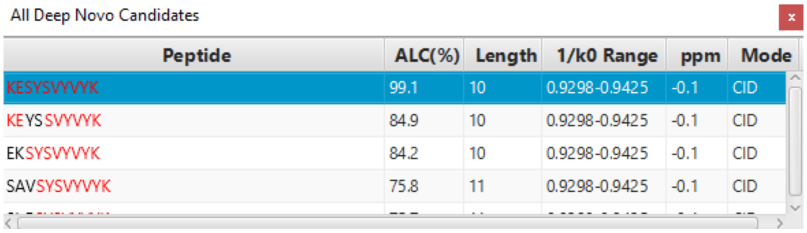

5.4.4 PSM selection

Click on the Left and Right arrow buttons to switch between PSMs related to the currently selected feature.

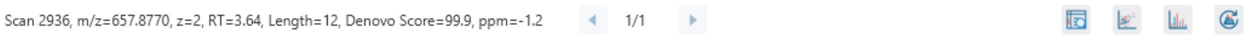

All other components in the De novo View interface will update according to the currently selected PSM.

For timsTOF instruments, Precursor ID is used rather than Scan number.

5.4.4.1 Show spectrum in Data View

Click on to open the Data Refine MS/MS tab (if not already opened) and jump to the corresponding selected Scan.

5.4.4.2 Show spectrum in LC/MS View

Click on to open the Data Refine LC/MS tab (if not already opened) and jump to the corresponding selected Scan, highlighting the De novo marker and the feature if present.

5.4.4.3 Show Raw Spectrum View

Click on to show the raw spectrum chart of the currently selected Scan.

5.4.5 Annotated Spectrum Chart

Click on button to switch from the Mirror plot view and annotated spectrum chart view.

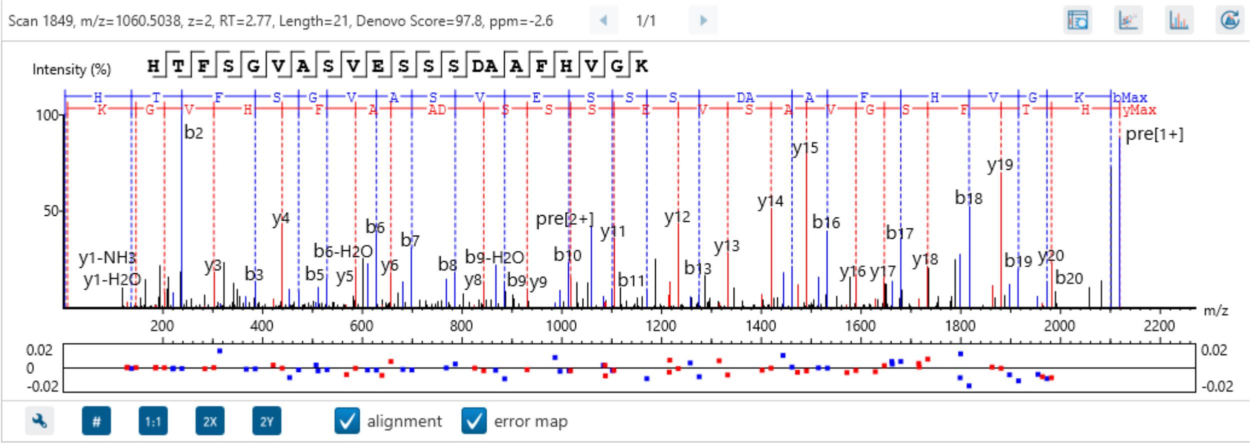

For the currently selected PSM, a graphical representation is shown in the form of an annotated spectrum chart. There are several controls provided for this view which will be explored in the following sections.

5.4.5.1 Annotated Spectrum Chart - Chart Navigation

Moving the cursor over the peptide sequence in the spectrum will show the mass transitions for a particular amino acid residue.

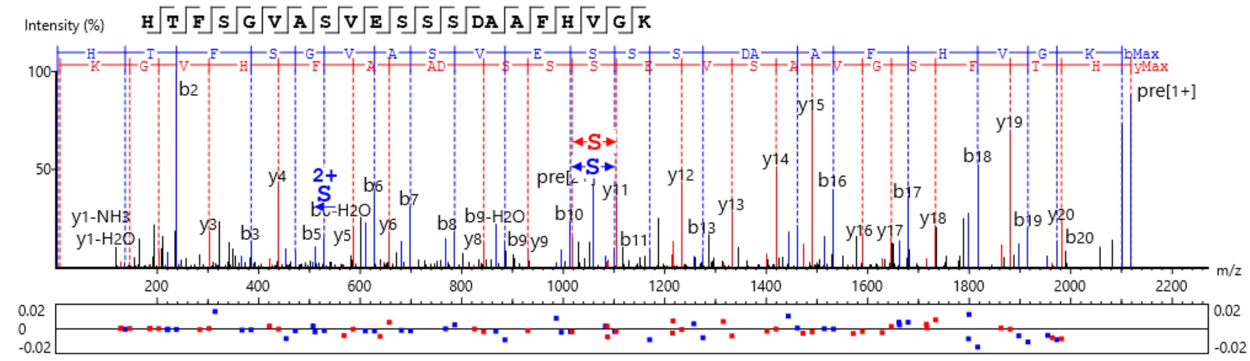

Zoom to a m/z region: Drag horizontally from the start m/z and to the end m/z with left mouse button.

Zoom in/out smoothly: Place the cursor at a particular m/z value (right below the x-axis), zoom in/out by scrolling the mouse wheel.

Increase/Decrease peak intensity: Place the cursor in the spectrum and scroll the mouse wheel.

See the whole spectrum: Double click in the spectrum or click the 1:1 button.

5.4.5.2 Annotated Spectrum Chart - Spectrum Annotation Settings

Click on the button to show the spectrum annotation settings.

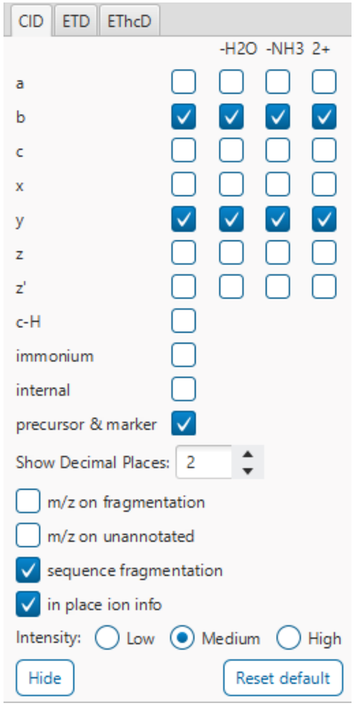

The spectra in the PEAKS results can be annotated by selecting ion types from the thorough collection of ions that PEAKS offers. The selected ion types will be displayed in the "Ion Match" table, as well. It is possible to annotate the spectrum with various ion types for CID, EThcD and ETD. Each of these fragmentation modes can be set with different settings which will be shared with other results with the same fragmentation type.

To reset the defaults, click on the "Reset default" button.

Show Decimal Places: Select the number of decimal places that will appear in the ion table and the spectrum view. The default is set to two decimal places.

m/z on Fragmentation: Select this to display the m/z value on top of the annotated ions.

m/z on Unannotated: Select this to display the m/z value on top of the peaks without ion matches.

Sequence Fragmentation: Select this to display the sequence fragmentation on the top-left corner of the "Spectrum Annotation" view.

In Place Ion Info: Ion information, m/z value, and relative intensity are all displayed in a pop-up within the "Spectrum Annotation" view when this option is checked and the cursor is placed on a peak.

Intensity: Set the intensity threshold for spectrum annotation to low (2%), medium (5%), or high (10%).

5.4.5.3 Annotated Spectrum Chart - Additional Chart Controls

Click on 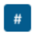 or 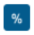 to toggle between absolute and percentage intensity values in the Y axis.

Click on 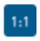 to reset the zoom level of the annotated spectrum chart to the default.

Click on 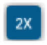 to zoom into the X axis by a factor of 2.

Click on 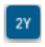 to zoom into the Y axis by a factor of 2.

The "alignment" check box ☑ alignment allows user to display/hide how the fragment ions generated from the peptide align with the spectrum. N-terminal ions are shown in blue and C-terminal ions are shown in red.

The “error map” check box ☑ error map is used to show/hide the error map underneath the chart.

5.4.5.4 Annotated Spectrum Chart - Mirror Plot

Deep Novo results have a Mirror Plot view. Click on the button to switch to the Mirror Plot.

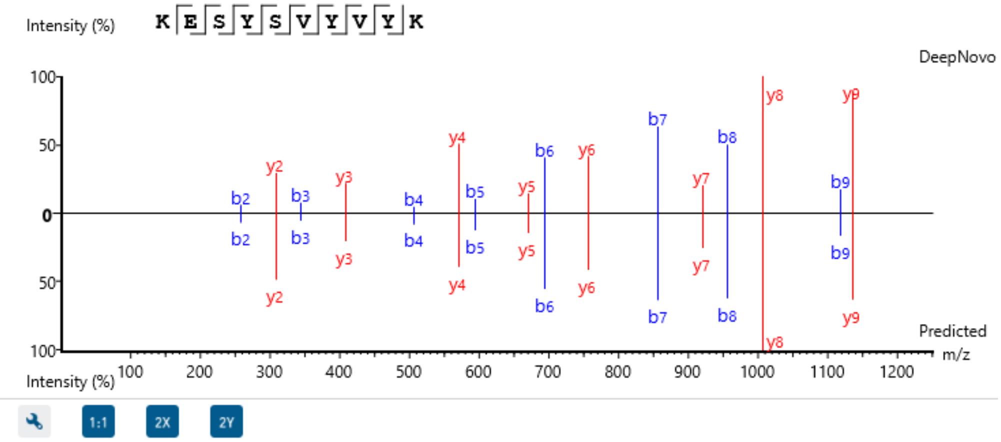

This view shows the ions from the query spectrum compared to the predicted spectrum.

5.4.6 Ion Match Table

The “Ion Match” tab in the bottom pane contains a table with possible fragment ions for the selected MS/MS scan. Each ion in the table shows the calculated fragment mass, where mass values displayed in color indicate fragment ions present in the spectrum. N-terminal ions are shown in blue and C-terminal ions are shown in red. A fragment ion is found when there is a matching peak within the mass error tolerance, as defined in the de novo sequencing parameters, and when relative intensity of the matching peak is at least 2%. The ion types displayed in the table can be configured in "Spectrum Annotation Settings" (see 4.4.5.2 Annotated Spectrum Chart - Spectrum Annotation Settings).

Glycan Ion Match | **Ion Match** | Survey

#	b	b-H2O	b-NH3	b(2+)	Seq	y	y-H2O	y-NH3	y(2+)	#
1	129.066	111.056	112.039	65.033	Q					21
2	257.125	239.114	240.098	129.062	Q	2387.065	2369.055	2370.039	1194.033	20
3	385.182	367.170	368.157	193.092	Q	2259.007	2240.996	2241.980	1130.004	19
4	522.241	504.232	505.214	261.621	H	2130.948	2112.938	2113.921	1065.988	18
5	635.326	617.317	618.299	318.163	L	1993.890	1975.879	1976.862	997.445	17
6	782.396	764.382	765.370	391.697	F	1880.805	1862.795	1863.778	940.903	16
7	839.416	821.395	822.389	420.208	G	1733.737	1715.726	1716.710	867.369	15
8	926.448	908.455	909.421	463.724	S	1676.716	1658.705	1659.689	838.858	14
9	1040.491	1022.481	1023.464	520.746	N(+1622.58)	1589.683	1571.673	1572.656	795.342	13
10	1139.560	1121.549	1122.516	570.280	V	1475.644	1457.630	1458.614	738.320	12
[illegible]	[illegible]	[illegible]	[illegible]	[illegible]	[illegible]	[illegible]	[illegible]	[illegible]	[illegible]	[illegible]

Note: Uncheck the alignment checkbox in the spectrum annotation view to see the highlighted fragment ions more easily.

5.4.6.1 Ion Match Table - Predicted matching table

While in mirror plot view, the Ion Match table updates to display the ion matches between fragment query ions and predicted ions.

Ion Match

#	Label	Predicted M/Z	Predicted Intensity (%)	Query M/Z	Query Intensity
1	b2	258.15	6.67	258.14	4.06E2(5.86%)
2	y2	310.18	48.38	310.17	2.02E3(29.15%)
3	b3	345.18	5.21	345.17	5.24E2(7.56%)
4	y3	409.24	20.13	409.24	1.51E3(21.79%)
5	b4	508.24	7.93	508.24	3.23E2(4.66%)
6	y4	572.31	38.81	572.30	3.5E3(50.51%)
7	b5	595.27	12.40	595.27	7.17E2(10.35%)
8	y5	671.38	14.42	671.37	9.8E2(14.14%)
9	b6	694.34	55.14	694.33	2.81E3(40.55%)
10	y6	758.41	41.06	758.40	2.88E3(41.56%)
11	b7	857.40	63.27	857.40	4.37E3(63.06%)

Predicted intensity and Query intensity are reported relative to the highest intensity ion. All columns can be rearranged and sorted.

5.4.7 Survey Chart

The “Survey” tab displays the precursor ion spectrum. The buttons that appear in this section are identical to those explained above in 4.4.5.3 Annotated Spectrum Chart - Additional Chart Controls.

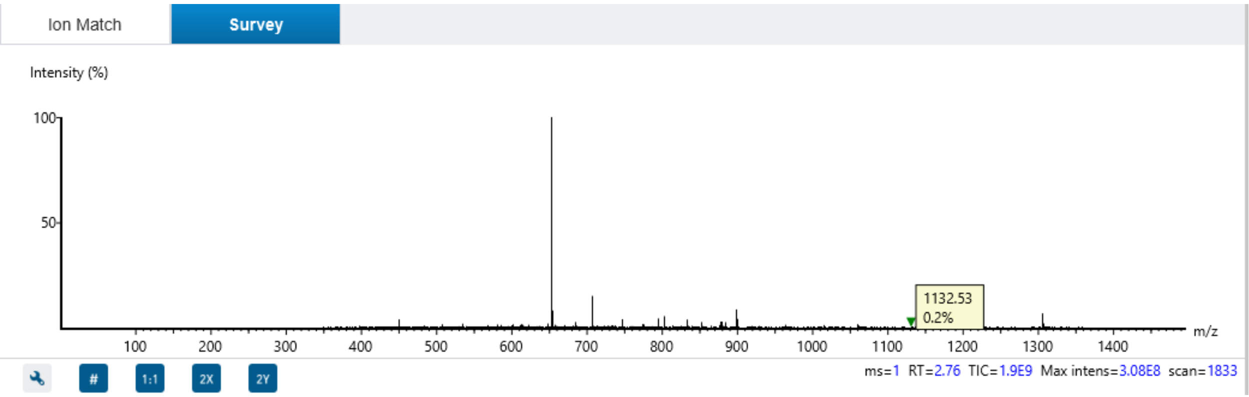

5.4.8 LC/MS Snapshot

The LC-MS Snapshot displays a zoomed-in view of the precursor scan in the LC-MS heat map 2-D view. The span of the orange line in the LC-MS Snapshot designates the isolation window.

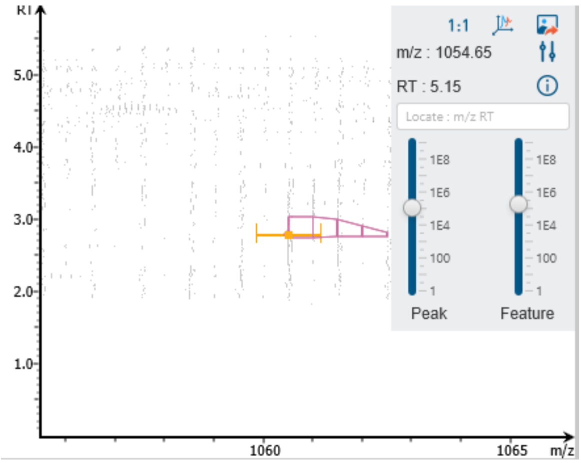

Users can change the view from the 2-D heat map to a 3-D intensity view by clicking the intensity view button in the upper right-hand corner of the LC-MS Snapshot. In addition, users can export the LC-MS heat map by clicking the export image button in the upper right-hand corner of the LC-MS Snapshot.

Expand the options dropdown by clicking to reveal a search option to specify a *m/z RT* (ensure there is a space between these two numbers) location to zoom into. There is also a Peak slider filter to filter out low intensity peaks and a feature area filter to filter out low area features.

5.4.9 Precursor Profile

The Precursor Profile, or eXtracted Ion Chromatogram (XIC) of the precursor ion, graphs the elution profile of the selected peptide feature vector over the retention time range where it was identified. The graph is plotted with the retention time along the x-axis and the intensity along the y-axis. The first two isotopes are also drawn as M+1 and M+2 respectively.

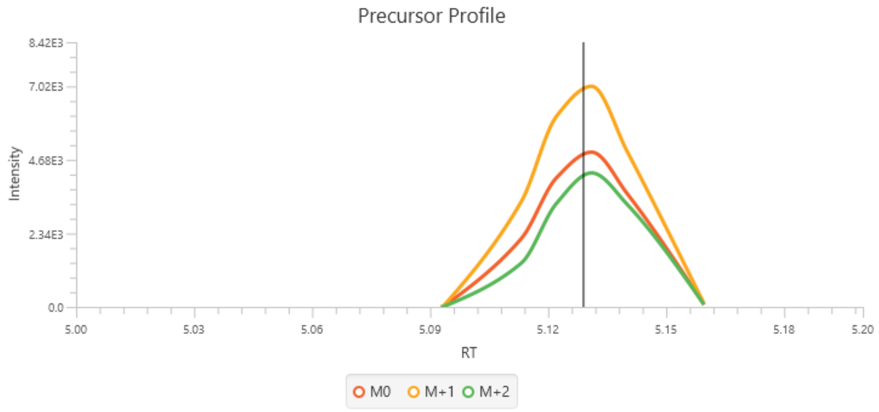

5.5. Deep Novo result - Exporting

In Project View, double-click on Export to open the Export node.

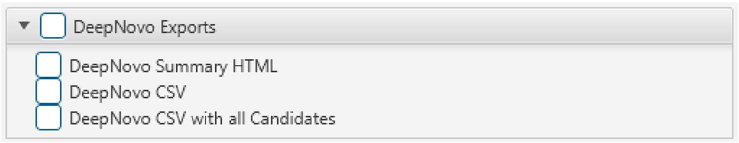

There are several options for exporting the Deep Novo results:

- DeepNovo Summary HTML: Exports the Summary page including all figures in HTML format. The content will be the same as shown in PEAKS Studio.
- DeepNovo CSV: Exports the Deep Novo table. This export does not consider any sorting and will export a separate file for each sample.
- DeepNovo CSV with all Candidates: Exports the Deep Novo all candidates. This export does not consider any sorting and will export a separate file for each sample.

5.6 Peptide Search result

Double-click on the Peptide Search result node to open the result. The analysis results for peptide search tools are presented in two tabs:

- **Summary:** This tab shows an outline of the PEAKS Studio peptide search results with key statistics. The overall quality of the experiment can be examined and the filters for peptide and protein identifications can be adjusted. This page shows after the search is done.
- **Peptide:** This tab shows a list of peptide identifications. This view also provides spectrum annotation and other detailed information for peptide precursor spectrum matches.

The following sections will explore each of these two tabs in the Identification result.

5.6.1 Peptide Search result - Summary tab

The Summary view reports key statistics as an overview of the result. In the report, several statistical charts can be examined to assess result quality.

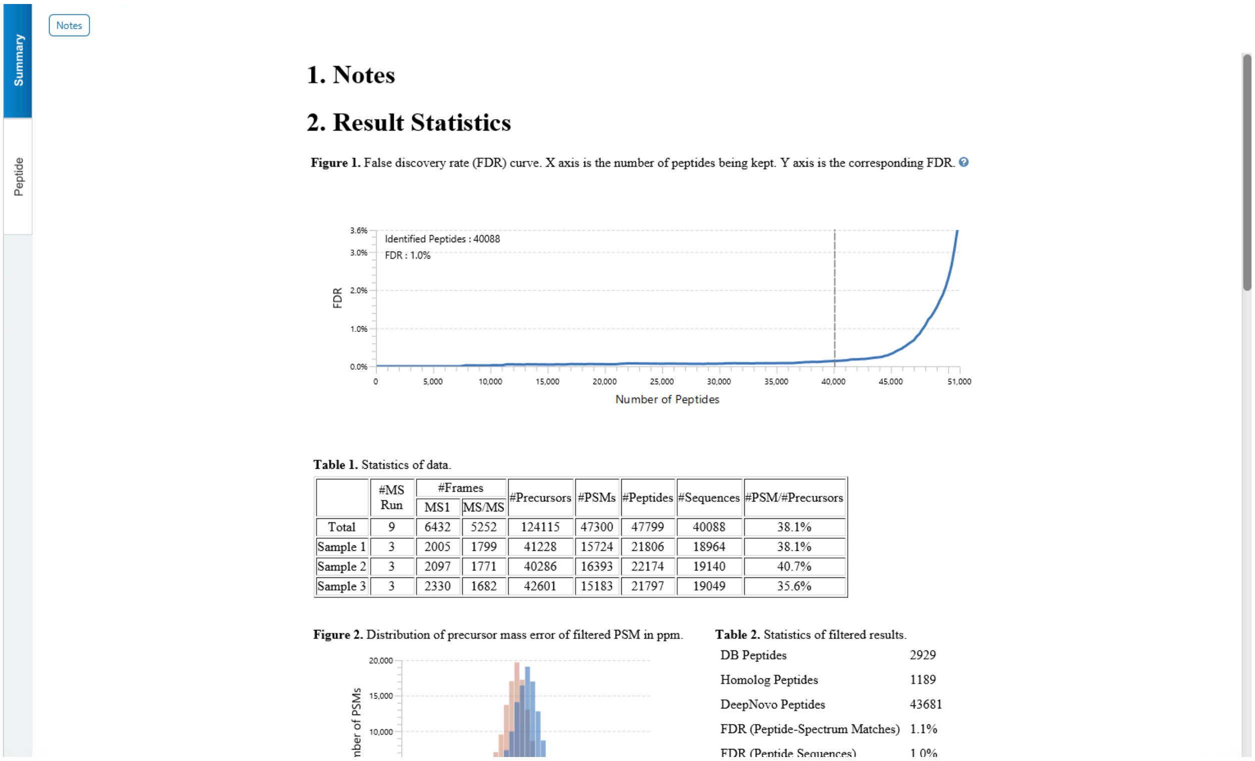

	#MS Run	#Frames		#Precursors	#PSMs	#Peptides	#Sequences	#PSM/#Precursors
		MS1	MS/MS					
Total	9	6432	5252	124115	47300	47799	40088	38.1%
Sample 1	3	2005	1799	41228	15724	21806	18964	38.1%
Sample 2	3	2097	1771	40286	16393	22174	19140	40.7%
Sample 3	3	2330	1682	42601	15183	21797	19049	35.6%

DB Peptides	2929
Homolog Peptides	1189
DeepNovo Peptides	43681
FDR (Peptide-Spectrum Matches)	1.1%
FDR (Peptide Sequences)	1.0%

Click on Notes to open the Notes dialog to save useful information regarding this Analysis. The Notes will appear in Section 1 of the summary page at the top.

The Summary view reports key statistics as an overview of the result. In the report, several statistical charts can be examined to assess result quality.

5.6.1.1 Peptide Search result - Figure 1. False Discovery Rate (FDR) curve.

Figure 1 shows the FDR curve with respect to the number of PSMs being kept after filtration by the peptide -10lgP score. By lowering the score threshold, more PSMs are kept in the filtered result. Conversely, the FDR increases because more false positives are kept. In this figure, the vertical line indicates the current score threshold. The number of PSMs and the corresponding FDR at the current score threshold are shown in the top-left corner. PEAKS Search estimates FDR using the "decoy-fusion" approach. Decoy-fusion is an enhanced target-decoy approach that makes more conservative FDR estimations. For details of the "decoy-fusion" approach, please refer to the publication, *"PEAKS DB: De Novo sequencing assisted database search for sensitive and accurate peptideidentification", Mol Cell Proteomics, 2011 Dec 20.*

It is commonly recommended to set FDR at <1% by adjusting the score threshold. If a rapid growth of the FDR around the 1% FDR threshold is noticed, some PSMs may be sacrificed to significantly reduce the FDR.

Note: When counting the number of PSMs, PEAKS Studio keeps one peptide per spectrum at most. Thus, the number of PSMs is actually the number of spectra with assigned peptides.

Note: During Analysis creation, if Peptide FDR (%) filter was used in Report step, the Summary page FDR curve in the results will show the Peptide FDR curve and corresponding number of Identified peptides.

Note: Decoy matches are not counted in the number of PSMs. Unless otherwise specified, decoy matches are also excluded from the other statistical numbers shown in the Summary view.

5.6.1.2 Peptide Search result - Table 1. Statistics of data

Statistics of Data tabulates MS1 and MS2 information, as well as identification in the peptide and protein level. This table in the Summary view provides a condensed statistical information for the analysis.

Table 1. Statistics of data.

	#MS Run	#Frames		#Precursors	#PSMs	#Peptides	#Sequences	#PSM/#Precursors
		MS1	MS/MS					
Total	9	6432	5252	124115	47300	47799	40088	38.1%
Sample 1	3	2005	1799	41228	15724	21806	18964	38.1%
Sample 2	3	2097	1771	40286	16393	22174	19140	40.7%
Sample 3	3	2330	1682	42601	15183	21797	19049	35.6%

#MS Run: The number of data files in each sample

#MS1: Total # of MS1 spectra for each sample. For timsTOF data this shows the # MS1 frames.

#MS/MS: Total # of MS2 spectra for each sample. This does not include chimeric scans. For timsTOF data this shows the # MS2 frames.

#Precursors: The number of precursors in the result. Only displayed for timsTOF data.

#PSMs: The number of peptide-spectrum matches.

#Peptides: The unique number of peptide sequences with modifications not including I/L differentiation.

#Sequences: The unique number of peptide sequences not including modifications and I/L differentiation.

#PSM/MS2: The identification rate calculated by dividing #PSM by the #MS2. For timsTOF data, it is calculated by dividing #PSM by the #Precursors

5.6.1.3 Peptide Search result - Figure 2. PSM score distribution

Figure 2 in the Summary View show the precursor mass errors of PSMs in filtered results. It shows the distribution of the precursor mass errors in a histogram with and without software calibration. The precursor mass error is calculated in ppm as $10^6 \times$ (precursor mass - peptide mass) / peptide mass. Calibration is for display purposes only and PEAKS corrects m/z value for each PSM to correct systematic biases and instrument measurement drift. For a well-calibrated instrument, precursor mass errors should center at 0 ppm across the range of m/z.

Figure 2. Distribution of precursor mass error of filtered PSM in ppm.

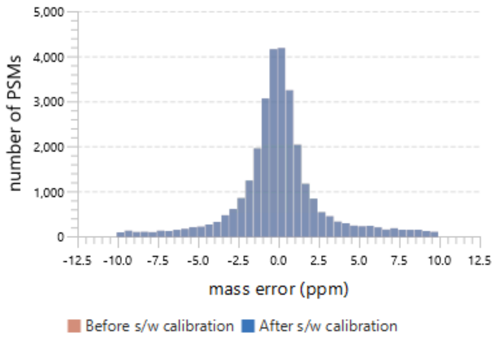

5.6.1.4 Peptide Search result - Result filtration parameters

Tables 2 show the statistical numbers of the data and results based on report filters set.

Table 2. Statistics of filtered results.

DB Peptides	5731
Homolog Peptides	67
DeepNovo Peptides	12480
FDR (Peptide-Spectrum Matches)	1.0%
FDR (Peptide Sequences)	1.3%
-10LgP Cutoff (Peptide-Spectrum Matches)	0.01907082

5.6.1.5 Peptide Search result - RT Figures

RT figures with outliers removed are displayed for the first data file in the analysis.

Figure 3(a) shows the ΔRT distribution with a histogram. Peptides found by Database Search, DeepNovo, and Homolog are separated into colour-coded bars. Figure 3(b) shows the alignment of predicted RT and real RT in minutes. The peptides found are split into different colour-coded points in the scatterplot and a regression function is drawn to indicate the correlation.

Figure 3. **(a)** Histogram of peptide ΔRT. **(b)** Scatterplot of peptide RT versus Predicted RT.

RT figures for 20180924_50ngHeLa_1.0.25.1_Hystar5.0SR1_S2-A1_1_2042.d with outliers removed

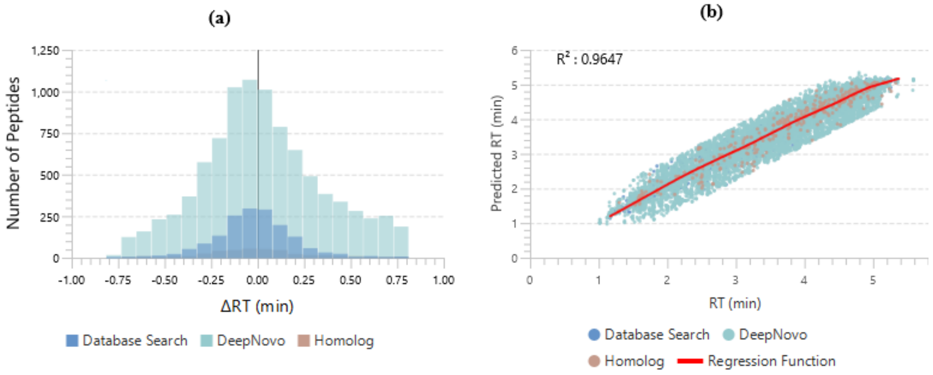

For timsTOF data, a Regression plot of 1/k0 is also provided in Figure 4.

Figure 4. Regression plot of peptide 1/k0 versus Predicted 1/k0.

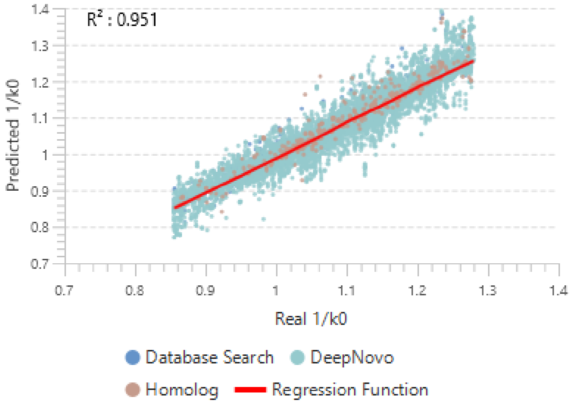

5.6.1.6 Peptide Search result - Boxplot

Figure 5 shows a boxplot of the median ΔRT across all MS runs.

Figure 5. Boxplot for median ΔRT across all MS runs.

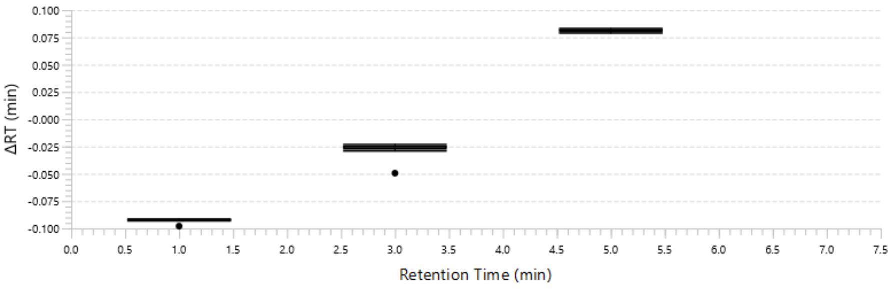

5.6.1.7 Peptide Search result - Other Information

Table 3 summarizes the parameters defined by the user to analyze the data. This information includes error mass tolerances, database(s) used, etc.

3. Other Information

Table 3. Search parameters.

Search Engine Name: PEAKS
Parent Mass Error Tolerance: 20.0 ppm
Fragment Mass Error Tolerance: 0.05 Da
Enzyme: Trypsin
Fixed Modifications:
  Carbamidomethylation (+57.02)
Variable Modifications:
  Deamidation (NQ) (+0.98)
  Oxidation (M) (+15.99)
Max Variable PTM Per Peptide: 2
Database: uniprot_sprot_June-2-2020.fasta
Taxon: Homo sapiens (human)
Searched Entries: 20376
Deep Novo ALC: 50.0%
Deep Novo Protein Association Tag Sharing: 5

5.6.2 Peptide Search result - Peptide Tab

The Peptide Table shows the filtered native peptide identification results. Each row in the table is a native peptide identification represented by its highest-scoring PSM. When there are more than 1000 peptides, the table is split into multiple pages.

Note: If there are multiple PSMs matched to a peptide, the table displays the information for the top-scoring PSM. Peptides with the same sequence but different modifications are considered different and shown in different rows.

To copy the sequence, right click on a row (or multiple rows) in the table and select “Copy selected peptide sequence(s)”. Another option is to mouse over any cell in the table and hitting Ctrl + C on your keyboard to copy the contents of those selected cells.

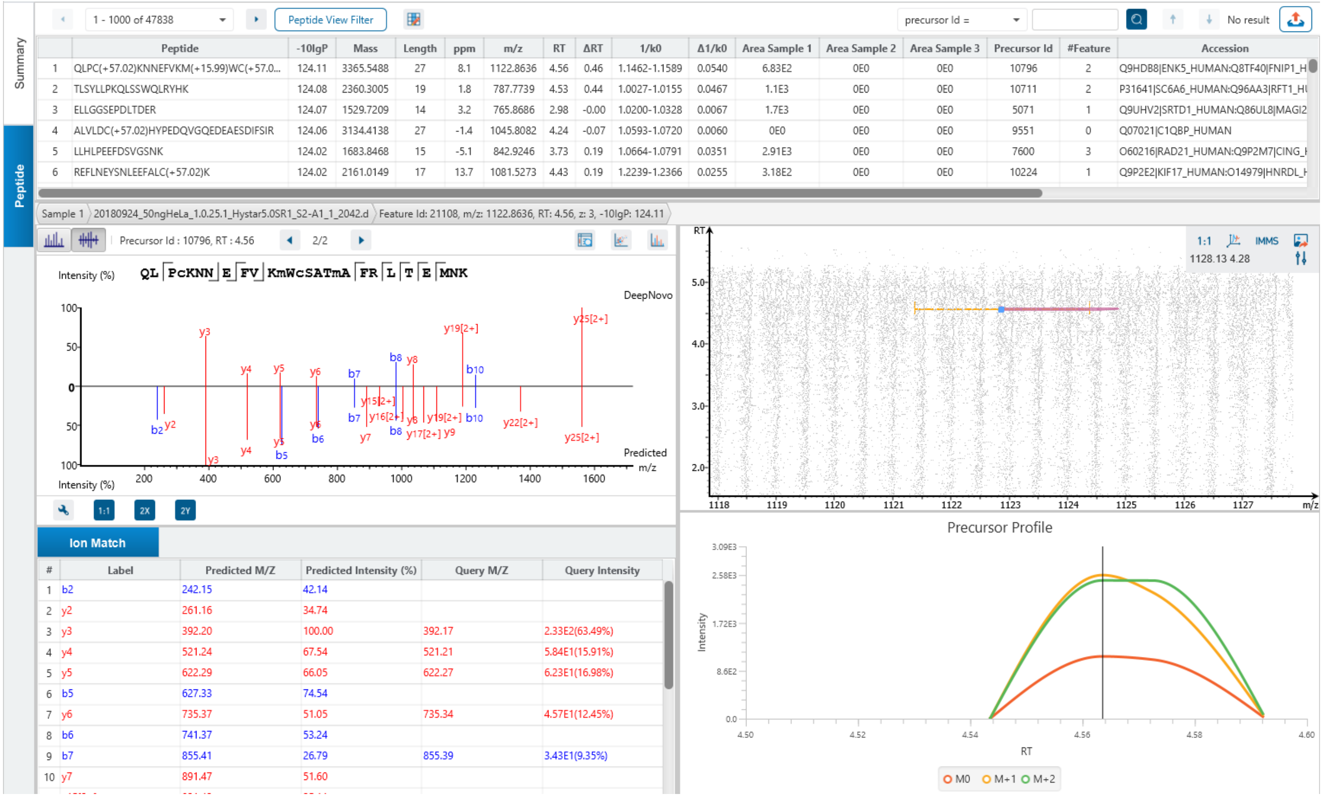

The following list describes the contents in each of the default columns:

- **Peptide:** The amino acid sequence of the peptide, as determined by the PEAKS search workflow. A modified residue is followed by a pair of parentheses enclosing the modification.
- **-10lgP:** The peptide -10lgP score. The score indicates the scoring significance of a peptide-precursor spectrum match.
- **Mass:** The monoisotopic mass of the peptide.
- **Length:** The number of amino acids in the sequence backbone.
- **ppm:** The precursor mass error, calculated as $10^6 \times$ (precursor mass - peptide mass) / peptide mass.
- **m/z:** The precursor mass-to-charge ratio.
- **RT:** The retention time (elution time) of the spectrum as recorded in the data.
- **ΔRT:** The difference between predicted RT and RT of peptide.
- **1/k0:** The 1/k0 range for timsTOF data only.
- **Δ1/k0:** The difference between average predicted 1/k0 and 1/k0 of peptide for timsTOF data.
- **Area per Sample:** The combined area for all features in that sample associated with this peptide.
- **Scan / Precursor Id:** The scan number of the spectrum that matches the peptide sequence with the highest -10lgP. For timsTOF, this is the Precursor Id instead.

- **#Feature:** For single sample analysis, shows number of features assigned to the peptide sequence from identification.
- **Accession:** The accessions of proteins that contain this peptide.
- **PTM:** The types and the numbers of modifications present in the peptide shown in color-coded icons.
- **AScore:** Localization score assigned to modifications on the peptide. It is the -10 log of a p-value. In this case, the p-value is the probability that the modification occurs at the reported position compared to other possible positions. A -10lgP of 20 is equal to a p-value of 0.01. Scores are listed in the format amino acid, amino acid residue position, modification, AScore. For example, serine at amino acid 3 is phosphorylated with an AScore of 54.01 will be reported as: S3 P 54.01.
- **Found By:** The name of the PEAKS search workflow that identifies the peptide; this can be either Database Search, Homolog, or DeepNovo.

5.6.3 Peptide Table controls

The menu above the peptide table provides options to scroll through the table of peptides, filter the table, show optional columns and search for specific entries.

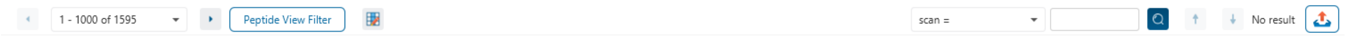

5.6.3.1 Peptide Table controls - Pagination

For easy viewing and to improve performance, the peptide table is limited to showing 1000 entries at once. Pagination is used to manage which set of 1000 entries will be viewed.

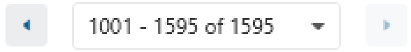

In the top left, there is a dropdown to select the rows for viewing. Click the left arrow or right arrow to select the previous/next 1000 rows. If the table is sorted or filtered, it may update the pagination set of rows that is currently in view.

5.6.3.2 Peptide Table controls - View Filter

Click on the 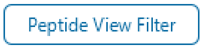

button to open the Peptide View Filter which is used to filter the Peptide table. The following are the filter options provided:

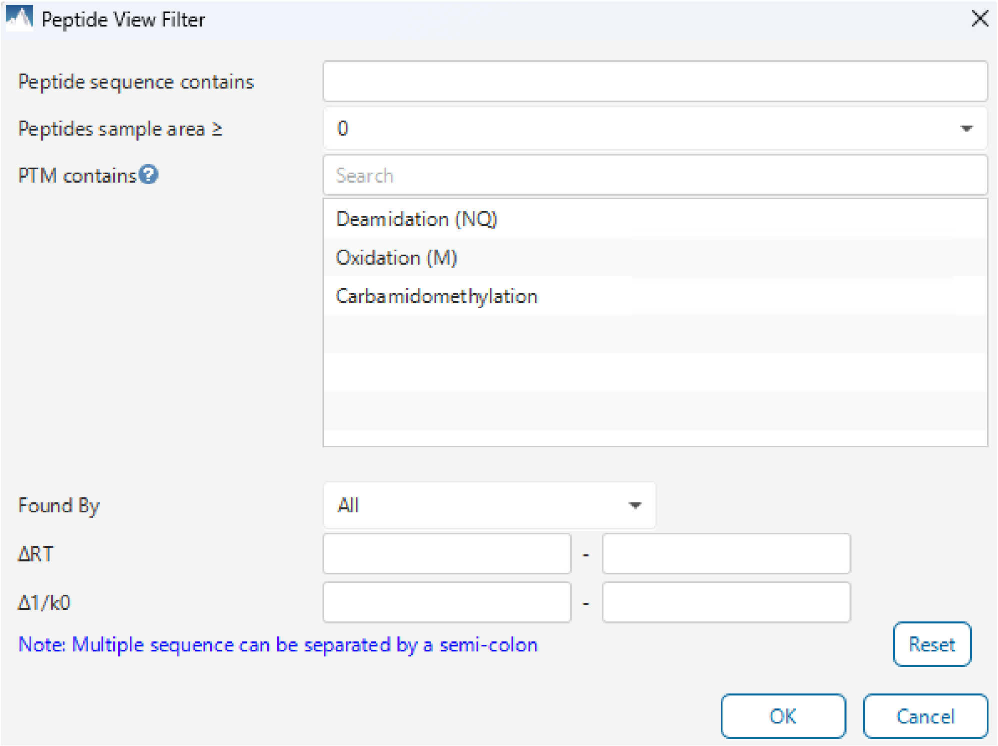

- Peptide sequence contains: Enter an amino acid sequence, only peptides that contain that sequence will be displayed in the peptide table. Adding PTMs in brackets in the sequence search is not handled, meaning only the sequence backbone is considered in the filter. Use the PTM contains for further filtering if necessary.
- Peptide sample area: Filters based on total peptide area found in each sample. Sample areas are calculated using the total of all peptide features from all spectra that identified the native peptide. If the peptide is found in multiple samples, only one sample must pass this filter for the peptide to be included.
- PTM contains: Type the names of PTMs you would like to include. Peptides containing PTMs that match the text will be included. If multiple PTMs match the text they will all be included in the table.
- Found By: A dropdown that allows selection of only keeping peptides found by Database Search, DeepNovo, or Homolog.
- ΔRT: The filter for the range of RT difference.
- Δ1/k0: The filter for the range of 1/k0 difference. Only shown for timsTOF data.

Note: In all filters, multiple entries can be separated by a semi-colon.

Click on the Reset button to reset all filters to their default setting.

Click on OK to apply the filters to the Peptide table.

Click on Cancel to discard the changes in the View Filter.

5.6.3.3 Peptide Table controls - Optional Columns

Click on the 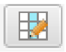 button to open the optional columns dropdown. By default the sample area columns are displayed. Sample Feature count columns can also be shown. These sample feature counts are always from identification, whether or not there are feature vectors present. For projects with many samples, it might be useful to hide these optional columns and reduce the width of the peptide table.

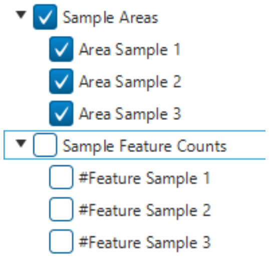

5.6.3.4 Peptide Table controls - Search function

On the top right of menu, there is a search function with 4 options. Search results can be navigated between using the Up or Down arrows.

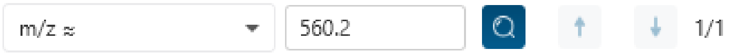

Options for searching include:

- **Scan =:** Search for the Scan number associated with each peptide. One scan can be associated to multiple peptides. For projects with multiple data files, the scan number is searched on every fraction. For timsTOF data, this search function is for Precursor Id rather than Scan.
- **m/z ≈:** Search for the approximate m/z. The search will consider a range of m/z equal to the search value +/- 1 of the last significant digit. For example, searching for m/z ≈ 560.21 will find all m/z between [560.20, 560.22]
- **RT ≈:** Search for the approximate RT. Likewise, the search will consider a range of RT equal to the search value +/- 1 of the last significant digit set. For example, searching for RT ≈ 55.5 will find all m/z between [55.4, 55.6]
- **Seq contains:** Search for the sequence in the table. If specifying a sequence without PTMs, the results will include that sequence and any sequences with PTMs with the same backbone. If specifying a sequence with bracket and some mass value (like in the above image), then only sequence that have a substring with an exact match will be considered.
  Additionally, since the peptide table shows both I/L and they can not be distinguished, then searching for I in the sequence is equivalent to searching for L in the sequence. i.e. Searching for "LVLK" is the same as searching for "LVIK", "IVLK", and "IVIK"

5.6.4 Feature selection breadcrumb

There is a sample and feature selection breadcrumb which allows you to switch between features associated with the peptide. Click on the Sample in the breadcrumb to reveal other samples and the features in that sample related to the current peptide selection. From here you can select other samples, fractions, or features.

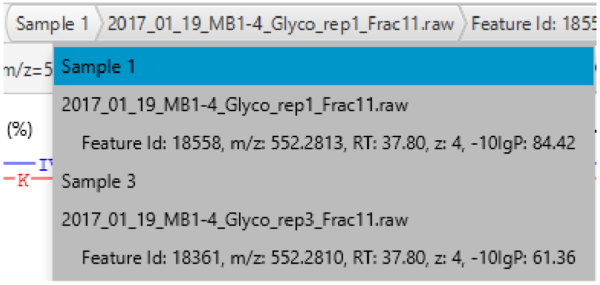

By clicking on the fraction level, all fractions in this sample are revealed as well as the features in each fraction associated with this peptide. From here you can select other fractions and features to view.

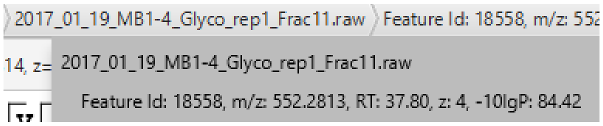

Lastly, clicking on the feature level will show all features in the currently selected fraction. Features with Feature Id = 0 are known as fake features and are constructed to enhance identification.

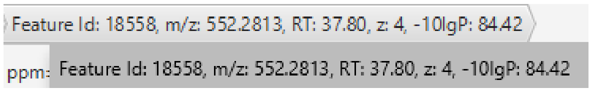

5.6.5 Peptide View - PSM selection

Underneath the feature breadcrumb will shown the currently selected PSM. Click on the Left and Right arrow buttons to switch between PSMs related to the currently selected feature.

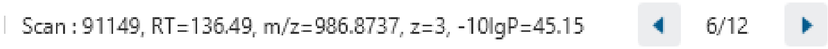

All other components in the Peptide tab interface will update according to the currently selected PSM.

For timsTOF data, Precursor Id is used rather than Scan.

5.6.5.1 Show spectrum in Data View

Click on 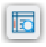 to open the Data Refine MS/MS tab (if not already opened) and jump to the corresponding selected Scan.

5.6.5.2 Show spectrum in LC/MS View

Click on 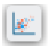 to open the Data Refine LC/MS tab (if not already opened) and jump to the corresponding selected Scan, highlighting the peptide MS/MS marker and the feature if present.

5.6.5.3 Show Raw Spectrum View

Click on 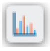 to show the raw spectrum chart of the currently selected Scan.

5.6.6 Peptide View - Annotated Spectrum Chart

Click on button to switch from the Mirror plot view and annotated spectrum chart view.

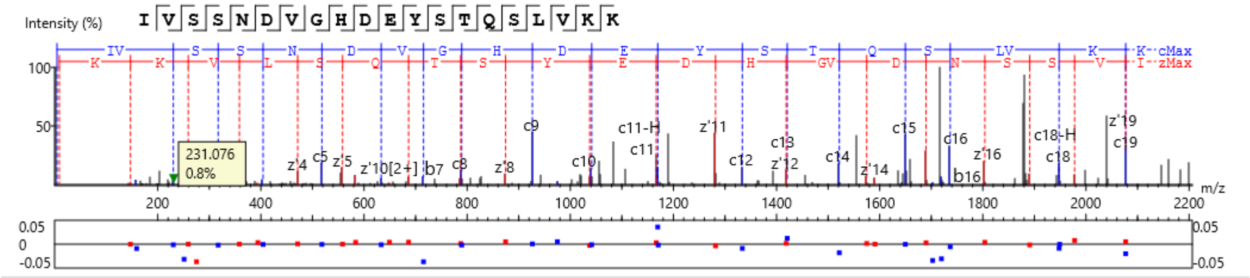

For the currently selected PSM, a graphical representation is shown in the form of an annotated spectrum chart. There are several controls provided for this view which will be explored in the following sections.

5.6.6.1 Annotated Spectrum Chart - Chart Navigation

Moving the cursor over the peptide sequence in the spectrum will show the mass transitions for a particular amino acid residue.

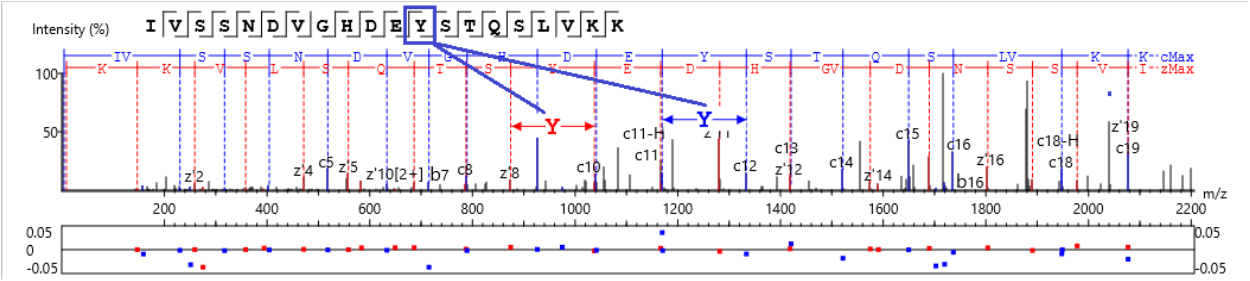

Zoom to a m/z region: Drag horizontally from the start m/z and to the end m/z with left mouse button.

Zoom in/out smoothly: Place the cursor at a particular m/z value (right below the x-axis), zoom in/out by scrolling the mouse wheel.

Increase/Decrease peak intensity: Place the cursor in the spectrum and scroll the mouse wheel.

See the whole spectrum: Double click in the spectrum or click the 1:1 button.

5.6.6.2 Annotated Spectrum Chart - Spectrum Annotation Settings

Click on the button to show the spectrum annotation settings.

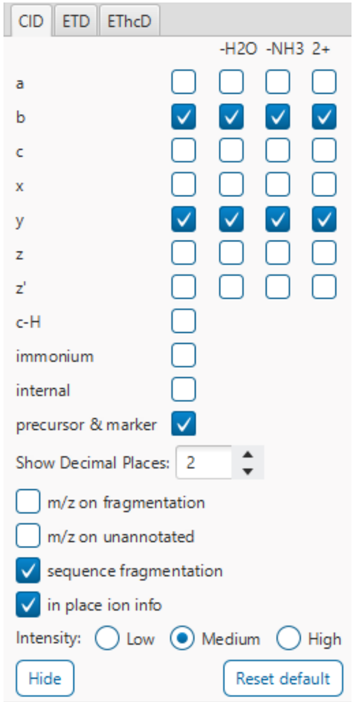

The spectra in the PEAKS results can be annotated by selecting ion types from the thorough collection of ions that PEAKS offers. The selected ion types will be displayed in the "Ion Match" table, as well. It is possible to annotate the spectrum with various ions for CID, EThcD and ETD. Each of these fragmentation modes can be set with different settings which will be shared with other results with the same fragmentation type.

To reset the defaults, click on the "Reset default" button.

Show Decimal Places: Select the number of decimal places that will appear in the ion table and the spectrum view. The default is set to two decimal places.

m/z on Fragmentation: Select this to display the m/z value on top of the annotated ions.

m/z on Unannotated: Select this to display the m/z value on top of the peaks without ion matches.

Sequence Fragmentation: Select this to display the sequence fragmentation on the top-left corner of the "Spectrum Annotation" view.

In Place Ion Info: Ion information, m/z value,and relative intensity are all displayed in a pop-up within the "Spectrum Annotation" view when this option is checked and the cursor is placed on a peak.

Intensity: Set the intensity threshold for spectrum annotation to low (2%), medium (5%), or high (10%).

5.6.6.3 Annotated Spectrum Chart - Additional Chart Controls

Click on 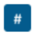

or 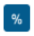

to toggle between absolute and percentage intensity values in the Y axis.

Click on 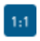

to reset the zoom level of the annotated spectrum chart to the default.

Click on 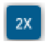

to zoom into the X axis by a factor of 2.

Click on 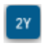

to zoom into the Y axis by a factor of 2.

The "alignment" check box ☑ alignment allows user to display/hide how the fragment ions generated from the peptide align with the spectrum. N-terminal ions are shown in blue and C-terminal ions are shown in red.

The "error map" check box ☑ error map is used to show/hide the error map underneath the chart.

5.6.6.4 Annotated Spectrum Chart - Mirror Plot

Spectral Library results have a Mirror Plot view. Click on the button to switch to the Mirror Plot.

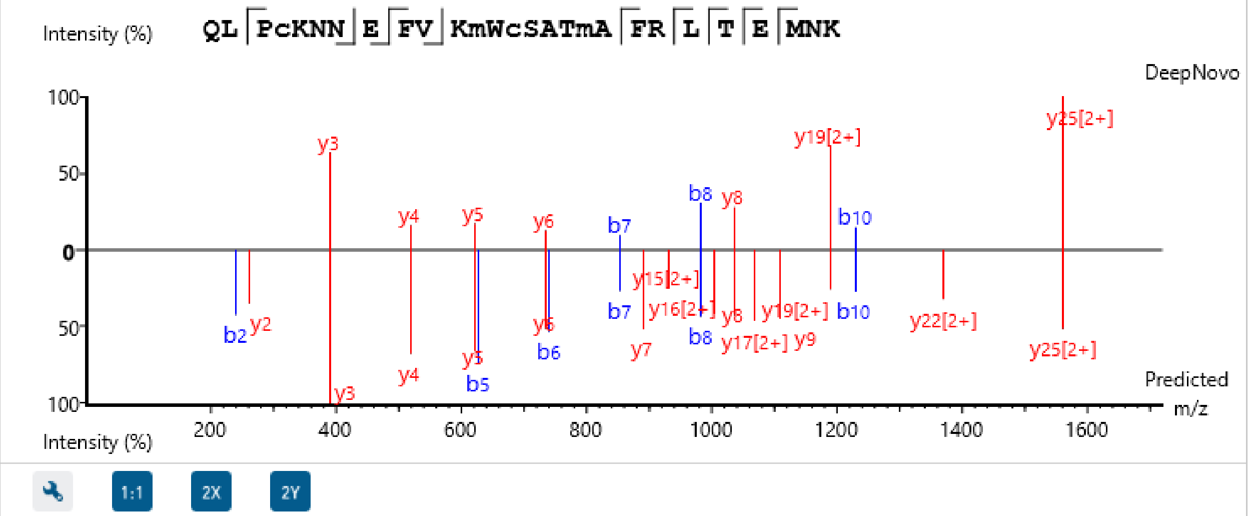

This view shows the ions from the query spectrum compared to the library spectrum.

5.6.6.5 Peptide View - Ion Match Table

The "Ion Match" tab in the bottom pane contains a table with possible fragment ions for the selected MS/MS scan. Each ion in the table shows the calculated mass, where if the fragment ion is present in the spectrum, its mass value is displayed in color. N-terminal ions are shown in blue and C-terminal ions are shown in red. A fragment ion is found when there is a matching peak within the mass error tolerance, as defined in the de novo sequencing parameters, and when relative intensity of the matching peak is at least 2%. The ion types displayed in the table can be configured in "Spectrum Annotation Settings".

Ion Match | Survey

#	b	b-H2O	b-NH3	b(2+)	Seq	y	y-H2O	y-NH3	y(2+)	#
10	1079.503	1061.488	1062.498	540.248	E	1596.643	1578.636	1579.610	798.818	13
11	1265.576	1247.569	1248.557	633.292	W	1467.601	1449.588	1450.585	734.297	12
12	1394.626	1376.616	1377.600	697.813	E	1281.521	1263.512	1264.488	641.266	11
13	1481.659	1463.648	1464.632	741.329	S	1152.480	1134.469	1135.461	576.736	10
14	1596.682	1578.671	1579.655	798.841	N(+0.98)	1065.449	1047.440	1048.427	533.228	9
15	1653.703	1635.693	1636.676	827.352	G	950.422	932.407	933.390	475.709	8
16	1781.773	1763.751	1764.735	891.381	Q	893.397	875.388	876.370	447.198	7
17	1878.815	1860.804	1861.788	939.907	P	765.341	747.326	748.315	383.169	6
18	2007.857	1989.847	1990.830	1004.429	E	668.288	650.274	651.258	334.642	5
19	2122.880	2104.870	2105.853	1061.940	N(+0.98)	539.245	521.231	522.222	270.124	4
[illegible]	[illegible]	[illegible]	[illegible]	[illegible]	[illegible]	[illegible]	[illegible]	[illegible]	[illegible]	[illegible]

Note: Uncheck the alignment checkbox in the spectrum annotation view to see the highlighted fragment ions more easily.

5.6.7 Peptide View - LC/MS Snapshot

The LC-MS Snapshot displays a zoomed-in view of the precursor scan in the LC-MS heat map 2-D view. The peptide feature is highlighted in purple and the selected PSM marker is blue. The span of the orange line in the LC-MS Snapshot designates the isolation window.

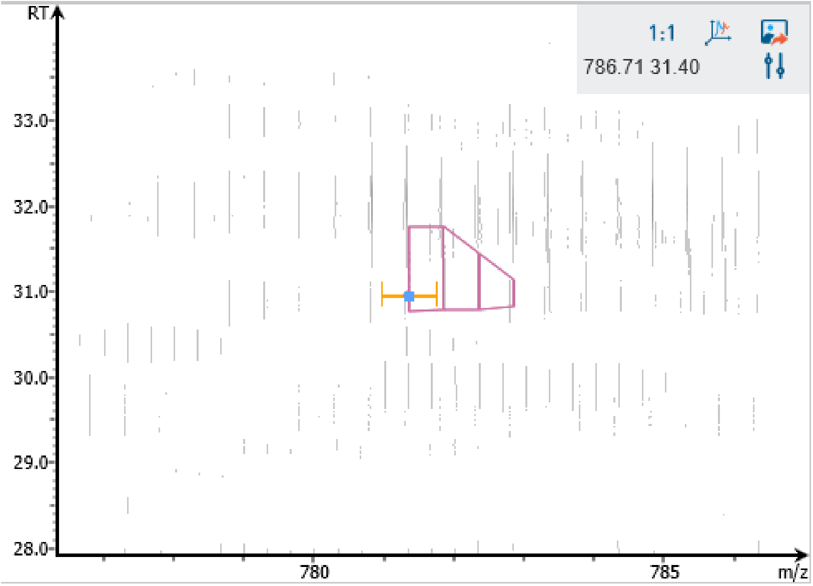

Users can change the view from the 2-D heat map to a 3-D intensity view by clicking the intensity view button in the upper right-hand corner of the LC-MS Snapshot. In addition, users can export the LC-MS heat map by clicking the export image button in the upper right-hand corner of the LC-MS Snapshot.

Expand the options dropdown by clicking to reveal a search option to specify a *m/z RT* (ensure there is a space between these two numbers) location to zoom into. There is also a Peak slider filter to filter out low intensity peaks and a feature area filter to filter out low area features.

5.6.8 Peptide View - Precursor Profile

The Precursor Profile, or eXtracted Ion Chromatogram (XIC) of the precursor ion, graphs the elution profile of the selected peptide feature vector over the retention time range where it was identified. The graph is plotted with the retention time along the x-axis and the intensity along the y-axis. The first two isotopes are displayed in the chart as well as M+1 and M+2.

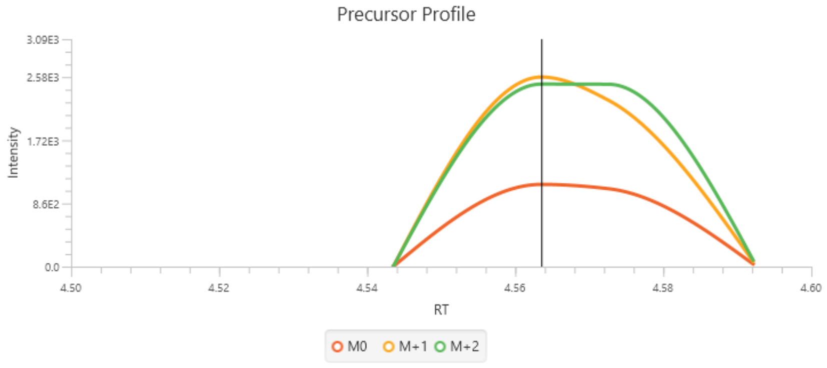

5.7 Peptide Search result - Exporting

In Project View, double-click on Export to open the Export node.

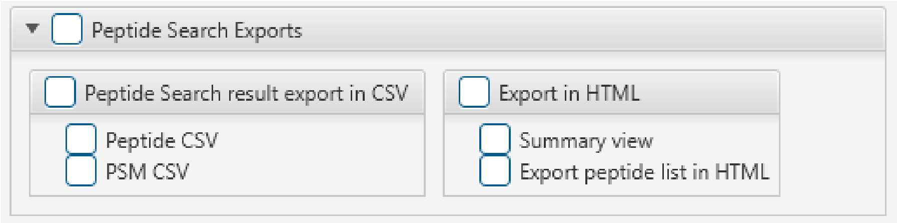

5.7.1 Peptide Search result - Exporting - comma separated values (CSV) format

There are several options for exporting the PEAKS Identification results in CSV format:

- **Peptide CSV:** All of the identified peptides and details will be saved to peptides.csv.
- **PSM CSV:** The peptide-spectrum matches (PSMs) will be exported to peptideDn.psms.csv. Peptides differentiated with only I/L isoform are represented by separate entries. As a result, the number of entries in this file might be bigger than the number of PSMs mentioned in the Summary view.

5.7.2 Peptide Search result - Exporting - HTML format

Export in HTML: This will generate single or multiple HTML reports in the specified location.

The following exporting options are available:

- **Summary view:** The “Summary” view page will be saved as a summary.html file in HTML format in the specified location.
- **Export peptide list in html:** creates a separate HTML-containing peptide identifications.

6. Peptide, PTM, and Mutation Identification (PEAKS DB, PEAKS PTM, and SPIDER)

PEAKS Studio provides a complete set of database search tools for in-depth protein analysis. With PEAKS DB, PEAKS PTM, and SPIDER, proteins in the sample can be identified with high sensitivity and accuracy. It is also possible to find potential sites for modification and mutation. PEAKS Studio supports multiple enzyme digestion by which almost full sequence coverage can be achieved for single protein studies. PEAKS Studio database search tools have built-in result validation using an enhanced target-decoy approach. The false discovery rate (FDR) is estimated to ensure that only valid results are reported. PEAKS DB is a database search tool assisted by PEAKS de novo sequencing to achieve high sensitivity and accuracy.

Note: For more details, refer to the paper*: "PEAKS DB: De Novo sequencing assisted database search for sensitive and accurate peptide identification", Mol Cell Proteomics, 2011, Dec 20.*

PEAKS PTM is a dedicated search tool for peptides with unspecified modifications and mutations. It can search with unlimited number of modifications, allowing searches with all modifications in the Unimod database.

Note: For more details, refer to the paper: *"PeaksPTM: Mass Spectrometry Based Identification of Peptides with Unspecified Modifications", Journal of Proteomics Research, 2011, 10(7): 2930-2936.*

SPIDER is a dedicated search tool for finding novel peptides that are homologous to peptides in a given protein database.

Note: For more details, refer to the paper: *"SPIDER: Software for Protein Identification from Sequence Tags Containing De Novo Sequencing Error", J Bioinform Comput Biol, 2005, Jun;3(3):697-716.*

6.1 Database Search Workflow and Parameter Settings

PEAKS DB, PEAKS PTM, and SPIDER can be launched together as a work flow in a PEAKS Studio analysis. Users can also do PEAKS DB only, PEAKS DB plus PEAKS PTM, or PEAKS DB plus SPIDER.

To run a Database Search workflow, select PEAKS DB (In-depth de novo assisted search) in the Project Wizard.

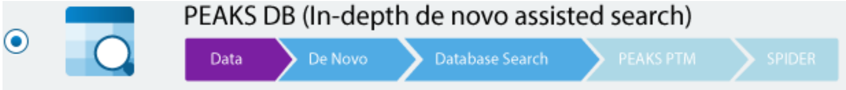

After deciding the Data Refine option, proceed to the next step to see the DB Search parameters.

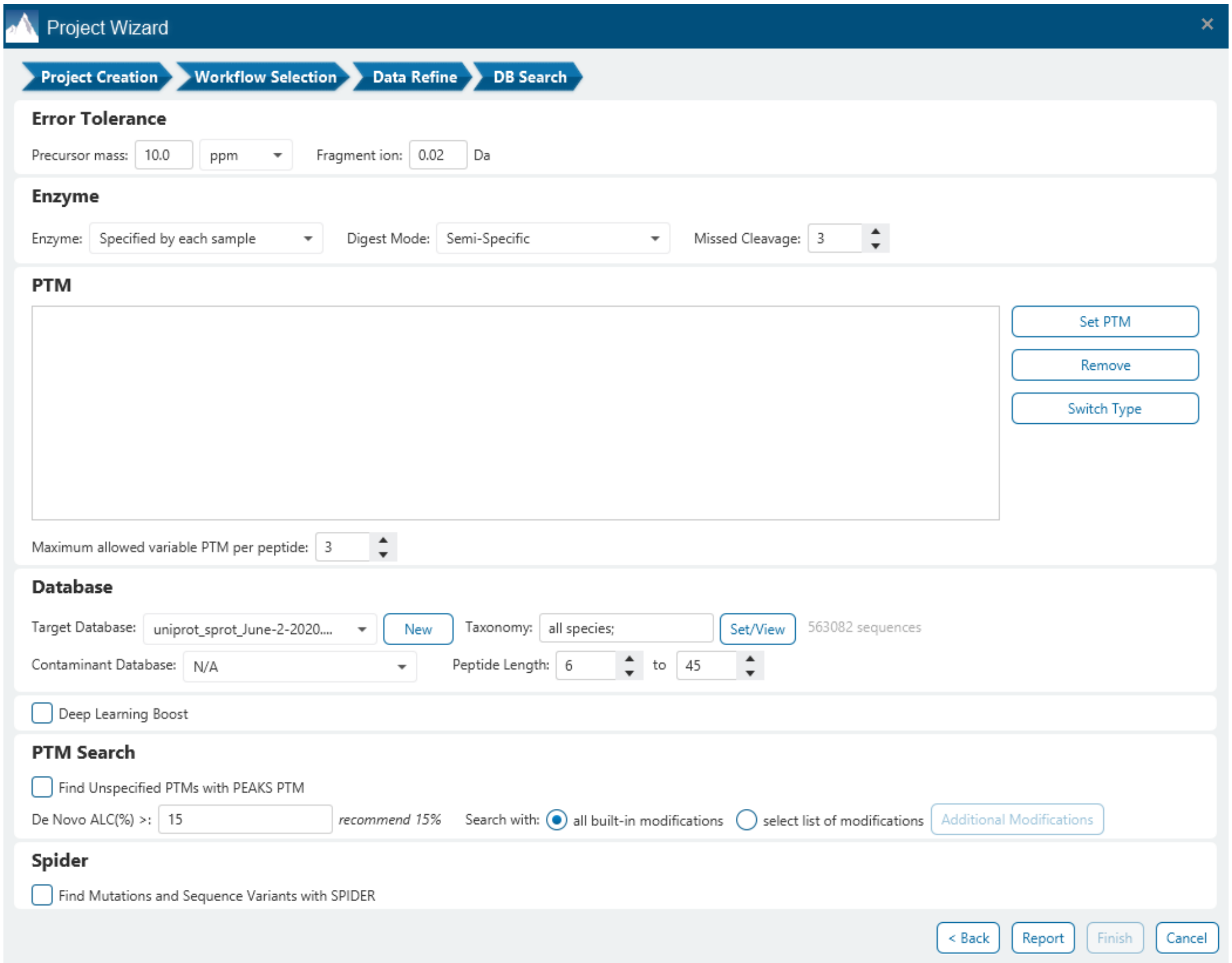

The following parameter settings are provided:

Precursor Mass Error Tolerance: The maximum mass difference between the peptide and the monoisotopic mass of the precursor ion.

Precursor Mass Tolerance unit: PEAKS generates peptides within the precursor mass error tolerance. Precursor mass error tolerance can be specified in either Daltons or ppm.

Fragment Mass Error Tolerance: PEAKS uses this value when scoring peptide sequences. PEAKS Studio considers a fragment ion to be matched if the calculated m/z is within the specified error tolerance. Units are Daltons.

Both precursor and fragment error tolerance parameters need to be set consistently with the mass accuracy of the instrument.

Enzyme Settings: PEAKS Studio digests the protein database *in silico* to generate peptide candidates. It is necessary to specify the enzyme for protein digestion from the Enzyme drop-down menu.

Note: When the selected dataset is digested with different enzymes, "Specified by each sample" allows samples to be analyzed separately using their respective enzymes specified during project creation.

Note: "None" is a special enzyme allowing non-specific cleavage at both ends of the peptide. It is the recommended option when no digestion enzyme was used or when the digestion enzyme exhibits a high degree of non-specificity.

Missed Cleavages: This specifies the number of missed cleavage sites allowed in a peptide.

Digest Mode: This specifies the type of in silico cleavages allowed at a peptide termini based on the method used for protein digestion. If "Specific" is selected, the specificity of the selected enzyme is strictly enforced at both termini of a peptide. "Semispecific" allows one terminus to disobey the enzyme specificity rules.

Note: If the enzyme is specified as "None", then no matter which mode is selected for "Digest Mode", the "None" enzyme digest rule will be applied.

PTM Settings: Click “Set PTM” to open the **PTM Options** dialog and specify the fixed PTMs and a few common variable PTMs expected in the experiment. Highlight PTMs and click on the ➡ button to place them as either Fixed or Variable PTM. From there, highlighted PTMs can be removed by clicking the “Remove” button. Clicking “Remove All” will reset the PTM options. Highlighting and clicking “Switch Type” will move the Fixed PTM in into the Variable PTM list and vice-verse. Click on “New” to create a new custom PTM. While a PTM is selected, click on “View” to see more details.

Note: Multiple PTMs can be highlighted by holding down the CTRL key or SHIFT key on the keyboard.

Note: For a specific residue, only one fixed modification is allowed.

Once the PTMs are selected, the maximum number of variable PTMs per peptide can be specified and a value of less than 4 is recommended.

Note: The use of variable modifications increases the size of the computational search space for the de novo sequencing algorithm. It is recommended not to select too many variable modifications in PEAKS de novo. See PEAKS PTM for alternate search options.

Target Database: Select a protein sequence database for the search. Select one from the list of databases that have been configured in PEAKS and set the taxonomy by clicking on [Set/View], if applicable. To configure a new database, click on [New] next to the Target Database dropdown to open the Database Configuration dialog.

Contaminant Database: To search for contaminants, select a contaminant database from the list of databases that have been configured in PEAKS. Keep the selection as N/A if no contaminant database is required for the search.

Peptide Length: The length for a peptide can be set when creating an analysis, where the default peptide length range is between 6 and 45.

Deep Learning Boost: Check this option to use PEAKS enhanced deeplearning algorithm to better identify PSMs in the DB Search process.

Find Unspecified PTMs with PEAKS PTM: Select this option to enable a PEAKS PTM search after a PEAKS DB search finishes. PEAKS PTM analyzes spectra with good de novo sequences that remain unidentified by PEAKS DB. The default setting for PEAKS PTM is to search with all the built-in modifications in the "Common" and "Uncommon" lists, which include all of the natural modifications in the Unimod database.

Alternatively, PEAKS PTM can search with a list of preferred modifications. Once "Search with preferred modifications" has been selected, click the Additional Modifications button to select desired PTMs for the PTM search. Importantly, PEAKS PTM allows an unlimited number of variable modifications to be searched, however, limiting the number can improve search accuracy. By default, PEAKS PTM considers the same number of maximum number of variable PTMs per peptide as set in the DB search parameters. A threshold on the de novo ALC score can be specified so that only the spectra with good de novo sequences are analyzed by PEAKS PTM. In the PEAKS PTM result node, search results from PEAKS DB and PEAKS PTM are combined and shown.

Find More Mutations with SPIDER: Select this option to enable a SPIDER search. SPIDER performs homology searches on spectra with good de novo sequences (i.e. ALC>15%) that remain unidentified by either PEAKS DB and/or PEAKS PTM. SPIDER identifies novel peptides that are homologous to peptides in the searched protein database. SPIDER is good for cross-species searches and for finding point mutations of the protein. In the SPIDER result page, search results from PEAKS DB, PEAKS PTM (if applicable), and SPIDER are combined and shown.

Click on “Report” to proceed to the final step. Report filters can be set here which will filter the final result.

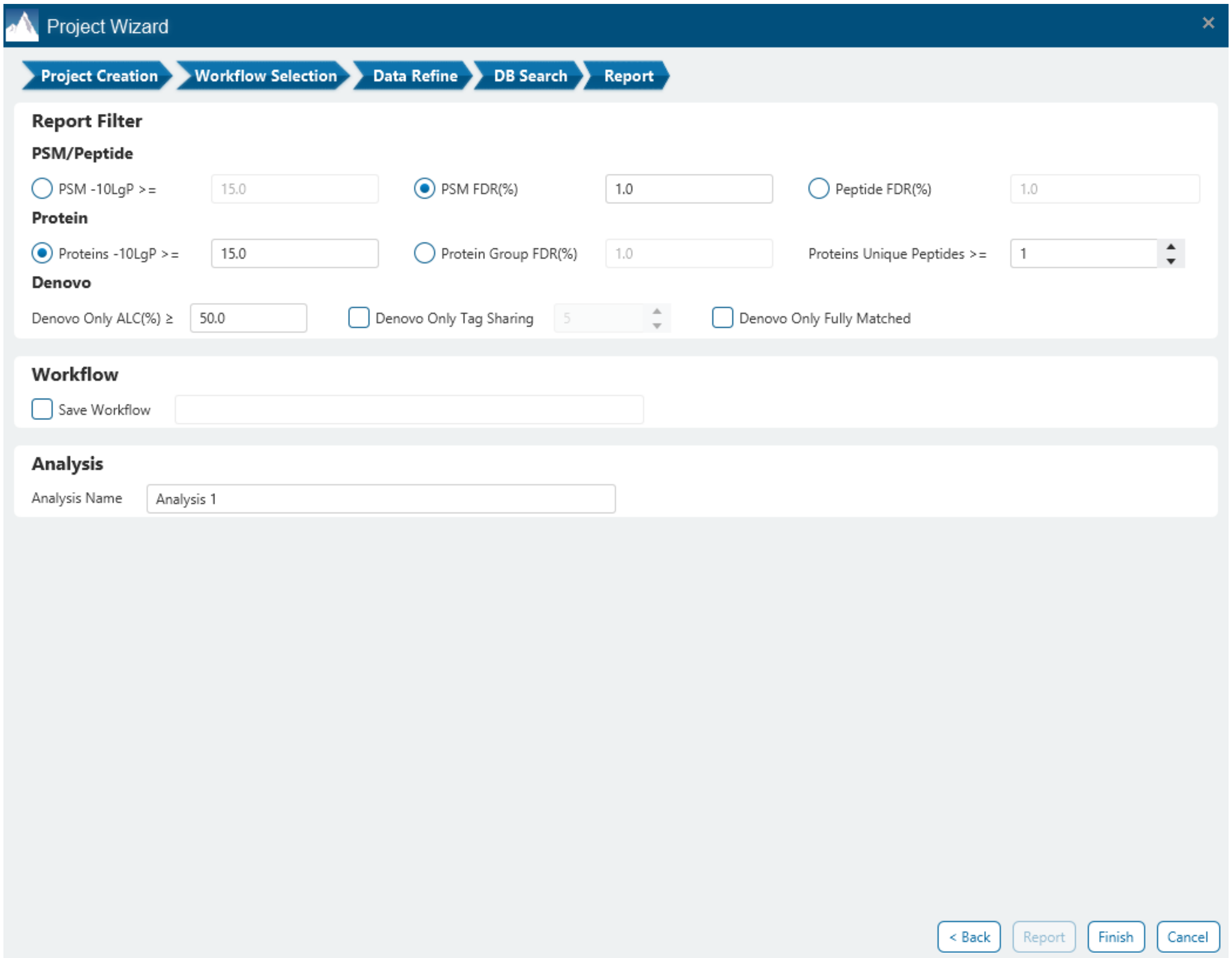

These filters are provided:

PSM/Peptide: this section is a mandatory section to filter out some results under the user-defined filter. Any changes to the filter will create a new analysis with old parameters and update the results and statistics.

PSM -10lgP: The PEAKS peptide score (-10lgP) is calculated for every peptide-spectrum match (PSM) reported by PEAKS DB, PEAKS PTM, and SPIDER. The score is derived from the p-value that indicates the statistical significance of the peptide-spectrum match. A peptide may be matched to many spectra, resulting in multiple PSMs. In that case, the peptide's score is calculated as the maximum among all PSM scores. For details of the scoring algorithm, please refer to the publication, *"PEAKS DB: De Novo sequencing assisted database search for sensitive and accurate peptide identification ", Mol Cell Proteomics, 2011 Dec 20.* A minimum requirement can be set and all identifications must pass this filter.

PSM FDR (%): The PSM filter can also be set to use the false discovery rate. FDR is estimated using a "decoy-fusion" approach.

Peptide FDR (%): The Peptides can also be set to use the false discovery rate. Peptides are scored based on their top PSM and using a “decoy-fusion” approach, the result is reported at that FDR.

Note: Decoy fusion is an enhanced target-decoy method for result validation with FDR. Decoy fusion appends a decoy sequence to each protein as the "negative control" for the search. See BSI's web tutorial (http://www.bioinfor.com/fdr-tutorial/) for more details.

De Novo ALC (%): This filter controls the minimum ALC score required for display in the de novo only result. A default of 50% is set.

Denovo Only Tag Sharing: The number set here corresponds to the length of de novo only tags to be shown in the result Protein coverage. For de novo only peptides that are not fully matched, they must share a tag length with of minimum of this parameter with the protein residue to be displayed.

Denovo Only Fully Matched: Checking this option will show the de novo only peptides in the Protein coverage regardless of what tag sharing parameter is set if the entire de novo only sequence matches the protein residue.

In the Report step, there also is the option to specify an Analysis Name (which can be later renamed) and/or decide to save the Workflow to quickly re-use in future analyses.

6.2 Understanding PEAKS DB Search Results

After a PEAKS Search is complete, all searches selected during the analysis creation will generate result nodes in Project View. One is the de novo sequencing result, which includes de novo peptide sequences listed, spectrum annotation and other detailed information. The other result nodes are identification results from the database search tools: PEAKS DB, PEAKS PTM, and SPIDER. If more than one database search tool is enabled, the results from previous search tools are automatically merged.

Double-click on the result node DB Search, PEAKS PTM, SPIDER to open their respective results. The analysis results for database search tools are presented in five tabs:

- **Summary:** This shows an outline of the PEAKS Studio database search results with key statistics. The overall quality of the experiment can be examined and the filters for peptide and protein identifications can be adjusted. This page shows after the search is done.
- **Proteins:** This shows a list of protein identifications. This view also visualizes protein sequence coverage and helps with protein characterization.

- **Peptide:** This shows a list of peptide identifications. This view also provides spectrum annotation and other detailed information for peptide precursor spectrum matches.
- **De novo only:** This shows a list of quality peptide sequences detected by de novo sequencing that remain unidentified by the PEAKS Studio database search.
- **Feature:** This shows a list of features with and without peptide identifications.

The following sections will explore each of these five tabs in the Identification result.

6.3 PEAKS DB Search result - Summary tab

The Summary view reports key statistics as an overview of the result. In the report, several statistical charts can be examined to assess result quality. At the top of each summary result page, protein filters can be set. Identifications that fall below the filters will not be visible in the result pages or exports.

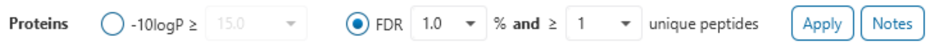

Proteins -10lgP: The PEAKS protein score (-10lgP) is calculated as the weighted sum of the -10lgP scores of the protein's supporting peptides. After removing any redundant peptides, the supporting peptides are sorted by -10lgP scores in descending order, and the k-th ranked peptide contributes to the weighted sum with a weight of 1/k. A default threshold of 15 is set.

Proteins FDR: The Proteins can also be filtered in accordance to the false-discovery rate of the protein set.

Proteins Unique Peptides: The minimum number of unique supporting peptides for a protein identification. A unique peptide is defined as a peptide that can be mapped to only one protein group.

After changing the filters, click 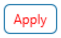

to apply the new filter and update the results accordingly.

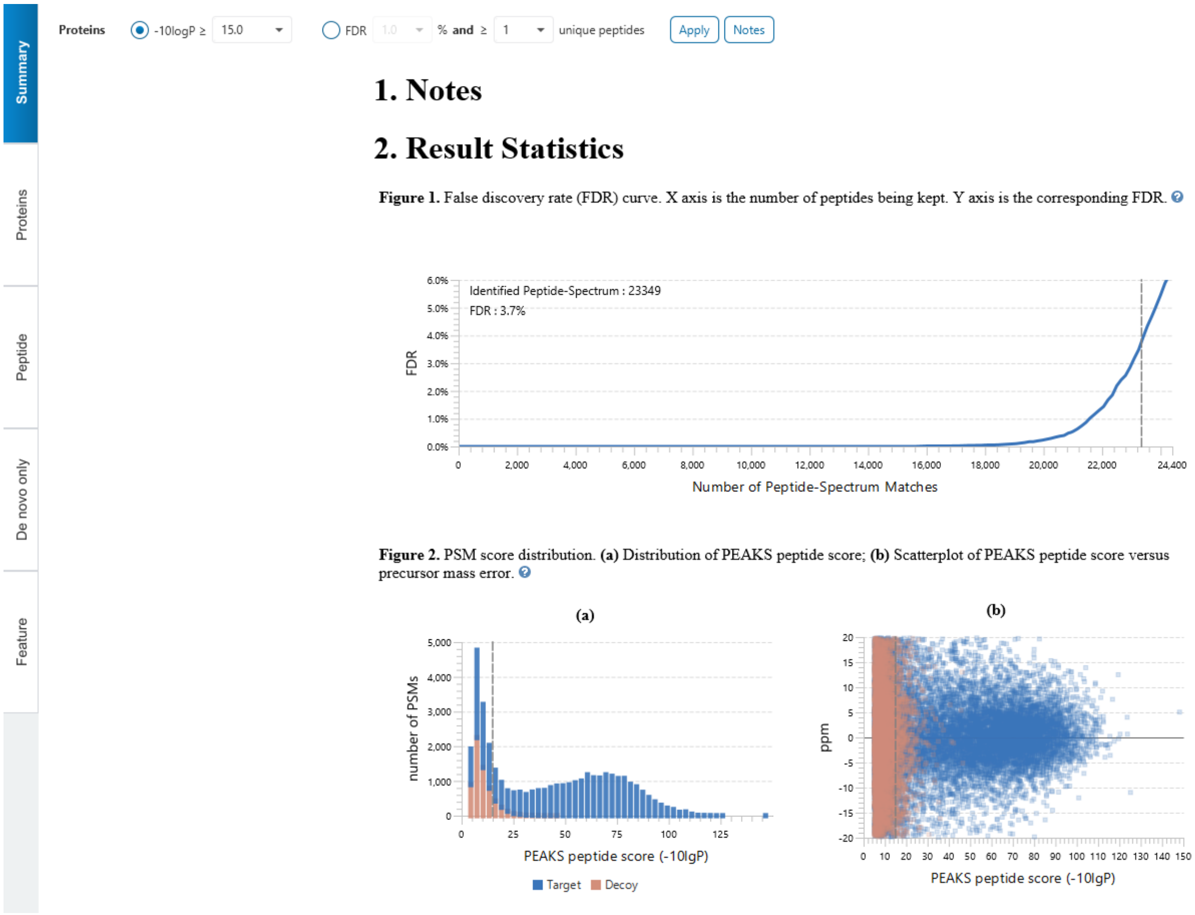

Click on Notes to open the Notes dialog to save useful information regarding this Analysis. The Notes will appear in Section 1 of the summary page at the top.

The Summary view reports key statistics as an overview of the result. In the report, several statistical charts can be examined to assess result quality.

6.3.1 PEAKS DB Search result - Figure 1. False Discovery Rate (FDR) curve.

Figure 1 shows the FDR curve with respect to the number of PSMs being kept after filtration by the peptide -10lgP score. By lowering the score threshold, more PSMs are kept in the filtered result. Conversely, the FDR increases because more false positives are kept. In this figure, the vertical line indicates the current score threshold. The number of PSMs and the corresponding FDR at the current score threshold are shown in the top-left corner. PEAKS Search estimates FDR using the "decoy-fusion" approach. Decoy-fusion is an enhanced target-decoy approach that makes more conservative FDR estimations. For details of the "decoy-fusion" approach, please referto the publication, *"PEAKS DB: De Novo sequencing assisted database search for sensitive and accurate peptideidentification", Mol Cell Proteomics, 2011 Dec 20.*

It is commonly recommended to set FDR at <1% by adjusting the score threshold. If a rapid growth of the FDR around the 1% FDR threshold is noticed, some PSMs may be sacrificed to significantly reduce the FDR.

Note: When counting the number of PSMs, PEAKS Studio keeps one peptide per spectrum at most. Thus, the number of PSMs is actually the number of spectra with assigned peptides.

Note: During Analysis creation, if Peptide FDR (%) filter was used in Report step, the Summary page FDR curve in the results will show the Peptide FDR curve and corresponding number of Identified peptides.

Note: Decoy matches are not counted in the number of PSMs. Unless otherwise specified, decoy matches are also excluded from the other statistical numbers shown in the Summary view.

6.3.2 PEAKS DB Search result - Figure 2. PSM score distribution

Figures 2(a) and 2(b) in the Summary view show the peptide score distribution of PSMs. When FDR estimation is enabled in the PEAKS Search parameters, the target and decoy matches are respectively shown in two different colors.

Figure 2(a) is a stacked histogram showing the number of target and decoy matches in each score interval. There should be a similar number of target and decoy matches with low scores, but very few decoy matches with high scores. The vertical line indicates the current score threshold.

Figure 2(b) is a scatter plot showing the peptide score versus the precursor mass error in ppm for all PSMs. This figure is most useful for high resolution instruments. The PSMs with high scores should be centered near the mass error of 0, and the PSMs with low scores are usually scattered to larger mass error. The vertical dotted line indicates the current score threshold.

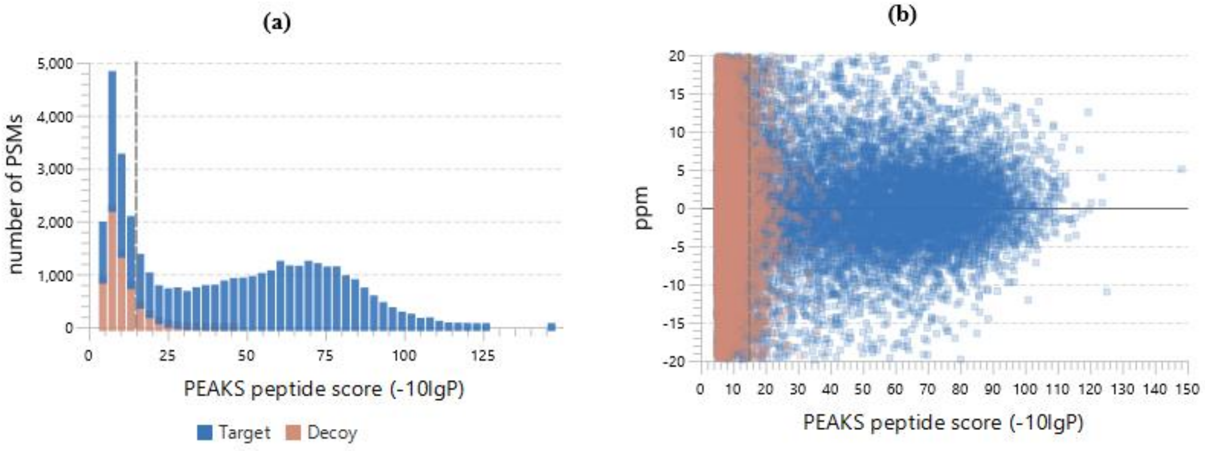

6.3.3 PEAKS DB Search result - Table 1. Statistics of data

Statistics of Data tabulates MS1 and MS2 information, as well as identification in the peptide and protein level. This table in the Summary view provides a condensed statistical information for the analysis.

Table 1. Statistics of data.

	#Scans			#Features	Identified			#Peptides	#Sequences	#Proteins*		
	MS1	MS/MS	#Chimera		#PSMs	#Scans	#Features**			Groups	All	Top
Total	23104	117254	101150	397739	56845	52923	43670	11450	10202	1184	1334	1220
SMA_1	3906	19343	17093	64673	9711	9000	7417	6735	6251	891	985	912
SMA_2	3799	19317	16581	63508	9500	8847	7289	6614	6121	876	985	897
SMA_3	3915	19445	16358	63352	9621	8970	7310	6662	6161	877	962	896
WT_1	3742	19403	16850	68969	9225	8587	7185	6354	5946	898	1005	924
WT_2	3950	19935	16746	67043	9392	8753	7204	6410	6005	923	1031	948
WT_3	3792	19811	17522	70194	9396	8766	7265	6457	6055	933	1043	961

* proteins with significant peptides are used in counts.
** features are identified by DB search only.

#MS1: Total # of MS1 spectra for each sample. For timsTOF data this shows the # MS1 frames.

#MS/MS: Total # of MS2 spectra for each sample. This does not include chimeric scans. For timsTOF data this shows the # MS2 frames.

#Chimera: The number of chimeric tandem scans in the sample.

#Features: Total # of MS1 features detected from each sample.

Identified #PSMs: The number of peptide-spectrum matches.

Identified #Scans: The number of tandem scans that have a peptide-spectrum associated with it.

Identified #Features: The number of features that have a PSM.

#Peptides: The unique number of peptide sequences with modifications not including I/L differentiation.

#Sequences: The unique number of peptide sequences not including modifications and I/L differentiation.

#Proteins Groups: PEAKS Studio groups proteins identified by a common set of peptides. This number shows the number of protein groups in the filtered result based on All proteins.

#All Proteins: Total number of proteins with significant peptides, according to current protein filters.

#Top Proteins: Total number of top proteins with significant peptides, according to current protein filters.

6.3.4 PEAKS DB Search result - Figure 3. Sample overlap for Proteins and Peptides

Figures 3(a), 3(b), and 3(c) overlaps between samples in the search for all proteins, top proteins, and peptides, respectively.

Note: Top proteins are supported by the most number of unique peptides in the protein group.

Note: Peptide sequences with I/L differences will only be counted once.

Sample overlaps are displayed as venn diagrams for 2-4 samples.

Figure 3. Sample overlap for Proteins and Peptides (up to 8 samples). **(a)** All Proteins; **(b)** Top Proteins; **(c)** Peptides;

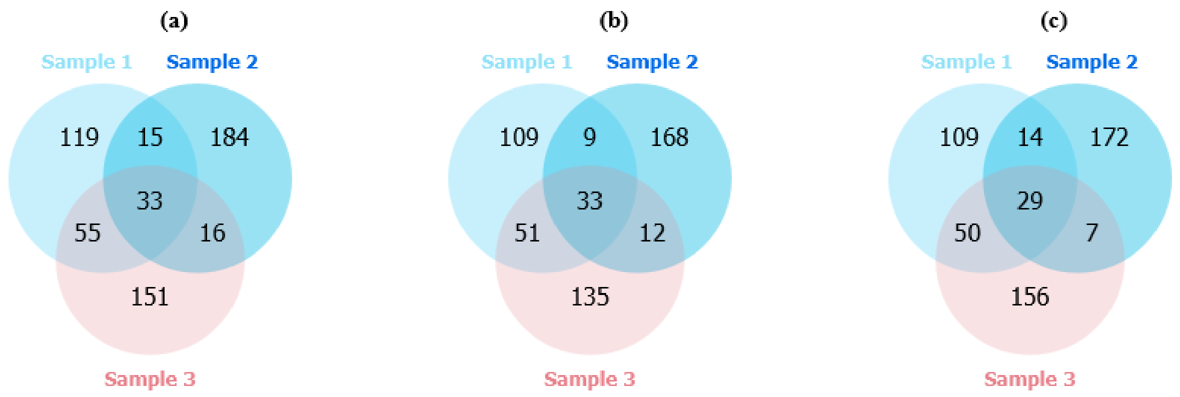

Sample overlaps are displayed as tables for 5-8 samples.

(a)

	1	2	3	4	5	6	Unique
1	1043	919	915	818	837	828	24
2	919	1031	907	832	841	834	22
3	915	907	1005	805	812	800	23
4	818	832	805	962	860	866	11
5	837	841	812	860	985	863	25
6	828	834	800	866	863	985	25

(b)

	1	2	3	4	5	6	Unique
1	961	844	841	765	768	775	22
2	844	948	830	776	768	779	22
3	841	830	924	753	745	750	21
4	765	776	753	896	796	807	10
5	768	768	745	796	897	802	21
6	775	779	750	807	802	912	16

(c)

	1	2	3	4	5	6	Unique
1	6457	5115	5062	4480	4496	4482	485
2	5115	6410	5022	4475	4472	4444	498
3	5062	5022	6354	4399	4439	4400	511
4	4480	4475	4399	6662	5246	5311	524
5	4496	4472	4439	5246	6614	5185	559
6	4482	4444	4400	5311	5185	6735	634

1 - SMA_1 2 - SMA_2 3 - SMA_3 4 - WT_1
5 - WT_2 6 - WT_3

Sample overlaps are not displayed for analysis with only 1 sample or more than 8 samples.

6.3.5 PEAKS DB Search result - Figures 4 and 5. Distribution of peptide feature detection

Figures 4(a), 4(b), and 5(a) in the Summary View show the distribution of peptide feature detection.

Figure 4(a) in the Summary View provides all detectable features distributed over the m/z range. This stacked histogram shows the number of detectable features in each m/z interval. In addition, the number of detectable features with database results are highlighted in orange and the number of detectable features with de novo results in addition to the database results are highlighted in light blue. In a typical dataset, it is often observed that a higher number of detectable features occur in the lower m/z range, which is reflected within the identification results.

(a)

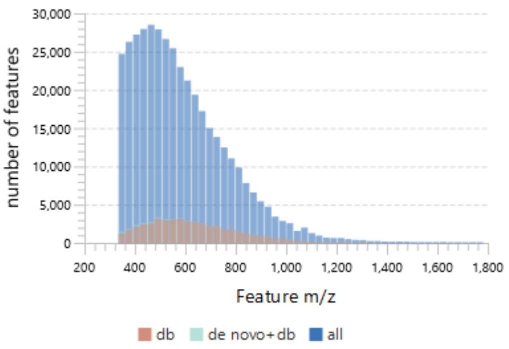

Figure 4(b) in the Summary View provides all detectable features distributed over the total retention time. This stacked histogram shows the number of detectable features in each RT interval. In addition, the number of detectable features with database results are highlighted in orange and the number of detectable features with database and de novo results are highlighted in light blue. In a typical dataset, it is often observed that a higher number of detectable features occur in the middle of the RT window, which aligns well with the identification results.

(b)

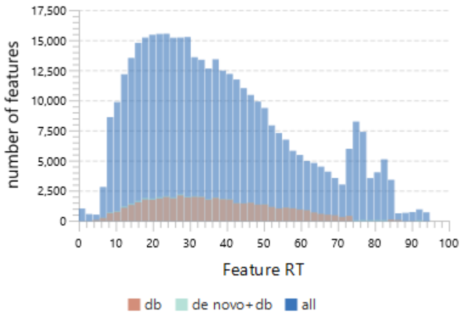

Figure 5(a) in the "Summary" view provides all detectable features distributed over the feature abundance measured as the area under the XIC curve. This stacked histogram shows the number of detectable features measured with a certain total abundance. In addition, the number of detectable features with database results are highlighted in orange and the number of detectable features with de novo results in addition to the database results are highlighted in light blue. In a typical dataset, it is often observed that more peptide features will be assigned a database ID when they exhibit a stronger LC-MS signal.

Figure 5. Distribution of identified peptide features. **(a)** Feature abundance distribution;

(a)

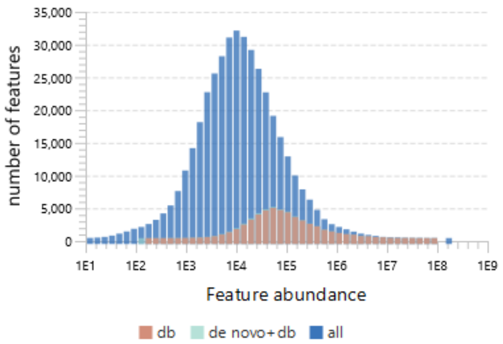

6.3.6 PEAKS DB Search result - Result filtration parameters

Tables 2-4 show the statistical numbers of the data and results.

Table 2. Result filtration parameters.

Peptide -10lgP	≥ 15
Protein -10lgP	≥ 15
Proteins unique peptides	≥ 1
De novo score(%)	≥ 50%

Table 3. Statistics of filtered result.

FDR (Peptide-Spectrum Matches)	60.1%
FDR (Peptide Sequences)	66.9%
FDR (Protein Group)	0.9%
De Novo Only Spectra	14425

Table 4. PTM profile.

Name	ΔMass	Position	#PSM	-10lgP	Abundance	AScore
13C6-15N4	10.01	R	142	80.83		
Deamidation	.98	NQ	122	52.59	1.35E6	
13C6-15N2	8.01	K	105	38.08	2.36E4	
13C(6)	6.02	R	44	48.94	1.46E7	
Carbamidomethyl	57.02	C	41	37.72	8.52E5	
Oxidation	15.99	M	33	80.83		
Lys4	4.03	K	11	18.66	3.28E6	

Table 2. Result filtration parameters: Table 2 summarizes the result filtration parameters set at the top of the Summary View page. This information provides a helpful reference for exported data.

Table 3. Statistics of filtered results: Table 3 presents the statistics based on the data analysis results after setting the desired filters. See below for further explanation:

- **FDR (Peptide-Spectrum Matches):** The total number of decoy database assignments to spectra relative to the total number of target database assignments to spectra represented as a percentage.
- **FDR (Peptide Sequences):** The total number of decoy database assignments to unique peptide sequences relative to the total number of target database assignments to unique peptide sequences represented as a percentage.
- **FDR (Protein Group):** The total number of decoy protein groups where the top hit in the protein group is a decoy database protein relative to the total number of target database protein groups where the top hit is from the target database.
- **De Novo Only Spectra:** Corresponds to the number of existing de novo sequencing results without a positive protein identification, given the filters set for the search results

Table 4. PTM Profile: shows modifications identified in the filtered result and the number of PSMs containing each modification

6.3.7 PEAKS DB Search result - Experiment Control

Figures 6(a) and 6(b) in the Summary View show the precursor mass errors of PSMs in filtered results. Figure 6(a) shows the distribution of the precursor mass errors in a histogram with and without software calibration. Figure 6(b) shows precursor m/z versus precursor mass error in a scatter plot after software calibration. The precursor mass error is calculated in ppm as $10^6 \times$ (precursor mass - peptide mass) / peptide mass. Calibration is for display purposes only and PEAKS corrects m/z value for each PSM to correct systematic biases and instrument measurement drift. Figures 5(a) and 5(b) help examine whether or not the mass spectrometer is properly calibrated. For a well-calibrated instrument, precursor mass errors should center at 0 ppm across the range of m/z.

Figure 6. Precursor mass error of peptide-spectrum matches (PSM) in filtered result. **(a)** Distribution of precursor mass error in ppm; **(b)** Scatterplot of precursor m/z versus precursor mass error in ppm.

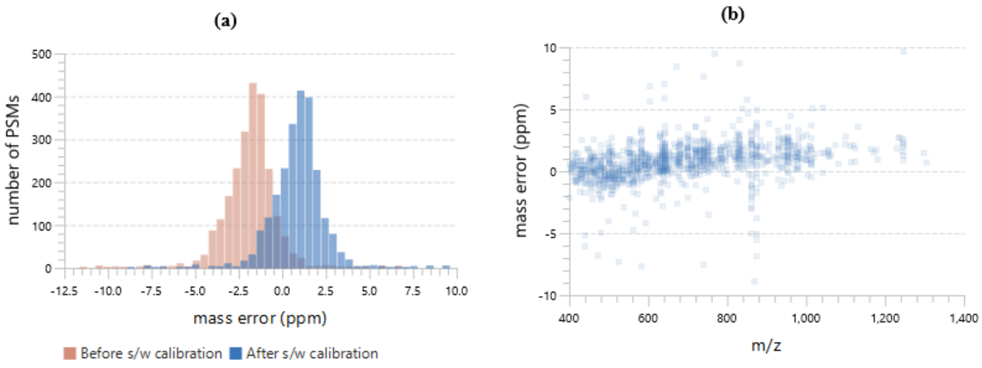

Table 5 shows the number of identified peptides in each sample with the number of missed cleavages, indicating the enzyme digestion efficiency.

Table 5. Number of identified peptides in each sample by the number of missed cleavages.

Missed Cleavages	0	1	2	3	4+
Sample 1	132	56	0	0	0
Sample 2	113	51	0	0	0
Sample 3	133	60	0	0	0

6.3.8 PEAKS DB Search result - Other Information

Table 6 summarizes the parameters defined by the user to analyze the data. This information includes error mass tolerances, missed cleavages, database(s) used, etc.

4. Other Information

Table 6. Search parameters.

Search Engine Name: PEAKS
Parent Mass Error Tolerance: 10.0 ppm
Fragment Mass Error Tolerance: 0.02 Da
Precursor Mass Search Type: monoisotopic
Enzyme: Specified by each sample
Max Missed Cleavages: 1
Digest Mode: Semi-Specific
Peptide Length Range: 6 - 45
Fixed Modifications:
  Carbamidomethylation (+57.02)
Variable Modifications:
  Deamidation (NQ) (+0.98)
  Oxidation (M) (+15.99)
Max Variable PTM Per Peptide: 2
Database: uniprot_sprot_June-2-2020.fasta
Taxon: Mammalia
Searched Entries: 67217
Deep Learning Boost: False
FDR Estimation: Enabled

6.4 PEAKS DB Search result - Proteins View

The Protein View lists protein identifications that have been filtered by the current settings in Summary View. It also visualizes the protein sequence coverage for identified proteins. The view has four main components:

- Protein table: This lists protein identifications that have been filtered by the current settings in the Summary View and the “Protein View Filters”.
- Coverage: This visualizes the protein sequence coverage of the selected protein, showing the mapping of support peptides and de novo tags to the protein sequence.
- Peptides: This lists supporting peptides mapped to the selected protein.
- De novo Tags: This lists "de novo only" peptides mapped to the selected protein.

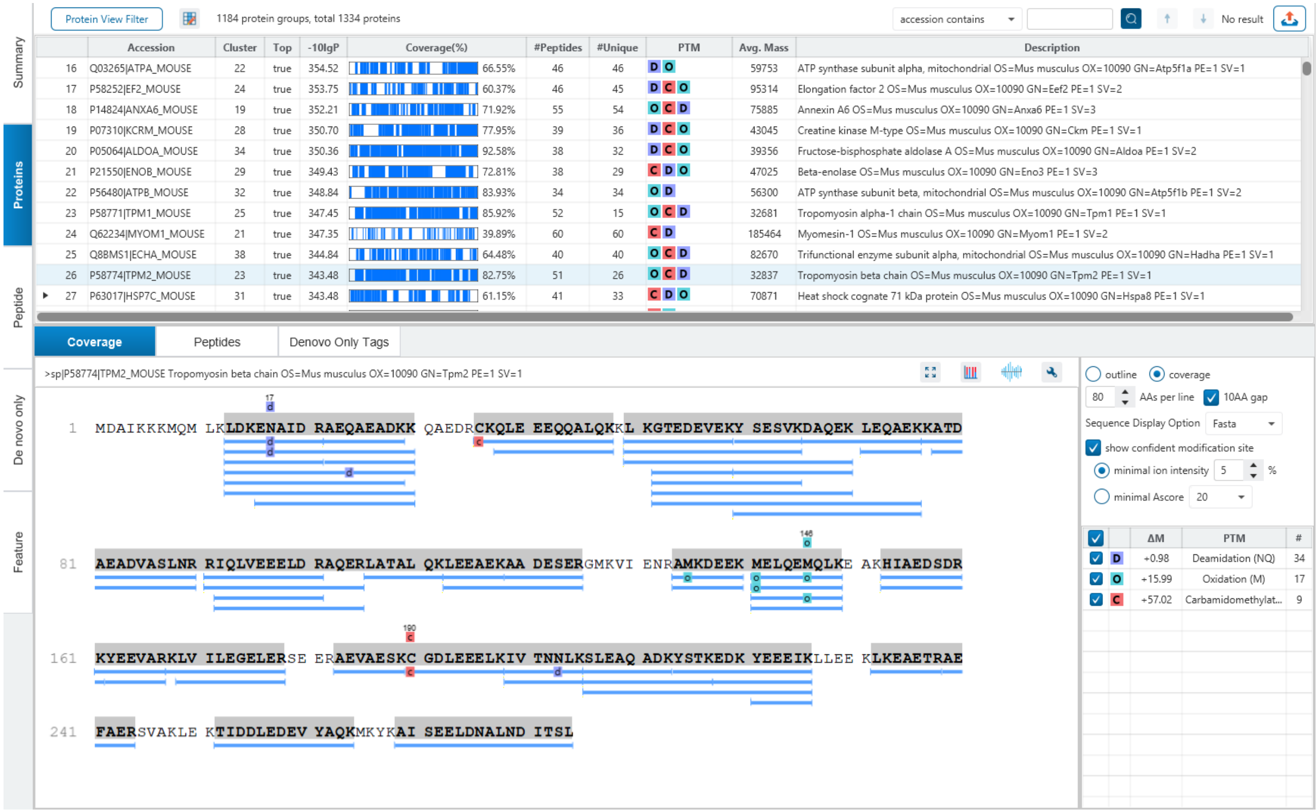

6.4.1 Proteins View - Protein Table controls

At the top of the Proteins View, there are the options to filter, show/hide columns, search, and export the protein table.

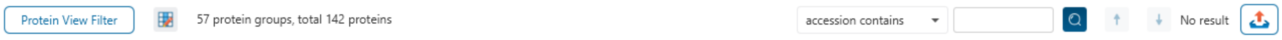

6.4.1.1 Protein Table controls - Protein View Filters

Click on the 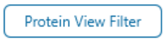

button to open the Protein View Filter.

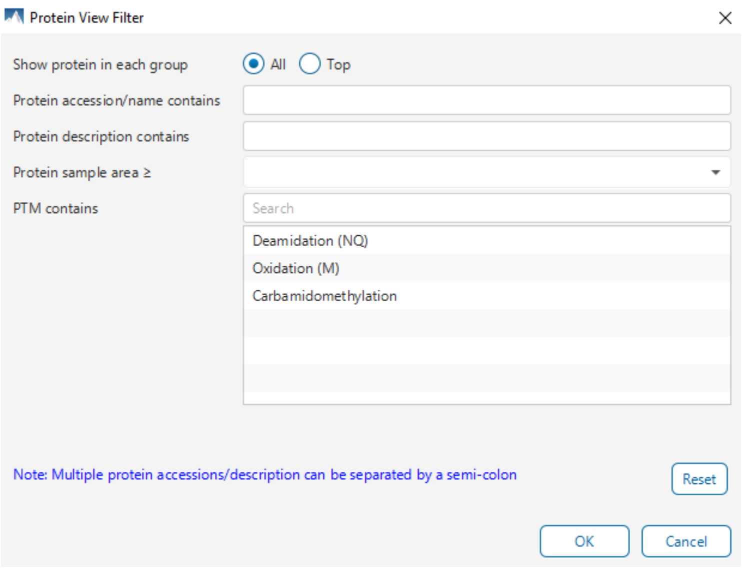

The following options are available for filtering the protein table:

Show Top, All, or First proteins in each group: Adjust the protein list based on Protein grouping, top is selected by default. Proteins are grouped based on parsimony.

- **Top:** Show only the top proteins in each group. These proteins are supported by the most unique peptides in the group. Proteins in the group that share a subset of the unique peptides that support the top protein (subproteins) will not be displayed.
- **All:** Show all proteins in each group. Proteins are grouped together if they are supported by the same set or a sub-set of the top protein in the group. If a protein is supported by a peptide not supporting the top protein it will be added to a new group.

Protein accession/name contains: Restrict the protein table to only display protein IDs which contain the entered text in its accession/name.

Protein description contains: Only proteins with descriptions that contain the entered text will be included in the protein table if text is entered in this field.

> **Note:** In these above two filters, multiple entries can be separated by a semi-colon.

Protein sample area: Filter proteins based on total protein area found in each sample. Sample areas are calculated using the total of all peptide features from unique supporting peptides. If a protein is identified and has areas from multiple samples, only one of the samples must pass this filter to be included.

PTM contains: Filter protein results by the presence of selected PTMs. Proteins that are supported by peptides containing modifications that match the selected PTMs will be included. If multiple PTMs match the modification name, they will all be included in the table. For SPIDER results, the mutations will also be listed in the PTM contains filter.

Click on the 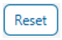

button to remove all filters.

Click on 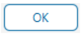

to apply the Protein View Filter to the Protein table.

Click on 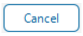

to discard changes and close the dialog.

6.4.1.2 Protein Table controls - Protein count

At the top of the protein table shows the count of protein groups and number of proteins in the table based on the current Protein View Filters.

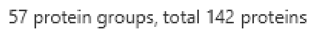

6.4.1.3 Protein Table controls - Optional columns

Click on the 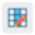 button to see a list of sample-based optional columns.

Select the corresponding sample-based Coverage, Spectra count, or Area to add into the Protein Table view.

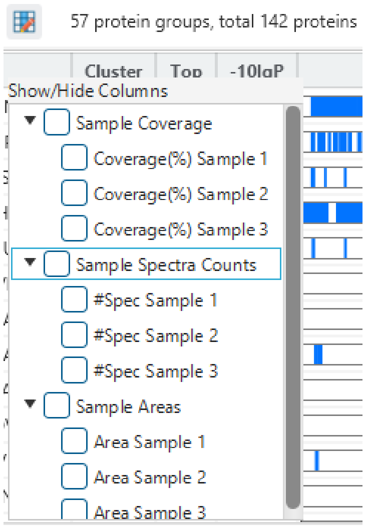

- **Coverage by Sample:** The protein coverage as a percentage will be displayed for the individual sample when selected.
- **#Spec by Sample:** The total number of spectra identified that support the given protein. Totals are given for each sample included in the search.
- **Area by Sample:** The total area of peptide features from unique supporting peptides in each sample will be displayed.

The exports will always include the contents of these optional columns even if they are hidden.

6.4.1.4 Protein Table controls - Protein Table Search Function

An easy-to-use search function is available for the protein table. The protein accession or description substring can be searched for, and all search results can be navigated through with the Up and Down arrows.

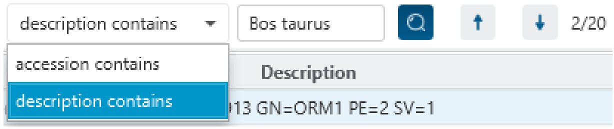

Protein accession/name contains: Restrict the protein table to only display protein IDs which contain the entered text in its accession/name.

Protein description contains: Only proteins with descriptions that contain the entered text will be included in the protein table if text is entered in this field.

6.4.1.5 Protein Table controls - Protein Table Export

Click on the button to export the contents of the current table. This considers the Protein View Filters, the sorting applied to the table and is consistent with the view. There is the option to export the Protein table and/or the Supporting peptide table in CSV format.

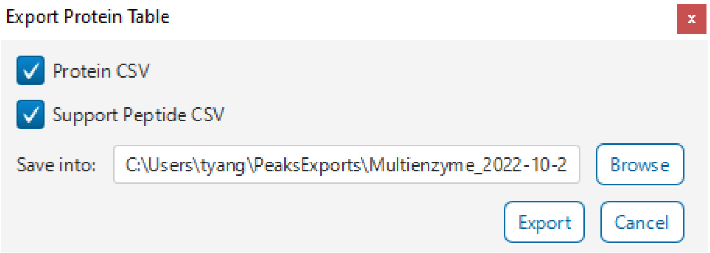

6.4.2 Proteins View - Protein Table

Each row in the table represents a group of proteins that are supported by a common set of peptides. To expand the group, click the right arrow button at the left.

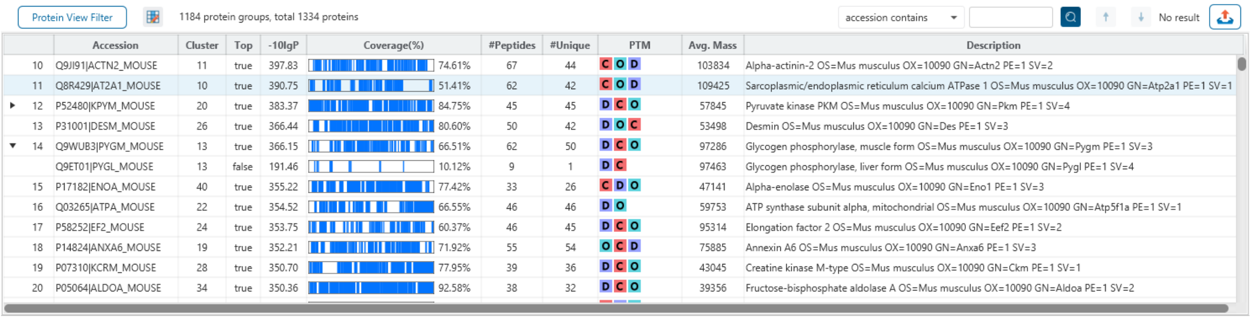

	Accession	Cluster	Top	-10lgP	Coverage(%)	#Peptides	#Unique	PTM	Avg. Mass	Description
10	Q9JI91|ACTN2_MOUSE	11	true	397.83	74.61%	67	44	C O D	103834	Alpha-actinin-2 OS=Mus musculus OX=10090 GN=Actn2 PE=1 SV=2
11	Q8R429|AT2A1_MOUSE	10	true	390.75	51.41%	62	42	C O D	109425	Sarcoplasmic/endoplasmic reticulum calcium ATPase 1 OS=Mus musculus OX=10090 GN=Atp2a1 PE=1 SV=1
▸ 12	P52480|KPYM_MOUSE	20	true	383.37	84.75%	45	45	D C O	57845	Pyruvate kinase PKM OS=Mus musculus OX=10090 GN=Pkm PE=1 SV=4
13	P31001|DESM_MOUSE	26	true	366.44	80.60%	50	42	D O C	53498	Desmin OS=Mus musculus OX=10090 GN=Des PE=1 SV=3
▾ 14	Q9WUB3|PYGM_MOUSE	13	true	366.15	66.51%	62	50	D C O	97286	Glycogen phosphorylase, muscle form OS=Mus musculus OX=10090 GN=Pygm PE=1 SV=3
	Q9ET01|PYGL_MOUSE	13	false	191.46	10.12%	9	1	D C	97463	Glycogen phosphorylase, liver form OS=Mus musculus OX=10090 GN=Pygl PE=1 SV=4
15	P17182|ENOA_MOUSE	40	true	355.22	77.42%	33	26	C D O	47141	Alpha-enolase OS=Mus musculus OX=10090 GN=Eno1 PE=1 SV=3
16	Q03265|ATPA_MOUSE	22	true	354.52	66.55%	46	46	D O	59753	ATP synthase subunit alpha, mitochondrial OS=Mus musculus OX=10090 GN=Atp5f1a PE=1 SV=1
17	P58252|EF2_MOUSE	24	true	353.75	60.37%	46	45	D C O	95314	Elongation factor 2 OS=Mus musculus OX=10090 GN=Eef2 PE=1 SV=2
18	P14824|ANXA6_MOUSE	19	true	352.21	71.92%	55	54	O C D	75885	Annexin A6 OS=Mus musculus OX=10090 GN=Anxa6 PE=1 SV=3
19	P07310|KCRM_MOUSE	28	true	350.70	77.95%	39	36	D C O	43045	Creatine kinase M-type OS=Mus musculus OX=10090 GN=Ckm PE=1 SV=1
20	P05064|ALDOA_MOUSE	34	true	350.36	92.58%	38	32	D C O	39356	Fructose-bisphosphate aldolase A OS=Mus musculus OX=10090 GN=Aldoa PE=1 SV=2

The columns in the Protein Table are:

- **Accession:** The accession number of the protein as seen in the FASTA database.
- **Cluster:** A unique identifier that classifies which protein group the protein belongs to.
- **Top:** Indicates whether this protein is a top protein.
- **-10lgP:** The protein confidence score.
- **Coverage (%):** The percentage of the protein sequence that is covered by supporting peptides. The coverage is visualized by a colored bar. Light blue blocks indicate the parts of the sequence covered by low-confidence peptides. Dark blue blocks indicate the parts covered by high-confidence peptides. This is the total coverage including results from all samples.
- **#Peptides:** The number of high-confidence supporting peptides.
- **#Unique:** The number of high-confidence supporting peptides that are mapped to only one protein group. Unique peptides with same sequence but different modifications are only counted once in this number.
- **PTM:** The identified modifications displayed with color-coded icons.
- **Avg. Mass:** The protein mass calculated using the average mass.
- **Description:** The protein's header information as seen in the FASTA database.

Note: For #Peptides and #Unique, two peptides with the same starting and ending positions in the protein are counted as one, regardless of their PTM forms. This is to follow the MCP (Molecular & Cellular Proteomics) guidelines.

6.4.3 Proteins View - Coverage Pane

The Protein Coverage view visually maps the supporting peptides and de novo tags to the protein selected in the Protein table. It also shows all identified sites with modifications or mutations to assist with protein characterization at the amino acid level. The "Protein Coverage" view is composed of three major components - Protein Sequence Coverage, Coverage Control Panel, and Protein Tools.

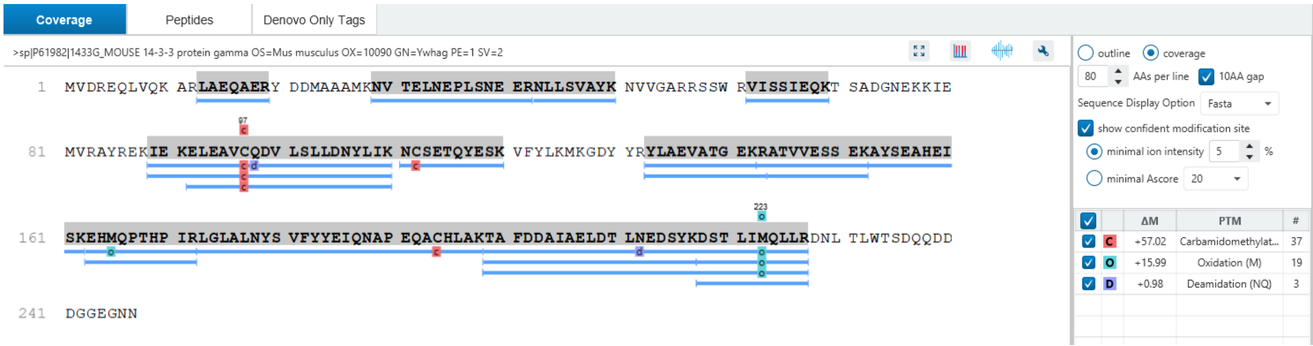

6.4.3.1 Protein Sequence Coverage

This area visualizes the coverage of the protein sequence.

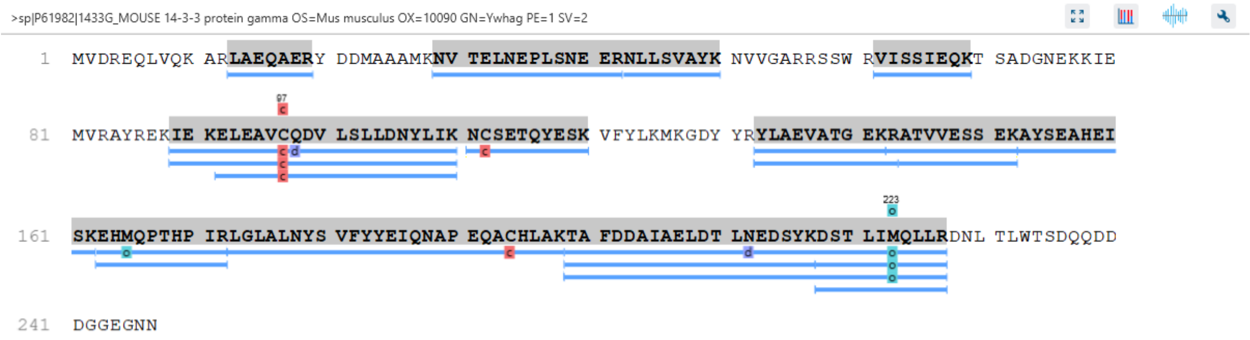

Regions in the protein sequence that are covered by supporting peptides are displayed in bold font with a grey background. Confident modifications and mutations identified in supporting peptides are displayed as icons above the protein sequence. Modifications are represented by colored icons with the first letter of its modification name. If a residue is modified by more than one modification in the same supporting peptide, "*" is used instead of a letter. Mutations are represented by framed white icons with a letter indicating the post-modification residue. Placing the cursor over a mutation icon shows the full name of the modification/mutation. The number above a mutation icon indicates the position in the protein sequence. The header information of the protein is shown on the top of the protein sequence.

The supporting peptides and matched de novo tags can be shown as colored bars under the protein sequence.

Placing the cursor over a bar shows a tooltip with detailed information of the peptide. Left clicking on a bar shows the annotated spectrum from which the supporting peptide is identified.

Right clicking on a peptide bar shows additional options. “Show in spectrum” has the same behaviour as left clicking on the peptide bar which is to open the annotated spectrum chart. “Show in table” has the function to jump to the supporting peptides table.

6.4.3.2 Coverage Control Panel

This panel controls what to display in the protein sequence coverage view.

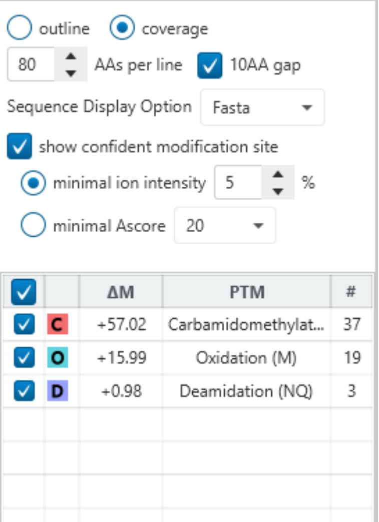

Sequence Display Mode: The protein sequence can be displayed in either "outline" or "coverage" mode.

Outline mode: In outline mode, the protein sequence is displayed without supporting peptides and matched de novo tags. However, the sequence coverage at a specific position can be examined by left-clicking a residue.

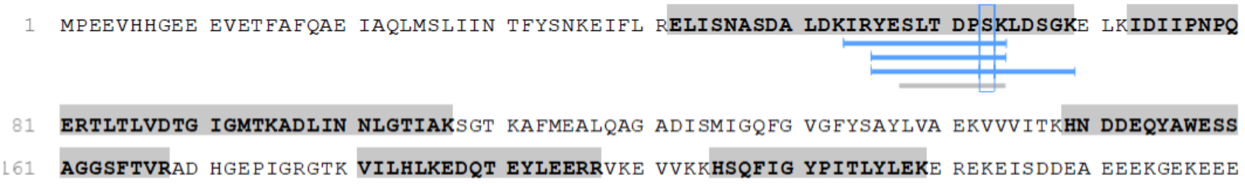

Coverage mode: In coverage mode, all the supporting peptides and matched de novo tags are shown.

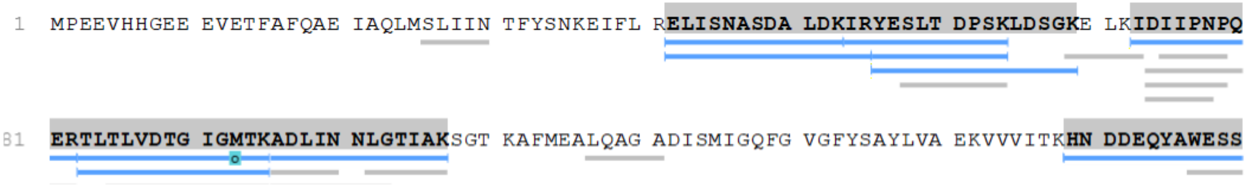

AAs per line and 10AA gap: Specify the number of amino acids per line and whether to show a gap for every 10 amino acids in the protein sequence.

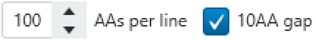

Sequence Display Option: Select whether to view the protein sequence as Fasta or a specific enzyme used in the project. When a particular enzyme is selected, the protein sequence will be highlighted to emphasize expected peptides generated by the selected enzyme's cleavage specificity.

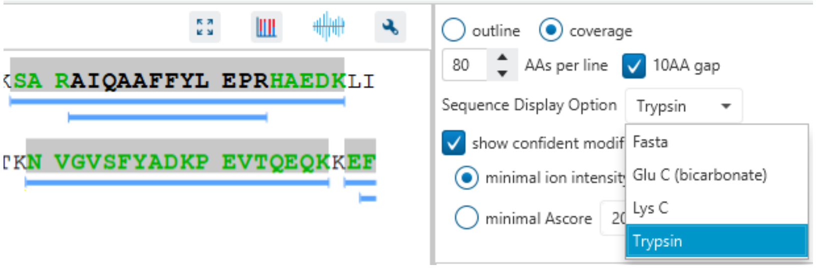

Modification minimal ion intensity / Ascore: A modification or mutation is considered confident if there are fragment ions supporting the modified residue with the minimal peak intensity above the specified threshold. Only confident modifications and mutations are displayed as icons above the protein sequence.

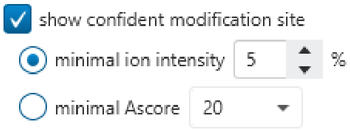

Modification table: The modification table shows the modifications identified in supporting peptides on the protein. For each modification, the number of supporting PSMs with this particular modification is shown. The checkbox on the left controls whether to indicate the modification in the protein coverage.

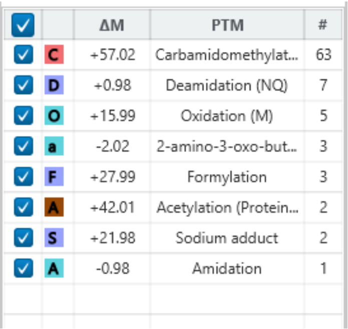

		ΔM	PTM	#
	C	+57.02	Carbamidomethylat...	63
	D	+0.98	Deamidation (NQ)	7
	O	+15.99	Oxidation (M)	5
	a	-2.02	2-amino-3-oxo-but...	3
	F	+27.99	Formylation	3
	A	+42.01	Acetylation (Protein...	2
	S	+21.98	Sodium adduct	2
	A	-0.98	Amidation	1

6.4.3.3 Proteins View - Protein Tools

The Proteins View toolbar is at the upper-right corner of the protein sequence coverage tab and contains helpful protein tools.

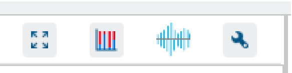

Full screen: When selected, the protein coverage window expands in full screen mode. Click to return from full screen mode.

Tool box: The tool box contains the following tools:

- Copy protein sequence. This copies the protein sequence of the current selected protein into the system clipboard.
- Save protein coverage as image. Different scale options are provided to increase the image size.
- Coverage statistics. This provides useful statistics based on the current selected protein.

PTM Profiling: Calculates the difference in abundance between peptides with confidently identified PTMs versus unmodified peptides. Peptide feature areas are used for this comparison.

For the Identification results (DB/PTM/SPIDER), the PTM Profiling tool provides quantitative information of modified peptides compared with unmodified peptides for the modification sites of the protein across all MS samples.

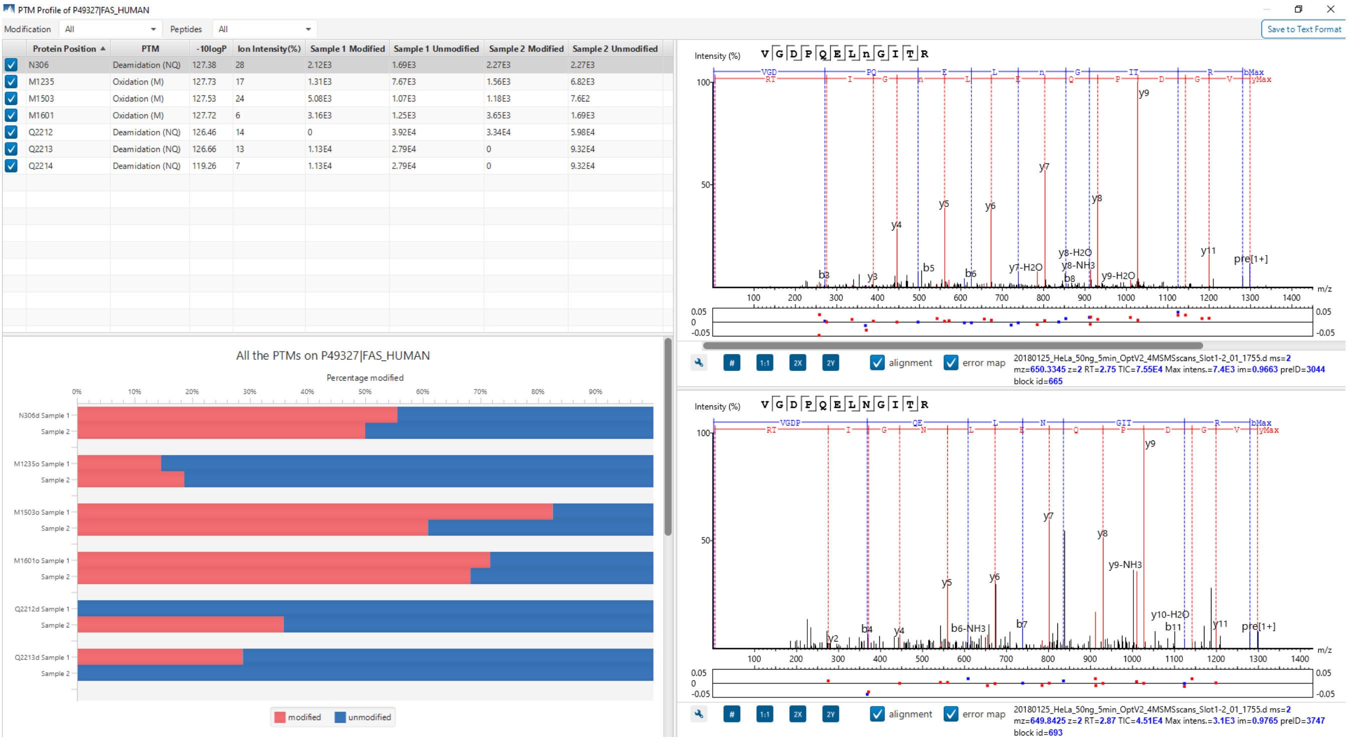

The inputs for the profiling can be set using the drop-down lists at the top-left corner of the dialog.

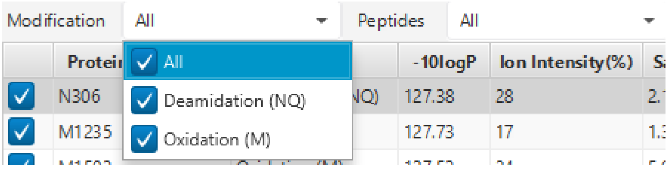

- **Proteins:** The accession ID of the selected protein to be analyzed for PTM profiling is shown in the title of the dialog. The selected protein can not be changed within the dialog. To run profiling on another protein, close the dialog, select the protein of interest from the protein table and run the profiling by clicking button.
- **Modification:** The drop-down list contains the detected and selected confident PTMs for the protein. Select the type of modification to study a specific PTM or choose “All the PTMs” to visualize the profiling information of all PTM sites at together.

  Note: The fixed PTMs are not used for profiling.

- **Peptides:** Data source used for the analysis. “All” considers all the peptides for this protein. When “Fully digested” is selected, only the full enzyme digested peptides will be used to calculate the modification abundance.

PTM Profile Table

The PTM Profile Table shows the detailed quantitative information for the modification sites. Each row in the table shows the modified and unmodified area for each sample of the selected PTM at each detected modification site. The table can be sorted by any column. The following list describes the contents in each column:

- **(Check box):** Allow the selection of each table row. Only the marked rows are used for the PTM Profile Chart.
- **Protein Position:** The detected modification sites in the protein. The modified amino acid symbol together with the position in the protein sequence is shown.
- **PTM:** The type of PTM being profiled.
- **-10logP:** The best -10logP identification score for the corresponding modified peptides.
- **AScore:** The best PTM AScore associated with the corresponding PTM at the specified site of the modified peptides.
- **Sample Modified:** Total area of the features associated with the corresponding modified peptides for each sample.
- **Sample Unmodified:** Total area of the features associated with the corresponding unmodified peptides for each sample.

	Protein Position	PTM	-10logP	Ion Intensity(%)	Sample 1 Modified	Sample 1 Unmodified	Sample 2 Modified	Sample 2 Unmodified
☑	N306	Deamidation (NQ)	127.38	28	2.12E3	1.69E3	2.27E3	2.27E3
☑	M1235	Oxidation (M)	127.73	17	1.31E3	7.67E3	1.56E3	6.82E3
☑	M1503	Oxidation (M)	127.53	24	5.08E3	1.07E3	1.18E3	7.6E2
☑	M1601	Oxidation (M)	127.72	6	3.16E3	1.25E3	3.65E3	1.69E3
☑	Q2212	Deamidation (NQ)	126.46	14	0	3.92E4	3.34E4	5.98E4
☑	Q2213	Deamidation (NQ)	126.66	13	1.13E4	2.79E4	0	9.32E4
☑	Q2214	Deamidation (NQ)	119.26	7	1.13E4	2.79E4	0	9.32E4

When “Fully digested” is selected, there are two more columns shown in the PTM Profile Table:

- **Peptide:** The fully digested peptide sequence.
- **Peptide Position:** Position of the modified amino acid in the peptide sequence.

PTM Profile Chart

The PTM Profile Chart is located at the bottom left corner in the dialog. It gives a visualization of the ratio between unmodified and modified amino peptides at each detected modification sites. The x-axis indicates the percentage (%) of modified or unmodified peptide feature areas relative to the total abundance whereas the y-axis indicates the position in the protein sequence where a modification was identified for each individual sample.

Note: Since the percentage is calculated over the sum of the modified and unmodified areas, the modified and unmodified portion always sum up to 100%.

Note: For samples with the specific PTM not detected, the corresponding bars are left as blank.

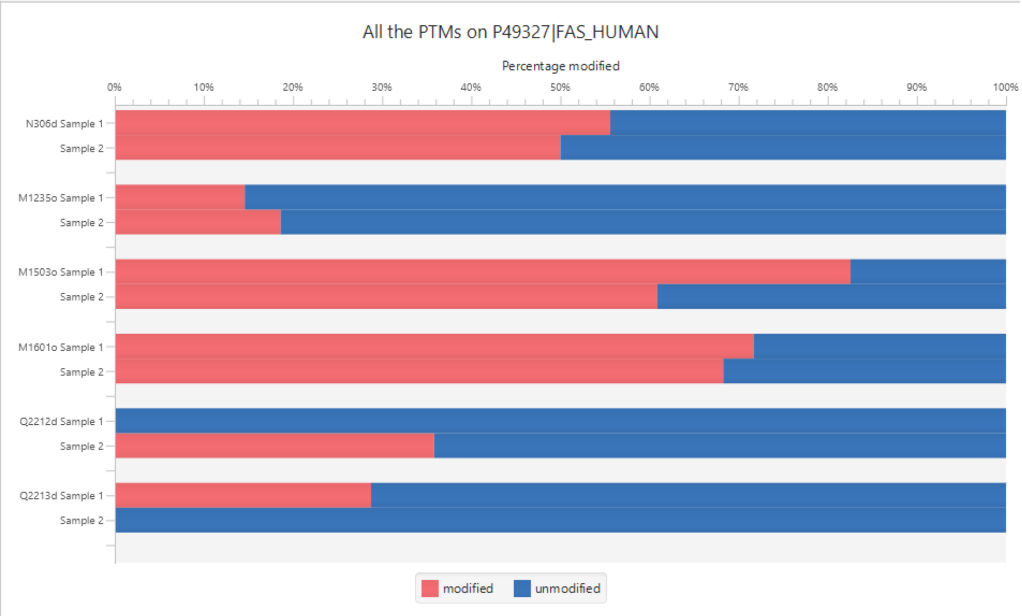

Spectrum Annotation View

The Spectrum Annotation View provides a graphical representation for the best modified peptide and the best unmodified peptide when "All" is selected from the "Peptides" drop-down list. The top spectrum corresponds to the best modified peptide, whereas the bottom spectrum shows the best unmodified peptide. When "Fully digested" is considered, the best modified peptide and the best unmodified peptide are displayed in a compare view for quick visualization and validation.

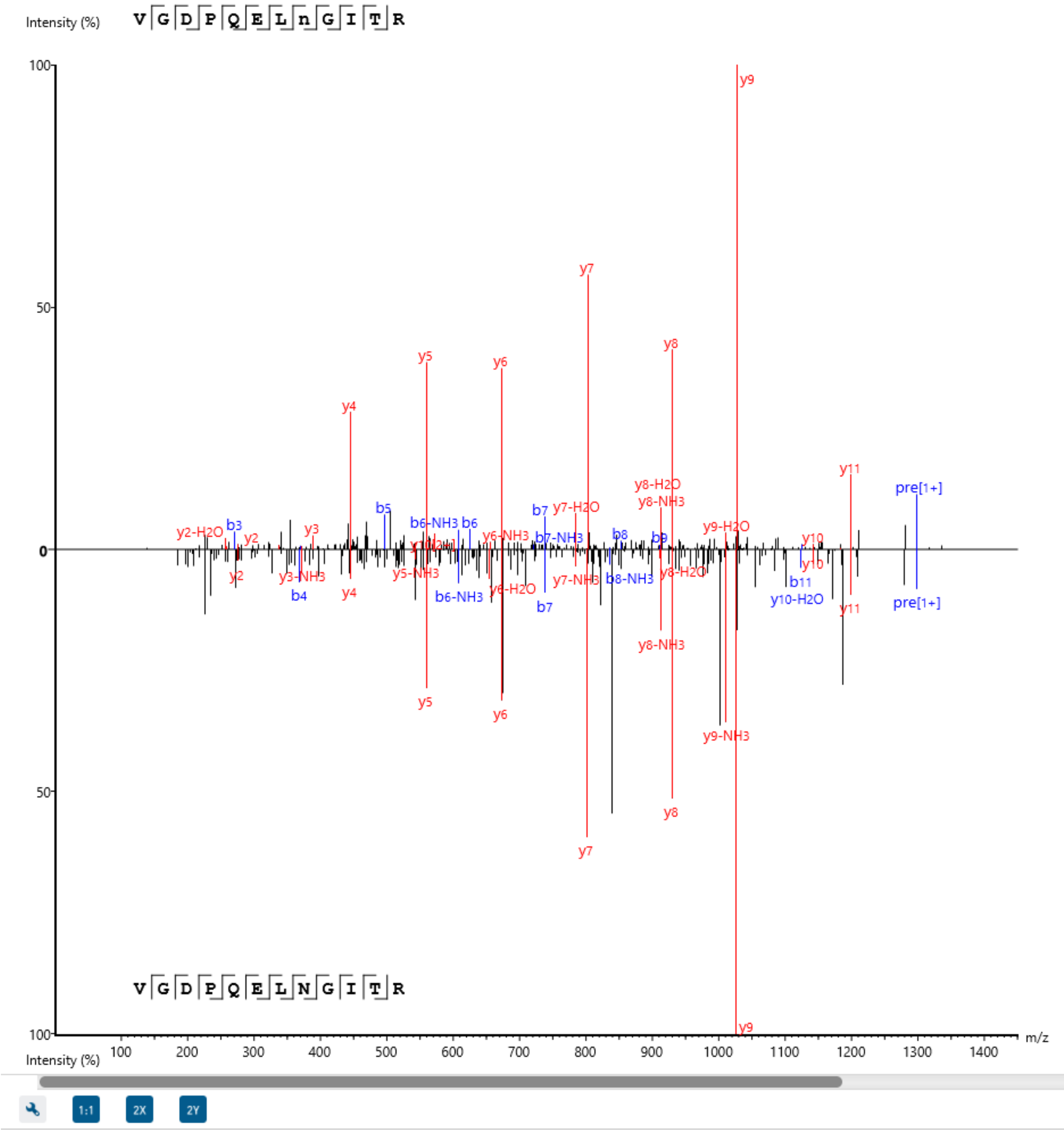

PTM Profile Export

The PTM profile can be exported as an image as well as in text format.

Image export: To save the profile chart, right click and choose Save to Image... menu item to save the displayed graph as a PNG file.

Text format export: Click [Save to Text Format] button at the top-right corner to export the calculated PTM Profile data to a text file in CSV format. The PTM Profile of the selected modification on the selected protein will be exported by default. In addition, profiles on all proteins for the selected modification may also be exported, if "Export ptm profile for all proteins" is checked. The exported file contains all necessary information to construct the PTM profile graph. The PTM profile data are grouped together by protein, then by modification type, and then by the modification site on the protein sequence. The headers of the CSV column are similar to the PTM Profile Table and also self-explanatory. The following items are explained for clarity:

- **Modified Peptide Sequence Window:** protein sequence containing 10 AA ahead of the modification site and 10 AA after the modification site with padding as necessary.
- **Sample Best AScore:** best AScore for each sample.

Note: When "Export PTM profile for all proteins" is not checked, only the profiling data that is marked in the PTM Profile Table will be exported.

Peptide Mapping:

A peptide map is often referred to as a “fingerprint” of a protein. It is a powerful tool that provides a comprehensive understanding of the protein being analyzed. It gives a detailed picture of the ion signal of all peptides associated with the protein. In addition to generating the peptide map, PEAKS has integrated an annotated tool-tip which allows for easy identification of the peptide shown at any point on the map. If multiple data files are searched together in the analysis, then multiple peptide maps may be compared to one another. This allows users to easily determine the consistency of the protein between samples.

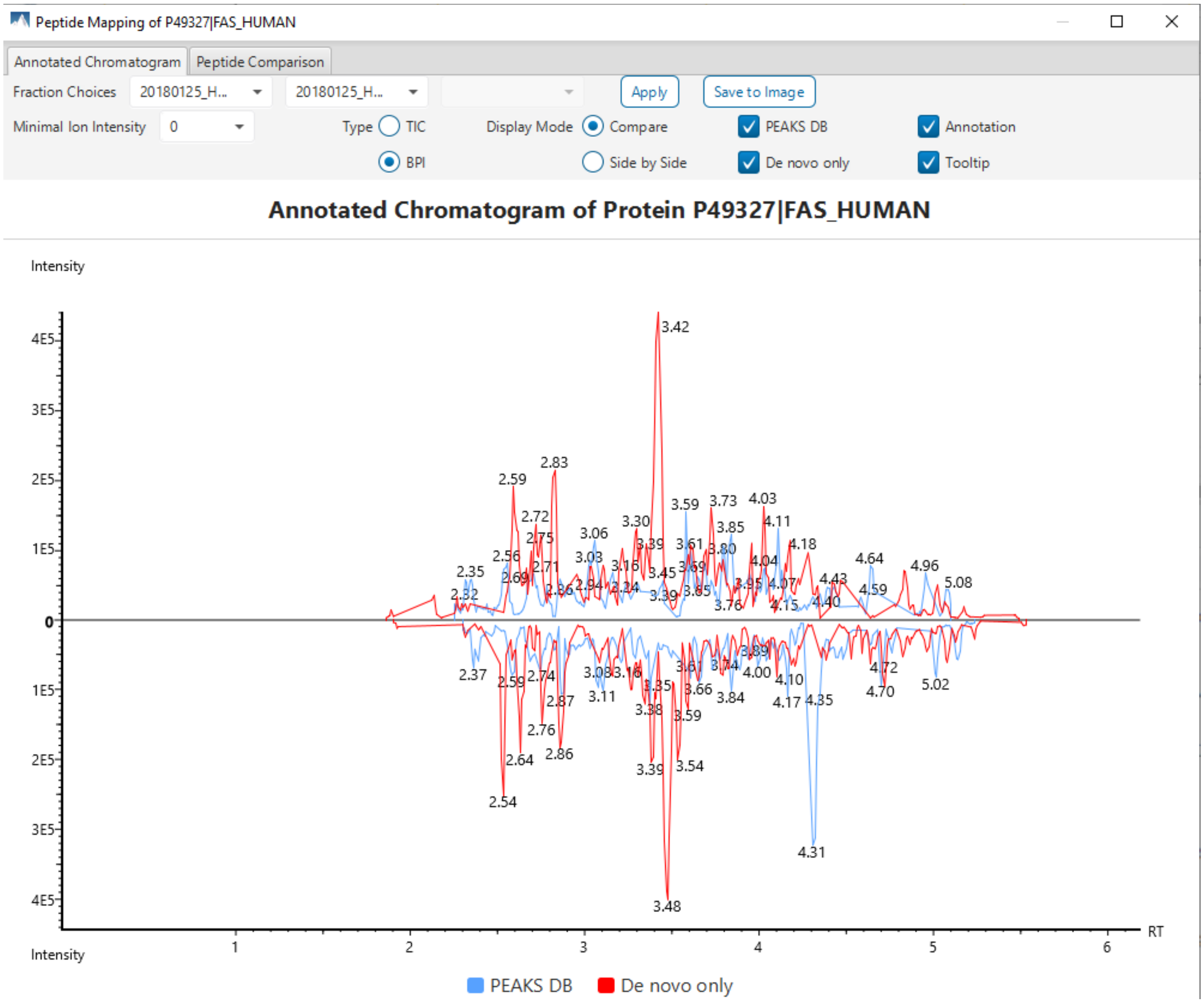

PEAKS provides two different approaches to analyze peptide mapping of a protein.

Annotated Chromatogram: The annotated chromatogram of the selected protein is displayed in profile mode. The intensity of each peptide feature associated to the protein is mapped to its retention time (RT). The annotated chromatogram has its own input selection, result display, and result exporting options.

- **Fraction Choices:** The data source used for peptide mapping. When the search result involves multiple fractions, a comparison of annotated chromatograms for the selected protein among (a maximum of three) fractions, can be done by selecting the second and third fraction from the corresponding drop down menu.
- **Minimal Ion Intensity:** A filter used to set an intensity threshold, where peptide features below this value will not be included in the display.
- **Type:** Configures the display to view the Total Ion Chromatogram (TIC) or Base Peak Intensity (BPI).
- **Display Mode:** PEAKS offers two modes to view multiple chromatograms for better comparison.
    - **Compare:** In compare mode, the protein chromatograms for the specified data samples will appear on opposite sides of the x-axis. This allows the user to directly compare the protein chromatograms between two samples.
    - **Side by Side:** In Side-by-Side mode, the chromatograms are displayed individually; each with their own set of x- and y-axes. This display mode allows for the comparison of up to three samples.
- **PEAKS DB:** Peptide features associated with peptides identified by database searching are highlighted in the annotated chromatogram.
- **De novo only:** This highlights the peptide features associated with de novo only peptides.
- **Annotation:** When selected, the retention time of major peptide features are displayed on the peptide map.
- **Tool Tip:** When selected, a pop-up will appear when the cursor hovers over any peptide within the peptide map. The pop-up contains the retention time, the intensity (TIC or base peak), and the peptide sequences found at that particular retention time.

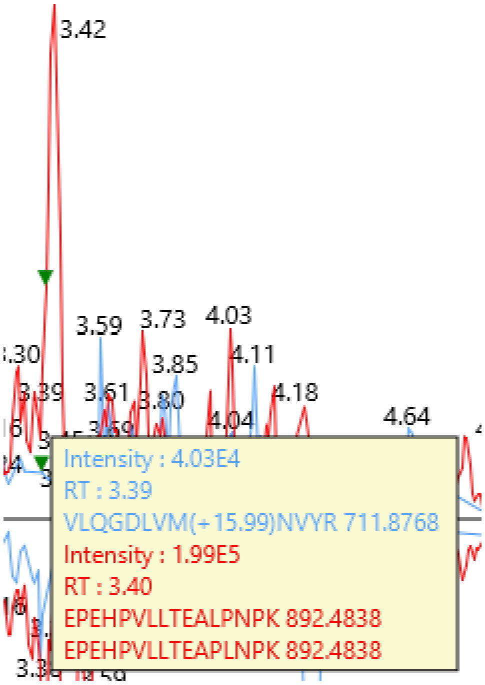

- **Annotated Chromatogram Chart controls:** This chart gives a visualization of the peptide map for the selected data. It provides a few convenient ways to zoom-in and navigate the chromatogram.

- Zoom to an RT region - Click the desired starting retention time, drag horizontally to the desired final retention time, and then release the mouse button.
- Zoom in/out smoothly - Place the cursor at a particular RT value (right below the x-axis line), scroll the mouse wheel button.
- Increase/decrease the ion intensity - Place the cursor on the chromatogram.
- See the whole chromatogram - Double-click on the chromatogram.
- Pan to an RT region: Place the cursor on the chromatogram, right-click, and then hold and drag to pan to an RT region.

- Result Export: The annotated chromatogram chart can be exported as an image in PNG format. Click the [Save to Image] button to save the displayed chart.

Peptide Comparison: Another option PEAKS offers its users to analyze the peptide map is in terms of a peptide comparison view. In this view, the total area of the features associated with a peptide is mapped to the mass of the peptide. The peptide comparison has its own input selection, result display, and result exporting options.

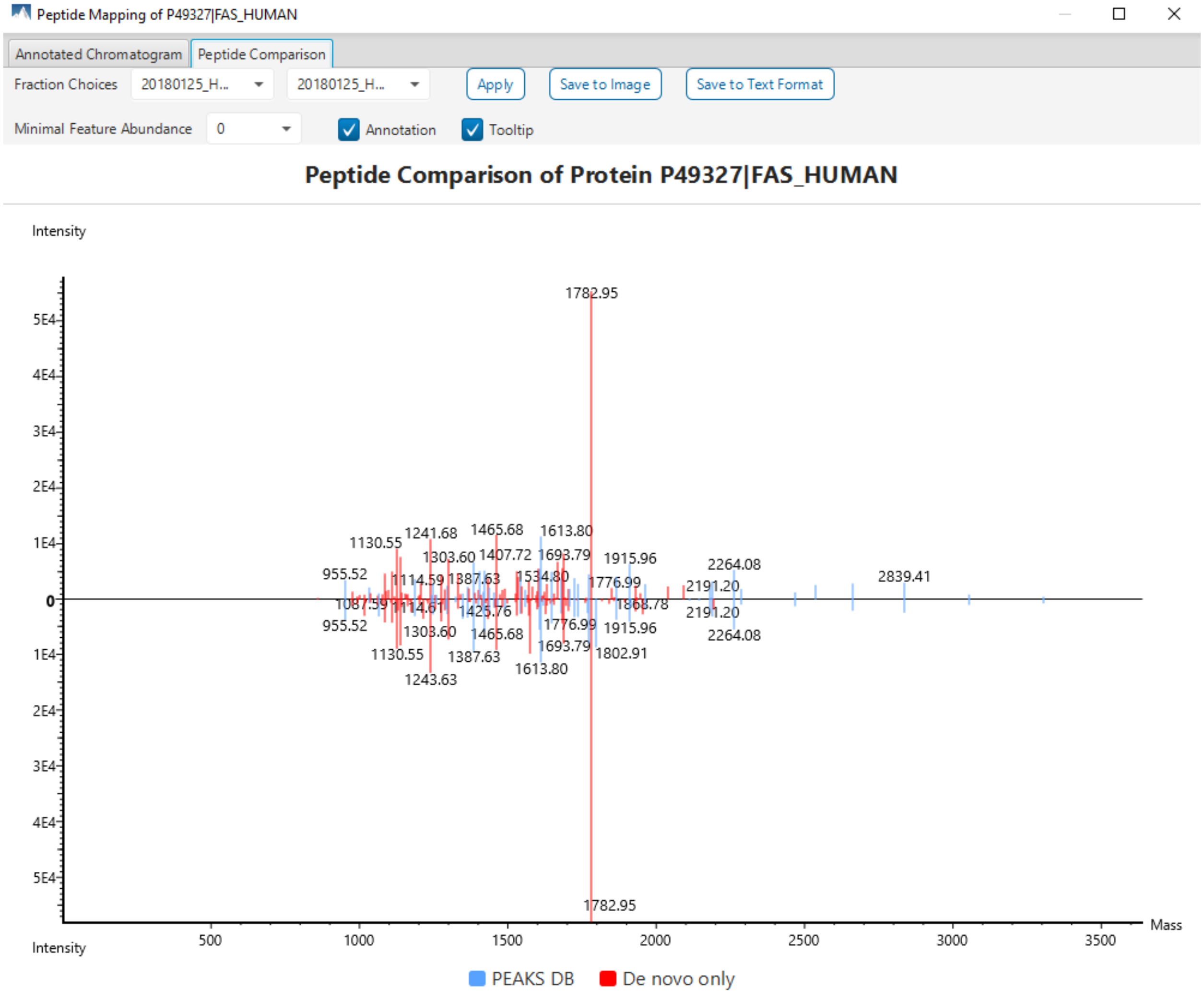

Inputs: The inputs for the peptide comparison can be set using the drop-down menus at the top-left corner of the dialogue.

- **Fraction Choices:** The data source used for peptide mapping. Peptide comparison allows a maximum of two fractions as the source. After making choices for the fraction selection, click the Apply button to update the chart.
- **Minimal Feature Abundance:** A filter used to set a feature area threshold, where peptide features below this value will not be included in the display.
- **Annotation:** When selected, the masses of the major peptides are displayed on the comparison chart.
- **Tool Tip:** When selected, a pop-up will appear when the cursor hovers over any peptide within the comparison chart. The pop-up contains the peptide mass, the total area for that peptide, and the peptide sequence.

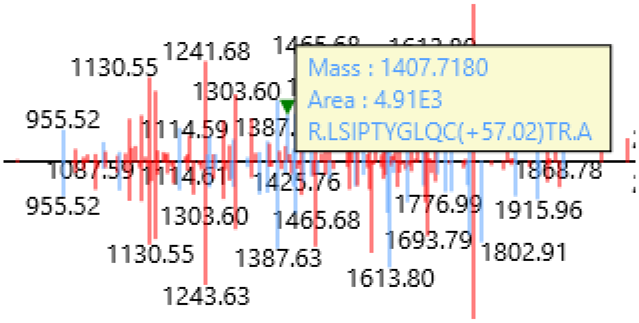

- **Peptide Comparison Chart:** This chart gives a visualization of the peptide comparison for the selected data. The chart is displayed in Peak Mode, where each peptide is represented as a bar and the height of that bar represents the total area of the associated peptide features in the specified fraction. The chart provides similar convenient ways to zoom-in and navigate the comparison chart.
- **Result Export:** The peptide comparison of a protein can be exported as an image, as well as in text format.
  - **Image export:** Click the Save to Image button to save the displayed graph as a PNG file.
  - **Text format export:** Click the Save to Text Format button to export the peptide comparison data to a text file in CSV format. The exported file contains all of the necessary information to construct the peptide comparison chart. The data are grouped together by peptide sequence and mass. The information for the same peptide are combined together if they belong to the same group, as mentioned earlier. The headers of the CSV column are self-explanatory. The following items are explained for clarity:
    - **Unique:** This specifies that the peptide is unique (Y) or not unique (N).
    - **-10lgP or ALC:** The significance score of the peptide identified by database searching or by de novo sequencing for de novo only peptides.
    - **Mass:** The mass of the peptide.
    - **ppm:** The error tolerance of the peptide.
    - **Feature Abundance:** This list of areas of the features associated with the corresponding peptide. The format of the feature areas is (area [space] +charge [semi-colon])*. For example, 500 +2; 800 +2; 700 +3
    - **Total Abundance:** The sum of the areas of the features associated with the corresponding peptide in the specified fraction.
    - **Ratio:** When the data sources contain two fractions, the ratio of the total areas of the corresponding peptide between two fractions is calculated.

Note: -10lgP, ALC(%), and ppm values are from the best PSM of the corresponding peptide.

6.4.3.4 Proteins View - Supporting Peptides

The "Peptides" tab displays a table of supporting peptides for the protein of interest. This table is similar to the peptide table in the **Peptide** view, except that "Accession" is excluded and the columns "(Checkbox)", "Unique", "Start", and "End" are included. Descriptions of extra columns are given in the following sections.

- **(Checkbox):** This controls whether a peptide is shown in the protein coverage as a blue bar.
- **Unique:** This shows whether the peptide is a unique supporting peptide to the protein group.
- **Start:** This shows the peptide's starting position in the protein.
- **End:** This shows the peptide's ending position (inclusive) in the protein.

The supporting peptides with peptide -10lgP score below the peptide filtering threshold are listed in the table as well. By default, they are shown in grey and are not displayed in the protein coverage.

Coverage | Peptides | Denovo Only Tags

	Peptide	Unique	-10lgP	Mass	Length	ppm	m/z	RT	Area Sample 1	Area Sample 2	Area Sample 3	Scan	#Feature	Start	End	PTM	AScore	Found By
1	K.DFSALESQLQDTQELLQEENRQK.L	true	114.84	2748.3202	23	-0.9	917.1132	253.93	3.57e+05	0.00e+00	0.00e+00	92174	1	1302	1324			DB Search
2	K.TQ(+0.98)LEELEDELQATEDAK.L	false	109.61	1961.8953	17	2.0	981.9568	236.33	0.00e+00	0.00e+00	0.00e+00	85431	0	1539	1555	D	Q3 D 61.14	DB Search
3	K.KLEEEQIILEDQNC(+57.02)K.L	true	104.07	1887.9248	15	1.0	630.3162	61.23	0.00e+00	2.45e+05	0.00e+00	23296	1	975	989	C		DB Search
4	K.LQVELDNVTGLLSQSDSK.S	true	103.89	1945.0004	18	3.3	973.5107	114.77	0.00e+00	4.31e+05	3.29e+05	44906	2	1278	1295			DB Search
5	K.TQLEELEDELQATEDAK.L	false	102.64	1960.9113	17	2.0	981.4648	110.64	5.40e+05	1.56e+06	5.39e+05	43203	3	1539	1555			DB Search
6	K.M(+15.99)QQNIQELEEQLEEEESA...	true	102.48	2604.1973	21	0.1	869.0731	91.75	0.00e+00	0.00e+00	1.29e+05	35296	1	941	961	O	M2 O 1000.00	DB Search
7	K.QTLENERGELANEVK.V	true	101.44	1728.8642	15	0.7	577.2958	49.96	0.00e+00	0.00e+00	7.03e+04	18355	1	1220	1234			DB Search
8	K.ALSLARALEEAMEQK.A	true	101.41	1658.8661	15	0.5	553.9630	117.43	1.67e+06	8.74e+05	3.65e+05	45911	3	1478	1492			DB Search
9	K.NMDPLNDNIATLLHQSSDK.F	true	101.18	2125.0110	19	0.9	709.3449	107.13	0.00e+00	0.00e+00	0.00e+00	41432	0	588	606			DB Search
10	K.RALEQQVEEMK.T	true	100.85	1359.6816	11	2.2	454.2355	42.34	0.00e+00	0.00e+00	3.18e+04	15320	1	1528	1538			DB Search
11	K.KFDQLLAEEK.T	false	100.40	1219.6448	10	1.0	407.5559	53.41	0.00e+00	1.14e+05	0.00e+00	20117	1	1445	1454			DB Search
12	K.TELEDTLDSTAAQQELRSK.R	true	100.29	2134.0389	19	1.8	712.3549	73.50	0.00e+00	0.00e+00	1.16e+05	27931	1	1146	1164			DB Search
13	K.LLEDRIAEFTTNLTEEEEK.S	true	99.00	2279.1168	19	1.1	760.7137	111.66	3.49e+05	0.00e+00	1.55e+06	43237	2	996	1014			DB Search
14	K.HSQAVEELAEQLEQTK.R	true	98.38	1838.9010	16	1.0	613.9749	86.02	0.00e+00	0.00e+00	3.64e+05	32937	1	1194	1209			DB Search
15	K.IAQLEEQLDNETK.E	true	95.17	1529.7573	13	1.1	765.8868	61.83	0.00e+00	1.88e+06	1.05e+06	23541	2	1816	1828			DB Search
16	K.EERTFHIFYYLLSGAGEHLK.T	true	85.81	2409.2117	20	0.7	603.3106	124.47	0.00e+00	0.00e+00	4.55e+04	48351	1	270	289			DB Search
17	K.TLEEEAK.T	true	68.58	818.4021	7	-6.3	410.2057	16.17	0.00e+00	2.55e+05	1.63e+05	5074	2	1175	1181			DB Search
18	K.LEGDSTDLSDQIAELQAQIAELK.M	true	58.88	2486.2387	23	1.5	1244.1285	259.24	2.79e+05	0.00e+00	0.00e+00	94260	1	1053	1075			DB Search
19	K.LLEDRIAEFTTNLTEEEEK(+8.01).S	true	52.58	2287.1310	19	1.4	763.3854	111.63	0.00e+00	3.24e+06	1.75e+06	43192	2	996	1014	C	K20 C 1000.00	DB Search
20	K.DFSALESQLQDTQELLQEENRQK(+...	true	49.54	2756.3344	23	2.6	919.7878	126.50	0.00e+00	3.72e+06	1.99e+06	49603	2	1302	1324	C	K24 C 1000.00	DB Search
21	K.ALSLARALEEAM(+15.99)EQK.A	true	45.41	1674.8610	15	1.0	559.2949	90.96	0.00e+00	0.00e+00	1.10e+05	34958	1	1478	1492	O	M13 O 1000.00	DB Search

6.4.3.5 Proteins View - De novo Tags

The "Denovo Only Tags" tab shows a table of "de novo only" peptides with sequence tags matched to the protein.

Coverage | Peptides | Denovo Only Tags

	Feature	Scan	Peptide	ALC(%)	Length	m/z	z	RT	Area	Mass	ppm	PTM	Mode	Source File
1	35412	89365	LTGMAFRVPTANVSVVDLTC(+57.02)RLEK	99.3	24	670.1096	4	246.70	2.86e+06	2676.4092	0.1	C	HCD	20161129_LUMOS1_nLC13_AH_long_Bench_HeLaYeast_p...
2	2127	17659	VLPELDGK(+4.03)	99.1	8	437.7633	2	47.53	1.38e+06	873.5109	1.3	D	HCD	20171106_LUMOS1_nLC13_AH_TechBench2_SILACH_1.raw
3	2242	17302	VLPELDGK(+8.01)	93.4	8	439.7578	2	47.32	4.40e+05	877.5000	1.3	C	HCD	20171106_LUMOS1_nLC13_AH_TechBench2_SILACL_1.raw
4	2088	16117	LVPELNGK(+4.03)	92.1	8	437.2711	2	44.38	5.02e+04	872.5269	0.9	D	HCD	20171106_LUMOS1_nLC13_AH_TechBench2_SILACL_1.raw
5	2226	17664	VLPELDGK(+8.01)	91.4	8	439.7576	2	47.58	1.60e+06	877.5000	0.8	C	HCD	20171106_LUMOS1_nLC13_AH_TechBench2_SILACH_1.raw

6.5 PEAKS DB Search result - Peptide View

The "Peptide View" displays the resulting peptide identifications that have been filtered by the current settings in Summary View and "Peptide View Filters". The table on the top shows the details of each peptide identification. The bottom section provides additional information about the peptide-spectrum matches for the native peptide selected in the table.

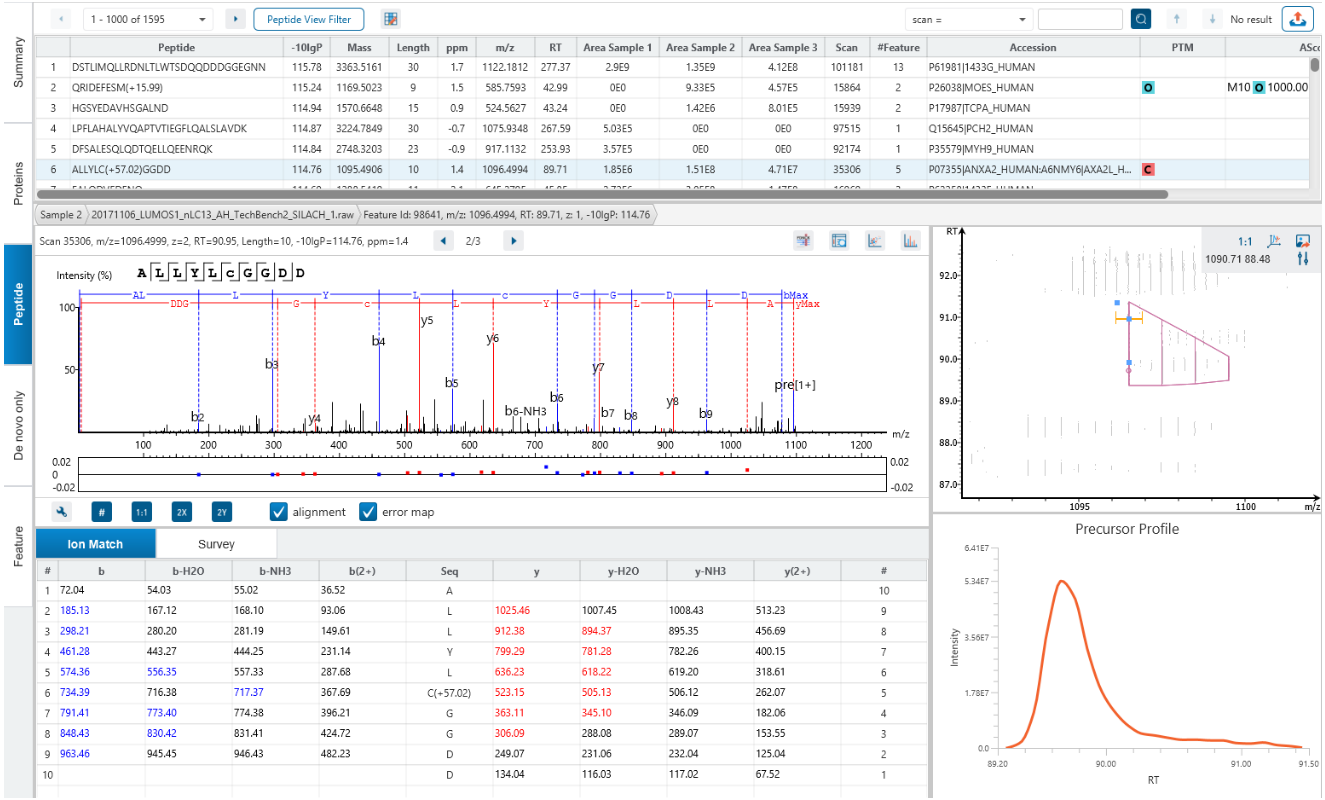

6.5.1 Peptide Table

The Peptide Table shows the filtered native peptide identification results. Each row in the table is a native peptide identification represented by its highest-scoring PSM. When there are more than 1000 peptides, the table is split into multiple pages.

Note: If there are multiple PSMs matched to a peptide, the table displays the information for the top-scoring PSM. Peptides with the same sequence but different modifications are considered different and shown in different rows.

To copy the sequence, right click on a row (or multiple rows) in the table and select "Copy selected peptide sequence(s)". Another option is to mouse over any cell in the table and hitting Ctrl + C on your keyboard to copy the contents of those selected cells.

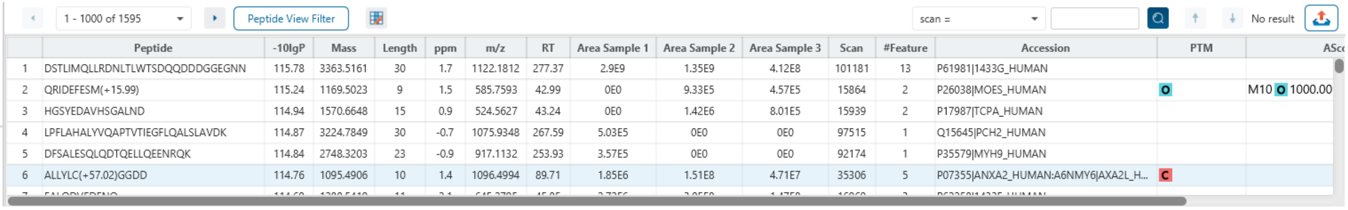

The following list describes the contents in each of the default columns:

- **Peptide:** The amino acid sequence of the peptide, as determined by the PEAKS searchworkflow. A modified residue is followed by a pair of parentheses enclosing the modification.
- **-10lgP:** The peptide -10lgP score. The score indicates the scoring significance of a peptide-precursor spectrum match.
- **Mass:** The monoisotopic mass of the peptide.
- **Length:** The number of amino acids in the sequence backbone.
- **ppm:** The precursor mass error, calculated as $10^6 \times$ (precursor mass - peptide mass) / peptide mass.
- **m/z:** The precursor mass-to-charge ratio.
- **RT:** The retention time (elution time) of the spectrum as recorded in the data.
- **Area per Sample:** The combined area for all features in that sample associated with this peptide.
- **Scan:** The scan number of the spectrum that matches the peptide sequence with the highest -10lgP.
- **#Feature:** For single sample analysis, shows number of features assigned to the peptide sequence from identification. For multiple samples, if peptide has feature vectors, it will show number of features used (area > 0) in all feature vectors. If not, it shows the number of features from identification.
- **Accession:** The accessions of proteins that contain this peptide.
- **PTM:** The types and the numbers of modifications present in the peptide shown in color-coded icons.
- **AScore:** Localization score assigned to modifications on the peptide. It is the -10 log of a p-value. In this case, the p-value is the probability that the modification occurs at the reported position compared to other possible positions. A -10lgP of 20 is equal to a p-value of 0.01. Scores are listed in the format AminoAcid, peptide position, modification, Ascore. For example, serine at amino acid 3 is phosphorylated with an AScore of 54.01 will be reported as: S3 P 54.01.
- **Found By:** The name of the PEAKS search workflow that identifies the peptide; this can be either PEAKS DB, PEAKS PTM, or SPIDER.

6.5.2 Peptide Table controls

The menu above the peptide table provides options to scroll through the table of peptides, filter the table, show optional columns and search for specific entries.

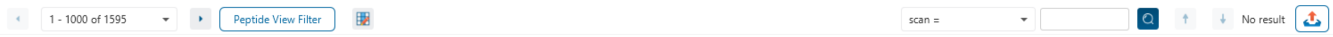

6.5.2.1 Peptide Table controls - Pagination

For easy viewing and to improve performance, the peptide table is limited to showing 1000 entries at once. Pagination is used to manage which set of 1000 entries will be viewed.

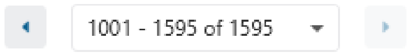

In the top left, there is a dropdown to select the rows for viewing. Click the left arrow or right arrow to select the previous/next 1000 rows. If the table is sorted or filtered, it may update the pagination set of rows that is currently in view.

6.5.2.2 Peptide Table controls - View Filter

Click on the 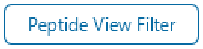

button to open the Peptide View Filter which is used to filter the Peptide table. The following are the filter options provided:

- Peptide sequence contains: Enter an amino acid sequence, only peptides that contain that sequence will be displayed in the peptide table. Adding PTMs in brackets in the sequence search is not handled, meaning only the sequence backbone is considered in the filter. Use the PTM contains for further filtering if necessary.
- Peptide sample area: Filters based on total peptide area found in each sample. Sample areas are calculated using the total of all peptide features from all spectra that identified the native peptide. If the peptide is found in multiple samples, only one sample must pass this filter for the peptide to be included.
- PTM contains: Type the names of PTMs you would like to include. Peptides containing PTMs that match the text will be included. If multiple PTMs match the text they will all be included in the table.

Note: In all filters, multiple entries can be separated by a semi-colon.

Click on the Reset button to reset all filters to their default setting.

Click on OK to apply the filters to the Peptide table.

Click on Cancel to discard the changes in the View Filter.

6.5.2.3 Peptide Table controls - Optional Columns

Click on the 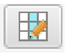 button to open the optional columns dropdown. By default the sample area columns are displayed. Sample Feature count columns can also be shown. These sample feature counts are always from identification, and does not consider whether there are feature vectors present. For projects with many samples, it might be useful to hide these optional columns and reduce the width of the peptide table.

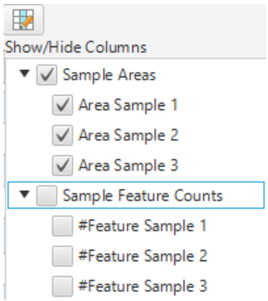

6.5.2.4 Peptide Table controls - Search function

On the top right of menu, there is a search function with 4 options. Search results can be navigated between using the Up or Down arrows.

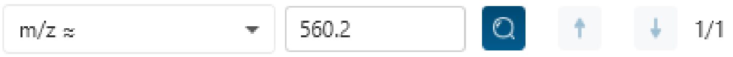

Options for searching include:

- **Scan =:** Search for the Scan number associated with each peptide. One scan can be associated to multiple peptides. For projects with multiple data files, the scan number is searched on every fraction.
- **m/z ≈:** Search for the approximate m/z. The search will consider a range of m/z equal to the search value +/- 1 of the last significant digit. For example, searching for m/z ≈ 560.21 will find all m/z between [560.20, 560.22]
- **RT ≈:** Search for the approximate RT. Likewise, the search will consider a range of RT equal to the search value +/- 1 of the last significant digit set. For example, searching for RT ≈ 55.5 will find all m/z between [55.4, 55.6]
- **Seq contains:** Search for the sequence in the table. If specifying a sequence without PTMs, the results will include that sequence and any sequences with PTMs with the same backbone. If specifying a sequence with bracket and some mass value (like in the above image), then only sequence that have a substring with an exact match will be considered.
  Additionally, since the peptide table shows both I/L and they can not be distinguished, then searching for I in the sequence is equivalent to searching for L in the sequence. i.e. Searching for “LVLK” is the same as searching for “LVIK”, “IVLK”, and “IVIK”

6.5.3 Feature selection breadcrumb

There is a sample and feature selection breadcrumb which allows you to switch between features associated with the peptide. Click on the Sample in the breadcrumb to reveal other samples and the features in that sample related to the current peptide selection. From here you can select other samples, fractions, or features.

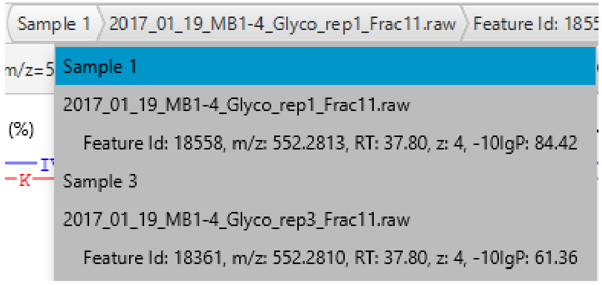

By clicking on the fraction level, all fractions in this sample are revealed as well as the features in each fraction associated with this peptide. From here you can select other fractions and features to view.

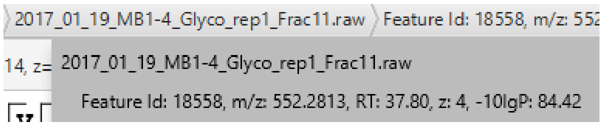

Lastly, clicking on the feature level will show all features in the currently selected fraction. Features with Feature Id = 0 are known as fake features and are constructed to enhance identification.

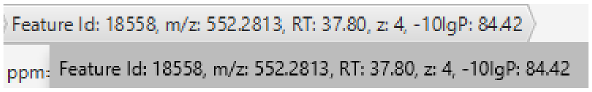

6.5.4 PSM selection

Underneath the feature breadcrumb will shown the currently selected PSM. Click on the Left and Right arrow buttons to switch between PSMs related to the currently selected feature.

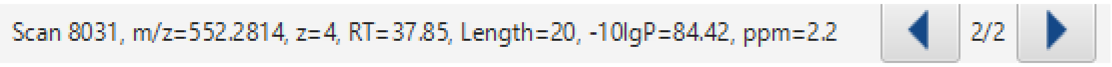

All other components in the Peptide tab interface will update according to the currently selected PSM.

For timsTOF instruments, Precursor ID is used rather than Scan number.

6.5.4.1 Protein jump button

Click on 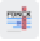 to jump to a protein that contains the currently selected PSM. If there are more than 1 protein, a dropdown is revealed which allows the user to select which protein to jump to. If a protein is filtered out due to a protein summary filter or summary page protein filter, then the option to jump will be grayed out.

6.5.4.2 Show spectrum in Data View

Click on 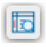 to open the Data Refine MS/MS tab (if not already opened) and jump to the corresponding selected Scan.

6.5.4.3 Show spectrum in LC/MS View

Click on 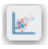 to open the Data Refine LC/MS tab (if not already opened) and jump to the corresponding selected Scan, highlighting the peptide MS/MS marker and the feature if present.

6.5.4.4 Show Raw Spectrum View

Click on 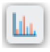 to show the raw spectrum chart of the currently selected Scan.

6.5.5 Annotated Spectrum Chart

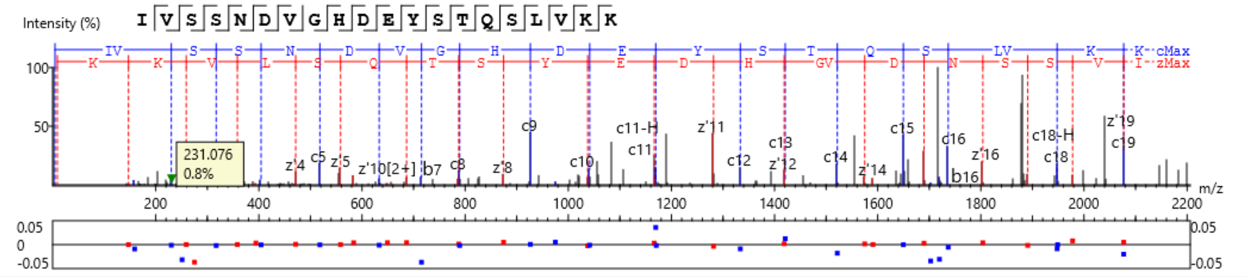

For the currently selected PSM, a graphical representation is shown in the form of an annotated spectrum chart. There are several controls provided for this view which will be explored in the following sections.

6.5.5.1 Annotated Spectrum Chart - Chart Navigation

Moving the cursor over the peptide sequence in the spectrum will show the mass transitions for a particular amino acid residue.

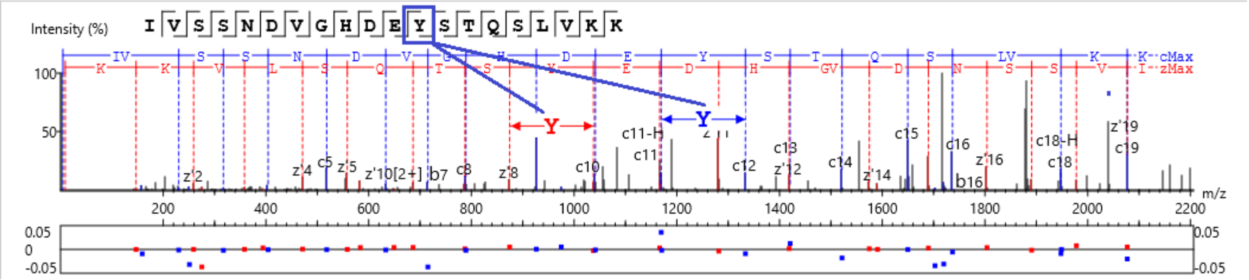

Zoom to a m/z region: Drag horizontally from the start m/z and to the end m/z with left mouse button.

Zoom in/out smoothly: Place the cursor at a particular m/z value (right below the x-axis), zoom in/out by scrolling the mouse wheel.

Increase/Decrease peak intensity: Place the cursor in the spectrum and scroll the mouse wheel.

See the whole spectrum: Double click in the spectrum or click the 1:1 button.

6.5.5.2 Annotated Spectrum Chart - Spectrum Annotation Settings

Click on the 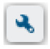 button to show the spectrum annotation settings.

Click on the 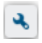 button to show the spectrum annotation settings.

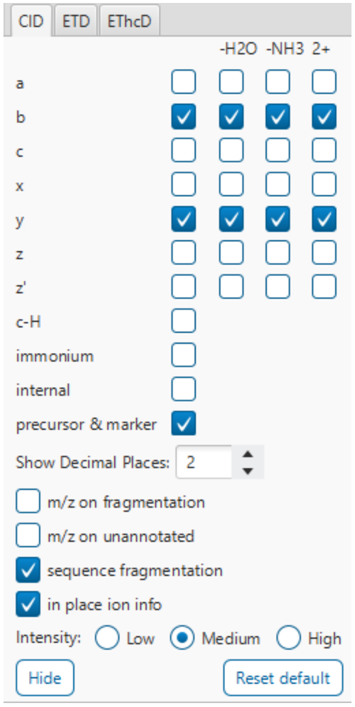

The spectra in the PEAKS results can be annotated by selecting ion types from the thorough collection of ions that PEAKS offers. The selected ion types will be displayed in the “Ion Match” table, as well. It is possible to annotate the spectrum with various ions for CID, EThcD and ETD. Each of these fragmentation modes can be set with different settings which will be shared with other results with the same fragmentation type.

To reset the defaults, click on the “Reset default” button.

Show Decimal Places: Select the number of decimal places that will appear in the ion table and the spectrum view. The default is set to two decimal places.

m/z on Fragmentation: Select this to display the m/z value on top of the annotated ions.

m/z on Unannotated: Select this to display the m/z value on top of the peaks without ion matches.

Sequence Fragmentation: Select this to display the sequence fragmentation on the top-left corner of the “Spectrum Annotation” view.

In Place Ion Info: Ion information, m/z value,and relative intensity are all displayed in a pop-up within the “Spectrum Annotation” view when this option is checked and the cursor is placed on a peak.

Intensity: Set the intensity threshold for spectrum annotation to low (2%), medium (5%), or high (10%).

6.5.5.3 Annotated Spectrum Chart - Additional Chart Controls

Click on # or % to toggle between absolute and percentage intensity values in the Y axis.

Click on 1:1 to reset the zoom level of the annotated spectrum chart to the default.

Click on 2X to zoom into the X axis by a factor of 2.

Click on 2Y to zoom into the Y axis by a factor of 2.

The "alignment" check box ☑ alignment allows user to display/hide how the fragment ions generated from the peptide align with the spectrum. N-terminal ions are shown in blue and C-terminal ions are shown in red.

The “error map” check box ☑ error map is used to show/hide the error map underneath the chart.

6.5.6 Ion Match Table

The “Ion Match” tab in the bottom pane contains a table with possible fragment ions for the selected MS/MS scan. Each ion in the table shows the calculated mass, where if the fragment ion is present in the spectrum, its mass value is displayed in color. N-terminal ions are shown in blue and C-terminal ions are shown in red. A fragment ion is found when there is a matching peak within the mass error tolerance, as defined in the de novo sequencing parameters, and when relative intensity of the matching peak is at least 2%. The ion types displayed in the table can be configured in "Spectrum Annotation Settings”.

Ion Match | Survey

#	b	b-H2O	b-NH3	b(2+)	Seq	y	y-H2O	y-NH3	y(2+)	#
10	1079.503	1061.488	1062.498	540.248	E	1596.643	1578.636	1579.610	798.818	13
11	1265.576	1247.569	1248.557	633.292	W	1467.601	1449.588	1450.585	734.297	12
12	1394.626	1376.616	1377.600	697.813	E	1281.521	1263.512	1264.488	641.266	11
13	1481.659	1463.648	1464.632	741.329	S	1152.480	1134.469	1135.461	576.736	10
14	1596.682	1578.671	1579.655	798.841	N(+0.98)	1065.449	1047.440	1048.427	533.228	9
15	1653.703	1635.693	1636.676	827.352	G	950.422	932.407	933.390	475.709	8
16	1781.773	1763.751	1764.735	891.381	Q	893.397	875.388	876.370	447.198	7
17	1878.815	1860.804	1861.788	939.907	P	765.341	747.326	748.315	383.169	6
18	2007.857	1989.847	1990.830	1004.429	E	668.288	650.274	651.258	334.642	5
19	2122.880	2104.870	2105.853	1061.940	N(+0.98)	539.245	521.231	522.222	270.124	4
[illegible]	[illegible]	[illegible]	[illegible]	[illegible]	[illegible]	[illegible]	[illegible]	[illegible]	[illegible]	[illegible]

Note: Uncheck the alignment checkbox in the spectrum annotation view to see the highlighted fragment ions more easily.

6.5.7 Survey Chart

The "Survey" tab displays the precursor ion spectrum. The buttons that appear in this section are identical to those explained above in Section 6.5.5.3 Annotated Spectrum Chart - Additional Chart Controls.

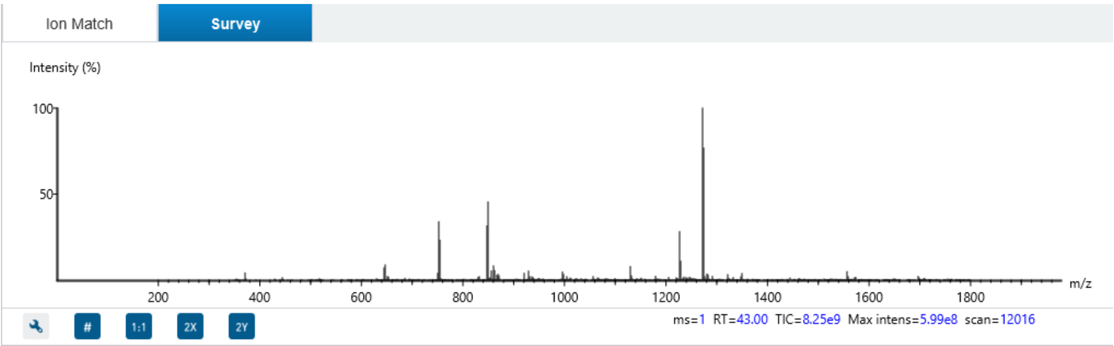

6.5.8 LC/MS Snapshot

The LC-MS Snapshot displays a zoomed-in view of the precursor scan in the LC-MS heat map 2-D view. The peptide feature is highlighted in purple and the selected PSM marker is blue. The span of the orange line in the LC-MS Snapshot designates the isolation window.

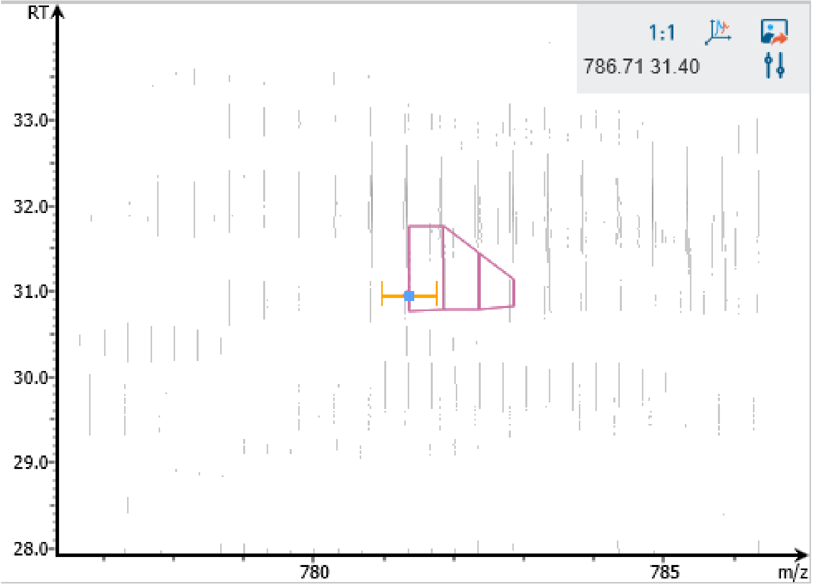

Users can change the view from the 2-D heat map to a 3-D intensity view by clicking the intensity view button 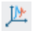 in the upper right-hand corner of the LC-MS Snapshot. In addition, users can export the LC-MS heat map by clicking the export image button 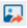 in the upper right-hand corner of the LC-MS Snapshot.

Expand the options dropdown by clicking 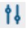 to reveal a search option to specify a *m/z RT* (ensure there is a space between these two numbers) location to zoom into. There is also a Peak slider filter to filter out low intensity peaks and a feature area filter to filter out low area features.

6.5.9 Precursor Profile

The Precursor Profile, or eXtracted Ion Chromatogram (XIC) of the precursor ion, graphs the elution profile of the selected peptide feature vector over the retention time range where it was identified. The graph is plotted with the retention time along the x-axis and the intensity along the y-axis.

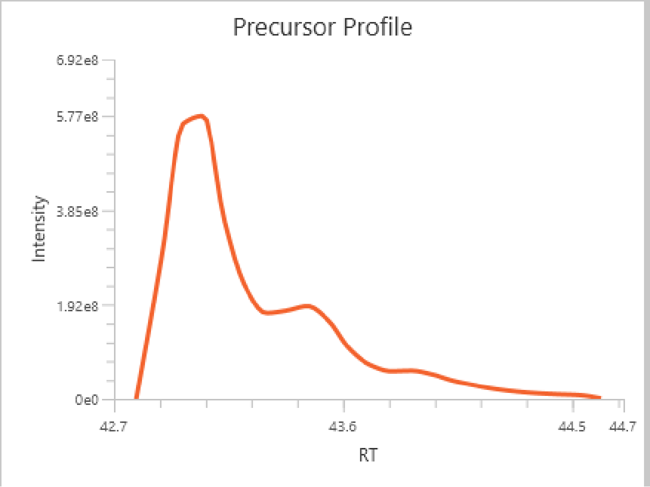

6.6 PEAKS DB Search result - De novo only View

The "De novo only View" displays the filtered de novo only sequences. A de novo sequence is de novo only if it is from an MS/MS spectrum that is not confidently assigned to any database peptide. The table shows the filtered de novo only sequences by the current settings in the Summary View. De novo only sequences may suggest novel peptides, peptides with unknown modifications, or other interesting research subjects. The interface is identical to the Peptide View in a de novo sequencing result node. For detailed instructions, please refer to Section 4.4 De novo result - De novo View.

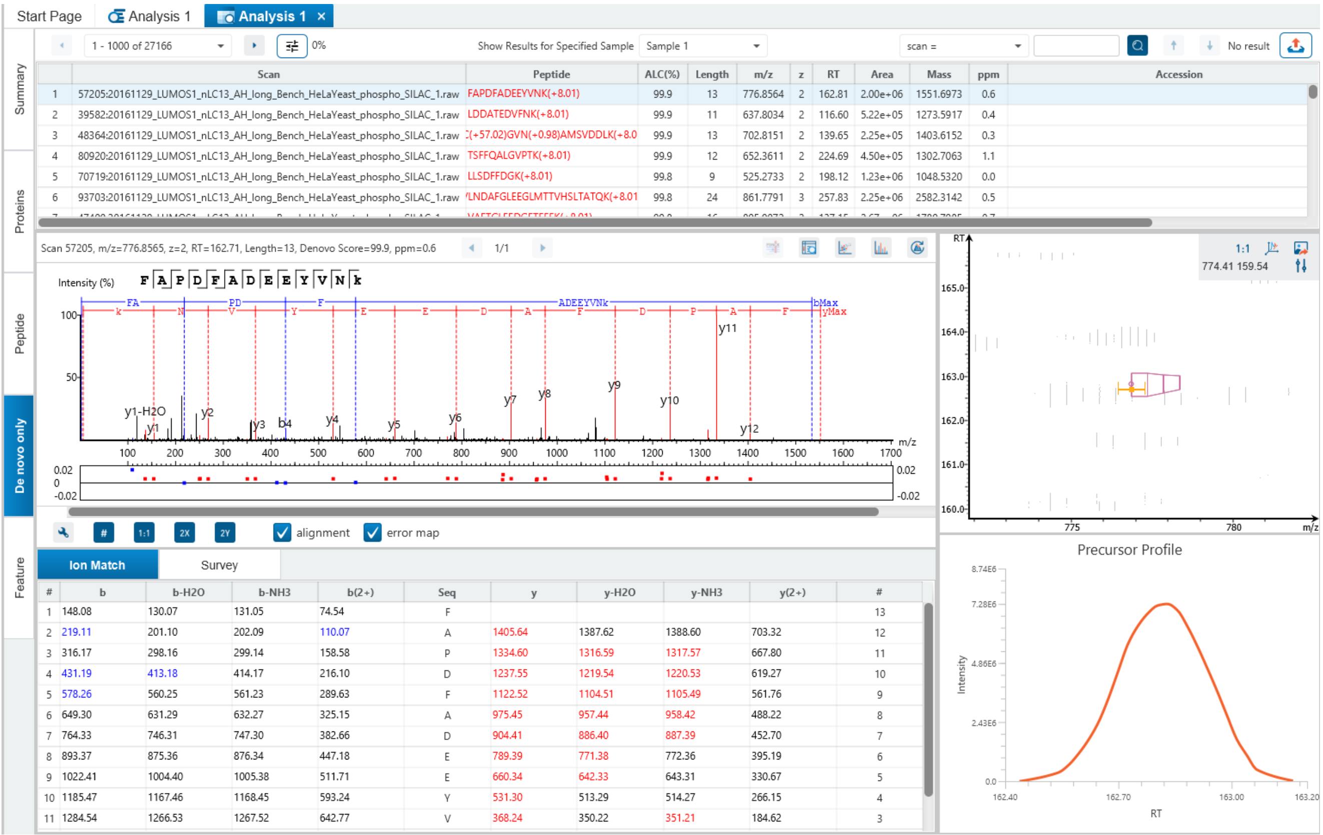

6.7 PEAKS DB Search result - Feature View

The "Feature View" displays all the feature information in greater detail, as shown in the next figure. The table on the top displays features, while the bottom section provides additional information about the feature, precursor profile, and if available peptide-spectrum match. For detailed instructions, please refer to Section 4.4 De novo result - De novo View.

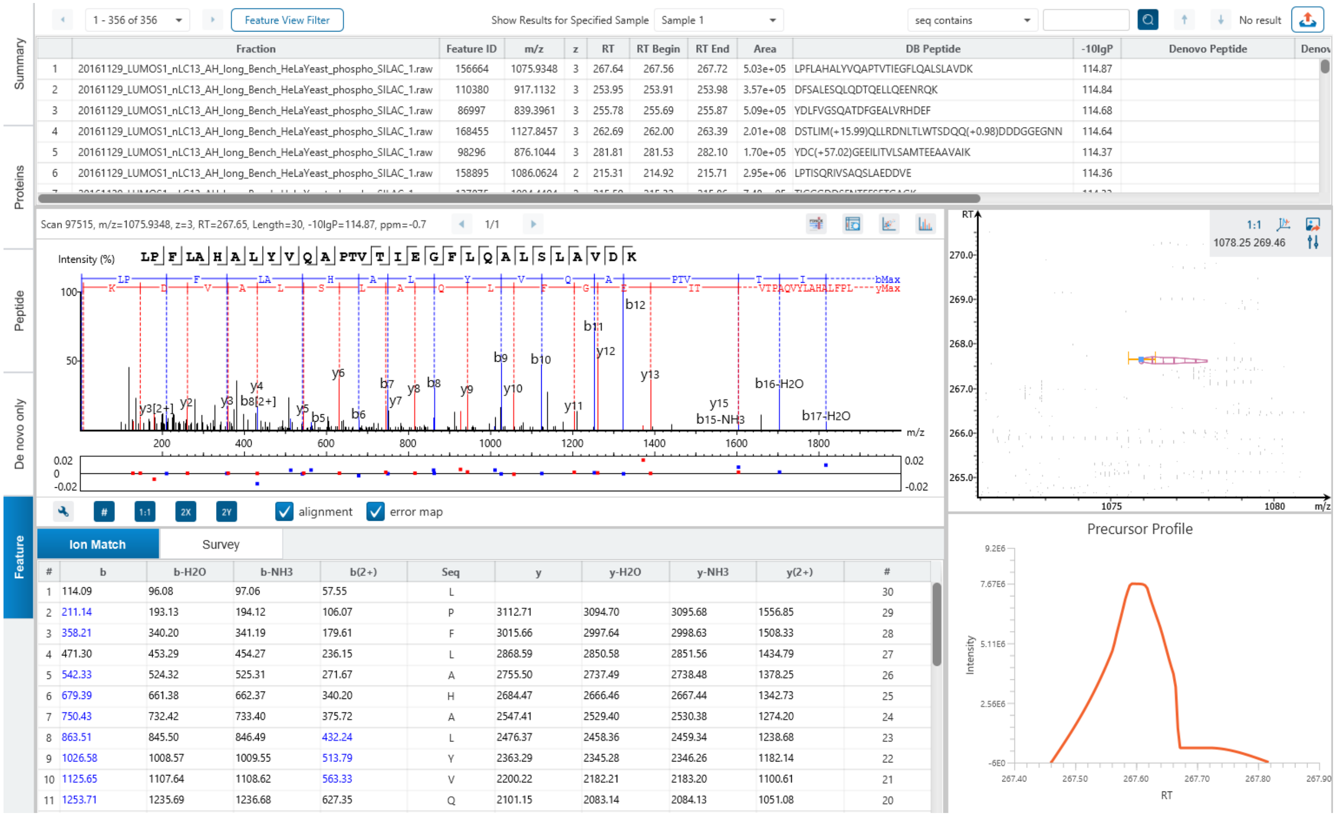

6.8 PEAKS DB Search result - Exporting

In Project View, double-click on Export to open the Export node.

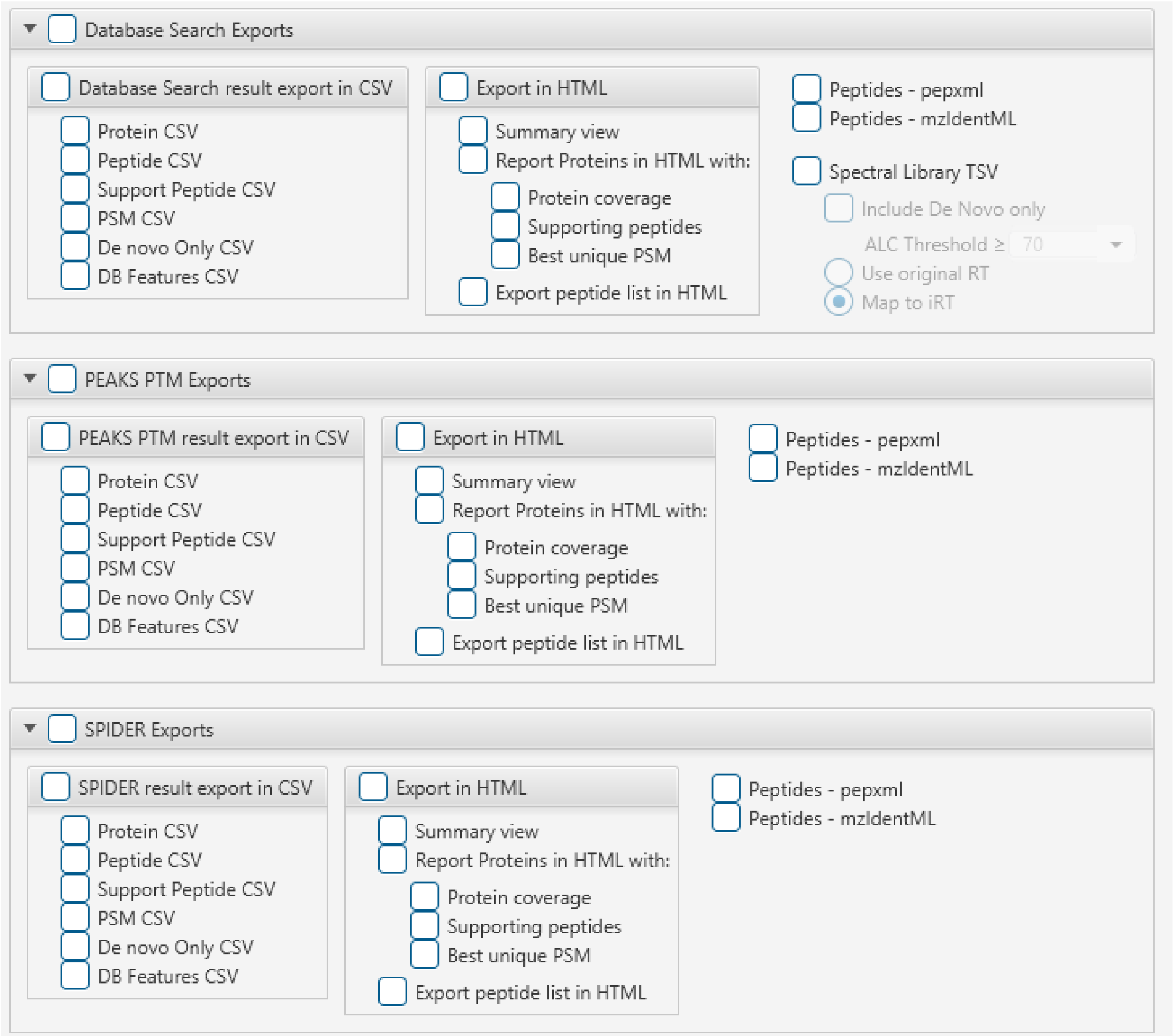

6.8.1 PEAKS DB Search result - Exporting - comma separated values (CSV) format

There are several options for exporting the PEAKS Identification results in CSV format:

- **Proteins CSV:** The list of protein identifications, filtered by the protein filters in the Summary view and will be saved to proteins.csv.
- **Peptide CSV:** All of the identified peptides and details will be saved to peptides.csv.
- **Support peptides CSV:** A list of supporting peptides of each protein identification, filtered by the protein filters in the "Summary" view and will be exported to protein-peptides.csv. This usually contains more entries than the Peptide table as a peptide can be identified in several proteins and, therefore, can be reported multiple times in this file.
- **PSM CSV:** The peptide-spectrum matches (PSMs) will be exported to DB search psms.csv. Peptides differentiated with only I/L isoform are represented by separate entries. As a result, the number of entries in this file might be bigger than the number of PSMs mentioned in the Summary view.
- **De novo only CSV:** Exports the De novo only table. This export does not consider any sorting and will export a separate file for each sample.
- **DB Features CSV:** This export does not consider any Feature view filters or sorting. Each sample will be in a separate export.

6.8.2 PEAKS DB Search result - Exporting - HTML format

Export in HTML: This will generate single or multiple HTML reports in the specified location.

The following exporting options are available:

- **Summary view:** The “Summary” view page will be saved as a summary.html file in HTML format in the specified location.
- **Protein coverage:** The coverage pane will be saved for each protein.
- **Supporting peptides:** A list of supporting peptides will be saved for each protein.
- **Best unique PSM:** The best unique PSM will be saved for each protein.
- **Export peptide list in html:** creates a separate HTML-containing peptide identifications.

An individual protein will have its own HTML output file, where the corresponding protein coverage, the supporting peptides, and the best unique PSM are gathered.

6.8.3 PEAKS DB Search result - Exporting - pepXML and mzIdentML

Peptides - pepXML: A list of peptide-spectrum matches will be saved to pep.xml in pepXML format. The number of the “spectrum_query”elements will be the same as the number of PSMs mentioned in the "Summary" view, and the number of the “search_hit” elements will be the same as the number of PSMs counted from Peptide table (summing up #Spec column). Current version of pepXML export is 1.22.

Peptides - mzidentml: A list of peptide-spectrum matches will be saved to mzidentml.xml in mzIdentML format version 1.2.0. To validate the export, search for the following keywords and compare with the results in PEAKS Studio:

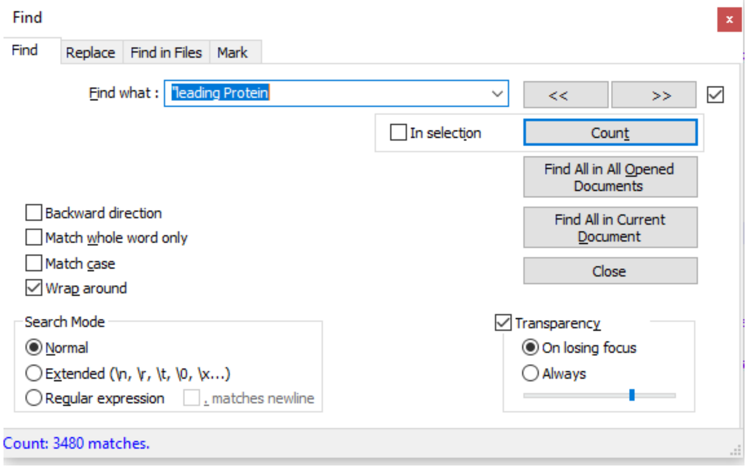

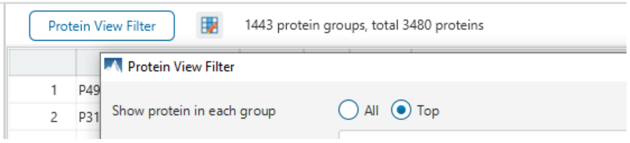

</PeptideSequence>: Equal to the number of peptides in the Peptide table

DBSearch accession: Equal to the total protein count in the Protein table

</ProteinAmbiguityGroup>: Equal to the number of protein groups

“leading Protein: Equal to the number of top proteins, disregarding protein filters

Non-leading Protein: Equal to the number of non-top proteins, disregarding protein filters

“protein group passes threshold” value =”true”: Equal to the number of protein groups based on current protein filters

passThreshold=”true”>: Equal to the number of proteins based on current protein filters

6.8.4 PEAKS DB Search result - Exporting - Spectral Library generation

Spectral Library TSV: PEAKS identification results can be exported as a spectral library that can be used by the Library Search engine. Check “Include De Novo only” and select an ALC threshold to include de novo only peptides in the library. Select “Use original RT” to keep original RT values in the library. Select “Map to iRT” to save iRT values in the library. The map to iRT function maps experimental retention times for peptides to an indexed RT (iRT) to account for chromatographic differences between runs (e.g. different gradient lengths).

For more information on how to configure a PEAKS generated spectral library, refer to Section 2.9 Spectral Library Configuration

6.9 PEAKS PTM Finder

PEAKS PTM Finder is an optional dedicated search tool for unspecified modification search performed after Database Search. Open the PEAKS PTM result by double-clicking on the PEAKS PTM node in the Project View.

PEAKS PTM uses the entire list of common and uncommon built-in PTMs in PEAKS Studio unless specified by the user (see section 6.1 Database Search Workflow and Parameter Settings)

For more details, refer to the paper: *"PeaksPTM: Mass Spectrometry Based Identification of Peptides with Unspecified Modifications" Journal of Proteomics Research, 2011, 10(7): 2930-2936.*

In the Protein table it will be common to see Proteins with many PTMs associated with their supporting peptides.

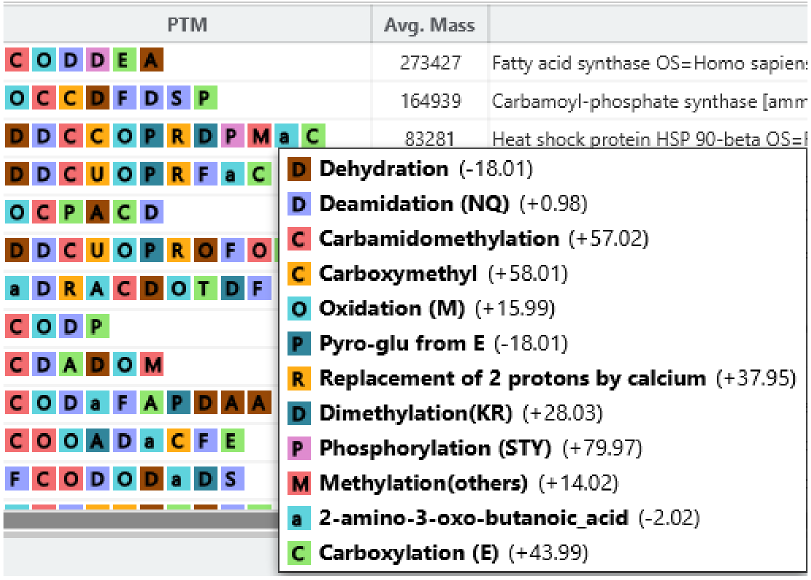

All user interface related functions as described in the previous sections in this Chapter 6 are the same for the PEAKS PTM result.

6.10 SPIDER

SPIDER is an optional dedicated search tool for homology search after Database Search.

For more details, refer to the paper: *"SPIDER: Software for Protein Identification from Sequence Tags Containing De Novo Sequencing Error." J Bioinform Comput Biol. 2005 Jun;3(3):697-716.*

Open the SPIDER result by double-clicking on the 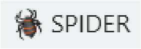

node in the Project View.

In the Protein table, supporting peptides with mutations are shown as white coloured blocks.

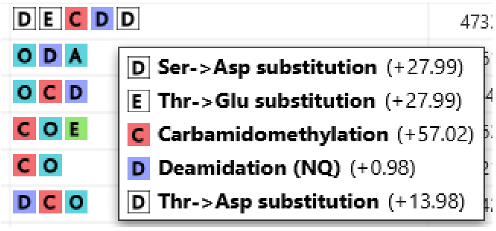

All user interface related functions as described in the previous sections in this Chapter 6 are the same for the SPIDER result.

7. DIA Workflow (Spectral Library, DB Search, De Novo, Quantification)

PEAKS Library Search offers an accurate and sensitive identification method for complex spectra, such as DIA spectra. In addition to annotated spectra along with precursor m/z and charge, PEAKS Library search also uses predicted retention time and ion- mobility collisional cross section (CCS) data for improved sensitivity.

To start a DIA analysis, ensure all samples selected for the workflow have DIA Acquisition mode.

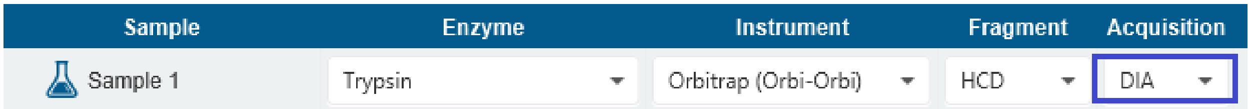

7.1 DIA Identification Analysis Workflow and Parameter Settings

To run a DIA Identification Analysis, Select Identification in Workflow Selection

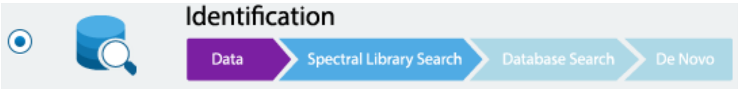

The Spectral Library, Database Search, and De Novo are all optional steps in the DIA workflow. Any combination of these analysis can be run, but at least one of these steps must be included in the analysis.

7.1.1 DIA Identification - Spectral Library Search parameters

The first step is the optional Spectral Library Search.

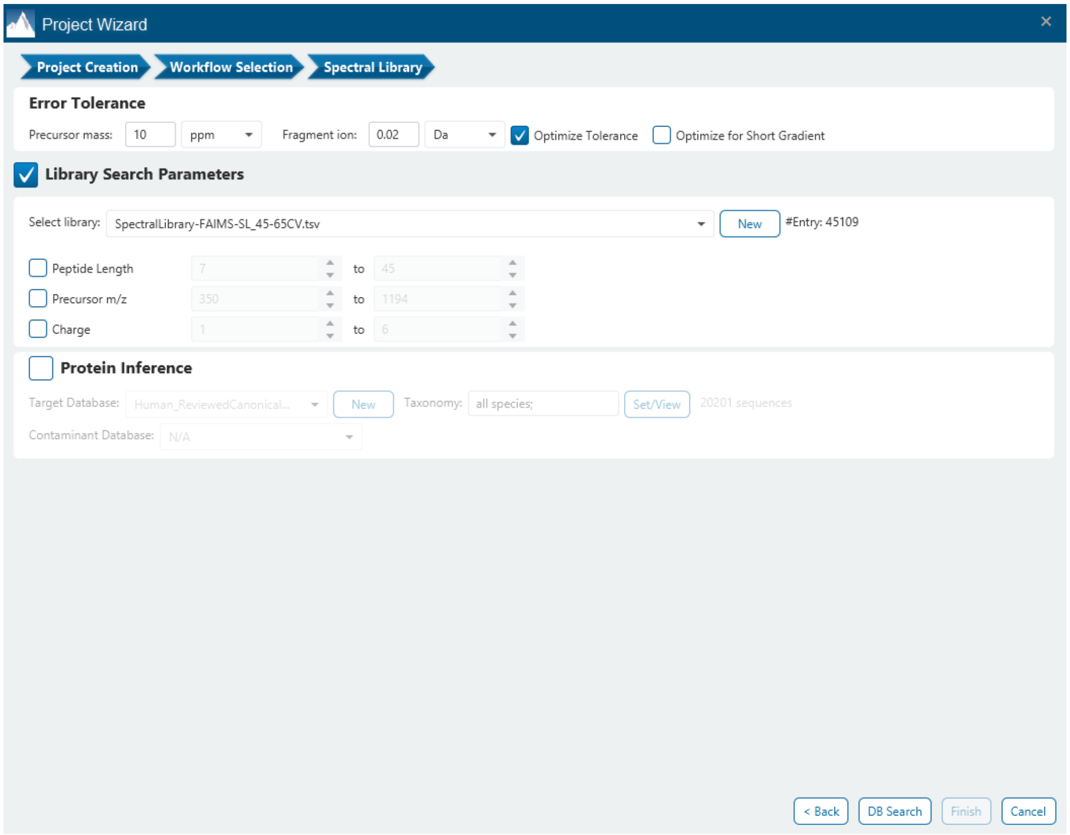

The following parameter settings are provided:

Precursor Mass Error Tolerance: The maximum mass difference between the peptide and the monoisotopic mass of the precursor ion.

Precursor Mass Tolerance unit: PEAKS generates peptides within the precursor mass error tolerance. Precursor mass error tolerance can be specified in either Daltons or ppm.

Fragment Mass Error Tolerance: PEAKS uses this value when scoring peptide sequences. PEAKS Studio considers a fragment ion to be matched if the calculated m/z is within the specified error tolerance. Units are Daltons.

Both precursor and fragment error tolerance parameters need to be set consistently with the mass accuracy of the instrument.

Optimize Tolerance: When enabled, PEAKS Studio will determine the optimal values to use for Precusor and Fragment Mass Error Tolerance for each fraction in your analysis. These values will replace the values provided in the previous Precursor and Fragment Mass Tolerance sections.

Library Selection: Select a PEAKS-generated library for the search. Select one from the list of libraries that have been configured in PEAKS Studio and filter for the peptide length, precursor m/z, and charge when necessary. To configure a new library, click on the New button. For more information on Spectral Library Configuration, refer to 2.9 Spectral Library Configuration. Also of interest is the chapter on Library Viewer.

Library Filters: The spectra that will be used from the library can be filtered based on peptide length, precursor m/z, and charge. This is optional. Each filter can be enabled by checking the checkbox to the left of the filter inputs.

Protein inference can optionally be enabled to search peptides found in the selected library against a provided database.

Target Database: Select a reference database from the list of configured databases or create a new one, and set the taxonomy if applicable. This database is the database used for protein inference.

Contaminant Database: A contaminant database can optionally be provided as well. #CONTAM# will be added to the beginning of the accession of all identifications from the contaminant database.

7.1.2 DIA Identification - DIA DB Search parameters

PEAKS DIA DB can be launched as a standalone workflow, or launched on top of a PEAKS Library Search workflow. It is an optional step in the workflow.

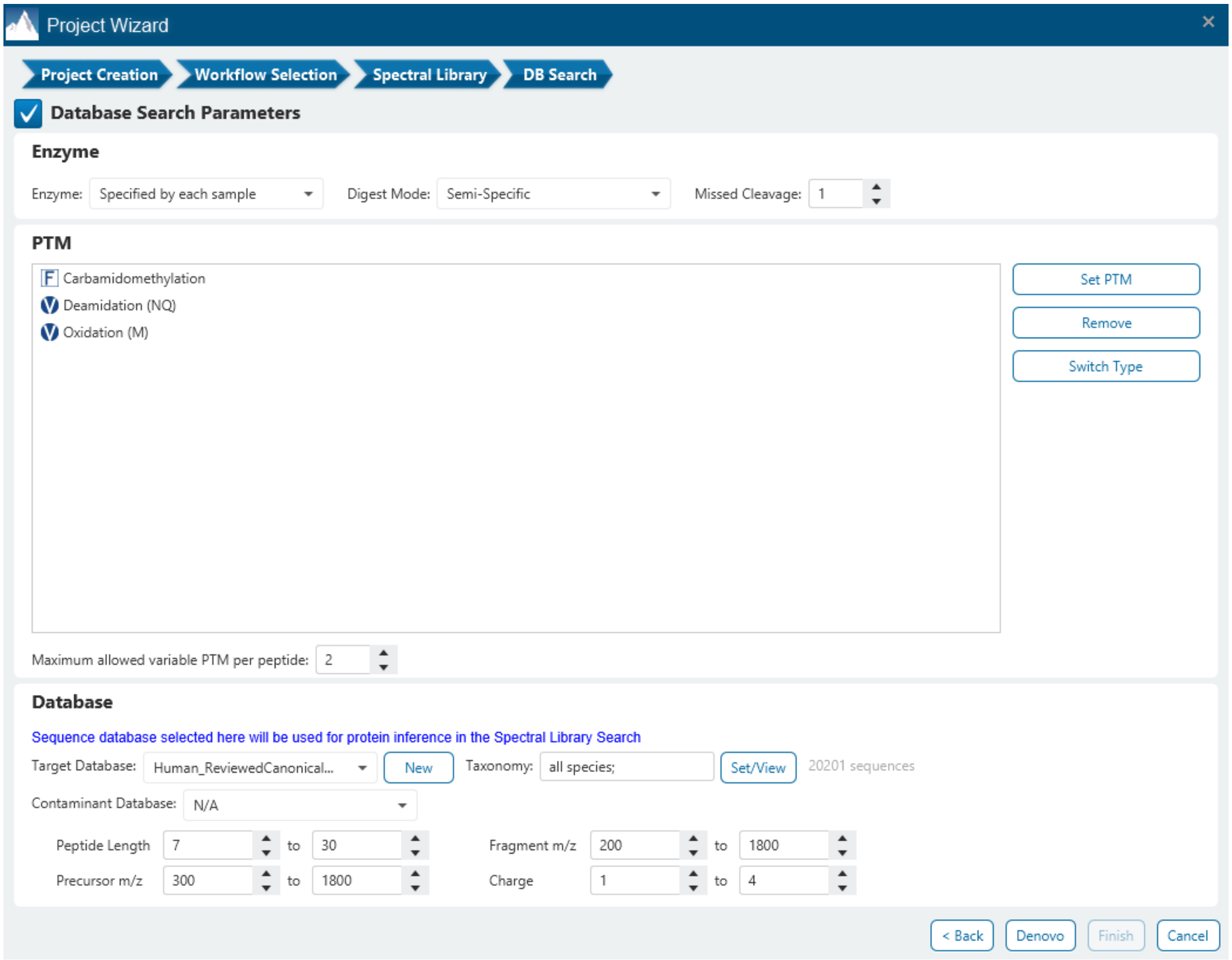

The Precursor and Fragment Mass Error Tolerance are set and shared in the previous Spectral Library step.

Enzyme Settings: Select the enzyme used for protein digestion from the enzyme drop-down menu.

> **Note:** "None" is a special enzyme allowing non-specific cleavage at both ends of the peptide. It is the recommended option when no digestion enzyme was used or when the digestion enzyme exhibits a high degree of non-specificity.

> **Note:** When the selected dataset is digested with different enzymes, "Specified by each sample" allows samples to be analyzed separately using their respective enzymes specified during project creation.

Digest Mode: This specifies the type of in silico cleavages allowed at a peptide termini based on the method used for protein digestion. If "Specific" is selected, the specificity of the selected enzyme is strictly enforced at both termini of a peptide. "Semispecific" allows one terminus to disobey the enzyme specificity rules.

> **Note:** If the enzyme is specified as "None", then no matter which mode is selected for "Digest Mode", the "None" enzyme digest rule will be applied.

Missed Cleavages: This specifies the number of missed cleavage sites allowed in a peptide.

PTM Settings: Click “Set PTM” to open the **PTM Options** dialog and specify the fixed PTMs and a few common variable PTMs expected in the experiment. Highlight PTMs and click on the button to place them as either Fixed or Variable PTM. From there, highlighted PTMs can be removed by clicking the “Remove” button. Clicking “Remove All” will reset the PTM options. Highlighting and clicking “Switch Type” will move the Fixed PTM in into the Variable PTM list and vice-verse. Click on “New” to create a new custom PTM. While a PTM is selected, click on “View” to see more details.

> **Note:** Multiple PTMs can be highlighted by holding down the CTRL key or SHIFT key on the keyboard.
>
> **Note:** For a specific residue, only one fixed modification is allowed.
>
> **Note:** For DIA Database search, currently only the following PTMs are supported: Carbamidomethylation, Oxidation (M), Deamidation (NQ), and Acetylation (Protein N-term).

Once the PTMs are selected, the maximum number of variable PTMs per peptide can be specified and a value of less than 4 is recommended.

Target Database: Select a protein sequence database for the search. Select one from the list of databases that have been configured in PEAKS and set the taxonomy by clicking on Set/View, if applicable. To configure a new database, click on New next to the Target Database dropdown to open the Database Configuration dialog.

Note: If running Spectral Library search, the database selected here will be used for protein inference, regardless if it was checked or not.

Contaminant Database: To search for contaminants, select a contaminant database from the list of databases that have been configured in PEAKS. Keep the selection as N/A if no contaminant database is required for the search.

Database search filters: The peptide length, fragment m/z, precursor m/z, and charge are filters applied to the final database search result.

7.1.3 DIA Identification - DIA De Novo parameters

PEAKS DIA De Novo is an optional step that can be launched as a standalone workflow, or launched on top of a PEAKS Library Search, PEAKS DIA Database Search, or PEAKS Library Search + PEAKS Database Search workflow.

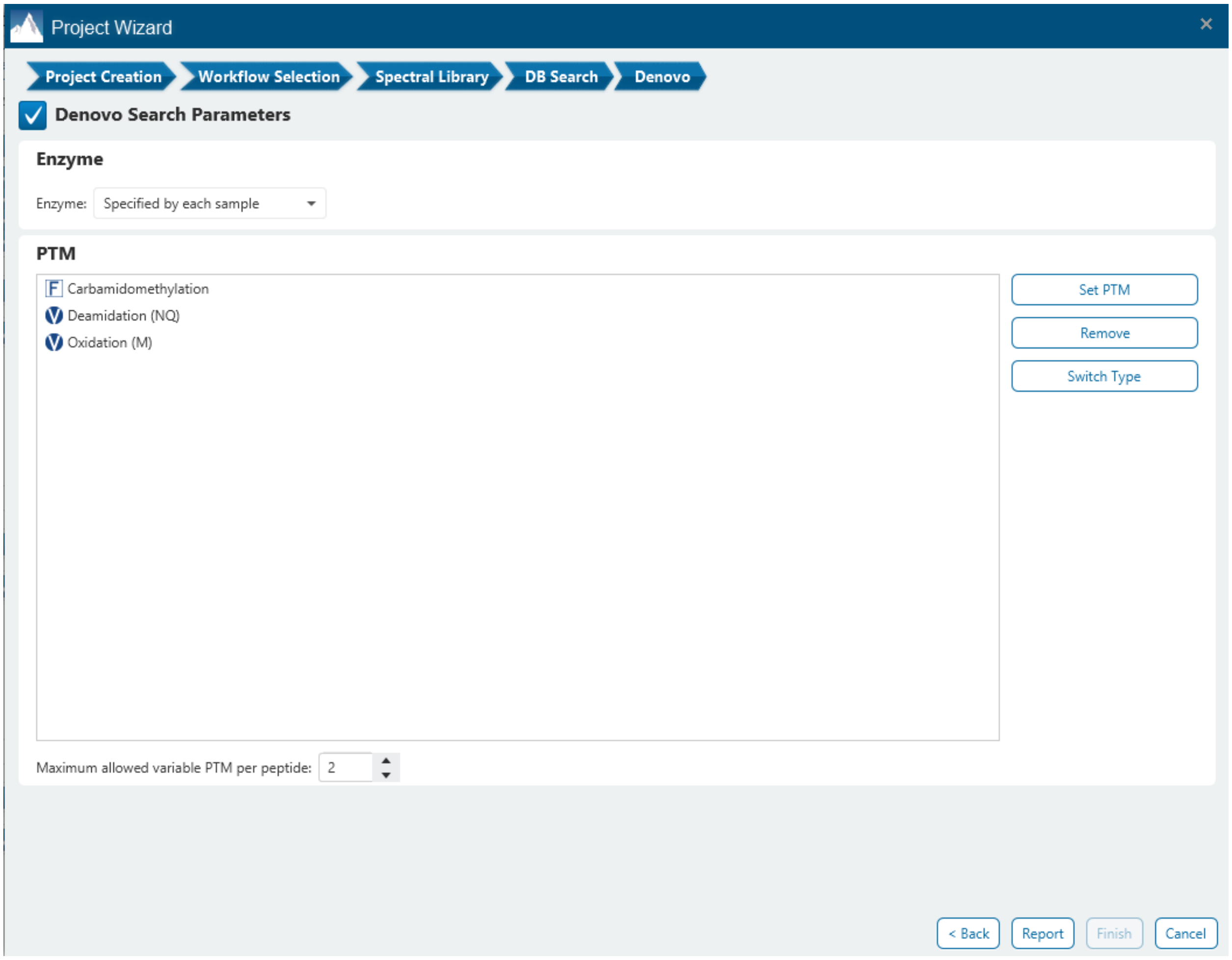

The Precursor and Fragment Mass Error Tolerance are set and shared in the Spectral Library step.

Refer to the previous section 7.1.2 DIA Identification - DIA DB Search parameters for details on configuring the Enzyme and PTM settings.

7.1.4 DIA Quantification (DIA LFQ) Analysis Workflow and Parameter Settings

DIA Label Free Quantification requires at least 2 samples in the analysis. It is recommended to only run LFQ with 1 data file per sample, but if you choose to have fractionated samples, ensure each sample has the same number of fractions. All samples must have DIA Acquisition mode set.

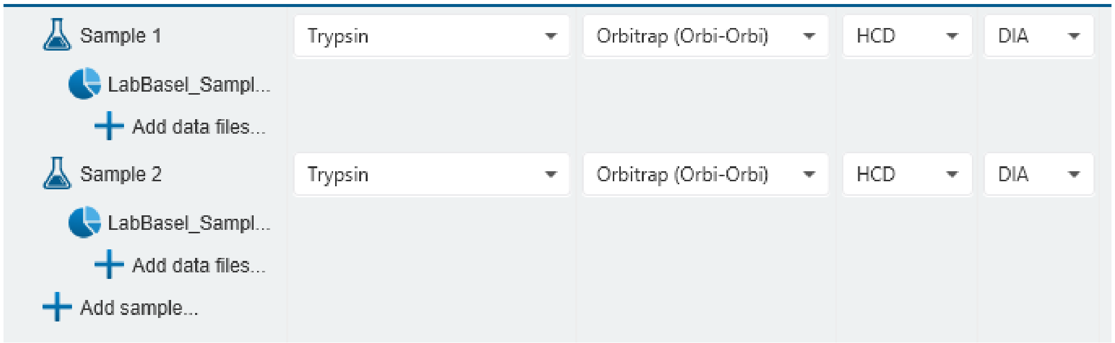

To run the DIA Label Free Quantification workflow, choose the Quantification option in Workflow Selection.

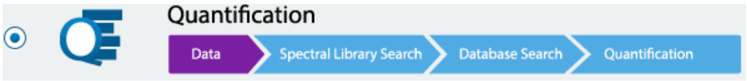

The Spectral Library Search step can be selected and details are provided in section 7.1.1 DIA Identification - Spectral Library Search parameters.

The DIA DB Search step can be selected and details are provided in above section 7.1.2 DIA Identification - DIA DB Search parameters.

Select one or both of these identification steps in order to run DIA Label free quantification.

After deciding on the DIA Identification parameters, click on 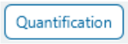

to proceed to the DIA label free quantification parameters page.

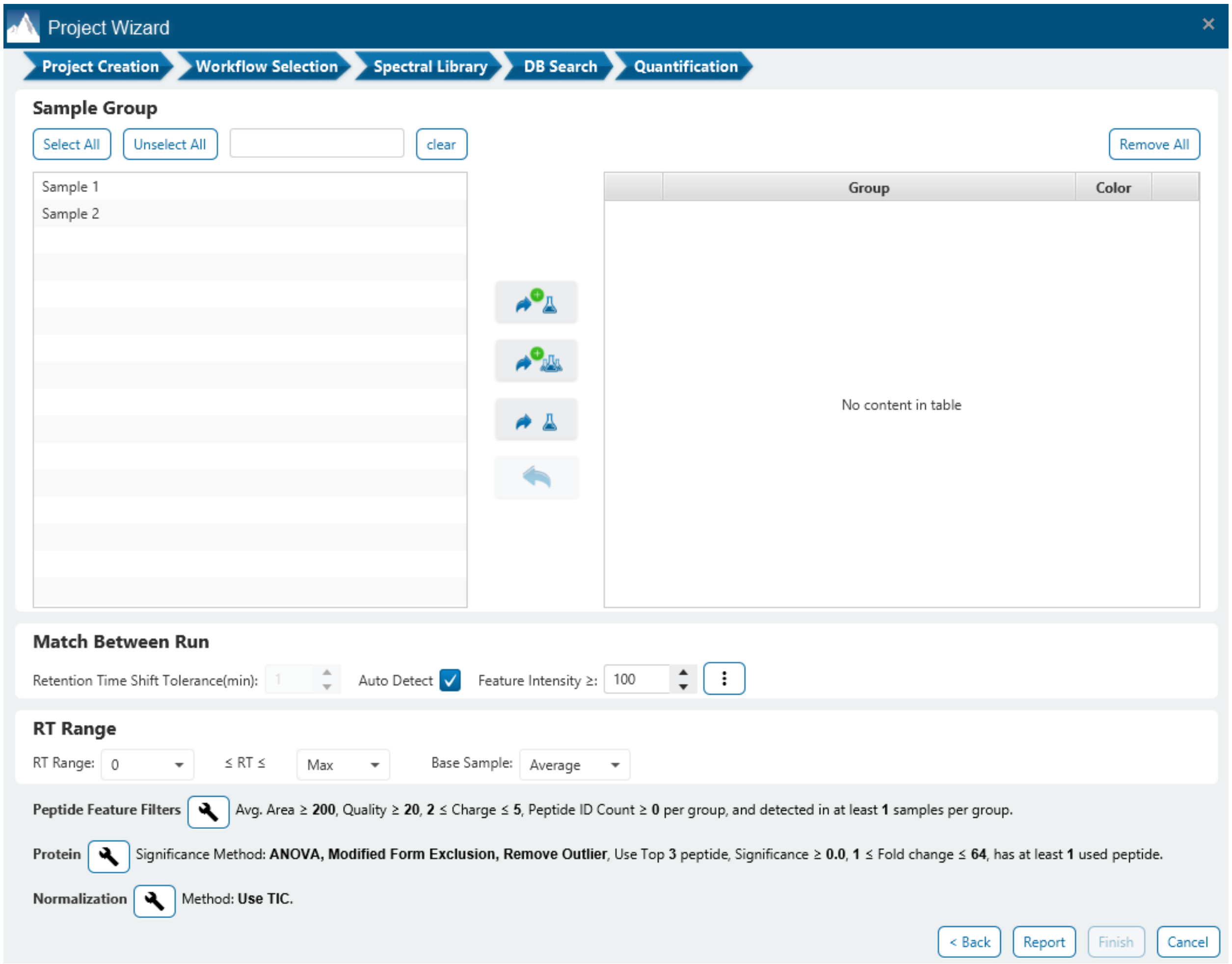

The following parameters are provided for Label Free Quantification (LFQ):

Sample Group: LFQ requires sample grouping. All available samples are listed in the left window. After selecting a sample, it can be added to a new group by clicking the 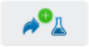 button or to an existing group by clicking the 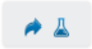 button and selecting that option from the drop-down menu. If you select multiple files, you can add each

one individually to create multiple new groups by clicking the button. Samples and Groups can be removed by clicking the button next to each element. The names and colours for each group can be edited by clicking on the coloured square dropdown, respectively.

Match Between Run:

Retention Time Shift Tolerance: The retention time shift tolerance is the maximum elution time difference that is considered for the quantification of an identified peptide after RT alignment. Select "Auto Detect" to let PEAKS Studio detect the optimal Retention Time Shift Tolerance for your data based on the observed trend.

Ion Mobility Tolerance (1/k0) (if applicable): The maximum 1/k0 differential for features to transfer IDs.

Note: This parameter is only available when the analysis contains timsTOF data.

Feature intensity: Set a value for the minimum intensity a peptide feature must exhibit.

RT Range: Feature vectors within the selected retention time range will be included in the results while everything else will be removed.

Base Sample: The base sample will act as the reference. The peptide and protein abundance corresponding to this reference label will be the denominator in all of the abundance ratio calculations.

Peptide Feature Filters:

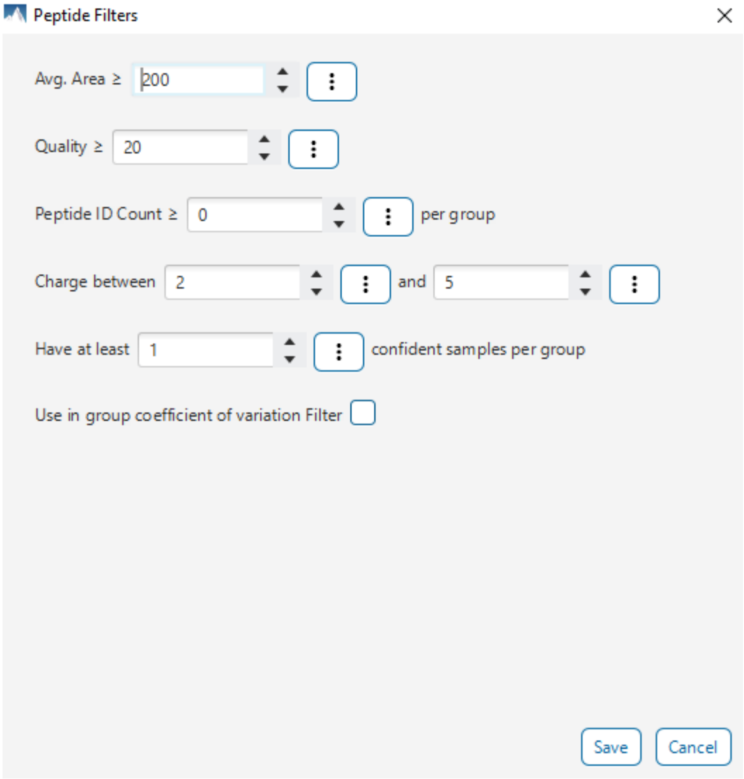

- **Avg. Area:** The minimum average area of all peptide features associated with the peptide. Features with higher abundance have been shown to be more reproducible across replicates. Only peptide features with an average area above this threshold will be used for quantification.

- **Quality:** The quality score for the peptide feature. Factors that affect the quality score include m/z difference, RT difference, XIC shape similarity, and the feature intensities.
- **Peptide ID Count:** This filter sets the minimum limit for how many times a peptide must be identified within a group to be included in the LFQ results.
- **Charge between:** Only peptides with feature vectors that all fall within this range will be used in protein quantification.
- **Have at least ___ confident samples per group:** This filter sets the minimum number of sample(s) per group that the peptide feature has an abundance value. A peptide is more quantifiable when it is detected in more samples.
- **Use in group Coefficient of Variation Filter:** For each feature vector (set of peptide features associated with a peptide), at least one group must have a coefficient of variation (CV) less than the CV threshold. The CV threshold is automatically selected by the software based on the current data set.

Protein Filters:

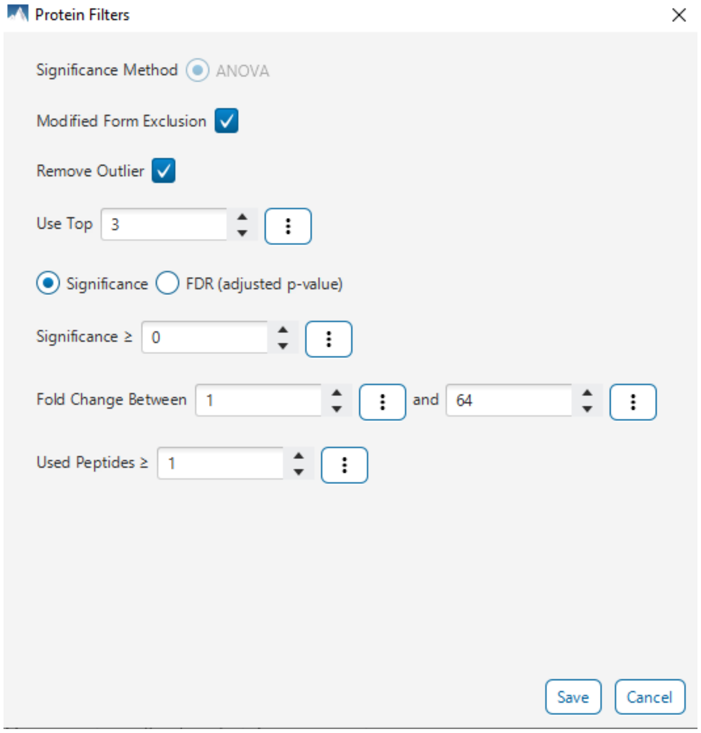

- **Significance Method:** Protein abundance is used for the significance testing calculation. When there are replicates in each group, ANOVA is suggested to be used for significance testing. For more details on ANOVA, refer to the following paper: *"On the comparison of several mean values: an alternative approach ", Biometrika, 1951, 38(3/4): 330-336*
- **Modified Form Exclusion:** The expression level of modified peptides might be different than the overall expression level of a protein. In such cases, including modified peptides for protein ratio calculation might lead to incorrect results. By checking this box, if a peptide has modified and unmodified forms, both will not be used as candidates for protein ratio calculation, if a peptide only has one modified form, it is still a candidate, but if a peptide has more than one modified form, all forms will not be used as candidates
- **Remove Outliers:** This filter removes supporting peptides that have a different variation trend compared to others.
- **Use Top # peptides:** This is the number of quantifiable supporting peptide that will be used to calculate the protein profile. Three is the default setting.

- **Significance:** Only protein groups with a significance above this threshold will be listed in the “Protein” view. The significance score is calculated as the -10log of the significance testing p-value (e.g. Significance score of 20 is equivalent to a p- value of 0.05). PEAKS provides the significance testing method: ANOVA.
- **FDR:** Adopt the Benjamin-Hochberg method to adjust the p-value to the false discovery rate for all protein groups that have already passed the other filters. Only protein groups with significance scores passing the calculated FDR will be listed in the "Protein" view. Either this or "Significance" can be selected to set a significance threshold.
- **Fold Change:** Only protein groups below the lower threshold or above the upper threshold value will be listed in the "Protein" view.
- **Used Peptides:** Only protein groups that were quantified with this number of peptides (or more) will be listed in the "Protein" view.

Normalization Method:

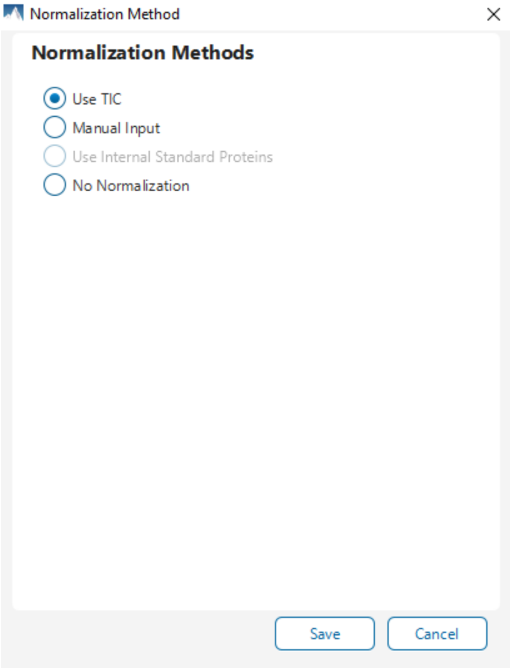

- **Use TIC:** By default, PEAKS Studio uses the total ion current (TIC) of the samples to calculate the normalization factors. Normalized abundance for each sample is calculated from the raw abundance divided by the normalization factor for that sample.
- **Manual input:** This option performs the same default calculation as auto normalization ("Use TIC") but also allows for the manual change of each sample's expected ratios. This option should be used if the expected ratio is not 1:1 for each sample as the software attempts to normalize to a 1:1 ratio.
- **Use internal standard proteins:** This option displays a list of identified proteins (when a database or library search has been completed) that can be selected to create a normalization ratio. A search bar is included to enable the quick location of select proteins.
- **No Normalization:** All samples are automatically assigned Factors of 1.0.

Click on “Report” to proceed to the final step. Report filters can be set here which will filter the final result.

Both the DIA Identification and DIA Quantification workflows have the same Report filter step.

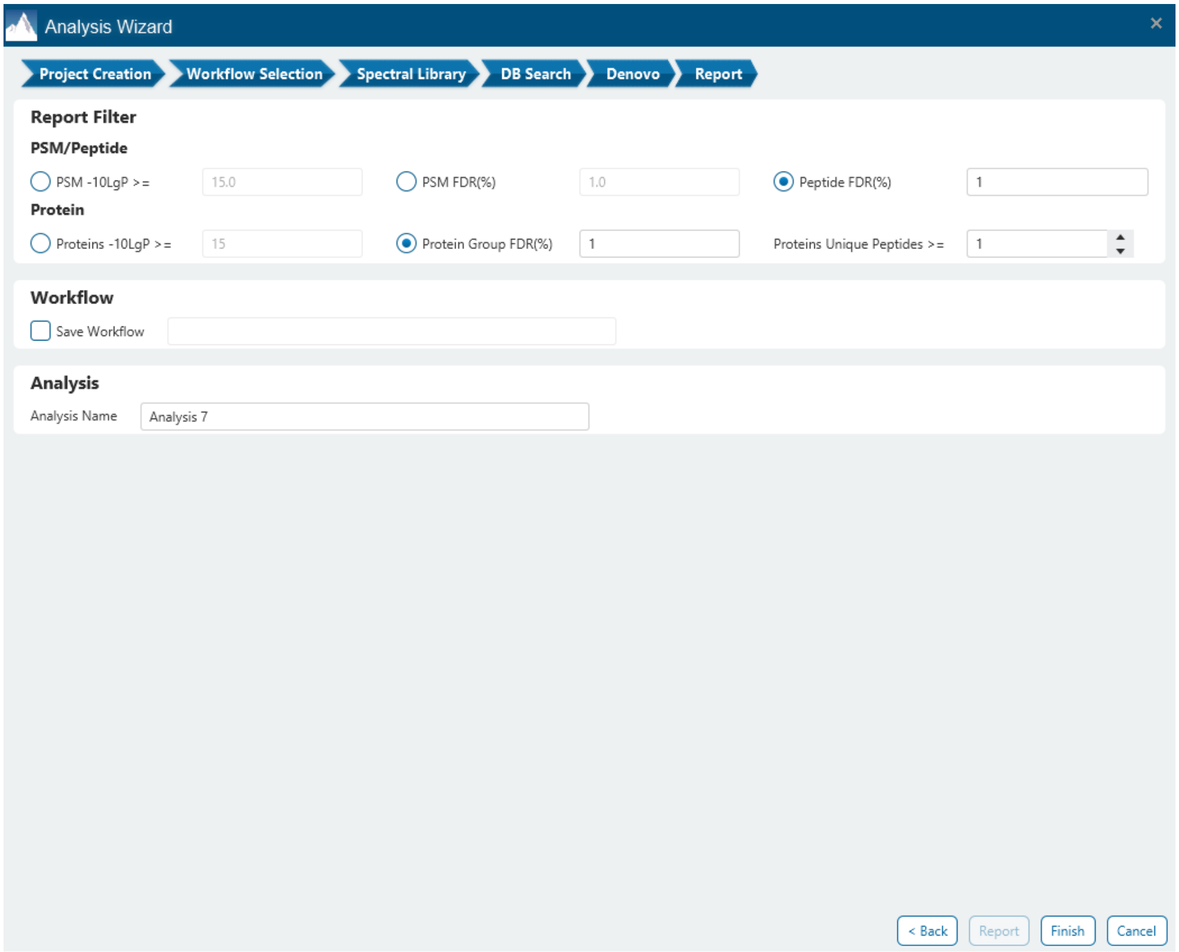

These filters are provided:

PSM/Peptide: this section is a mandatory section to filter out some results under the user-defined filter. Any changes to the filter will create a new analysis with old parameters and update the results and statistics.

PSM -10lgP: The PEAKS peptide score (-10lgP) is calculated for every peptide-spectrum match (PSM) reported by PEAKS DB, PEAKS PTM, and SPIDER. The score is derived from the p-value that indicates the statistical significance of the peptide-spectrum match. A peptide may be matched to many spectra, resulting in multiple PSMs. In that case, the peptide's score is calculated as the maximum among all PSM scores. For details of the scoring algorithm, please refer to the publication, *"PEAKS DB: De Novo sequencing assisted database search for sensitive and accurate peptide identification ", Mol Cell Proteomics, 2011 Dec 20.* A minimum requirement can be set and all identifications must pass this filter.

PSM FDR (%): The PSM filter can also be set to use the false discovery rate. FDR is estimated using a "decoy-fusion" approach.

Peptide FDR (%): The Peptides can also be set to use the false discovery rate. Peptides are scored based on their top PSM and using a “decoy-fusion” approach, the result is reported at that FDR.

Note: Decoy fusion is an enhanced target-decoy method for result validation with FDR. Decoy fusion appends a decoy sequence to each protein as the "negative control" for the search. See BSI's web tutorial (http://www.bioinfor.com/fdr-tutorial/) for more details.

From here, there is the option to specify an Analysis Name (which can be later renamed) and/or decide to save the Workflow to quickly re-use in future analyses.

7.2 Understanding PEAKS Spectral Library Search Results

After the library searches are complete, library result nodes are generated. Double-click the 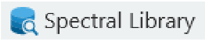

node to examine the analysis report. The analysis report is presented in three pages:

- Summary: This shows an outline of the library search results with key statistics. The overall quality of the experiment can be examined and the filters for peptide and protein identifications can be adjusted.
- Protein: This shows a list of protein identifications. This view also visualizes protein sequence coverage and helps with protein characterization. This tab only appears if Protein Inference was enabled for the PEAKS Spectral Library Search.
- Peptide: This shows a list of peptide identifications. This view also provides spectrum annotation and other detailed information for peptide precursor spectrum matches.

7.3 Spectral Library - Summary View

The Library result **Summary View** is similar to the PEAKS database identification result.

The Summary view reports key statistics as an overview of the result. In the report, several statistical charts can be examined to assess result quality. At the top of each summary result page, protein filters can be set. Identifications that fall below the filters will not be visible in the result pages or exports.

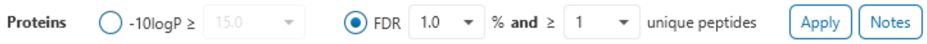

Proteins -10lgP: The PEAKS protein score (-10lgP) is calculated as the weighted sum of the -10lgP scores of the protein's supporting peptides. After removing any redundant peptides, the supporting peptides are sorted by -10lgP scores in descending order, and the k-th ranked peptide contributes to the weighted sum with a weight of 1/k. A default threshold of 15 is set.

Proteins FDR: The Proteins can also be filtered in accordance to the false-discovery rate of the protein set.

Proteins Unique Peptides: The minimum number of unique supporting peptides for a protein identification. A unique peptide is defined as a peptide that can be mapped to only one protein group.

After changing the filters, click 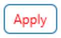

to apply the new filter and update the results accordingly.

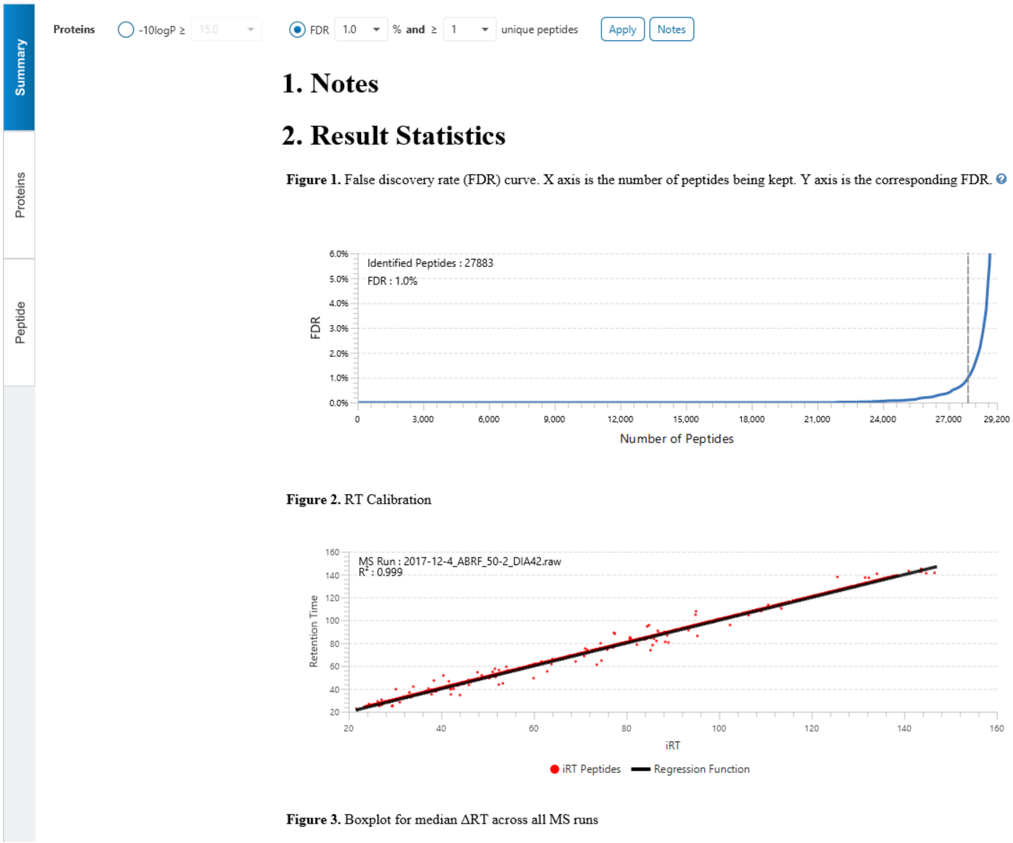

Click on Notes to open the Notes dialog to save useful information regarding this Analysis. The Notes will appear in Section 1 of the summary page at the top.

The Summary view reports key statistics as an overview of the result. In the report, several statistical charts can be examined to assess result quality.

7.3.1 Spectral Library result - Figure 1. False Discovery Rate (FDR) curve.

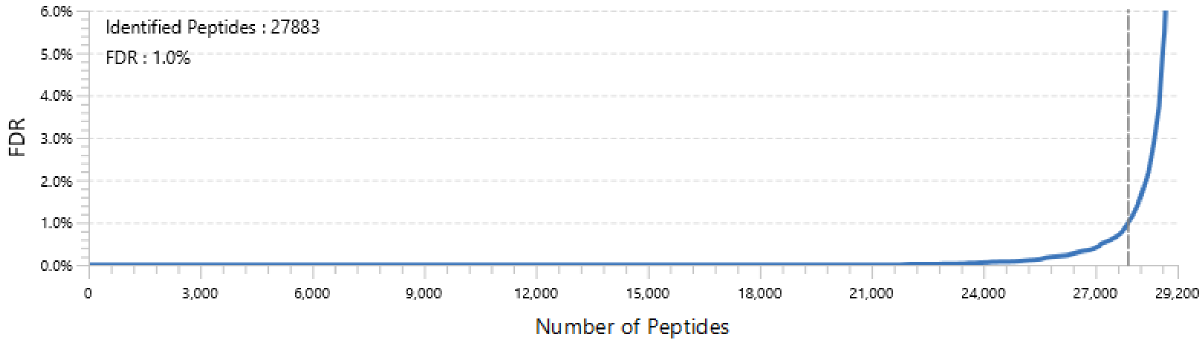

Figure 1 shows the FDR curve with respect to the number of Peptides (or PSMs) being kept after filtration by the -10lgP score. By lowering the score threshold, more Peptides are kept in the filtered result. Conversely, the FDR increases because more false positives are kept. In this figure, the vertical line indicates the current score threshold. The number of Peptides and the corresponding FDR by the current score threshold are shown in the top-left corner. PEAKS Search estimates FDR using the "decoy-fusion" approach. Decoy-fusion is an enhanced

target-decoy approach that makes more conservative FDR estimations. For details of the "decoy-fusion" approach, please referto the publication, *"PEAKS DB: De Novo sequencing assisted database search for sensitive and accurate peptideidentification", Mol Cell Proteomics, 2011 Dec 20.*

It is commonly recommended to set FDR at <1% by adjusting the score threshold. If a rapid growth of the FDR around the 1% FDR threshold is noticed, some PSMs may be sacrificed to significantly reduce the FDR.

Note: When counting the number of PSMs, PEAKS Studio keeps one peptide per spectrum at most. Thus, the number of PSMs is actually the number of spectra with assigned peptides.

Note: During Analysis creation, if Peptide FDR (%) filter was used in Report step, the Summary page FDR curve in the results will show the Peptide FDR curve and corresponding number of Identified peptides.

Note: Decoy matches are not counted in the number of PSMs. Unless otherwise specified, decoy matches are also excluded from the other statistical numbers shown in the Summary view.

7.3.2 Spectral Library result - Figure 2. RT Calibration

Figure 2. RT Calibration

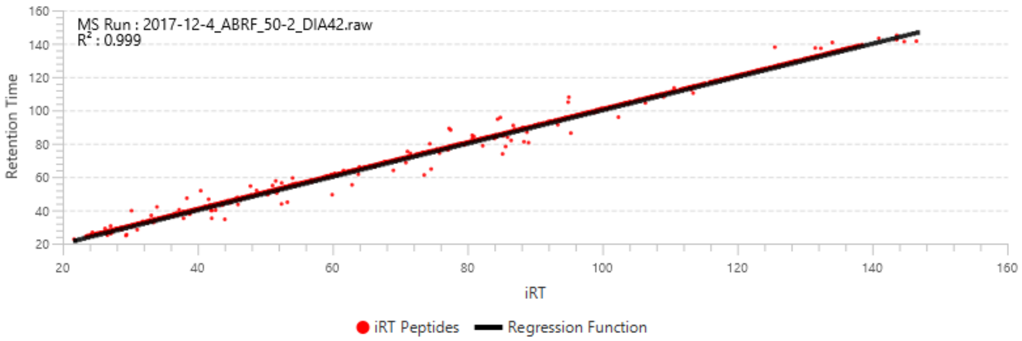

Plots the RT regression function used during the spectral library search. Peptides from the first MS Run in the analysis are plotted against their indexed retention time (iRT) predicted using machine learning. The R^2 value is presented based on the regression function.

7.3.3 Spectral Library result - Boxplot for RT change

Figure 3. Boxplot for median ΔRT across all MS runs

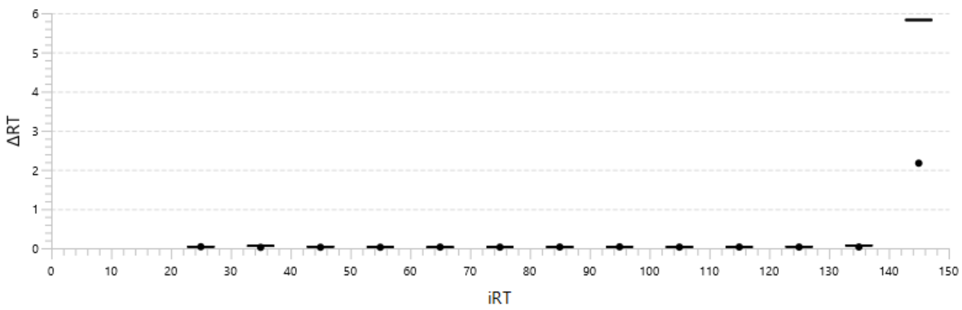

A box plot showing the difference between the observed retention time and iRT peptides within set retention time bins across all fractions, showing the accuracy of retention time prediction.

7.3.3 Spectral Library result - Table 1. Statistics of data

Statistics of Data tabulates MS1 and MS2 information, as well as identification in the peptide and protein level. The protein level statistics will only be presented if protein inference was performed. This table in the Summary view provides a condensed statistical information for the analysis.

Table 1. Statistics of data.

	#MS Run	#Scans		#PSMs	#Peptides	#Sequences	#Proteins			#PSM/#MS2
		MS1	MS/MS				Groups	All	Top	
Total	2	4678	196476	59460	27883	27104	3973	4314	4047	30.3%
Sample 1	1	2339	98238	29541	26931	26225	3967	4307	4041	30.1%
Sample 2	1	2339	98238	29919	27277	26552	3971	4309	4045	30.5%

#MS Run: Total # of runs in each sample.

#MS1: Total # of MS1 spectra for each sample. For timsTOF data this shows the # MS1 frames.

#MS/MS: Total # of MS2 spectra for each sample. This does not include chimeric scans. For timsTOF data this shows the # MS2 frames.

#PSMs: The number of peptide-spectrum matches.

#Peptides: The unique number of peptide sequences with modifications.

#Sequences: The unique number of peptide sequences not including modifications.

#Proteins Groups: PEAKS Studio groups proteins identified by a common set of peptides. This number shows the number of protein groups in the filtered result based on All proteins.

#All Proteins: Total number of proteins with significant peptides, according to current protein filters.

#Top Proteins: Total number of top proteins with significant peptides, according to current protein filters.

#PSM/#MS2: The identification rate by dividing #PSM by #MS2 for the sample.

7.3.4 Spectral Library result - Result filtration parameters

Tables 2-4 show the statistical numbers of the data and results.

Table 2. Result filtration parameters.

Peptide -10lgP	≥ 0
Protein -10lgP	≥ 35.9
Proteins unique peptides	≥ 1

Table 3. Statistics of filtered result.

FDR (Peptide-Spectrum Matches)	0.7%
FDR (Peptide Sequences)	1.0%
FDR (Protein Group)	1.0%

Table 4. PTM profile.

Name	ΔMass	Position	#PSM	#Peptides	-10lgP	Area	AScore
Carbamidomethyl	57.02	C	6310	2874	45.13	1.7E7	
Deamidation	.98	NQ	1664	775	45.09	4.78E6	
Oxidation	15.99	M	1452	690	45.07	4.92E6	

Table 2. Result filtration parameters: Table 2 summarizes the result filtration parameters set at the top of the Summary View page. This information provides a helpful reference for exported data.

Table 3. Statistics of filtered results: Table 3 presents the statistics based on the data analysis results after setting the desired filters. See below for further explanation:

- **FDR (Peptide-Spectrum Matches):** The total number of decoy database assignments to spectra relative to the total number of target database assignments to spectra represented as a percentage.
- **FDR (Peptide Sequences):** The total number of decoy database assignments to unique peptide sequences relative to the total number of target database assignments to unique peptide sequences represented as a percentage.
- **FDR (Protein Group):** The total number of decoy protein groups where the top hit in the protein group is a decoy database protein relative to the total number of target database protein groups where the top hit is from the target database.

Table 4. PTM Profile: shows modifications identified in the filtered result and the number of PSMs containing each modification

7.3.5 Spectral Library result - Experiment Control

Figure 4 in the Summary View show the precursor mass errors of PSMs in filtered results. The precursor mass error is calculated in ppm as 10^6 × (precursor mass - peptide mass) / peptide mass. Calibration is for display purposes only and PEAKS corrects m/z value for each PSM to correct systematic biases and instrument measurement drift.

Figure 4. Distribution of precursor mass error of filtered PSM in ppm.

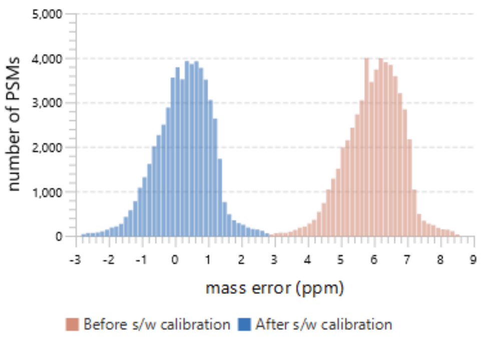

Table 5 shows the number of identified peptides in each sample with the number of missed cleavages, indicating the enzyme digestion efficiency.

Table 5. Number of identified peptides in each sample by the number of missed cleavages.

Missed Cleavages	0	1	2	3	4+
Sample 1	26015	878	32	6	0
Sample 2	26352	888	31	6	0

7.4 Spectral Library result - Proteins View

The Proteins View is only displayed if Protein Inference was selected in Spectral Library parameter step (shown below) in Project Wizard. It will automatically be selected if DIA Database Search was performed. It will also have to be performed if running DIA Quantification workflow.

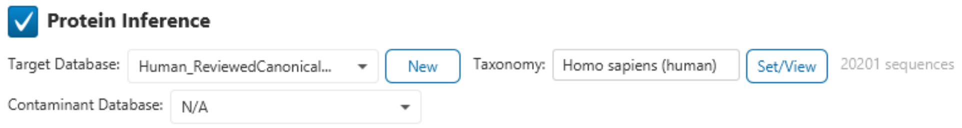

The Protein View lists protein identifications that have been filtered by the current settings in Summary View. It also visualizes the protein sequence coverage for identified proteins. The view has four main components:

- Protein table: This lists protein identifications that have been filtered by the current settings in the Summary View and the "Protein View Filters".
- Coverage: This visualizes the protein sequence coverage of the selected protein, showing the mapping of support peptides and de novo tags to the protein sequence.
- Peptides: This lists supporting peptides mapped to the selected protein.

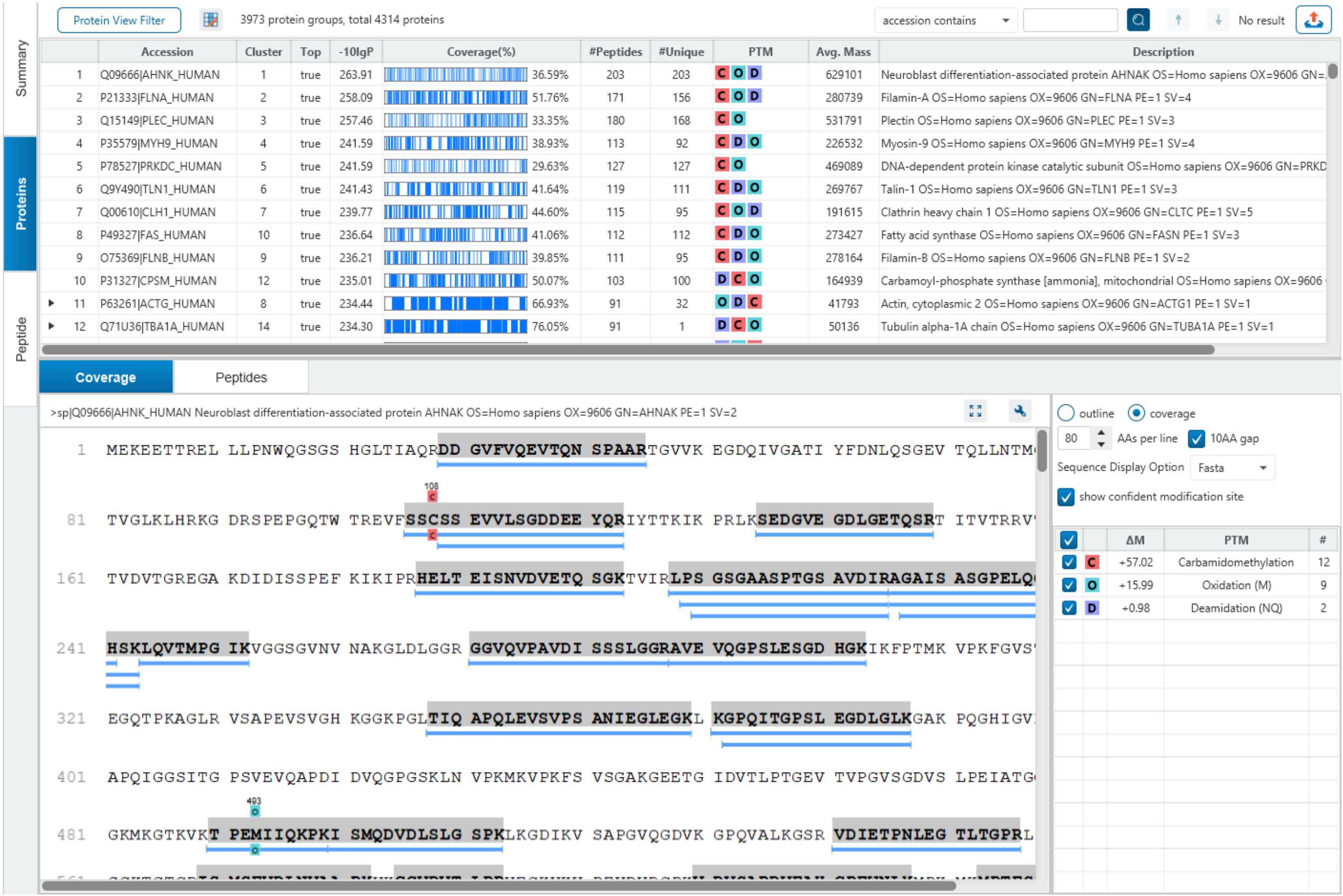

7.4.1 Proteins View - Protein Table controls

At the top of the Proteins View, there are the options to filter, show/hide columns, search, and export the protein table.

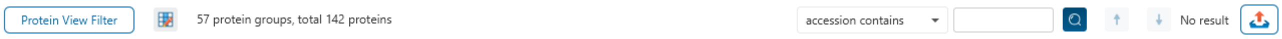

7.4.1.1 Protein Table controls - Protein View Filters

Click on the [Protein View Filter] button to open the Protein View Filter.

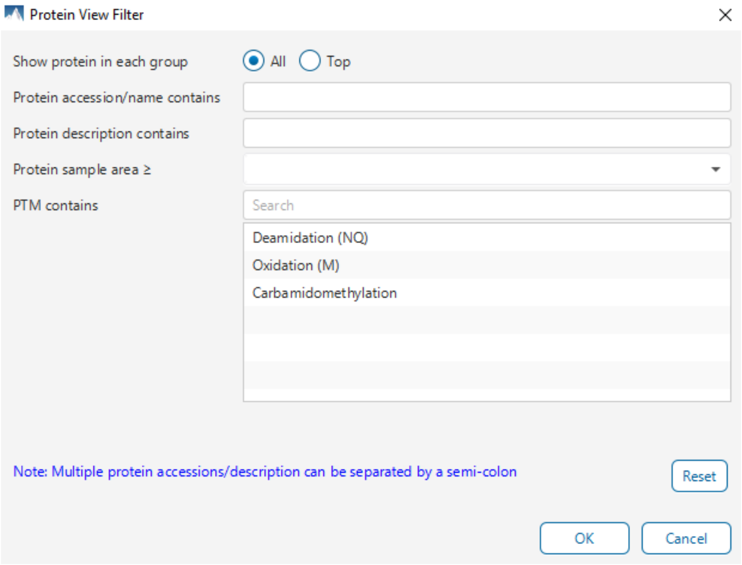

The following options are available for filtering the protein table:

Show Top, All, or First proteins in each group: Adjust the protein list based on Protein grouping, top is selected by default. Proteins are grouped based on parsimony.

- **Top:** Show only the top proteins in each group. These proteins are supported by the most unique peptides in the group. Proteins in the group that share a subset of the unique peptides that support the top protein (subproteins) will not be displayed.
- **All:** Show all proteins in each group. Proteins are grouped together if they are supported by the same set or a sub-set of the top protein in the group. If a protein is supported by a peptide not supporting the top protein it will be added to a new group.

Protein accession/name contains: Restrict the protein table to only display protein IDs which contain the entered text in its accession/name.

Protein description contains: Only proteins with descriptions that contain the entered text will be included in the protein table if text is entered in this field.

Note: In these above two filters, multiple entries can be separated by a semi-colon.

Protein sample area: Filter proteins based on total protein area found in each sample. Sample areas are calculated using the total of all peptide features from unique supporting peptides. If a protein is identified and has areas from multiple samples, only one of the samples must pass this filter to be included.

PTM contains: Filter protein results by the presence of selected PTMs. Proteins that are supported by peptides containing modifications that match the selected PTMs will be included. If multiple PTMs match the modification name, they will all be included in the table. For SPIDER results, the mutations will also be listed in the PTM contains filter.

Click on the Reset button to remove all filters.

Click on OK to apply the Protein View Filter to the Protein table.

Click on Cancel to discard changes and close the dialog.

7.4.1.2 Protein Table controls - Protein count

At the top of the protein table shows the count of protein groups and number of proteins in the table based on the current Protein View Filters.

57 protein groups, total 142 proteins

7.4.1.3 Protein Table controls - Optional columns

Click on the button to see a list of sample-based optional columns.

Select the corresponding sample-based Coverage, Spectra count, or Area to add into the Protein Table view.

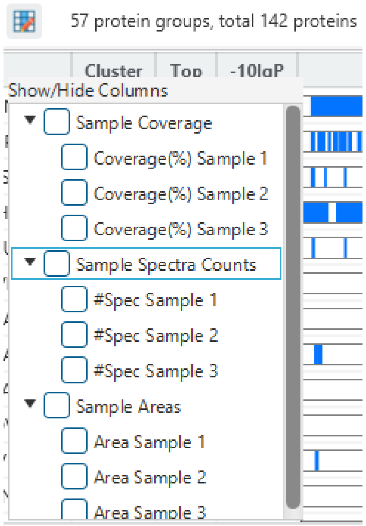

- **Coverage by Sample:** The protein coverage as a percentage will be displayed for the individual sample when selected.
- **#Spec by Sample:** The total number of spectra identified that support the given protein. Totals are given for each sample included in the search.
- **Area by Sample:** The total area of peptide features from unique supporting peptides in each sample will be displayed.

The exports will always include the contents of these optional columns even if they are hidden.

7.4.1.4 Protein Table controls - Protein Table Search Function

An easy-to-use search function is available for the protein table. The protein accession or description substring can be searched for, and all search results can be navigated through with the Up and Down arrows.

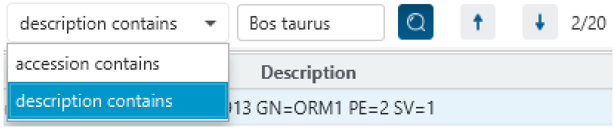

Protein accession/name contains: Restrict the protein table to only display protein IDs which contain the entered text in its accession/name.

Protein description contains: Only proteins with descriptions that contain the entered text will be included in the protein table if text is entered in this field.

7.4.1.5 Protein Table controls - Protein Table Export

Click on the button to export the contents of the current table. This considers the Protein View Filters, the sorting applied to the table and is consistent with the view. There is the option to export the Protein table and/or the Supporting peptide table in CSV format.

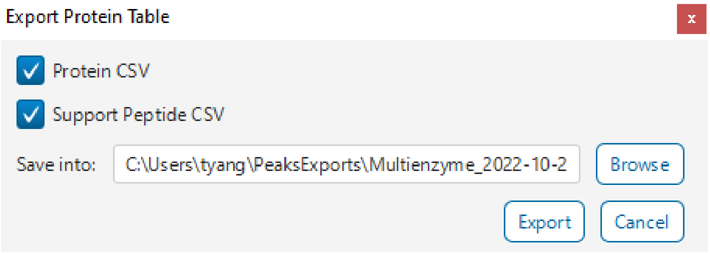

7.4.2 Proteins View - Protein Table

Each row in the table represents a group of proteins that are supported by a common set of peptides. To expand the group, click the right arrow button at the left.

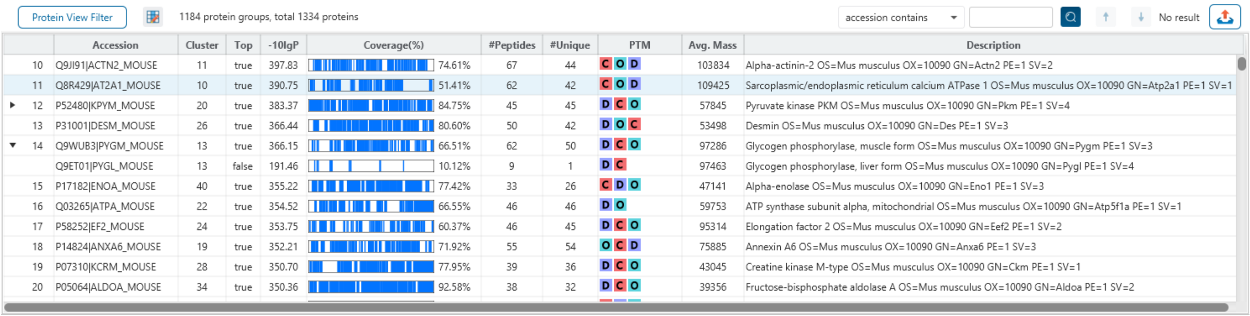

	Accession	Cluster	Top	-10lgP	Coverage(%)	#Peptides	#Unique	PTM	Avg. Mass	Description
10	Q9JI91|ACTN2_MOUSE	11	true	397.83	74.61%	67	44	C O D	103834	Alpha-actinin-2 OS=Mus musculus OX=10090 GN=Actn2 PE=1 SV=2
11	Q8R429|AT2A1_MOUSE	10	true	390.75	51.41%	62	42	C O D	109425	Sarcoplasmic/endoplasmic reticulum calcium ATPase 1 OS=Mus musculus OX=10090 GN=Atp2a1 PE=1 SV=1
▸ 12	P52480|KPYM_MOUSE	20	true	383.37	84.75%	45	45	D C O	57845	Pyruvate kinase PKM OS=Mus musculus OX=10090 GN=Pkm PE=1 SV=4
13	P31001|DESM_MOUSE	26	true	366.44	80.60%	50	42	D O C	53498	Desmin OS=Mus musculus OX=10090 GN=Des PE=1 SV=3
▾ 14	Q9WUB3|PYGM_MOUSE	13	true	366.15	66.51%	62	50	D C O	97286	Glycogen phosphorylase, muscle form OS=Mus musculus OX=10090 GN=Pygm PE=1 SV=3
	Q9ET01|PYGL_MOUSE	13	false	191.46	10.12%	9	1	D C	97463	Glycogen phosphorylase, liver form OS=Mus musculus OX=10090 GN=Pygl PE=1 SV=4
15	P17182|ENOA_MOUSE	40	true	355.22	77.42%	33	26	C D O	47141	Alpha-enolase OS=Mus musculus OX=10090 GN=Eno1 PE=1 SV=3
16	Q03265|ATPA_MOUSE	22	true	354.52	66.55%	46	46	D O	59753	ATP synthase subunit alpha, mitochondrial OS=Mus musculus OX=10090 GN=Atp5f1a PE=1 SV=1
17	P58252|EF2_MOUSE	24	true	353.75	60.37%	46	45	D C O	95314	Elongation factor 2 OS=Mus musculus OX=10090 GN=Eef2 PE=1 SV=2
18	P14824|ANXA6_MOUSE	19	true	352.21	71.92%	55	54	O C D	75885	Annexin A6 OS=Mus musculus OX=10090 GN=Anxa6 PE=1 SV=3
19	P07310|KCRM_MOUSE	28	true	350.70	77.95%	39	36	D C O	43045	Creatine kinase M-type OS=Mus musculus OX=10090 GN=Ckm PE=1 SV=1
20	P05064|ALDOA_MOUSE	34	true	350.36	92.58%	38	32	D C O	39356	Fructose-bisphosphate aldolase A OS=Mus musculus OX=10090 GN=Aldoa PE=1 SV=2

The columns in the Protein Table are:

- **Accession:** The accession number of the protein as seen in the FASTA database.
- **Cluster:** A unique identifier that classifies which protein group the protein belongs to.
- **Top:** Indicates whether this protein is a top protein.
- **-10lgP:** The protein confidence score.
- **Coverage (%):** The percentage of the protein sequence that is covered by supporting peptides. The coverage is visualized by a colored bar. Light blue blocks indicate the parts of the sequence covered by low-confidence peptides. Dark blue blocks indicate the parts covered by high-confidence peptides. This is the total coverage including results from all samples.

- **#Peptides:** The number of high-confidence supporting peptides.
- **#Unique:** The number of high-confidence supporting peptides that are mapped to only one protein group. Unique peptides with same sequence but different modifications are only counted once in this number.
- **PTM:** The identified modifications displayed with color-coded icons.
- **Avg. Mass:** The protein mass calculated using the average mass.
- **Description:** The protein's header information as seen in the FASTA database.

Note: For #Peptides and #Unique, two peptides with the same starting and ending positions in the protein are counted as one, regardless of their PTM forms. This is to follow the MCP (Molecular & Cellular Proteomics) guidelines.

7.4.3 Proteins View - Coverage Pane

The Protein Coverage view visually maps the supporting peptides and de novo tags to the protein selected in the Protein table. It also shows all identified sites with modifications or mutations to assist with protein characterization at the amino acid level. The "Protein Coverage" view is composed of three major components - Protein Sequence Coverage, Coverage Control Panel, and Protein Tools.

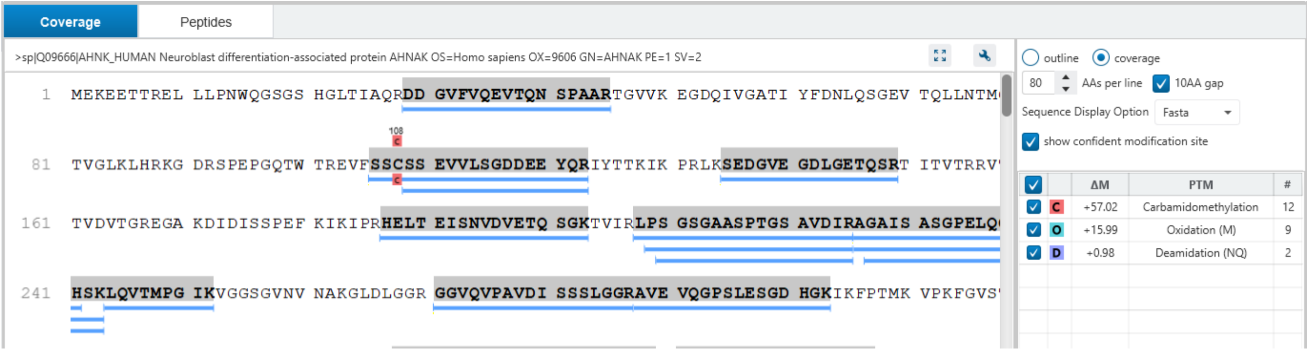

7.4.3.1 Coverage Pane - Protein Sequence Coverage

This area visualizes the coverage of the protein sequence.

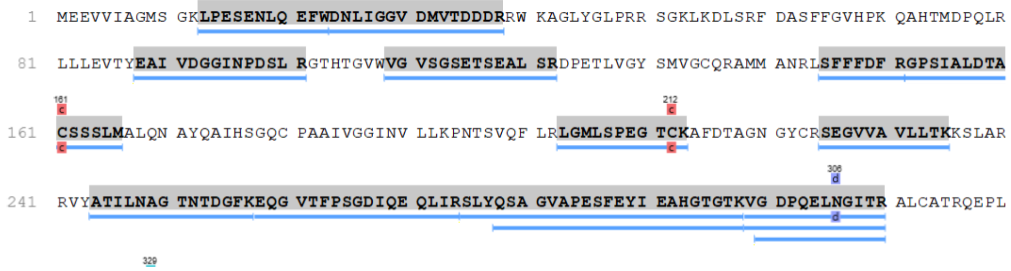

Regions in the protein sequence that are covered by supporting peptides are displayed in bold font with a grey background. Confident modifications and mutations identified in supporting peptides are displayed as icons above the protein sequence. Modifications are represented by colored icons with the first letter of its modification name. If a residue is modified by more than one modification in the same supporting peptide, "*" is used instead of a letter. The header information of the protein is shown on the top of the protein sequence.

The supporting peptides can be shown as colored bars under the protein sequence.

Placing the cursor over a bar shows a tooltip with detailed information of the peptide. Left clicking on a bar shows the annotated spectrum from which the supporting peptide is identified.

Right clicking on a peptide bar shows additional options. “Show in spectrum” has the same behaviour as left clicking on the peptide bar which is to open the annotated spectrum chart. “Show in table” has the function to jump to the supporting peptides table.

7.4.3.2 Coverage Pane - Coverage Control Panel

This panel controls what to display in the protein sequence coverage view.

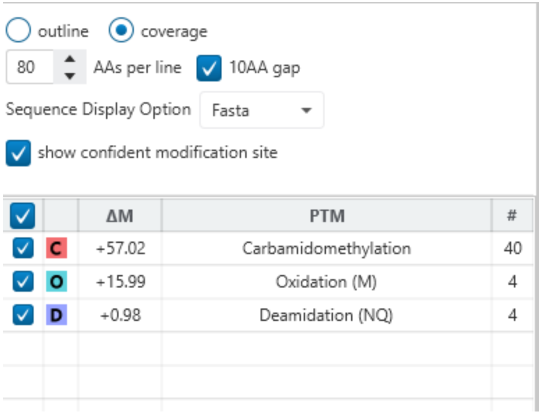

Sequence Display Mode: The protein sequence can be displayed in either "outline" or "coverage" mode.

Outline mode: In outline mode, the protein sequence is displayed without supporting peptides and matched de novo tags. However, the sequence coverage at a specific position can be examined by left-clicking a residue.

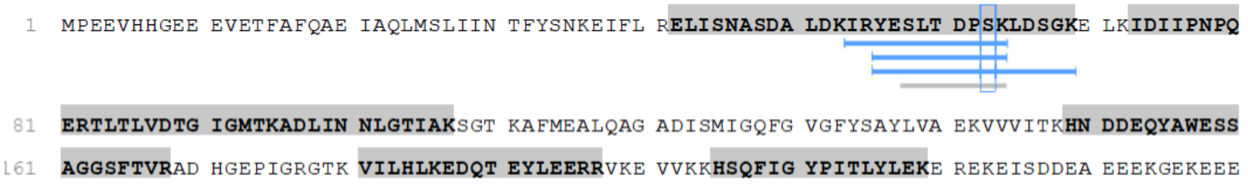

Coverage mode: In coverage mode, all the supporting peptides and matched de novo tags are shown.

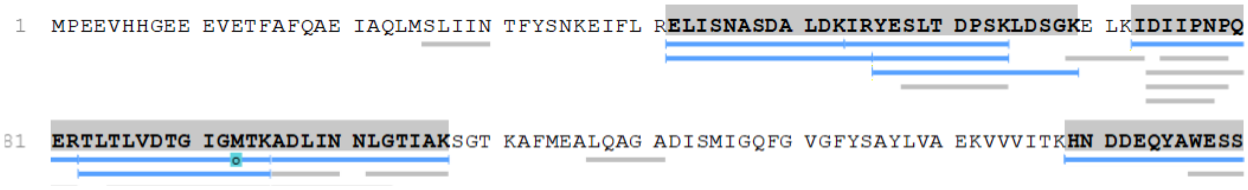

AAs per line and 10AA gap: Specify the number of amino acids per line and whether to show a gap for every 10 amino acids in the protein sequence.

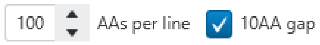

Sequence Display Option: Select whether to view the protein sequence as Fasta or a specific enzyme used in the project. When a particular enzyme is selected, the protein sequence will be highlighted to emphasize expected peptides generated by the selected enzyme's cleavage specificity.

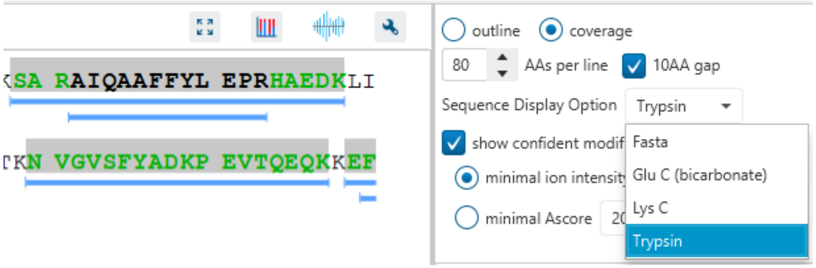

Modification table: The modification table shows the modifications identified in supporting peptides on the protein. For each modification, the number of supporting PSMs with this particular modification is shown. The checkbox on the left controls whether to indicate the modification in the protein coverage.

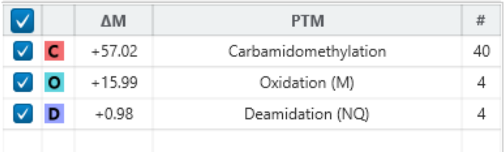

☑		ΔM	PTM	#
☑	C	+57.02	Carbamidomethylation	40
☑	O	+15.99	Oxidation (M)	4
☑	D	+0.98	Deamidation (NQ)	4

7.4.3.3 Proteins View - Protein Tools

The Proteins View toolbar is at the upper-right corner of the protein sequence coverage tab and contains helpful protein tools.

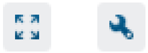

Full screen: When selected, the protein coverage window expands in full screen mode. Click to return from full screen mode.

Tool box: The tool box contains the following tools:

- Copy protein sequence. This copies the protein sequence of the current selected protein into the system clipboard.
- Save protein coverage as image. Different scale options are provided to increase the image size.
- Coverage statistics. This provides useful statistics based on the current selected protein.

7.4.3.4 Proteins View - Supporting Peptides

The "Peptides" tab displays a table of supporting peptides for the protein of interest. This table is similar to the peptide table in the **Peptide** view, except that "Accession" is excluded and the columns "(Checkbox)", "Unique", "Start", and "End" are included. Descriptions of extra columns are given in the following sections.

- **(Checkbox):** This controls whether a peptide is shown in the protein coverage as a blue bar.
- **Unique:** This shows whether the peptide is a unique supporting peptide to the protein group.
- **Start:** This shows the peptide's starting position in the protein.
- **End:** This shows the peptide's ending position (inclusive) in the protein.

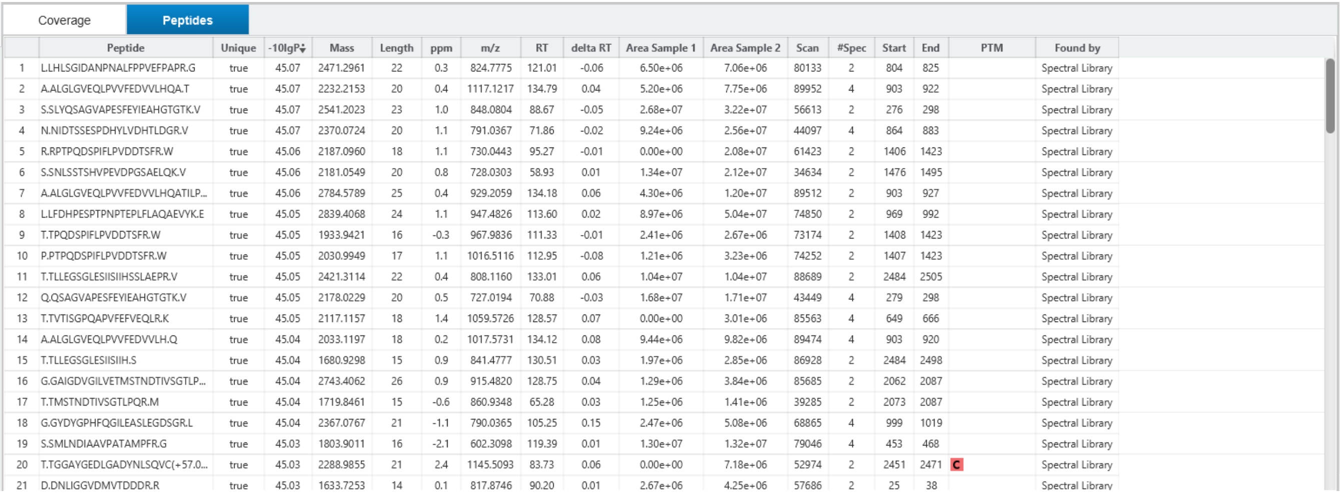

Coverage | Peptides

#	Peptide	Unique	-10lgP	Mass	Length	ppm	m/z	RT	delta RT	Area Sample 1	Area Sample 2	Scan	#Spec	Start	End	PTM	Found by
1	L.LHLSGIDANPNALFPPVEFPAPR.G	true	45.07	2471.2961	22	0.3	824.7775	121.01	-0.06	6.50e+06	7.06e+06	80133	2	804	825		Spectral Library
2	A.ALGLGVEQLPVVFEDVVLHQA.T	true	45.07	2232.2153	20	0.4	1117.1217	134.79	0.04	5.20e+06	7.75e+06	89952	4	903	922		Spectral Library
3	S.SLYQSAGVAPESFEYIEAHGTGTK.V	true	45.07	2541.2023	23	1.0	848.0804	88.67	-0.05	2.68e+07	3.22e+07	56613	2	276	298		Spectral Library
4	N.NIDTSSESPDHYLVDHTLDGR.V	true	45.07	2370.0724	20	1.1	791.0367	71.86	-0.02	9.24e+06	2.56e+07	44097	4	864	883		Spectral Library
5	R.RPTPQDSPIFLPVDDTSFR.W	true	45.06	2187.0960	18	1.1	730.0443	95.27	-0.01	0.00e+00	2.08e+07	61423	2	1406	1423		Spectral Library
6	S.SNLSSTSHVPEVDPGSAELQK.V	true	45.06	2181.0549	20	0.8	728.0303	58.93	0.01	1.34e+07	2.12e+07	34634	2	1476	1495		Spectral Library
7	A.ALGLGVEQLPVVFEDVVLHQATILP...	true	45.06	2784.5789	25	0.4	929.2059	134.18	0.06	4.30e+06	1.20e+07	89512	2	903	927		Spectral Library
8	L.LFDHPESPTPNPTEPLFLAQAEVYK.E	true	45.05	2839.4068	24	1.1	947.4826	113.60	0.02	8.97e+06	5.04e+07	74850	2	969	992		Spectral Library
9	T.TPQDSPIFLPVDDTSFR.W	true	45.05	1933.9421	16	-0.3	967.9836	111.33	-0.01	2.41e+06	2.67e+06	73174	2	1408	1423		Spectral Library
10	P.PTPQDSPIFLPVDDTSFR.W	true	45.05	2030.9949	17	1.1	1016.5116	112.95	-0.08	1.21e+06	3.23e+06	74252	2	1407	1423		Spectral Library
11	T.TLLEGSGLESIISIIHSSLAEPR.V	true	45.05	2421.3114	22	0.4	808.1160	133.01	0.06	1.04e+07	1.04e+07	88689	2	2484	2505		Spectral Library
12	Q.QSAGVAPESFEYIEAHGTGTK.V	true	45.05	2178.0229	20	0.5	727.0194	70.88	-0.03	1.68e+07	1.71e+07	43449	4	279	298		Spectral Library
13	T.TVTISGPQAPVFEFVEQLR.K	true	45.05	2117.1157	18	1.4	1059.5726	128.57	0.07	0.00e+00	3.01e+06	85563	4	649	666		Spectral Library
14	A.ALGLGVEQLPVVFEDVVLH.Q	true	45.04	2033.1197	18	0.2	1017.5731	134.12	0.08	9.44e+06	9.82e+06	89474	4	903	920		Spectral Library
15	T.TLLEGSGLESIISIIH.S	true	45.04	1680.9298	15	0.9	841.4777	130.51	0.03	1.97e+06	2.85e+06	86928	2	2484	2498		Spectral Library
16	G.GAIGDVGILVETMSTNDTIVSGTLP...	true	45.04	2743.4062	26	0.9	915.4820	128.75	0.04	1.29e+06	3.84e+06	85685	2	2062	2087		Spectral Library
17	T.TMSTNDTIVSGTLPQR.M	true	45.04	1719.8461	15	-0.6	860.9348	65.28	0.03	1.25e+06	1.41e+06	39285	2	2073	2087		Spectral Library
18	G.GYDYGPHFQGILEASLEGDSGR.L	true	45.04	2367.0767	21	-1.1	790.0365	105.25	0.15	2.47e+06	5.08e+06	68865	4	999	1019		Spectral Library
19	S.SMLNDIAAVPATAMPFR.G	true	45.03	1803.9011	16	-2.1	602.3098	119.39	0.01	1.30e+07	1.32e+07	79046	4	453	468		Spectral Library
20	T.TGGAYGEDLGADYNLSQVC(+57.0...	true	45.03	2288.9855	21	2.4	1145.5093	83.73	0.06	0.00e+00	7.18e+06	52974	2	2451	2471	C	Spectral Library
21	D.DNLIGGVDMVTDDDR.R	true	45.03	1633.7253	14	0.1	817.8746	90.20	0.01	2.67e+06	4.25e+06	57686	2	25	38		Spectral Library

7.5 Spectral Library Result - Peptide View

The "Peptide View" displays the resulting peptide identifications that have been filtered by the current settings in Summary View and "Peptide View Filters". The table on the top shows the details of each peptide identification. The bottom section provides additional information about the peptide-spectrum matches for the native peptide selected in the table.

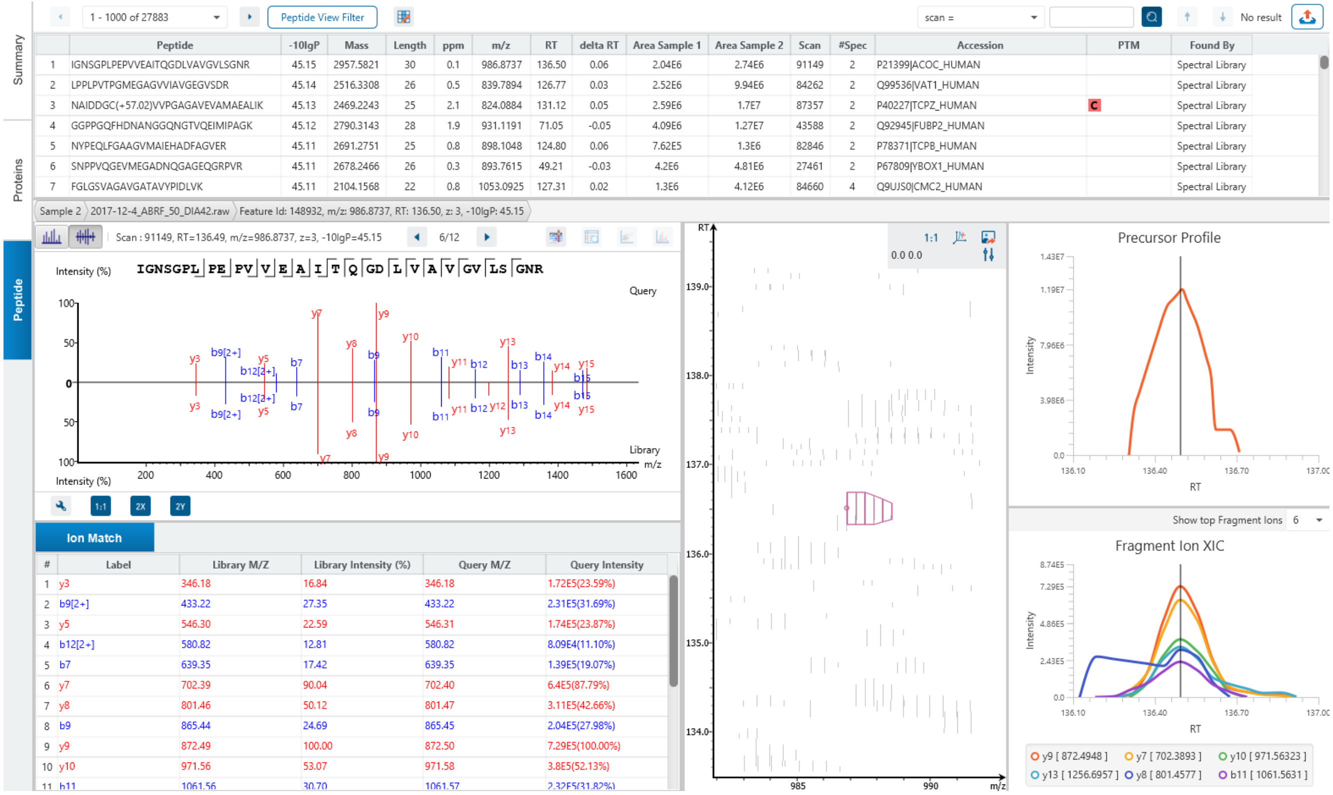

7.5.1 Peptide View - Peptide Table

The Peptide Table shows the filtered native peptide identification results. Each row in the table is a native peptide identification represented by its highest-scoring PSM. When there are more than 1000 peptides, the table is split into multiple pages.

Note: If there are multiple PSMs matched to a peptide, the table displays the information for the top-scoring PSM. Peptides with the same sequence but different modifications are considered different and shown in different rows.

To copy the sequence, right click on a row (or multiple rows) in the table and select “Copy selected peptide sequence(s)”. Another option is to mouse over any cell in the table and hitting Ctrl + C on your keyboard to copy the contents of those selected cells.

1 - 1000 of 27883 | Peptide View Filter | scan = | No result

	Peptide	-10lgP	Mass	Length	ppm	m/z	RT	delta RT	Area Sample 1	Area Sample 2	Scan	#Spec	Accession	PTM	Found By
1	IGNSGPLPEPVVEAITQGDLVAVGVLSGNR	45.15	2957.5821	30	0.1	986.8737	136.50	0.06	2.04E6	2.74E6	91149	2	P21399|ACOC_HUMAN		Spectral Library
2	LPPLPVTPGMEGAGVVIAVGEGVSDR	45.14	2516.3308	26	0.5	839.7894	126.77	0.03	2.52E6	9.94E6	84262	2	Q99536|VAT1_HUMAN		Spectral Library
3	NAIDDGC(+57.02)VVPGAGAVEVAMAEALIK	45.13	2469.2243	25	2.1	824.0884	131.12	0.05	2.59E6	1.7E7	87357	2	P40227|TCPZ_HUMAN	C	Spectral Library
4	GGPPGQFHDNANGGQNGTVQEIMIPAGK	45.12	2790.3143	28	1.9	931.1191	71.05	-0.05	4.09E6	1.27E7	43588	2	Q92945|FUBP2_HUMAN		Spectral Library
5	NYPEQLFGAAGVMAIEHADFAGVER	45.11	2691.2751	25	0.8	898.1048	124.80	0.06	7.62E5	1.3E6	82846	2	P78371|TCPB_HUMAN		Spectral Library
6	SNPPVQGEVMEGADNQGAGEQGRPVR	45.11	2678.2466	26	0.3	893.7615	49.21	-0.03	4.2E6	4.81E6	27461	2	P67809|YBOX1_HUMAN		Spectral Library
7	FGLGSVAGAVGATAVYPIDLVK	45.11	2104.1568	22	0.8	1053.0925	127.31	0.02	1.3E6	4.12E6	84660	4	Q9UJS0|CMC2_HUMAN		Spectral Library

The following list describes the contents in each of the default columns:

- **Peptide:** The amino acid sequence of the peptide, as determined by the PEAKS searchworkflow. A modified residue is followed by a pair of parentheses enclosing the modification.
- **-10lgP:** The peptide -10lgP score. The score indicates the scoring significance of a peptide-precursor spectrum match.
- **Mass:** The monoisotopic mass of the peptide.
- **Length:** The number of amino acids in the sequence backbone.
- **ppm:** The precursor mass error, calculated as 10^6 × (precursor mass - peptide mass) / peptide mass.
- **m/z:** The precursor mass-to-charge ratio.
- **RT:** The retention time (elution time) of the spectrum as recorded in the data.
- **delta RT:** The difference between the query and library RT.
- **Area per Sample:** The combined area for all features in that sample associated with this peptide.
- **Scan:** The scan number of the spectrum that matches the peptide sequence with the highest -10lgP.
- **#Spec:** number of MS2 spectra assigned to the peptide.
- **Accession:** The accessions of proteins that contain this peptide.
- **PTM:** The types and the numbers of modifications present in the peptide shown in color-coded icons.
- **Found By:** The name of the PEAKS search workflow that identifies the peptide; this can only be Spectral Library for this result.

7.5.2 Peptide View - Peptide Table controls

The menu above the peptide table provides options to scroll through the table of peptides, filter the table, show optional columns and search for specific entries.

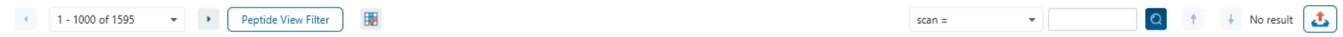

7.5.2.1 Peptide Table controls - Pagination

For easy viewing and to improve performance, the peptide table is limited to showing 1000 entries at once. Pagination is used to manage which set of 1000 entries will be viewed.

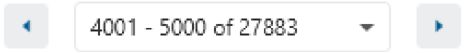

In the top left, there is a dropdown to select the rows for viewing. Click the left arrow or right arrow to select the previous/next 1000 rows. If the table is sorted or filtered, it may update the pagination set of rows that is currently in view.

7.5.2.2 Peptide Table controls - View Filter

Click on the 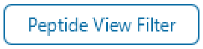

button to open the Peptide View Filter which is used to filter the Peptide table. The following are the filter options provided:

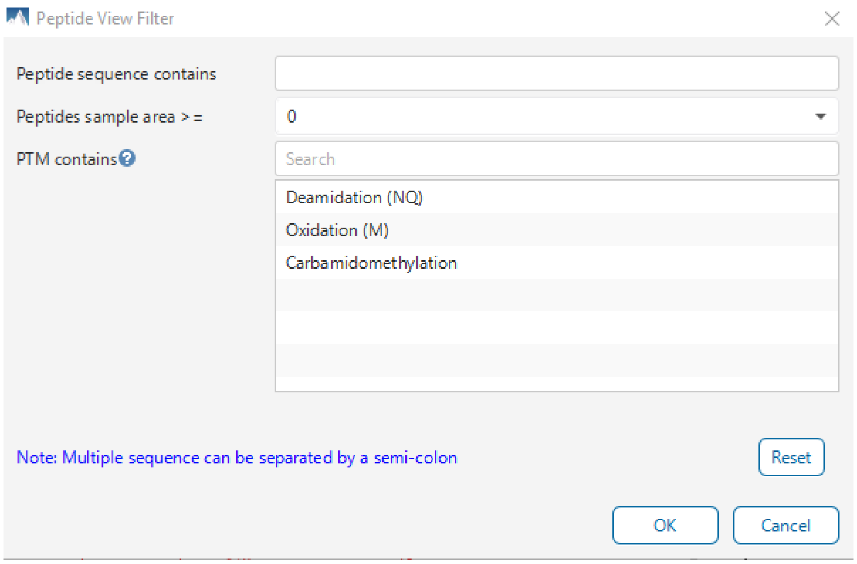

- Peptide sequence contains: Enter an amino acid sequence, only peptides that contain that sequence will be displayed in the peptide table. Adding PTMs in brackets in the sequence search is not handled, meaning only the sequence backbone is considered in the filter. Use the PTM contains for further filtering if necessary.
- Peptide sample area: Filters based on total peptide area found in each sample. Sample areas are calculated using the total of all peptide features from all spectra that identified the native peptide. If the peptide is found in multiple samples, only one sample must pass this filter for the peptide to be included.

- PTM contains: Type the names of PTMs you would like to include. Peptides containing PTMs that match the text will be included. If multiple PTMs match the text they will all be included in the table.

Note: In all filters, multiple entries can be separated by a semi-colon.

Click on the Reset button to reset all filters to their default setting.

Click on OK to apply the filters to the Peptide table.

Click on Cancel to discard the changes in the View Filter.

7.5.2.3 Peptide Table controls - Optional Columns

Click on the 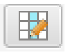 button to open the optional columns dropdown. By default the sample area columns are displayed. Sample Spec count columns can also be shown. These sample spec counts are always from identification, and does not consider whether there are feature vectors present. For projects with many samples, it might be useful to hide these optional columns and reduce the width of the peptide table.

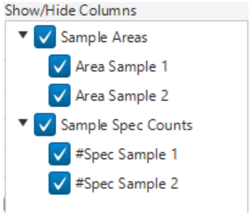

7.5.2.4 Peptide Table controls - Search function

On the top right of menu, there is a search function with 4 options. Search results can be navigated between using the Up or Down arrows.

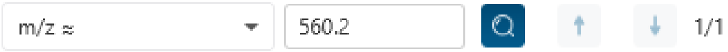

Options for searching include:

- **Scan =:** Search for the Scan number associated with each peptide. One scan can be associated to multiple peptides. For projects with multiple data files, the scan number is searched on every fraction.
- **m/z ≈:** Search for the approximate m/z. The search will consider a range of m/z equal to the search value +/- 1 of the last significant digit. For example, searching for m/z ≈ 560.21 will find all m/z between [560.20, 560.22]
- **RT ≈:** Search for the approximate RT. Likewise, the search will consider a range of RT equal to the search value +/- 1 of the last significant digit set. For example, searching for RT ≈ 55.5 will find all m/z between [55.4, 55.6]
- **Seq contains:** Search for the sequence in the table. If specifying a sequence without PTMs, the results will include that sequence and any sequences with PTMs with the same backbone. If specifying a sequence with bracket and some mass value (like in the above image), then only sequence that have a substring with an exact match will be considered.
  Additionally, since the peptide table shows both I/L and they can not be distinguished, then searching for I in the sequence is equivalent to searching for L in the sequence. i.e. Searching for “LVLK” is the same as searching for “LVIK”, “IVLK”, and “IVIK”

7.5.3 Peptide View - Feature selection breadcrumb

There is a sample and feature selection breadcrumb which allows you to switch between features associated with the peptide. Click on the Sample in the breadcrumb to reveal other samples and the features in that sample related to the current peptide selection. From here you can select other samples, fractions, or features.

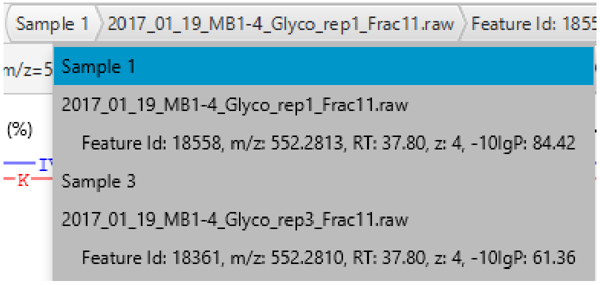

By clicking on the fraction level, all fractions in this sample are revealed and the features in each fraction associated with this peptide. From here you can select other fractions and features to view.

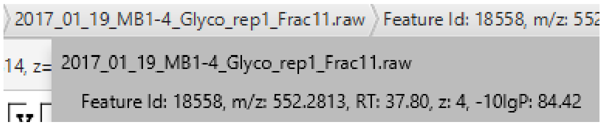

Lastly, clicking on the feature level will show all features in the currently selected fraction. Features with Feature Id = 0 are known as fake features and are constructed to enhance identification.

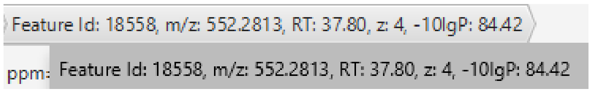

7.5.4 Peptide View - PSM selection

Underneath the feature breadcrumb will shown the currently selected PSM. Click on the Left and Right arrow buttons to switch between PSMs related to the currently selected feature.

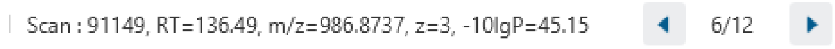

All other components in the Peptide tab interface will update according to the currently selected PSM.

For diaPASEF instruments, Frame is used rather than Scan number.

7.5.4.1 Peptide View - Protein jump button

Click on 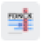 to jump to a protein that contains the currently selected PSM. If there are more than 1 protein, a dropdown is revealed which allows the user to select which protein to jump to. If a protein is filtered out due to a protein summary filter or summary page protein filter, then the option to jump will be grayed out. If there is no protein tab in the result, then the button will always be disabled.

7.5.5 Peptide View - Annotated Spectrum Chart

Click on button to switch from the Mirror plot view and annotated spectrum chart view.

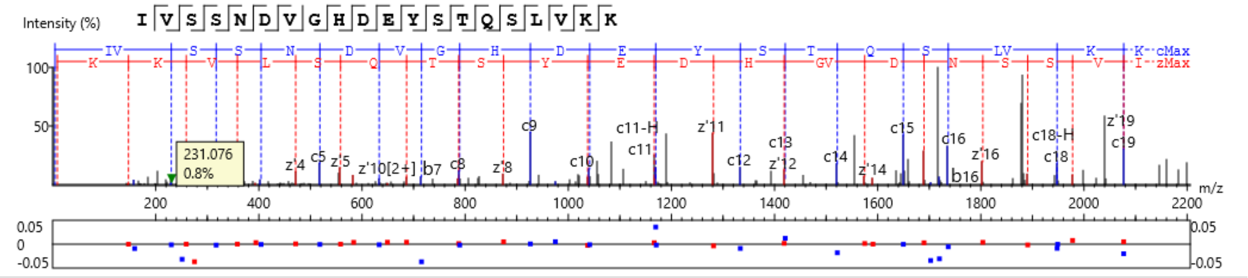

For the currently selected PSM, a graphical representation is shown in the form of an annotated spectrum chart. There are several controls provided for this view which will be explored in the following sections.

7.5.5.1 Annotated Spectrum Chart - Chart Navigation

Moving the cursor over the peptide sequence in the spectrum will show the mass transitions for a particular amino acid residue.

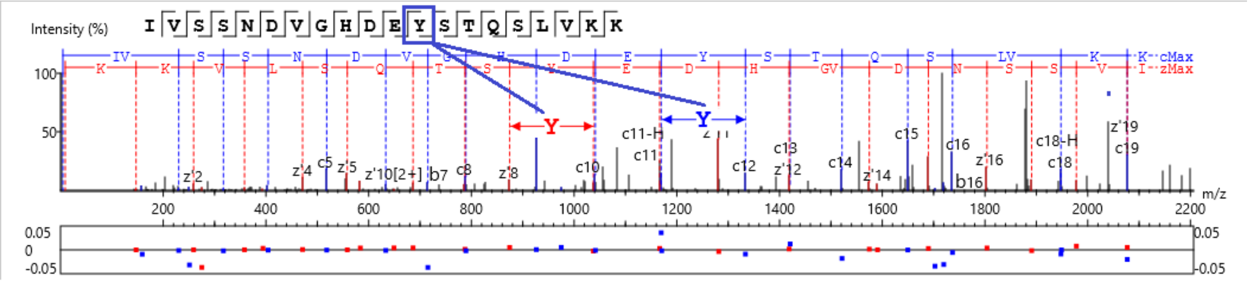

Zoom to a m/z region: Drag horizontally from the start m/z and to the end m/z with left mouse button.

Zoom in/out smoothly: Place the cursor at a particular m/z value (right below the x-axis), zoom in/out by scrolling the mouse wheel.

Increase/Decrease peak intensity: Place the cursor in the spectrum and scroll the mouse wheel.

See the whole spectrum: Double click in the spectrum or click the 1:1 button.

7.5.5.2 Annotated Spectrum Chart - Spectrum Annotation Settings

Click on the button to show the spectrum annotation settings.

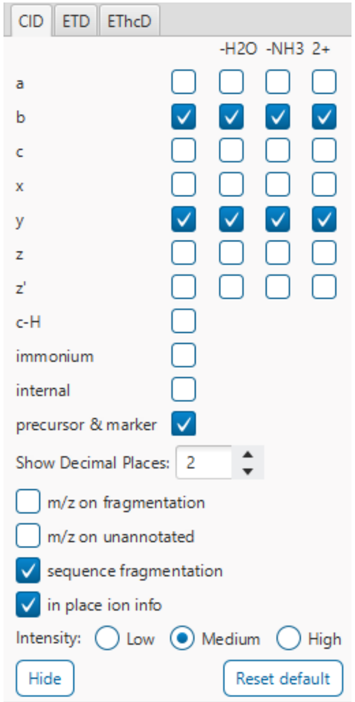

The spectra in the PEAKS results can be annotated by selecting ion types from the thorough collection of ions that PEAKS offers. The selected ion types will be displayed in the “Ion Match” table, as well. It is possible to annotate the spectrum with various ions for CID, EThcD and ETD. Each of these fragmentation modes can be set with different settings which will be shared with other results with the same fragmentation type.

To reset the defaults, click on the “Reset default” button.

Show Decimal Places: Select the number of decimal places that will appear in the ion table and the spectrum view. The default is set to two decimal places.

m/z on Fragmentation: Select this to display the m/z value on top of the annotated ions.

m/z on Unannotated: Select this to display the m/z value on top of the peaks without ion matches.

Sequence Fragmentation: Select this to display the sequence fragmentation on the top-left corner of the “Spectrum Annotation” view.

In Place Ion Info: Ion information, m/z value,and relative intensity are all displayed in a pop-up within the “Spectrum Annotation” view when this option is checked and the cursor is placed on a peak.

Intensity: Set the intensity threshold for spectrum annotation to low (2%), medium (5%), or high (10%).

7.5.5.3 Annotated Spectrum Chart - Additional Chart Controls

Click on 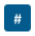

or 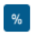

to toggle between absolute and percentage intensity values in the Y axis.

Click on 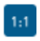

to reset the zoom level of the annotated spectrum chart to the default.

Click on 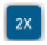

to zoom into the X axis by a factor of 2.

Click on 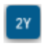

to zoom into the Y axis by a factor of 2.

The "alignment" check box alignment allows user to display/hide how the fragment ions generated from the peptide align with the spectrum. N-terminal ions are shown in blue and C-terminal ions are shown in red.

The “error map” check box error map is used to show/hide the error map underneath the chart.

7.5.5.4 Annotated Spectrum Chart - Mirror Plot

Spectral Library results have a Mirror Plot view. Click on the button to switch to the Mirror Plot.

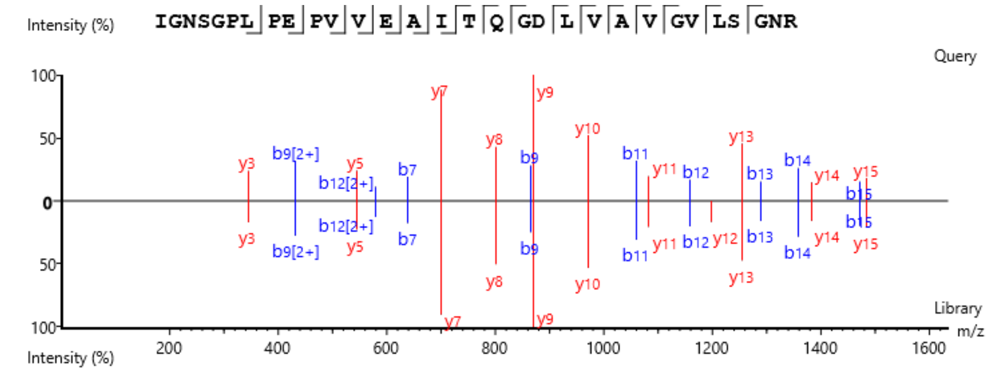

This view shows the ions from the query spectrum compared to the library spectrum.

7.5.6 Peptide View - Ion Match Table

The “Ion Match” tab in the bottom pane contains a table with possible fragment ions for the selected MS/MS scan. Each ion in the table shows the calculated mass, where if the fragment ion is present in the spectrum, its mass value is displayed in color. N-terminal ions are shown in blue and C-terminal ions are shown in red. A fragment ion is found when there is a matching peak within the mass error tolerance, as defined in the de novo sequencing parameters, and when relative intensity of the matching peak is at least 2%. The ion types displayed in the table can be configured in "Spectrum Annotation Settings”.

Ion Match | Survey

#	b	b-H2O	b-NH3	b(2+)	Seq	y	y-H2O	y-NH3	y(2+)	#
10	1079.503	1061.488	1062.498	540.248	E	1596.643	1578.636	1579.610	798.818	13
11	1265.576	1247.569	1248.557	633.292	W	1467.601	1449.588	1450.585	734.297	12
12	1394.626	1376.616	1377.600	697.813	E	1281.521	1263.512	1264.488	641.266	11
13	1481.659	1463.648	1464.632	741.329	S	1152.480	1134.469	1135.461	576.736	10
14	1596.682	1578.671	1579.655	798.841	N(+0.98)	1065.449	1047.440	1048.427	533.228	9
15	1653.703	1635.693	1636.676	827.352	G	950.422	932.407	933.390	475.709	8
16	1781.773	1763.751	1764.735	891.381	Q	893.397	875.388	876.370	447.198	7
17	1878.815	1860.804	1861.788	939.907	P	765.341	747.326	748.315	383.169	6
18	2007.857	1989.847	1990.830	1004.429	E	668.288	650.274	651.258	334.642	5
19	2122.880	2104.870	2105.853	1061.940	N(+0.98)	539.245	521.231	522.222	270.124	4
[illegible]	[illegible]	[illegible]	[illegible]	[illegible]	[illegible]	[illegible]	[illegible]	[illegible]	[illegible]	[illegible]

Note: Uncheck the alignment checkbox in the spectrum annotation view to see the highlighted fragment ions more easily.

7.5.6.1 Ion Match Table - Library matching table

While in mirror plot view, the Ion Match table updates to display the ion matches between fragment query ions and library ions.

Library intensity and Query intensity are reported relative to the highest intensity ion. All columns can be rearranged and sorted.

7.5.7 Peptide View - LC/MS Snapshot

The LC-MS Snapshot displays a zoomed-in view of the precursor scan in the LC-MS heat map 2-D view. The peptide feature is highlighted in purple and the selected PSM marker is blue. The span of the orange line in the LC-MS Snapshot designates the isolation window.

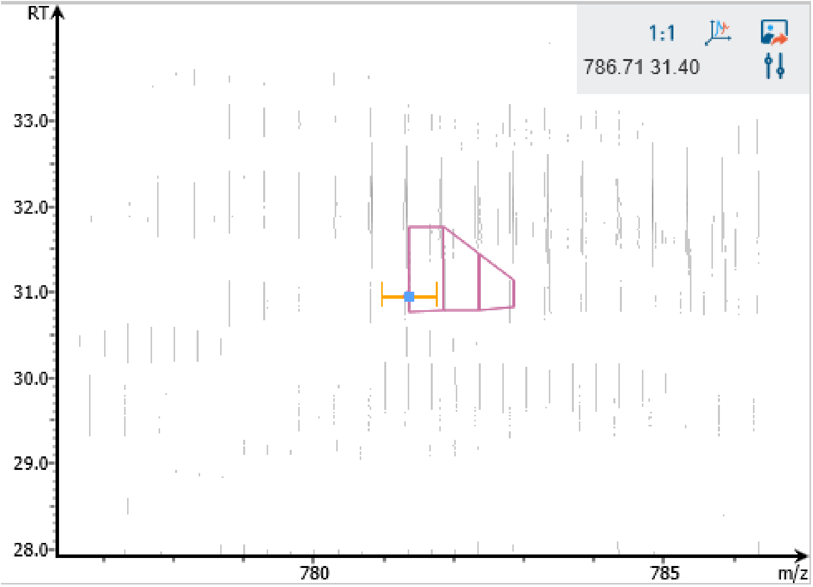

Users can change the view from the 2-D heat map to a 3-D intensity view by clicking the intensity view button in the upper right-hand corner of the LC-MS Snapshot. In addition, users can export the LC-MS heat map by clicking the export image button in the upper right-hand corner of the LC-MS Snapshot.

Expand the options dropdown by clicking to reveal a search option to specify a *m/z RT* (ensure there is a space between these two numbers) location to zoom into. There is also a Peak slider filter to filter out low intensity peaks and a feature area filter to filter out low area features.

7.5.8 Peptide View - Precursor Profile

The Precursor Profile, or eXtracted Ion Chromatogram (XIC) of the precursor ion, graphs the elution profile of the selected peptide feature vector over the retention time range where it was identified. The graph is plotted with the retention time along the x-axis and the intensity along the y-axis.

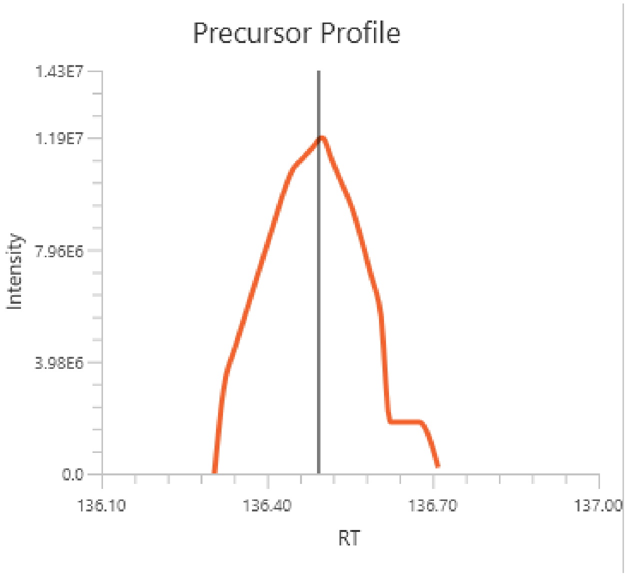

7.5.9 Peptide View - Fragment Ion XIC

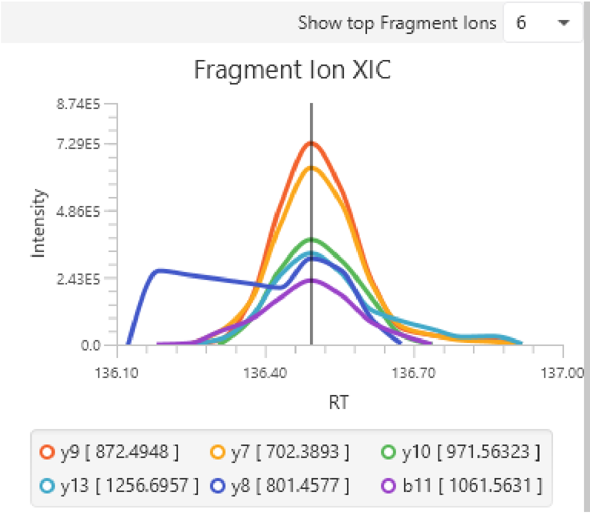

An eXtracted Ion Chromatogram (XIC) chart that displays the shape of each identified fragment ion across all scans for the selected PSM. Select the number of ions to display in the chart using the "Show top Fragment Ions" dropdown.

7.6 Spectral Library Search Result - Exporting

In Project View, double-click on Export to open the Export node.

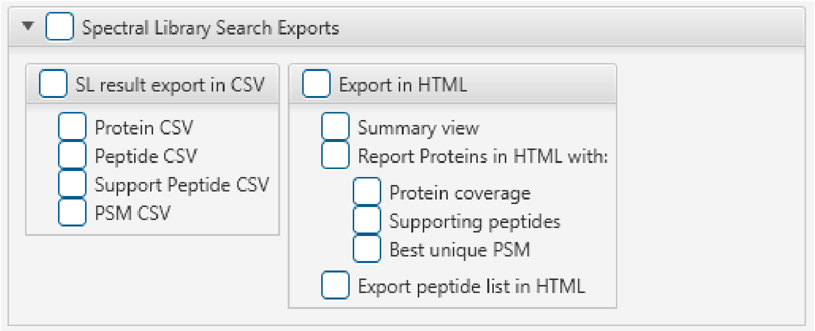

7.6.1 Spectral Library Search - Exporting - comma separated values (CSV) format

There are several options for exporting the PEAKS Identification results in CSV format:

- **Protein CSV:** The list of protein identifications, filtered by the protein filters in the Summary view and will be saved to proteins.csv.
- **Peptide CSV:** All of the identified peptides and details will be saved to peptides.csv.
- **Support peptides CSV:** A list of supporting peptides of each protein identification, filtered by the protein filters in the "Summary" view and will be exported to protein-peptides.csv. This usually contains more entries than the Peptide table as a peptide can be identified in several proteins and, therefore, can be reported multiple times in this file.
- **PSM CSV:** The peptide-spectrum matches (PSMs) will be exported to DB search psms.csv. Peptides differentiated with only I/L isoform are represented by separate entries. As a result, the number of entries in this file might be bigger than the number of PSMs mentioned in the Summary view.

7.6.2 Spectral Library Search - Exporting - HTML format

Export in HTML: This will generate single or multiple HTML reports in the specified location.

The following exporting options are available:

- **Summary view:** The “Summary” view page will be saved as a summary.html file in HTML format in the specified location.
- **Protein coverage:** The coverage pane will be saved for each protein.
- **Supporting peptides:** A list of supporting peptides will be saved for each protein.
- **Best unique PSM:** The best unique PSM will be saved for each protein.
- **Export peptide list in HTML:** creates a separate HTML-containing peptide identifications.

An individual protein will have its own HTML output file, where the corresponding protein coverage, the supporting peptides, and the best unique PSM are gathered.

7.7 Understanding PEAKS DIA Database Search Results

After the DIA database search is complete, DB Search result nodes are generated. Double-click the DB Search node to examine the analysis report. The analysis report is presented in three pages:

- Summary: This shows an outline of the library search results with key statistics. The overall quality of the experiment can be examined and the filters for peptide and protein identifications can be adjusted.

- Protein: This shows a list of protein identifications. This view also visualizes protein sequence coverage and helps with protein characterization.
- Peptide: This shows a list of peptide identifications. This view also provides spectrum annotation and other detailed information for peptide precursor spectrum matches.

7.8 DIA Database Search - Summary View

The DIA DB Search result **Summary View** is similar to the PEAKS Spectral Library Search identification result.

The Summary view reports key statistics as an overview of the result. In the report, several statistical charts can be examined to assess result quality. At the top of each summary result page, protein filters can be set. Identifications that fall below the filters will not be visible in the result pages or exports.

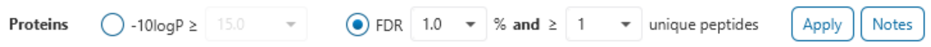

Proteins -10lgP: The PEAKS protein score (-10lgP) is calculated as the weighted sum of the -10lgP scores of the protein's supporting peptides. After removing any redundant peptides, the supporting peptides are sorted by -10lgP scores in descending order, and the k-th ranked peptide contributes to the weighted sum with a weight of 1/k. A default threshold of 15 is set.

Proteins FDR: The Proteins can also be filtered in accordance to the false-discovery rate of the protein set.

Proteins Unique Peptides: The minimum number of unique supporting peptides for a protein identification. A unique peptide is defined as a peptide that can be mapped to only one protein group.

After changing the filters, click Apply to apply the new filter and update the results accordingly.

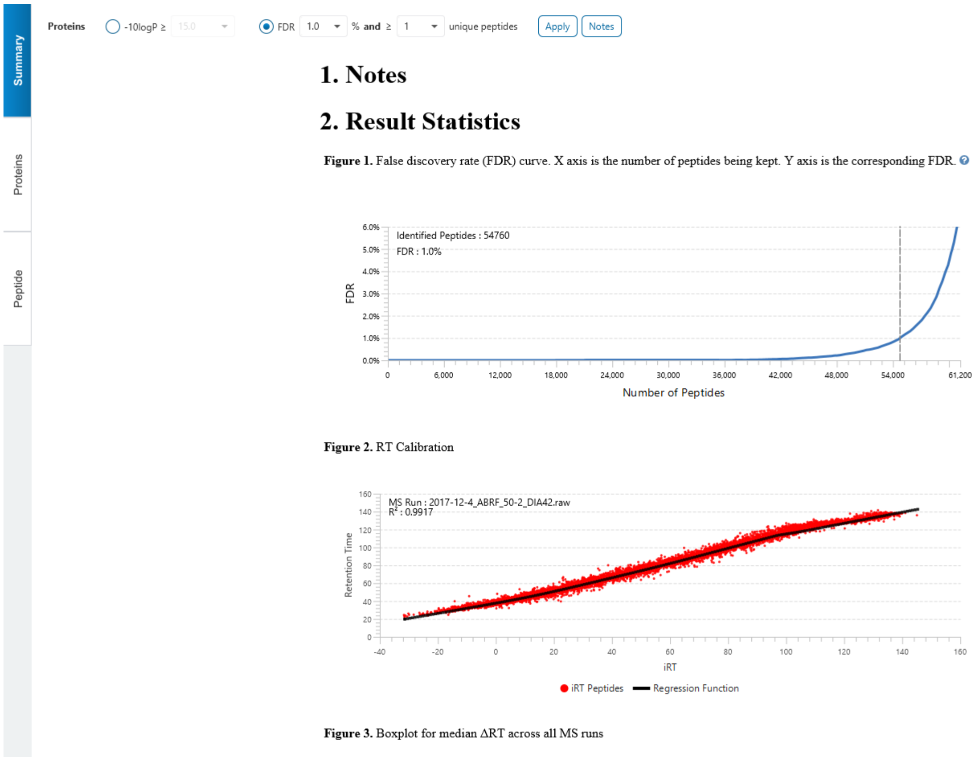

Click on 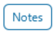

to open the Notes dialog to save useful information regarding this Analysis. The Notes will appear in Section 1 of the summary page at the top.

The Summary view reports key statistics as an overview of the result. In the report, several statistical charts can be examined to assess result quality.

7.8.1 DIA Database Search - Figure 1. False Discovery Rate (FDR) curve.

Figure 1. False discovery rate (FDR) curve. X axis is the number of peptides being kept. Y axis is the corresponding FDR.

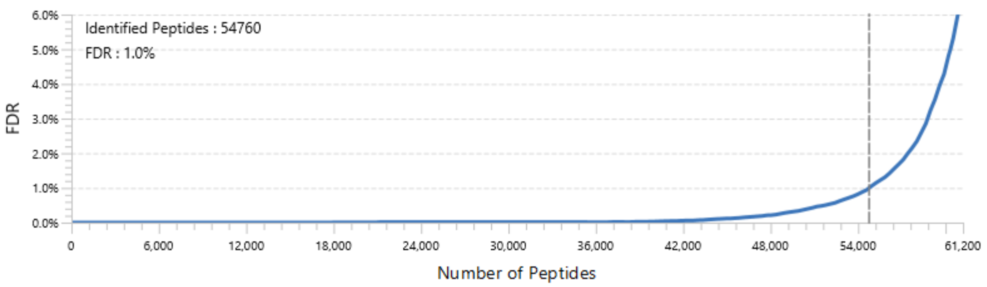

Figure 1 shows the FDR curve with respect to the number of Peptides (or PSMs) being kept after filtration by the -10lgP score. By lowering the score threshold, more Peptides are kept in the filtered result. Conversely, the FDR increases because more false positives are kept. In this figure, the vertical line indicates the current score threshold. The number of Peptides and the corresponding FDR by the current score threshold are shown in the top-left corner. PEAKS Search estimates FDR using the "decoy-fusion" approach. Decoy-fusion is an enhanced target-decoy approach that makes more conservative FDR estimations. For details of the "decoy-fusion" approach, please referto the publication, *"PEAKS DB: De Novo sequencing assisted database search for sensitive and accurate peptideidentification", Mol Cell Proteomics, 2011 Dec 20.*

It is commonly recommended to set FDR at <1% by adjusting the score threshold. If a rapid growth of the FDR around the 1% FDR threshold is noticed, some PSMs may be sacrificed to significantly reduce the FDR.

Note: When counting the number of PSMs, PEAKS Studio keeps one peptide per spectrum at most. Thus, the number of PSMs is actually the number of spectra with assigned peptides.

Note: During Analysis creation, if Peptide FDR (%) filter was used in Report step, the Summary page FDR curve in the results will show the Peptide FDR curve and corresponding number of Identified peptides.

Note: Decoy matches are not counted in the number of PSMs. Unless otherwise specified, decoy matches are also excluded from the other statistical numbers shown in the Summary view.

7.8.2 DIA Database Search result - Figure 2. RT Calibration

Figure 2. RT Calibration

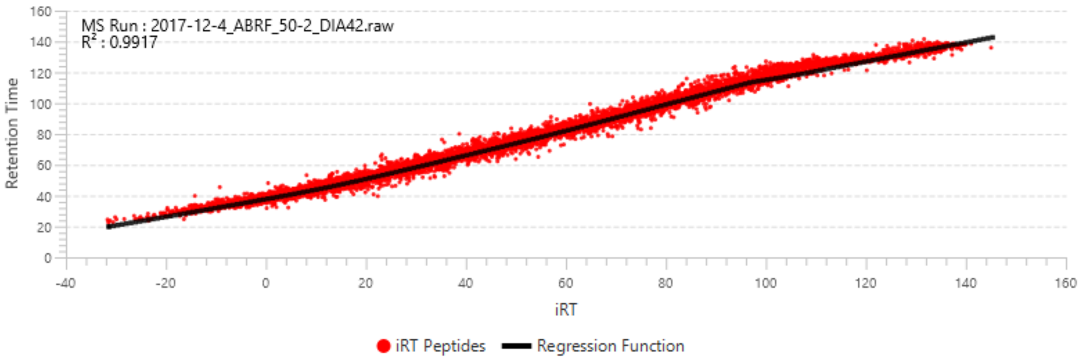

Plots the RT regression function used during the DIA database search. Peptides from the first MS Run in the analysis are plotted against their indexed retention time (iRT) predicted using machine learning. The R^2 value is presented based on the regression function.

7.8.3 DIA Database Search result - Boxplot for RT change

Figure 3. Boxplot for median ΔRT across all MS runs

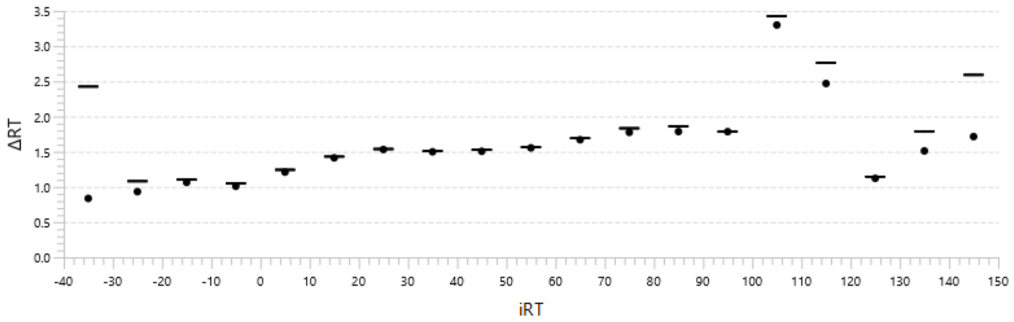

A box plot showing the difference between the observed retention time and iRT peptides within set retention time bins across all fractions, showing the accuracy of retention time prediction.

7.8.4 DIA Database Search result - Table 1. Statistics of data

Statistics of Data tabulates MS1 and MS2 information, as well as identification in the peptide and protein level. The protein level statistics will only be presented if protein inference was performed. This table in the Summary view provides a condensed statistical information for the analysis.

Table 1. Statistics of data.

	#MS Run	#Scans		#PSMs	#Peptides	#Sequences	#Proteins			#PSM/#MS2
		MS1	MS/MS				Groups	All	Top	
Total	2	4678	196476	143537	54760	47432	5700	5999	5770	73.1%
Sample 1	1	2339	98238	71632	50057	43887	5664	5959	5731	72.9%
Sample 2	1	2339	98238	71905	50172	44116	5668	5961	5737	73.2%

#MS Run: Total # of runs in each sample.

#MS1: Total # of MS1 spectra for each sample. For timsTOF data this shows the # MS1 frames.

#MS/MS: Total # of MS2 spectra for each sample. This does not include chimeric scans. For timsTOF data this shows the # MS2 frames.

#PSMs: The number of peptide-spectrum matches.

#Peptides: The unique number of peptide sequences with modifications.

#Sequences: The unique number of peptide sequences not including modifications.

#Proteins Groups: PEAKS Studio groups proteins identified by a common set of peptides. This number shows the number of protein groups in the filtered result based on All proteins.

#All Proteins: Total number of proteins with significant peptides, according to current protein filters.

#Top Proteins: Total number of top proteins with significant peptides, according to current protein filters.

#PSM/#MS2: The identification rate by dividing #PSM by #MS2 for the sample.

7.8.5 DIA Database Search result - Result filtration parameters

Tables 2-4 show the statistical numbers of the data and results.

Table 2. Result filtration parameters.

Peptide -10lgP	≥ 0
Protein -10lgP	≥ 41.5
Proteins unique peptides	≥ 1

Table 3. Statistics of filtered result.

FDR (Peptide-Spectrum Matches)	0.5%
FDR (Peptide Sequences)	1.0%
FDR (Protein Group)	100.0%

Table 4. PTM profile.

Name	ΔMass	Position	#PSM	#Peptides	-10lgP	Area	AScore
Deamidation	.98	NQ	20897	5495	51.59		
Carbamidomethyl	57.02	C	17500	8620	51.30		
Oxidation	15.99	M	4787	1713	51.36	9.09E6	

Table 2. Result filtration parameters: Table 2 summarizes the result filtration parameters set at the top of the Summary View page. This information provides a helpful reference for exported data.

Table 3. Statistics of filtered results: Table 3 presents the statistics based on the data analysis results after setting the desired filters. See below for further explanation:

- **FDR (Peptide-Spectrum Matches):** The total number of decoy database assignments to spectra relative to the total number of target database assignments to spectra represented as a percentage.
- **FDR (Peptide Sequences):** The total number of decoy database assignments to unique peptide sequences relative to the total number of target database assignments to unique peptide sequences represented as a percentage.
- **FDR (Protein Group):** The total number of decoy protein groups where the top hit in the protein group is a decoy database protein relative to the total number of target database protein groups where the top hit is from the target database.

Table 4. PTM Profile: shows modifications identified in the filtered result and the number of PSMs containing each modification

7.8.6 DIA Database Search result - Experiment Control

Figure 4 in the Summary View show the precursor mass errors of PSMs in filtered results. The precursor mass error is calculated in ppm as 10^6 × (precursor mass - peptide mass) / peptide mass. Calibration is for display purposes only and PEAKS corrects m/z value for each PSM to correct systematic biases and instrument measurement drift.

Figure 4. Distribution of precursor mass error of filtered PSM in ppm.

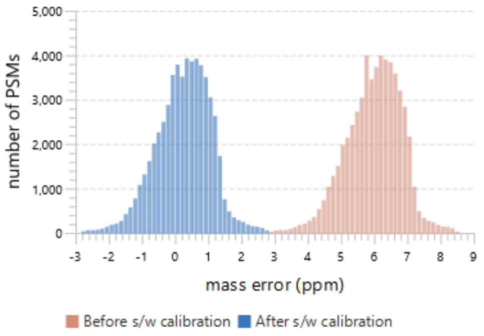

Table 5 shows the number of identified peptides in each sample with the number of missed cleavages, indicating the enzyme digestion efficiency.

Table 5. Number of identified peptides in each sample by the number of missed cleavages.

Missed Cleavages	0	1	2	3	4+
Sample 1	26015	878	32	6	0
Sample 2	26352	888	31	6	0

7.9 DIA Database Search - Proteins View

The Protein View lists protein identifications that have been filtered by the current settings in Summary View. It also visualizes the protein sequence coverage for identified proteins. The view has four main components:

- Protein table: This lists protein identifications that have been filtered by the current settings in the Summary View and the “Protein View Filters”.
- Coverage: This visualizes the protein sequence coverage of the selected protein, showing the mapping of support peptides and de novo tags to the protein sequence.
- Peptides: This lists supporting peptides mapped to the selected protein.

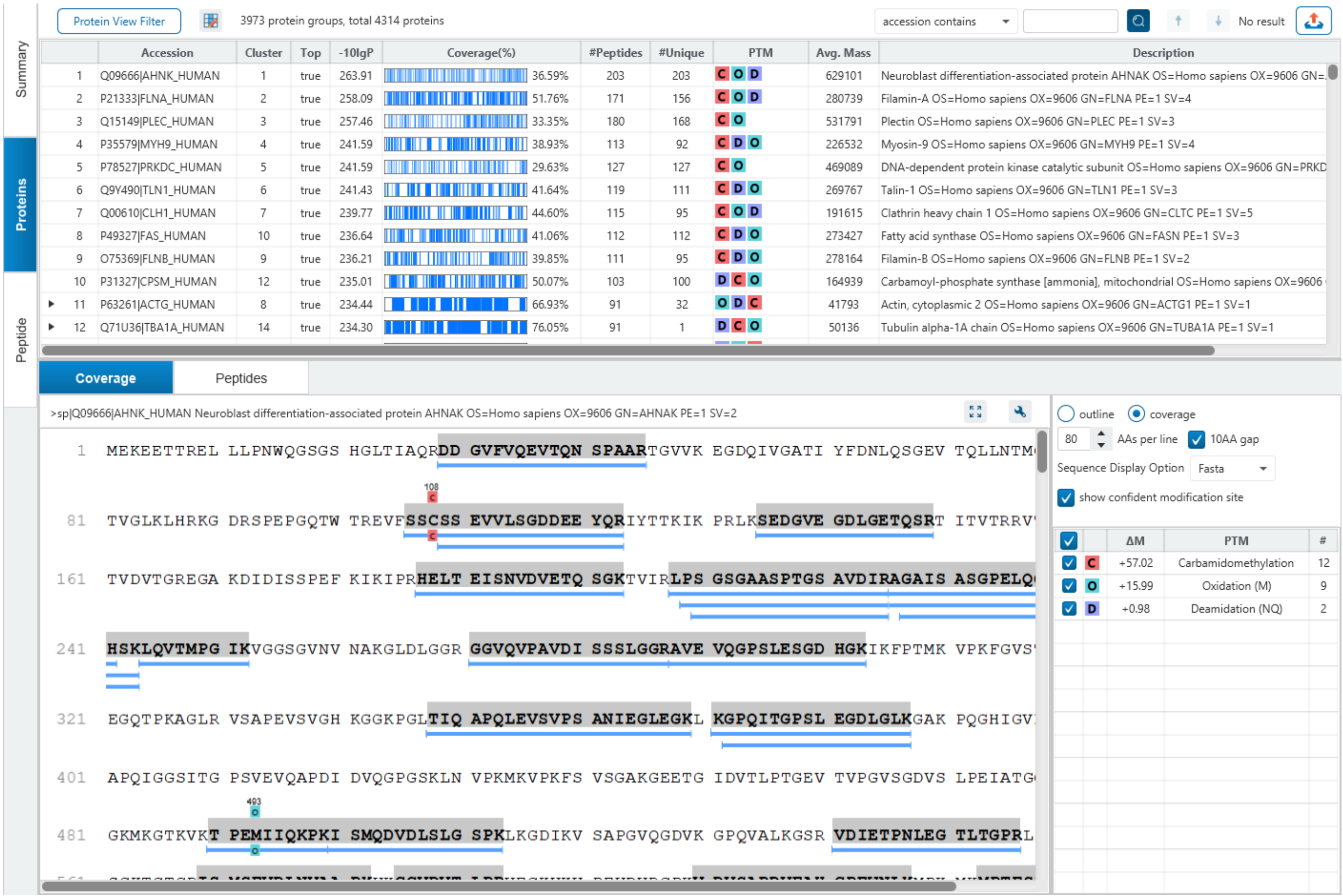

7.9.1 Proteins View - Protein Table controls

At the top of the Proteins View, there are the options to filter, show/hide columns, search, and export the protein table.

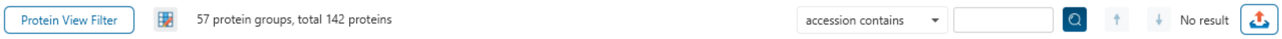

7.9.1.1 Protein Table controls - Protein View Filters

Click on the Protein View Filter button to open the Protein View Filter.

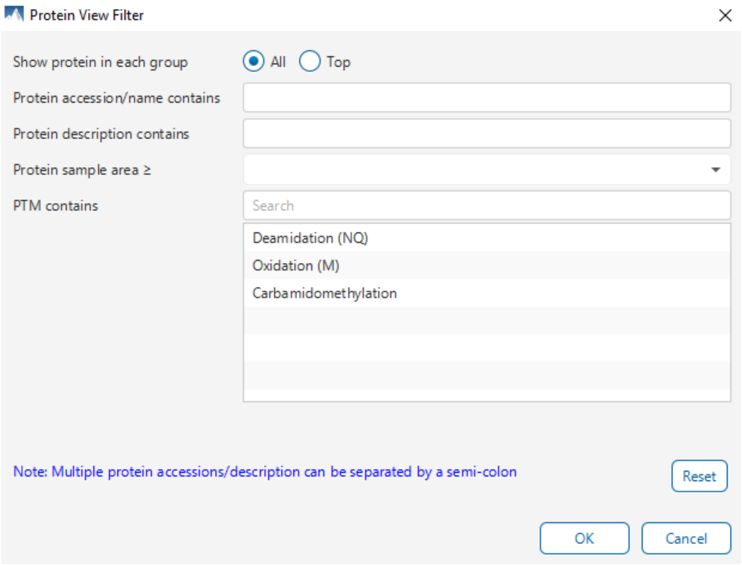

The following options are available for filtering the protein table:

Show Top, All, or First proteins in each group: Adjust the protein list based on Protein grouping, top is selected by default. Proteins are grouped based on parsimony.

- **Top:** Show only the top proteins in each group. These proteins are supported by the most unique peptides in the group. Proteins in the group that share a subset of the unique peptides that support the top protein (subproteins) will not be displayed.
- **All:** Show all proteins in each group. Proteins are grouped together if they are supported by the same set or a sub-set of the top protein in the group. If a protein is supported by a peptide not supporting the top protein it will be added to a new group.

Protein accession/name contains: Restrict the protein table to only display protein IDs which contain the entered text in its accession/name.

Protein description contains: Only proteins with descriptions that contain the entered text will be included in the protein table if text is entered in this field.

Note: In these above two filters, multiple entries can be separated by a semi-colon.

Protein sample area: Filter proteins based on total protein area found in each sample. Sample areas are calculated using the total of all peptide features from unique supporting peptides. If a protein is identified and has areas from multiple samples, only one of the samples must pass this filter to be included.

PTM contains: Filter protein results by the presence of selected PTMs. Proteins that are supported by peptides containing modifications that match the selected PTMs will be included. If multiple PTMs match the modification name, they will all be included in the table. For SPIDER results, the mutations will also be listed in the PTM contains filter.

Click on the Reset button to remove all filters.

Click on OK to apply the Protein View Filter to the Protein table.

Click on Cancel to discard changes and close the dialog.

7.9.1.2 Protein Table controls - Protein count

At the top of the protein table shows the count of protein groups and number of proteins in the table based on the current Protein View Filters.

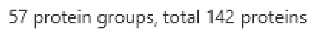

7.9.1.3 Protein Table controls - Optional columns

Click on the button to see a list of sample-based optional columns.

Select the corresponding sample-based Coverage, Spectra count, or Area to add into the Protein Table view.

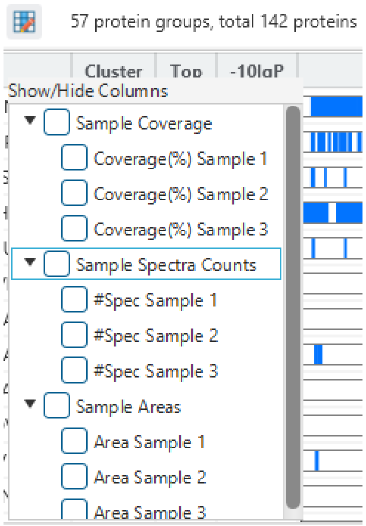

- **Coverage by Sample:** The protein coverage as a percentage will be displayed for the individual sample when selected.
- **#Spec by Sample:** The total number of spectra identified that support the given protein. Totals are given for each sample included in the search.
- **Area by Sample:** The total area of peptide features from unique supporting peptides in each sample will be displayed.

The exports will always include the contents of these optional columns even if they are hidden.

7.9.1.4 Protein Table controls - Protein Table Search Function

An easy-to-use search function is available for the protein table. The protein accession or description substring can be searched for, and all search results can be navigated through with the Up and Down arrows.

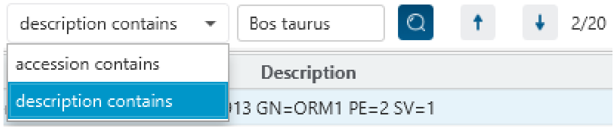

Protein accession/name contains: Restrict the protein table to only display protein IDs which contain the entered text in its accession/name.

Protein description contains: Only proteins with descriptions that contain the entered text will be included in the protein table if text is entered in this field.

7.9.1.5 Protein Table controls - Protein Table Export

Click on the button to export the contents of the current table. This considers the Protein View Filters, the sorting applied to the table and is consistent with the view. There is the option to export the Protein table and/or the Supporting peptide table in CSV format.

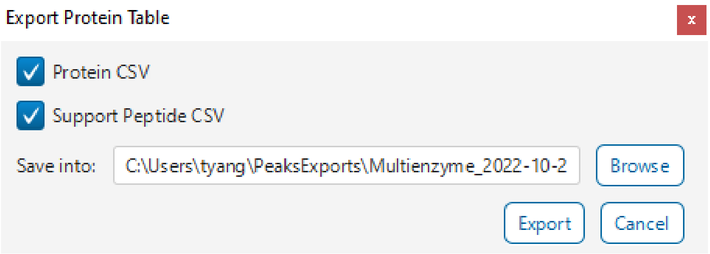

7.9.2 Proteins View - Protein Table

Each row in the table represents a group of proteins that are supported by a common set of peptides. To expand the group, click the right arrow button at the left.

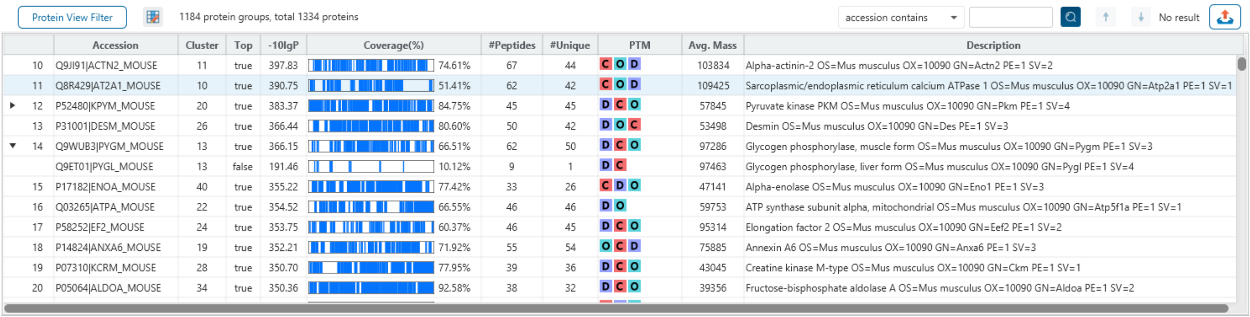

	Accession	Cluster	Top	-10lgP	Coverage(%)	#Peptides	#Unique	PTM	Avg. Mass	Description
10	Q9JI91|ACTN2_MOUSE	11	true	397.83	74.61%	67	44	C O D	103834	Alpha-actinin-2 OS=Mus musculus OX=10090 GN=Actn2 PE=1 SV=2
11	Q8R429|AT2A1_MOUSE	10	true	390.75	51.41%	62	42	C O D	109425	Sarcoplasmic/endoplasmic reticulum calcium ATPase 1 OS=Mus musculus OX=10090 GN=Atp2a1 PE=1 SV=1
▸ 12	P52480|KPYM_MOUSE	20	true	383.37	84.75%	45	45	D C O	57845	Pyruvate kinase PKM OS=Mus musculus OX=10090 GN=Pkm PE=1 SV=4
13	P31001|DESM_MOUSE	26	true	366.44	80.60%	50	42	D O C	53498	Desmin OS=Mus musculus OX=10090 GN=Des PE=1 SV=3
▾ 14	Q9WUB3|PYGM_MOUSE	13	true	366.15	66.51%	62	50	D C O	97286	Glycogen phosphorylase, muscle form OS=Mus musculus OX=10090 GN=Pygm PE=1 SV=3
	Q9ET01|PYGL_MOUSE	13	false	191.46	10.12%	9	1	D C	97463	Glycogen phosphorylase, liver form OS=Mus musculus OX=10090 GN=Pygl PE=1 SV=4
15	P17182|ENOA_MOUSE	40	true	355.22	77.42%	33	26	C D O	47141	Alpha-enolase OS=Mus musculus OX=10090 GN=Eno1 PE=1 SV=3
16	Q03265|ATPA_MOUSE	22	true	354.52	66.55%	46	46	D O	59753	ATP synthase subunit alpha, mitochondrial OS=Mus musculus OX=10090 GN=Atp5f1a PE=1 SV=1
17	P58252|EF2_MOUSE	24	true	353.75	60.37%	46	45	D C O	95314	Elongation factor 2 OS=Mus musculus OX=10090 GN=Eef2 PE=1 SV=2
18	P14824|ANXA6_MOUSE	19	true	352.21	71.92%	55	54	O C D	75885	Annexin A6 OS=Mus musculus OX=10090 GN=Anxa6 PE=1 SV=3
19	P07310|KCRM_MOUSE	28	true	350.70	77.95%	39	36	D C O	43045	Creatine kinase M-type OS=Mus musculus OX=10090 GN=Ckm PE=1 SV=1
20	P05064|ALDOA_MOUSE	34	true	350.36	92.58%	38	32	D C O	39356	Fructose-bisphosphate aldolase A OS=Mus musculus OX=10090 GN=Aldoa PE=1 SV=2

The columns in the Protein Table are:

- **Accession:** The accession number of the protein as seen in the FASTA database.
- **Cluster:** A unique identifier that classifies which protein group the protein belongs to.
- **Top:** Indicates whether this protein is a top protein.
- **-10lgP:** The protein confidence score.
- **Coverage (%):** The percentage of the protein sequence that is covered by supporting peptides. The coverage is visualized by a colored bar. Light blue blocks indicate the parts of the sequence covered by low-confidence peptides. Dark blue blocks indicate the parts covered by high-confidence peptides. This is the total coverage including results from all samples.
- **#Peptides:** The number of high-confidence supporting peptides.
- **#Unique:** The number of high-confidence supporting peptides that are mapped to only one protein group. Unique peptides with same sequence but different modifications are only counted once in this number.
- **PTM:** The identified modifications displayed with color-coded icons.
- **Avg. Mass:** The protein mass calculated using the average mass.
- **Description:** The protein's header information as seen in the FASTA database.

Note: For #Peptides and #Unique, two peptides with the same starting and ending positions in the protein are counted as one, regardless of their PTM forms. This is to follow the MCP (Molecular & Cellular Proteomics) guidelines.

7.9.3 Proteins View - Coverage Pane

The Protein Coverage view visually maps the supporting peptides and de novo tags to the protein selected in the Protein table. It also shows all identified sites with modifications or mutations to assist with protein characterization at the amino acid level. The "Protein Coverage" view is composed of three major components - Protein Sequence Coverage, Coverage Control Panel, and Protein Tools.

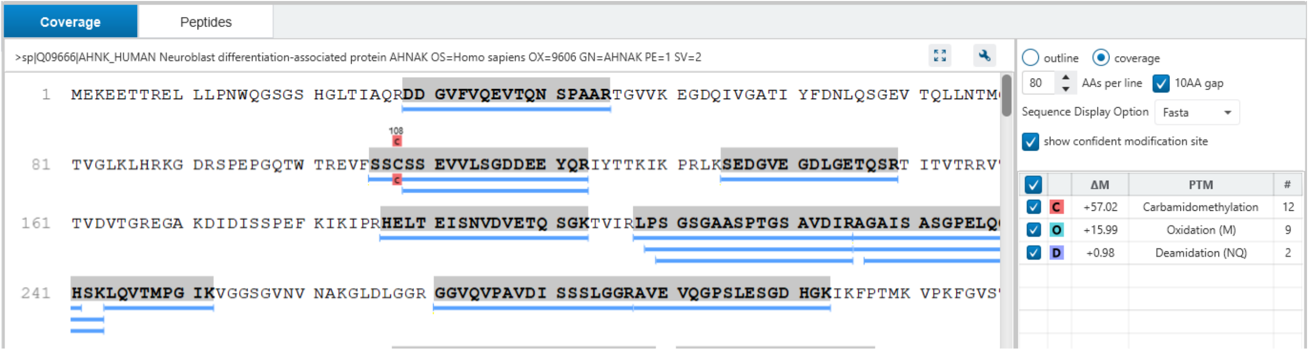

7.9.3.1 Coverage Pane - Protein Sequence Coverage

This area visualizes the coverage of the protein sequence.

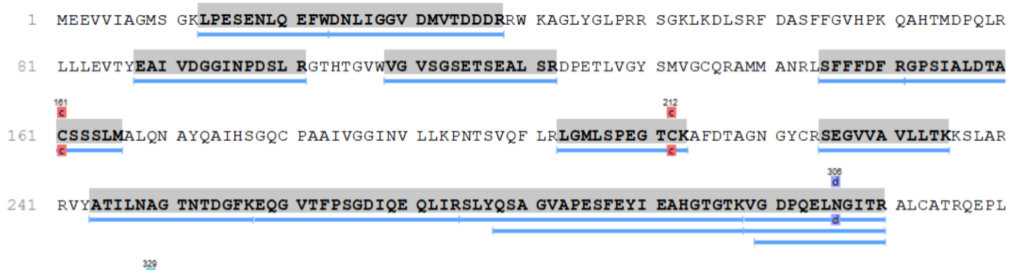

Regions in the protein sequence that are covered by supporting peptides are displayed in bold font with a grey background. Confident modifications and mutations identified in supporting peptides are displayed as icons above the protein sequence. Modifications are represented by colored icons with the first letter of its modification name. If a residue is modified by more than one modification in the same supporting peptide, "*" is used instead of a letter. The header information of the protein is shown on the top of the protein sequence.

The supporting peptides can be shown as colored bars under the protein sequence.

Placing the cursor over a bar shows a tooltip with detailed information of the peptide. Left clicking on a bar shows the annotated spectrum from which the supporting peptide is identified.

Right clicking on a peptide bar shows additional options. “Show in spectrum” has the same behaviour as left clicking on the peptide bar which is to open the annotated spectrum chart. “Show in table” has the function to jump to the supporting peptides table.

7.9.3.2 Coverage Pane - Coverage Control Panel

This panel controls what to display in the protein sequence coverage view.

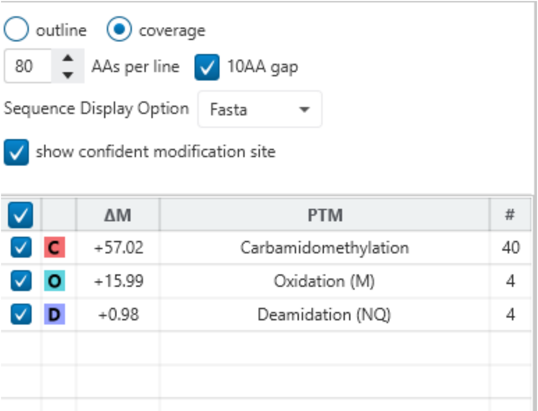

Sequence Display Mode: The protein sequence can be displayed in either "outline" or "coverage" mode.

Outline mode: In outline mode, the protein sequence is displayed without supporting peptides and matched de novo tags. However, the sequence coverage at a specific position can be examined by left-clicking a residue.

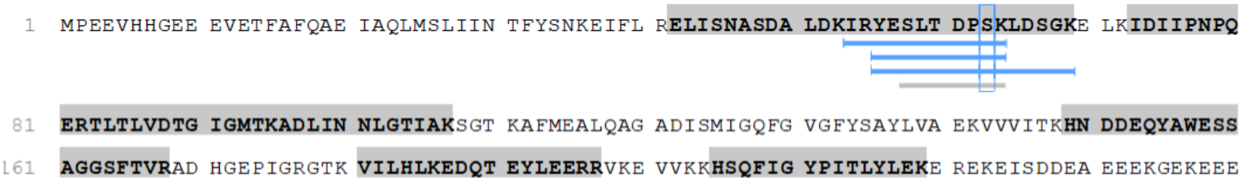

Coverage mode: In coverage mode, all the supporting peptides and matched de novo tags are shown.

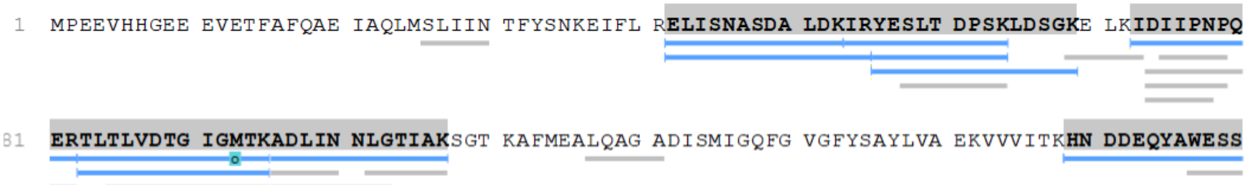

AAs per line and 10AA gap: Specify the number of amino acids per line and whether to show a gap for every 10 amino acids in the protein sequence.

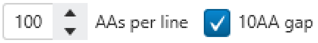

Sequence Display Option: Select whether to view the protein sequence as Fasta or a specific enzyme used in the project. When a particular enzyme is selected, the protein sequence will be highlighted to emphasize expected peptides generated by the selected enzyme's cleavage specificity.

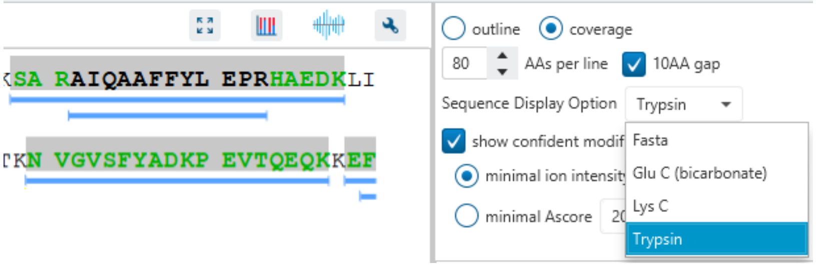

Modification table: The modification table shows the modifications identified in supporting peptides on the protein. For each modification, the number of supporting PSMs with this particular modification is shown. The checkbox on the left controls whether to indicate the modification in the protein coverage.

☑		ΔM	PTM	#
☑	C	+57.02	Carbamidomethylation	40
☑	O	+15.99	Oxidation (M)	4
☑	D	+0.98	Deamidation (NQ)	4

7.9.3.3 Proteins View - Protein Tools

The Proteins View toolbar is at the upper-right corner of the protein sequence coverage tab and contains helpful protein tools.

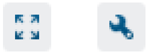

Full screen: 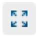 When selected, the protein coverage window expands in full screen mode. Click to return from full screen mode.

Tool box: 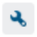 The tool box contains the following tools:

- Copy protein sequence. This copies the protein sequence of the current selected protein into the system clipboard.
- Save protein coverage as image. Different scale options are provided to increase the image size.
- Coverage statistics. This provides useful statistics based on the current selected protein.

7.9.3.4 Proteins View - Supporting Peptides

The "Peptides" tab displays a table of supporting peptides for the protein of interest. This table is similar to the peptide table in the **Peptide** view, except that "Accession" is excluded and the columns "(Checkbox)", "Unique", "Start", and "End" are included. Descriptions of extra columns are given in the following sections.

- **(Checkbox):** This controls whether a peptide is shown in the protein coverage as a blue bar.
- **Unique:** This shows whether the peptide is a unique supporting peptide to the protein group.
- **Start:** This shows the peptide's starting position in the protein.
- **End:** This shows the peptide's ending position (inclusive) in the protein.

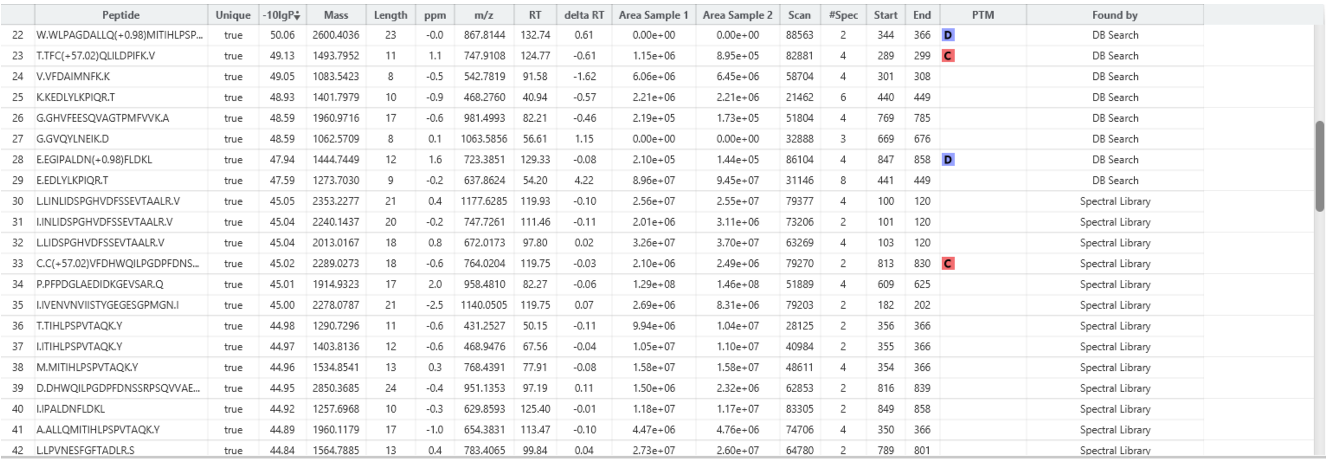

	Peptide	Unique	-10lgP	Mass	Length	ppm	m/z	RT	delta RT	Area Sample 1	Area Sample 2	Scan	#Spec	Start	End	PTM	Found by
22	W.WLPAGDALLQ(+0.98)MITIHLPSP...	true	50.06	2600.4036	23	-0.0	867.8144	132.74	0.61	0.00e+00	0.00e+00	88563	2	344	366	D	DB Search
23	T.TFC(+57.02)QLILDPIFK.V	true	49.13	1493.7952	11	1.1	747.9108	124.77	-0.61	1.15e+06	8.95e+05	82881	4	289	299	C	DB Search
24	V.VFDAIMNFK.K	true	49.05	1083.5423	8	-0.5	542.7819	91.58	-1.62	6.06e+06	6.45e+06	58704	4	301	308		DB Search
25	K.KEDLYLKPIQR.T	true	48.93	1401.7979	10	-0.9	468.2760	40.94	-0.57	2.21e+06	2.21e+06	21462	6	440	449		DB Search
26	G.GHVFEESQVAGTPMFVVK.A	true	48.59	1960.9716	17	-0.6	981.4993	82.21	-0.46	2.19e+05	1.73e+05	51804	4	769	785		DB Search
27	G.GVQYLNEIK.D	true	48.59	1062.5709	8	0.1	1063.5856	56.61	1.15	0.00e+00	0.00e+00	32888	3	669	676		DB Search
28	E.EGIPALDN(+0.98)FLDKL	true	47.94	1444.7449	12	1.6	723.3851	129.33	-0.08	2.10e+05	1.44e+05	86104	4	847	858	D	DB Search
29	E.EDLYLKPIQR.T	true	47.59	1273.7030	9	-0.2	637.8624	54.20	4.22	8.96e+07	9.45e+07	31146	8	441	449		DB Search
30	L.LINLIDSPGHVDFSSEVTAALR.V	true	45.05	2353.2277	21	0.4	1177.6285	119.93	-0.10	2.56e+07	2.55e+07	79377	4	100	120		Spectral Library
31	I.INLIDSPGHVDFSSEVTAALR.V	true	45.04	2240.1437	20	-0.2	747.7261	111.46	-0.11	2.01e+06	3.11e+06	73206	2	101	120		Spectral Library
32	L.LIDSPGHVDFSSEVTAALR.V	true	45.04	2013.0167	18	0.8	672.0173	97.80	0.02	3.26e+07	3.70e+07	63269	4	103	120		Spectral Library
33	C.C(+57.02)VFDHWQILPGDPFDNS...	true	45.02	2289.0273	18	-0.6	764.0204	119.75	-0.03	2.10e+06	2.49e+06	79270	2	813	830	C	Spectral Library
34	P.PFPDGLAEDIDKGEVSAR.Q	true	45.01	1914.9323	17	2.0	958.4810	82.27	-0.06	1.29e+08	1.46e+08	51889	4	609	625		Spectral Library
35	I.IVENVNVIISTYGEGESGPMGN.I	true	45.00	2278.0787	21	-2.5	1140.0505	119.75	0.07	2.69e+06	8.31e+06	79203	2	182	202		Spectral Library
36	T.TIHLPSPVTAQK.Y	true	44.98	1290.7296	11	-0.6	431.2527	50.15	-0.11	9.94e+06	1.04e+07	28125	2	356	366		Spectral Library
37	I.ITIHLPSPVTAQK.Y	true	44.97	1403.8136	12	-0.6	468.9476	67.56	-0.04	1.05e+07	1.10e+07	40984	2	355	366		Spectral Library
38	M.MITIHLPSPVTAQK.Y	true	44.96	1534.8541	13	0.3	768.4391	77.91	-0.08	1.58e+07	1.58e+07	48611	4	354	366		Spectral Library
39	D.DHWQILPGDPFDNSSRPSQVVAE...	true	44.95	2850.3685	24	-0.4	951.1353	97.19	0.11	1.50e+06	2.32e+06	62853	2	816	839		Spectral Library
40	I.IPALDNFLDKL	true	44.92	1257.6968	10	-0.3	629.8593	125.40	-0.01	1.18e+07	1.17e+07	83305	2	849	858		Spectral Library
41	A.ALLQMITIHLPSPVTAQK.Y	true	44.89	1960.1179	17	-1.0	654.3831	113.47	-0.10	4.47e+06	4.76e+06	74706	4	350	366		Spectral Library
42	L.LPVNESFGFTADLR.S	true	44.84	1564.7885	13	0.4	783.4065	99.84	0.04	2.73e+07	2.60e+07	64780	2	789	801		Spectral Library

7.10 DIA Database Search Result - Peptide View

The "Peptide View" displays the resulting peptide identifications that have been filtered by the current settings in Summary View and "Peptide View Filters". The table on the top shows the details of each peptide identification. The bottom section provides additional information about the peptide-spectrum matches for the native peptide selected in the table.

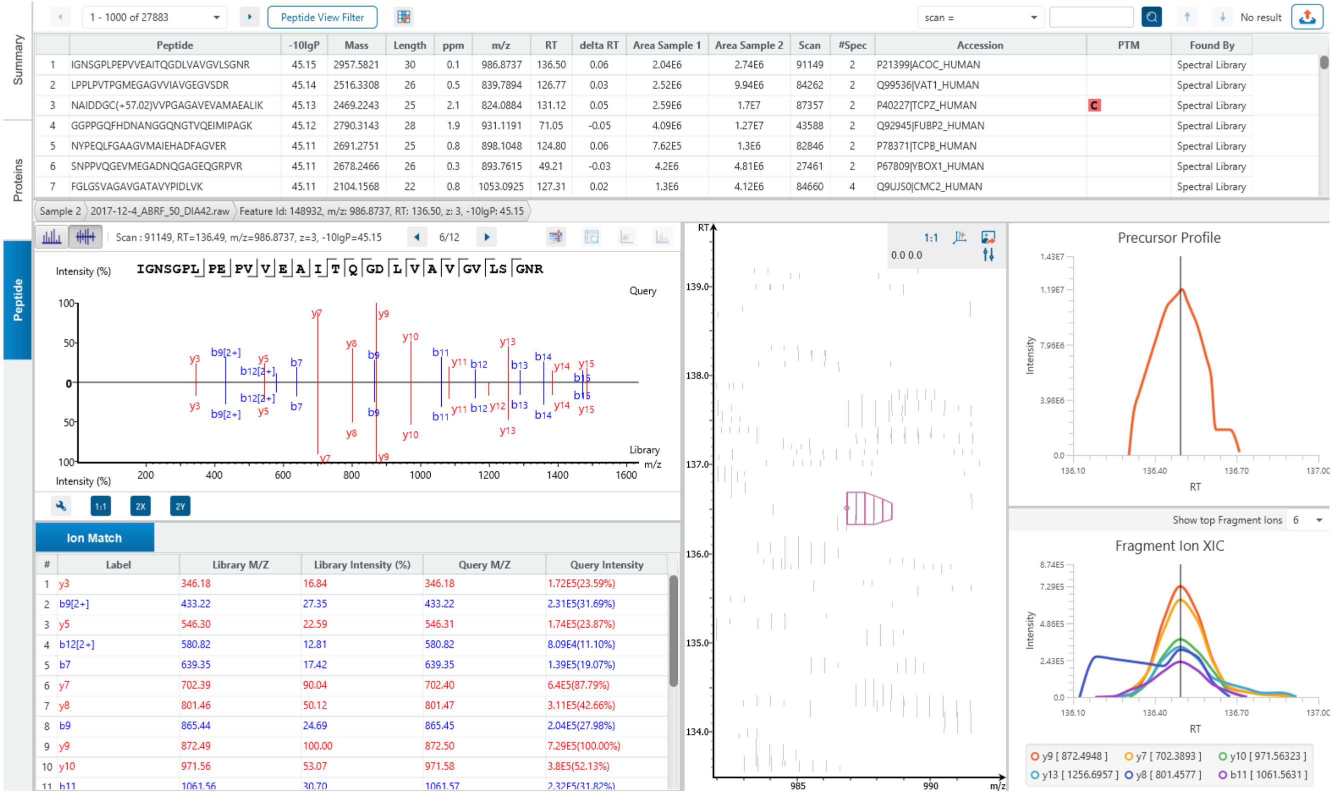

7.10.1 Peptide View - Peptide Table

The Peptide Table shows the filtered native peptide identification results. Each row in the table is a native peptide identification represented by its highest-scoring PSM. When there are more than 1000 peptides, the table is split into multiple pages.

Note: If there are multiple PSMs matched to a peptide, the table displays the information for the top-scoring PSM. Peptides with the same sequence but different modifications are considered different and shown in different rows.

To copy the sequence, right click on a row (or multiple rows) in the table and select “Copy selected peptide sequence(s)”. Another option is to mouse over any cell in the table and hitting Ctrl + C on your keyboard to copy the contents of those selected cells.

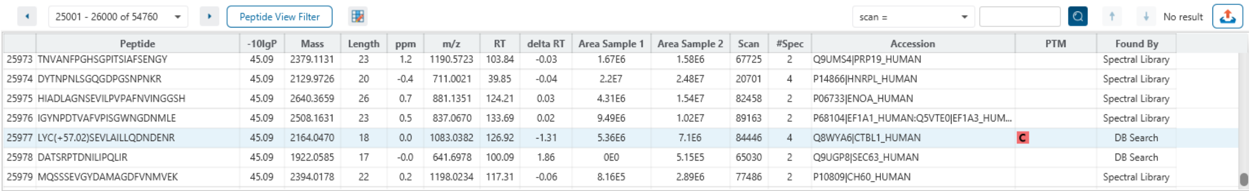

	Peptide	-10lgP	Mass	Length	ppm	m/z	RT	delta RT	Area Sample 1	Area Sample 2	Scan	#Spec	Accession	PTM	Found By
25973	TNVANFPGHSGPITSIAFSENGY	45.09	2379.1131	23	1.2	1190.5723	103.84	-0.03	1.67E6	1.58E6	67725	2	Q9UMS4|PRP19_HUMAN		Spectral Library
25974	DYTNPNLSGQGDPGSNPNKR	45.09	2129.9726	20	-0.4	711.0021	39.85	-0.04	2.2E7	2.48E7	20701	4	P14866|HNRPL_HUMAN		Spectral Library
25975	HIADLAGNSEVILPVPAFNVINGGSH	45.09	2640.3659	26	0.7	881.1351	124.21	0.03	4.31E6	1.54E7	82458	2	P06733|ENOA_HUMAN		Spectral Library
25976	IGYNPDTVAFVPISGWNGDNMLE	45.09	2508.1631	23	0.5	837.0670	133.69	0.02	9.49E6	1.02E7	89163	2	P68104|EF1A1_HUMAN:Q5VTE0|EF1A3_HUM...		Spectral Library
25977	LYC(+57.02)SEVLAILLQDNDENR	45.09	2164.0470	18	0.0	1083.0382	126.92	-1.31	5.36E6	7.1E6	84446	4	Q8WYA6|CTBL1_HUMAN	C	DB Search
25978	DATSRPTDNILIPQLIR	45.09	1922.0585	17	-0.0	641.6978	100.09	1.86	0E0	5.15E5	65030	2	Q9UGP8|SEC63_HUMAN		DB Search
25979	MQSSSEVGYDAMAGDFVNMVEK	45.09	2394.0178	22	0.2	1198.0234	117.31	-0.06	8.16E5	2.89E6	77486	2	P10809|CH60_HUMAN		Spectral Library

The following list describes the contents in each of the default columns:

- **Peptide:** The amino acid sequence of the peptide, as determined by the PEAKS searchworkflow. A modified residue is followed by a pair of parentheses enclosing the modification.
- **-10lgP:** The peptide -10lgP score. The score indicates the scoring significance of a peptide-precursor spectrum match.
- **Mass:** The monoisotopic mass of the peptide.
- **Length:** The number of amino acids in the sequence backbone.
- **ppm:** The precursor mass error, calculated as $10^6 \times$ (precursor mass - peptide mass) / peptide mass.
- **m/z:** The precursor mass-to-charge ratio.
- **RT:** The retention time (elution time) of the spectrum as recorded in the data.
- **delta RT:** The difference between the query and library RT.
- **Area per Sample:** The combined area for all features in that sample associated with this peptide.
- **Scan:** The scan number of the spectrum that matches the peptide sequence with the highest -10lgP.
- **#Spec:** number of MS2 spectra assigned to the peptide.
- **Accession:** The accessions of proteins that contain this peptide.
- **PTM:** The types and the numbers of modifications present in the peptide shown in color-coded icons.
- **Found By:** The name of the PEAKS search workflow that identifies the peptide; this can only be Spectral Library or DB Search for this result.

7.10.2 Peptide View - Peptide Table controls

The menu above the peptide table provides options to scroll through the table of peptides, filter the table, show optional columns and search for specific entries.

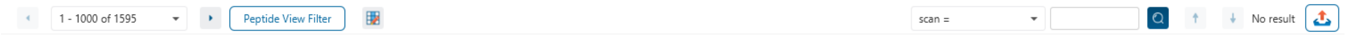

7.10.2.1 Peptide Table controls - Pagination

For easy viewing and to improve performance, the peptide table is limited to showing 1000 entries at once. Pagination is used to manage which set of 1000 entries will be viewed.

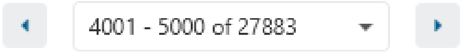

In the top left, there is a dropdown to select the rows for viewing. Click the left arrow or right arrow to select the previous/next 1000 rows. If the table is sorted or filtered, it may update the pagination set of rows that is currently in view.

7.10.2.2 Peptide Table controls - View Filter

Click on the 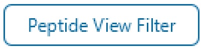

button to open the Peptide View Filter which is used to filter the Peptide table. The following are the filter options provided:

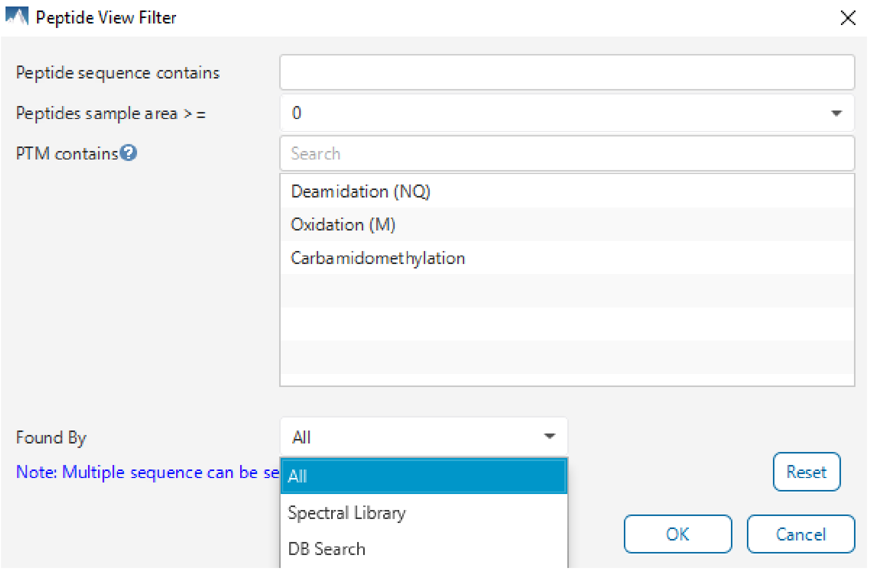

- **Peptide sequence contains**: Enter an amino acid sequence, only peptides that contain that sequence will be displayed in the peptide table. Adding PTMs in brackets in the sequence search is not handled, meaning only the sequence backbone is considered in the filter. Use the PTM contains for further filtering if necessary.
- **Peptide sample area:** Filters based on total peptide area found in each sample. Sample areas are calculated using the total of all peptide features from all spectra that identified the native peptide. If the peptide is found in multiple samples, only one sample must pass this filter for the peptide to be included.
- **PTM contains:** Type the names of PTMs you would like to include. Peptides containing PTMs that match the text will be included. If multiple PTMs match the text they will all be included in the table.
- **Found By:** The dropdown gives the option to show all peptides or only peptides found by the Spectral Library Search or DIA Database Search algorithm.

Note: In all filters, multiple entries can be separated by a semi-colon.

Click on the Reset button to reset all filters to their default setting.

Click on OK to apply the filters to the Peptide table.

Click on Cancel to discard the changes in the View Filter.

7.10.2.3 Peptide Table controls - Optional Columns

Click on the 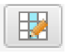 button to open the optional columns dropdown. By default the sample area columns are displayed. Sample Spec count columns can also be shown. These sample spec counts are always from identification, and does not consider whether there are feature vectors present. For projects with many samples, it might be useful to hide these optional columns and reduce the width of the peptide table.

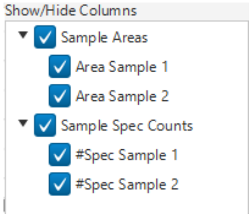

7.10.2.4 Peptide Table controls - Search function

On the top right of menu, there is a search function with 4 options. Search results can be navigated between using the Up or Down arrows.

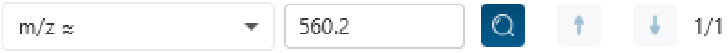

Options for searching include:

- **Scan =:** Search for the Scan number associated with each peptide. One scan can be associated to multiple peptides. For projects with multiple data files, the scan number is searched on every fraction.
- **m/z ≈:** Search for the approximate m/z. The search will consider a range of m/z equal to the search value +/- 1 of the last significant digit. For example, searching for m/z ≈ 560.21 will find all m/z between [560.20, 560.22]
- **RT ≈:** Search for the approximate RT. Likewise, the search will consider a range of RT equal to the search value +/- 1 of the last significant digit set. For example, searching for RT ≈ 55.5 will find all m/z between [55.4, 55.6]
- **Seq contains:** Search for the sequence in the table. If specifying a sequence without PTMs, the results will include that sequence and any sequences with PTMs with the same backbone. If specifying a sequence with bracket and some mass value (like in the above image), then only sequence that have a substring with an exact match will be considered.
  Additionally, since the peptide table shows both I/L and they can not be distinguished, then searching for I in the sequence is equivalent to searching for L in the sequence. i.e. Searching for “LVLK” is the same as searching for “LVIK”, “IVLK”, and “IVIK”

7.10.3 Peptide View - Feature selection breadcrumb

There is a sample and feature selection breadcrumb which allows you to switch between features associated with the peptide. Click on the Sample in the breadcrumb to reveal other samples and the features in that sample related to the current peptide selection. From here you can select other samples, fractions, or features.

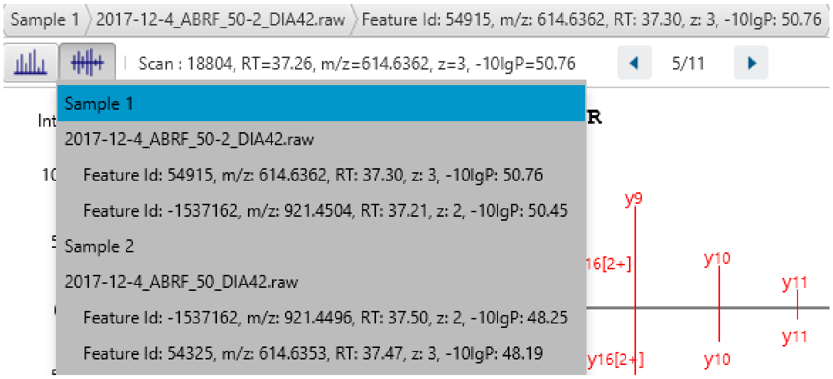

By clicking on the fraction level, all fractions in this sample are revealed as well as the features in each fraction associated with this peptide. From here you can select other fractions and features to view.

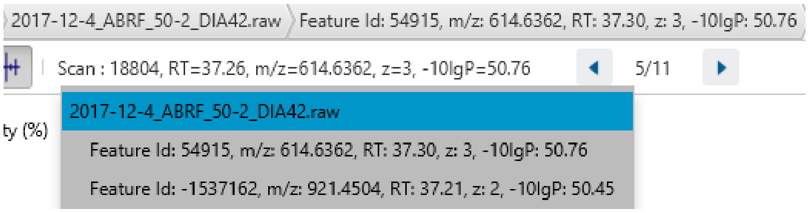

Lastly, clicking on the feature level will show all features in the currently selected fraction. Features with Feature Id <= 0 are known as fake features and are constructed to enhance identification.

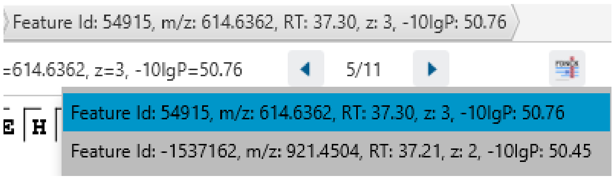

7.10.4 Peptide View - PSM selection

Underneath the feature breadcrumb will shown the currently selected PSM. Click on the Left and Right arrow buttons to switch between PSMs related to the currently selected feature.

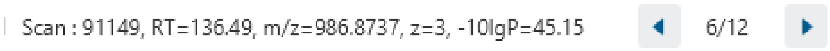

All other components in the Peptide tab interface will update according to the currently selected PSM.

For diaPASEF instruments, Frame is used rather than Scan number.

7.10.4.1 Peptide View - Protein jump button

Click on 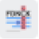 to jump to a protein that contains the currently selected PSM. If there are more than 1 protein, a dropdown is revealed which allows the user to select which protein to jump to. If a protein is filtered out due to a protein summary filter or summary page protein filter, then the option to jump will be grayed out.

7.10.5 Peptide View - Annotated Spectrum Chart

Click on button to switch from the Mirror plot view and annotated spectrum chart view.

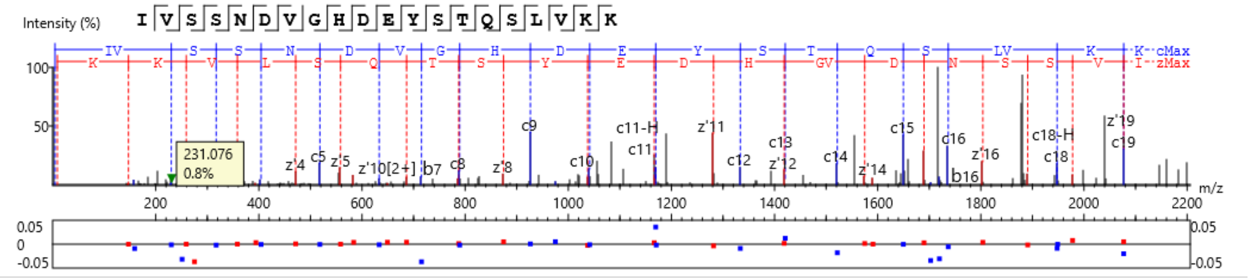

For the currently selected PSM, a graphical representation is shown in the form of an annotated spectrum chart. There are several controls provided for this view which will be explored in the following sections.

7.10.5.1 Annotated Spectrum Chart - Chart Navigation

Moving the cursor over the peptide sequence in the spectrum will show the mass transitions for a particular amino acid residue.

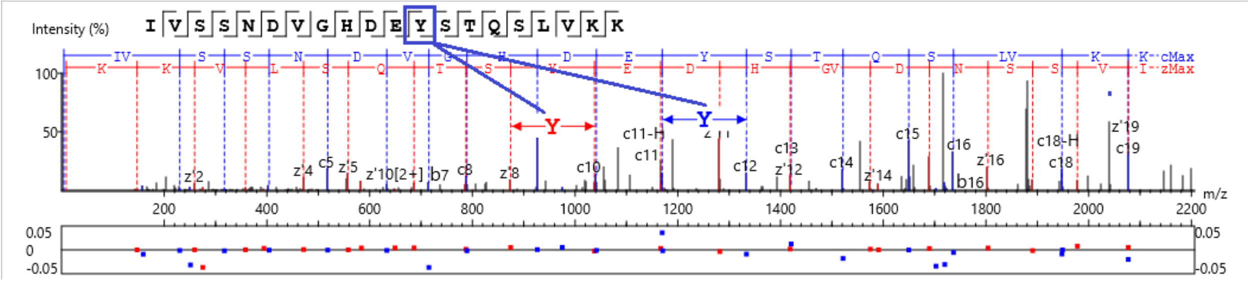

Zoom to a m/z region: Drag horizontally from the start m/z and to the end m/z with left mouse button.

Zoom in/out smoothly: Place the cursor at a particular m/z value (right below the x-axis), zoom in/out by scrolling the mouse wheel.

Increase/Decrease peak intensity: Place the cursor in the spectrum and scroll the mouse wheel.

See the whole spectrum: Double click in the spectrum or click the 1:1 button.

7.10.5.2 Annotated Spectrum Chart - Spectrum Annotation Settings

Click on the button to show the spectrum annotation settings.

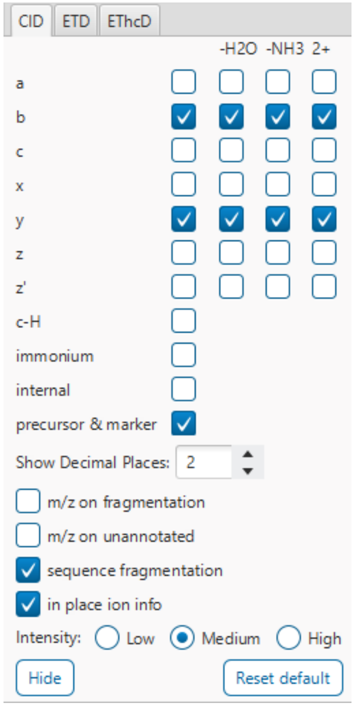

The spectra in the PEAKS results can be annotated by selecting ion types from the thorough collection of ions that PEAKS offers. The selected ion types will be displayed in the "Ion Match" table, as well. It is possible to annotate the spectrum with various ions for CID, EThcD and ETD. Each of these fragmentation modes can be set with different settings which will be shared with other results with the same fragmentation type.

To reset the defaults, click on the "Reset default" button.

Show Decimal Places: Select the number of decimal places that will appear in the ion table and the spectrum view. The default is set to two decimal places.

m/z on Fragmentation: Select this to display the m/z value on top of the annotated ions.

m/z on Unannotated: Select this to display the m/z value on top of the peaks without ion matches.

Sequence Fragmentation: Select this to display the sequence fragmentation on the top-left corner of the "Spectrum Annotation" view.

In Place Ion Info: Ion information, m/z value,and relative intensity are all displayed in a pop-up within the "Spectrum Annotation" view when this option is checked and the cursor is placed on a peak.

Intensity: Set the intensity threshold for spectrum annotation to low (2%), medium (5%), or high (10%).

7.10.5.3 Annotated Spectrum Chart - Additional Chart Controls

Click on or to toggle between absolute and percentage intensity values in the Y axis.

Click on to reset the zoom level of the annotated spectrum chart to the default.

Click on to zoom into the X axis by a factor of 2.

Click on to zoom into the Y axis by a factor of 2.

The "alignment" check box ☑ alignment allows user to display/hide how the fragment ions generated from the peptide align with the spectrum. N-terminal ions are shown in blue and C-terminal ions are shown in red.

The “error map” check box ☑ error map is used to show/hide the error map underneath the chart.

7.10.5.4 Annotated Spectrum Chart - Mirror Plot

Spectral Library results have a Mirror Plot view. Click on the button to switch to the Mirror Plot.

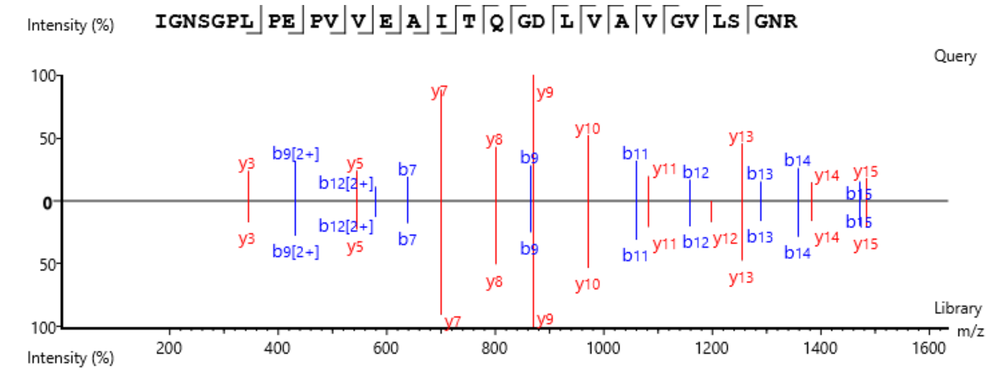

This view shows the ions from the query spectrum compared to the library spectrum.

7.10.6 Peptide View - Ion Match Table

The “Ion Match” tab in the bottom pane contains a table with possible fragment ions for the selected MS/MS scan. Each ion in the table shows the calculated mass, where if the fragment ion is present in the spectrum, its mass value is displayed in color. N-terminal ions are shown in blue and C-terminal ions are shown in red. A fragment ion is found when there is a matching peak within the mass error tolerance, as defined in the de novo sequencing parameters, and when relative intensity of the matching peak is at least 2%. The ion types displayed in the table can be configured in "Spectrum Annotation Settings”.

Ion Match | Survey

#	b	b-H2O	b-NH3	b(2+)	Seq	y	y-H2O	y-NH3	y(2+)	#
10	1079.503	1061.488	1062.498	540.248	E	1596.643	1578.636	1579.610	798.818	13
11	1265.576	1247.569	1248.557	633.292	W	1467.601	1449.588	1450.585	734.297	12
12	1394.626	1376.616	1377.600	697.813	E	1281.521	1263.512	1264.488	641.266	11
13	1481.659	1463.648	1464.632	741.329	S	1152.480	1134.469	1135.461	576.736	10
14	1596.682	1578.671	1579.655	798.841	N(+0.98)	1065.449	1047.440	1048.427	533.228	9
15	1653.703	1635.693	1636.676	827.352	G	950.422	932.407	933.390	475.709	8
16	1781.773	1763.751	1764.735	891.381	Q	893.397	875.388	876.370	447.198	7
17	1878.815	1860.804	1861.788	939.907	P	765.341	747.326	748.315	383.169	6
18	2007.857	1989.847	1990.830	1004.429	E	668.288	650.274	651.258	334.642	5
19	2122.880	2104.870	2105.853	1061.940	N(+0.98)	539.245	521.231	522.222	270.124	4
[illegible]	[illegible]	[illegible]	[illegible]	[illegible]	[illegible]	[illegible]	[illegible]	[illegible]	[illegible]	[illegible]

Note: Uncheck the alignment checkbox in the spectrum annotation view to see the highlighted fragment ions more easily.

7.10.6.1 Ion Match Table - Library matching table

While in mirror plot view, the Ion Match table updates to display the ion matches between fragment query ions and library ions.

Library intensity and Query intensity are reported relative to the highest intensity ion. All columns can be rearranged and sorted.

7.10.7 Peptide View - LC/MS Snapshot

The LC-MS Snapshot displays a zoomed-in view of the precursor scan in the LC-MS heat map 2-D view. The peptide feature is highlighted in purple and the selected PSM marker is blue. The span of the orange line in the LC-MS Snapshot designates the isolation window.

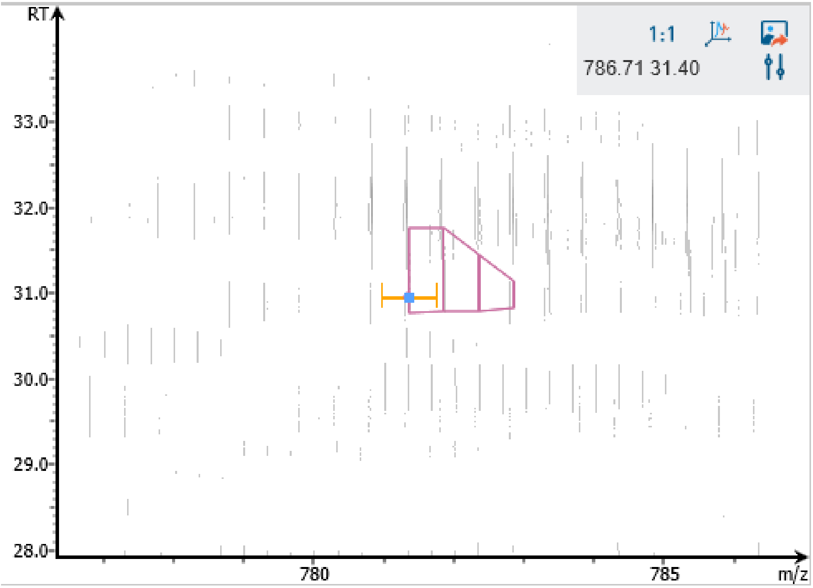

Users can change the view from the 2-D heat map to a 3-D intensity view by clicking the intensity view button in the upper right-hand corner of the LC-MS Snapshot. In addition, users can export the LC-MS heat map by clicking the export image button in the upper right-hand corner of the LC-MS Snapshot.

Expand the options dropdown by clicking to reveal a search option to specify a *m/z RT* (ensure there is a space between these two numbers) location to zoom into. There is also a Peak slider filter to filter out low intensity peaks and a feature area filter to filter out low area features.

7.10.8 Peptide View - Precursor Profile

The Precursor Profile, or eXtracted Ion Chromatogram (XIC) of the precursor ion, graphs the elution profile of the selected peptide feature vector over the retention time range where it was identified. The graph is plotted with the retention time along the x-axis and the intensity along the y-axis.

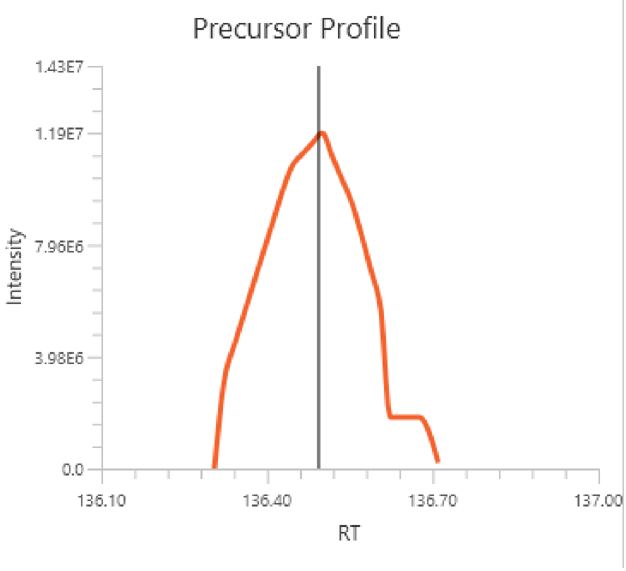

7.10.9 Peptide View - Fragment Ion XIC

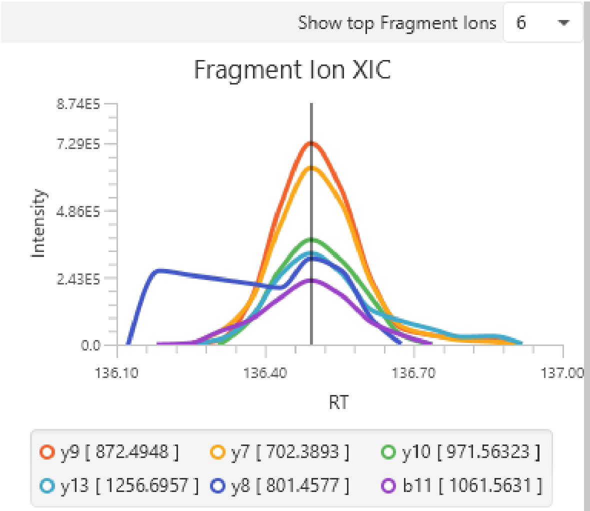

An eXtracted Ion Chromatogram (XIC) chart that displays the shape of each identified fragment ion across all scans for the selected PSM. Select the number of ions to display in the chart using the “Show top Fragment Ions” dropdown.

7.11 DIA Database Search Result - Exporting

In Project View, double-click on Export to open the Export node.

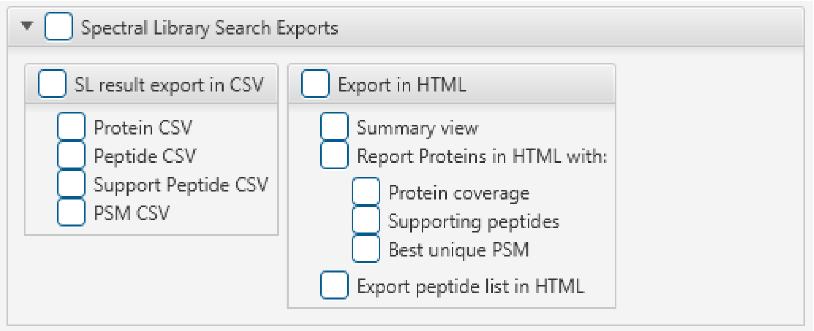

7.11.1 DIA Database Search - Exporting - comma separated values (CSV) format

There are several options for exporting the PEAKS Identification results in CSV format:

- **Protein CSV:** The list of protein identifications, filtered by the protein filters in the Summary view and will be saved to proteins.csv.
- **Peptide CSV:** All of the identified peptides and details will be saved to peptides.csv.
- **Support peptides CSV:** A list of supporting peptides of each protein identification, filtered by the protein filters in the "Summary" view and will be exported to protein-peptides.csv. This usually contains more entries than the Peptide table as a peptide can be identified in several proteins and, therefore, can be reported multiple times in this file.
- **PSM CSV:** The peptide-spectrum matches (PSMs) will be exported to DB search psms.csv. Peptides differentiated with only I/L isoform are represented by separate entries. As a result, the number of entries in this file might be bigger than the number of PSMs mentioned in the Summary view.

7.11.2 DIA Database Search - Exporting - HTML format

Export in HTML: This will generate single or multiple HTML reports in the specified location.

The following exporting options are available:

- **Summary view:** The “Summary” view page will be saved as a summary.html file in HTML format in the specified location.
- **Protein coverage:** The coverage pane will be saved for each protein.
- **Supporting peptides:** A list of supporting peptides will be saved for each protein.
- **Best unique PSM:** The best unique PSM will be saved for each protein.
- **Export peptide list in HTML:** creates a separate HTML-containing peptide identifications.

An individual protein will have its own HTML output file, where the corresponding protein coverage, the supporting peptides, and the best unique PSM are gathered.

7.12 Understanding PEAKS DIA De Novo Results

After the DIA de novo sequencing is complete, De Novo result nodes are generated. Double-click the De Novo node to examine the analysis report. The analysis report is presented in two pages:

- Summary: This shows an outline of the De Novo results with key statistics. The overall quality of the experiment can be examined and the filters for de novo ALC can be adjusted.

- De novo: This shows a list of peptide sequences that pass the ALC threshold detected by de novo sequencing.

The de novo view displays the de novo sequencing results. The table on the top displays de novo sequences, while the bottom section provides additional information about the peptide-spectrum match. The results can be filtered and exported.

7.13 DIA De Novo - Summary View

The DIA De Novo **Summary View** is similar to the PEAKS Deep Novo sequencing result.

In the summary tab, A PEAKS Studio DIA de novo sequencing result can be filtered using Average Local Confidence (ALC) score. Low quality deep novo sequences can be filtered out by specifying a minimal threshold of the ALC score. The purpose of filtering is to remove poor sequences in which residues are incorrectly identified. By default, the ALC threshold is set to 50%. After changing the threshold, click Apply to apply the new filter. The result in the De novo result view will be updated accordingly.

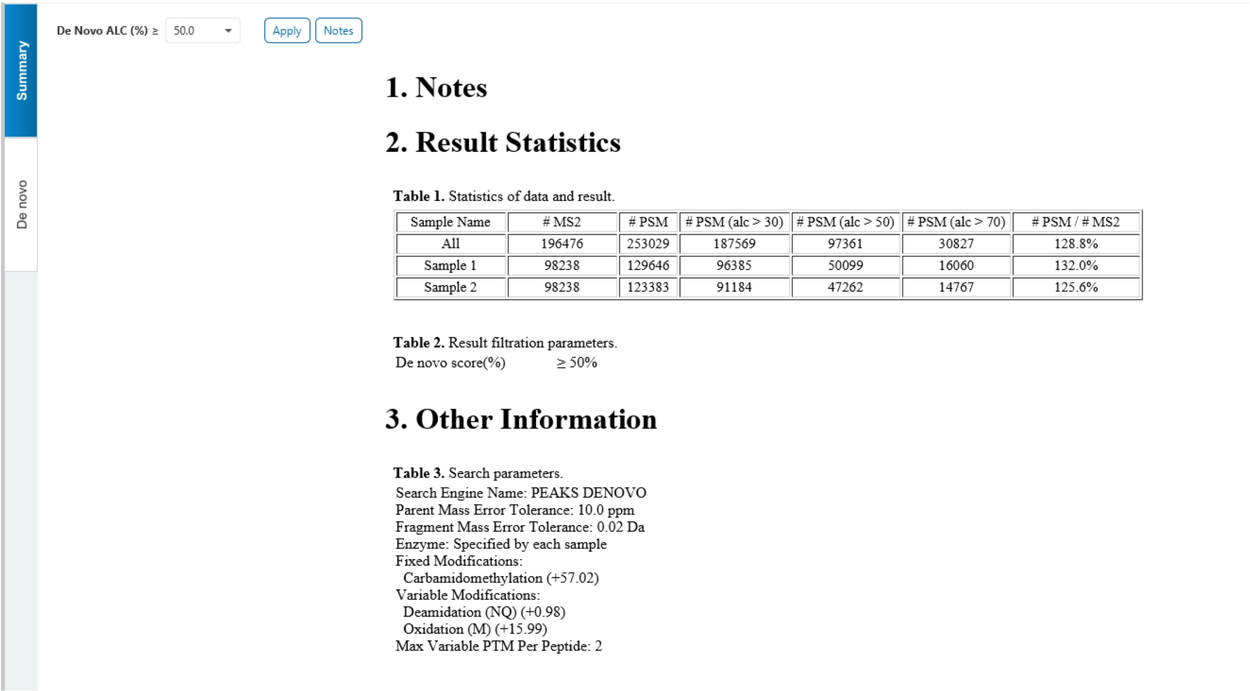

1. Notes

2. Result Statistics

Table 1. Statistics of data and result.

Sample Name	# MS2	# PSM	# PSM (alc > 30)	# PSM (alc > 50)	# PSM (alc > 70)	# PSM / # MS2
All	196476	253029	187569	97361	30827	128.8%
Sample 1	98238	129646	96385	50099	16060	132.0%
Sample 2	98238	123383	91184	47262	14767	125.6%

Table 2. Result filtration parameters.
De novo score(%) ≥ 50%

3. Other Information

Table 3. Search parameters.
Search Engine Name: PEAKS DENOVO
Parent Mass Error Tolerance: 10.0 ppm
Fragment Mass Error Tolerance: 0.02 Da
Enzyme: Specified by each sample
Fixed Modifications:
Carbamidomethylation (+57.02)
Variable Modifications:
Deamidation (NQ) (+0.98)
Oxidation (M) (+15.99)
Max Variable PTM Per Peptide: 2

Click on Notes to open the Notes dialog to save useful information regarding this Analysis. The Notes will appear in Section 1 of the summary page at the top.

Section 2 Result Statistics gives key statistics in Table 1 on the number of MS2 scans and # PSMs total as well as with different ALC thresholds for each sample. The #PSM / #MS2 provides a de novo sequencing identification rate metric for each sample.

Table 2 simply shows what is the current De novo ALC filter set. This is placed here so that when the Summary page is exported, the viewer will know what the ALC threshold was at the time.

Section 3 Other Information shows the parameters that were set when the analysis was ran.

7.14 DIA De Novo result - De Novo View

The **De novo View** displays the DIA de novo sequencing results in greater detail, as shown in the next figure. The table on the top displays de novo sequences, while the bottom section provides additional information about the peptide-spectrum match.

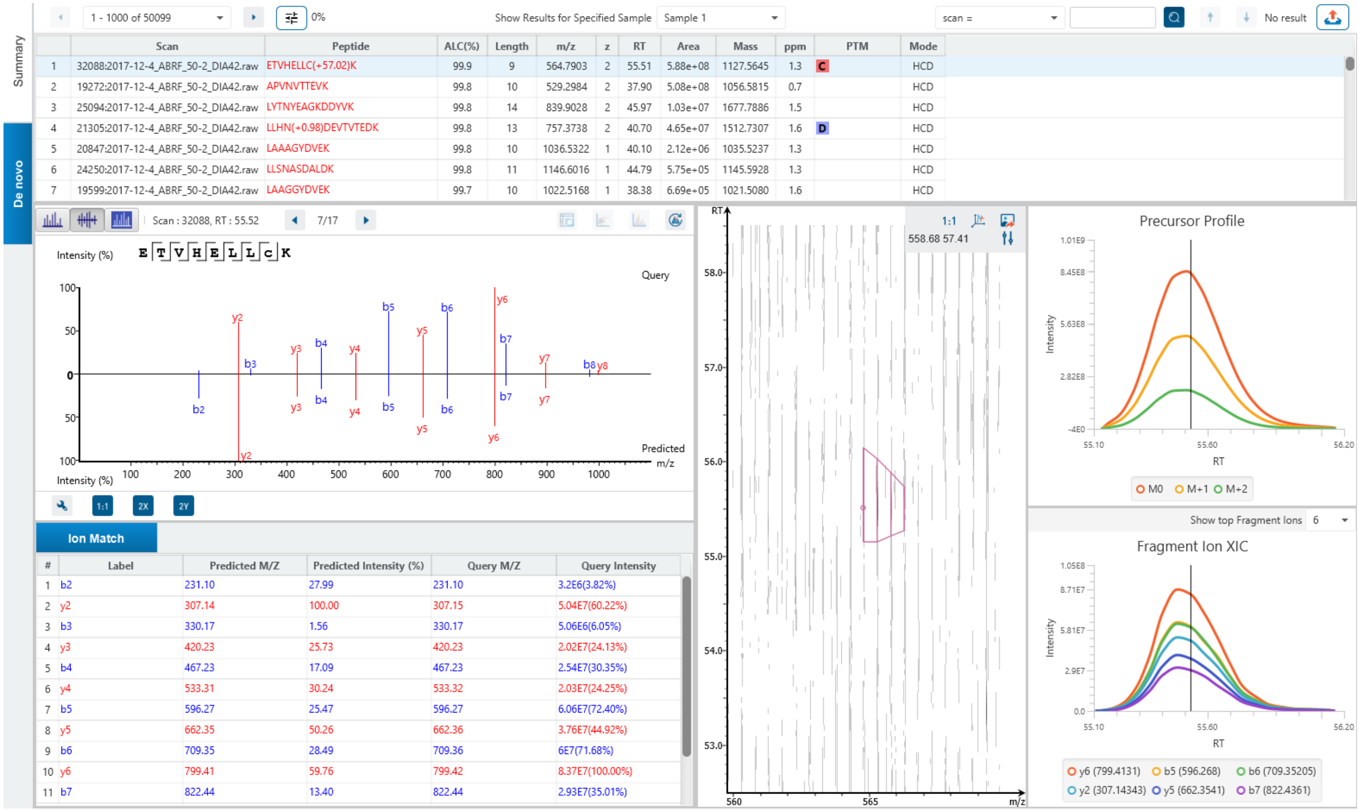

7.14.1 De Novo View - De novo table

The following list describes the contents in each of the columns of the Denovo table:

- **Scan:** The scan number and data file it belongs to. The scan number is a unique index for tandem mass spectra in the data.
- **Peptide:** The amino acid sequence of the peptide as determined by de novo sequencing. A modified residue is followed by a pair of parentheses enclosing the mass of that modification.
- **ALC(%):** The average local confidence. ALC is calculated as the total of the residue local confidence scores in the peptide divided by the peptide length. The higher the score the more confident the denovo candidate.
- **Frame:** This column is only displayed for diaPASEF data. A unique identifier is assigned for each frame obtained from the raw data.
- **Length:** The number of amino acids in the sequence backbone.
- **m/z:** The precursor mass-to-charge ratio.
- **z:** The precursor charge.
- **RT:** The retention time (elution time) of the spectrum as recorded in the data.
- **1/k0 Range:** This shows the ion mobility range associated with precursor. This column is only shown if the analysis contains diaPASEF data.

- **Area:** The area under the curve of the peptide feature found at the same m/z and retention time as the MS/MS scan. This can be used as an indicator of the abundance of the peptide.
- **Mass:** The monoisotopic mass of the peptide.
- **ppm:** The precursor mass error, calculated as $10^6 \times$ (precursor mass - peptide mass) / peptide mass.
- **PTM:** The types and the numbers of modifications present in the peptide shown in color-coded icons.
- **Mode:** The fragmentation mode in which de novo sequencing is performed by the algorithm.

Click on any of the column headers to sort the column.

To copy the sequence, right click on a row (or multiple rows) in the table and select “Copy selected peptide sequence(s)”. Another option is to mouse over any cell in the table and hitting Ctrl + C on your keyboard to copy the contents of those selected cells.

In the table, Amino acids in deep novo sequences are color-coded according to their local confidence scores. Red represents a very high confidence (greater than 90%); purple represents a high confidence (80 to 90%); blue represents a medium confidence (60 to 80%); and black represents low confidence (less than 60%). Hover the cursor over the sequence to view local confidence scores of each individual amino acid.

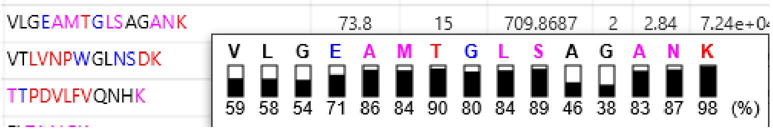

7.14.2 De Novo View - De Novo table controls

The menu above the De Novo table provides options to scroll through the table of deep novo peptides, filter for mass tags, switch between samples, search for specific entries, and export the table.

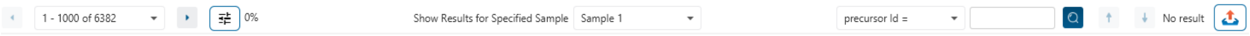

7.14.2.1 De Novo table controls - Sample Selection

Result for different samples can be viewed by selecting the sample using the "Show Results for Specified Sample" drop-down menu.

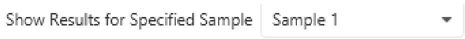

7.14.2.2 De Novo table controls - Pagination

1000 rows are visible at any one time in the De Novo table. Use the top left Pagination options to switch between result records.

7.14.2.3 De Novo table controls - Mass Tag Filter

Click on 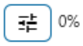 to use the mass tag confidence slider filter which will replace amino acids in the sequence with their mass tag values if their local confidence does not meet the threshold.

7.14.2.4 De Novo table controls - Search function

Use the search function to search for scan # or precursor ID, m/z, RT, or sequence contains. Search results can be navigated between using the Up or Down arrows.

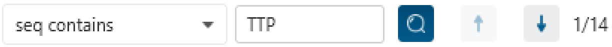

Options for searching include:

- **Scan =:** Search for the Scan number associated with each peptide. One scan can be associated to multiple peptides. For projects with multiple data files, the scan number is searched on every fraction. For timsTOF, there is the option to search for Precursor ID instead.

- **m/z ≈:** Search for the approximate m/z. The search will consider a range of m/z equal to the search value +/- 1 of the last significant digit.

  For example, searching for m/z ≈ 580.8 will find all m/z between [580.7, 580.9]

- **RT ≈:** Search for the approximate RT. Likewise, the search will consider a range of RT equal to the search value +/- 1 of the last significant digit set.

  For example, searching for RT ≈ 64 will find all m/z between [63, 65]

- **Seq contains:** Search for the sequence in the table. If specifying a sequence without glycans or PTMs, the results will include that sequence and any sequences with glycans or PTMs with the same backbone. If specifying a sequence with bracket and some mass value (like in the above image), then only sequence that have a substring with an exact match will be considered.

  Additionally, since the glycan peptide table shows both I/L and they can not be distinguished, then searching for I in the sequence is equivalent to searching for L in the sequence. i.e. Searching for “LVLK” is the same as searching for “LVIK”, “IVLK”, and “IVIK”

7.14.2.5 De Novo table controls - Table exporting

Click on the button to export the contents of the current table. This considers sorting applied to the table and is consistent with the view. There is the option to export the De Novo table and/or the De novo all candidates table in CSV format.

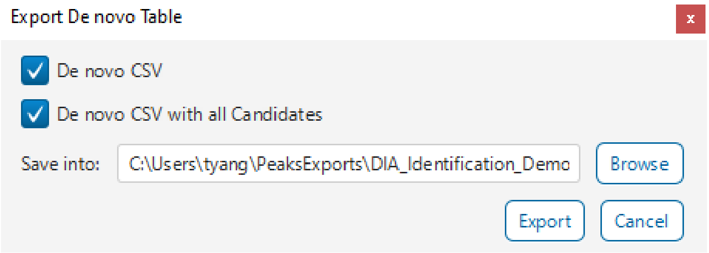

7.14.3 De Novo View - Deep Novo All Candidates

Click on the button to view the All de novo candidates for that spectrum. By default, the top 5 candidates are reported. The highest ALC scoring candidate is the one displayed in the De novo table.

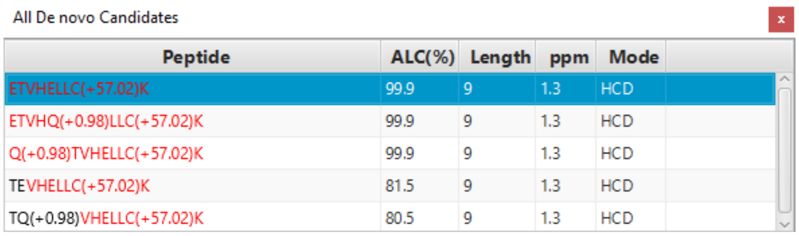

7.14.4 PSM selection

Click on the Left and Right arrow buttons to switch between PSMs related to the currently selected feature.

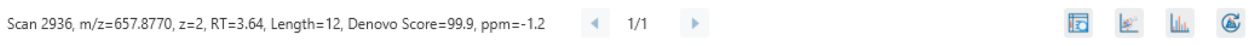

All other components in the De novo View interface will update according to the currently selected PSM.

For diaPASEF instruments, Frameis used rather than Scan number.

7.14.4.1 Show spectrum in Data View

Click on to open the Data Refine MS/MS tab (if not already opened) and jump to the corresponding selected Scan.

7.14.4.2 Show spectrum in LC/MS View

Click on to open the Data Refine LC/MS tab (if not already opened) and jump to the corresponding selected Scan, highlighting the De novo marker and the feature if present.

7.14.4.3 Show Raw Spectrum View

Click on to show the raw spectrum chart of the currently selected Scan.

7.14.5 Annotated Spectrum Chart

Click on button to switch from the Mirror plot view and annotated spectrum chart view.

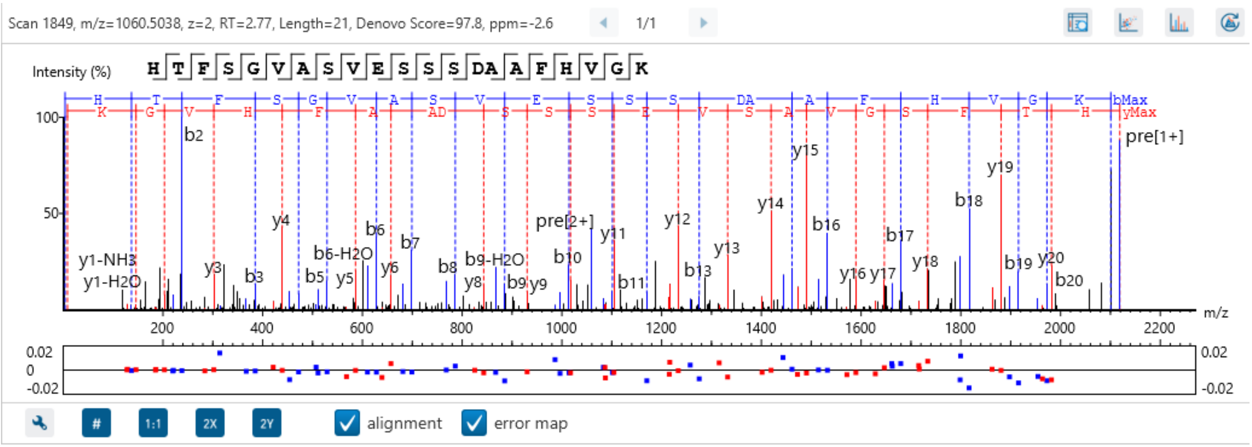

For the currently selected PSM, a graphical representation is shown in the form of an annotated spectrum chart. There are several controls provided for this view which will be explored in the following sections.

7.14.5.1 Annotated Spectrum Chart - Chart Navigation

Moving the cursor over the peptide sequence in the spectrum will show the mass transitions for a particular amino acid residue.

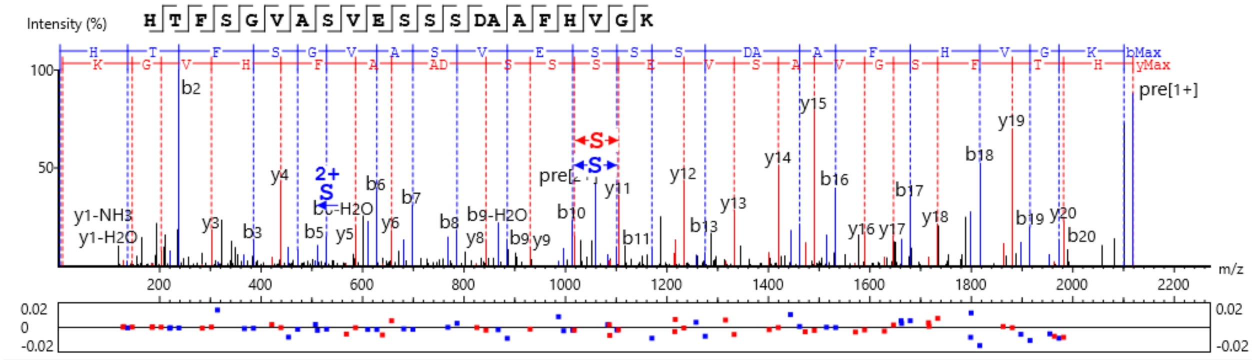

Zoom to a m/z region: Drag horizontally from the start m/z and to the end m/z with left mouse button.

Zoom in/out smoothly: Place the cursor at a particular m/z value (right below the x-axis), zoom in/out by scrolling the mouse wheel.

Increase/Decrease peak intensity: Place the cursor in the spectrum and scroll the mouse wheel.

See the whole spectrum: Double click in the spectrum or click the 1:1 button.

7.14.5.2 Annotated Spectrum Chart - Spectrum Annotation Settings

Click on the 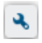 button to show the spectrum annotation settings.

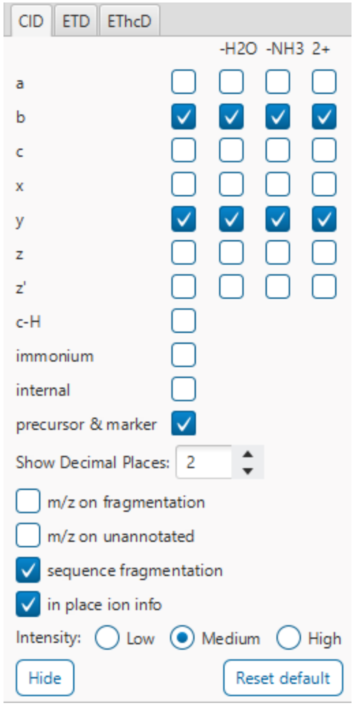

The spectra in the PEAKS results can be annotated by selecting ion types from the thorough collection of ions that PEAKS offers. The selected ion types will be displayed in the “Ion Match” table, as well. It is possible to annotate the spectrum with various ions for CID, EThcD and ETD. Each of these fragmentation modes can be set with different settings which will be shared with other results with the same fragmentation type.

To reset the defaults, click on the “Reset default” button.

Show Decimal Places: Select the number of decimal places that will appear in the ion table and the spectrum view. The default is set to two decimal places.

m/z on Fragmentation: Select this to display the m/z value on top of the annotated ions.

m/z on Unannotated: Select this to display the m/z value on top of the peaks without ion matches.

Sequence Fragmentation: Select this to display the sequence fragmentation on the top-left corner of the “Spectrum Annotation” view.

In Place Ion Info: Ion information, m/z value,and relative intensity are all displayed in a pop-up within the “Spectrum Annotation” view when this option is checked and the cursor is placed on a peak.

Intensity: Set the intensity threshold for spectrum annotation to low (2%), medium (5%), or high (10%).

7.14.5.3 Annotated Spectrum Chart - Additional Chart Controls

Click on # or % to toggle between absolute and percentage intensity values in the Y axis.

Click on 1:1 to reset the zoom level of the annotated spectrum chart to the default.

Click on 2X to zoom into the X axis by a factor of 2.

Click on 2Y to zoom into the Y axis by a factor of 2.

The "alignment" check box ☑ alignment allows user to display/hide how the fragment ions generated from the peptide align with the spectrum. N-terminal ions are shown in blue and C-terminal ions are shown in red.

The “error map” check box ☑ error map is used to show/hide the error map underneath the chart.

7.14.5.4 Annotated Spectrum Chart - Mirror Plot

Deep Novo results have a Mirror Plot view. Click on the button to switch to the Mirror Plot.

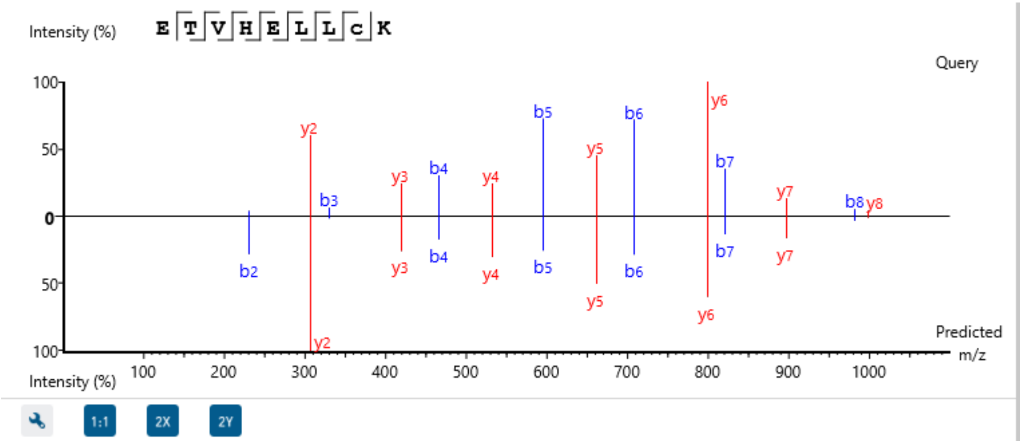

This view shows the ions from the query spectrum compared to the predicted spectrum.

7.14.5.5 Annotated Spectrum Chart - Pseudo Scan

Click on the button to show the Pseudo Scan.

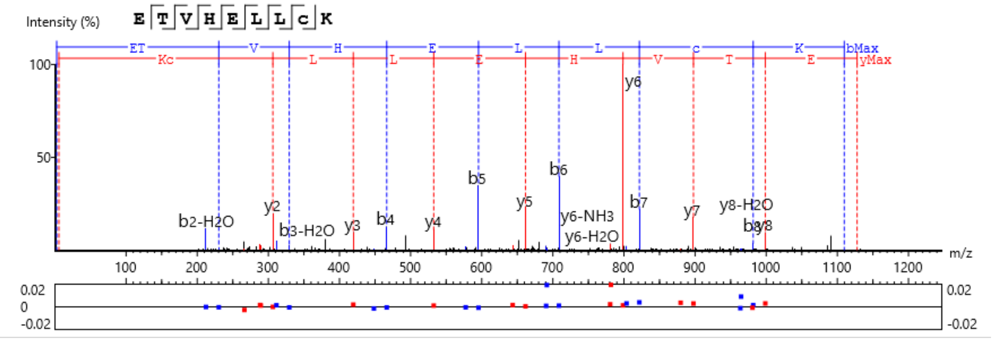

7.14.6 DIA De Novo Ion Match Table

The “Ion Match” tab in the bottom pane contains a table with possible fragment ions for the selected MS/MS scan. Each ion in the table shows the calculated mass, where if the fragment ion is present in the spectrum, its mass value is displayed in color. N-terminal ions are shown in blue and C-terminal ions are shown in red. A fragment ion is found when there is a matching peak within the mass error tolerance, as defined in the de novo sequencing parameters, and when relative intensity of the matching peak is at least 2%. The ion types displayed in the table can be configured in "Spectrum Annotation Settings" (see 7.14.5.2 Annotated Spectrum Chart - Spectrum Annotation Settings).

Ion Match

#	b	b-H2O	b-NH3	b(2+)	Seq	y	y-H2O	y-NH3	y(2+)	#
1	130.05	112.04	113.02	65.53	E					9
2	231.10	213.09	214.07	116.05	T	999.54	981.52	982.50	500.27	8
3	330.17	312.16	313.14	165.58	V	898.49	880.48	881.45	449.75	7
4	467.23	449.22	450.20	234.13	H	799.42	781.41	782.39	400.21	6
5	596.27	578.26	579.24	298.63	E	662.36	644.35	645.33	331.68	5
6	709.36	691.35	692.33	355.18	L	533.32	515.30	516.28	267.15	4
7	822.44	804.43	805.41	411.72	L	420.23	402.22	403.21	210.61	3
8	982.47	964.46	965.45	491.73	C(+57.02)	307.15	289.13	290.12	154.07	2
9					K	147.11	129.10	130.09	74.06	1

Note: Uncheck the alignment checkbox in the spectrum annotation view to see the highlighted fragment ions more easily.

7.14.6.1 Ion Match Table - Predicted matching table

While in mirror plot view, the Ion Match table updates to display the ion matches between fragment query ions and predicted ions.

Ion Match

#	Label	Predicted M/Z	Predicted Intensity (%)	Query M/Z	Query Intensity
1	b2	258.15	6.67	258.14	4.06E2(5.86%)
2	y2	310.18	48.38	310.17	2.02E3(29.15%)
3	b3	345.18	5.21	345.17	5.24E2(7.56%)
4	y3	409.24	20.13	409.24	1.51E3(21.79%)
5	b4	508.24	7.93	508.24	3.23E2(4.66%)
6	y4	572.31	38.81	572.30	3.5E3(50.51%)
7	b5	595.27	12.40	595.27	7.17E2(10.35%)
8	y5	671.38	14.42	671.37	9.8E2(14.14%)
9	b6	694.34	55.14	694.33	2.81E3(40.55%)
10	y6	758.41	41.06	758.40	2.88E3(41.56%)
11	b7	857.40	63.27	857.40	4.37E3(63.06%)

Predicted intensity and Query intensity are reported relative to the highest intensity ion. All columns can be rearranged and sorted.

7.14.7 DIA De Novo View - LC/MS Snapshot

The LC-MS Snapshot displays a zoomed-in view of the precursor scan in the LC-MS heat map 2-D view. The span of the orange line in the LC-MS Snapshot designates the isolation window.

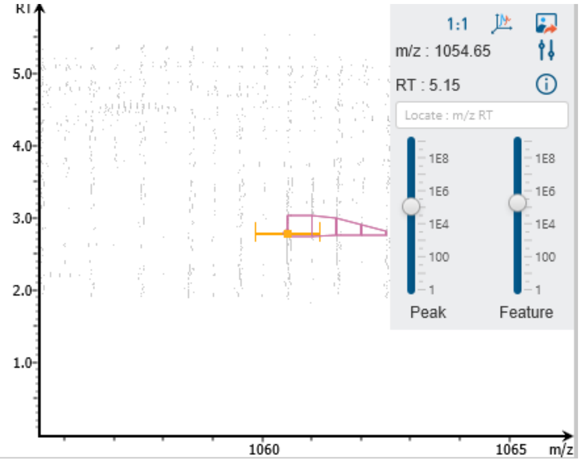

Users can change the view from the 2-D heat map to a 3-D intensity view by clicking the intensity view button in the upper right-hand corner of the LC-MS Snapshot. In addition, users can export the LC-MS heat map by clicking the export image button in the upper right-hand corner of the LC-MS Snapshot.

Expand the options dropdown by clicking to reveal a search option to specify a *m/z RT* (ensure there is a space between these two numbers) location to zoom into. There is also a Peak slider filter to filter out low intensity peaks and a feature area filter to filter out low area features.

7.14.8 DIA De Novo View - Precursor Profile

The Precursor Profile, or eXtracted Ion Chromatogram (XIC) of the precursor ion, graphs the elution profile of the selected peptide feature vector over the retention time range where it was identified. The graph is plotted with the retention time along the x-axis and the intensity along the y-axis. The first two isotopes are also drawn as M+1

and M+2 respectively.

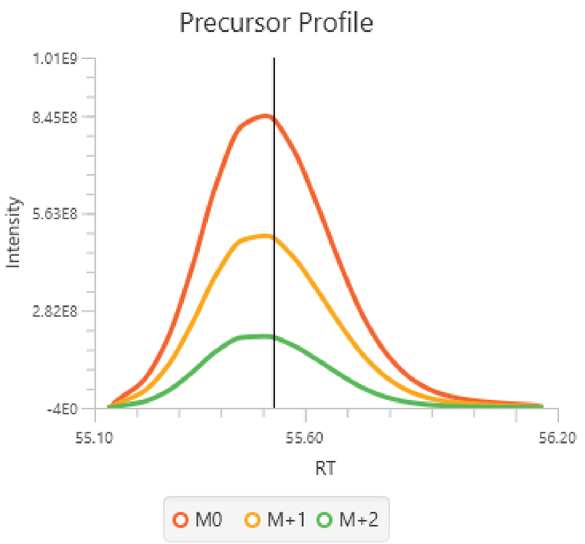

7.14.9 DIA De Novo View - Fragment Ion XIC

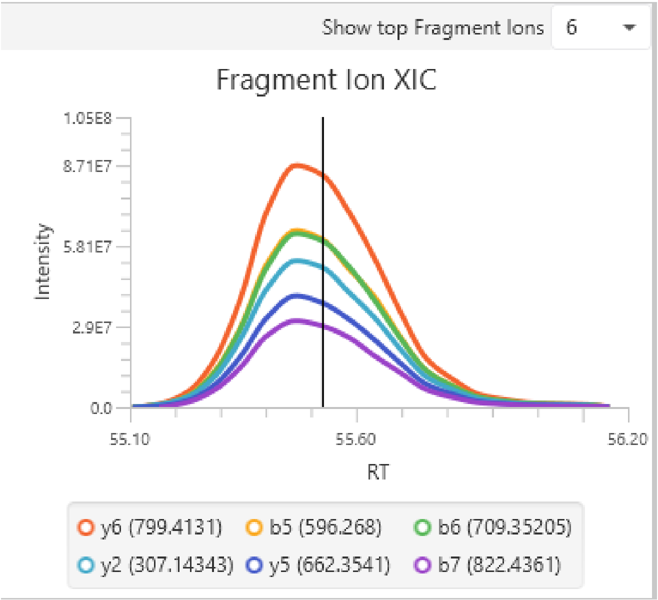

An eXtracted Ion Chromatogram (XIC) chart that displays the shape of each identified fragment ion across all scans for the selected PSM. Select the number of ions to display in the chart using the “Show top Fragment Ions” dropdown.

7.15 DIA De Novo result - Exporting

In Project View, double-click on Export to open the Export node.

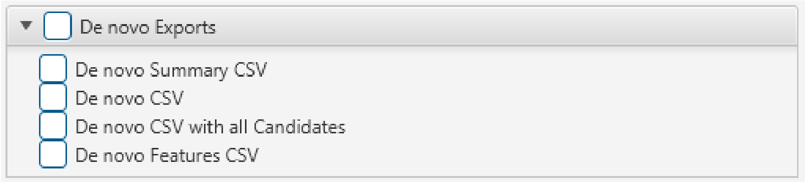

There are several options for exporting the Deep Novo results:

- De novo Summary HTML: Exports the Summary page including all figures in HTML format. The content will the same as shown in PEAKS Studio.
- De novo CSV: Exports the De Novo table. This export does not consider any sorting and will export a separate file for each sample.
- De novo CSV with all Candidates: Exports the De novo all candidates. This export does not consider any sorting and will export a separate file for each sample.
- De novo Features CSV: Exports the features list with de novo sequencing results. Each sample will have its own export file.

7.16 DIA Label Free Quantification result

The DIA LFQ result is very similar to the following chapter 8. Label Free Quantification (LFQ)

Label free quantification can be used as a quick profiling tool to examine the relative abundance of proteins in large proteomic datasets. In PEAKS Studio this quantification method is based on the relative abundance of peptide features detected in multiple samples. PEAKS Studio performs feature detection separately on each sample, and uses an EM (expectation-maximization) algorithm to detects and deconvolute overlapped features. Importantly, features of the same peptide from different samples are reliably aligned together (i.e. matched between runs) using a high performance retention time alignment algorithm to improve overall protein quantification rates.

Note: For details of the retention alignment algorithm, refer to the paper " *A Combinatorial Approach to the Peptide Feature Matching Problem for Label-Free Quantification", Bioinformatics, 2013, 10.1093 .*

To generate a DIA LFQ result, follow the steps in above section 7.1.4 DIA Quantification (DIA LFQ) Analysis Workflow and Parameter Settings

7.17 Understanding DIA LFQ Results

Once completed, a label free quantification result node will be added to the project tree. Double-click on this 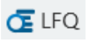

node to open the LFQ result that contains multiple tabs as described below:

Summary: The outline of the PEAKS LFQ search results with statistics. This is the place to examine the overall performance of the experiment and adjust filters.

Proteins: The quantified proteins with a list of supporting peptide features for each protein.

Peptides: The quantified peptides listed in a table. Extracted ion chromatogram (XIC), sample features and retention time alignment also displayed for each peptide.

7.18. DIA LFQ - Summary View

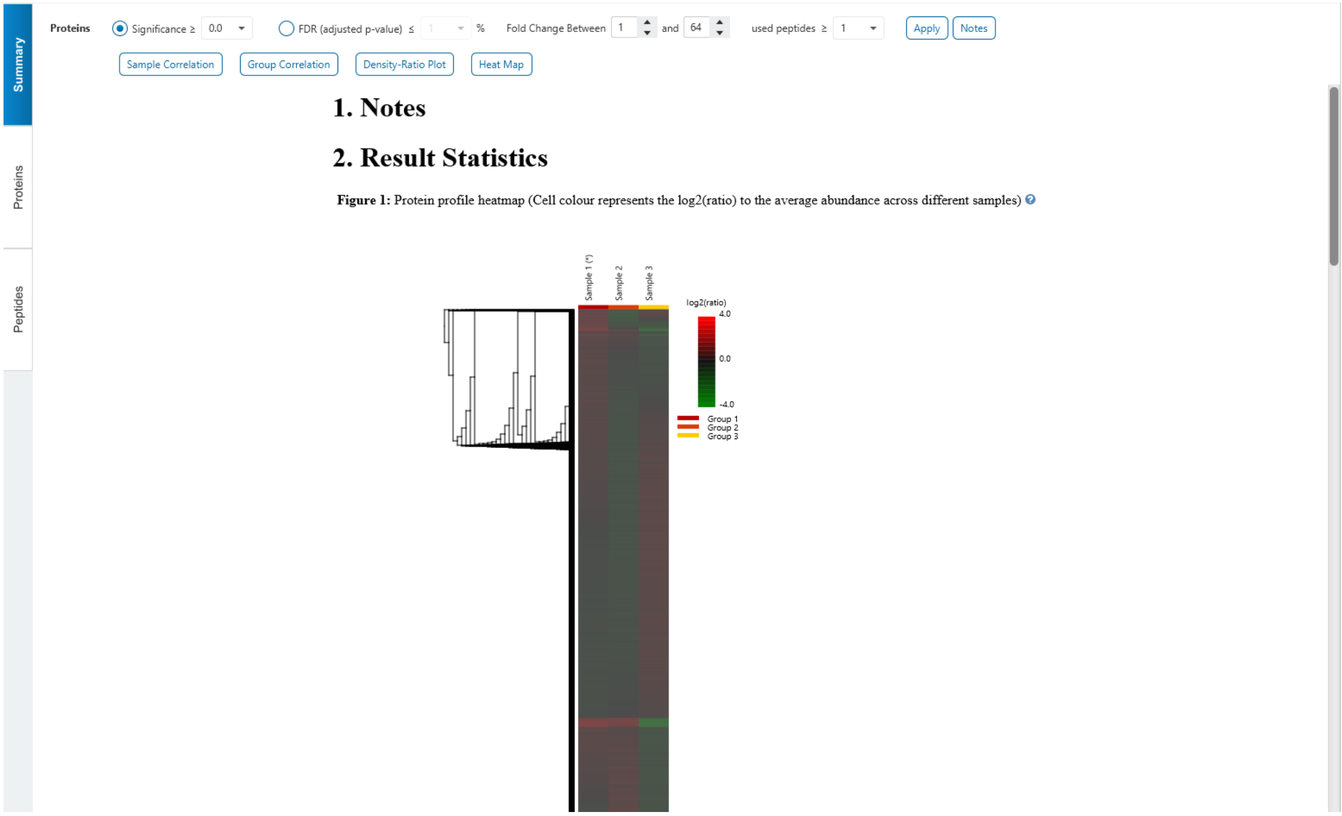

7.18.1 Summary View - Protein Filters

Filter settings used in the screenshot above means that no protein filter is applied, thus giving all quantifiable protein.

Alternately, parameters can be adjusted for additional analysis by updating the protein filters above.

Significance: Only protein groups with a significance above this threshold will be listed in the **Protein View**. The significance score is calculated as the -10log10 of the significance testing p-value. PEAKS provides ANOVA significance testing methods A significance score threshold of 20 is recommended, which equals to a significance testing p value of 0.01. Either this or "FDR (adjusted p-value)" can be selected to set a significance threshold.

Fold Change: Only proteins at or above this fold change threshold will be listed in the Protein View.

Used Peptides: Only proteins with at least this many number of used peptides will be listed in the Protein View.

Click on Apply to apply these filters changes to the result. This will update charts on the Summary View and will update the Protein table accordingly.

7.18.2 Summary View - Notes

Click on Notes to open the Notes dialog and save information about the result and saved in section 1. Notes on the Summary page.

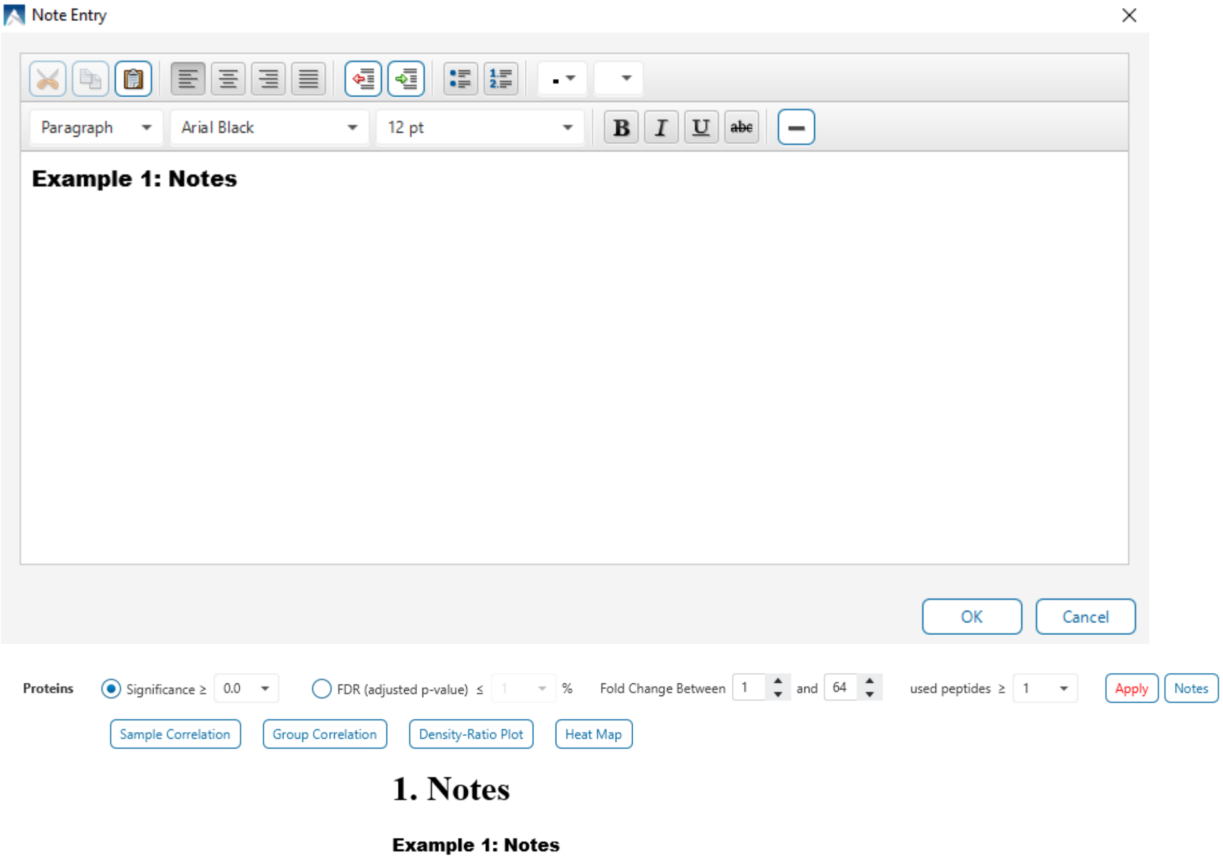
Note Entry
Paragraph
Arial Black
12 pt
Example 1: Notes
OK
Cancel
Proteins
Significance ≥
0.0
FDR (adjusted p-value) ≤
%
Fold Change Between
1
and
64
used peptides ≥
1
Apply
Notes
Sample Correlation
Group Correlation
Density-Ratio Plot
Heat Map
1. Notes
Example 1: Notes

7.18.3 Summary View - Heat Map

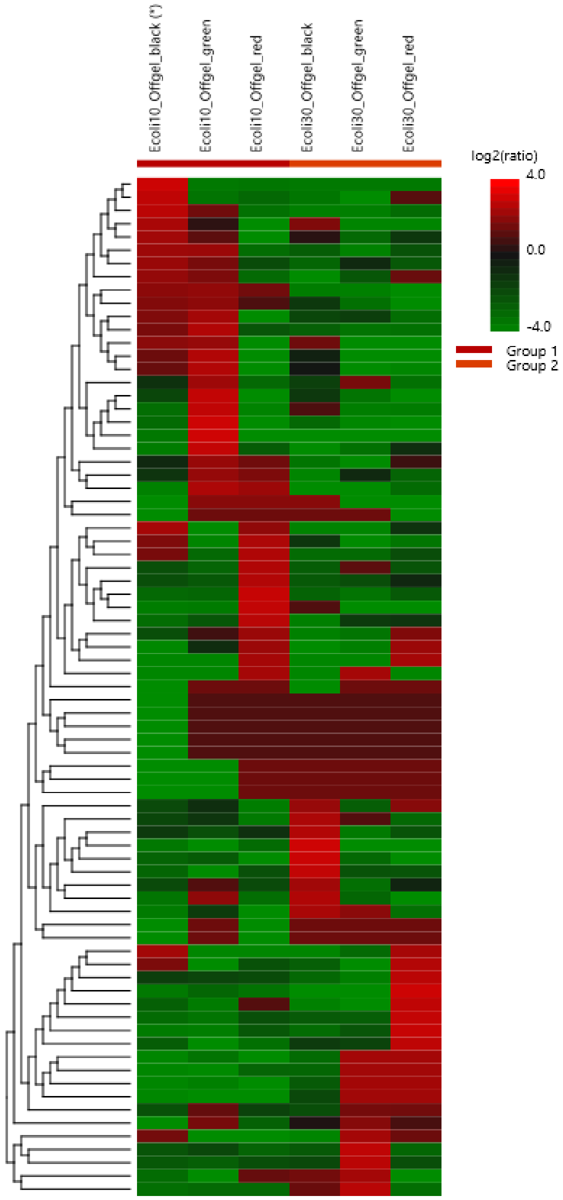

The heat map displays the protein groups that passed the filters. The relative protein abundance is represented as a heat map of the representative proteins of each protein group. These representative proteins are clustered if they exhibit a similar expression trend across the samples. The hierarchical clustering is generated using a neighbour-joining algorithm with a Euclidean distance similarity measurement of the log2 ratios of the abundance of each sample relative to the average abundance. Similarly, the conditions in different samples are clustered if they exhibit a similar expression trend across the protein groups. An interactive protein profile heat map is opened in a new window when the Heat Map button is clicked options.

Mouse over the dendogram in a section of the heatmap and click on it to open the Variation Trend chart.

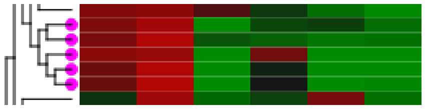

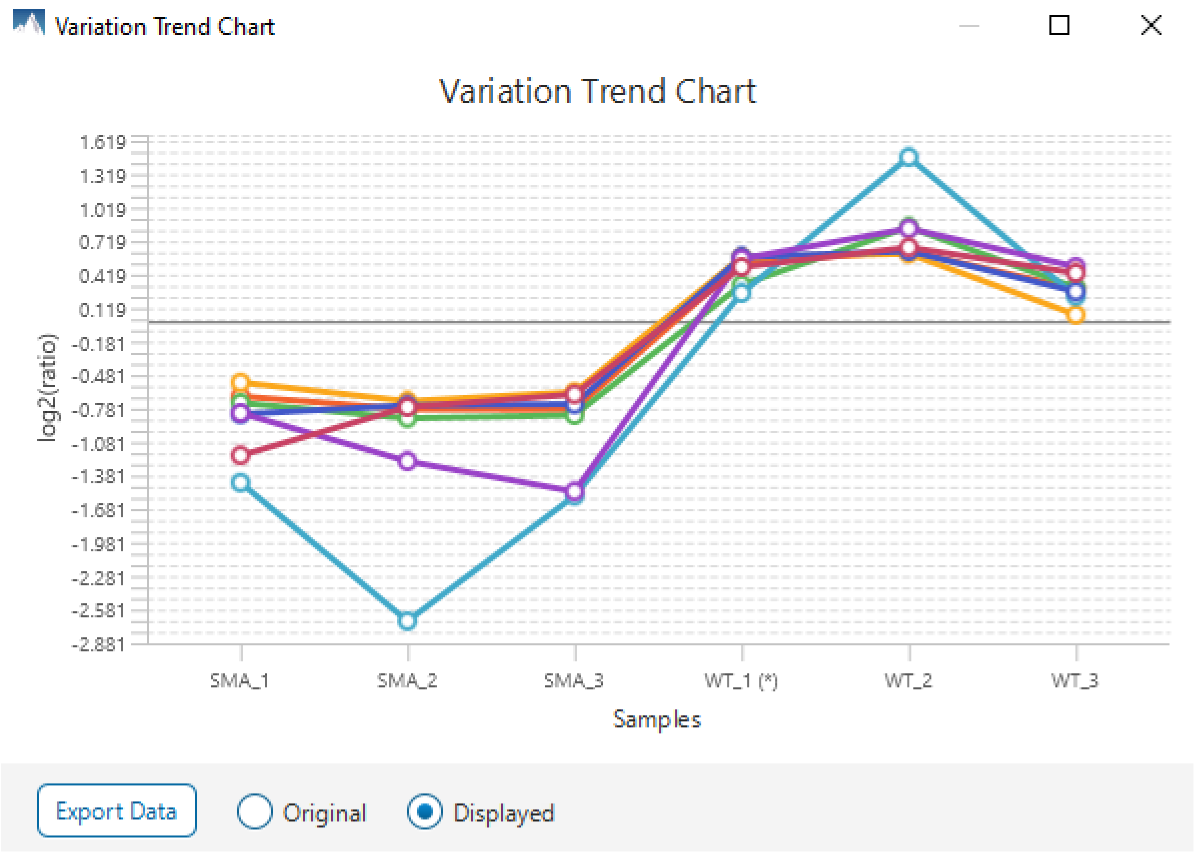

The Variation Trend chart displays the expression level of the selected proteins in the samples.

Mouse over points on the variation trend chart to see a tooltip of the protein, sample, and log2ratio.

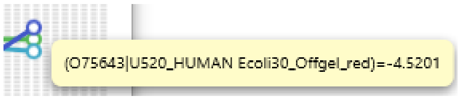

Click on the Export Data button to export the variation trend chart data in a text file format. When Displayed is selected, the log2 ratios will be in the export. When Original is selected, the original ratio values will be in the export instead.

7.18.4 Summary View - Density-Ratio Plot

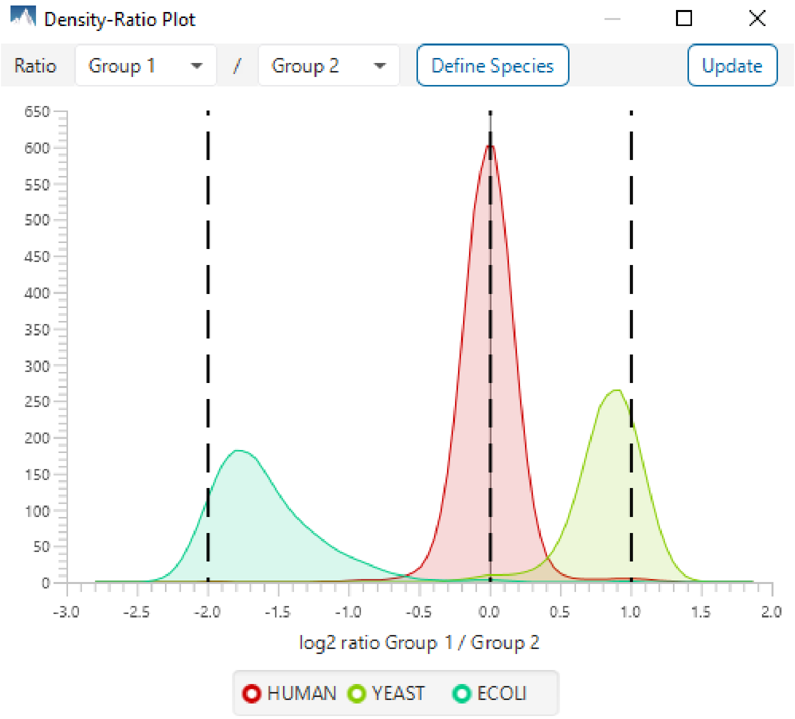

To view the group ratio distribution of the quantified proteins, click the Density-Ratio button. By default, all proteins will be shown. Click Define Species to group the different species together.

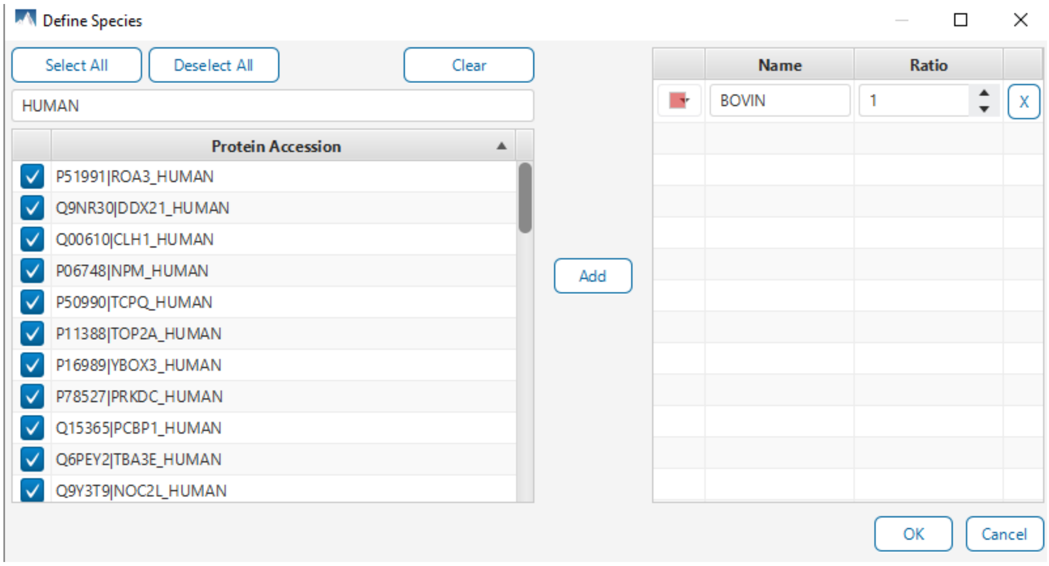

The proteins can be searched from the list by typing into the search box, marked and then added as a species group. The expected ratio of the species in the "Ratio" column can be edited to match with the experiment design. Once all the species are added and organized, click Ok to finish. In the plot window, click Update to apply the changes.

7.18.5 Summary View - Sample Correlation plot

To view the abundance correlation plot for the detected features and proteins, click the Sample Correlation button from the Summary View title bar. A Correlation Plot Options window will pop-up to select which samples to include.

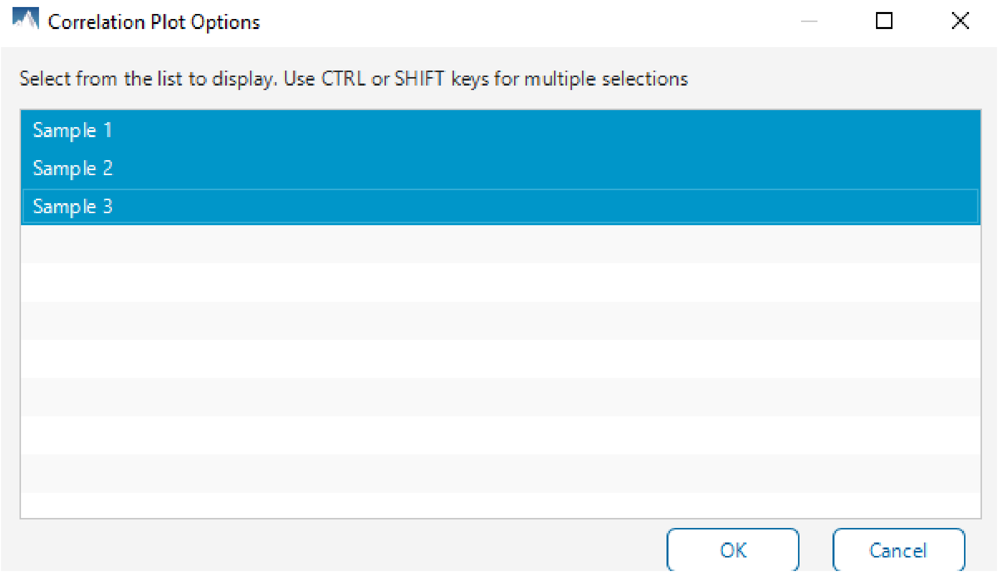

By default, the first 5 samples are selected. At most 5 samples can be used to generate the correlation plot, and at least 2 samples must be selected.

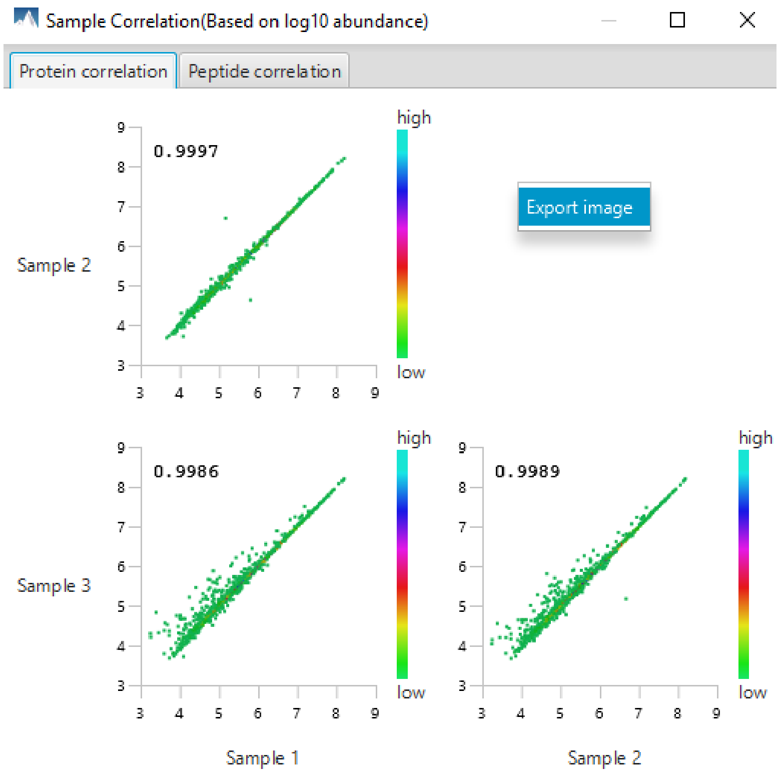

The Protein Correlation and Peptide Correlation plots will be displayed for the samples selected. The Pearson's correlation score indicates the reproducibility of the experiment based on a selected pair of samples. To export the image right click on the plots and click on "Export Image".

7.18.6 Summary View - Group Correlation plot

To view the abundance correlation plot for the detected features and proteins, click the Group Correlation button from the Summary View title bar. A Correlation Plot Options window will pop-up to select which groups to include.

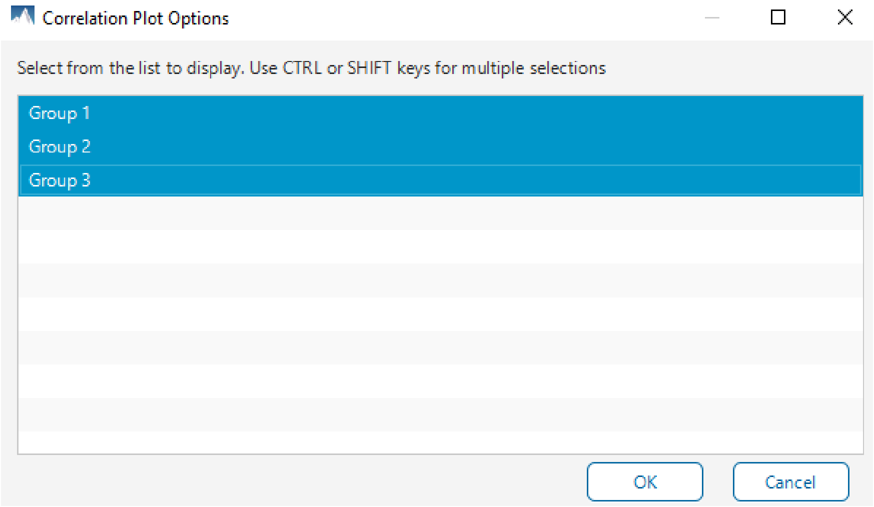

By default, the first 5 groups are selected. At most 5 groups can be used to generate the correlation plot, and at least 2 groups must be selected.

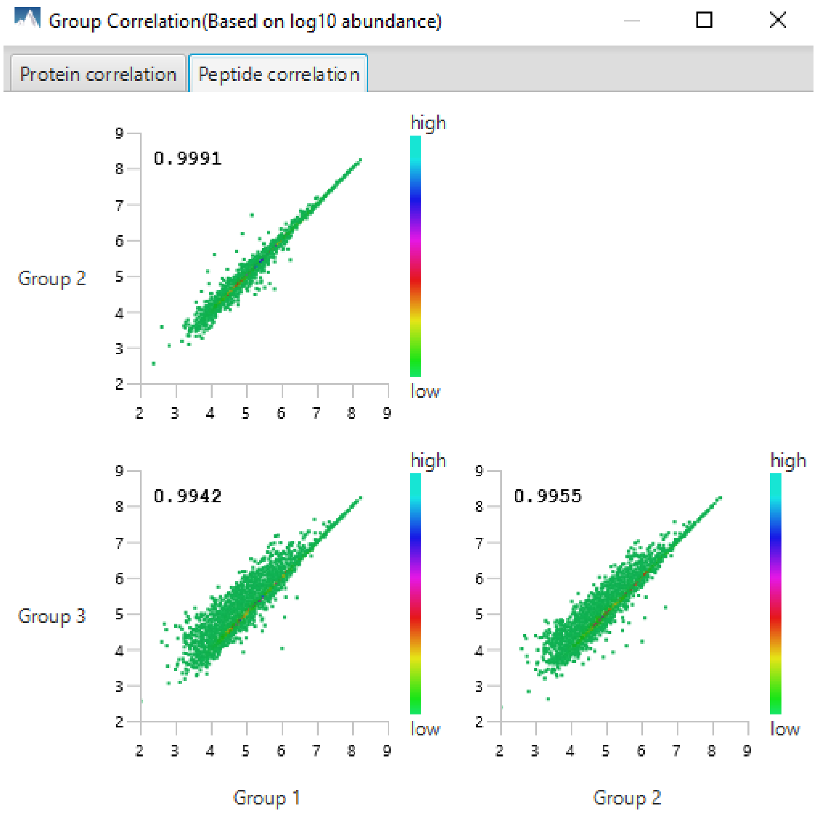

The Protein Correlation and Peptide Correlation plots will be displayed for the samples selected. The Pearson's correlation score indicates the reproducibility of the experiment based on a selected pair of samples. To export the image right click on the plots and click on "Export Image".

7.18.7 Summary View - Volcano plot

Figure 2. volcano plot combines a statistical test with the magnitude of the change enabling quick visual identification of those data-points that display large-magnitude changes and that are also statistically significant. The volcano plot in Figure 2(a) plots significance versus fold-change of the quantified proteins.

Horizontal broken grey line: The selected significance threshold

Vertical broken grey lines: The selected fold change thresholds

Plotting points in this way results in two regions of interest in the plot: those points that are found towards the top of the plot that are far to either the left or the right side. These represent values that display large magnitude fold changes (hence being left or right of center) as well as high statistical significance (hence being towards the top).

Figure 2. The volcano plot for proteins.

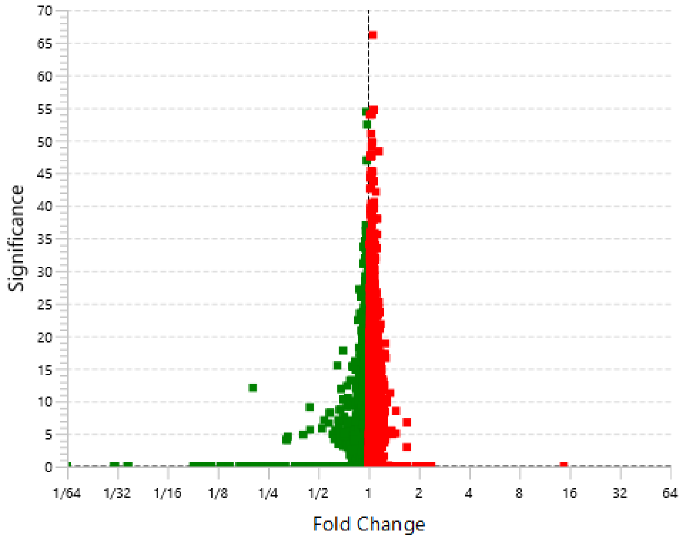

In this figure, the ratio is the group ratio set with respect to the base group. If more than two groups are present, the max ratio is used instead.

The Protein View contains an interactive volcano plot that can be hidden or displayed. Markers for the proteins that are above the set significance threshold will be displayed in colour. Scrolling the cursor over a marker will highlight it and, subsequently, clicking on this marker will highlight the protein in the interface.

7.18.8 Summary View - RT and m/z Shift Distribution

Figure 3(a) plots the retention time shift distribution to the base sample. Figure 3(b) plots the distribution of the m/z shift (in ppm) of the data with respect to the base sample. These two figures can be used to refine the parameter settings for label free quantification.

Figure 3. (a) RT shift distribution; **(b)** M/Z shift distribution.

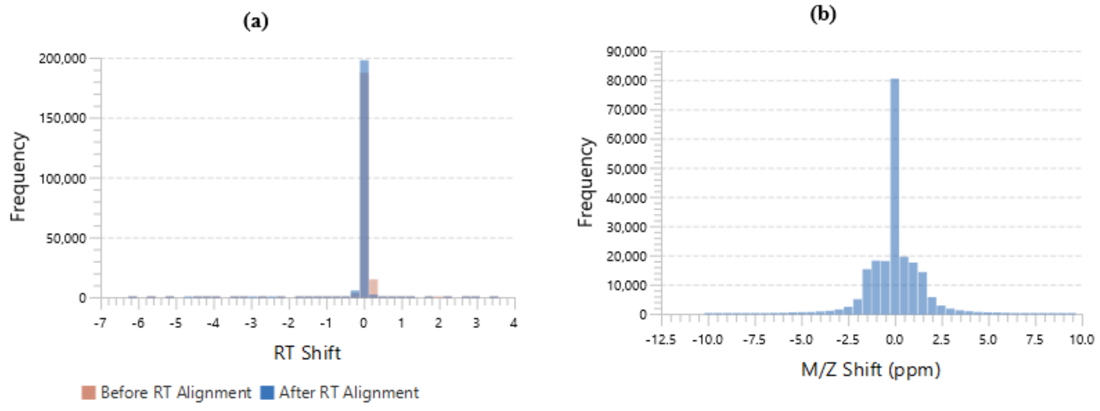

7.18.9 Summary View - Missing Value percentage chart

Figure 4 shows the percentage of missing values before and after ID transfer for each sample.

Figure 4. Percentage of missing values in each sample with or without ID transfer.

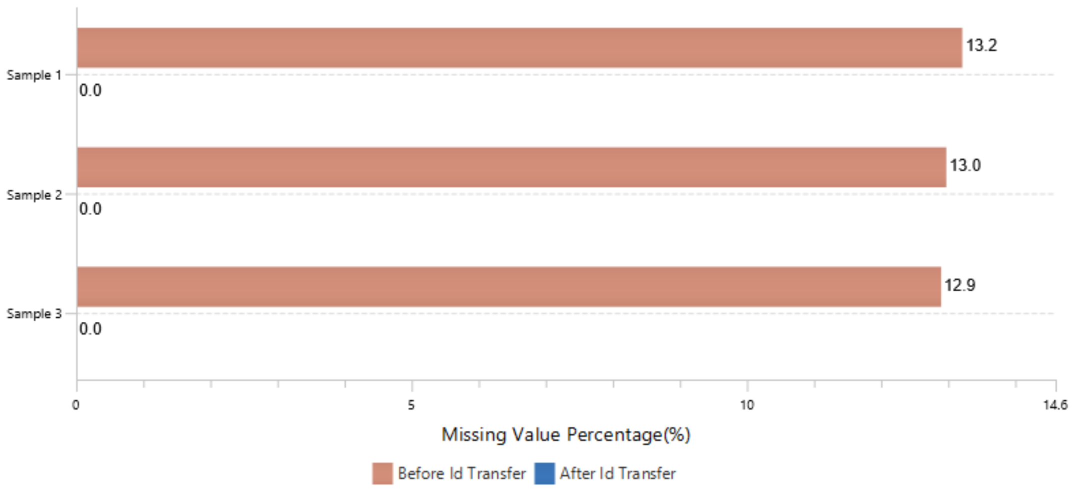

7.18.10 Summary View - Filtration parameters and statistics

Tables 1 and 2 list the filtration parameters and filtered results, respectively.

Table 1. Result filtration parameters.

Significance	≥ 0
Min Fold Change	≥ 1
Max Fold Change	≤ 64
Used peptides	≥ 1

Table 2. Statistics of filtered result.

Features	203883
Features with ID	177325
Feature vectors with ID	67961
Protein groups	5633

7.18.11 Summary View - Search parameters

Table 3 lists the parameters as set in the workflow.

Table 3. Search Parameters

Search Engine Name: PEAKS
Quantification type: Label free quantification
LFQ method: Identification directed quantification
Retention time shift tolerance: Auto detected
Feature intensity ≥ 100.0
Retention time range: [0.0 - Max]
Base sample: Sample 1
Peptide Feature Filter:
  Avg area ≥ 200.0
  Quality ≥ 20.0
  Charge between: [2 - 5]
  Peptide ID count ≥ 0 per group
  Detected in at least: 1 samples per group
Protein Filter:
  Significance method: ANOVA
  Modified Form Exclusion
  Remove Outlier
  Use Top 3 peptide
  Significance ≥ 0.0
  Fold change between: [1.0 - 64.0]
  Has at least: 1 used peptide
Normalization method: Use TIC
Samples: 3 samples in 3 groups
  Group 1:
    Sample 1
  Group 2:
    Sample 2
  Group 3:
    Sample 3

7.19 LFQ - Protein View

The **Proteins View** lists all the quantified proteins present in the sample, characterizes each protein at the amino acid level, and lists the supporting peptide features of each protein. It has six components:

Protein Table: Lists all the quantified proteins present in the sample.

Protein View Filters: Allows filtering the protein table to select specific proteins.

Protein Volcano Plot: Click on a marker that is above the significance threshold (the horizontal dashed line) set in the Summary View. Upon selection, the corresponding protein in the Protein Table will be highlighted.

PTM Profiling: Click on to open PTM profiling.

Coverage: Characterizes the protein sequence at the amino acid level. All the PTMs occurred on the protein sequence will be displayed together with the MS/MS data supporting the inference.

Peptides: Lists the supporting peptides identified from the selected proteins in the protein table.

7.19.1 Proteins View - Protein Table controls

At the top of the Proteins View, there are the options to filter, show/hide columns, search, and export the protein table.

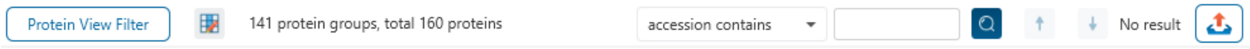

7.19.1.1 Protein Table controls - Protein View Filters

Click on the Protein View Filter button to open the Protein View Filter.

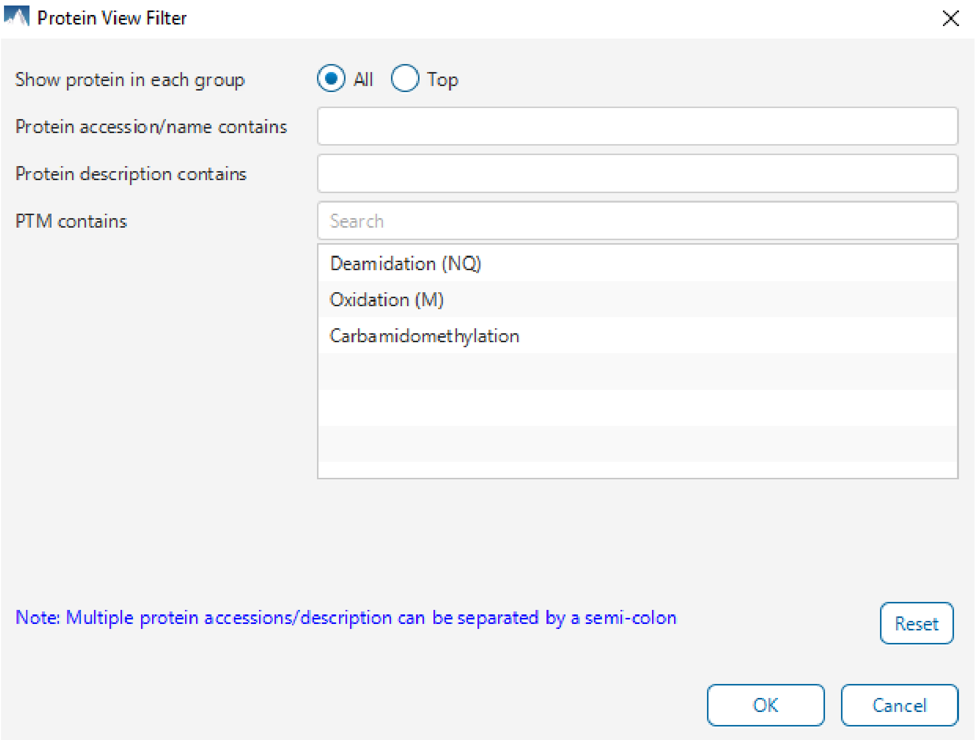

The following options are available for filtering the protein table:

Show All or Top proteins in each group: Adjust the protein list based on Protein grouping, top is selected by default. Proteins are grouped based on parsimony.

- **Top:** Show only the top proteins in each group. These proteins are supported by the most unique peptides in the group. Proteins in the group that share a subset of the unique peptides that support the top protein (subproteins) will not be displayed.
- **All:** Show all proteins in each group. Proteins are grouped together if they are supported by the same set or a sub-set of the top protein in the group. If a protein is supported by a peptide not supporting the top protein it will be added to a new group.

Protein accession/name contains: Restrict the protein table to only display protein IDs which contain the entered text in its accession/name.

Protein description contains: Only proteins with descriptions that contain the entered text will be included in the protein table if text is entered in this field.

Note: In these above two filters, multiple entries can be separated by a semi-colon.

PTM contains: Filter protein results by the presence of selected PTMs. Proteins that are supported by peptides containing modifications that match the selected PTMs will be included. If multiple PTMs match the modification name, they will all be included in the table. For SPIDER results, the mutations will also be listed in the PTM contains filter.

Click on the 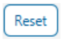

button to remove all filters.

Click on 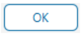

to apply the Protein View Filter to the Protein table.

Click on 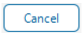

to discard changes and close the dialog.

7.19.1.2 Protein Table controls - Protein count

At the top of the protein table shows the count of protein groups and number of proteins in the table based on the current Protein View Filters.

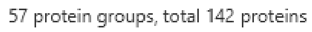

7.19.1.3 Protein Table controls - Optional columns

Click on the button to see a list of sample-based optional columns.

Select the corresponding sample-based and group-based ratio in the Protein Table view.

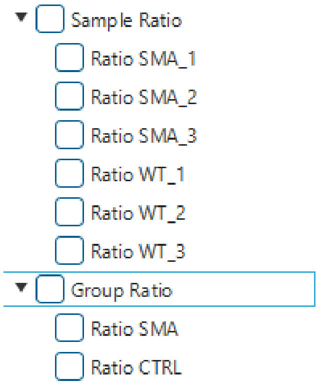

The exports will always include the contents of these optional columns even if they are hidden.

7.19.1.4 Protein Table controls - Protein Table Search Function

An easy-to-use search function is available for the protein table. The protein accession or description substring can be searched for, and all search results can be navigated through with the Up and Down arrows.

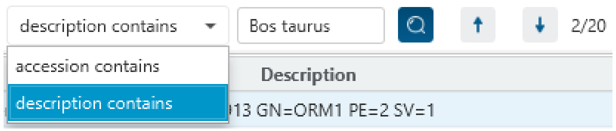

Protein accession/name contains: Restrict the protein table to only display protein IDs which contain the entered text in its accession/name.

Protein description contains: Only proteins with descriptions that contain the entered text will be included in the protein table if text is entered in this field.

7.19.1.5 Protein Table controls - Protein Table Export

Click on the button to export the contents of the current table. This considers the Protein View Filters, the sorting applied to the table and is consistent with the view. There is the option to export the Protein table and/or the Supporting peptide table in CSV format.

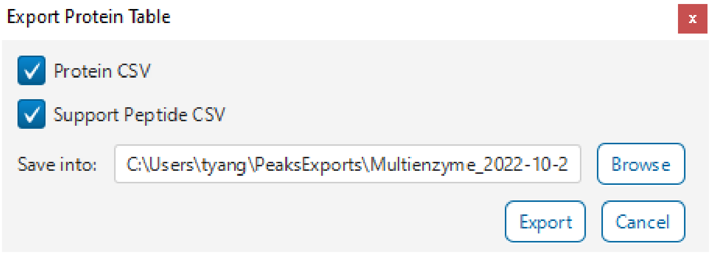

7.19.2 Proteins View - Protein Table

Each row in the table represents a group of proteins that are supported by a common set of peptides. To expand the group, click the right arrow button at the left.

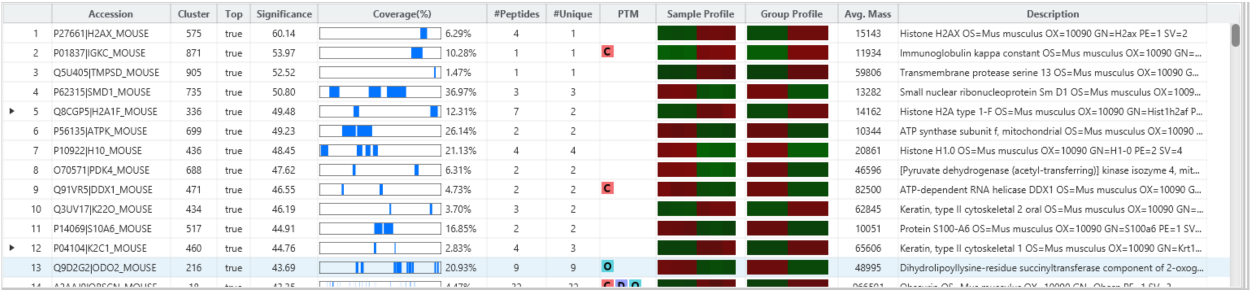

	Accession	Cluster	Top	Significance	Coverage(%)	#Peptides	#Unique	PTM	Sample Profile	Group Profile	Avg. Mass	Description
1	P27661|H2AX_MOUSE	575	true	60.14	6.29%	4	1				15143	Histone H2AX OS=Mus musculus OX=10090 GN=H2ax PE=1 SV=2
2	P01837|IGKC_MOUSE	871	true	53.97	10.28%	1	1	C			11934	Immunoglobulin kappa constant OS=Mus musculus OX=10090 GN=...
3	Q5U405|TMPSD_MOUSE	905	true	52.52	1.47%	1	1				59806	Transmembrane protease serine 13 OS=Mus musculus OX=10090 G...
4	P62315|SMD1_MOUSE	735	true	50.80	36.97%	3	3				13282	Small nuclear ribonucleoprotein Sm D1 OS=Mus musculus OX=1009...
▸ 5	Q8CGP5|H2A1F_MOUSE	336	true	49.48	12.31%	7	2				14162	Histone H2A type 1-F OS=Mus musculus OX=10090 GN=Hist1h2af P...
6	P56135|ATPK_MOUSE	699	true	49.23	26.14%	2	2				10344	ATP synthase subunit f, mitochondrial OS=Mus musculus OX=10090 ...
7	P10922|H10_MOUSE	436	true	48.45	21.13%	4	4				20861	Histone H1.0 OS=Mus musculus OX=10090 GN=H1-0 PE=2 SV=4
8	O70571|PDK4_MOUSE	688	true	47.62	6.31%	2	2				46596	[Pyruvate dehydrogenase (acetyl-transferring)] kinase isozyme 4, mit...
9	Q91VR5|DDX1_MOUSE	471	true	46.55	4.73%	2	2	C			82500	ATP-dependent RNA helicase DDX1 OS=Mus musculus OX=10090 G...
10	Q3UV17|K22O_MOUSE	434	true	46.19	3.70%	3	2				62845	Keratin, type II cytoskeletal 2 oral OS=Mus musculus OX=10090 GN=...
11	P14069|S10A6_MOUSE	517	true	44.91	16.85%	2	2				10051	Protein S100-A6 OS=Mus musculus OX=10090 GN=S100a6 PE=1 SV...
▸ 12	P04104|K2C1_MOUSE	460	true	44.76	2.83%	4	3				65606	Keratin, type II cytoskeletal 1 OS=Mus musculus OX=10090 GN=Krt1...
13	Q9D2G2|ODO2_MOUSE	216	true	43.69	20.93%	9	9	O			48995	Dihydrolipoyllysine-residue succinyltransferase component of 2-oxog...
[illegible]	[illegible]	[illegible]	[illegible]	[illegible]	[illegible]	[illegible]	[illegible]	[illegible]			[illegible]	[illegible]

The columns in the Protein Table are:

- **Accession:** The accession number of the protein as seen in the FASTA database.
- **Cluster:** A unique identifier that classifies which protein group the protein belongs to.
- **Top:** Indicates whether this protein is a top protein.
- **Significance:** The protein significance.
- **Coverage (%):** The percentage of the protein sequence that is covered by supporting peptides. The coverage is visualized by a colored bar. Light blue blocks indicate the parts of the sequence covered by low-confidence peptides. Dark blue blocks indicate the parts covered by high-confidence peptides. This is the total coverage including results from all samples.
- **#Peptides:** The number of high-confidence supporting peptides.
- **#Unique:** The number of high-confidence supporting peptides that are mapped to only one protein group. Unique peptides with same sequence but different modifications are only counted once in this number.
- **PTM:** The identified modifications displayed with color-coded icons.
- **Sample Profile:** The protein abundance among the samples is depicted as a heat map. Hold the cursor on a profile to view the sample channels, abundances, and ratios with respect to the base sample.
- **Group Profile:** The protein abundance among the groups is depicted as a heat map. The Group Profile is determined by calculated the total abundance (area) of supporting peptides within a group. Hold the cursor on a profile to view the group channels, abundances, and ratios compared to the group that contains the base sample.
- **Avg. Mass:** The protein mass calculated using the average mass.
- **Description:** The protein's header information as seen in the FASTA database.

Note: For #Peptides and #Unique, two peptides with the same starting and ending positions in the protein are counted as one, regardless of their PTM forms. This is to follow the MCP (Molecular & Cellular Proteomics) guidelines.

7.19.3 Proteins View - Coverage Pane

The Protein Coverage view visually maps the supporting peptides and de novo tags to the protein selected in the Protein table. It also shows all identified sites with modifications or mutations to assist with protein characterization at the amino acid level. The "Protein Coverage" view is composed of three major components - Protein Sequence Coverage, Coverage Control Panel, and Protein Tools.

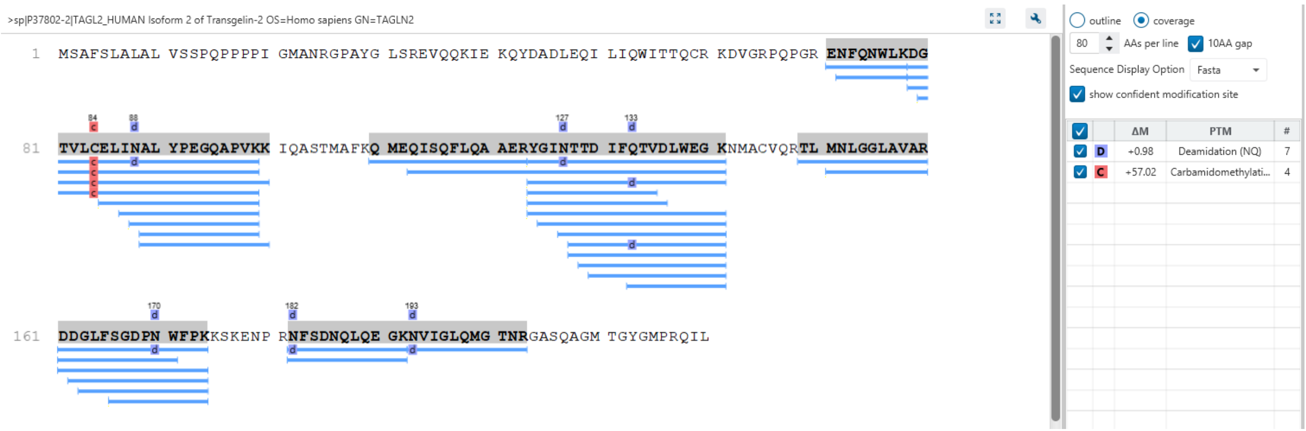

7.19.3.1 Protein Sequence Coverage

This area visualizes the coverage of the protein sequence.

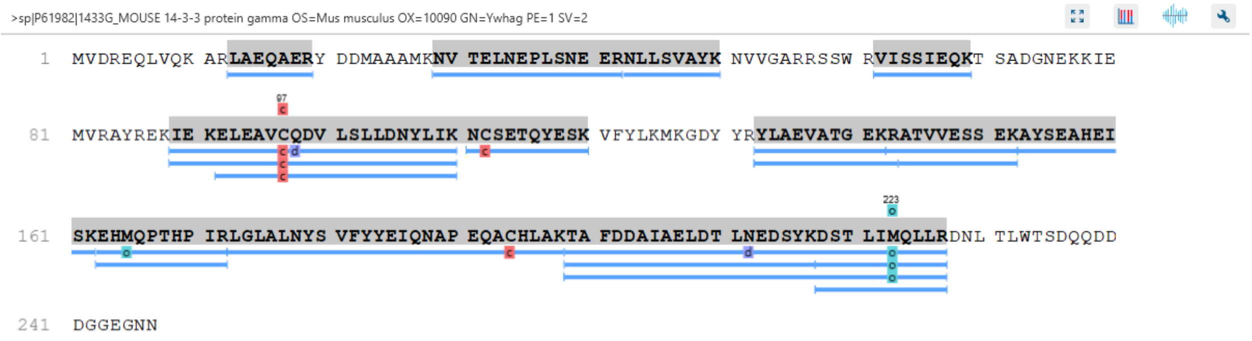

Regions in the protein sequence that are covered by supporting peptides are displayed in bold font with a grey background. Confident modifications identified in supporting peptides are displayed as icons above the protein sequence. Modifications are represented by colored icons with the first letter of its modification name. If a residue is modified by more than one modification in the same supporting peptide, "*" is used instead of a letter. The header information of the protein is shown on the top of the protein sequence.

The supporting peptides and matched de novo tags can be shown as colored bars under the protein sequence.

Placing the cursor over a bar shows a tooltip with detailed information of the peptide.

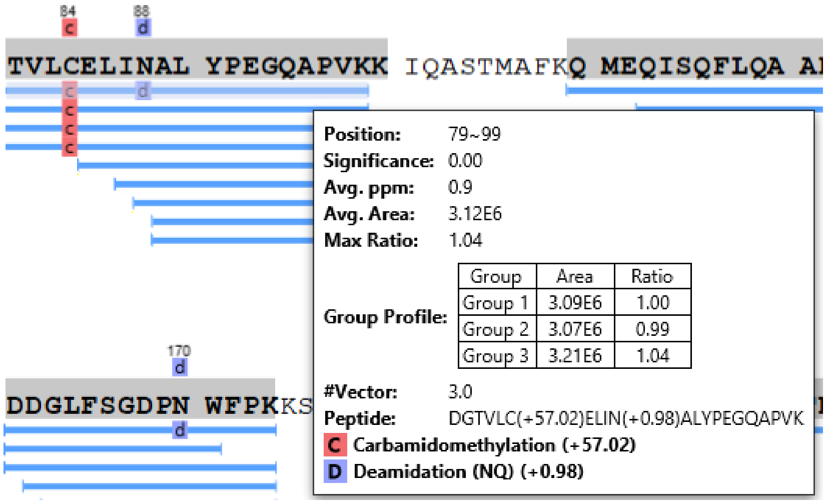

Left clicking on a bar shows the details from which the supporting peptide is identified.

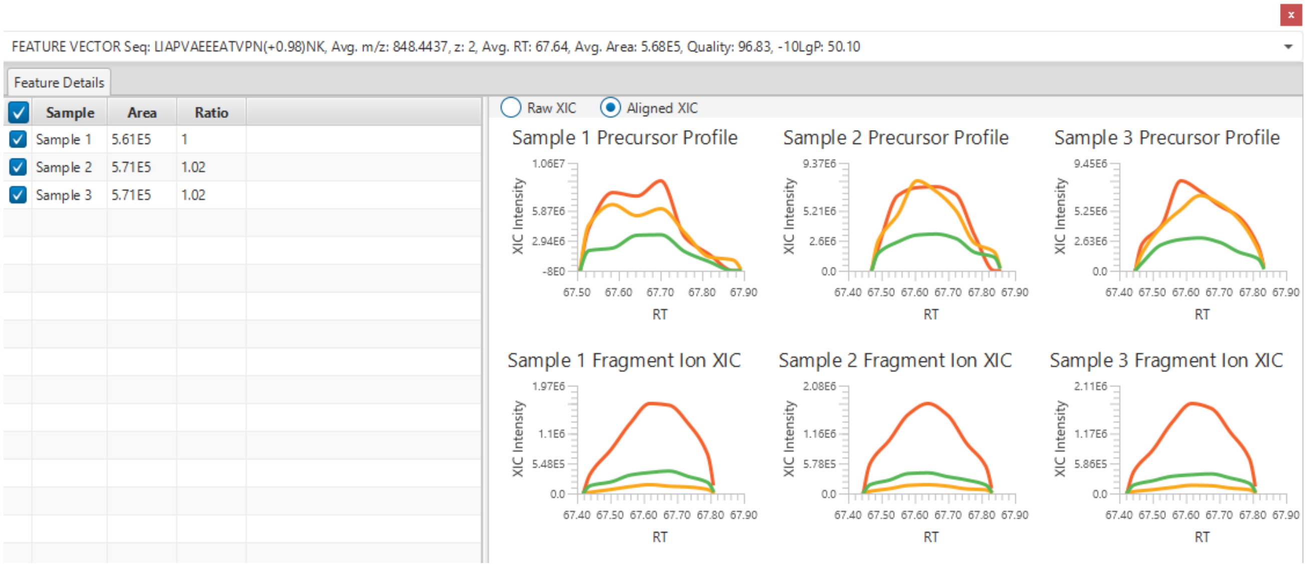

Right clicking on a peptide bar shows additional options. “Show in spectrum” has the same behaviour as left clicking on the peptide bar which is to open the annotated spectrum chart. “Show in table” has the function to jump to the supporting peptides table.

7.19.3.2 Coverage Control Panel

This panel controls what to display in the protein sequence coverage view.

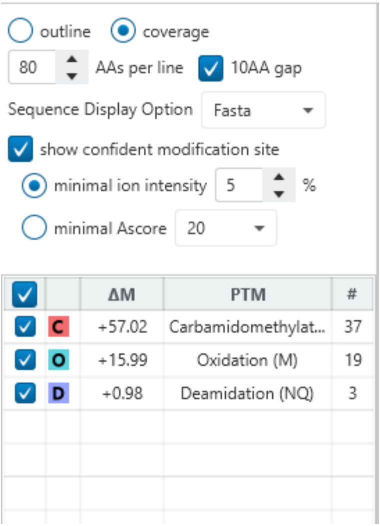

Sequence Display Mode: The protein sequence can be displayed in either "outline" or "coverage" mode.

Outline mode: In outline mode, the protein sequence is displayed without supporting peptides and matched de novo tags. However, the sequence coverage at a specific position can be examined by left-clicking a residue.

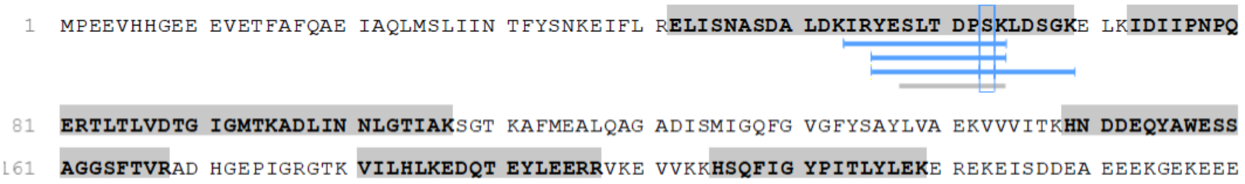

Coverage mode: In coverage mode, all the supporting peptides and matched de novo tags are shown.

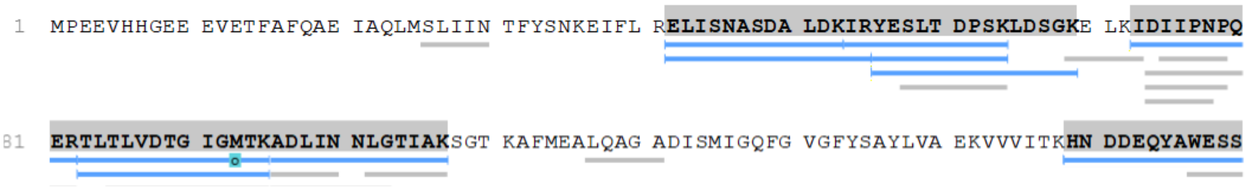

AAs per line and 10AA gap: Specify the number of amino acids per line and whether to show a gap for every 10 amino acids in the protein sequence.

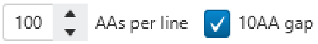

Sequence Display Option: Select whether to view the protein sequence as Fasta or a specific enzyme used in the project. When a particular enzyme is selected, the protein sequence will be highlighted to emphasize expected peptides generated by the selected enzyme's cleavage specificity.

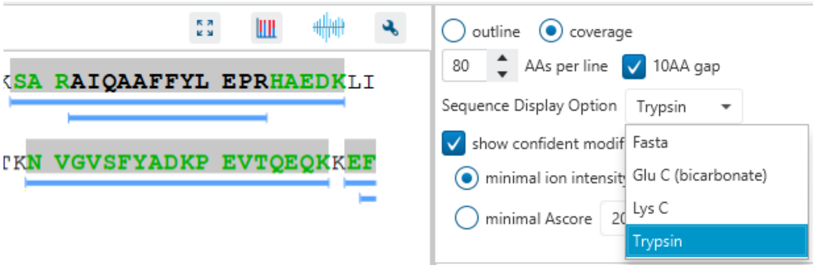

Modification minimal ion intensity / Ascore: A modification or mutation is considered confident if there are fragment ions supporting the modified residue with the minimal peak intensity above the specified threshold. Only confident modifications and mutations are displayed as icons above the protein sequence.

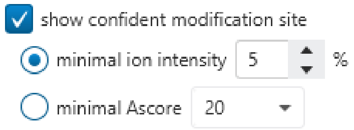

Modification table: The modification table shows the modifications identified in supporting peptides on the protein. For each modification, the number of supporting PSMs with this particular modification is shown. The checkbox on the left controls whether to indicate the modification in the protein coverage.

☑		ΔM	PTM	#
☑	C	+57.02	Carbamidomethylat...	63
☑	D	+0.98	Deamidation (NQ)	7
☑	O	+15.99	Oxidation (M)	5
☑	a	-2.02	2-amino-3-oxo-but...	3
☑	F	+27.99	Formylation	3
☑	A	+42.01	Acetylation (Protein...	2
☑	S	+21.98	Sodium adduct	2
☑	A	-0.98	Amidation	1

7.19.3.3 Proteins View - Protein Tools

The Proteins View toolbar is at the upper-right corner of the protein sequence coverage tab and contains helpful protein tools.

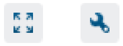

Full screen: When selected, the protein coverage window expands in full screen mode. Click to return from full screen mode.

Tool box: The tool box contains the following tools:

- Copy protein sequence. This copies the protein sequence of the current selected protein into the system clipboard.
- Save protein coverage as image. Different scale options are provided to increase the image size.
- Coverage statistics. This provides useful statistics based on the current selected protein.

7.19.3.4 Proteins View - Supporting Peptides

The supporting peptides assigned to the protein are shown in a separate tab beside the Protein Coverage view. The sequence can be clicked and a pop-up window will show up to display the feature details.

Coverage | **Peptides**

	Peptide	Used	Quality	Significance	Avg. ppm	Avg. Area	Sample Profile	Group Profile	Group 1 Area	Group 2 Area	Group 3 Area	Max Ratio	RT mean	#Vector	Start	End	PTM
1	GLTSVINQK	√	91.72	0.00	0.6	2.71E7			2.67E7	2.59E7	2.86E7	1.10	42.80	1	300	308	
2	IVVVTAGVR	√	95.92	0.00	0.7	4.87E6			4.81E6	4.73E6	5.07E6	1.07	47.25	1	92	100	
3	SVADLIESMLK	√	93.37	0.00	0.7	1.47E7			1.47E7	1.41E7	1.54E7	1.09	131.01	1	256	266	
4	ADELALVDVLEDK		98.74	0.00	0.7	1.18E5			1.19E5	1.16E5	1.17E5	1.03	109.55	1	46	58	
5	ADLIESMLK		94.32	0.00	0.6	1.54E6			1.54E6	1.57E6	1.52E6	1.03	90.44	1	258	266	
6	AIGLSVADLIESM(+15.99)LK		82.08	0.00	0.6	5.01E5			5.59E5	5.43E5	4.01E5	1.39	126.68	2	252	266	O
7	AIGLSVADLIESMLK		97.87	0.00	0.6	1.18E6			1.12E6	9.31E5	1.49E6	1.60	136.96	2	252	266	
8	ALVDVLEDK		97.27	0.00	0.5	3.47E6			3.41E6	3.46E6	3.53E6	1.04	68.26	1	50	58	
9	APVAEEEATVPNNK		92.97	0.00	1.0	7.33E5			7.38E5	7.85E5	6.75E5	1.16	36.19	1	10	23	
10	DELALVDVLEDK		101.65	0.00	1.1	1E5			1.01E5	9.97E4	1E5	1.01	119.72	1	47	58	
11	DLIESMLK		100.52	0.00	0.7	2.66E5			2.59E5	2.64E5	2.75E5	1.06	95.31	1	259	266	
12	ELALVDVLEDK		98.30	0.00	0.6	1.71E6			1.74E6	1.72E6	1.69E6	1.03	109.49	1	48	58	
13	ELNPEMGTDNDSENWK		100.71	0.00	0.5	7.95E4			7.94E4	7.79E4	8.12E4	1.04	64.14	1	214	229	
14	FIIPQIVK		106.07	0.00	0.6	2.43E5			2.42E5	2.42E5	2.46E5	1.02	91.64	1	120	127	
15	GEMMDLQ(+0.98)HGSLFLQTPK		90.71	0.00	0.6	1.38E5			1.37E5	1.4E5	1.37E5	1.03	91.14	2	61	77	D
16	GEMMDLQHGSLFLQTPK		98.56	0.00	1.1	1.05E5			1.03E5	1.07E5	1.07E5	1.04	91.17	2	61	77	
17	GLSVADLIESM(+15.99)LK		103.34	0.00	0.3	4.87E4			5.58E4	5.71E4	3.32E4	1.72	122.24	1	254	266	O
18	GLSVADLIESMLK		92.16	0.00	0.5	7.88E5			7.59E5	7.34E5	8.7E5	1.19	135.66	1	254	266	
19	GMYGIENEVF		101.34	0.00	0.6	6.78E4			6.58E4	6.87E4	6.9E4	1.05	107.68	1	280	289	
20	GMYGIENEVFL		97.63	0.00	0.4	9.81E4			1.09E5	8.46E4	1.01E5	1.29	124.77	1	280	290	

This table contains the following information of the quantifiable support peptides for the selected protein:

- **Peptide:** The amino acid sequence of the peptide, as determined in PEAKS Database or Spectral Library search. A modified residue is followed by a pair of parentheses enclosing the modification mass. All non-unique peptides and those not passing the filter will be excluded automatically.
- **Used:** Shows whether this peptide is used to calculate the protein abundance. The top three peptides with highest abundance are used to calculate the protein abundance.
- **Quality:** The feature vector quality score.
- **Significance:** The -10lg of a p-value represents the likelihood that the observed change between conditions is caused by random chance. The peptides are first separated into groups based on similar quality score. Significance is then calculated for each quality score group, or bin, using a two-tail T-test that assumes log normal distribution but does not assume equal variance. The -10log p-value is then calculated from this result and then displayed in the significance column.
- **Avg. ppm:** A peptide can have one or more feature vectors. Each feature vector has its own ppm calculated from the mass error of each feature. The average mass error of a peptide is the average of mass errors of all the feature vectors. This column displays the average mass error in ppm.
- **Avg. Area:** Each feature in the feature vector has its own area under the XIC curve. The average area of a feature vector is the average area of all the features associated with the peptide.
- **Sample Profile:** The peptide abundance among the samples is depicted as a heat map. Hold the cursor on a profile to view the samples, the peptide areas in samples, and the corresponding ratios with respect to the base sample.
- **Group Profile:** The peptide abundance among the groups is depicted as a heat map. Hold the cursor on a profile to view the groups, the peptide areas in groups, and the corresponding ratios with respect to the group which contains the base sample.
- **Group Area:** This column shows the peptide area for a group. It is calculated by summing up the areas of feature vectors within a group
- **Max Ratio:** This shows the maximum of the fold change values compared to the reference sample.
- **RT Mean:** Each feature in the feature vector has its own retention time center. This column displays the average retention time center of the feature vector.
- **#Vector:** This shows the number of quantifiable feature vectors of a peptide.
- **Start:** This shows the protein position of the first residue of the peptide.
- **End:** This shows the protein position of the last residue (inclusive) of the peptide.
- **PTM:** The types and the numbers of modifications present in the peptide shown using color-coded icons. Scroll over each icon to see the modification name and mass.

- **Feature Detail:** Double click on a supporting peptide to go to the peptide page where the eXtracted Ion Chromatogram (XIC) curves are displayed (as seen below).

7.20 DIA LFQ result - Peptides View

Quantified proteins are displayed in the Peptide View along with their detailed information.

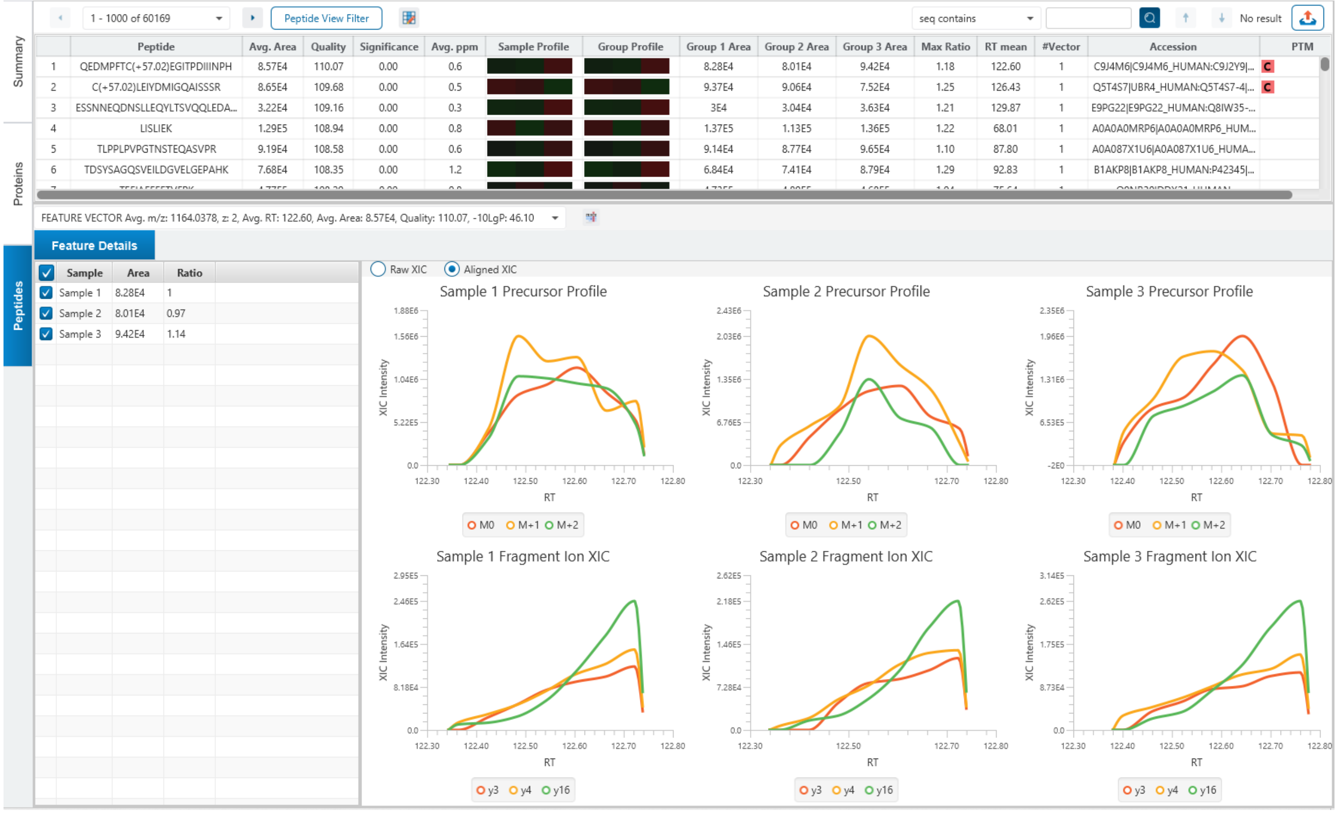

7.20.1 Peptides View - Peptide Table

The peptides are grouped together based on quantifiable feature vectors with the same sequence. When there are more than 1000 peptides, the table is split into multiple pages.

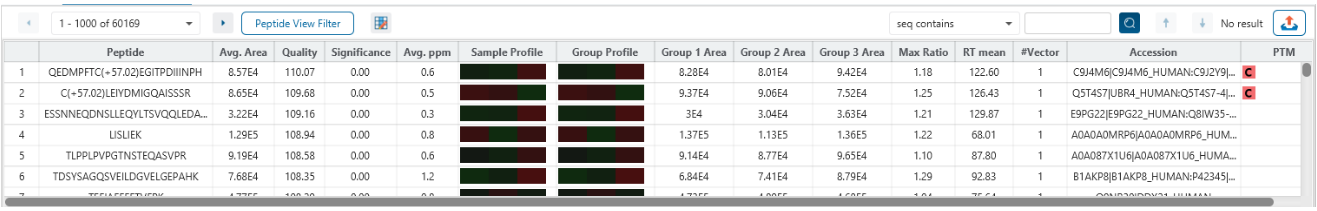

The view is similar to the support peptide table described in the previous section Section 7.19.3.4 Proteins View - Supporting Peptides. The Peptide table entries may be filtered by using the Peptide View Filters on top of the peptide table. To reiterate, the columns are:

- **Peptide:** The amino acid sequence of the peptide, as determined in PEAKS Database or Spectral Library search. A modified residue is followed by a pair of parentheses enclosing the modification mass. All non-unique peptides and those not passing the filter will be excluded automatically.
- **Avg. Area:** Each feature in the feature vector has its own area under the XIC curve. The average area of a feature vector is the average area of all the features associated with the peptide.
- **Quality:** The feature vector quality score.
- **Significance:** The -10lg of a p-value represents the likelihood that the observed change between conditions is caused by random chance. The peptides are first separated into groups based on similar quality score. Significance is then calculated for each quality score group, or bin, using a two-tail T-test that assumes log normal distribution but does not assume equal variance. The -10log p-value is then calculated from this result and then displayed in the significance column.
- **Avg. ppm:** A peptide can have one or more feature vectors. Each feature vector has its own ppm calculated from the mass error of each feature. The average mass error of a peptide is the average of mass errors of all the feature vectors. This column displays the average mass error in ppm.
- **Sample Profile:** The peptide abundance among the samples is depicted as a heat map. Hold the cursor on a profile to view the samples, the peptide areas in samples, and the corresponding ratios with respect to the base sample.
- **Group Profile:** The peptide abundance among the groups is depicted as a heat map. Hold the cursor on a profile to view the groups, the peptide areas in groups, and the corresponding ratios with respect to the group which contains the base sample.
- **Group Area:** This column shows the peptide area for a group. It is calculated by summing up the areas of feature vectors within a group
- **Max Ratio:** This shows the maximum of the fold change values compared to the reference sample.
- **RT Mean:** Each feature in the feature vector has its own retention time center. This column displays the average retention time center of the feature vector.
- **#Vector:** This shows the number of quantifiable feature vectors of a peptide.
- **Accession:** The proteins that the peptide was found in.
- **PTM:** The types and the numbers of modifications present in the peptide shown using color-coded icons. Scroll over each icon to see the modification name and mass.

To copy the sequence, right click on a row (or multiple rows) in the table and select “Copy selected peptide sequence(s)”. Another option is to mouse over any cell in the table and hitting Ctrl + C on your keyboard to copy the contents of those selected cells.

7.20.2 Peptide Table controls

The menu above the peptide table provides options to scroll through the table of peptides, filter the table, show optional columns and search for specific entries.

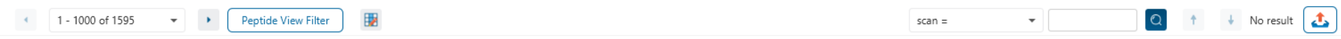

7.20.2.1 Peptide Table controls - Pagination

For easy viewing and to improve performance, the peptide table is limited to showing 1000 entries at once. Pagination is used to manage which set of 1000 entries will be viewed.

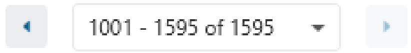

In the top left, there is a dropdown to select the rows for viewing. Click the left arrow or right arrow to select the previous/next 1000 rows. If the table is sorted or filtered, it may update the pagination set of rows that is currently in view.

7.20.2.2 Peptide Table controls - View Filter

Click on the 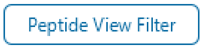

button to open the Peptide View Filter which is used to filter the Peptide table. The following are the filter options provided:

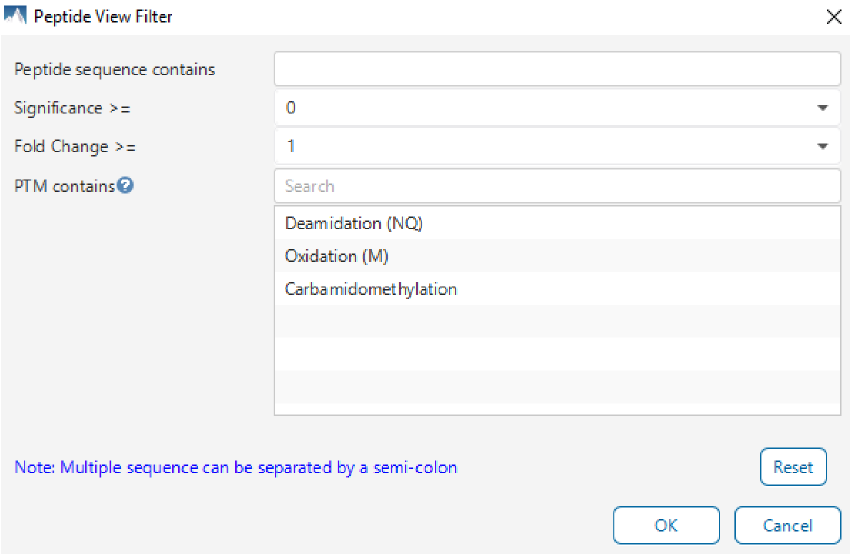

- **Peptide sequence contains:** Enter an amino acid sequence, only peptides that contain that sequence will be displayed in the peptide table. Adding PTMs in brackets in the sequence search is not handled, meaning only the sequence backbone is considered in the filter. Use the PTM contains for further filtering if necessary.
- **Significance >=:** Only peptides that pass this Significance threshold will remain in the table.
- **Fold Change >=:** Peptides with Max Ratio below this threshold will be removed from the table.
- **PTM contains:** Type the names of PTMs you would like to include. Peptides containing PTMs that match the text will be included. If multiple PTMs match the text they will all be included in the table.

Note: In all filters, multiple entries can be separated by a semi-colon.

Click on the Reset button to reset all filters to their default setting.

Click on OK to apply the filters to the Peptides table.

Click on Cancel to discard the changes in the View Filter.

7.20.2.3 Peptide Table controls - Optional Columns

Click on the 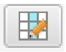 button to open the optional columns dropdown. The sample ratios and group ratios can be displayed in the peptide table for easy view and sorting. For projects with many samples, it might be useful to hide these optional columns and reduce the width of the peptide table.

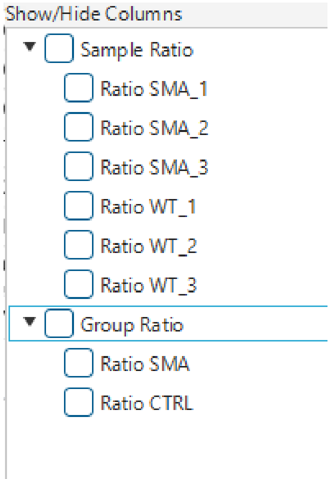

7.20.2.4 Peptide Table controls - Search function

On the top right of menu, there is a search function with 2 options. Search results can be navigated between using the Up or Down arrows.

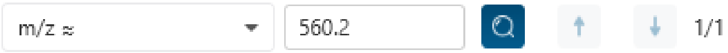

Options for searching include:

- **m/z ≈:** Search for the approximate m/z. The search will consider a range of m/z equal to the search value +/- 1 of the last significant digit. For example, searching for m/z ≈ 560.21 will find all m/z between [560.20, 560.22]
- **Seq contains:** Search for the sequence in the table. If specifying a sequence without PTMs, the results will include that sequence and any sequences with PTMs with the same backbone. If specifying a sequence with bracket and some mass value (like in the above image), then only sequence that have a substring with an exact match will be considered.
  Additionally, since the peptide table shows both I/L and they can not be distinguished, then searching for I in the sequence is equivalent to searching for L in the sequence. i.e. Searching for “LVLK” is the same as searching for “LVIK”, “IVLK”, and “IVIK”

7.20.3 Feature vector selection

Click on the Feature Vector dropdown to open a dropdown to display the feature vectors that correspond to the selected peptide. The peptide -10lgP score is the highest among the -10lgP scores of its feature vectors. The peptide quality score corresponds to the average of the quality scores of all feature vectors.

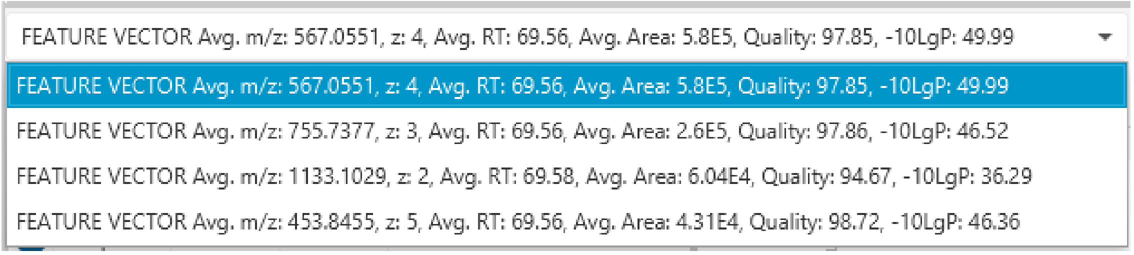

7.20.4 Protein jump button

Click on 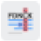 to jump to a protein that contains the currently selected PSM. If there are more than 1 protein, a dropdown is revealed which allows the user to select which protein to jump to. If a protein is filtered out due to a protein summary filter or summary page protein filter, then the option to jump will be grayed out.

7.20.5 Feature Details

The Feature Details contains the eXtracted Ion Chromatogram (XIC) chart. The eXtracted Ion Chromatogram (XIC) chart displays the shape of the selected peptide feature vector over the retention time range associated with the identification. The table on the left displays both the area and the ratio of the features in the highest average abundance feature vector. This table also works as a control to select the features that will be used to draw the XIC plot. By default, the first sample in each group is selected.

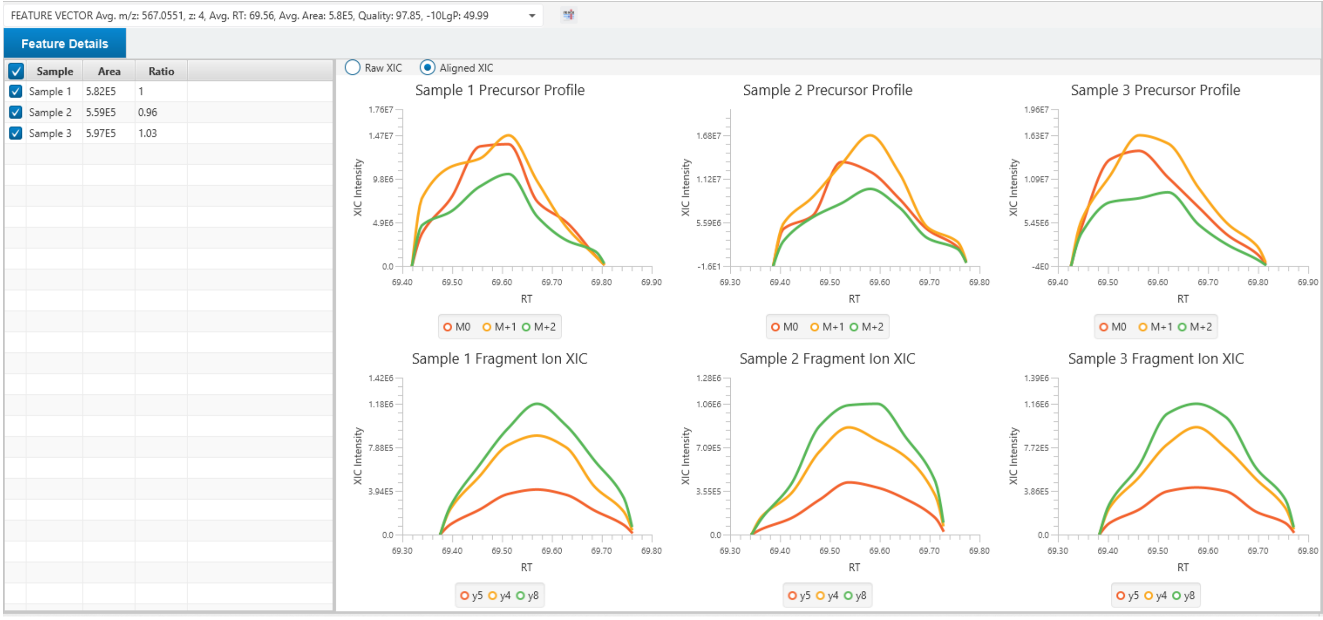

Click on the “Raw XIC” and “Aligned XIC” to switch between raw and aligned RT view.

Right click on the chart area to export the XIC charts.

7.21 DIA LFQ Exporting

In Project View, double-click on 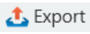 to open the Export node.

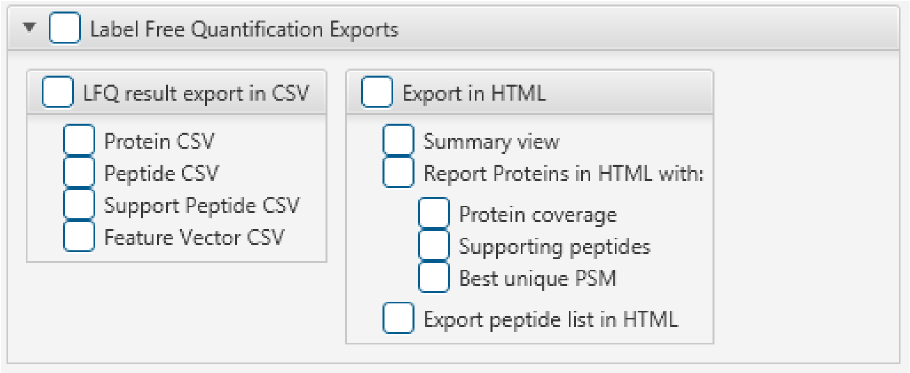

7.21.1 LFQ result - Exporting - comma separated values (CSV) format

There are several options for exporting the PEAKS Label Free Quantification results in CSV format:

- **Proteins CSV:** The list of protein quantifications, filtered by the protein filters in the Summary view and will be saved to proteins.csv.
- **Peptide CSV:** A complete list of all quantified peptides that pass the peptide feature filters set on the "Summary" page and their associated details.
- **Support peptides CSV:** These are the supporting peptides of the quantified proteins and their associated details.
- **Feature Vector CSV:** All predicted peptide features and their associated quantification information. Both identified and unidentified peptide features are included. To include unidentified peptide features, set the Peptide Id Count equal to 0 in the Peptide feature filter.

7.21.2 LFQ result - Exporting - HTML format

Export in HTML: This will generate single or multiple HTML reports in the specified location.

The following exporting options are available:

- **Summary view:** The “Summary” view page will be saved as a summary.html file in HTML format in the specified location.
- **Protein coverage:** The coverage pane will be saved for each protein.
- **Supporting peptides:** A list of supporting peptides will be saved for each protein.
- **Best unique PSM:** The best unique PSM will be saved for each protein.
- **Export peptide list in html:** creates a separate HTML-containing peptide identifications.

An individual protein will have its own HTML output file, where the corresponding protein coverage, the supporting peptides, and the best unique PSM are gathered.

8. Label Free Quantification (LFQ)

Label free quantification can be used as a quick profiling tool to examine the relative abundance of proteins in large proteomic datasets. In PEAKS Studio this quantification method is based on the relative abundance of peptide features detected in multiple samples. PEAKS Studio performs feature detection separately on each sample, and uses an EM (expectation-maximization) algorithm to detects and deconvolute overlapped features. Importantly, features of the same peptide from different samples are reliably aligned together (i.e. matched between runs) using a high performance retention time alignment algorithm to improve overall protein quantification rates.

Note: For details of the retention alignment algorithm, refer to the paper *" A Combinatorial Approach to the Peptide Feature Matching Problem for Label-Free Quantification", Bioinformatics, 2013, 10.1093 .*

8.1 Label Free Quantification Analysis Workflow and Settings

Label Free Quantification requires at least 2 samples in the analysis. It is recommended to only run LFQ with 1 data file per sample, but if you choose to have fractionated samples, ensure each sample has the same number of fractions.

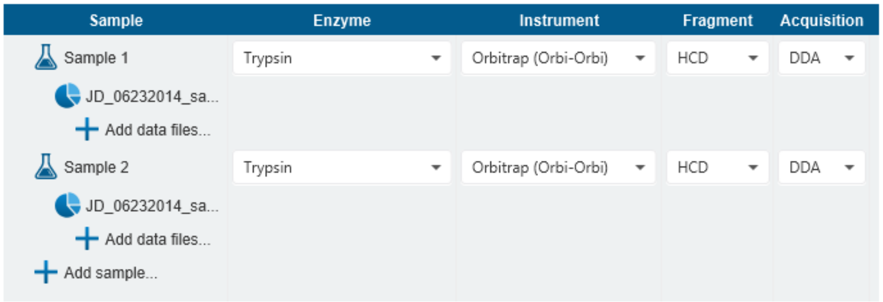

Select the PEAKS Q workflow in the Workflow Selection step

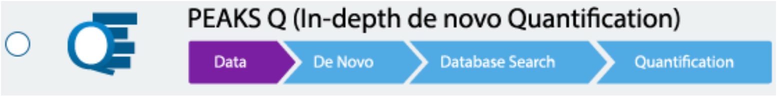

The following steps are the same as in the PEAKS DB workflow (as in section 6.1 Database Search Workflow and Parameter Settings) and is reiterated below.

Click on Data Refine to proceed through the workflow.

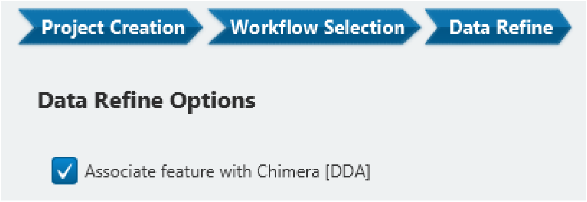

After deciding the Data Refine option, proceed to the next step to see the DB Search parameters.

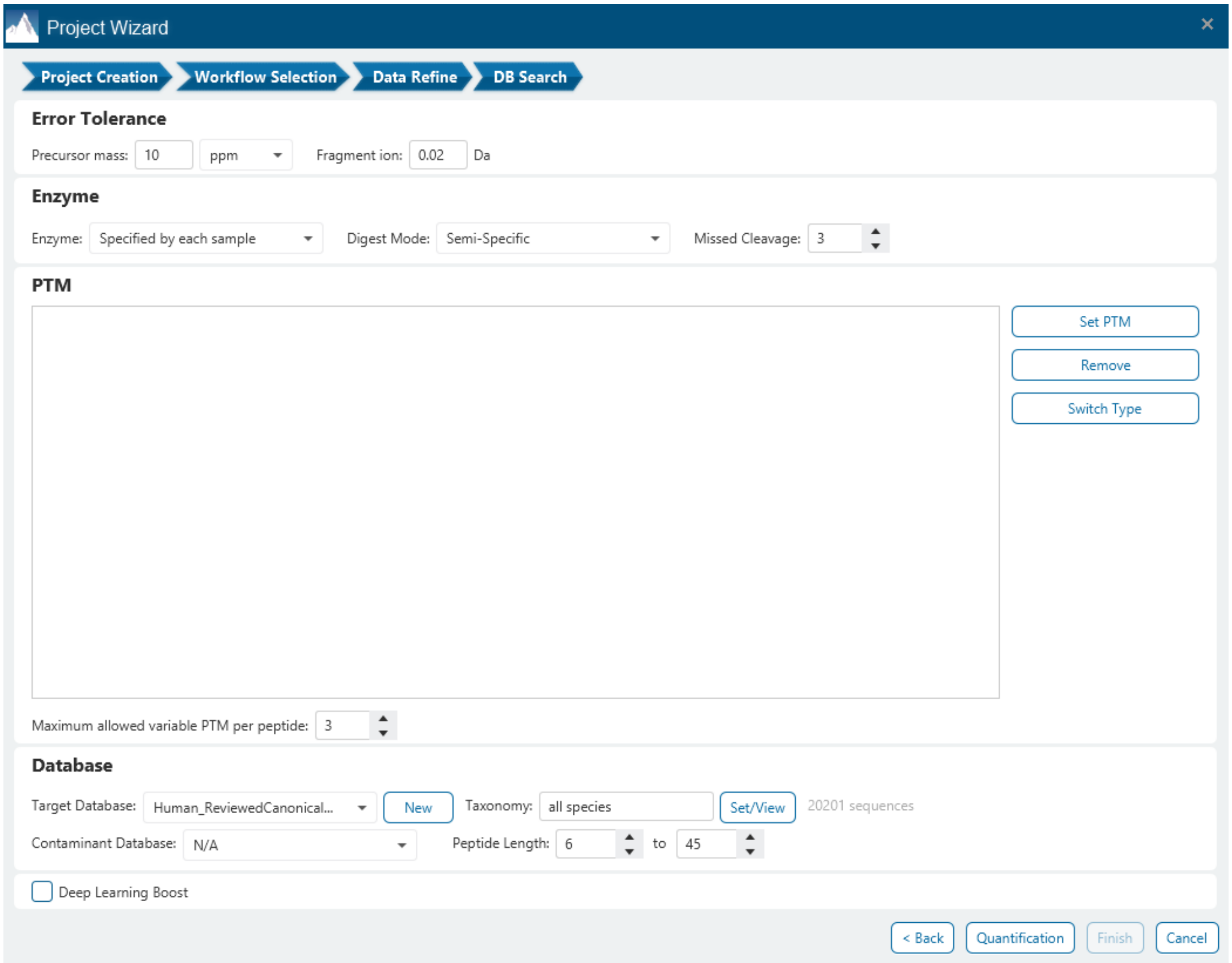

The following parameter settings are provided:

Precursor Mass Error Tolerance: The maximum mass difference between the peptide and the monoisotopic mass of the precursor ion.

Precursor Mass Tolerance unit: PEAKS generates peptides within the precursor mass error tolerance. Precursor mass error tolerance can be specified in either Daltons or ppm.

Fragment Mass Error Tolerance: PEAKS uses this value when scoring peptide sequences. PEAKS Studio considers a fragment ion to be matched if the calculated m/z is within the specified error tolerance. Units are Daltons.

Both precursor and fragment error tolerance parameters need to be set consistently with the mass accuracy of the instrument.

Enzyme Settings: PEAKS Studio digests the protein database *in silico* to generate peptide candidates. It is necessary to specify the enzyme for protein digestion from the Enzyme drop-down menu.

Note: When the selected dataset is digested with different enzymes, "Specified by each sample" allows samples to be analyzed separately using their respective enzymes specified during project creation.

Note: "None" is a special enzyme allowing non-specific cleavage at both ends of the peptide. It is the recommended option when no digestion enzyme was used or when the digestion enzyme exhibits a high degree of non-specificity.

Missed Cleavages: This specifies the number of missed cleavage sites allowed in a peptide.

Digest Mode: This specifies the type of in silico cleavages allowed at a peptide termini based on the method used for protein digestion. If "Specific" is selected, the specificity of the selected enzyme is strictly enforced at both termini of a peptide. "Semispecific" allows one terminus to disobey the enzyme specificity rules.

Note: If the enzyme is specified as "None", then no matter which mode is selected for "Digest Mode", the "None" enzyme digest rule will be applied.

PTM Settings: Click “Set PTM” to open the **PTM Options** dialog and specify the fixed PTMs and a few common variable PTMs expected in the experiment. Highlight PTMs and click on the button to place them as either Fixed or Variable PTM. From there, highlighted PTMs can be removed by clicking the “Remove” button. Clicking “Remove All” will reset the PTM options. Highlighting and clicking “Switch Type” will move the Fixed PTM in into the Variable PTM list and vice-verse. Click on “New” to create a new custom PTM. While a PTM is selected, click on “View” to see more details.

Note: Multiple PTMs can be highlighted by holding down the CTRL key or SHIFT key on the keyboard.

Note: For a specific residue, only one fixed modification is allowed.

Once the PTMs are selected, the maximum number of variable PTMs per peptide can be specified and a value of less than 4 is recommended.

Note: The use of variable modifications increases the size of the computational search space for the de novo sequencing algorithm. It is recommended not to select too many variable modifications in PEAKS de novo. See PEAKS PTM for alternate search options.

Target Database: Select a protein sequence database for the search. Select one from the list of databases that have been configured in PEAKS and set the taxonomy by clicking on Set/View, if applicable. To configure a new database, click on New next to the Target Database dropdown to open the Database Configuration dialog.

Contaminant Database: To search for contaminants, select a contaminant database from the list of databases that have been configured in PEAKS. Keep the selection as N/A if no contaminant database is required for the search.

Peptide Length: The length for a peptide can be set when creating an analysis, where the default peptide length range is between 6 and 45.

Deep Learning Boost: Check this option to use PEAKS enhanced deeplearning algorithm to better identify PSMs in the DB Search process.

Click on Quantification to proceed to the next step and select the Label Free option.

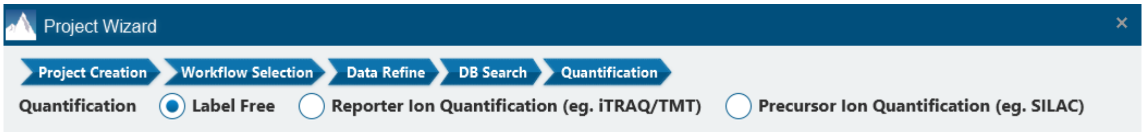

The following parameters are available:

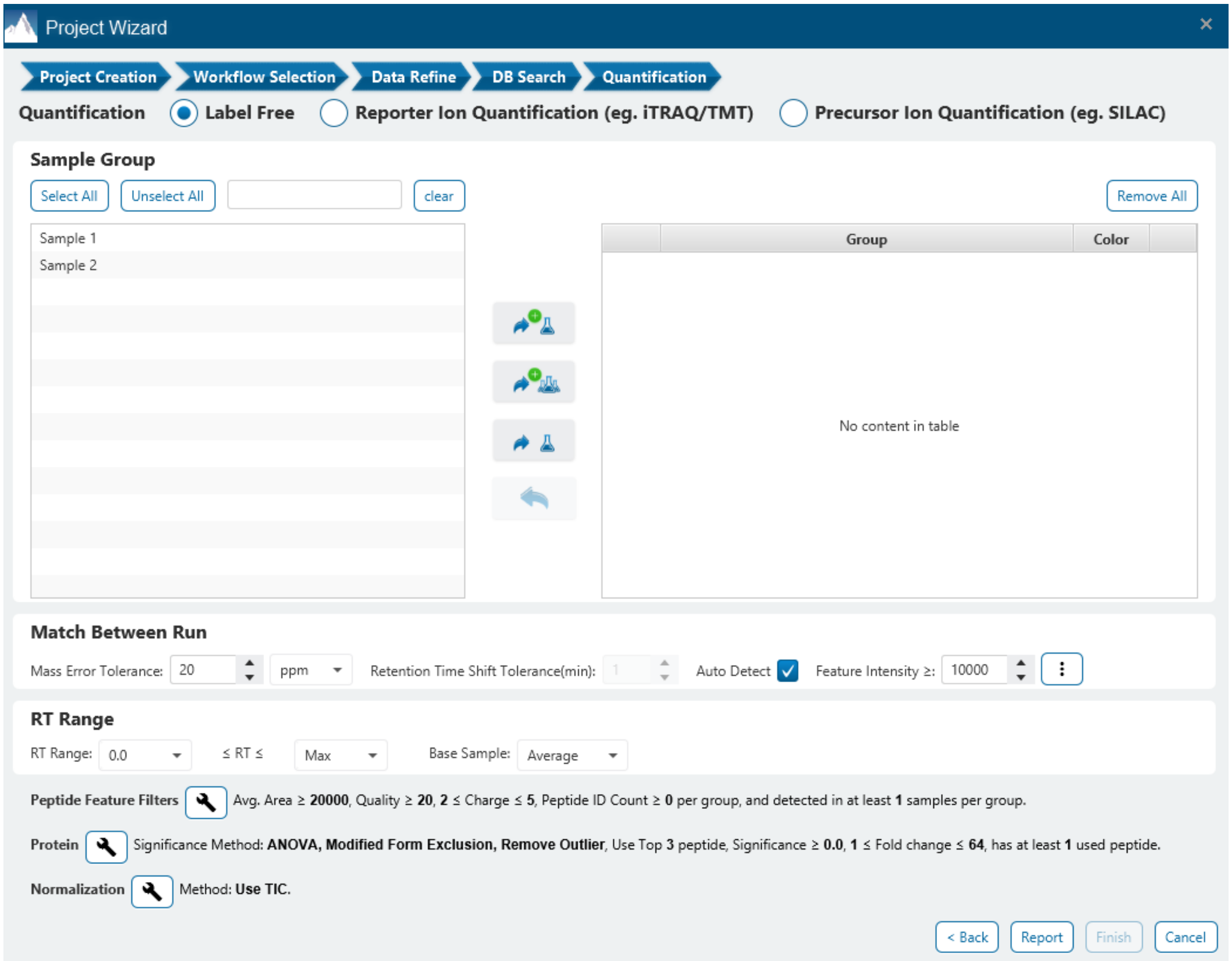

Sample Group: LFQ requires sample grouping. All available samples are listed in the left window. After selecting a sample, it can be added to a new group by clicking the 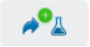 button or to an existing group by clicking the 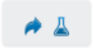 button and selecting that option from the drop-down menu. If you select multiple files, you can add each one individually to create multiple new groups by clicking the 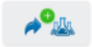 button. Samples and Groups can be removed by clicking the 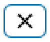 button next to each element. The names and colours for each group can be edited by clicking on the coloured square dropdown, respectively.

Mass Error Tolerance: The mass shift of a precursor ion between different runs. This can be set similar to the precursor mass error tolerance used for database search.

Retention Time Shift Tolerance: The retention time shift tolerance is the maximum elution time difference that is considered for the quantification of an identified peptide after RT alignment.

Auto Detect: Let PEAKS Studio detect the optimal Retention Time Shift Tolerance for your data based on the observed trend.

Ion Mobility Tolerance (1/k0) (if applicable): The maximum 1/k0 differential for features to transfer IDs.

Note: This parameter is only available when the analysis contains timsTOF data.

Feature intensity: Set a value for the minimum intensity a peptide feature must exhibit.

RT Range: Feature vectors within the selected retention time range will be included in the results while everything else will be removed.

Base Sample: The base sample will act as the reference. The peptide and protein abundance corresponding to this reference label will be the denominator in all of the abundance ratio calculations.

Click on the tool icon next to Peptide Feature Filters to open the Feature filter dialog.

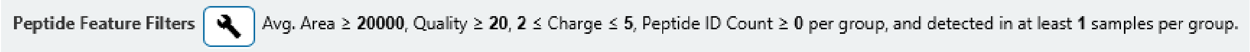

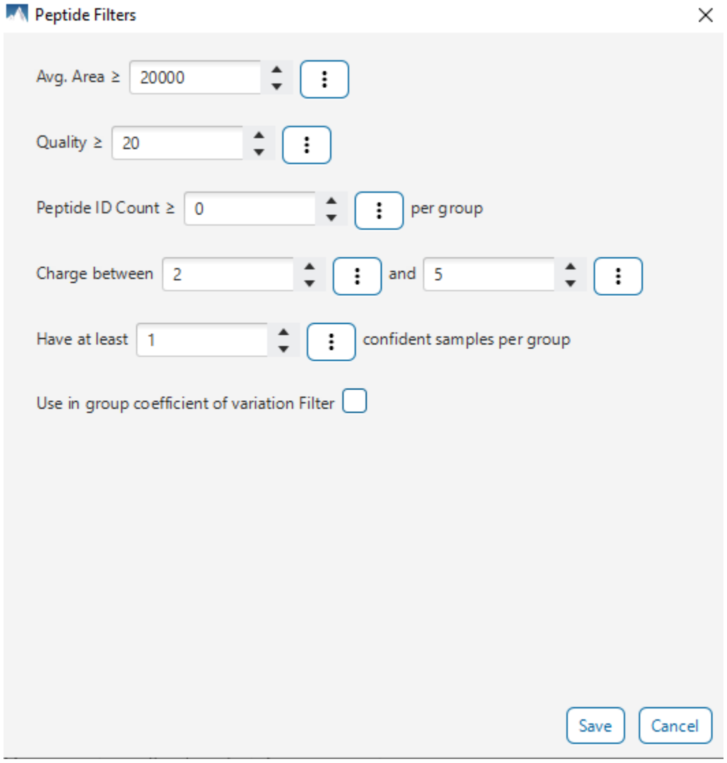

Avg. Area: The minimum average area of all peptide features associated with the peptide. Features with higher abundance have been shown to be more reproducible across replicates. Only peptide features with an average area above this threshold will be used for quantification.

Quality: The quality score for the peptide feature. Factors that affect the quality score include m/z difference, RT difference, XIC shape similarity, and the feature intensities.

Peptide ID Count: This filter sets the minimum limit for how many times a peptide must be identified within a group to be included in the LFQ results.

Charge between: Only peptides with feature vectors that all fall within this range will be used in protein quantification.

Have at least ___ confident samples per group: This filter sets the minimum number of sample(s) per group that the peptide feature has an abundance value. A peptide is more quantifiable when it is detected in more samples.

Use in group coefficient of variation filter: For each feature vector (set of peptide features associated with a peptide), at least one group must have a coefficient of variation (CV) less than the CV threshold. The CV threshold is automatically selected by the software based on the current data set.

Click on the tool icon next to Protein to open the Protein filter dialog

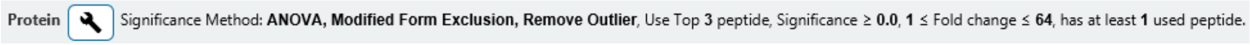

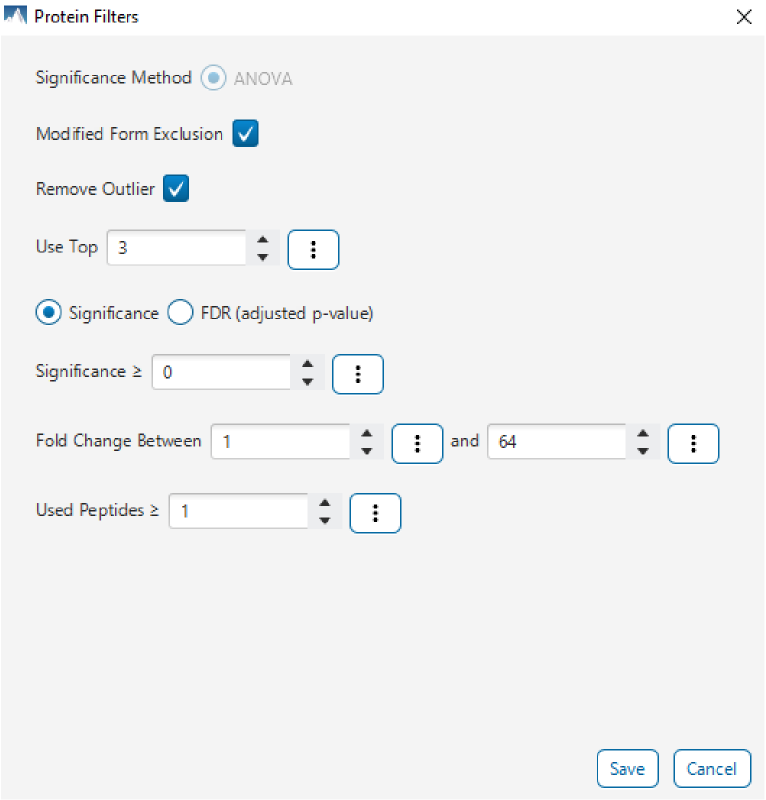

Significance Method: Protein abundance is used for the significance testing calculation. When there are replicates in each group, ANOVA is suggested to be used for significance testing. For more details on ANOVA, refer to the following paper: *"On the comparison of several mean values: an alternative approach ", Biometrika, 1951, 38(3/4): 330-336*

Modified Form Exclusion: The expression level of modified peptides might be different than the overall expression level of a protein. In such cases, including modified peptides for protein ratio calculation might lead to incorrect results. By checking this box, if a peptide has modified and unmodified forms, both will not be used as candidates for protein ratio calculation, if a peptide only has one modified form, it is still a candidate, but if a peptide has more than one modified form, all forms will not be used as candidates.

Remove Outliers: This filter removes supporting peptides that have a different variation trend compared to others.

Use Top # peptides: This is the number of quantifiable supporting peptide that will be used to calculate the protein profile. Three is the default setting.

Significance: Only protein groups with a significance above this threshold will be listed in the "Protein" view. The significance score is calculated as the -10log of the significance testing p-value (e.g. Significance score of 20 is equivalent to a p- value of 0.05). PEAKS provides the significance testing method: ANOVA.

FDR: Adopt the Benjamin-Hochberg method to adjust the p-value to the false discovery rate for all protein groups that have already passed the other filters. Only protein groups with significance scores passing the calculated FDR will be listed in the "Protein" view. Either this or "Significance" can be selected to set a significance threshold.

Fold Change: Only protein groups below the lower threshold or above the upper threshold value will be listed in the "Protein" view.

Used Peptides: Only protein groups that were quantified with this number of peptides (or more) will be listed in the "Protein" view.

Click on the tool icon next to Normalization to open the Normalization dialog.

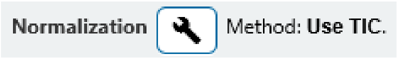

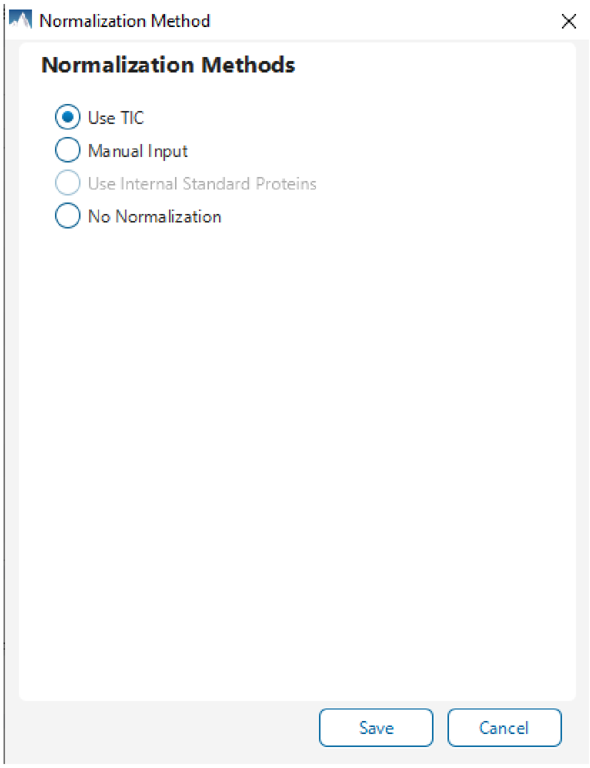

Use TIC: By default, PEAKS Studio uses the total ion current (TIC) of the samples to calculate the normalization factors. Normalized abundance for each sample is calculated from the raw abundance divided by the normalization factor for that sample.

Manual input: This option performs the same default calculation as auto normalization ("Use TIC") but also allows for the manual change of each sample's expected ratios. This option should be used if the expected ratio is not 1:1 for each sample as the software attempts to normalize to a 1:1 ratio.

Use internal standard proteins: This option displays a list of identified proteins (when a database search has been completed) that can be selected to create a normalization ratio. A search bar is included to enable the quick location of select proteins.

No Normalization: All samples are automatically assigned Factors of 1.0.

Click on "Report" to proceed to the final step. Report filters can be set here which will filter the final result.

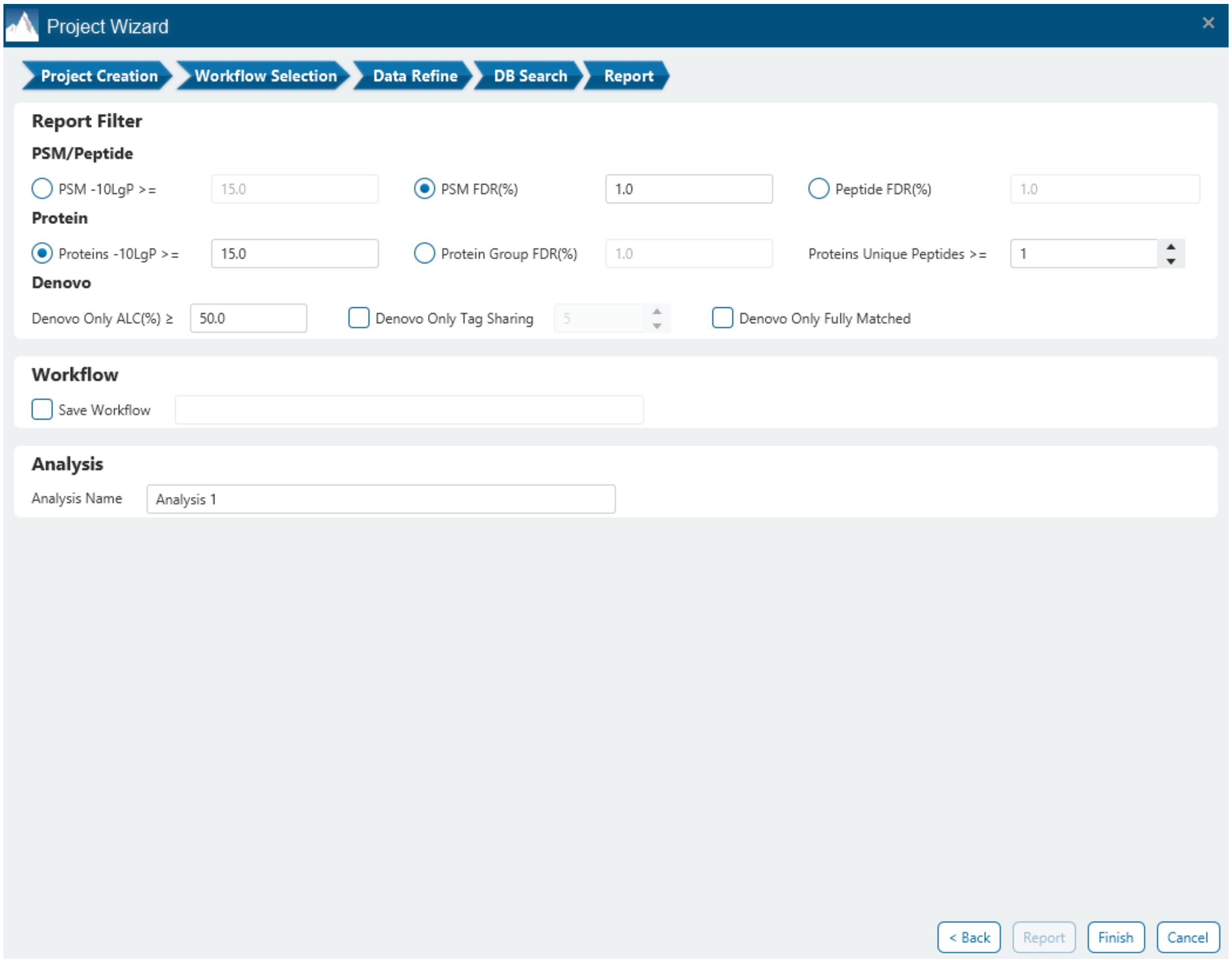

These filters are provided:

PSM/Peptide: this section is a mandatory section to filter out some results under the user-defined filter. Any changes to the filter will create a new analysis with old parameters and update the results and statistics.

PSM -10lgP: The PEAKS peptide score (-10lgP) is calculated for every peptide-spectrum match (PSM) reported by PEAKS DB, PEAKS PTM, and SPIDER. The score is derived from the p-value that indicates the statistical significance of the peptide-spectrum match. A peptide may be matched to many spectra, resulting in multiple PSMs. In that case, the peptide's score is calculated as the maximum among all PSM scores. For details of the scoring algorithm,

please refer to the publication, *"PEAKS DB: De Novo sequencing assisted database search for sensitive and accurate peptide identification ", Mol Cell Proteomics, 2011 Dec 20.* A minimum requirement can be set and all identifications must pass this filter.

PSM FDR (%): The PSM filter can also be set to use the false discovery rate. FDR is estimated using a "decoy-fusion" approach.

Peptide FDR (%): The Peptides can also be set to use the false discovery rate. Peptides are scored based on their top PSM and using a “decoy-fusion” approach, the result is reported at that FDR.

Note: Decoy fusion is an enhanced target-decoy method for result validation with FDR. Decoy fusion appends a decoy sequence to each protein as the "negative control" for the search. See BSI's web tutorial (http://www.bioinfor.com/fdr-tutorial/) for more details.

De Novo ALC (%): This filter controls the minimum ALC score required for display in the de novo only result. A default of 50% is set.

Denovo Only Tag Sharing: The number set here corresponds to the length of de novo only tags to be shown in the result Protein coverage. For de novo only peptides that are not fully matched, they must share a tag length with of minimum of this parameter with the protein residue to be displayed.

Denovo Only Fully Matched: Checking this option will show the de novo only peptides in the Protein coverage regardless of what tag sharing parameter is set if the entire de novo only sequence matches the protein residue.

In the Report step, there also is the option to specify an Analysis Name (which can be later renamed) and/or decide to save the Workflow to quickly re-use in future analyses.

8.2 Understanding LFQ Results

Once completed, a label free quantification result node will be added to the project tree. Double-click on this 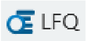

node to open the LFQ result that contains multiple tabs as described below:

Summary: The outline of the PEAKS LFQ search results with statistics. This is the place to examine the overall performance of the experiment and adjust filters.

Proteins: The quantified proteins with a list of supporting peptide features for each protein.

Peptides: The quantified peptides listed in a table. Extracted ion chromatogram (XIC), sample features and retention time alignment also displayed for each peptide.

8.3 LFQ - Summary View

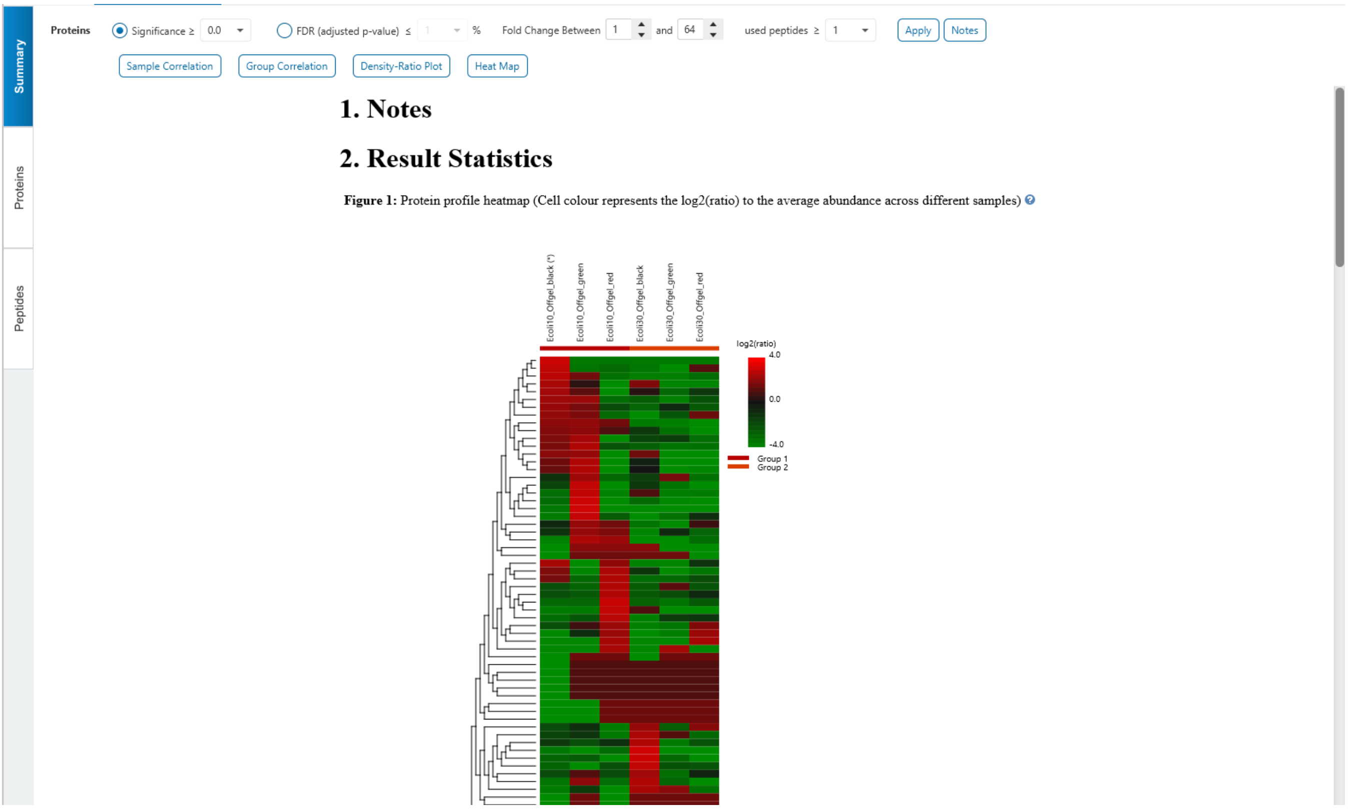

8.3.1 Summary View - Protein Filters

Filter settings used in the screenshot above means that no protein filter is applied, thus giving all quantifiable protein.

Alternately, parameters can be adjusted for additional analysis by updating the protein filters above.

Significance: Only protein groups with a significance above this threshold will be listed in the **Protein View**. The significance score is calculated as the -10log10 of the significance testing p-value. PEAKS provides ANOVA significance testing methods A significance score threshold of 20 is recommended, which equals to a significance testing p value of 0.01. Either this or “FDR (adjusted p-value)” can be selected to set a significance threshold.

Fold Change: Only proteins at or above this fold change threshold will be listed in the Protein View.

Used Peptides: Only proteins with at least this many number of used peptides will be listed in the Protein View.

Click on Apply to apply these filters changes to the result. This will update charts on the Summary View and will update the Protein table accordingly.

8.3.2 Summary View - Notes

Click on Notes to open the Notes dialog and save information about the result and saved in section 1. Notes on the Summary page.

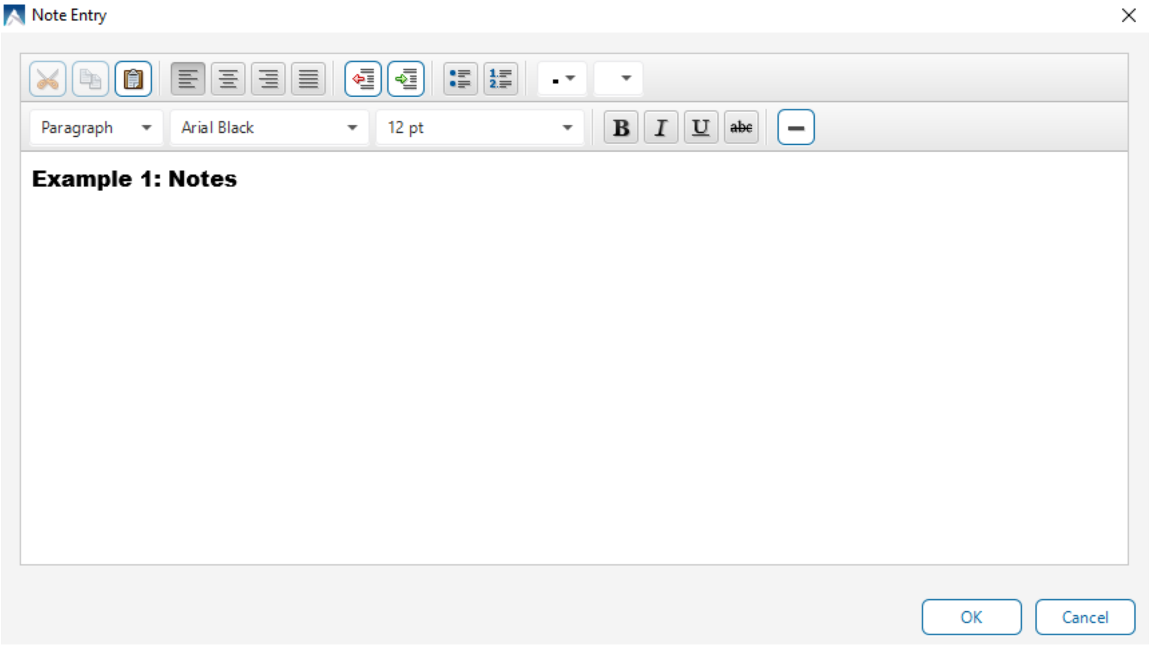
Note Entry
Paragraph
Arial Black
12 pt
Example 1: Notes
OK
Cancel

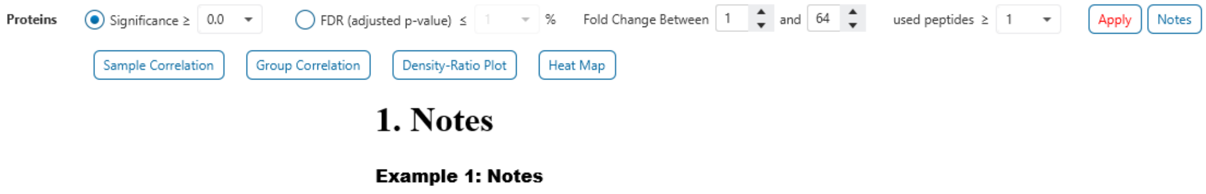
Proteins
Significance ≥ 0.0
FDR (adjusted p-value) ≤ 1 %
Fold Change Between 1 and 64
used peptides ≥ 1
Apply
Notes
Sample Correlation
Group Correlation
Density-Ratio Plot
Heat Map
1. Notes
Example 1: Notes

8.3.3 Summary View - Heat Map

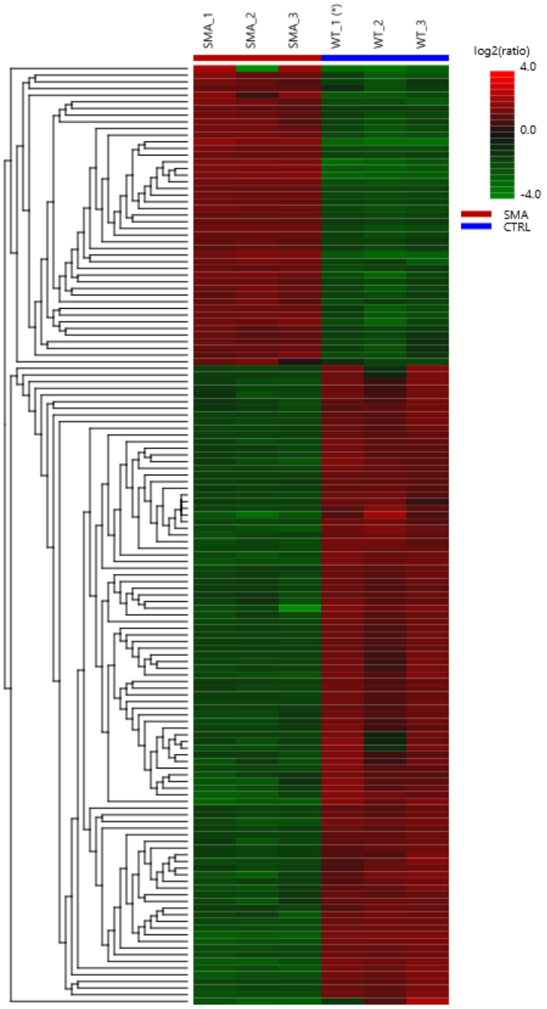

The heat map displays the protein groups that passed the filters. The relative protein abundance is represented as a heat map of the representative proteins of each protein group. These representative proteins are clustered if they exhibit a similar expression trend across the samples. The hierarchical clustering is generated using a neighbour-joining algorithm with a Euclidean distance similarity measurement of the log2 ratios of the abundance of each sample relative to the average abundance. Similarly, the conditions in different samples are clustered if they exhibit a similar expression trend across the protein groups. An interactive protein profile heat map is opened in a new window when the Heat Map button is clicked options.

Mouse over the dendogram in a section of the heatmap and click on it to open the Variation Trend chart.

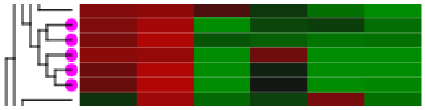

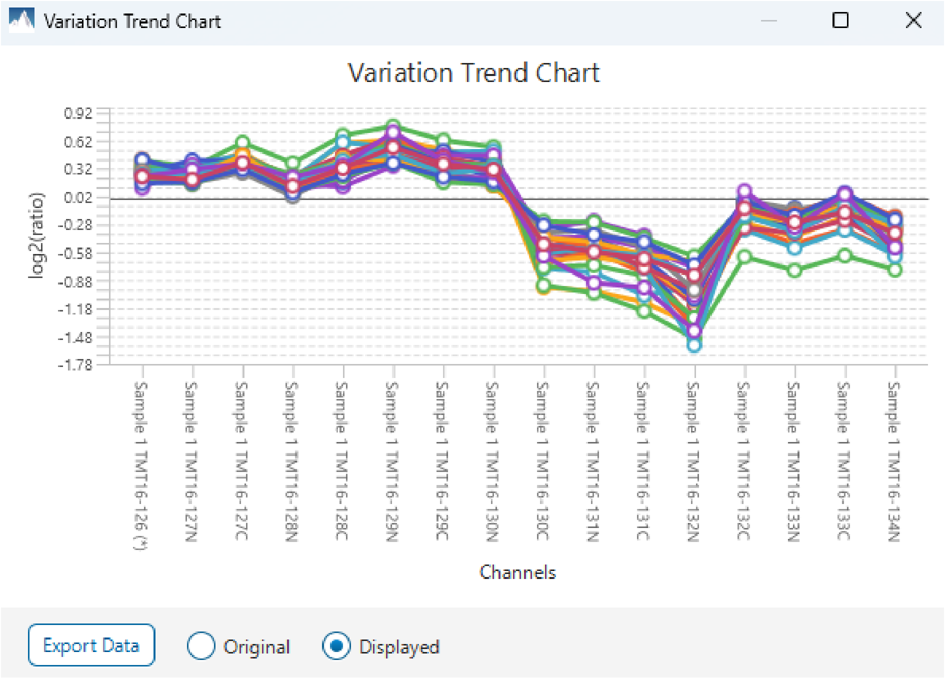

The Variation Trend chart displays the expression level of the selected proteins in the samples.

Mouse over points on the variation trend chart to see a tooltip of the protein, sample, and log2ratio.

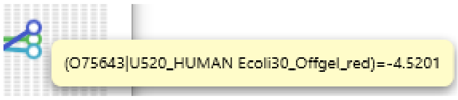

Click on the Export Data button to export the variation trend chart data in a text file format. When Displayed is selected, the log2 ratios will be in the export. When Original is selected, the original ratio values will be in the export instead.

8.3.4 Summary View - Density-Ratio Plot

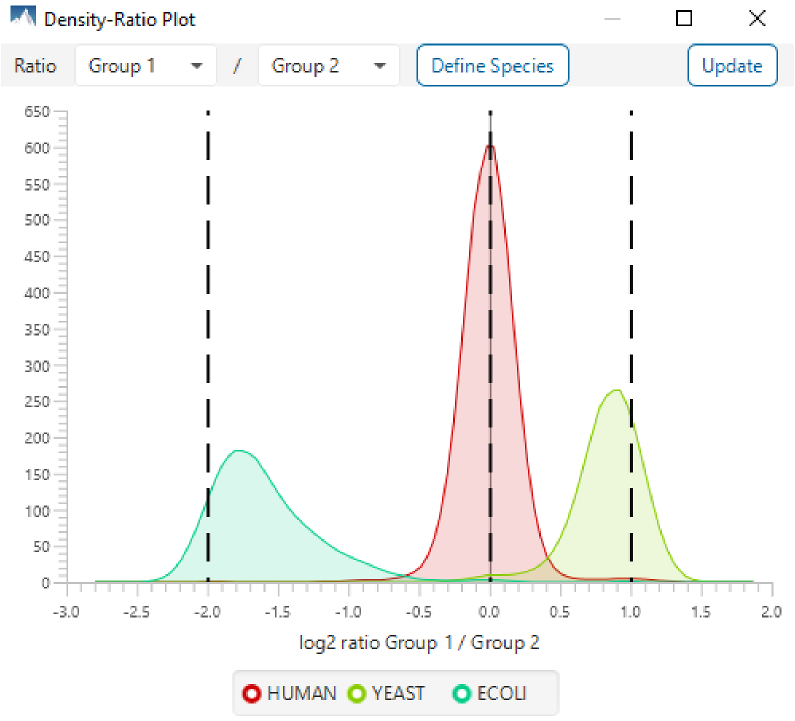

To view the group ratio distribution of the quantified proteins, click the Density-Ratio button. By default, all proteins will be shown. Click Define Species to group the different species together.

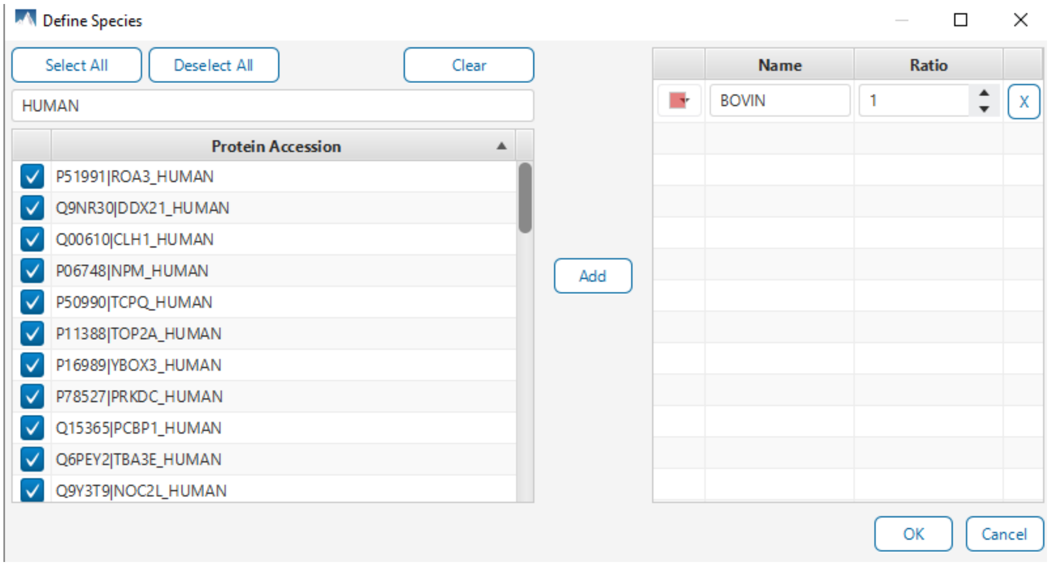

The proteins can be searched from the list by typing into the search box, marked and then added as a species group. The expected ratio of the species in the "Ratio" column can be edited to match with the experiment design. Once all the species are added and organized, click Ok to finish. In the plot window, click Update to apply the changes.

8.3.5 Summary View - Sample Correlation plot

To view the abundance correlation plot for the detected features and proteins, click the Sample Correlation button from the Summary View title bar. A Correlation Plot Options window will pop-up to select which samples to include.

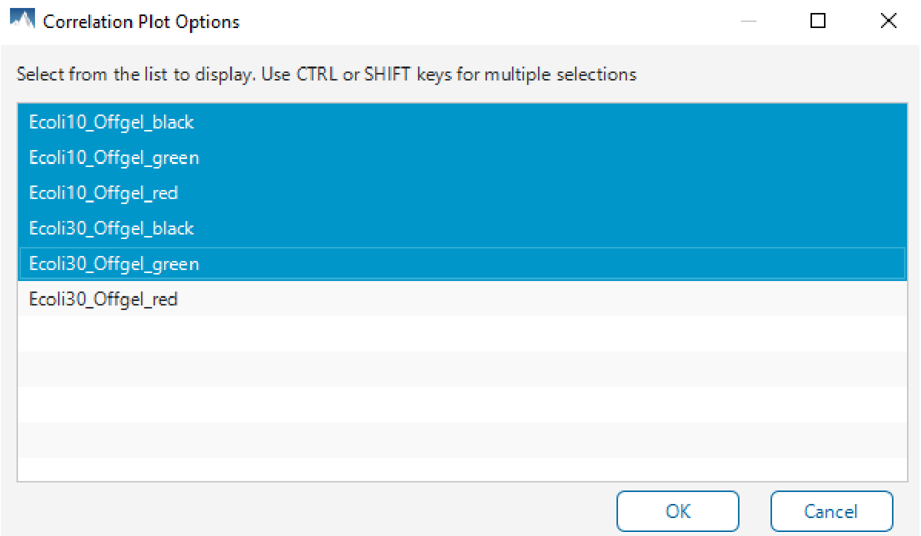

By default, the first 5 samples are selected. At most 5 samples can be used to generate the correlation plot, and at least 2 samples must be selected.

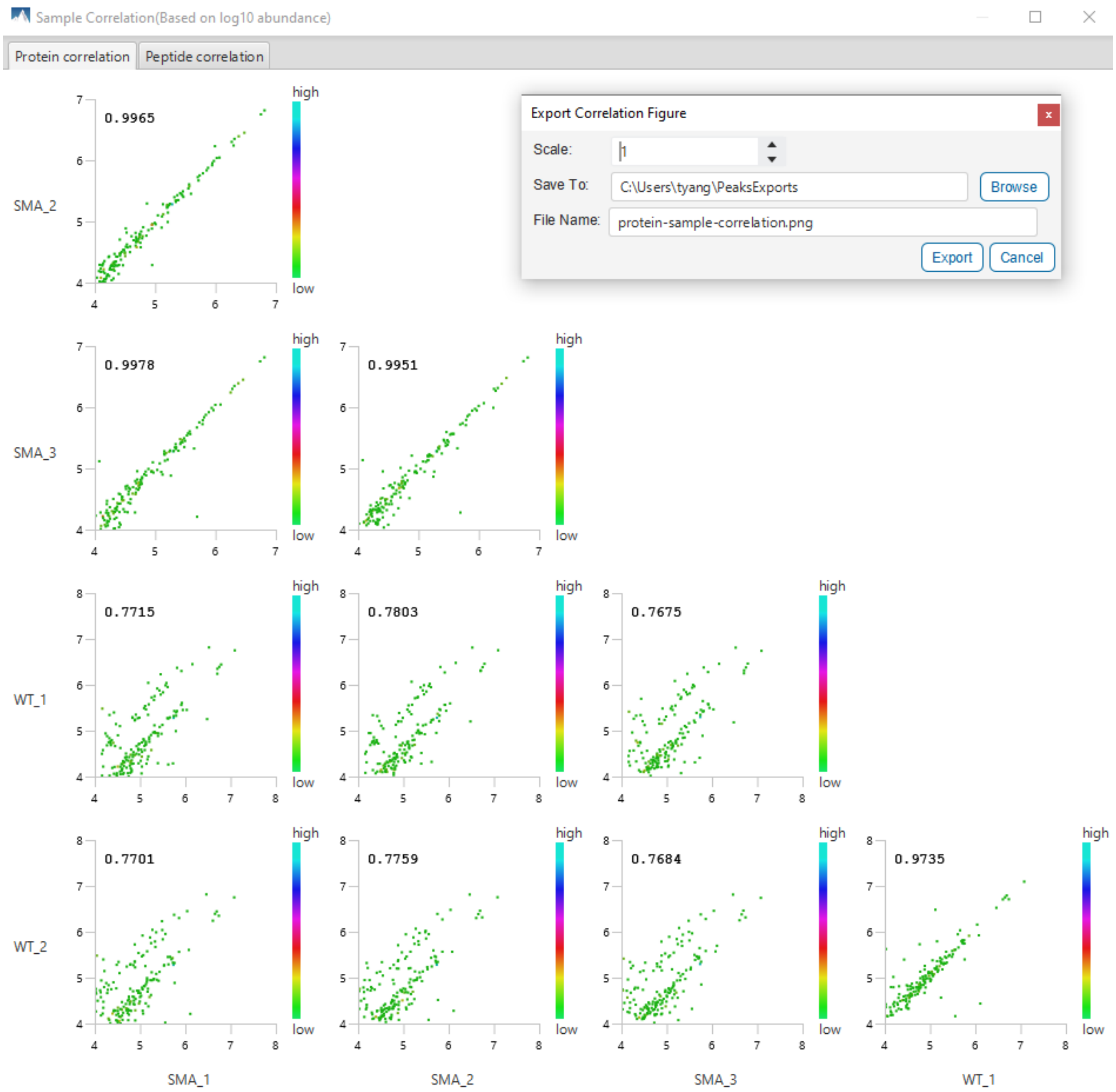

The Protein Correlation and Peptide Correlation plots will be displayed for the samples selected. The Pearson's correlation score indicates the reproducibility of the experiment based on a selected pair of samples. To export the image right click on the plots and click on "Export Image".

8.3.6 Summary View - Group Correlation plot

To view the abundance correlation plot for the detected features and proteins, click the 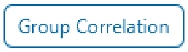

button from the Summary View title bar. A Correlation Plot Options window will pop-up to select which groups to include.

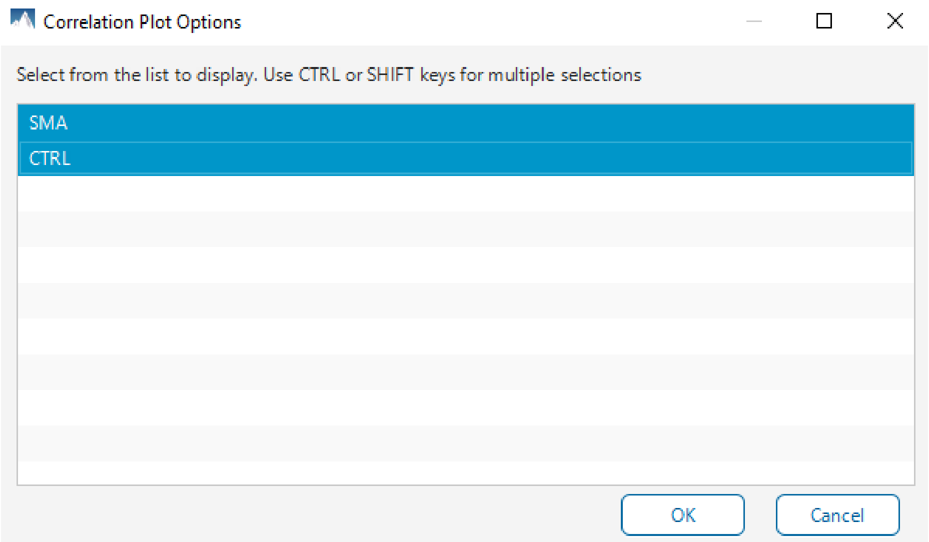

By default, the first 5 groups are selected. At most 5 groups can be used to generate the correlation plot, and at least 2 groups must be selected.

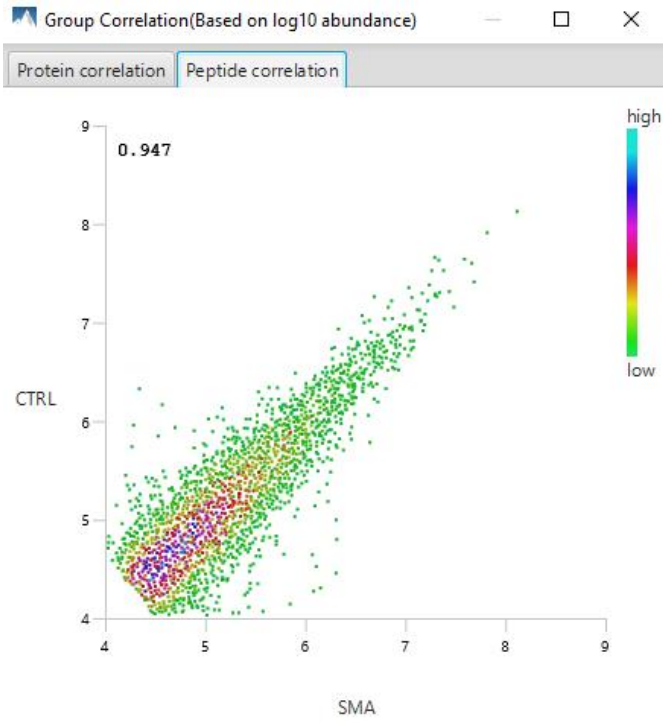

The Protein Correlation and Peptide Correlation plots will be displayed for the samples selected. The Pearson's correlation score indicates the reproducibility of the experiment based on a selected pair of samples. To export the image right click on the plots and click on "Export Image".

8.3.7 Summary View - Volcano plot

Figure 2. volcano plot combines a statistical test with the magnitude of the change enabling quick visual identification of those data-points that display large-magnitude changes and that are also statistically significant. The volcano plot in Figure 2(a) plots significance versus fold-change of the quantified proteins.

Horizontal broken grey line: The selected significance threshold

Vertical broken grey lines: The selected fold change thresholds

Plotting points in this way results in two regions of interest in the plot: those points that are found towards the top of the plot that are far to either the left or the right side. These represent values that display large magnitude fold changes (hence being left or right of center) as well as high statistical significance (hence being towards the top).

Figure 2. The volcano plot for proteins.

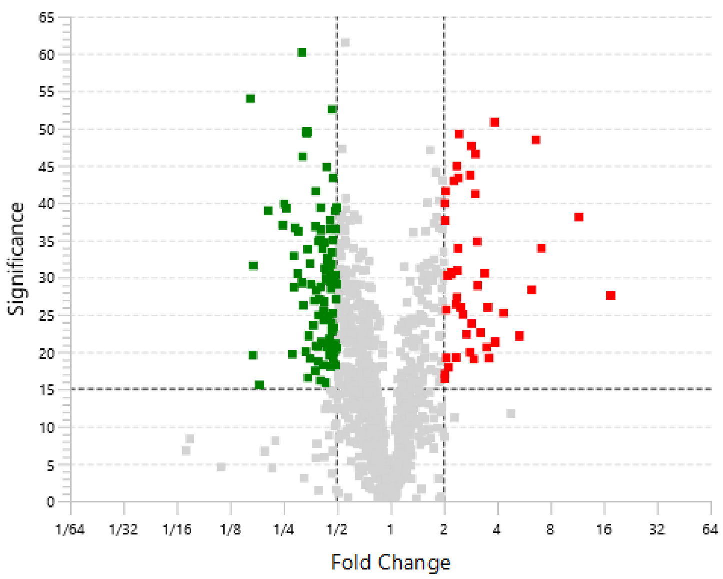

In this figure, the ratio is the group ratio set with respect to the base group. If more than two groups are present, the max ratio is used instead.

The Protein View contains an interactive volcano plot that can be hidden or displayed. Markers for the proteins that are above the set significance threshold will be displayed in colour. Scrolling the cursor over a marker will highlight it and, subsequently, clicking on this marker will highlight the protein in the interface.

8.3.8 Summary View - RT and m/z Shift Distribution

Figure 3(a) plots the retention time shift distribution to the base sample. Figure 3(b) plots the distribution of the m/z shift (in ppm) of the data with respect to the base sample. These two figures can be used to refine the parameter settings for label free quantification.

Figure 3. (a) RT shift distribution; **(b)** M/Z shift distribution.

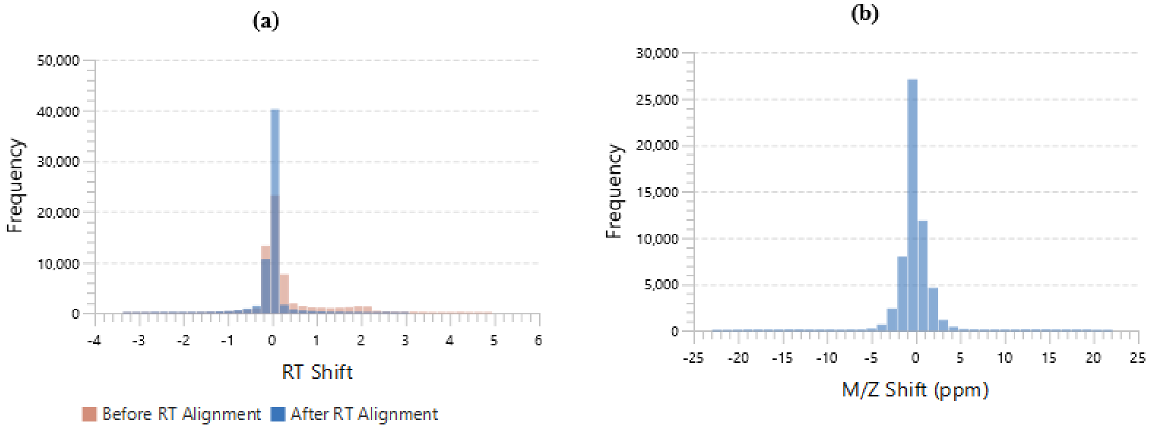

8.3.9 Summary View - Missing Value percentage chart

Figure 4 shows the percentage of missing values before and after ID transfer for each sample.

Figure 4. Percentage of missing values in each sample with or without ID transfer.

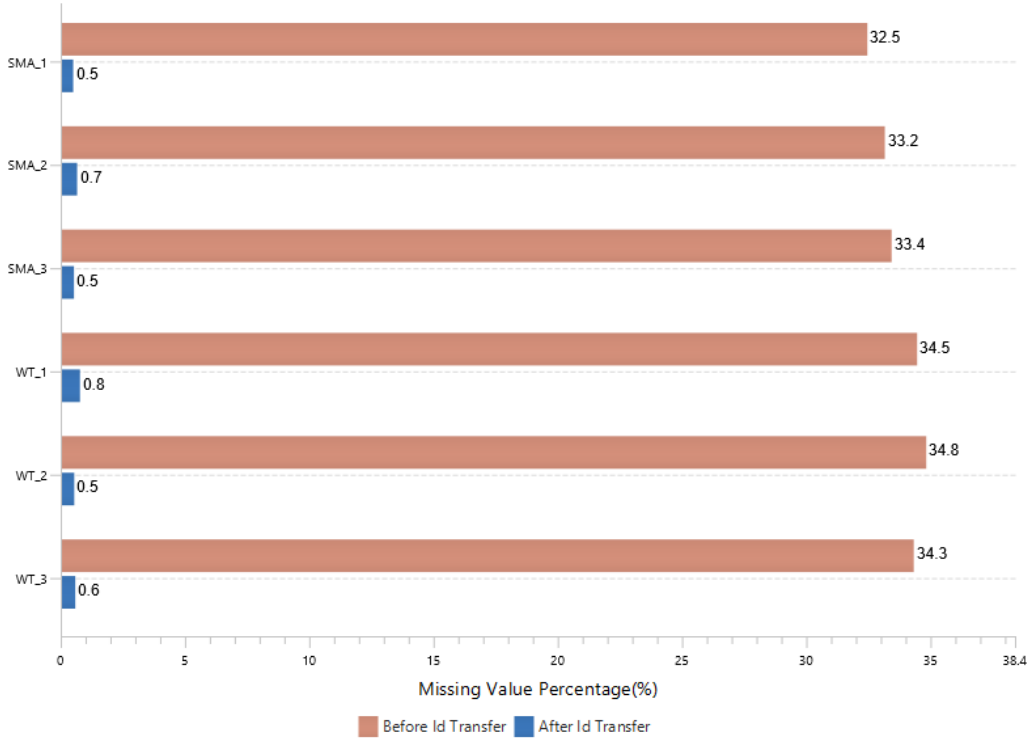

8.3.10 Summary View - Filtration parameters and statistics

Tables 1 and 2 list the filtration parameters and filtered results, respectively.

Table 1. Result filtration parameters.

Significance	≥ 15
Min Fold Change	≥ 2
Max Fold Change	≤ 64
Used peptides	≥ 1

Table 2. Statistics of filtered result.

Features	56866
Features with ID	37885
Feature vectors with ID	9535
Protein groups	141

8.3.11 Summary View - Search parameters

Table 3 lists the parameters as set in the workflow.

Table 3. Search Parameters

Search Engine Name: PEAKS
Quantification type: Label free quantification
LFQ method: Identification directed quantification
Mass Error Tolerance: 20.0 ppm
Retention time shift tolerance: Auto detected
Feature intensity ≥ 10000.0
Retention time range: [0.0 - Max]
Base sample: WT_1
Peptide Feature Filter:
- Avg area ≥ 20000.0
- Quality ≥ 20.0
- Charge between: [2 - 5]
- Peptide ID count ≥ 0 per group
- Detected in at least: 1 samples per group

Protein Filter:
- Significance method: ANOVA
- Modified Form Exclusion
- Remove Outlier
- Use Top 3 peptide
- Significance ≥ 0.0
- Fold change between: [1.0 - 64.0]
- Has at least: 1 used peptide

Normalization method: Use TIC
Samples: 6 samples in 2 groups
- SMA:
  - SMA_1
  - SMA_2
  - SMA_3
- CTRL:
  - WT_1
  - WT_2
  - WT_3

8.4 LFQ - Protein View

The **Proteins View** lists all the quantified proteins present in the sample, characterizes each protein at the amino acid level, and lists the supporting peptide features of each protein. It has six components:

Protein Table: Lists all the quantified proteins present in the sample.

Protein View Filters: Allows filtering the protein table to select specific proteins.

Protein Volcano Plot: Click on a marker that is above the significance threshold (the horizontal dashed line) set in the Summary View. Upon selection, the corresponding protein in the Protein Table will be highlighted.

PTM Profiling: Click on to open PTM profiling.

Coverage: Characterizes the protein sequence at the amino acid level. All the PTMs occurred on the protein sequence will be displayed together with the MS/MS data supporting the inference.

Peptides: Lists the supporting peptides identified from the selected proteins in the protein table.

8.4.1 Proteins View - Protein Table controls

At the top of the Proteins View, there are the options to filter, show/hide columns, search, and export the protein table.

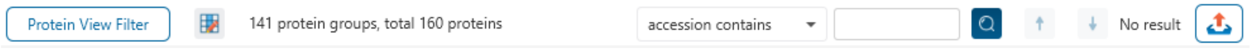

8.4.1.1 Protein Table controls - Protein View Filters

Click on the Protein View Filter button to open the Protein View Filter.

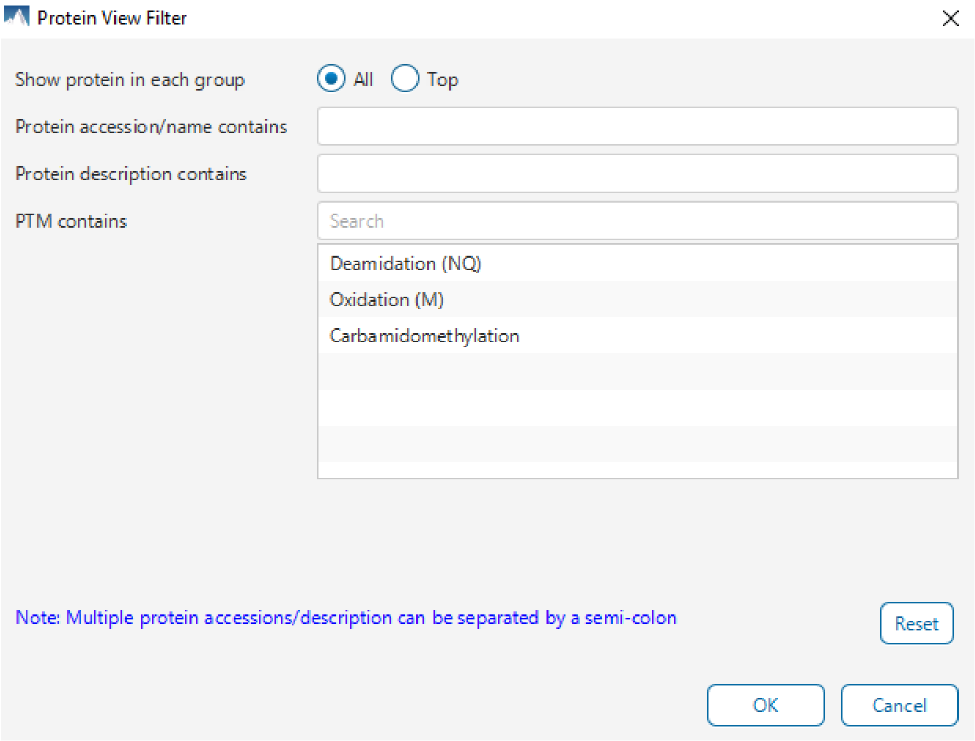

The following options are available for filtering the protein table:

Show All or Top proteins in each group: Adjust the protein list based on Protein grouping, top is selected by default. Proteins are grouped based on parsimony.

- **Top:** Show only the top proteins in each group. These proteins are supported by the most unique peptides in the group. Proteins in the group that share a subset of the unique peptides that support the top protein (subproteins) will not be displayed.
- **All:** Show all proteins in each group. Proteins are grouped together if they are supported by the same set or a sub-set of the top protein in the group. If a protein is supported by a peptide not supporting the top protein it will be added to a new group.

Protein accession/name contains: Restrict the protein table to only display protein IDs which contain the entered text in its accession/name.

Protein description contains: Only proteins with descriptions that contain the entered text will be included in the protein table if text is entered in this field.

Note: In these above two filters, multiple entries can be separated by a semi-colon.

PTM contains: Filter protein results by the presence of selected PTMs. Proteins that are supported by peptides containing modifications that match the selected PTMs will be included. If multiple PTMs match the modification name, they will all be included in the table. For SPIDER results, the mutations will also be listed in the PTM contains filter.

Click on the 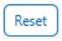

button to remove all filters.

Click on 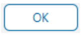

to apply the Protein View Filter to the Protein table.

Click on 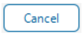

to discard changes and close the dialog.

8.4.1.2 Protein Table controls - Protein count

At the top of the protein table shows the count of protein groups and number of proteins in the table based on the current Protein View Filters.

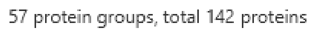

8.4.1.3 Protein Table controls - Optional columns

Click on the button to see a list of sample-based optional columns.

Select the corresponding sample-based and group-based ratio in the Protein Table view.

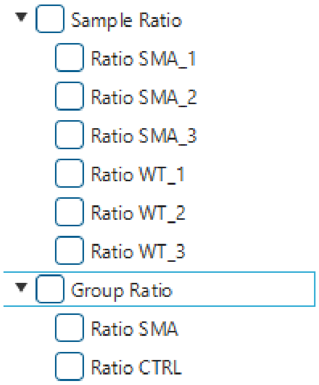

The exports will always include the contents of these optional columns even if they are hidden.

8.4.1.4 Protein Table controls - Protein Table Search Function

An easy-to-use search function is available for the protein table. The protein accession or description substring can be searched for, and all search results can be navigated through with the Up and Down arrows.

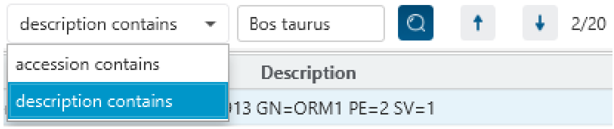

Protein accession/name contains: Restrict the protein table to only display protein IDs which contain the entered text in its accession/name.

Protein description contains: Only proteins with descriptions that contain the entered text will be included in the protein table if text is entered in this field.

8.4.1.5 Protein Table controls - Protein Table Export

Click on the button to export the contents of the current table. This considers the Protein View Filters, the sorting applied to the table and is consistent with the view. There is the option to export the Protein table and/or the Supporting peptide table in CSV format.

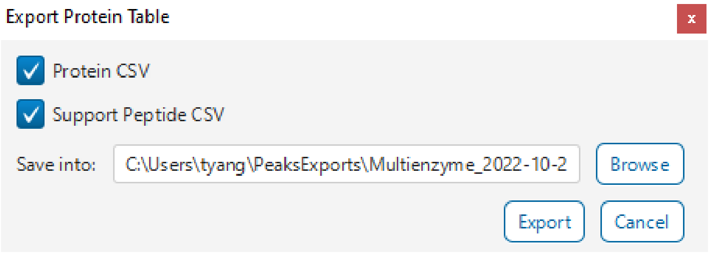

8.4.2 Proteins View - Protein Table

Each row in the table represents a group of proteins that are supported by a common set of peptides. To expand the group, click the right arrow button at the left.

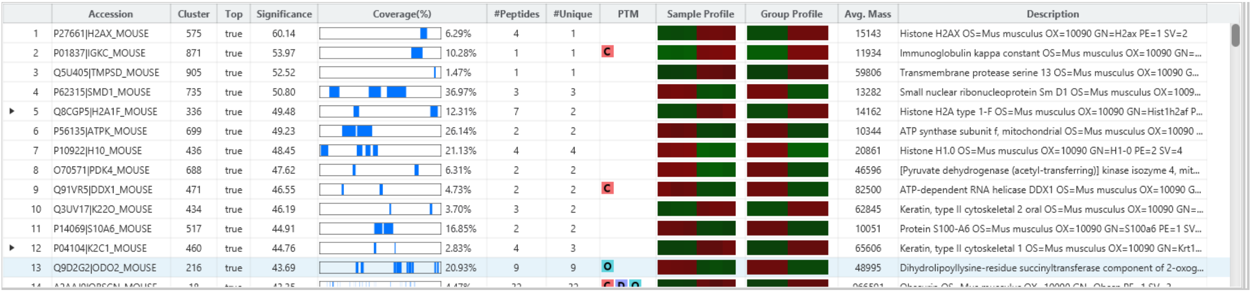

	Accession	Cluster	Top	Significance	Coverage(%)	#Peptides	#Unique	PTM	Sample Profile	Group Profile	Avg. Mass	Description
1	P27661|H2AX_MOUSE	575	true	60.14	6.29%	4	1				15143	Histone H2AX OS=Mus musculus OX=10090 GN=H2ax PE=1 SV=2
2	P01837|IGKC_MOUSE	871	true	53.97	10.28%	1	1	C			11934	Immunoglobulin kappa constant OS=Mus musculus OX=10090 GN=...
3	Q5U405|TMPSD_MOUSE	905	true	52.52	1.47%	1	1				59806	Transmembrane protease serine 13 OS=Mus musculus OX=10090 G...
4	P62315|SMD1_MOUSE	735	true	50.80	36.97%	3	3				13282	Small nuclear ribonucleoprotein Sm D1 OS=Mus musculus OX=1009...
▸ 5	Q8CGP5|H2A1F_MOUSE	336	true	49.48	12.31%	7	2				14162	Histone H2A type 1-F OS=Mus musculus OX=10090 GN=Hist1h2af P...
6	P56135|ATPK_MOUSE	699	true	49.23	26.14%	2	2				10344	ATP synthase subunit f, mitochondrial OS=Mus musculus OX=10090 ...
7	P10922|H10_MOUSE	436	true	48.45	21.13%	4	4				20861	Histone H1.0 OS=Mus musculus OX=10090 GN=H1-0 PE=2 SV=4
8	O70571|PDK4_MOUSE	688	true	47.62	6.31%	2	2				46596	[Pyruvate dehydrogenase (acetyl-transferring)] kinase isozyme 4, mit...
9	Q91VR5|DDX1_MOUSE	471	true	46.55	4.73%	2	2	C			82500	ATP-dependent RNA helicase DDX1 OS=Mus musculus OX=10090 G...
10	Q3UV17|K22O_MOUSE	434	true	46.19	3.70%	3	2				62845	Keratin, type II cytoskeletal 2 oral OS=Mus musculus OX=10090 GN=...
11	P14069|S10A6_MOUSE	517	true	44.91	16.85%	2	2				10051	Protein S100-A6 OS=Mus musculus OX=10090 GN=S100a6 PE=1 SV...
▸ 12	P04104|K2C1_MOUSE	460	true	44.76	2.83%	4	3				65606	Keratin, type II cytoskeletal 1 OS=Mus musculus OX=10090 GN=Krt1...
13	Q9D2G2|ODO2_MOUSE	216	true	43.69	20.93%	9	9	O			48995	Dihydrolipoyllysine-residue succinyltransferase component of 2-oxog...

The columns in the Protein Table are:

- **Accession:** The accession number of the protein as seen in the FASTA database.
- **Cluster:** A unique identifier that classifies which protein group the protein belongs to.
- **Top:** Indicates whether this protein is a top protein.
- **Significance:** The protein significance.
- **Coverage (%):** The percentage of the protein sequence that is covered by supporting peptides. The coverage is visualized by a colored bar. Light blue blocks indicate the parts of the sequence covered by low-confidence peptides. Dark blue blocks indicate the parts covered by high-confidence peptides. This is the total coverage including results from all samples.
- **#Peptides:** The number of high-confidence supporting peptides.
- **#Unique:** The number of high-confidence supporting peptides that are mapped to only one protein group. Unique peptides with same sequence but different modifications are only counted once in this number.
- **PTM:** The identified modifications displayed with color-coded icons.
- **Sample Profile:** The protein abundance among the samples is depicted as a heat map. Hold the cursor on a profile to view the sample channels, abundances, and ratios with respect to the base sample.
- **Group Profile:** The protein abundance among the groups is depicted as a heat map. The Group Profile is determined by calculated the total abundance (area) of supporting peptides within a group. Hold the cursor on a profile to view the group channels, abundances, and ratios compared to the group that contains the base sample.
- **Avg. Mass:** The protein mass calculated using the average mass.
- **Description:** The protein's header information as seen in the FASTA database.

Note: For #Peptides and #Unique, two peptides with the same starting and ending positions in the protein are counted as one, regardless of their PTM forms. This is to follow the MCP (Molecular & Cellular Proteomics) guidelines.

8.4.3 Proteins View - Coverage Pane

The Protein Coverage view visually maps the supporting peptides and de novo tags to the protein selected in the Protein table. It also shows all identified sites with modifications or mutations to assist with protein characterization at the amino acid level. The "Protein Coverage" view is composed of three major components - Protein Sequence Coverage, Coverage Control Panel, and Protein Tools.

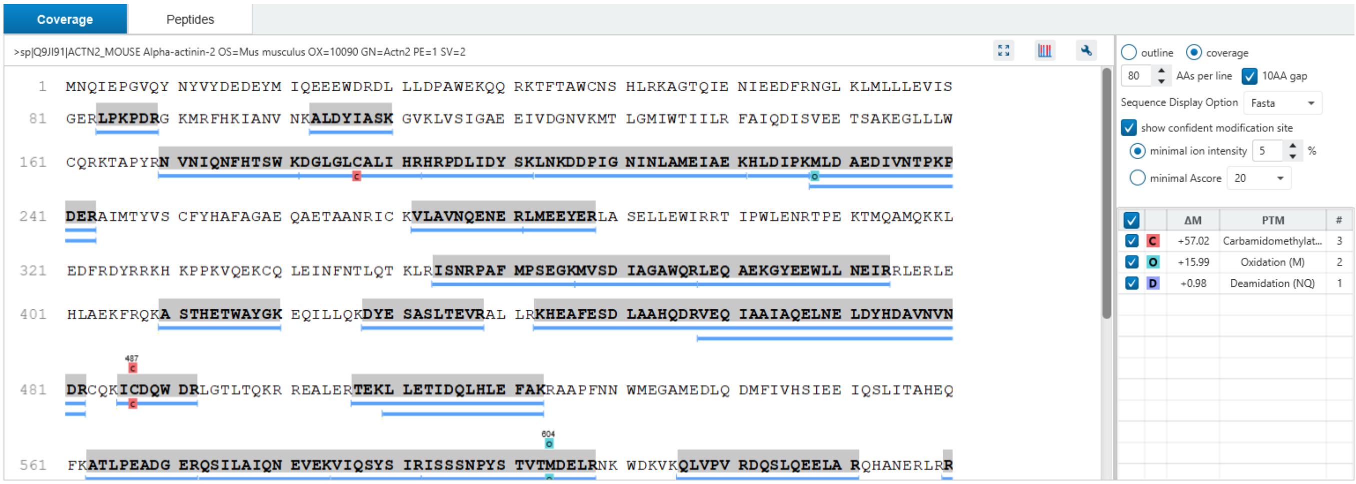

8.4.3.1 Protein Sequence Coverage

This area visualizes the coverage of the protein sequence.

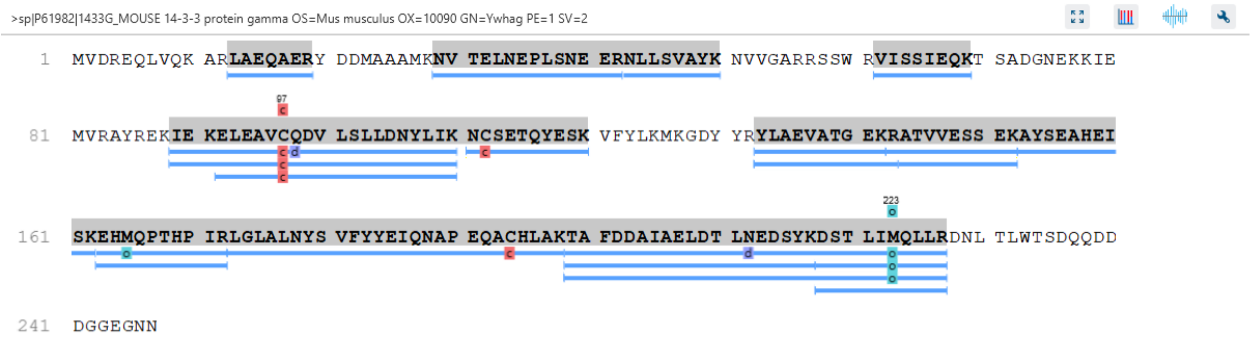

Regions in the protein sequence that are covered by supporting peptides are displayed in bold font with a grey background. Confident modifications identified in supporting peptides are displayed as icons above the protein sequence. Modifications are represented by colored icons with the first letter of its modification name. If a residue is modified by more than one modification in the same supporting peptide, "*" is used instead of a letter. The header information of the protein is shown on the top of the protein sequence.

The supporting peptides and matched de novo tags can be shown as colored bars under the protein sequence.

Placing the cursor over a bar shows a tooltip with detailed information of the peptide.

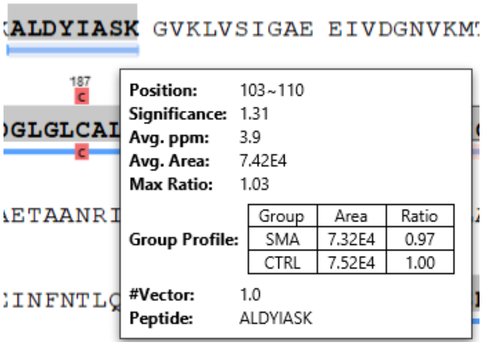

Left clicking on a bar shows the details from which the supporting peptide is identified.

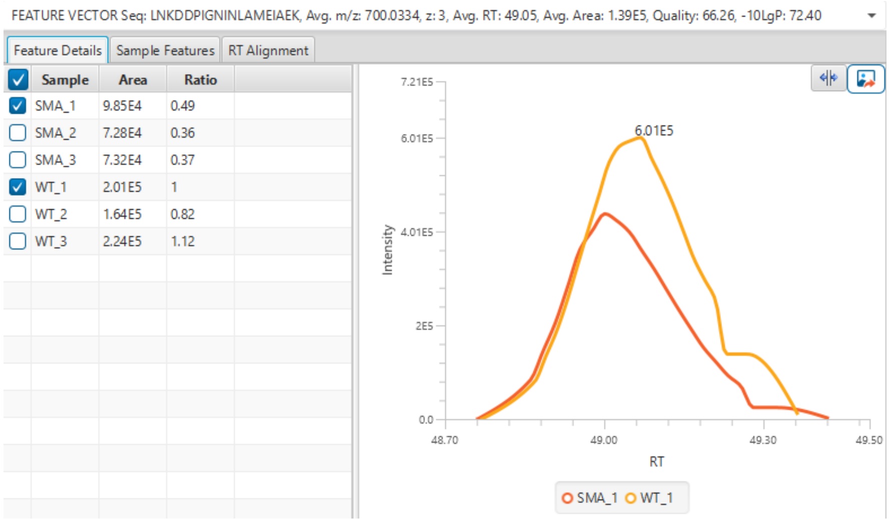

Right clicking on a peptide bar shows additional options. “Show in spectrum” has the same behaviour as left clicking on the peptide bar which is to open the annotated spectrum chart. “Show in table” has the function to jump to the supporting peptides table.

8.4.3.2 Coverage Control Panel

This panel controls what to display in the protein sequence coverage view.

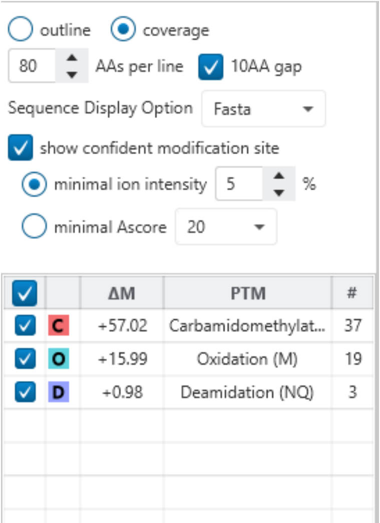

Sequence Display Mode: The protein sequence can be displayed in either "outline" or "coverage" mode.

Outline mode: In outline mode, the protein sequence is displayed without supporting peptides and matched de novo tags. However, the sequence coverage at a specific position can be examined by left-clicking a residue.

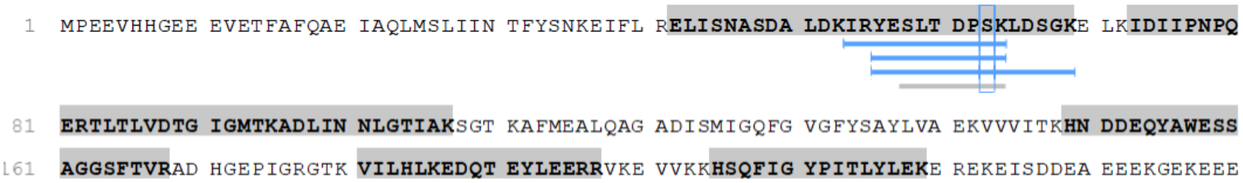

Coverage mode: In coverage mode, all the supporting peptides and matched de novo tags are shown.

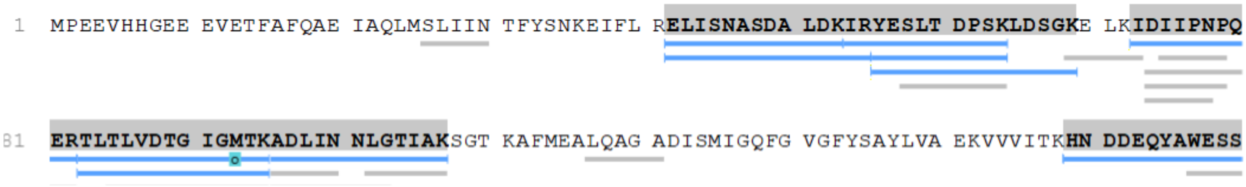

AAs per line and 10AA gap: Specify the number of amino acids per line and whether to show a gap for every 10 amino acids in the protein sequence.

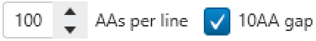

Sequence Display Option: Select whether to view the protein sequence as Fasta or a specific enzyme used in the project. When a particular enzyme is selected, the protein sequence will be highlighted to emphasize expected peptides generated by the selected enzyme's cleavage specificity.

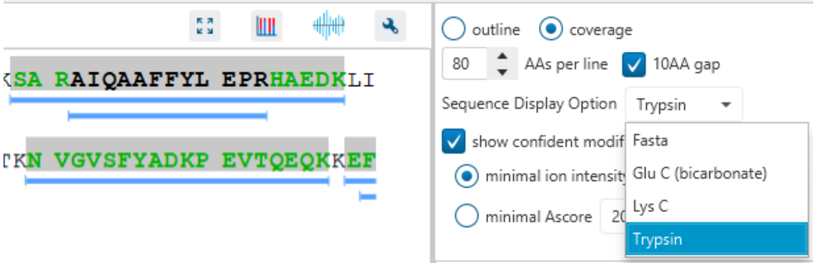

Modification minimal ion intensity / Ascore: A modification or mutation is considered confident if there are fragment ions supporting the modified residue with the minimal peak intensity above the specified threshold. Only confident modifications and mutations are displayed as icons above the protein sequence.

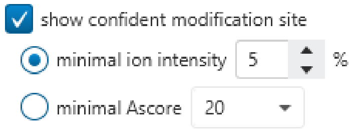

Modification table: The modification table shows the modifications identified in supporting peptides on the protein. For each modification, the number of supporting PSMs with this particular modification is shown. The checkbox on the left controls whether to indicate the modification in the protein coverage.

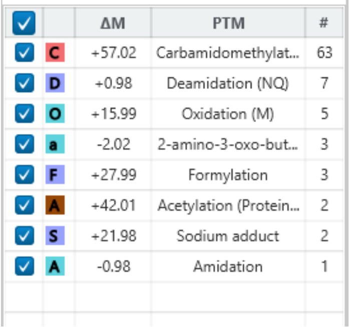

		ΔM	PTM	#
	C	+57.02	Carbamidomethylat...	63
	D	+0.98	Deamidation (NQ)	7
	O	+15.99	Oxidation (M)	5
	a	-2.02	2-amino-3-oxo-but...	3
	F	+27.99	Formylation	3
	A	+42.01	Acetylation (Protein...	2
	S	+21.98	Sodium adduct	2
	A	-0.98	Amidation	1

8.4.3.3 Proteins View - Protein Tools

The Proteins View toolbar is at the upper-right corner of the protein sequence coverage tab and contains helpful protein tools.

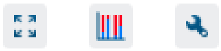

Full screen: 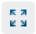 When selected, the protein coverage window expands in full screen mode. Click to return from full screen mode.

Tool box: 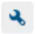 The tool box contains the following tools:

- Copy protein sequence. This copies the protein sequence of the current selected protein into the system clipboard.
- Save protein coverage as image. Different scale options are provided to increase the image size.
- Coverage statistics. This provides useful statistics based on the current selected protein.

PTM Profiling: 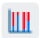 Calculates the difference in abundance between peptides with confidently identified PTMs versus unmodified peptides. Peptide feature areas are used for this comparison.

The PTM Profiling tool provides quantitative information of modified peptides compared with unmodified peptides for the modification sites of the protein across all MS samples.

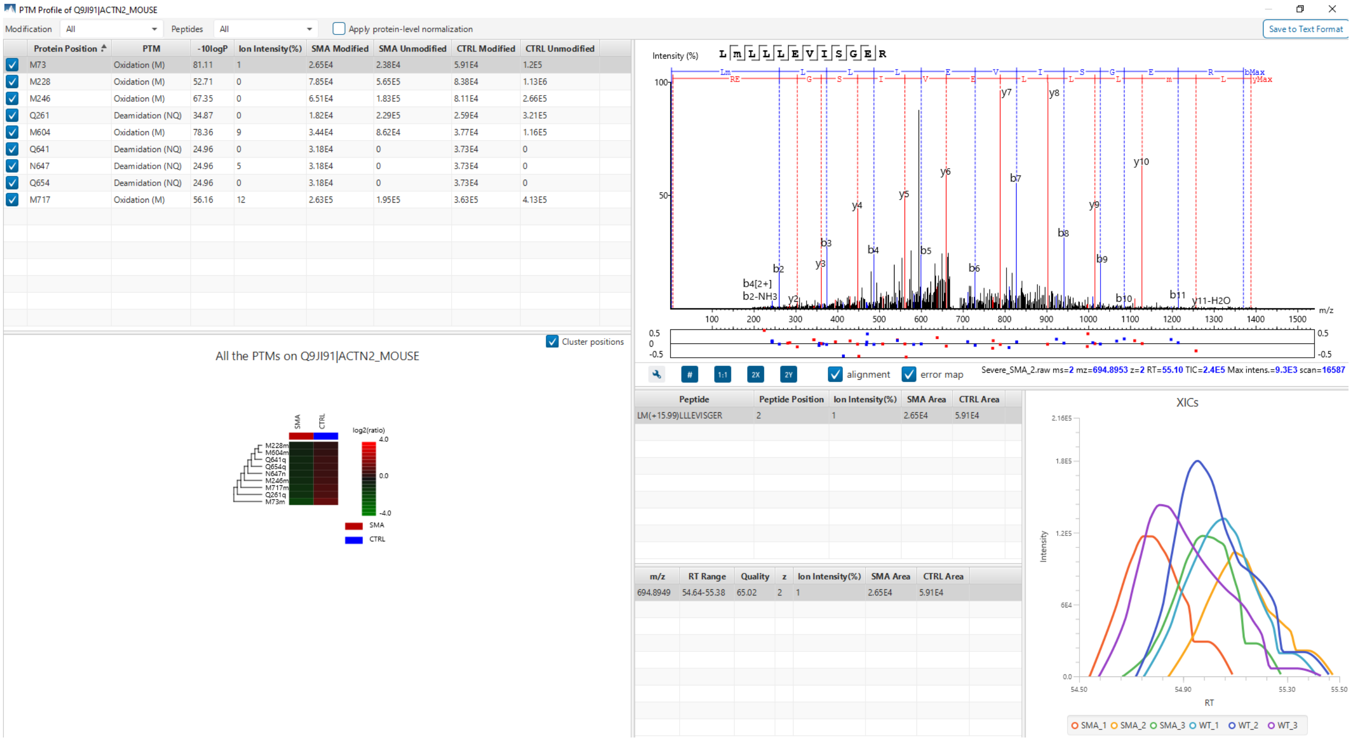

The inputs for the profiling can be set using the drop-down lists at the top-left corner of the dialog.

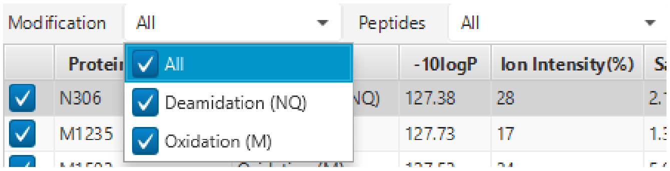

- **Proteins:** The accession ID of the selected protein to be analyzed for PTM profiling is shown in the title of the dialog. The selected protein can not be changed within the dialog. To run profiling on another protein, close the dialog, select the protein of interest from the protein table and run the profiling by clicking button.
- **Modification:** The drop-down list contains the detected and selected confident PTMs for the protein. Select the type of modification to study a specific PTM or choose “All the PTMs” to visualize the profiling information of all PTM sites at together.

  Note: The fixed PTMs are not used for profiling. In addition to this, the labeling modifications are not considered either.

- **Peptides:** Data source used for the analysis. “All” considers all the peptides for this protein.
- **Apply protein-level normalization:** By default, the protein-level normalization is not applied. If checked, the displayed modified/unmodified area is obtained by dividing the true modified/unmodified area by the corresponding ratio of the selected protein.

PTM Profile Table

The PTM Profile Table shows the detailed quantitative information for the modification sites. Each row in the table shows the modified and unmodified area for each sample of the selected PTM at each detected modification site. The table can be sorted by any column. The following list describes the contents in each column:

- **(Check box):** Allow the selection of each table row. Only the marked rows are used for the PTM Profile Chart.
- **Protein Position:** The detected modification sites in the protein. The modified amino acid symbol together with the position in the protein sequence is shown.
- **PTM:** The type of PTM being profiled.
- **-10logP:** The best -10logP identification score for the corresponding modified peptides.
- **AScore/ion intensity:** The best PTM AScore / ion intensity associated with the corresponding PTM at the specified site of the modified peptides.
- **Group Modified:** Total area of the features associated with the corresponding modified peptides for each sample.
- **Group Unmodified:** Total area of the features associated with the corresponding unmodified peptides for each sample.

Protein Position	PTM	-10logP	Ion Intensity(%)	SMA Modified	SMA Unmodified	CTRL Modified	CTRL Unmodified
M73	Oxidation (M)	81.11	1	2.65E4	2.38E4	5.91E4	1.2E5
M228	Oxidation (M)	52.71	0	7.85E4	5.65E5	8.38E4	1.13E6
M246	Oxidation (M)	67.35	0	6.51E4	1.83E5	8.11E4	2.66E5
Q261	Deamidation (NQ)	34.87	0	1.82E4	2.29E5	2.59E4	3.21E5
M604	Oxidation (M)	78.36	9	3.44E4	8.62E4	3.77E4	1.16E5
Q641	Deamidation (NQ)	24.96	0	3.18E4	0	3.73E4	0
N647	Deamidation (NQ)	24.96	5	3.18E4	0	3.73E4	0
Q654	Deamidation (NQ)	24.96	0	3.18E4	0	3.73E4	0
M717	Oxidation (M)	56.16	12	2.63E5	1.95E5	3.63E5	4.13E5

PTM Profile Heat Map

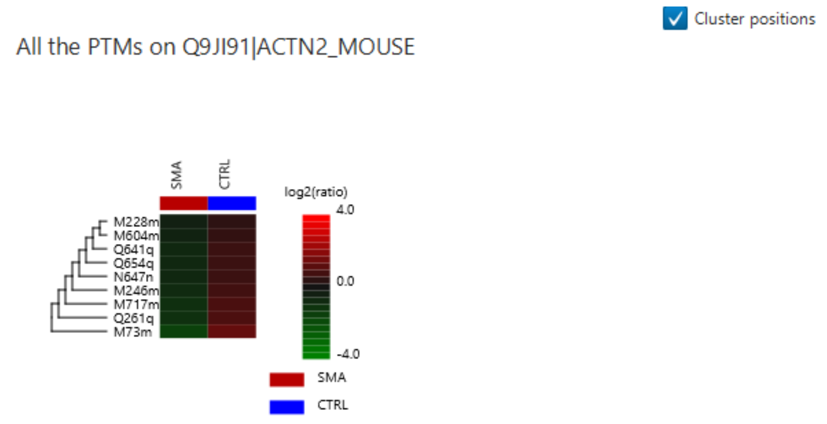

The PTM Profile Heat Map is located at the bottom left corner in the dialog. The relative abundance of modified peptides in all groups for the selected modification sites as a heat map. The color scheme is determined by the log2 ratios of the modified area of each group relative to the average abundance. For more details about the heat map, please refer to section 8.3.3 Summary View - Heat Map

Spectrum Annotation View

The Spectrum Annotation View provides a graphical representation for the best modified peptide and the best unmodified peptide when “All” is selected from the “Peptides” drop-down list. The top spectrum corresponds to the best modified peptide, whereas the bottom spectrum shows the best unmodified peptide.

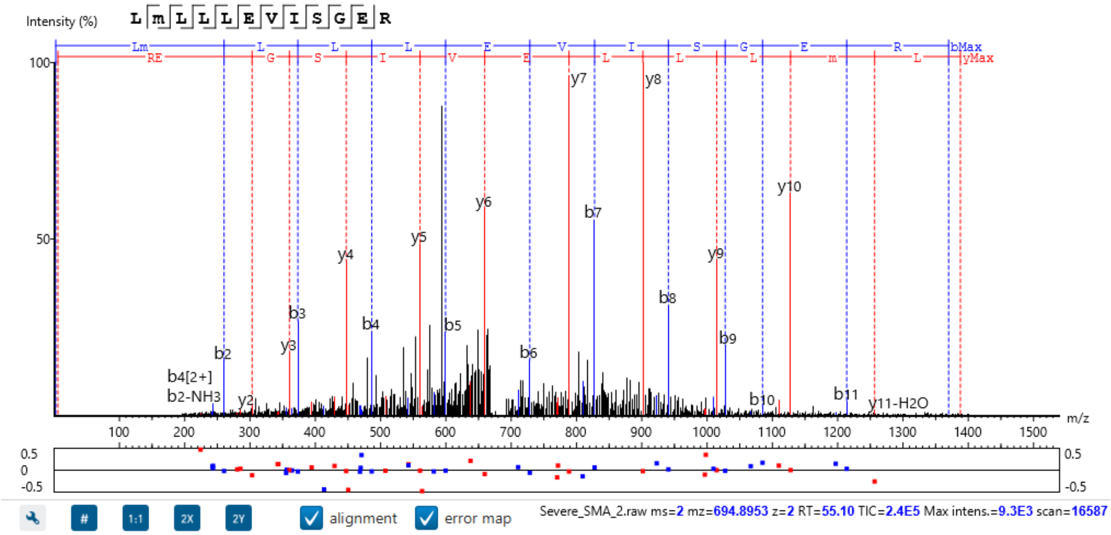

Peptides Table. Peptides table lists the modified peptides that contain the selected PTM at the specified modification site.

Peptide	Peptide Position	Ion Intensity(%)	SMA Area	CTRL Area
LM(+15.99)LLLEVISGER	2	1	2.65E4	5.91E4

All Matches Table. The supporting PSMs of the selected peptide in Peptides Table are listed in the All Matches Table. The headers are self-explanatory. The last column Area is the area of the feature that is associated with the corresponding PSM.

m/z	RT Range	Quality	z	Ion Intensity(%)	SMA Area	CTRL Area
694.8949	54.64-55.38	65.02	2	1	2.65E4	5.91E4

XICs. It shows the XICs figure for the selected modified match in the All Matches Table.

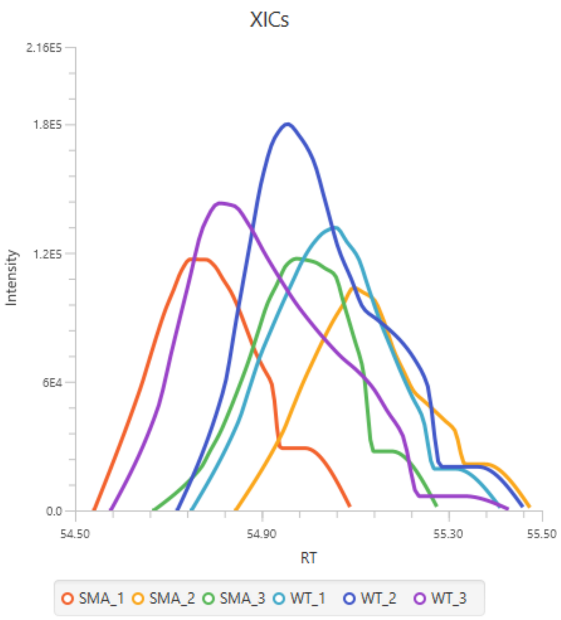

PTM Profile Export

The PTM profile can be exported as an image as well as in text format.

Image export: To save the profile chart, right click and choose Save to Image... menu item to save the displayed graph as a PNG file.

Text format export: Click Save to Text Format button at the top-right corner to export the calculated PTM Profile data to a text file in CSV format. The PTM Profile of the selected modification on the selected protein will be exported by default. In addition, profiles on all proteins for the selected modification may also be exported, if “Export ptm profile for all proteins” is checked. The exported file contains all necessary information to construct the PTM profile graph. The PTM profile data are grouped together by protein, then by modification type, and then by the modification site on the protein sequence. The headers of the CSV column are similar to the PTM Profile Table and also self-explanatory. The following items are explained for clarity:

- **Modified Peptide Sequence Window:** protein sequence containing 10 AA ahead of the modification site and 10 AA after the modification site with padding as necessary.
- **Sample Best AScore / Ion Intensity:** best AScore or Ion Intensity for each sample.

Note: When "Export PTM profile for all proteins" is not checked, only the profiling data that is marked in the PTM Profile Table will be exported.

8.4.3.4 Proteins View - Supporting Peptides

The supporting peptides assigned to the protein are shown in a separate tab beside the Protein Coverage view. The sequence can be clicked and a pop-up window will show up to display the feature details.

Coverage | Peptides

	Peptide	Used	Quality	Significance	Avg. ppm	Avg. Area	Sample Profile	Group Profile	SMA Area	CTRL Area	Max Ratio	RT mean	#Vector	Start	End	PTM
1	HLDIPK	√	76.33	30.02	0.8	7.15E5			4.64E5	9.66E5	2.08	20.73	1	222	227	
2	HRPDLIDYSK	√	80.22	34.33	0.6	1.7E6			1.21E6	2.19E6	1.80	24.06	2	193	202	
3	ILASDKPYILAEELR	√	82.25	36.92	0.4	1.37E6			7.53E5	1.98E6	2.63	42.48	2	837	851	
4	AC(+57.02)LISMGYDLGEAEFAR		65.24	4.06	0.9	1.02E5			9.93E4	1.06E5	1.06	53.76	2	780	796	C
5	ALDYIASK		65.94	1.31	3.9	7.42E4			7.32E4	7.52E4	1.03	26.47	1	103	110	
6	ASTHETWAYGK		65.51	40.64	0.9	2.24E5			8.87E4	3.58E5	4.04	21.75	2	410	420	
7	ATLPEADGER		74.67	16.14	0.9	1.84E5			1.99E5	1.69E5	1.18	22.28	1	563	572	
8	ATLPEADGERQSILAIQNEVEKVIQSY...		60.47	22.97	1.8	3.4E4			2.29E4	4.52E4	1.98	64.46	1	563	592	
9	DGLGLC(+57.02)ALIHR		70.07	29.20	0.6	1.67E5			1.08E5	2.25E5	2.09	42.86	2	182	192	C
10	DQSLQEELAR		76.98	20.66	0.4	1.42E5			1.59E5	1.25E5	1.27	32.32	1	622	631	
11	DYESASLTEVR		61.85	0.73	1.4	4.27E4			4E4	4.54E4	1.14	33.07	1	428	438	
12	ETADTDTAEQVIASFR		67.27	7.10	0.8	2.2E5			1.74E5	2.66E5	1.53	49.58	1	821	836	
13	GITQEQMNEFR		71.17	0.01	0.5	7.14E4			7.18E4	7.09E4	1.01	32.22	1	749	759	
14	IC(+57.02)DQWDR		73.82	4.18	0.9	1.05E5			1.08E5	1.03E5	1.05	25.82	1	486	492	C
15	ISNRPAFMPSEGK		63.72	26.58	0.9	1.61E5			1.2E5	2.06E5	1.72	26.55	2	354	366	
16	ISSSNPYSTVTM(+15.99)DELR		61.78	4.01	1.4	3.6E4			3.44E4	3.77E4	1.10	36.46	1	593	608	O
17	ISSSNPYSTVTMDELR		67.35	10.46	0.5	1.01E5			8.62E4	1.16E5	1.34	40.54	1	593	608	
18	KHEAFESDLAAHQDRVEQIAAIAQEL...		73.37	11.54	0.8	4.39E5			2.53E5	6.25E5	2.47	53.17	1	443	482	
19	LEQAEKGYEEWLLNEIR		64.90	23.55	0.6	5.08E4			6.47E4	3.68E4	1.76	51.03	1	378	394	
20	LLETIDQLHLEFAK		71.18	42.03	1.3	4.76E5			2.61E5	6.91E5	2.65	48.31	2	510	523	

This table contains the following information of the quantifiable support peptides for the selected protein:

- **Peptide:** The amino acid sequence of the peptide, as determined in PEAKS Database or Spectral Library search. A modified residue is followed by a pair of parentheses enclosing the modification mass. All non-unique peptides and those not passing the filter will be excluded automatically.
- **Used:** Shows whether this peptide is used to calculate the protein abundance. The top three peptides with highest abundance are used to calculate the protein abundance.
- **Quality:** The feature vector quality score.
- **Significance:** The -10lg of a p-value represents the likelihood that the observed change between conditions is caused by random chance. The peptides are first separated into groups based on similar quality score. Significance is then calculated for each quality score group, or bin, using a two-tail T-test that assumes log normal distribution but does not assume equal variance. The -10log p-value is then calculated from this result and then displayed in the significance column.
- **Avg. ppm:** A peptide can have one or more feature vectors. Each feature vector has its own ppm calculated from the mass error of each feature. The average mass error of a peptide is the average of mass errors of all the feature vectors. This column displays the average mass error in ppm.
- **Avg. Area:** Each feature in the feature vector has its own area under the XIC curve. The average area of a feature vector is the average area of all the features associated with the peptide.
- **Sample Profile:** The peptide abundance among the samples is depicted as a heat map. Hold the cursor on a profile to view the samples, the peptide areas in samples, and the corresponding ratios with respect to the base sample.
- **Group Profile:** The peptide abundance among the groups is depicted as a heat map. Hold the cursor on a profile to view the groups, the peptide areas in groups, and the corresponding ratios with respect to the group which contains the base sample.
- **Group Area:** This column shows the peptide area for a group. It is calculated by summing up the areas of feature vectors within a group
- **Max Ratio:** This shows the maximum of the fold change values compared to the reference sample.
- **RT Mean:** Each feature in the feature vector has its own retention time center. This column displays the average retention time center of the feature vector.
- **#Vector:** This shows the number of quantifiable feature vectors of a peptide.
- **Start:** This shows the protein position of the first residue of the peptide.
- **End:** This shows the protein position of the last residue (inclusive) of the peptide.
- **PTM:** The types and the numbers of modifications present in the peptide shown using color-coded icons. Scroll over each icon to see the modification name and mass.

- **Feature Detail:** Double click on a supporting peptide to go to the peptide page where the eXtracted Ion Chromatogram (XIC) curves are displayed (as seen below).

8.5 LFQ result - Peptides View

Quantified proteins are displayed in the Peptide View along with their detailed information.

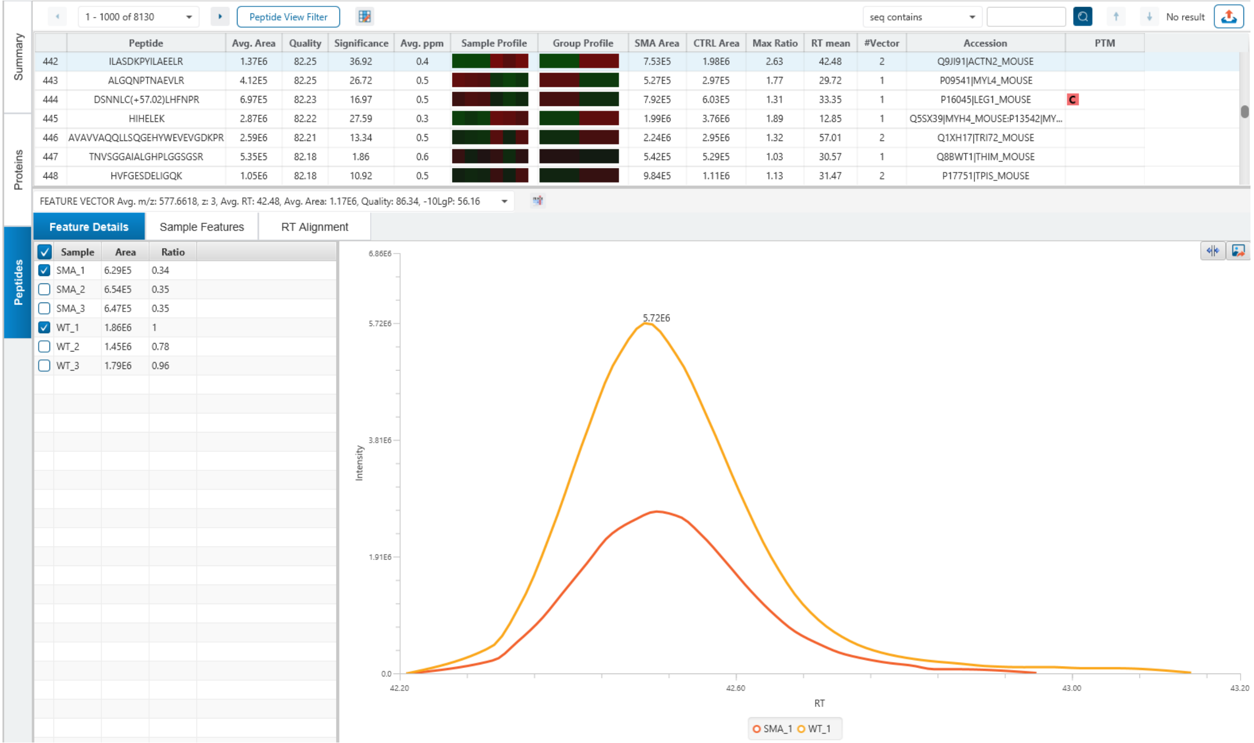

8.5.1 Peptides View - Peptide Table

The peptides are grouped together based on quantifiable feature vectors with the same sequence. When there are more than 1000 peptides, the table is split into multiple pages.

	Peptide	Avg. Area	Quality	Significance	Avg. ppm	Sample Profile	Group Profile	SMA Area	CTRL Area	Max Ratio	RT mean	#Vector	Accession	PTM
442	ILASDKPYILAEELR	1.37E6	82.25	36.92	0.4			7.53E5	1.98E6	2.63	42.48	2	Q9JI91|ACTN2_MOUSE	
443	ALGQNPTNAEVLR	4.12E5	82.25	26.72	0.5			5.27E5	2.97E5	1.77	29.72	1	P09541|MYL4_MOUSE	
444	DSNNLC(+57.02)LHFNPR	6.97E5	82.23	16.97	0.5			7.92E5	6.03E5	1.31	33.35	1	P16045|LEG1_MOUSE	C
445	HIHELEK	2.87E6	82.22	27.59	0.3			1.99E6	3.76E6	1.89	12.85	1	Q5SX39|MYH4_MOUSE:P13542|MY...	
446	AVAVVAQQLLSQGEHYWEVEVGDKPR	2.59E6	82.21	13.34	0.5			2.24E6	2.95E6	1.32	57.01	2	Q1XH17|TRI72_MOUSE	
447	TNVSGGAIALGHPLGGSGSR	5.35E5	82.18	1.86	0.6			5.42E5	5.29E5	1.03	30.57	1	Q8BWT1|THIM_MOUSE	
448	HVFGESDELIGQK	1.05E6	82.18	10.92	0.5			9.84E5	1.11E6	1.13	31.47	2	P17751|TPIS_MOUSE	

The view is similar to the support peptide table described in the previous section Section 8.4.3.4 Proteins View - Supporting Peptides. The Peptide table entries may be filtered by using the Peptide View Filters on top of the peptide table. To reiterate, the columns are:

- **Peptide:** The amino acid sequence of the peptide, as determined in PEAKS Database or Spectral Library search. A modified residue is followed by a pair of parentheses enclosing the modification mass. All non-unique peptides and those not passing the filter will be excluded automatically.
- **Avg. Area:** Each feature in the feature vector has its own area under the XIC curve. The average area of a feature vector is the average area of all the features associated with the peptide.

- **Quality:** The feature vector quality score.
- **Significance:** The -10lg of a p-value represents the likelihood that the observed change between conditions is caused by random chance. The peptides are first separated into groups based on similar quality score. Significance is then calculated for each quality score group, or bin, using a two-tail T-test that assumes log normal distribution but does not assume equal variance. The -10log p-value is then calculated from this result and then displayed in the significance column.
- **Avg. ppm:** A peptide can have one or more feature vectors. Each feature vector has its own ppm calculated from the mass error of each feature. The average mass error of a peptide is the average of mass errors of all the feature vectors. This column displays the average mass error in ppm.
- **Sample Profile:** The peptide abundance among the samples is depicted as a heat map. Hold the cursor on a profile to view the samples, the peptide areas in samples, and the corresponding ratios with respect to the base sample.
- **Group Profile:** The peptide abundance among the groups is depicted as a heat map. Hold the cursor on a profile to view the groups, the peptide areas in groups, and the corresponding ratios with respect to the group which contains the base sample.
- **Group Area:** This column shows the peptide area for a group. It is calculated by summing up the areas of feature vectors within a group
- **Max Ratio:** This shows the maximum of the fold change values compared to the reference sample.
- **RT Mean:** Each feature in the feature vector has its own retention time center. This column displays the average retention time center of the feature vector.
- **#Vector:** This shows the number of quantifiable feature vectors of a peptide.
- **Accession:** The proteins that the peptide was found in.
- **PTM:** The types and the numbers of modifications present in the peptide shown using color-coded icons. Scroll over each icon to see the modification name and mass.

To copy the sequence, right click on a row (or multiple rows) in the table and select “Copy selected peptide sequence(s)”. Another option is to mouse over any cell in the table and hitting Ctrl + C on your keyboard to copy the contents of those selected cells.

8.5.2 Peptide Table controls

The menu above the peptide table provides options to scroll through the table of peptides, filter the table, show optional columns and search for specific entries.

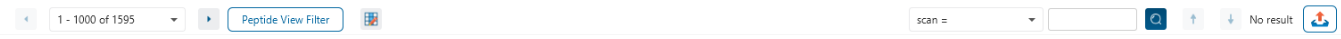

8.5.2.1 Peptide Table controls - Pagination

For easy viewing and to improve performance, the peptide table is limited to showing 1000 entries at once. Pagination is used to manage which set of 1000 entries will be viewed.

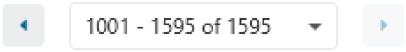

In the top left, there is a dropdown to select the rows for viewing. Click the left arrow or right arrow to select the previous/next 1000 rows. If the table is sorted or filtered, it may update the pagination set of rows that is currently in view.

8.5.2.2 Peptide Table controls - View Filter

Click on the Peptide View Filter button to open the Peptide View Filter which is used to filter the Peptide table. The following are the filter options provided:

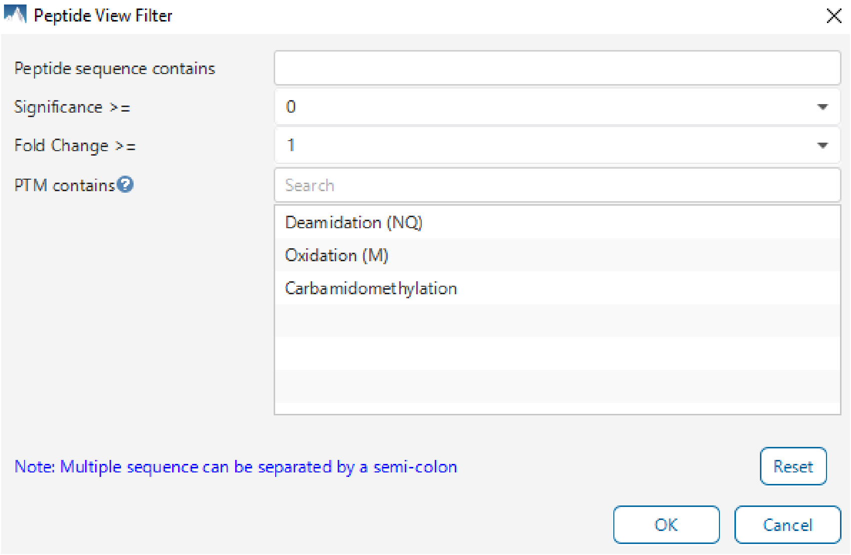

- **Peptide sequence contains:** Enter an amino acid sequence, only peptides that contain that sequence will be displayed in the peptide table. Adding PTMs in brackets in the sequence search is not handled, meaning only the sequence backbone is considered in the filter. Use the PTM contains for further filtering if necessary.
- **Significance >=:** Only peptides that pass this Significance threshold will remain in the table.
- **Fold Change >=:** Peptides with Max Ratio below this threshold will be removed from the table.
- **PTM contains:** Type the names of PTMs you would like to include. Peptides containing PTMs that match the text will be included. If multiple PTMs match the text they will all be included in the table.

Note: In all filters, multiple entries can be separated by a semi-colon.

Click on the Reset button to reset all filters to their default setting.

Click on OK to apply the filters to the Peptides table.

Click on Cancel to discard the changes in the View Filter.

8.5.2.3 Peptide Table controls - Optional Columns

Click on the 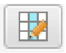 button to open the optional columns dropdown. The sample ratios and group ratios can be displayed in the peptide table for easy view and sorting. For projects with many samples, it might be useful to hide these optional columns and reduce the width of the peptide table.

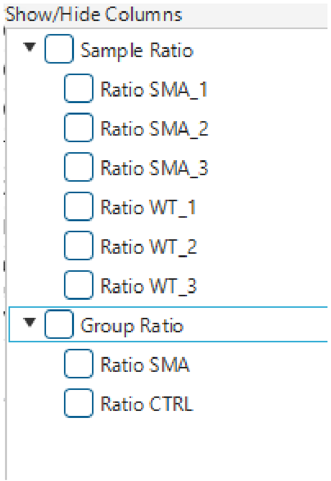

8.5.2.4 Peptide Table controls - Search function

On the top right of menu, there is a search function with 2 options. Search results can be navigated between using the Up or Down arrows.

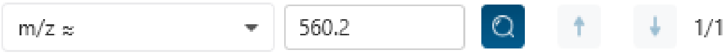

Options for searching include:

- **m/z ≈:** Search for the approximate m/z. The search will consider a range of m/z equal to the search value +/- 1 of the last significant digit. For example, searching for m/z ≈ 560.21 will find all m/z between [560.20, 560.22]
- **Seq contains:** Search for the sequence in the table. If specifying a sequence without PTMs, the results will include that sequence and any sequences with PTMs with the same backbone. If specifying a sequence with bracket and some mass value (like in the above image), then only sequence that have a substring with an exact match will be considered.
  Additionally, since the peptide table shows both I/L and they can not be distinguished, then searching for I in the sequence is equivalent to searching for L in the sequence. i.e. Searching for “LVLK” is the same as searching for “LVIK”, “IVLK”, and “IVIK”

8.5.3 Feature vector selection

Click on the Feature Vector dropdown to open a dropdown to display the feature vectors that correspond to the selected peptide. The peptide -10lgP score is the highest among the -10lgP scores of its feature vectors. The peptide quality score corresponds to the average of the quality scores of all feature vectors.

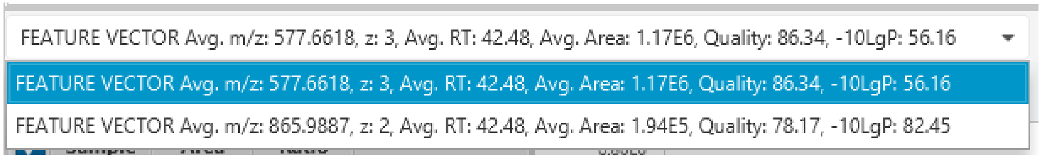

8.5.4 Protein jump button

Click on to jump to a protein that contains the currently selected PSM. If there are more than 1 protein, a dropdown is revealed which allows the user to select which protein to jump to. If a protein is filtered out due to a protein summary filter or summary page protein filter, then the option to jump will be grayed out.

8.5.5 Feature Details

The Feature Details contains the eXtracted Ion Chromatogram (XIC) chart. The eXtracted Ion Chromatogram (XIC) chart displays the shape of the selected peptide feature vector over the retention time range associated with the identification. The table on the left displays both the area and the ratio of the features in the highest average abundance feature vector. This table also works as a control to select the features that will be used to draw the XIC plot. By default, the first sample in each group is selected.

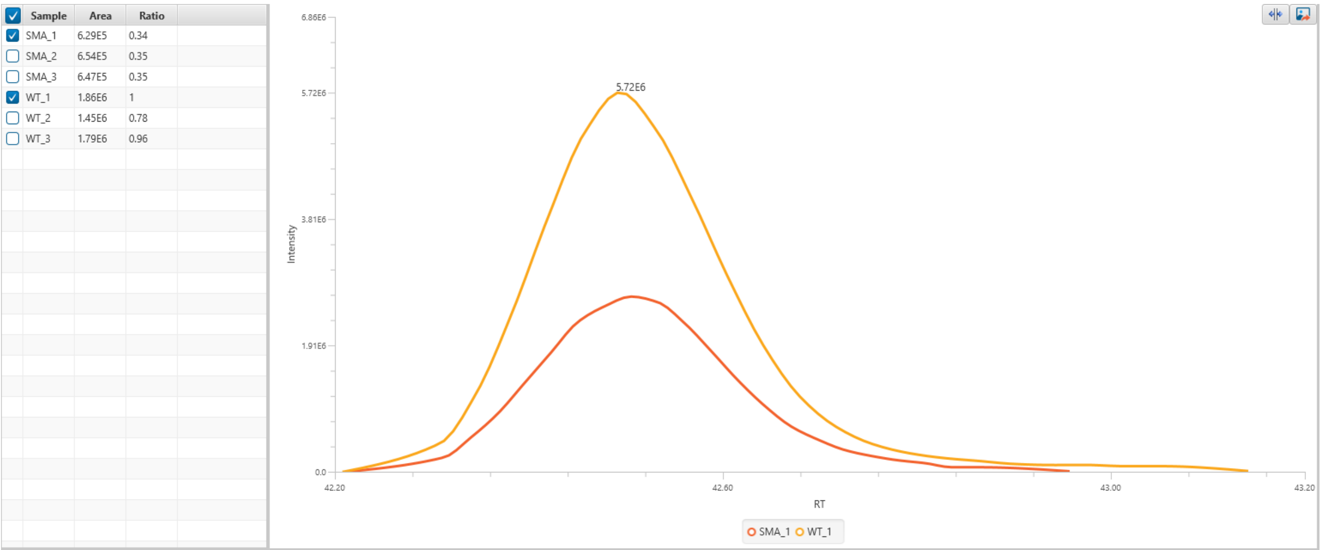

Sample	Area	Ratio
SMA_1	6.29E5	0.34
SMA_2	6.54E5	0.35
SMA_3	6.47E5	0.35
WT_1	1.86E6	1
WT_2	1.45E6	0.78
WT_3	1.79E6	0.96

Click on to switch between raw and aligned RT view.

Click on to export the XIC chart.

8.5.6 Sample Features

This tab displays a close up LC/MS view of the detected features from the selected samples. The feature boundary is shown by the bounding polygon. The center of the feature is denoted by a solid red dot on the monoisotopic peak cluster. Placing the cursor on this feature center displays a tool tip that shows the m/z value, the mean retention time, the charge state, and the intensity. The centers of the features are used to calculate the retention time alignment.

The button at the top-right corner of the tab can be used to toggle between the 2D and the 3D LC/MS views. To open a closed sample select the sample using the drop down found on the right hand side.

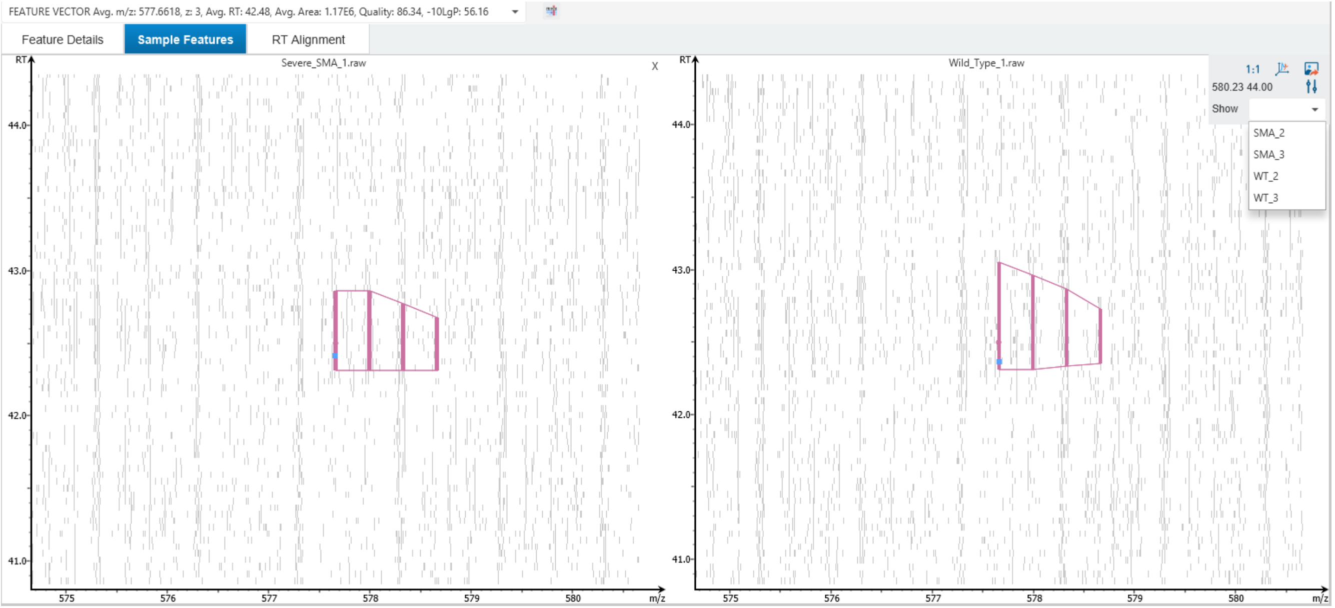

8.5.7 RT Alignment

This tab displays the aligned TIC curves of the selected samples with the highlighted retention time range of the selected feature. Use the toggle switch in the top-right corner to switch between the before and after RT alignment view. Click the button to view the pairwise RT alignment in the pop-up window. Select the sample pair from the drop-down menu to view the desired pairwise RT alignment.

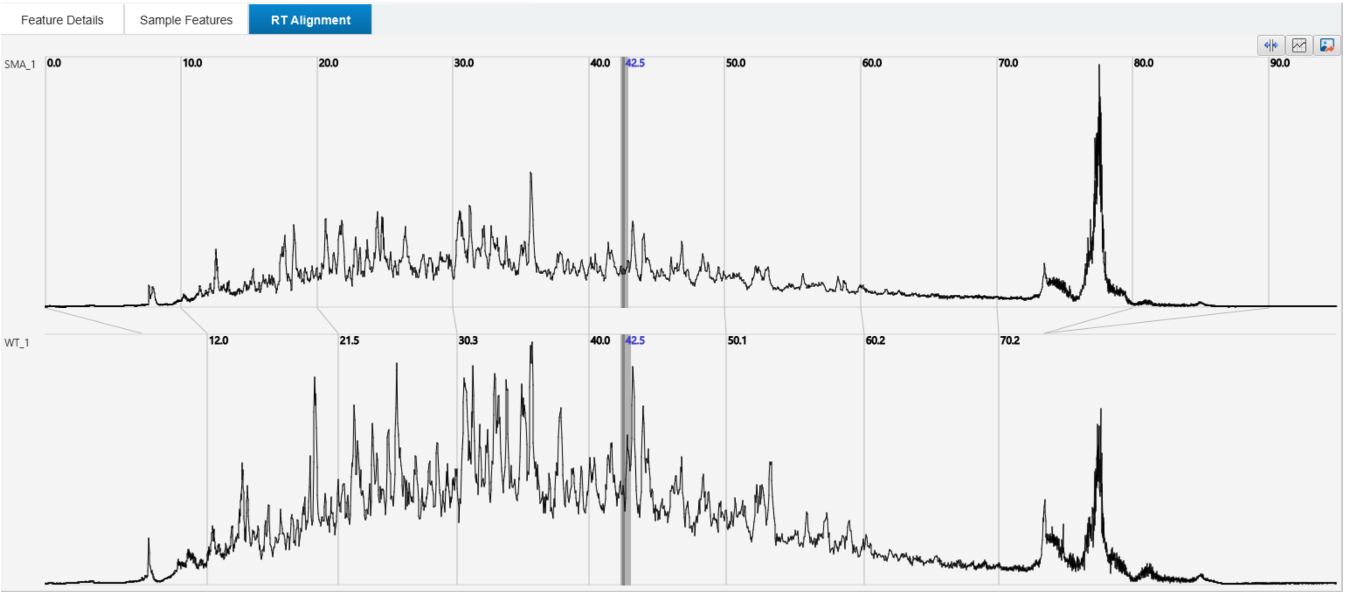

8.6 LFQ Exporting

In Project View, double-click on Export to open the Export node.

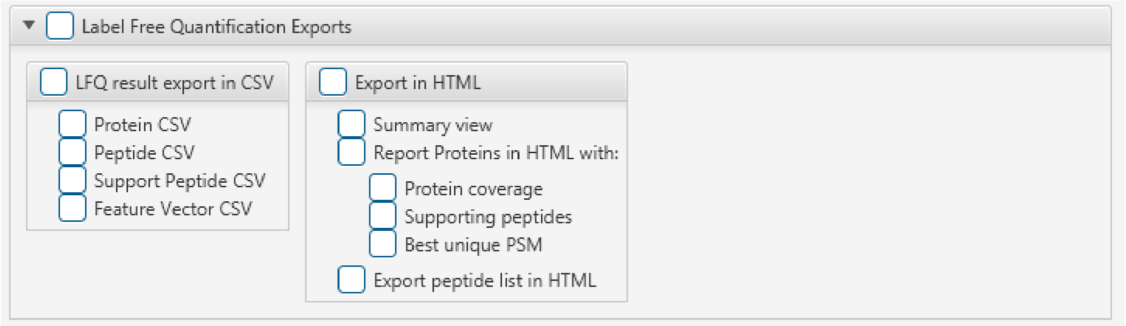

8.6.1 LFQ result - Exporting - comma separated values (CSV) format

There are several options for exporting the PEAKS Label Free Quantification results in CSV format:

- **Proteins CSV:** The list of protein quantifications, filtered by the protein filters in the Summary view and will be saved to proteins.csv.
- **Peptide CSV:** A complete list of all quantified peptides that pass the peptide feature filters set on the "Summary" page and their associated details.
- **Support peptides CSV:** These are the supporting peptides of the quantified proteins and their associated details.
- **Feature Vector CSV:** All predicted peptide features and their associated quantification information. Both identified and unidentified peptide features are included. To include unidentified peptide features, set the Peptide Id Count equal to 0 in the Peptide feature filter.

8.6.2 LFQ result - Exporting - HTML format

Export in HTML: This will generate single or multiple HTML reports in the specified location.

The following exporting options are available:

- **Summary view:** The “Summary” view page will be saved as a summary.html file in HTML format in the specified location.
- **Protein coverage:** The coverage pane will be saved for each protein.
- **Supporting peptides:** A list of supporting peptides will be saved for each protein.
- **Best unique PSM:** The best unique PSM will be saved for each protein.
- **Export peptide list in html:** creates a separate HTML-containing peptide identifications.

An individual protein will have its own HTML output file, where the corresponding protein coverage, the supporting peptides, and the best unique PSM are gathered.

9. Isobaric Labelling Quantification (TMT / iTRAQ)

Isobaric labelling quantification with chemical labels at the MS2 (or MS3) level is one of the three quantification modes that are supported by the optional PEAKS Q module of PEAKS Studio. In contrast to LFQ Quantification, TMT/iTRAQ Quantification is based on the relative intensities of the fragment peaks at fixed m/z values within an MS2 (or MS3) spectrum. To prepare for this, isobaric chemical labels are introduced into samples during the sample preparation stage. The samples are then combined and analyzed together in an LC-MS/MS experiment.

The same peptides from different samples will have the same precursor m/z and retention time, and are fragmented together. In the MS/MS (MS3) scans, labels from different samples will produce different reporter ions, which can then be used to calculate the quantification ratio between samples. Both user-defined and commercial labels (i.e. iTRAQ and TMT) are supported by PEAKS Q.

9.1 Isobaric Labelling Quantification Analysis Workflow and Settings

Select the PEAKS Q workflow in the Workflow Selection step

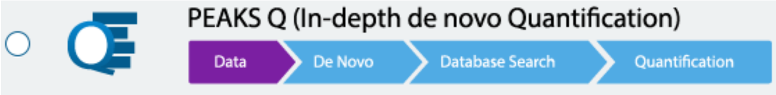

The following steps are the same as in the PEAKS DB workflow (as in section 6.1 Database Search Workflow and Parameter Settings) and is reiterated below.

Click on Data Refine to proceed through the workflow.

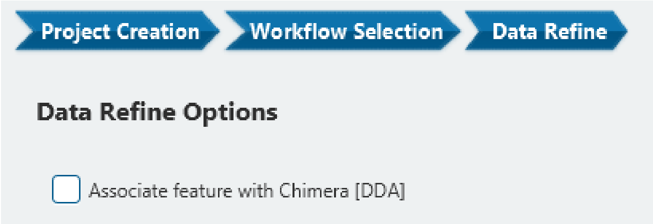

For Isobaric labelling quantification, it is recommended to **disable** the associate feature with Chimera option in Data Refine step. Proceed to the next step to see the DB Search parameters.

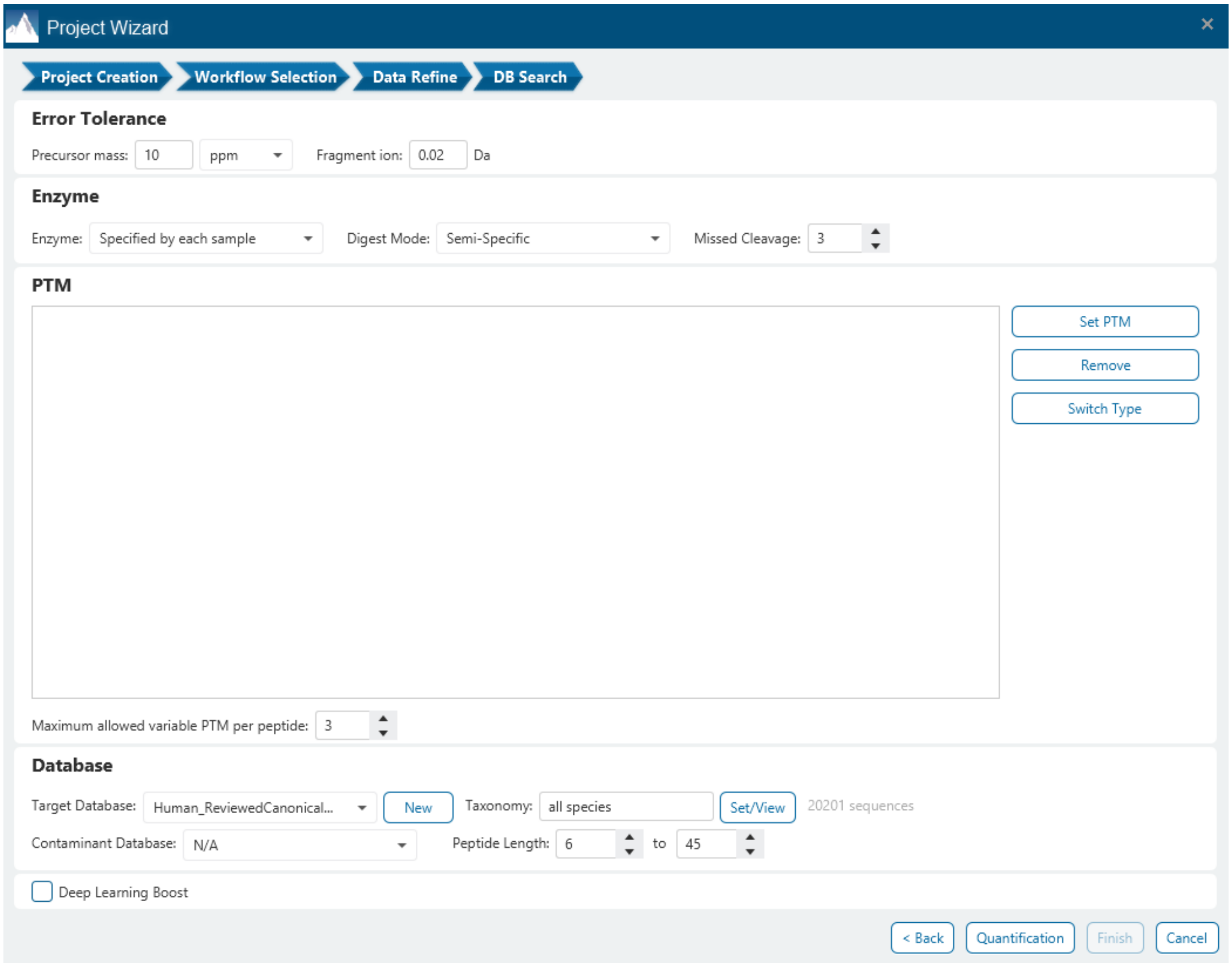

The following parameter settings are provided:

Precursor Mass Error Tolerance: The maximum mass difference between the peptide and the monoisotopic mass of the precursor ion.

Precursor Mass Tolerance unit: PEAKS generates peptides within the precursor mass error tolerance. Precursor mass error tolerance can be specified in either Daltons or ppm.

Fragment Mass Error Tolerance: PEAKS uses this value when scoring peptide sequences. PEAKS Studio considers a fragment ion to be matched if the calculated m/z is within the specified error tolerance. Units are Daltons.

Both precursor and fragment error tolerance parameters need to be set consistently with the mass accuracy of the instrument.

Enzyme Settings: PEAKS Studio digests the protein database *in silico* to generate peptide candidates. It is necessary to specify the enzyme for protein digestion from the Enzyme drop-down menu.

Note: When the selected dataset is digested with different enzymes, "Specified by each sample" allows samples to be analyzed separately using their respective enzymes specified during project creation.

Note: "None" is a special enzyme allowing non-specific cleavage at both ends of the peptide. It is the recommended option when no digestion enzyme was used or when the digestion enzyme exhibits a high degree of non-specificity.

Missed Cleavages: This specifies the number of missed cleavage sites allowed in a peptide.

Digest Mode: This specifies the type of in silico cleavages allowed at a peptide termini based on the method used for protein digestion. If "Specific" is selected, the specificity of the selected enzyme is strictly enforced at both termini of a peptide. "Semispecific" allows one terminus to disobey the enzyme specificity rules.

Note: If the enzyme is specified as "None", then no matter which mode is selected for "Digest Mode", the "None" enzyme digest rule will be applied.

PTM Settings: Click “Set PTM” to open the **PTM Options** dialog and specify the fixed PTMs and a few common variable PTMs expected in the experiment. Highlight PTMs and click on the 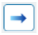 button to place them as either Fixed or Variable PTM. From there, highlighted PTMs can be removed by clicking the “Remove” button. Clicking “Remove All” will reset the PTM options. Highlighting and clicking “Switch Type” will move the Fixed PTM in into the Variable PTM list and vice-verse. Click on “New” to create a new custom PTM. While a PTM is selected, click on “View” to see more details.

Note: Multiple PTMs can be highlighted by holding down the CTRL key or SHIFT key on the keyboard.

Note: For a specific residue, only one fixed modification is allowed.

Once the PTMs are selected, the maximum number of variable PTMs per peptide can be specified and a value of less than 4 is recommended.

Note: The use of variable modifications increases the size of the computational search space for the de novo sequencing algorithm. It is recommended not to select too many variable modifications in PEAKS de novo. See PEAKS PTM for alternate search options.

Target Database: Select a protein sequence database for the search. Select one from the list of databases that have been configured in PEAKS and set the taxonomy by clicking on Set/View, if applicable. To configure a new database, click on New next to the Target Database dropdown to open the Database Configuration dialog.

Contaminant Database: To search for contaminants, select a contaminant database from the list of databases that have been configured in PEAKS. Keep the selection as N/A if no contaminant database is required for the search.

Peptide Length: The length for a peptide can be set when creating an analysis, where the default peptide length range is between 6 and 45.

Deep Learning Boost: Check this option to use PEAKS enhanced deeplearning algorithm to better identify PSMs in the DB Search process.

Click on Quantification to proceed to the next step and select the Reporter Ion Quantification (eg. iTRAQ/TMT) option.

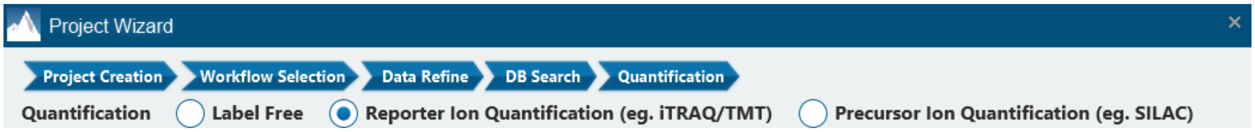

The following parameters are available:

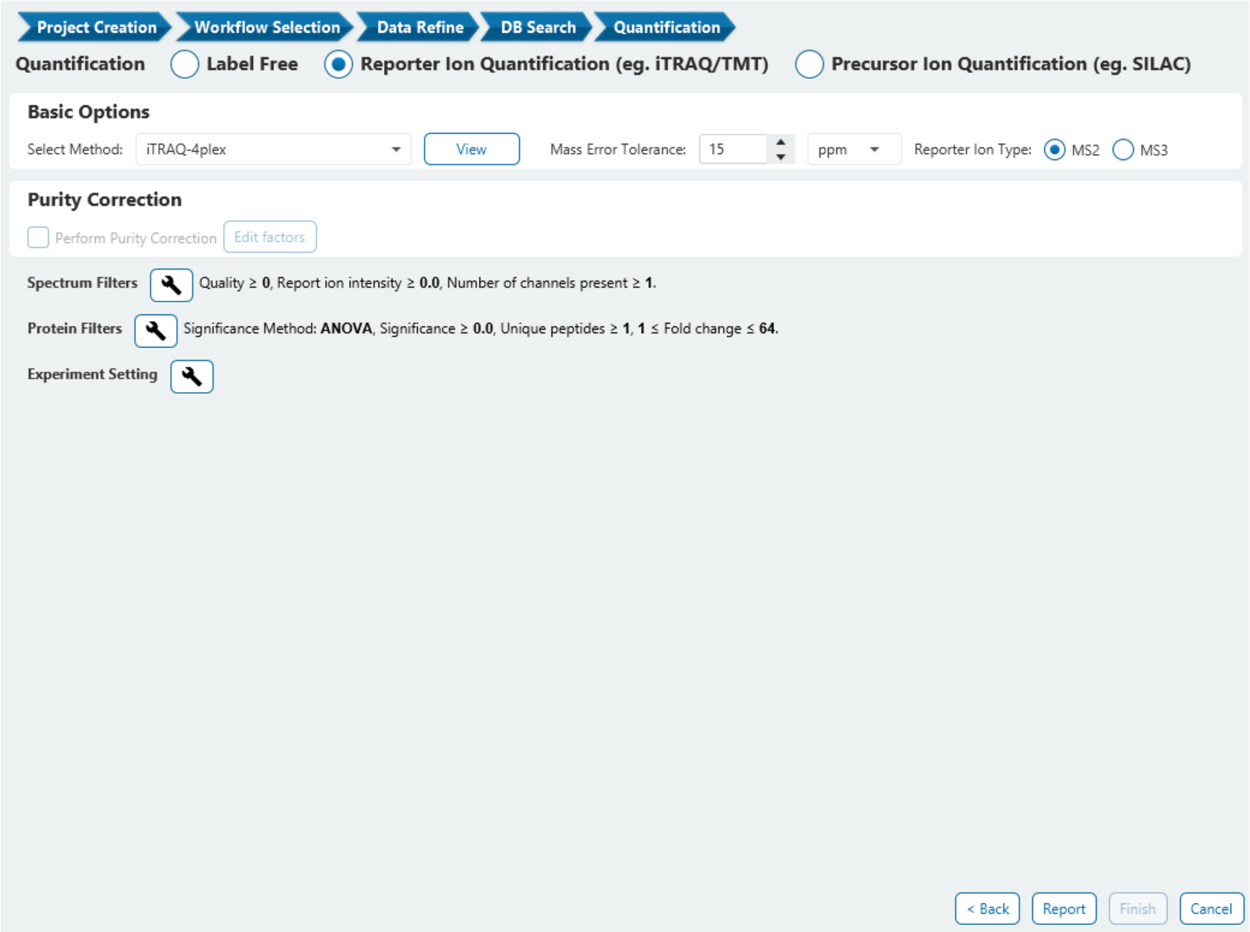

Select Methods: From the Select Methods drop-down menu, choose the appropriate quantification method used in the experiment, e.g. TMT-10plex

Mass Error Tolerance: This parameter is used to locate the reporter ion peaks in the MS/MS scan. Either Da or ppm can be selected as the mass unit.

Report Ion Type: This parameter allows for the selection of MS2 or MS3 as the reporter ion type for quantification.

Perform Purity Correction: Select the checkbox to edit correction factors for chemical labels (if applicable). Once the checkbox is selected, click the down arrow to open the dialogue that allows users to setup the purity correction. The dialogue is setup to mimic a Certificate of Analysis, where each factor within the table can be modified by double-clicking the particular cell of interest.

Click on the tool icon next to Spectrum Filters to open the Spectrum filter dialog.

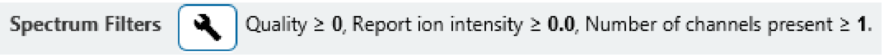

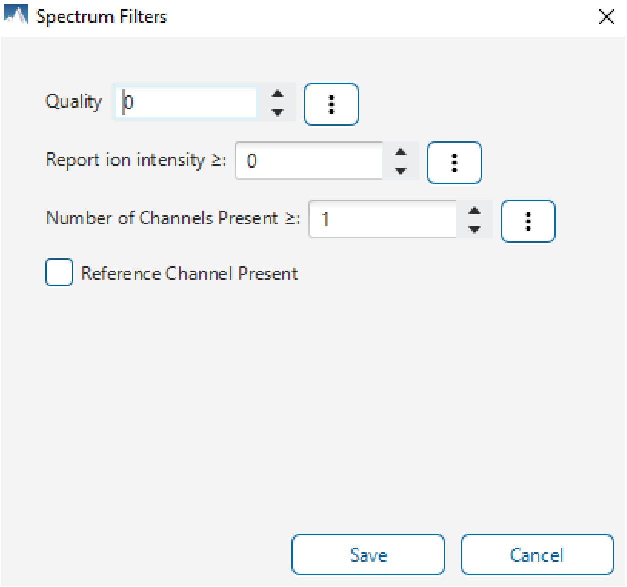

- **Quality:** A higher quality peptide indicates that the peptide is more quantifiable. Factors that affect the quality score include the identification -10LgP score, the noise around the reporter ions, and the mass error of reporter ions.
- **Reporter Ion Intensity:** Peptides with at least one reporter ion above this value will be kept.
- **Number of Channels Present:** Only quantifiable peptides that contain at least the number of labels selected will be used in protein quantification.
- **Reference Channel Present:** Only quantifiable peptides in which the reference label is present will be used in protein quantification. Note: The reference label is set in the experiment setting pop-up. See Experiment Setting section below

Click on the tool icon next to Protein Filters to open the Protein filter dialog

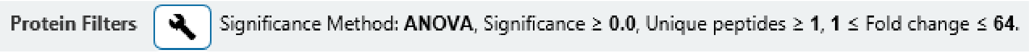

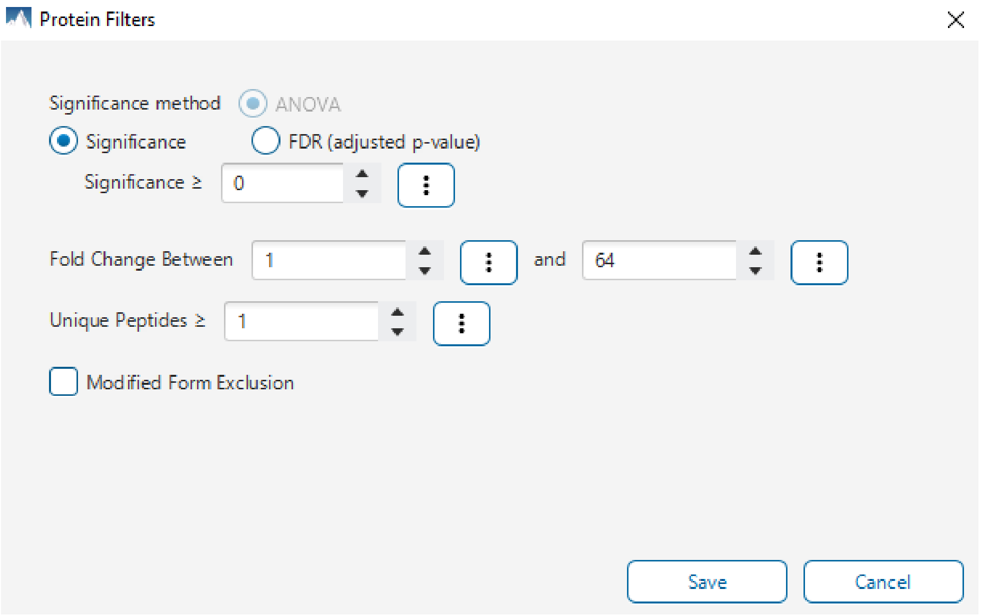

Modified Form Exclusion: Select the modified exclusion checkbox to exclude peptides with variable modifications from protein ratio calculation. Modified peptides have different ionization efficiency than unmodified ones, so this option allows users to exclude them to avoid this from having an effect on your quantification results.

Significance Method: ANOVA is the significance calculation that can be applied to the list of protein groups. Significance scores will only be calculated if each group contains at least two replicates. Lg (protein abundance) is used for the significance testing calculation. Please see reference below for further details.

Note: For more details on ANOVA, refer to the following paper: *"On the comparison of several mean values: an alternative approach ", Biometrika, 1951, 38(3/4): 330-336 .*

The other filters are the default filters set in the TMT/iTRAQ result Summary View. They can be modified later.

Significance: Only protein groups with a significance above this threshold will be listed in the “Protein” view. The significance score is calculated as the -10log of the significance testing p-value (e.g. Significance score of 20 is equivalent to a p- value of 0.05). PEAKS provides the significance testing method: ANOVA.

FDR: Adopt the Benjamin-Hochberg method to adjust the p-value to the false discovery rate for all protein groups that have already passed the other filters. Only protein groups with significance scores passing the calculated FDR will be listed in the "Protein" view. Either this or "Significance" can be selected to set a significance threshold.

Fold Change: Only protein groups below the lower threshold or above the upper threshold value will be listed in the "Protein" view.

Unique Peptides: Only protein groups that were quantified with this number of peptides (or more) will be listed in the "Protein" view.

Click on the tool icon next to Experiment Setting to open the Experiment Setting dialog.

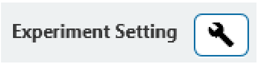

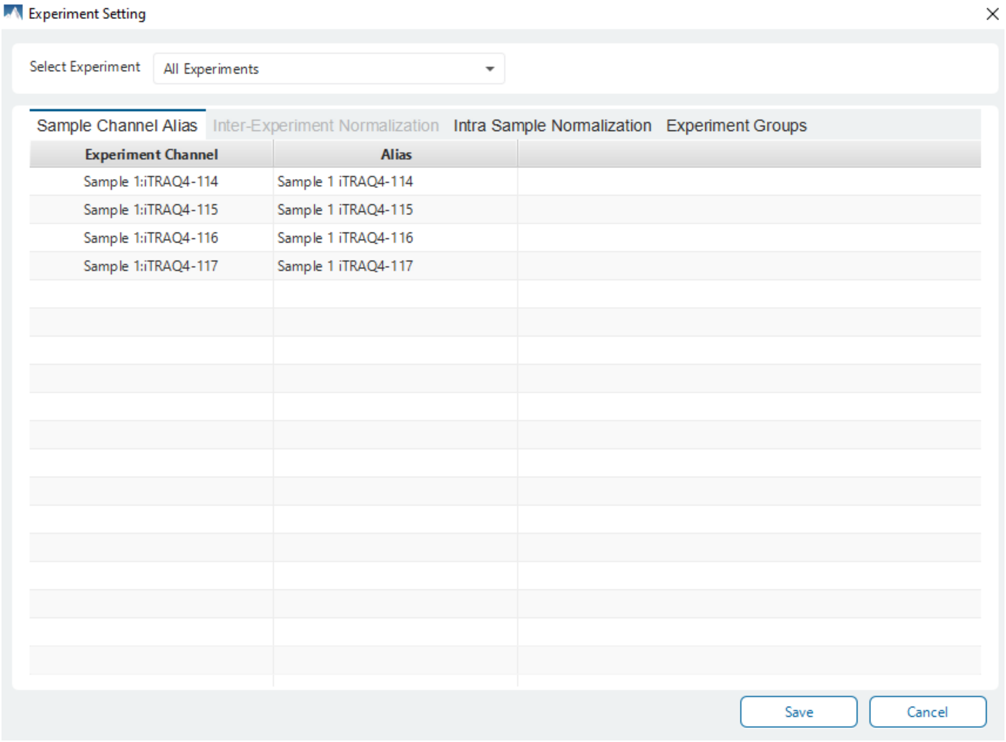

Select Experiment: When running quantification on an identification node that searched data within multiple samples (i.e., replicate experiments within different treatments), this drop-down menu allows for the selection of a single sample or every sample (All Experiments) for analysis. Every label of the sample(s) selected will appear in the screen located below the search bar.

Setting Sample Channel Alias: The name of the Sample Channel can be changed by using the alias name.

When there are two or more samples, the Inter-Experiment Normalization tab is enabled.

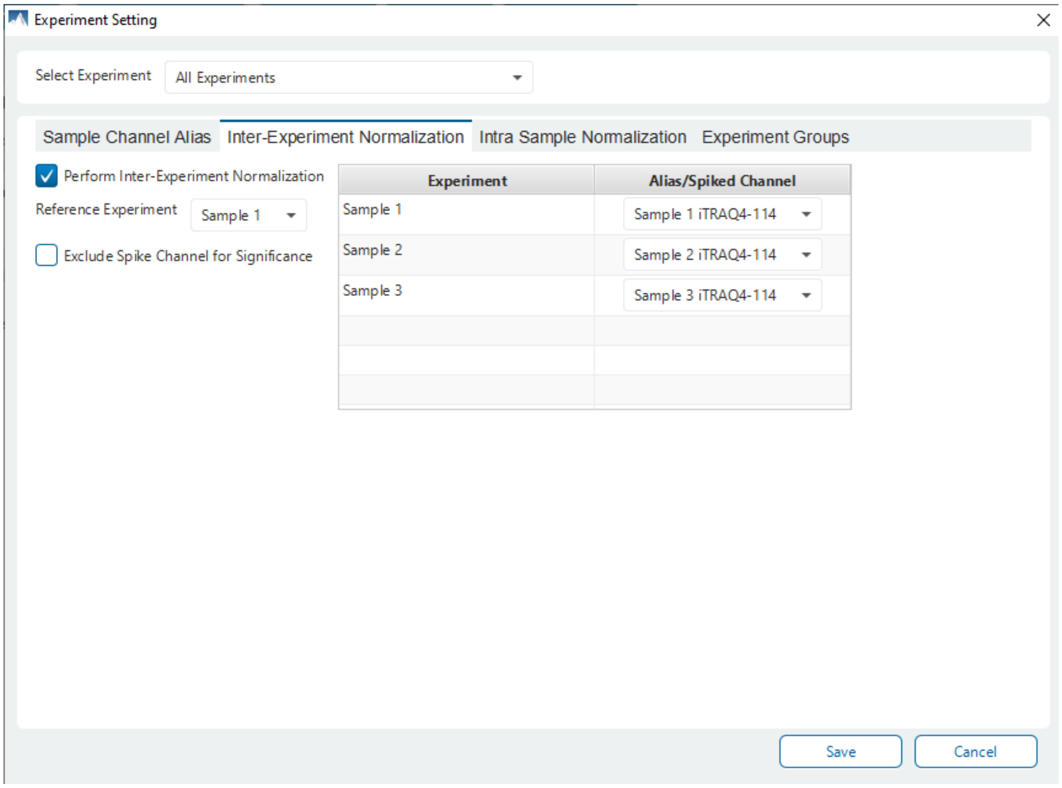

Perform Inter-Experiment Normalization: Check the Perform Inter-Experiment Normalization box if a spike label or normalization channel was used in the experiment. This is used to enable quantification between experiments.

Alias/Spike Channel Drop-down Menus: Select the channel in each sample that will be used for normalization. The software assumes that similar peptide features containing this label across experiments will approach a 1:1 ratio. Differences are assumed to be due to systematic error and all labeled channels are normalized to this spike label.

Exclude Spike Label for Significance: Since the spike label is assumed to be in a 1:1 ratio between experiments, it should not be included in the significance calculation.

Click on Intra Sample Normalization to switch the tab for intra-sample normalization.

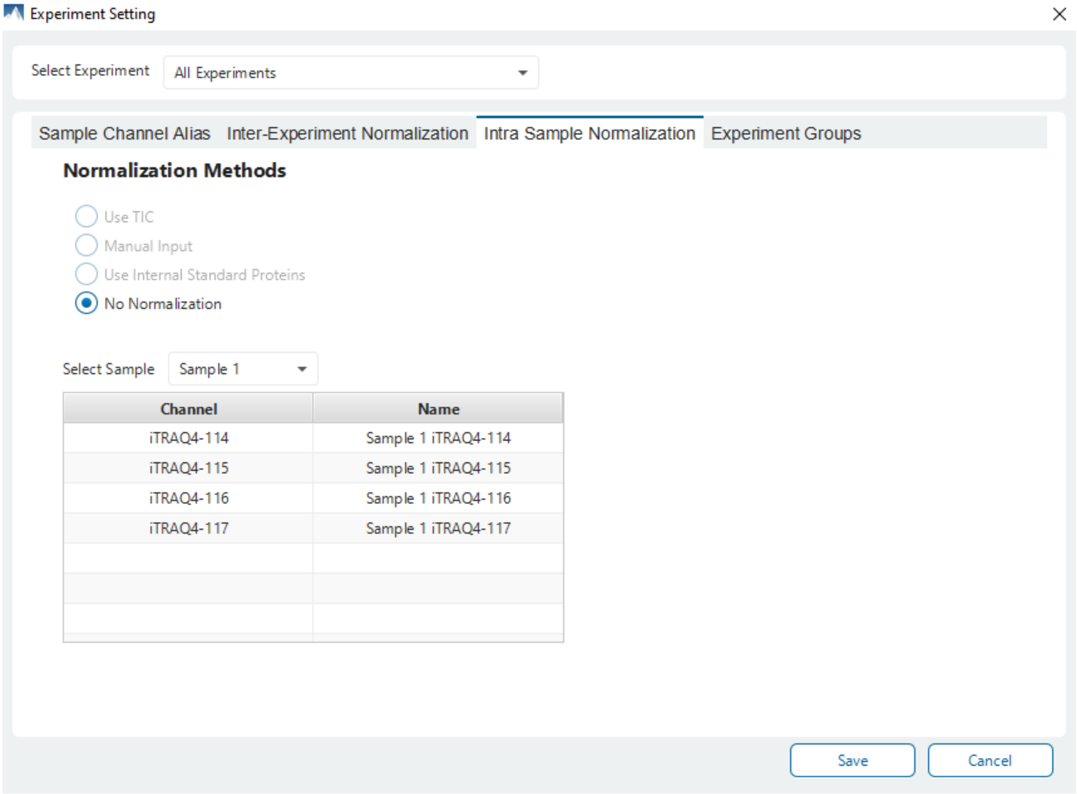

No Normalization: If there are multiple samples in the quantification run and all samples are selected, No Normalization is chosen as the default option. It will be the only normalization option available for selection unless "Perform Inter Experiment Normalization" is check-marked in Experiment Settings.

Auto normalization: This option will calculate a global ratio from the total intensity of all labels in all quantifiable peptides. Factor values and Expected Ratios between the channels (i.e., the labels) will be presented. Selecting another sample from the Experiment drop-down menu will display the results of other samples.

Manual input: This option performs the same default calculation as Auto normalization but also allows for the manual change of each label's Expected Ratios. After changing a ratio and hitting “Enter” on the keyboard, PEAKS will recalculate the Factor for the non-referenced label(s). The Experiment drop-down menu can be opened to display the results of another sample.

Use Internal Standard Proteins: This option displays a list of identified proteins that can be selected to create a normalization ratio. Use the search bar to quickly locate specific proteins. Right-clicking in the Protein list will open a pop-up menu to select all highlighted rows or remove all selected proteins instead. The expected ratios can be manually modified. It is assumed that the total protein content of the selected proteins in different labels have the ratios entered in the ‘Expected ratio’ column. If desired, sample names can be changed by double-clicking the chosen channel below the “Name” column and typing the new name.

Click on Experiment Groups to switch the tab and set Experiment Grouping.

Experiment Group: TMT/iTRAQ requires sample grouping. All available channels are listed in the left window. After selecting a channel, it can be added to a new group by clicking the 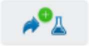 button or to an existing group by

clicking the 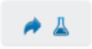 button and selecting that option from the drop-down menu. If you select multiple channels, you can add each one individually to create multiple new groups by clicking the 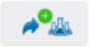 button. Channels and Groups can be removed by clicking the 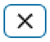 button next to each element. The names and colours for each group can be edited by clicking on the coloured square dropdown, respectively.

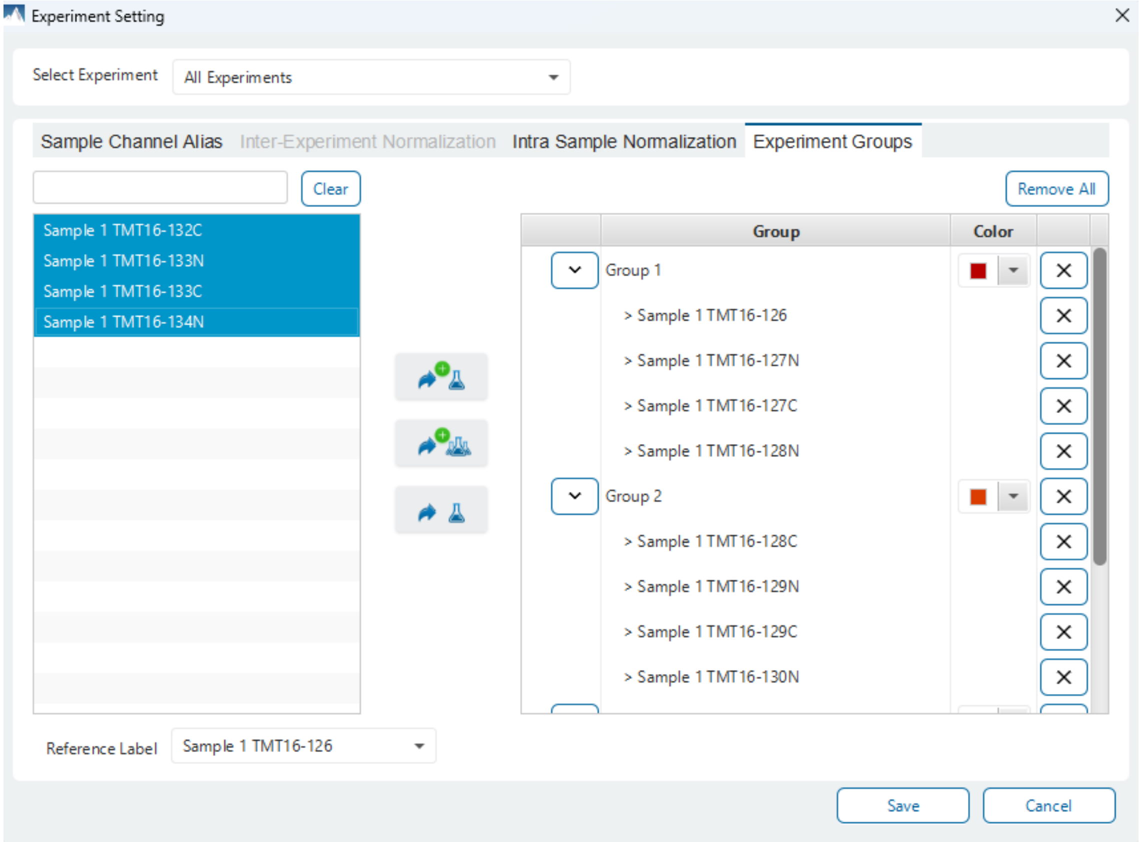

Note: If no experiment groups are set, PEAKS Studio will automatically set each channel in their own group. There will be a warning pop-up to give notice of this.

Click on “Report” to proceed to the final step. Report filters can be set here which will filter the final result.

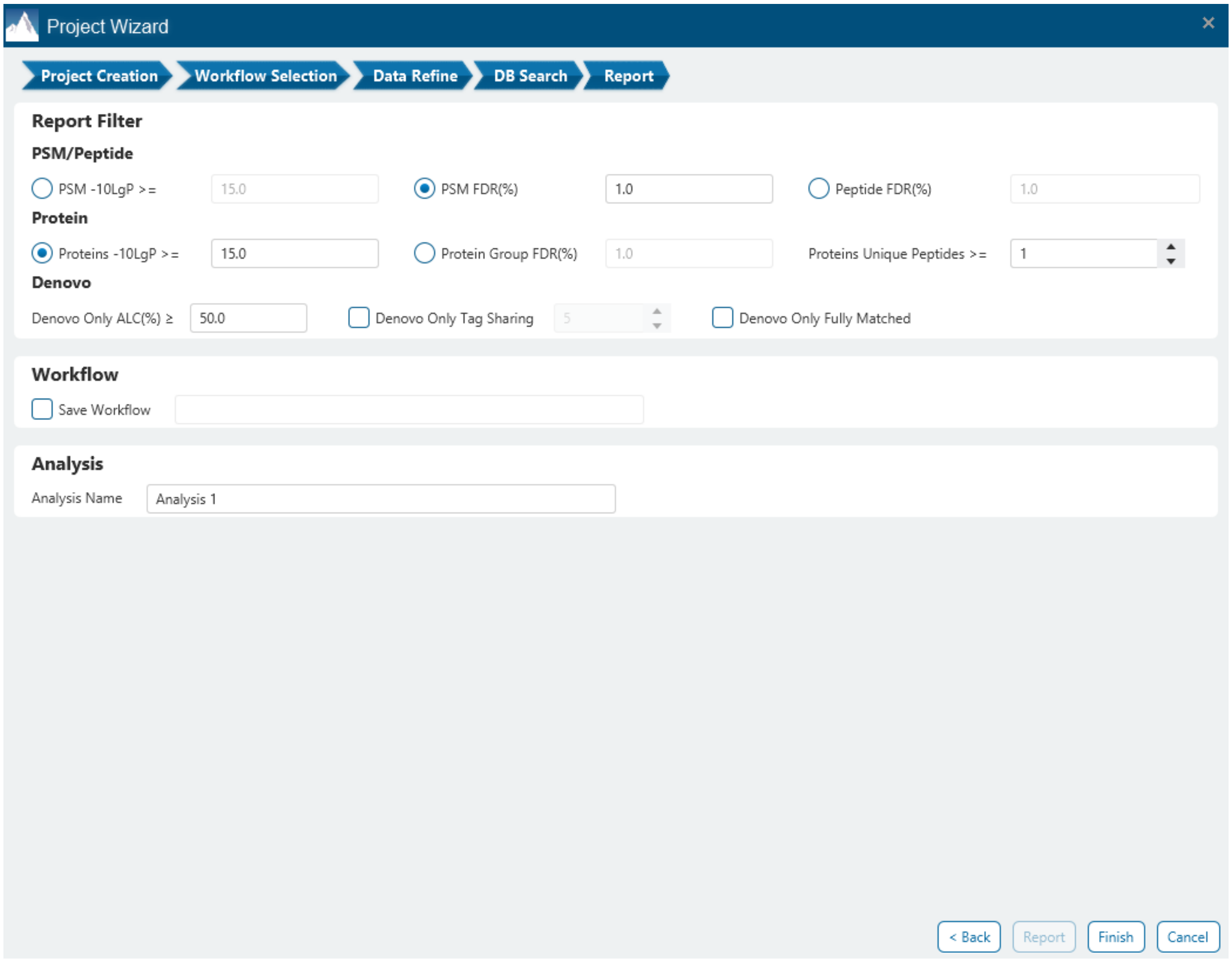

These filters are provided:

PSM/Peptide: this section is a mandatory section to filter out some results under the user-defined filter. Any changes to the filter will create a new analysis with old parameters and update the results and statistics.

PSM -10lgP: The PEAKS peptide score (-10lgP) is calculated for every peptide-spectrum match (PSM) reported by PEAKS DB, PEAKS PTM, and SPIDER. The score is derived from the p-value that indicates the statistical significance of the peptide-spectrum match. A peptide may be matched to many spectra, resulting in multiple PSMs. In that case, the peptide's score is calculated as the maximum among all PSM scores. For details of the scoring algorithm, please refer to the publication, *"PEAKS DB: De Novo sequencing assisted database search for sensitive and accurate peptide identification ", Mol Cell Proteomics, 2011 Dec 20.* A minimum requirement can be set and all identifications must pass this filter.

PSM FDR (%): The PSM filter can also be set to use the false discovery rate. FDR is estimated using a "decoy-fusion" approach.

Peptide FDR (%): The Peptides can also be set to use the false discovery rate. Peptides are scored based on their top PSM and using a “decoy-fusion” approach, the result is reported at that FDR.

Note: Decoy fusion is an enhanced target-decoy method for result validation with FDR. Decoy fusion appends a decoy sequence to each protein as the "negative control" for the search. See BSI's web tutorial (http://www.bioinfor.com/fdr-tutorial/) for more details.

De Novo ALC (%): This filter controls the minimum ALC score required for display in the de novo only result. A default of 50% is set.

Denovo Only Tag Sharing: The number set here corresponds to the length of de novo only tags to be shown in the result Protein coverage. For de novo only peptides that are not fully matched, they must share a tag length with of minimum of this parameter with the protein residue to be displayed.

Denovo Only Fully Matched: Checking this option will show the de novo only peptides in the Protein coverage regardless of what tag sharing parameter is set if the entire de novo only sequence matches the protein residue.

In the Report step, there also is the option to specify an Analysis Name (which can be later renamed) and/or decide to save the Workflow to quickly re-use in future analyses.

9.2 Understanding TMT/iTRAQ Results

Once completed, a TMT/iTRAQ result node will be added to the project tree. Double-click on this 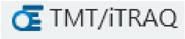

node to open the Isoberic Labelling Quantification result that contains three tabs as described below:

Summary: The outline of the isobaric labelling search results with statistics. This is the place to examine the overall performance of the experiment and adjust filters. If there are too many proteins, the heatmap will not show on the summary page.

Proteins: The quantified proteins with a list of supporting peptide features for each protein.

Peptides: The quantified peptides are listed in a table. Extracted ion chromatogram (XIC) and supporting peptide features are also displayed for each peptide.

9.3. TMT/iTRAQ - Summary View

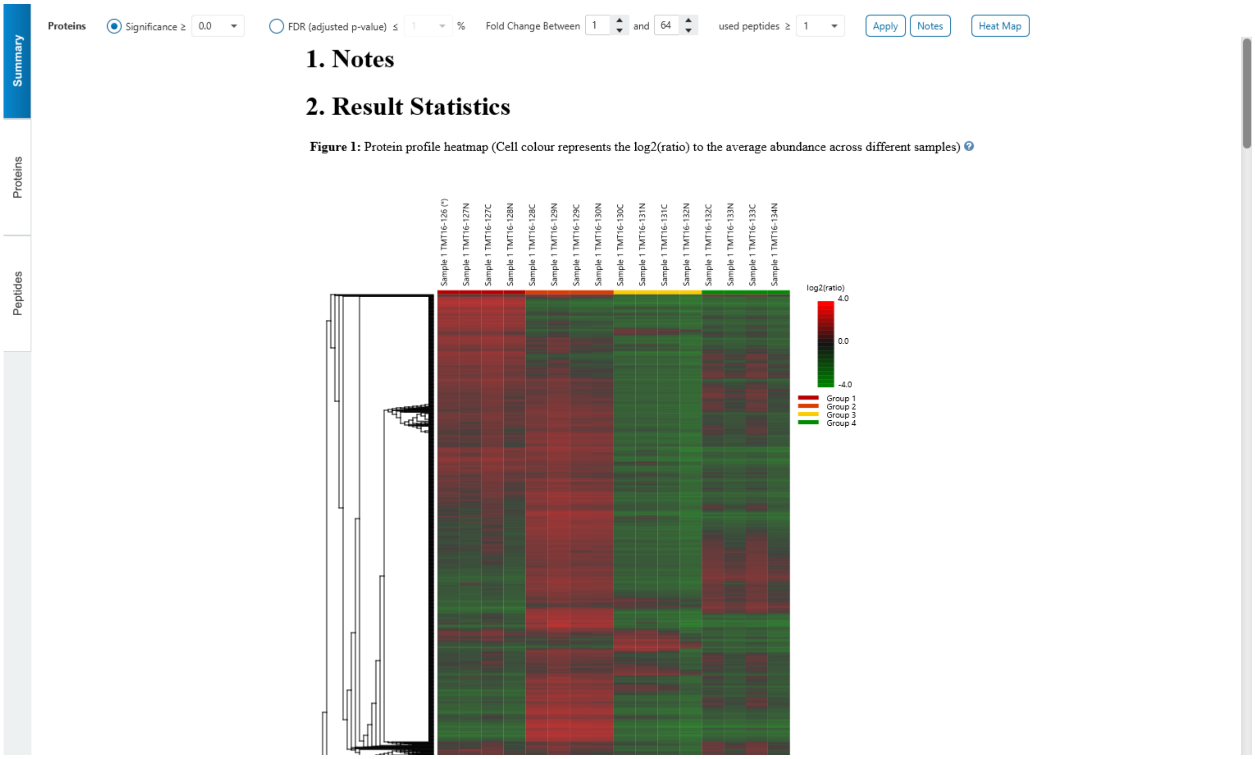

9.3.1 Summary View - Protein Filters

Filter settings used in the screenshot above means that no protein filter is applied, thus giving all quantifiable protein.

Alternately, parameters can be adjusted for additional analysis by updating the protein filters above.

Significance: Only protein groups with a significance above this threshold will be listed in the **Protein View**. The significance score is calculated as the -10log10 of the significance testing p-value. PEAKS provides ANOVA significance testing methods A significance score threshold of 20 is recommended, which equals to a significance testing p value of 0.01. Either this or “FDR (adjusted p-value)” can be selected to set a significance threshold.

Fold Change: Only proteins at or above this fold change threshold will be listed in the Protein View.

Used Peptides: Only proteins with at least this many number of used peptides will be listed in the Protein View.

Click on Apply to apply these filters changes to the result. This will update charts on the Summary View and will update the Protein table accordingly.

9.3.2 Summary View - Notes

Click on Notes to open the Notes dialog and save information about the result and saved in section 1. Notes on the Summary page.

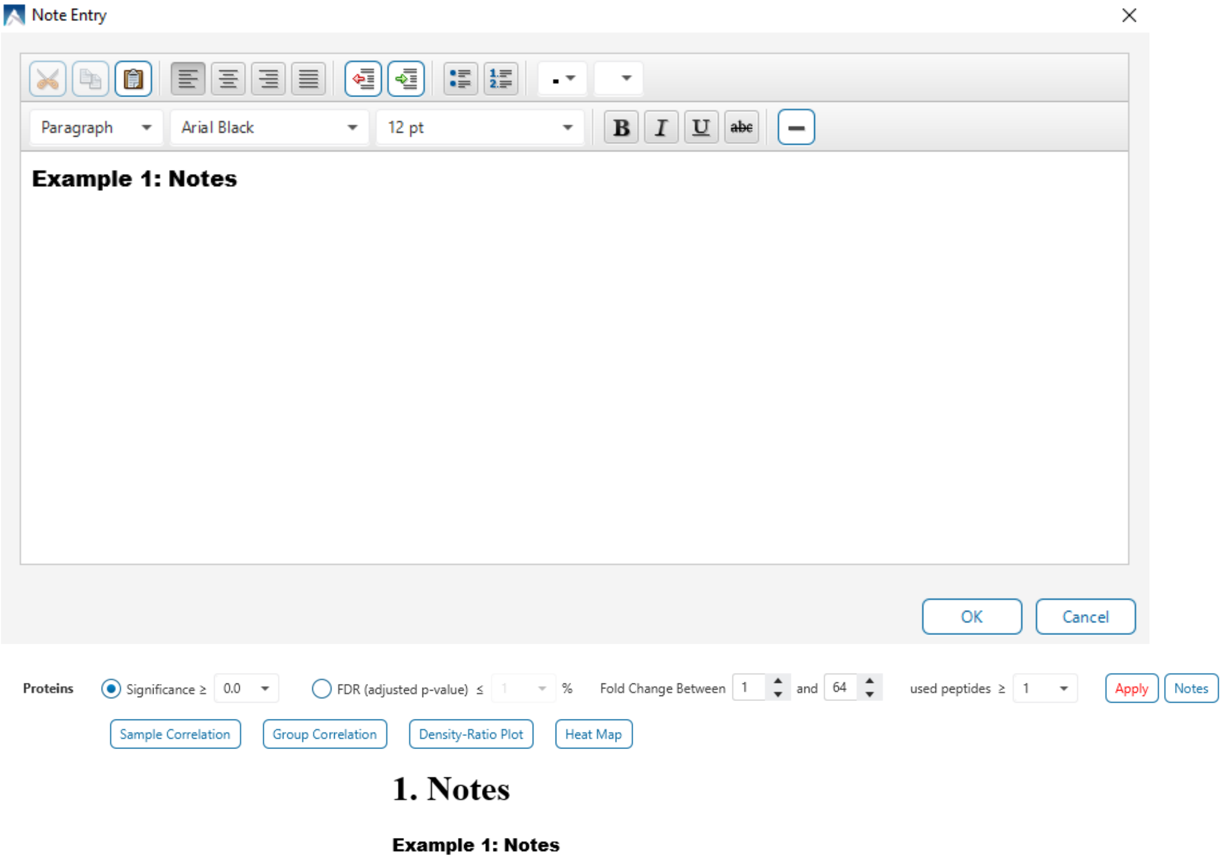
Note Entry
Paragraph
Arial Black
12 pt
Example 1: Notes
OK
Cancel
Proteins
Significance ≥ 0.0
FDR (adjusted p-value) ≤ 1 %
Fold Change Between 1 and 64
used peptides ≥ 1
Apply
Notes
Sample Correlation
Group Correlation
Density-Ratio Plot
Heat Map
1. Notes
Example 1: Notes

9.3.3 Summary View - Heat Map

Figure 1: Protein profile heatmap (Cell colour represents the log2(ratio) to the average abundance across different samples)

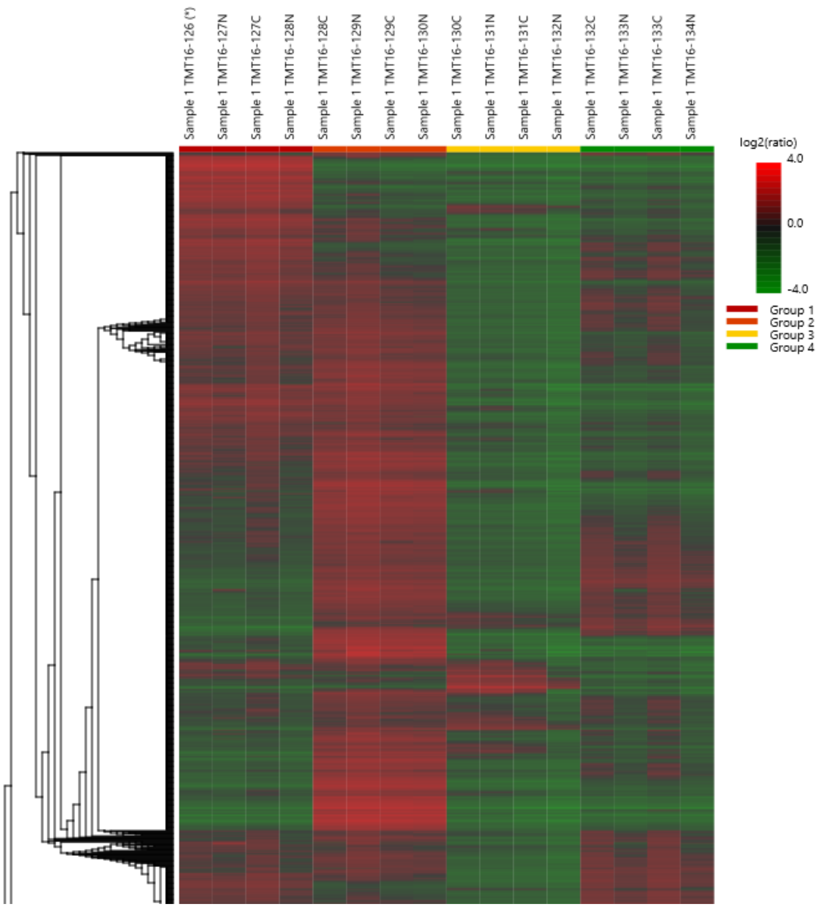

The heat map displays the protein groups that passed the filters. The relative protein abundance is represented as a heat map of the representative proteins of each protein group. These representative proteins are clustered if they exhibit a similar expression trend across the samples. The hierarchical clustering is generated using a neighbour-joining algorithm with a Euclidean distance similarity measurement of the log2 ratios of the abundance of each sample relative to the average abundance. Similarly, the conditions in different samples are clustered if they exhibit a similar expression trend across the protein groups. An interactive protein profile heat map is opened in a new window when the Heat Map button is clicked options.

Mouse over the dendogram in a section of the heatmap and click on it to open the Variation Trend chart.

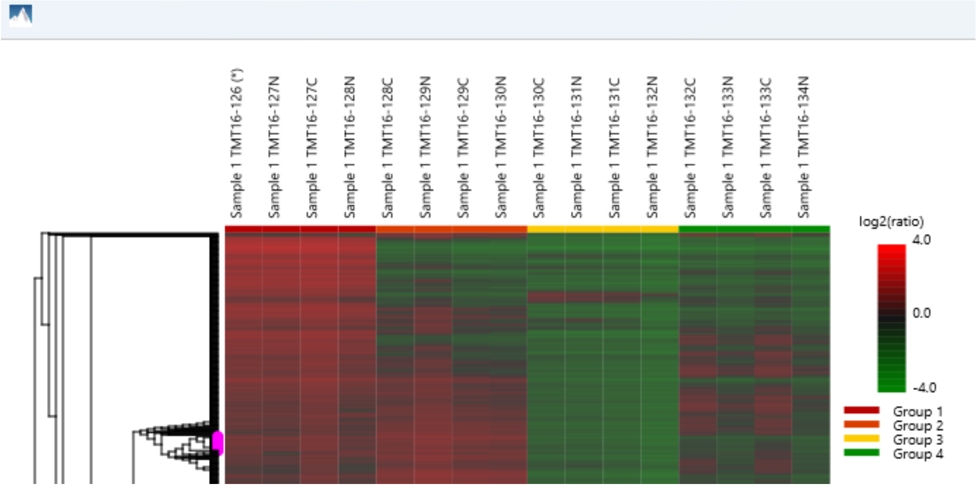

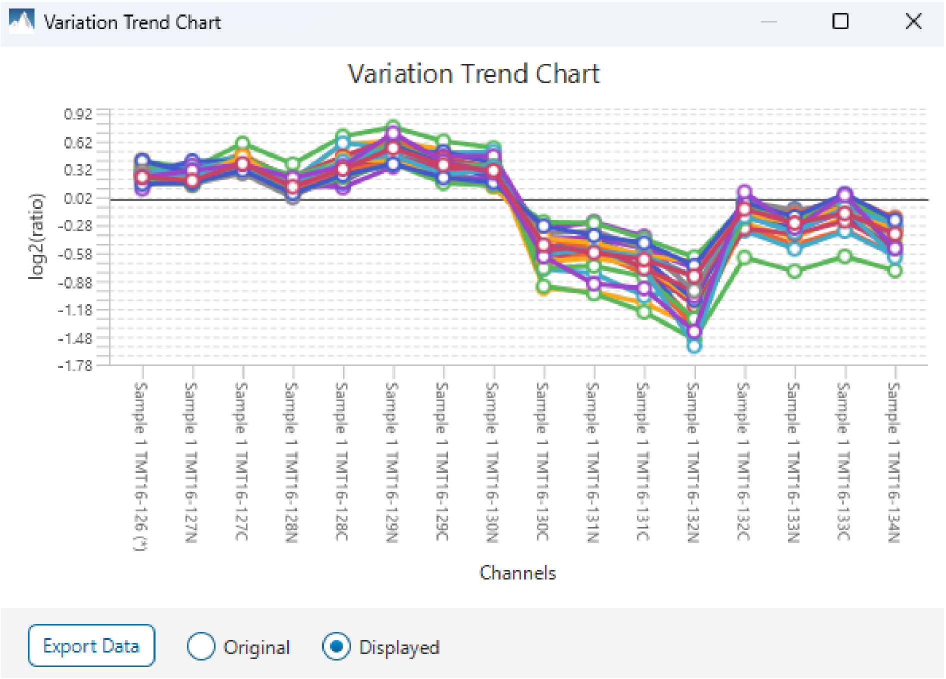

The Variation Trend chart displays the expression level of the selected proteins in the samples.

Mouse over points on the variation trend chart to see a tooltip of the protein, sample, and log2ratio.

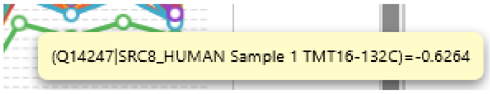

Click on the Export Data button to export the variation trend chart data in a text file format. When Displayed is selected, the log2 ratios will be in the export. When Original is selected, the original ratio values will be in the export instead.

9.3.4 Summary View - Volcano plot

Figure 2. volcano plot combines a statistical test with the magnitude of the change enabling quick visual identification of those data-points that display large-magnitude changes and that are also statistically significant. The volcano plot in Figure 2(a) plots significance versus fold-change of the quantified proteins.

Horizontal broken grey line: The selected significance threshold

Vertical broken grey lines: The selected fold change thresholds

Plotting points in this way results in two regions of interest in the plot: those points that are found towards the top of the plot that are far to either the left or the right side. These represent values that display large magnitude fold changes (hence being left or right of center) as well as high statistical significance (hence being towards the top).

Figure 2. The volcano plot for proteins.

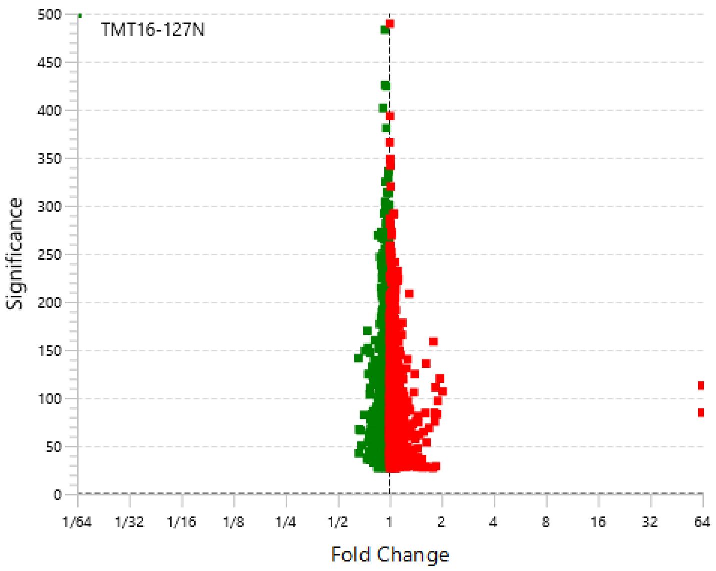

In this figure, the ratio is the group ratio set with respect to the base group. If more than two groups are present, the max ratio is used instead.

The Protein View contains an interactive volcano plot that can be hidden or displayed. Markers for the proteins that are above the set significance threshold will be displayed in colour. Scrolling the cursor over a marker will highlight it and, subsequently, clicking on this marker will highlight the protein in the interface.

9.3.5 Summary View - Filtration parameters and statistics

Tables 1 and 2 list the filtration parameters and filtered results, respectively.

Table 1. Result filtration parameters.

Significance	≥ 50
Min Fold Change	≥ 2
Max Fold Change	≤ 64
Used peptides	≥ 1

Table 2. Statistics of filtered result.

Peptide-Spectrum Matches	23905
All Peptide sequences	6872
Protein groups	763

9.3.6 Summary View - Search parameters

Table 3 lists the parameters as set in the workflow.

Table 3. Search Parameters

Search Engine Name: PEAKS
Reporter Ion Quantification method: TMT16PLEX
Mass Error Tolerance: 15.00 ppm
Reporter Ion type: MS2
Perform Purity Correction: No
Spectrum Filters:
  Quality ≥ 0
  Reporter Ion Intensity ≥ 0.0
  Number of channels present ≥ 1
  Reference Channel Present: No
Protein Filters:
  Significance method: ANOVA
  Significance ≥ 0.0
  Fold change between: [1.0 - 64.0]
  Unique peptides ≥ 1
Select Experiment: All Experiments
Perform Inter-Experiment Normalization: Yes
Exclude Spike Channel for Significance: No
Normalization method: No Normalization
Groups:
  Group 1:
    Sample 1 TMT16-126
    Sample 1 TMT16-127N
    Sample 1 TMT16-127C
    Sample 1 TMT16-128N
  Group 2:
    Sample 1 TMT16-128C
    Sample 1 TMT16-129N
    Sample 1 TMT16-129C
    Sample 1 TMT16-130N
  Group 3:
    Sample 1 TMT16-130C
    Sample 1 TMT16-131N
    Sample 1 TMT16-131C
    Sample 1 TMT16-132N
  Group 4:
    Sample 1 TMT16-132C
    Sample 1 TMT16-133N
    Sample 1 TMT16-133C
    Sample 1 TMT16-134N
Reference Label: Sample 1 TMT16-126

9.4 TMT/iTRAQ - Protein View

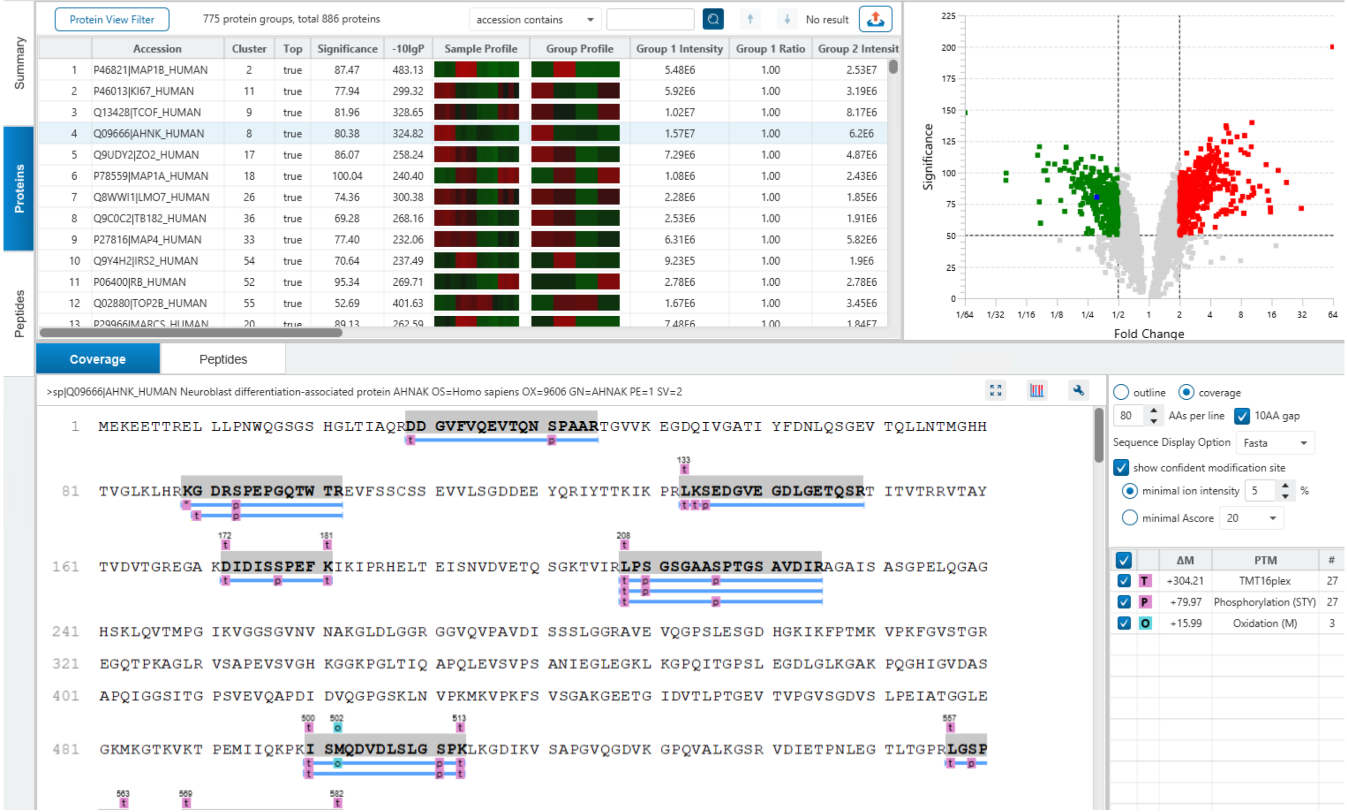

The **Proteins View** lists all the quantified proteins present in the sample, characterizes each protein at the amino acid level, and lists the supporting peptide features of each protein. It has six components:

Protein Table: Lists all the quantified proteins present in the sample.

Protein View Filters: Allows filtering the protein table to select specific proteins.

Protein Volcano Plot: Click on a marker that is above the significance threshold (the horizontal dashed line) set in the Summary View. Upon selection, the corresponding protein in the Protein Table will be highlighted.

PTM Profiling: Click on 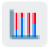 to open PTM profiling.

Coverage: Characterizes the protein sequence at the amino acid level. All the PTMs occurred on the protein sequence will be displayed together with the MS/MS data supporting the inference.

Peptides: Lists the supporting peptides identified from the selected proteins in the protein table.

9.4.1 Proteins View - Protein Table controls

At the top of the Proteins View, there are the options to filter, search, and export the protein table.

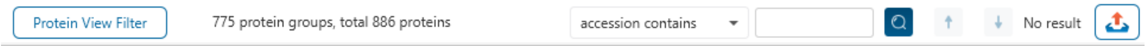

9.4.1.1 Protein Table controls - Protein View Filters

Click on the Protein View Filter button to open the Protein View Filter.

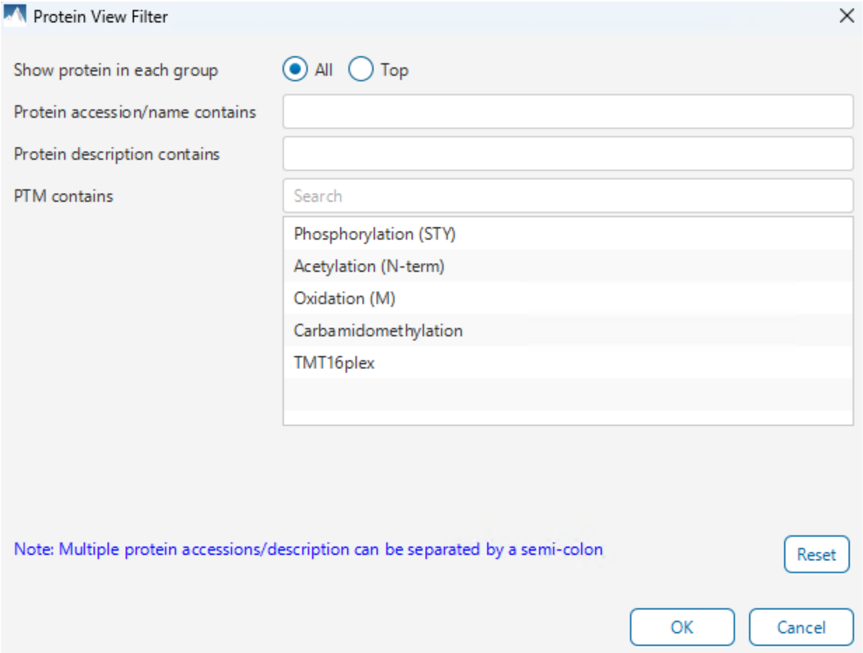

The following options are available for filtering the protein table:

Show All or Top proteins in each group: Adjust the protein list based on Protein grouping, top is selected by default. Proteins are grouped based on parsimony.

- **Top:** Show only the top proteins in each group. These proteins are supported by the most unique peptides in the group. Proteins in the group that share a subset of the unique peptides that support the top protein (subproteins) will not be displayed.
- **All:** Show all proteins in each group. Proteins are grouped together if they are supported by the same set or a sub-set of the top protein in the group. If a protein is supported by a peptide not supporting the top protein it will be added to a new group.

Protein accession/name contains: Restrict the protein table to only display protein IDs which contain the entered text in its accession/name.

Protein description contains: Only proteins with descriptions that contain the entered text will be included in the protein table if text is entered in this field.

> **Note:** In these above two filters, multiple entries can be separated by a semi-colon.

PTM contains: Filter protein results by the presence of selected PTMs. Proteins that are supported by peptides containing modifications that match the selected PTMs will be included. If multiple PTMs match the modification name, they will all be included in the table. For SPIDER results, the mutations will also be listed in the PTM contains filter.

Click on the Reset button to remove all filters.

Click on OK to apply the Protein View Filter to the Protein table.

Click on 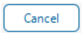 to discard changes and close the dialog.

9.4.1.2 Protein Table controls - Protein count

At the top of the protein table shows the count of protein groups and number of proteins in the table based on the current Protein View Filters.

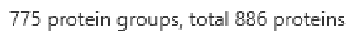

9.4.1.3 Protein Table controls - Protein Table Search Function

An easy-to-use search function is available for the protein table. The protein accession or description substring can be searched for, and all search results can be navigated through with the Up and Down arrows.

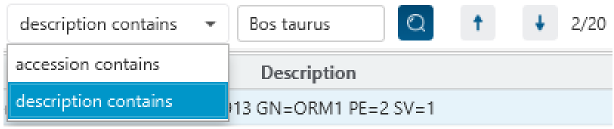

Protein accession/name contains: Restrict the protein table to only display protein IDs which contain the entered text in its accession/name.

Protein description contains: Only proteins with descriptions that contain the entered text will be included in the protein table if text is entered in this field.

9.4.1.4 Protein Table controls - Protein Table Export

Click on the 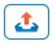 button to export the contents of the current table. This considers the Protein View Filters, the sorting applied to the table and is consistent with the view. There is the option to export the Protein table and/or the Supporting peptide table in CSV format.

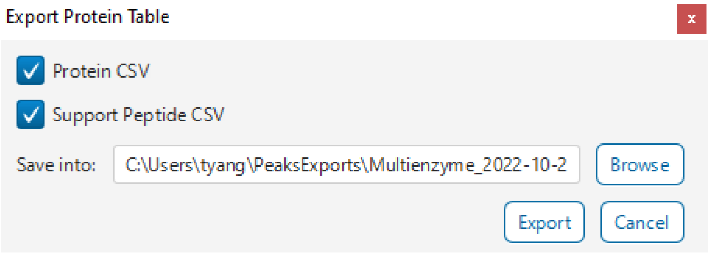

9.4.2 Proteins View - Protein Table

Each row in the table represents a group of proteins that are supported by a common set of peptides. To expand the group, click the right arrow button at the left.

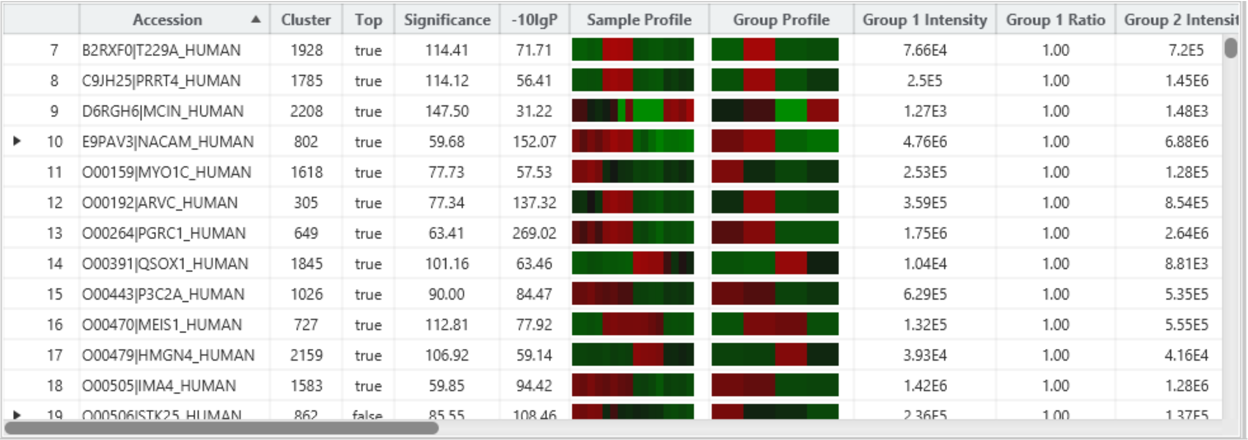

	Accession	Cluster	Top	Significance	-10lgP	Sample Profile	Group Profile	Group 1 Intensity	Group 1 Ratio	Group 2 Intensit
7	B2RXF0|T229A_HUMAN	1928	true	114.41	71.71			7.66E4	1.00	7.2E5
8	C9JH25|PRRT4_HUMAN	1785	true	114.12	56.41			2.5E5	1.00	1.45E6
9	D6RGH6|MCIN_HUMAN	2208	true	147.50	31.22			1.27E3	1.00	1.48E3
10	E9PAV3|NACAM_HUMAN	802	true	59.68	152.07			4.76E6	1.00	6.88E6
11	O00159|MYO1C_HUMAN	1618	true	77.73	57.53			2.53E5	1.00	1.28E5
12	O00192|ARVC_HUMAN	305	true	77.34	137.32			3.59E5	1.00	8.54E5
13	O00264|PGRC1_HUMAN	649	true	63.41	269.02			1.75E6	1.00	2.64E6
14	O00391|QSOX1_HUMAN	1845	true	101.16	63.46			1.04E4	1.00	8.81E3
15	O00443|P3C2A_HUMAN	1026	true	90.00	84.47			6.29E5	1.00	5.35E5
16	O00470|MEIS1_HUMAN	727	true	112.81	77.92			1.32E5	1.00	5.55E5
17	O00479|HMGN4_HUMAN	2159	true	106.92	59.14			3.93E4	1.00	4.16E4
18	O00505|IMA4_HUMAN	1583	true	59.85	94.42			1.42E6	1.00	1.28E6
19	O00506|STK25_HUMAN	862	false	85.55	108.46			2.36E5	1.00	1.37E5

The columns in the Protein Table are:

- **Accession:** The accession number of the protein as seen in the FASTA database.
- **Cluster:** A unique identifier that classifies which protein group the protein belongs to.
- **Top:** Indicates whether this protein is a top protein.
- **Significance:** The protein significance.
- **-10lgP:** The -10lgP score of the top PSM.
- **Sample Profile:** The protein abundance among the samples is depicted as a heat map. Hold the cursor on a profile to view the sample channels, abundances, and ratios with respect to the base sample.
- **Group Profile:** The protein abundance among the groups is depicted as a heat map. The Group Profile is determined by calculated the total abundance (area) of supporting peptides within a group. Hold the cursor on a profile to view the group channels, abundances, and ratios compared to the group that contains the base sample.
- **Group Intensity:** The intensity of each group as in the Group Profile tooltip.
- **Group Ratio:** The ratio of each group as in the Group Ratio tooltip.
- **Coverage (%):** The percentage of the protein sequence that is covered by supporting peptides. The coverage is visualized by a colored bar. Light blue blocks indicate the parts of the sequence covered by low-confidence peptides. Dark blue blocks indicate the parts covered by high-confidence peptides. This is the total coverage including results from all samples.
- **#Peptides:** The number of high-confidence supporting peptides.
- **#Unique:** The number of high-confidence supporting peptides that are mapped to only one protein group. Unique peptides with same sequence but different modifications are only counted once in this number.
- **PTM:** The identified modifications displayed with color-coded icons.
- **Avg. Mass:** The protein mass calculated using the average mass.
- **Description:** The protein's header information as seen in the FASTA database.

Note: For #Peptides and #Unique, two peptides with the same starting and ending positions in the protein are counted as one, regardless of their PTM forms. This is to follow the MCP (Molecular & Cellular Proteomics) guidelines.

9.4.3 Proteins View - Coverage Pane

The Protein Coverage view visually maps the supporting peptides and de novo tags to the protein selected in the Protein table. It also shows all identified sites with modifications or mutations to assist with protein

characterization at the amino acid level. The "Protein Coverage" view is composed of three major components - Protein Sequence Coverage, Coverage Control Panel, and Protein Tools.

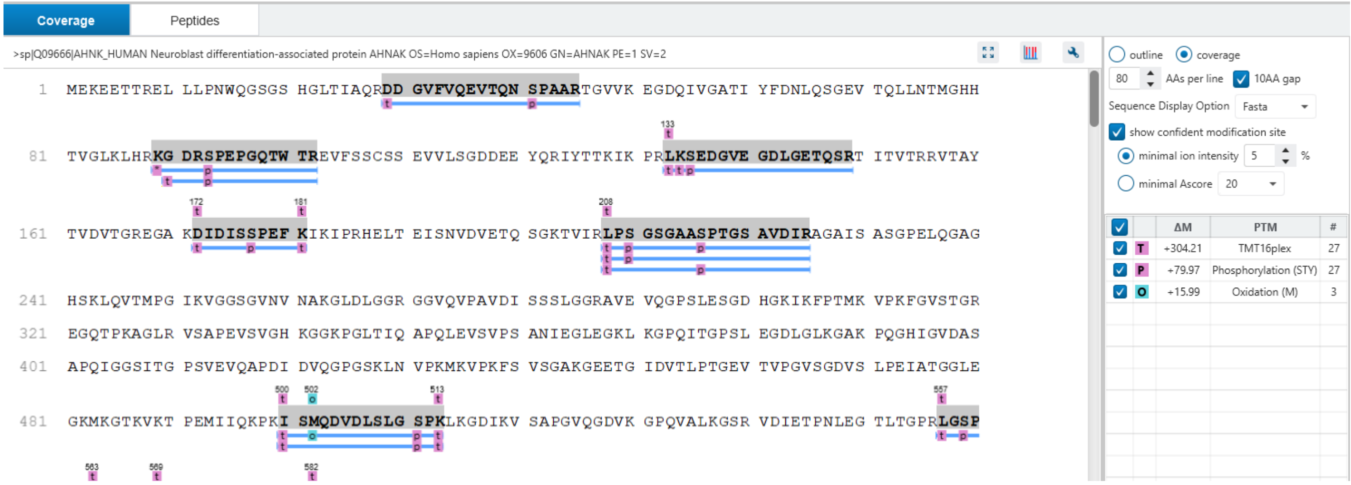

9.4.3.1 Protein Sequence Coverage

This area visualizes the coverage of the protein sequence.

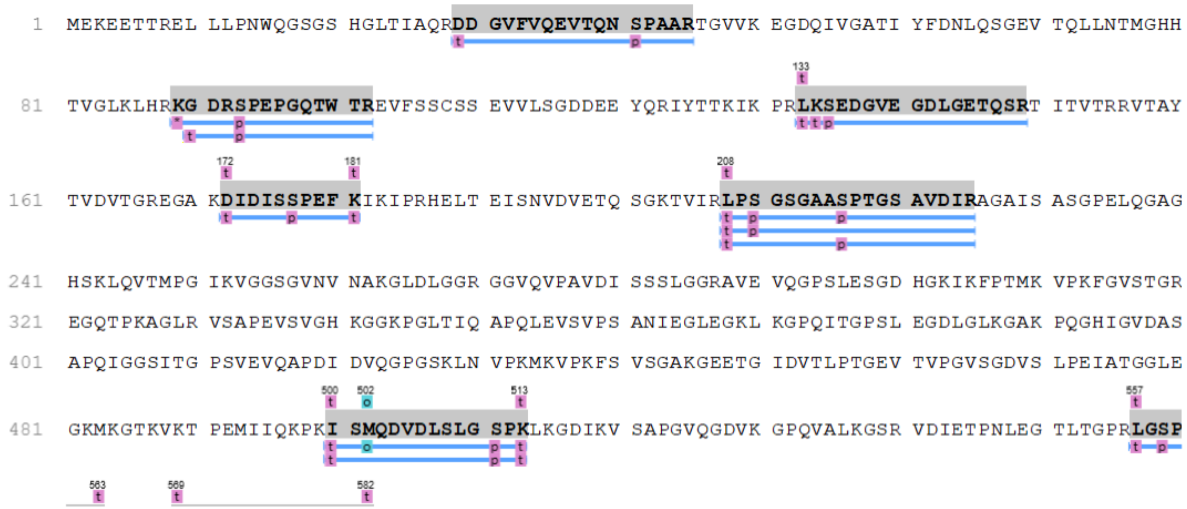

Regions in the protein sequence that are covered by supporting peptides are displayed in bold font with a grey background. Confident modifications and mutations identified in supporting peptides are displayed as icons above the protein sequence. Modifications are represented by colored icons with the first letter of its modification name. If a residue is modified by more than one modification in the same supporting peptide, "*" is used instead of a letter. The header information of the protein is shown on the top of the protein sequence.

The supporting peptides and matched de novo tags can be shown as colored bars under the protein sequence.

Placing the cursor over a bar shows a tooltip with detailed information of the peptide.

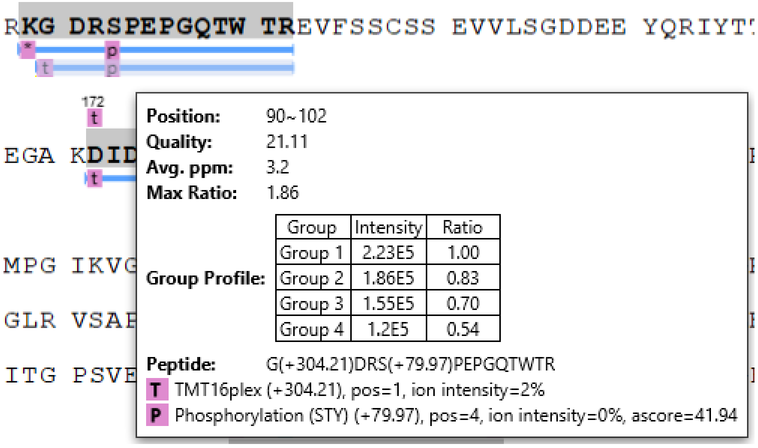

Left clicking on a bar shows the details from which the supporting peptide is identified.

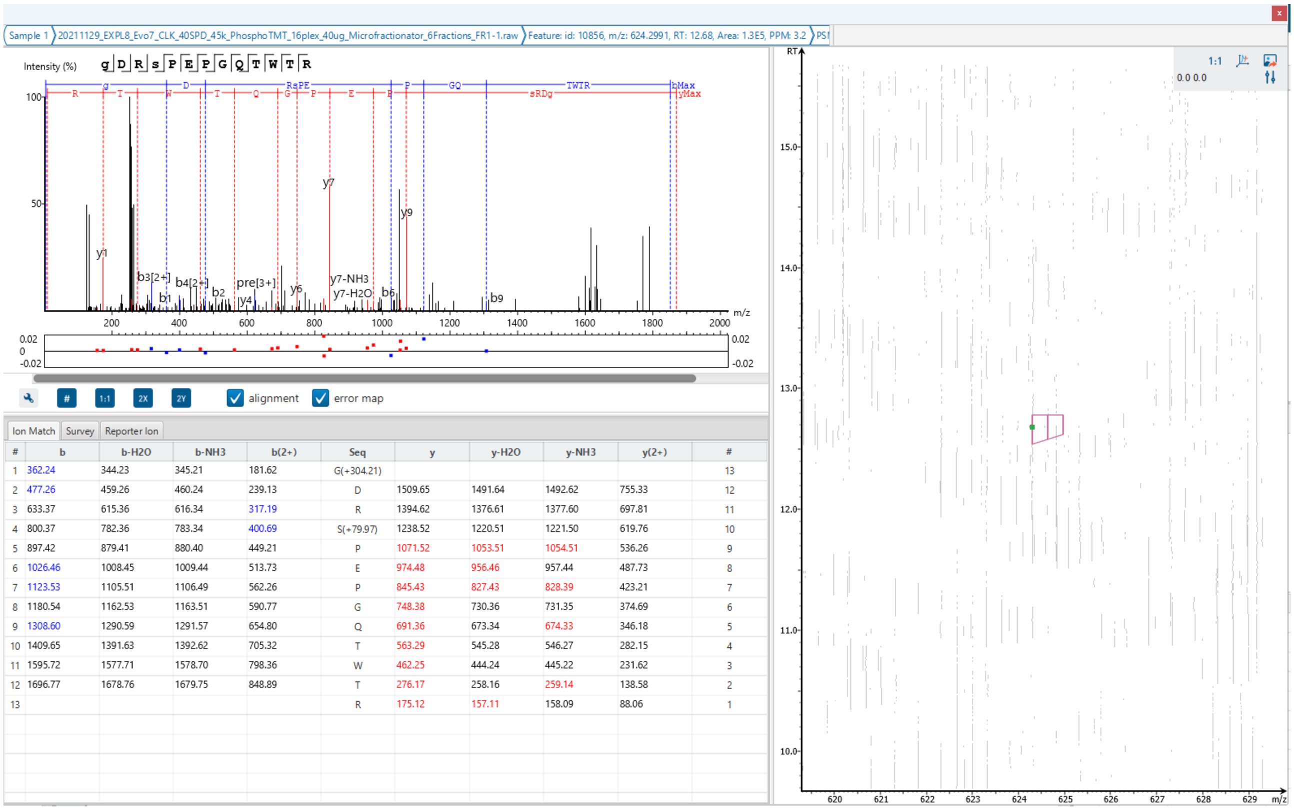

Right clicking on a peptide bar shows additional options. “Show in spectrum” has the same behaviour as left clicking on the peptide bar which is to open the annotated spectrum chart. “Show in table” has the function to jump to the supporting peptides table.

9.4.3.2 Coverage Control Panel

This panel controls what to display in the protein sequence coverage view.

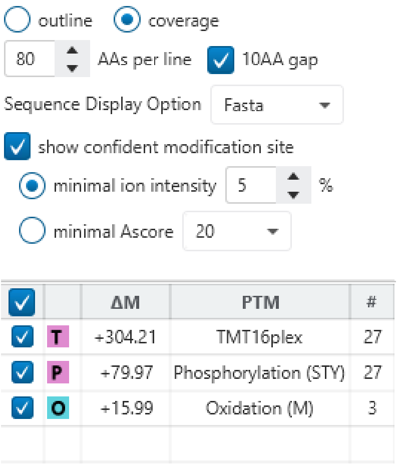

Sequence Display Mode: The protein sequence can be displayed in either "outline" or "coverage" mode.

Outline mode: In outline mode, the protein sequence is displayed without supporting peptides and matched de novo tags. However, the sequence coverage at a specific position can be examined by left-clicking a residue.

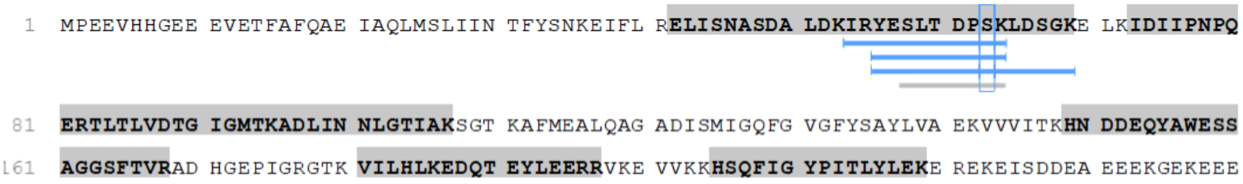

Coverage mode: In coverage mode, all the supporting peptides and matched de novo tags are shown.

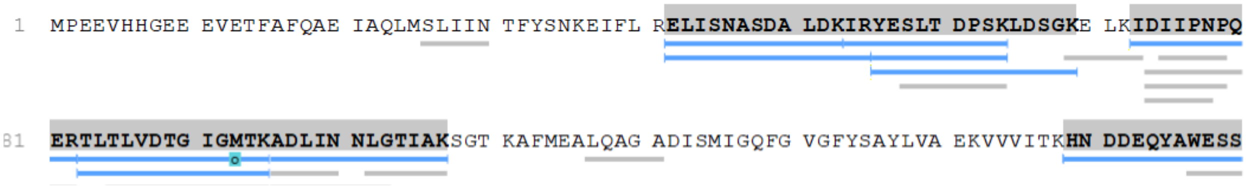

AAs per line and 10AA gap: Specify the number of amino acids per line and whether to show a gap for every 10 amino acids in the protein sequence.

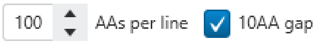

Sequence Display Option: Select whether to view the protein sequence as Fasta or a specific enzyme used in the project. When a particular enzyme is selected, the protein sequence will be highlighted to emphasize expected peptides generated by the selected enzyme's cleavage specificity.

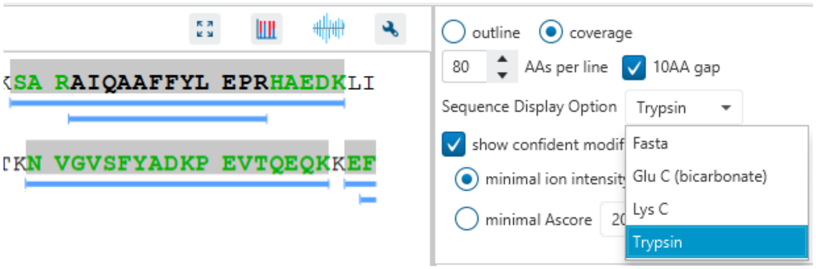

Modification minimal ion intensity / Ascore: A modification is considered confident if there are fragment ions supporting the modified residue with the minimal peak intensity above the specified threshold. Only confident modifications are displayed as icons above the protein sequence.

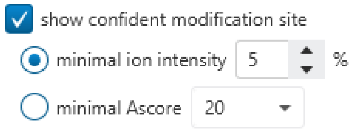

Modification table: The modification table shows the modifications identified in supporting peptides on the protein. For each modification, the number of supporting PSMs with this particular modification is shown. The checkbox on the left controls whether to indicate the modification in the protein coverage.

☑		ΔM	PTM	#
☑	T	+304.21	TMT16plex	27
☑	P	+79.97	Phosphorylation (STY)	27
☑	O	+15.99	Oxidation (M)	3

9.4.3.3 Proteins View - Protein Tools

The Proteins View toolbar is at the upper-right corner of the protein sequence coverage tab and contains helpful protein tools.

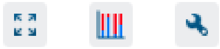

Full screen: 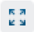 When selected, the protein coverage window expands in full screen mode. Click to return from full screen mode.

Tool box: 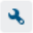 The tool box contains the following tools:

- Copy protein sequence. This copies the protein sequence of the current selected protein into the system clipboard.
- Save protein coverage as image. Different scale options are provided to increase the image size.
- Coverage statistics. This provides useful statistics based on the current selected protein.

PTM Profiling: 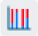 Calculates the difference in abundance between peptides with confidently identified PTMs versus unmodified peptides. Peptide feature areas are used for this comparison.

The PTM Profiling tool provides quantitative information of modified peptides compared with unmodified peptides for the modification sites of the protein across all MS samples.

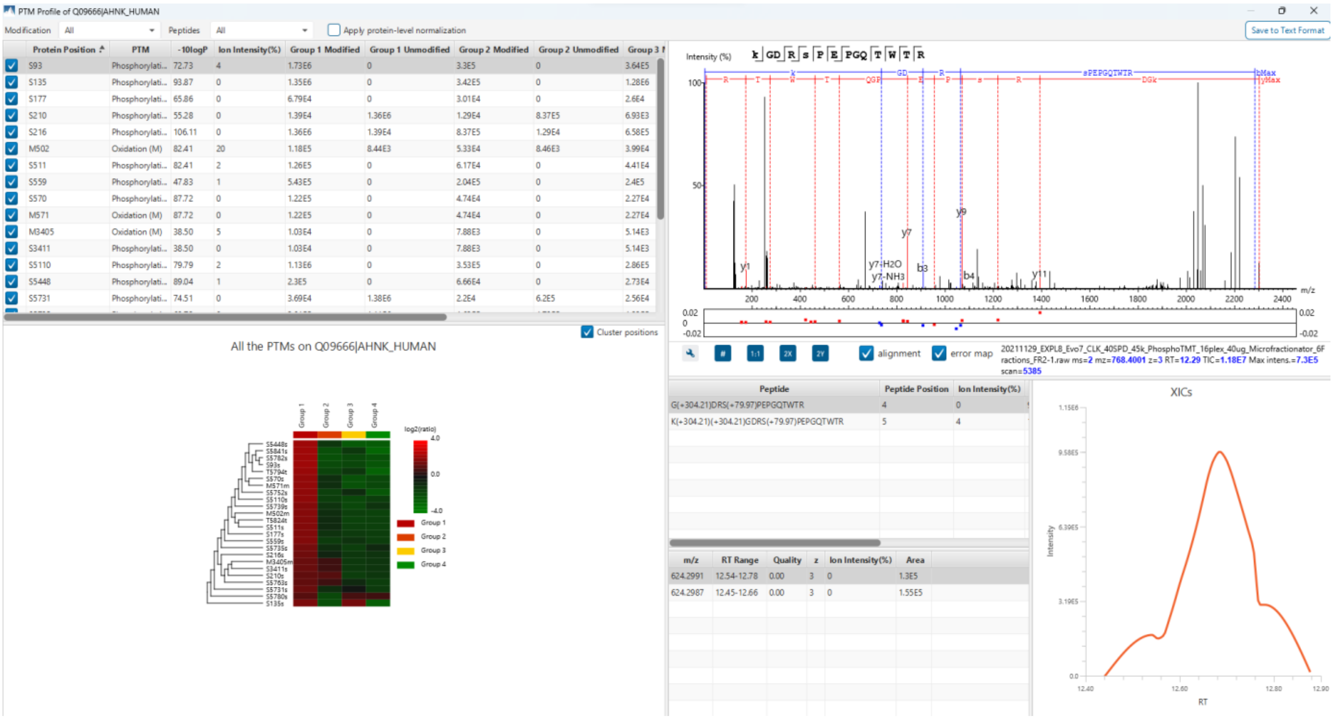

The inputs for the profiling can be set using the drop-down lists at the top-left corner of the dialog.

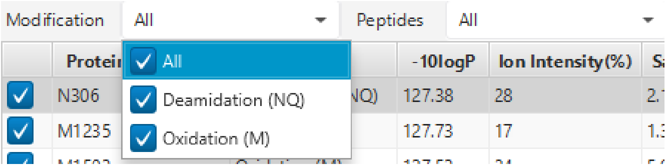

- **Proteins:** The accession ID of the selected protein to be analyzed for PTM profiling is shown in the title of the dialog. The selected protein can not be changed within the dialog. To run profiling on another protein, close the dialog, select the protein of interest from the protein table and run the profiling by clicking 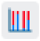 button.
- **Modification:** The drop-down list contains the detected and selected confident PTMs for the protein. Select the type of modification to study a specific PTM or choose “All the PTMs” to visualize the profiling information of all PTM sites at together.

  Note: The fixed PTMs are not used for profiling. In addition to this, the labeling modifications are not considered either.

- **Peptides:** Data source used for the analysis. “All” considers all the peptides for this protein.
- **Apply protein-level normalization:** By default, the protein-level normalization is not applied. If checked, the displayed modified/unmodified area is obtained by dividing the true modified/unmodified area by the corresponding ratio of the selected protein.

PTM Profile Table

The PTM Profile Table shows the detailed quantitative information for the modification sites. Each row in the table shows the modified and unmodified area for each sample of the selected PTM at each detected modification site. The table can be sorted by any column. The following list describes the contents in each column:

- **(Check box):** Allow the selection of each table row. Only the marked rows are used for the PTM Profile Chart.
- **Protein Position:** The detected modification sites in the protein. The modified amino acid symbol together with the position in the protein sequence is shown.
- **PTM:** The type of PTM being profiled.
- **-10logP:** The best -10logP identification score for the corresponding modified peptides.
- **AScore/ion intensity:** The best PTM AScore / ion intensity associated with the corresponding PTM at the specified site of the modified peptides.
- **Group Modified:** Total area of the features associated with the corresponding modified peptides for each sample.
- **Group Unmodified:** Total area of the features associated with the corresponding unmodified peptides for each sample.

Modification: All | Peptides: All | ☑ Apply protein-level normalization

	Protein Position	PTM	-10logP	Ion Intensity(%)	Group 1 Modified	Group 1 Unmodified	Group 2 Modified	Group 2 Unmodified	Group 3
☑	S93	Phosphorylati...	72.73	4	1.73E6	0	8.38E5	0	8.29E5
☑	S135	Phosphorylati...	93.87	0	1.35E6	0	8.67E5	0	2.91E6
☑	S177	Phosphorylati...	65.86	0	6.79E4	0	7.63E4	0	5.93E4
☑	S210	Phosphorylati...	55.28	0	1.39E4	1.36E6	3.26E4	2.13E6	1.58E4
☑	S216	Phosphorylati...	106.11	0	1.36E6	1.39E4	2.13E6	3.26E4	1.5E6
☑	M502	Oxidation (M)	82.41	20	1.18E5	8.44E3	1.35E5	2.15E4	9.11E4
☑	S511	Phosphorylati...	82.41	2	1.26E5	0	1.57E5	0	1.01E5
☑	S559	Phosphorylati...	47.83	1	5.43E5	0	5.17E5	0	5.46E5
☑	S570	Phosphorylati...	87.72	0	1.22E5	0	1.2E5	0	5.17E4
☑	M571	Oxidation (M)	87.72	0	1.22E5	0	1.2E5	0	5.17E4
☑	M3405	Oxidation (M)	38.50	5	1.03E4	0	2E4	0	1.17E4
☑	S3411	Phosphorylati...	38.50	0	1.03E4	0	2E4	0	1.17E4
☑	S5110	Phosphorylati...	79.79	2	1.13E6	0	8.96E5	0	6.52E5
☑	S5448	Phosphorylati...	89.04	1	2.3E5	0	1.69E5	0	6.23E4
☑	S5731	Phosphorylati...	74.51	0	3.69E4	1.38E6	5.59E4	1.57E6	5.83E4
[illegible]	[illegible]	[illegible]	[illegible]	[illegible]	[illegible]	[illegible]	[illegible]	[illegible]	[illegible]

PTM Profile Heat Map

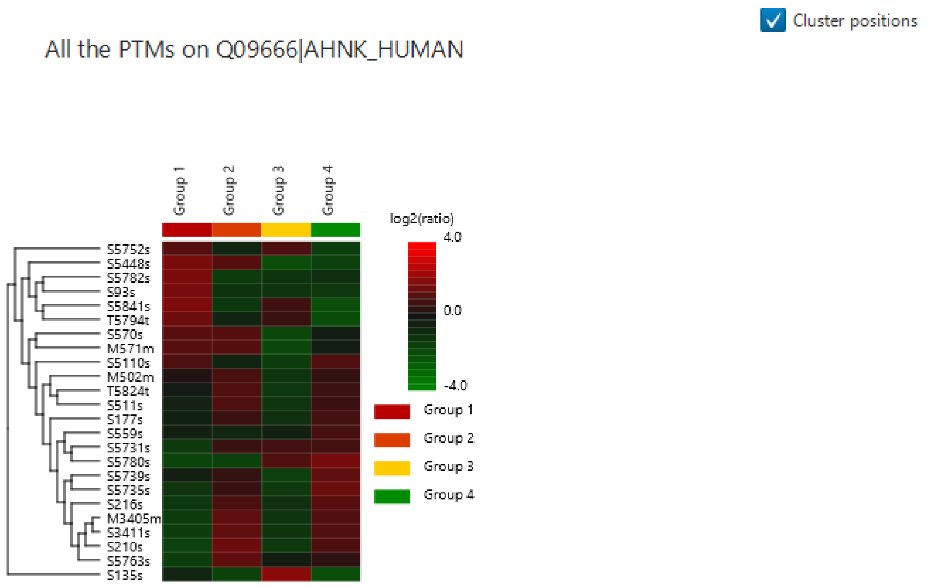

The PTM Profile Heat Map is located at the bottom left corner in the dialog. The relative abundance of modified peptides in all groups for the selected modification sites as a heat map. The color scheme is determined by the log2 ratios of the modified area of each group relative to the average abundance. For more details about the heat map, please refer to section 9.3.3 Summary View - Heat Map.

Spectrum Annotation View

The Spectrum Annotation View provides a graphical representation for the best modified peptide and the best unmodified peptide when “All” is selected from the “Peptides” drop-down list. The top spectrum corresponds to the best modified peptide, whereas the bottom spectrum shows the best unmodified peptide.

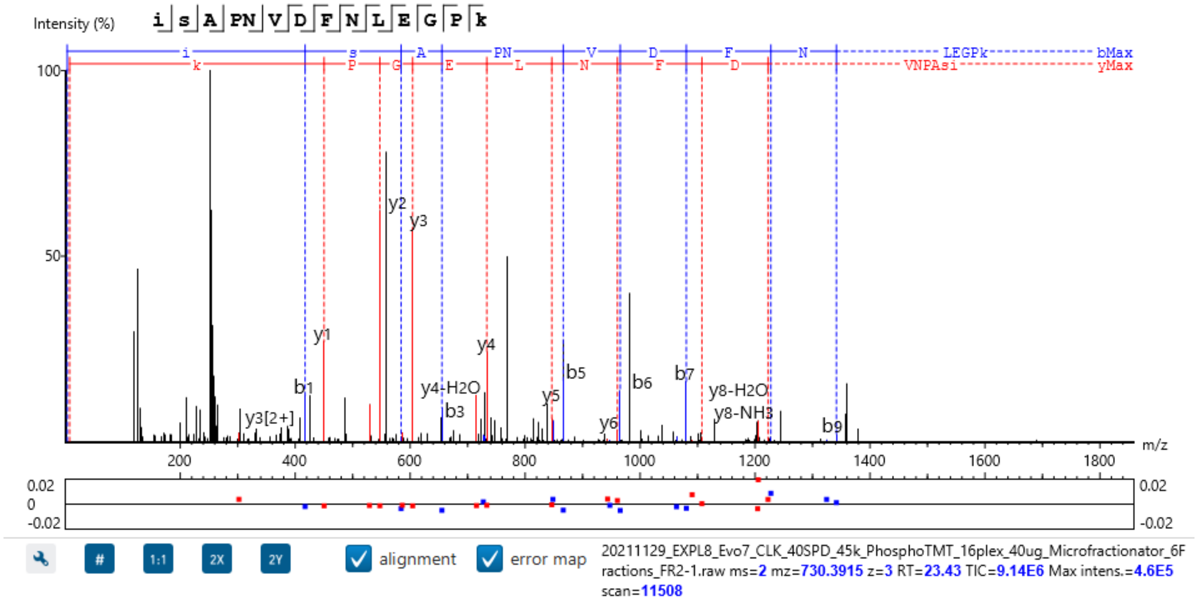

Peptides Table: Peptides table lists the modified peptides that contain the selected PTM at the specified modification site.

Peptide	Peptide Position	Ion Intensity(%)
I(+304.21)SM(+15.99)QDVDLSLGS(+79.97)PK(+304.21)	3	20

All Matches Table: The supporting PSMs of the selected peptide in Peptides Table are listed in the All Matches Table. The headers are self-explanatory. The last column Area is the area of the feature that is associated with the corresponding PSM.

m/z	RT Range	Quality	z	Ion Intensity(%)	Area
732.0529	22.40-22.48	0.00	3	18	4.79E4
732.0516	22.35-22.45	0.00	3	20	2.01E5

XICs: It shows the XICs figure for the selected modified match in the All Matches Table.

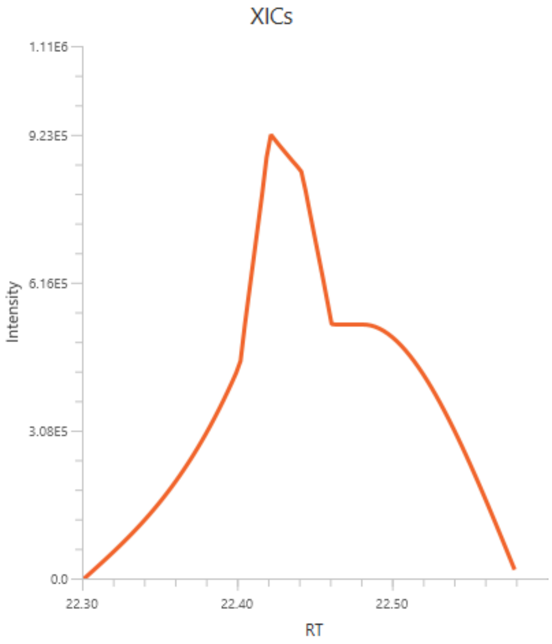

PTM Profile Export

The PTM profile can be exported as an image as well as in text format.

Image export: To save the profile chart, right click and choose Save to Image... menu item to save the displayed graph as a PNG file.

Text format export: Click Save to Text Format button at the top-right corner to export the calculated PTM Profile data to a text file in CSV format. The PTM Profile of the selected modification on the selected protein will be exported by default. In addition, profiles on all proteins for the selected modification may also be exported, if "Export ptm profile for all proteins" is checked. The exported file contains all necessary information to construct the PTM profile graph. The PTM profile data are grouped together by protein, then by modification type, and then by the modification site on the protein sequence. The headers of the CSV column are similar to the PTM Profile Table and also self-explanatory. The following items are explained for clarity:

- **Modified Peptide Sequence Window:** protein sequence containing 10 AA ahead of the modification site and 10 AA after the modification site with padding as necessary.
- **Sample Best AScore / Ion Intensity:** best AScore or Ion Intensity for each sample.

Note: When "Export PTM profile for all proteins" is not checked, only the profiling data that is marked in the PTM Profile Table will be exported.

9.4.3.4 Proteins View - Supporting Peptides

The supporting peptides assigned to the protein are shown in a separate tab beside the Protein Coverage view. The sequence can be clicked and a pop-up window will show up to display the feature details.

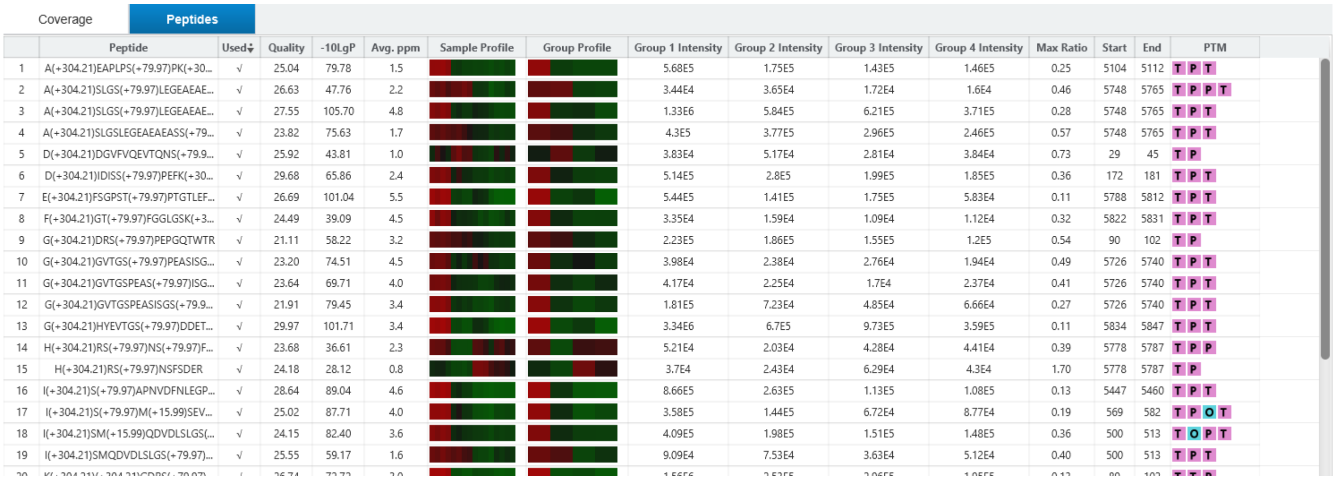

	Peptide	Used	Quality	-10LgP	Avg. ppm	Sample Profile	Group Profile	Group 1 Intensity	Group 2 Intensity	Group 3 Intensity	Group 4 Intensity	Max Ratio	Start	End	PTM
1	A(+304.21)EAPLPS(+79.97)PK(+30...	√	25.04	79.78	1.5			5.68E5	1.75E5	1.43E5	1.46E5	0.25	5104	5112	T P T
2	A(+304.21)SLGS(+79.97)LEGEAEAE...	√	26.63	47.76	2.2			3.44E4	3.65E4	1.72E4	1.6E4	0.46	5748	5765	T P P T
3	A(+304.21)SLGS(+79.97)LEGEAEAE...	√	27.55	105.70	4.8			1.33E6	5.84E5	6.21E5	3.71E5	0.28	5748	5765	T P T
4	A(+304.21)SLGSLEGEAEAEASS(+79...	√	23.82	75.63	1.7			4.3E5	3.77E5	2.96E5	2.46E5	0.57	5748	5765	T P T
5	D(+304.21)DGVFVQEVTQNS(+79.9...	√	25.92	43.81	1.0			3.83E4	5.17E4	2.81E4	3.84E4	0.73	29	45	T P
6	D(+304.21)IDISS(+79.97)PEFK(+30...	√	29.68	65.86	2.4			5.14E5	2.8E5	1.99E5	1.85E5	0.36	172	181	T P T
7	E(+304.21)FSGPST(+79.97)PTGTLEF...	√	26.69	101.04	5.5			5.44E5	1.41E5	1.75E5	5.83E4	0.11	5788	5812	T P T
8	F(+304.21)GT(+79.97)FGGLGSK(+3...	√	24.49	39.09	4.5			3.35E4	1.59E4	1.09E4	1.12E4	0.32	5822	5831	T P T
9	G(+304.21)DRS(+79.97)PEPGQTWTR	√	21.11	58.22	3.2			2.23E5	1.86E5	1.55E5	1.2E5	0.54	90	102	T P
10	G(+304.21)GVTGS(+79.97)PEASISG...	√	23.20	74.51	4.5			3.98E4	2.38E4	2.76E4	1.94E4	0.49	5726	5740	T P T
11	G(+304.21)GVTGSPEAS(+79.97)ISG...	√	23.64	69.71	4.0			4.17E4	2.25E4	1.7E4	2.37E4	0.41	5726	5740	T P T
12	G(+304.21)GVTGSPEASISGS(+79.9...	√	21.91	79.45	3.4			1.81E5	7.23E4	4.85E4	6.66E4	0.27	5726	5740	T P T
13	G(+304.21)HYEVTGS(+79.97)DDET...	√	29.97	101.71	3.4			3.34E6	6.7E5	9.73E5	3.59E5	0.11	5834	5847	T P T
14	H(+304.21)RS(+79.97)NS(+79.97)F...	√	23.68	36.61	2.3			5.21E4	2.03E4	4.28E4	4.41E4	0.39	5778	5787	T P P
15	H(+304.21)RS(+79.97)NSFSDER	√	24.18	28.12	0.8			3.7E4	2.43E4	6.29E4	4.3E4	1.70	5778	5787	T P
16	I(+304.21)S(+79.97)APNVDFNLEGP...	√	28.64	89.04	4.6			8.66E5	2.63E5	1.13E5	1.08E5	0.13	5447	5460	T P T
17	I(+304.21)S(+79.97)M(+15.99)SEV...	√	25.02	87.71	4.0			3.58E5	1.44E5	6.72E4	8.77E4	0.19	569	582	T P O T
18	I(+304.21)SM(+15.99)QDVDLSLGS(...	√	24.15	82.40	3.6			4.09E5	1.98E5	1.51E5	1.48E5	0.36	500	513	T O P T
19	I(+304.21)SMQDVDLSLGS(+79.97)...	√	25.55	59.17	1.6			9.09E4	7.53E4	3.63E4	5.12E4	0.40	500	513	T P T
20	[illegible]	[illegible]	[illegible]	[illegible]	[illegible]			[illegible]	[illegible]	[illegible]	[illegible]	[illegible]	[illegible]	[illegible]	[illegible]

This table contains the following information of the quantifiable support peptides for the selected protein:

- **Peptide:** The amino acid sequence of the peptide, as determined in PEAKS Database or Spectral Library search. A modified residue is followed by a pair of parentheses enclosing the modification mass. All non-unique peptides and those not passing the filter will be excluded automatically.
- **Used:** Shows whether this peptide is used to calculate the protein abundance. The top three peptides with highest abundance are used to calculate the protein abundance.
- **Quality:** This defines how well the peptide can be quantified. It is calculated by taking the average of the quality scores of the feature vectors.
- **-10lgP:** The -10lg of a p-value represents the likelihood that the observed change between conditions is caused by random chance. The peptides are first separated into groups based on similar quality score. Significance is then calculated for each quality score group, or bin, using a two-tail T-test that assumes log normal distribution but does not assume equal variance. The -10log p-value is then calculated from this result and then displayed in the significance column.
- **Avg. ppm:** A peptide can have one or more feature vectors. Each feature vector has its own ppm calculated from the mass error of each feature. The average mass error of a peptide is the average of mass errors of all the feature vectors. This column displays the average mass error in ppm.
- **Sample Profile:** The peptide abundance among the samples is depicted as a heat map. Hold the cursor on a profile to view the samples, the peptide areas in samples, and the corresponding ratios with respect to the base sample.
- **Group Profile:** The peptide abundance among the groups is depicted as a heat map. Hold the cursor on a profile to view the groups, the peptide areas in groups, and the corresponding ratios with respect to the group which contains the base sample.

- **Group Intensity:** This column shows the peptide area for a group. It is calculated by summing up the intensities of feature vectors within a group.
- **Max Ratio:** This shows the maximum of the fold change values compared to the reference sample.
- **Start:** This shows the protein position of the first residue of the peptide.
- **End:** This shows the protein position of the last residue (inclusive) of the peptide.
- **PTM:** The types and the numbers of modifications present in the peptide shown using color-coded icons. Scroll over each icon to see the modification name and mass.

9.5 TMT/iTRAQ result - Peptides View

Quantified proteins are displayed in the Peptide View along with their detailed information.

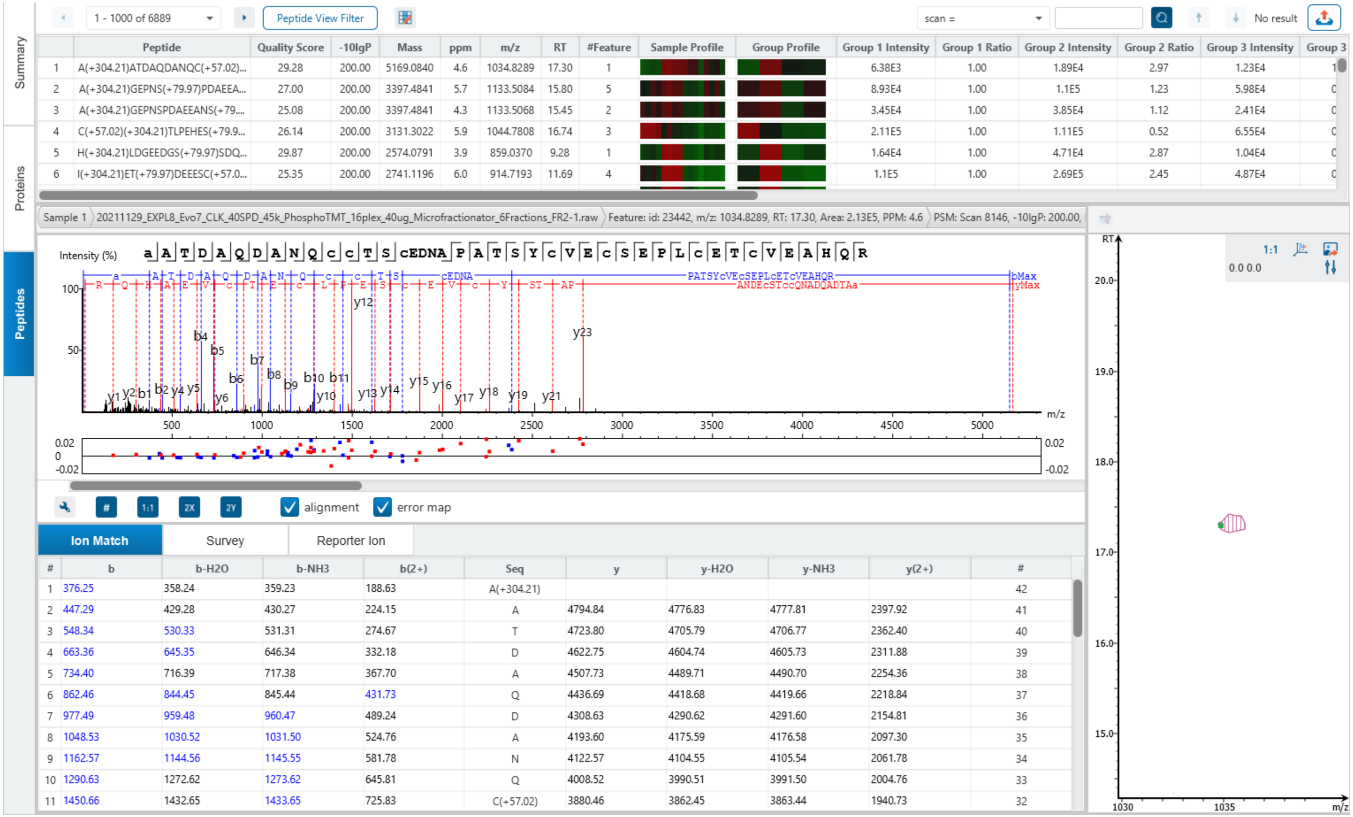

9.5.1 Peptides View - Peptide Table

The peptides are grouped together based on quantifiable feature vectors with the same sequence. When there are more than 1000 peptides, the table is split into multiple pages.

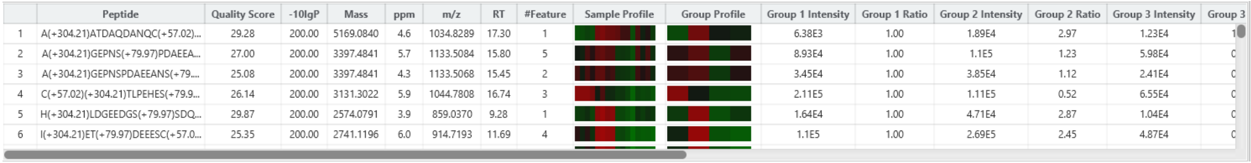

The view is similar to the support peptide table described in the previous section Section 9.4.3.4 Proteins View - Supporting Peptides. The Peptide table entries may be filtered by using the Peptide View Filters on top of the peptide table. To reiterate, the columns are:

- **Peptide:** The amino acid sequence of the peptide, as determined in PEAKS Database or Spectral Library search. A modified residue is followed by a pair of parentheses enclosing the modification mass. All non-unique peptides and those not passing the filter will be excluded automatically.
- **Quality:** This defines how well the peptide can be quantified. It is calculated by taking the average of the quality scores of the feature vectors.
- **-10lgP:** The -10lg of a p-value represents the likelihood that the observed change between conditions is caused by random chance. The peptides are first separated into groups based on similar quality score. Significance is then calculated for each quality score group, or bin, using a two-tail T-test that assumes log normal distribution but does not assume equal variance. The -10log p-value is then calculated from this result and then displayed in the significance column.
- **Mass:** The theoretical mass of the peptide.
- **ppm:** A peptide can have one or more feature vectors. Each feature vector has its own ppm calculated from the mass error of each feature. The average mass error of a peptide is the average of mass errors of all the feature vectors. This column displays the average mass error in ppm.
- **m/z:** The precursor mass-to-charge ratio.
- **RT:** The retention time (elution time) at which the spectrum was recorded in the data.
- **#Feature:** The number of features associated with this peptide.
- **Sample Profile:** The peptide abundance among the samples is depicted as a heat map. Hold the cursor on a profile to view the samples, the peptide areas in samples, and the corresponding ratios with respect to the base sample.
- **Group Profile:** The peptide abundance among the groups is depicted as a heat map. Hold the cursor on a profile to view the groups, the peptide areas in groups, and the corresponding ratios with respect to the group which contains the base sample.
- **Group Intensity:** This column shows the peptide area for a group. It is calculated by summing up the intensities of feature vectors within a group.
- **Group Ratio:** This column shows the peptide ratio for a group, based on the ratio of intensities of feature vectors within a group.
- **Scan:** The Scan of the top PSM and which data file it is from.
- **PTM:** The types and the numbers of modifications present in the peptide shown using color-coded icons. Scroll over each icon to see the modification name and mass.
- **Accession:** The proteins that this peptide was quantified in.

To copy the sequence, right click on a row (or multiple rows) in the table and select “Copy selected peptide sequence(s)”. Another option is to mouse over any cell in the table and hitting Ctrl + C on your keyboard to copy the contents of those selected cells.

9.5.2 Peptide Table controls

The menu above the peptide table provides options to scroll through the table of peptides, filter the table, show optional columns and search for specific entries.

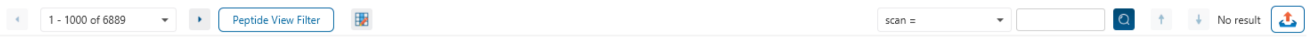

9.5.2.1 Peptide Table controls - Pagination

For easy viewing and to improve performance, the peptide table is limited to showing 1000 entries at once. Pagination is used to manage which set of 1000 entries will be viewed.

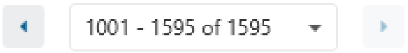

In the top left, there is a dropdown to select the rows for viewing. Click the left arrow or right arrow to select the previous/next 1000 rows. If the table is sorted or filtered, it may update the pagination set of rows that is currently in view.

9.5.2.2 Peptide Table controls - View Filter

Click on the 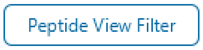

button to open the Peptide View Filter which is used to filter the Peptide table. The following are the filter options provided:

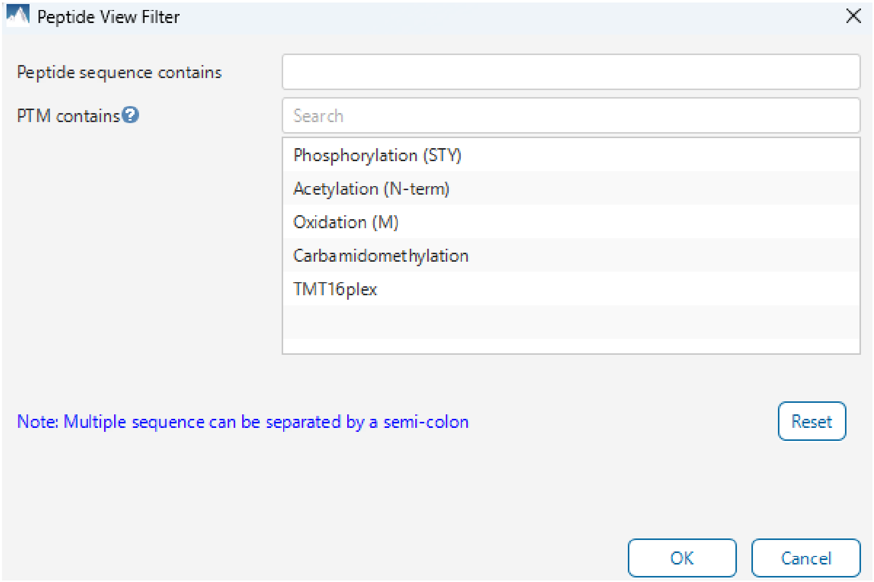

- **Peptide sequence contains:** Enter an amino acid sequence, only peptides that contain that sequence will be displayed in the peptide table. Adding PTMs in brackets in the sequence search is not handled, meaning only the sequence backbone is considered in the filter. Use the PTM contains for further filtering if necessary.
- **PTM contains:** Type the names of PTMs you would like to include. Peptides containing PTMs that match the text will be included. If multiple PTMs match the text they will all be included in the table.

Note: In all filters, multiple entries can be separated by a semi-colon.

Click on the Reset button to reset all filters to their default setting.

Click on OK to apply the filters to the Peptides table.

Click on Cancel to discard the changes in the View Filter.

9.5.2.3 Peptide Table controls - Optional Columns

Click on the 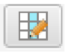 button to open the optional columns dropdown. By default, the group intensities and ratios are displayed. The sample intensities and ratios can also be displayed in the peptide table for easy view and sorting. For projects with many samples, it might be useful to hide these optional columns and reduce the width of the peptide table.

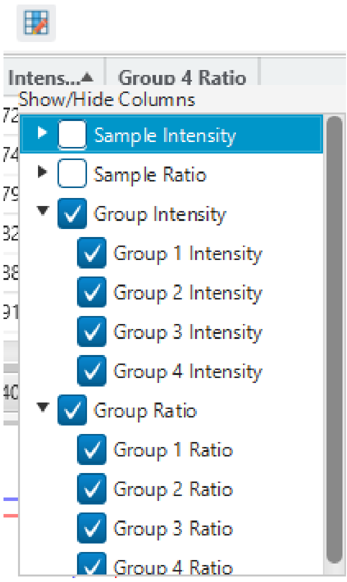

9.5.2.4 Peptide Table controls - Search function

On the top right of menu, there is a search function with 4 options. Search results can be navigated between using the Up or Down arrows.

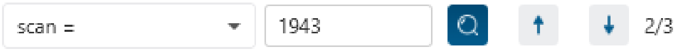

Options for searching include:

- **Scan =:** Search for the Scan number associated with each peptide. One scan can be associated to multiple peptides. For projects with multiple data files, the scan number is searched on every fraction.
- **m/z ≈:** Search for the approximate m/z. The search will consider a range of m/z equal to the search value +/- 1 of the last significant digit. For example, searching for m/z ≈ 560.21 will find all m/z between [560.20, 560.22]
- **RT ≈:** Search for the approximate RT. Likewise, the search will consider a range of RT equal to the search value +/- 1 of the last significant digit set. For example, searching for RT ≈ 55.5 will find all m/z between [55.4, 55.6]
- **Seq contains:** Search for the sequence in the table. If specifying a sequence without PTMs, the results will include that sequence and any sequences with PTMs with the same backbone. If specifying a sequence with bracket and some mass value (like in the above image), then only sequence that have a substring with an exact match will be considered.
  Additionally, since the peptide table shows both I/L and they can not be distinguished, then searching for I in the sequence is equivalent to searching for L in the sequence. i.e. Searching for “LVLK” is the same as searching for “LVIK”, “IVLK”, and “IVIK”

9.5.3 Feature selection breadcrumb

There is a sample and feature selection breadcrumb which allows you to switch between features associated with the peptide. Click on the Sample in the breadcrumb to reveal other samples and the features in that sample related to the current peptide selection. From here you can select other samples, fractions, or features.

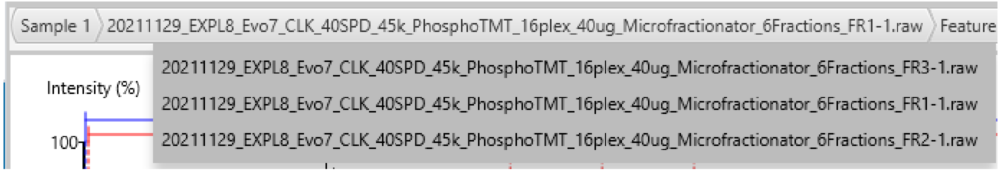

By clicking on the fraction level, all fractions in this sample are revealed as well as the features in each fraction associated with this peptide. From here you can select other fractions and features to view.

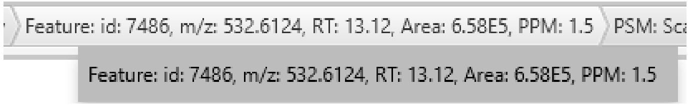

Clicking on the feature level will show all features in the currently selected fraction. Features with Feature Id = 0 are known as fake features and are constructed to enhance identification.

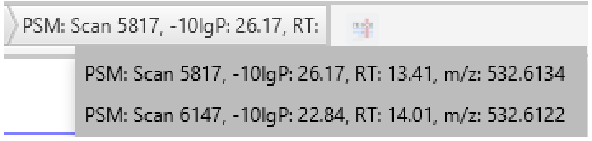

Switch between PSMs with the last portion of the breadcrumb.

All other components in the Peptide tab interface will update according to the currently selected PSM.

For timsTOF instruments, Precursor ID is used rather than Scan number.

9.5.4 Protein jump button

Click on 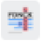 to jump to a protein that contains the currently selected PSM. If there are more than 1 protein, a dropdown is revealed which allows the user to select which protein to jump to. If a protein is filtered out due to a protein summary filter or summary page protein filter, then the option to jump will be grayed out.

9.5.5 Annotated Spectrum Chart

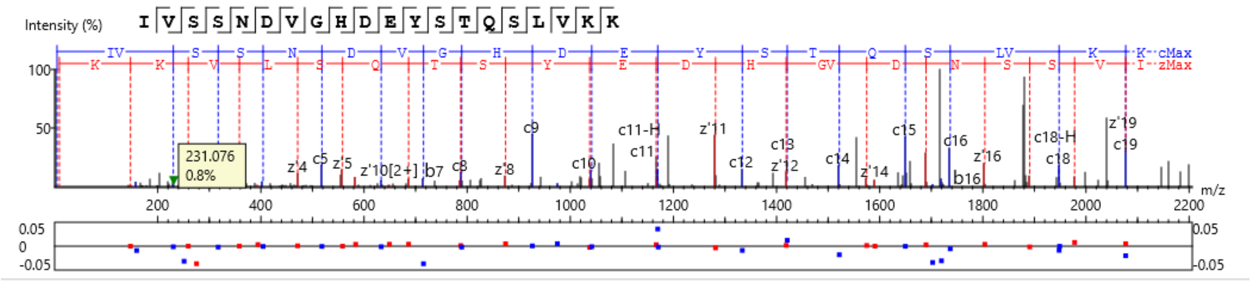

For the currently selected PSM, a graphical representation is shown in the form of an annotated spectrum chart. There are several controls provided for this view which will be explored in the following sections.

9.5.5.1 Annotated Spectrum Chart - Chart Navigation

Moving the cursor over the peptide sequence in the spectrum will show the mass transitions for a particular amino acid residue.

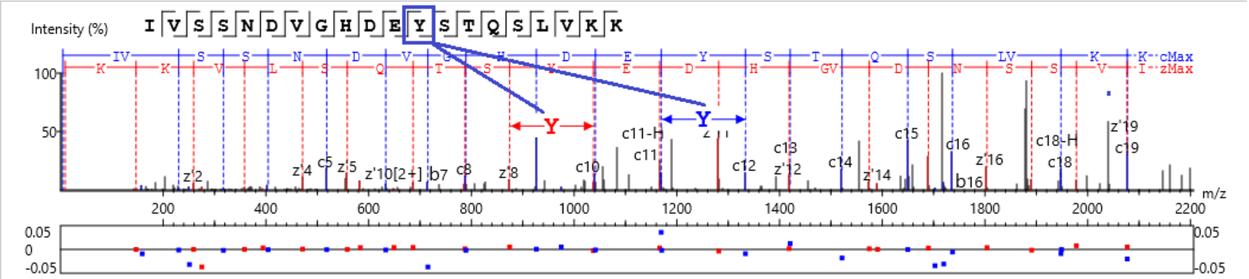

Zoom to a m/z region: Drag horizontally from the start m/z and to the end m/z with left mouse button.

Zoom in/out smoothly: Place the cursor at a particular m/z value (right below the x-axis), zoom in/out by scrolling the mouse wheel.

Increase/Decrease peak intensity: Place the cursor in the spectrum and scroll the mouse wheel.

See the whole spectrum: Double click in the spectrum or click the 1:1 button.

9.5.5.2 Annotated Spectrum Chart - Spectrum Annotation Settings

Click on the 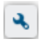 button to show the spectrum annotation settings.

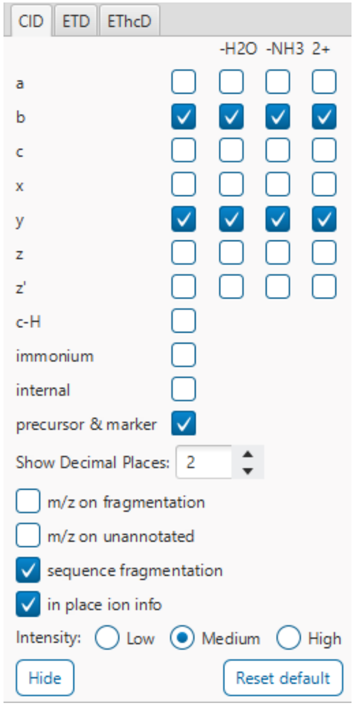

The spectra in the PEAKS results can be annotated by selecting ion types from the thorough collection of ions that PEAKS offers. The selected ion types will be displayed in the "Ion Match" table, as well. It is possible to annotate the spectrum with various ions for CID, EThcD and ETD. Each of these fragmentation modes can be set with different settings which will be shared with other results with the same fragmentation type.

To reset the defaults, click on the "Reset default" button.

Show Decimal Places: Select the number of decimal places that will appear in the ion table and the spectrum view. The default is set to two decimal places.

m/z on Fragmentation: Select this to display the m/z value on top of the annotated ions.

m/z on Unannotated: Select this to display the m/z value on top of the peaks without ion matches.

Sequence Fragmentation: Select this to display the sequence fragmentation on the top-left corner of the "Spectrum Annotation" view.

In Place Ion Info: Ion information, m/z value,and relative intensity are all displayed in a pop-up within the "Spectrum Annotation" view when this option is checked and the cursor is placed on a peak.

Intensity: Set the intensity threshold for spectrum annotation to low (2%), medium (5%), or high (10%).

9.5.5.3 Annotated Spectrum Chart - Additional Chart Controls

Click on 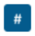 or 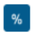 to toggle between absolute and percentage intensity values in the Y axis.

Click on 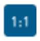 to reset the zoom level of the annotated spectrum chart to the default.

Click on 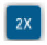 to zoom into the X axis by a factor of 2.

Click on 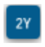 to zoom into the Y axis by a factor of 2.

The "alignment" check box alignment allows user to display/hide how the fragment ions generated from the peptide align with the spectrum. N-terminal ions are shown in blue and C-terminal ions are shown in red.

The “error map” check box error map is used to show/hide the error map underneath the chart.

9.5.6 Ion Match Table

The “Ion Match” tab in the bottom pane contains a table with possible fragment ions for the selected MS/MS scan. Each ion in the table shows the calculated mass, where if the fragment ion is present in the spectrum, its mass value is displayed in color. N-terminal ions are shown in blue and C-terminal ions are shown in red. A fragment ion is found when there is a matching peak within the mass error tolerance, as defined in the de novo sequencing parameters, and when relative intensity of the matching peak is at least 2%. The ion types displayed in the table can be configured in "Spectrum Annotation Settings”.

Ion Match | Survey

#	b	b-H2O	b-NH3	b(2+)	Seq	y	y-H2O	y-NH3	y(2+)	#
10	1079.503	1061.488	1062.498	540.248	E	1596.643	1578.636	1579.610	798.818	13
11	1265.576	1247.569	1248.557	633.292	W	1467.601	1449.588	1450.585	734.297	12
12	1394.626	1376.616	1377.600	697.813	E	1281.521	1263.512	1264.488	641.266	11
13	1481.659	1463.648	1464.632	741.329	S	1152.480	1134.469	1135.461	576.736	10
14	1596.682	1578.671	1579.655	798.841	N(+0.98)	1065.449	1047.440	1048.427	533.228	9
15	1653.703	1635.693	1636.676	827.352	G	950.422	932.407	933.390	475.709	8
16	1781.773	1763.751	1764.735	891.381	Q	893.397	875.388	876.370	447.198	7
17	1878.815	1860.804	1861.788	939.907	P	765.341	747.326	748.315	383.169	6
18	2007.857	1989.847	1990.830	1004.429	E	668.288	650.274	651.258	334.642	5
19	2122.880	2104.870	2105.853	1061.940	N(+0.98)	539.245	521.231	522.222	270.124	4
[illegible]	[illegible]	[illegible]	[illegible]	[illegible]	[illegible]	[illegible]	[illegible]	[illegible]	[illegible]	[illegible]

Note: Uncheck the alignment checkbox in the spectrum annotation view to see the highlighted fragment ions more easily.

9.5.7 Survey Chart

The “Survey” tab displays the precursor ion spectrum. The buttons that appear in this section are identical to those explained above in Section 9.5.5.3 Annotated Spectrum Chart - Additional Chart Controls

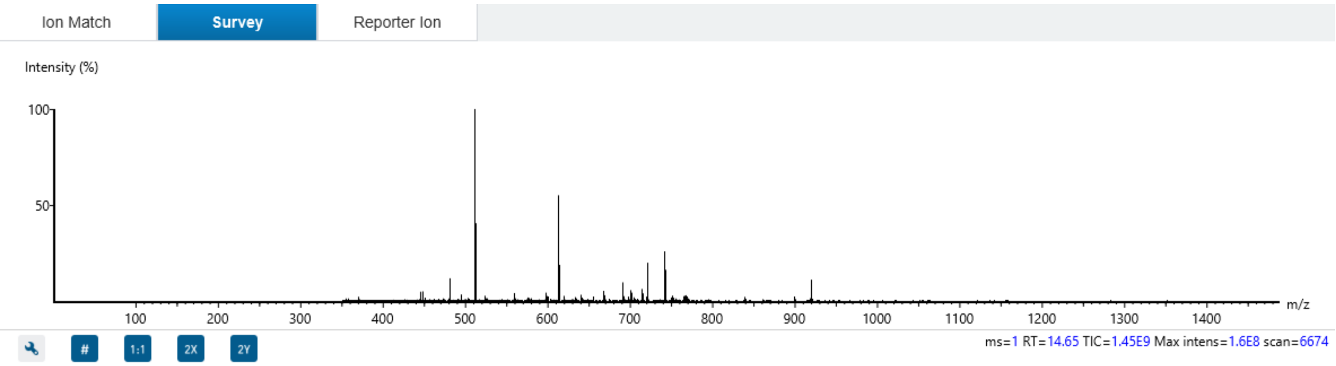

9.5.8 Reporter Ion

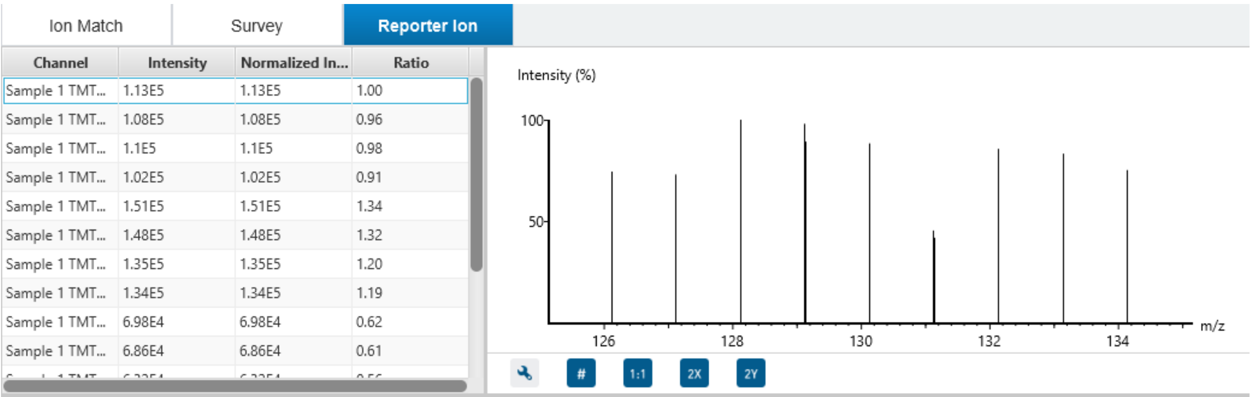

This view provides details on characteristics of the reporter ions used in the experiment. The table provides information on the intensity, the raw intensity, and the ratio of each channel with respect to the set reference channel. Additionally, an associated MS2 or MS3 spectrum is included of the reporter ions in the experiment, determined by the selection made in the Quantification parameter settings. This is an interactive spectrum, enabling zooming in and out using the scroll wheel of the mouse. Double-clicking will zoom out and reveal the entire MS3 spectrum.

9.5.9 LC/MS Snapshot

The LC-MS Snapshot displays a zoomed-in view of the precursor scan in the LC-MS heat map 2-D view. The peptide feature is highlighted in purple and the selected PSM marker is blue. The span of the orange line in the LC-MS Snapshot designates the isolation window.

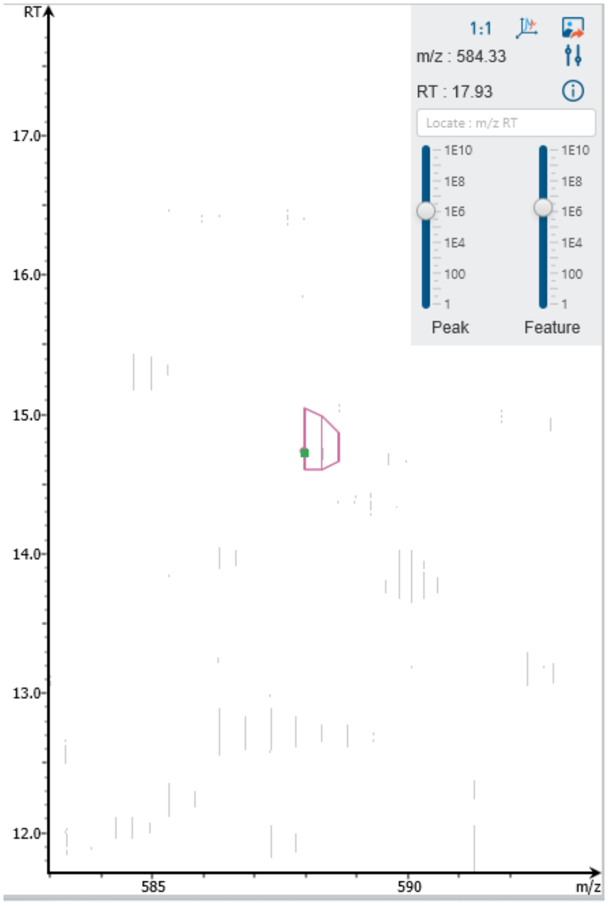

Users can change the view from the 2-D heat map to a 3-D intensity view by clicking the intensity view button in the upper right-hand corner of the LC-MS Snapshot. In addition, users can export the LC-MS heat map by clicking the export image button in the upper right-hand corner of the LC-MS Snapshot.

Expand the options dropdown by clicking to reveal a search option to specify a *m/z RT* (ensure there is a space between these two numbers) location to zoom into. There is also a Peak slider filter to filter out low intensity peaks and a feature area filter to filter out low area features.

9.6 TMT/iTRAQ Exporting

In Project View, double-click on Export to open the Export node.

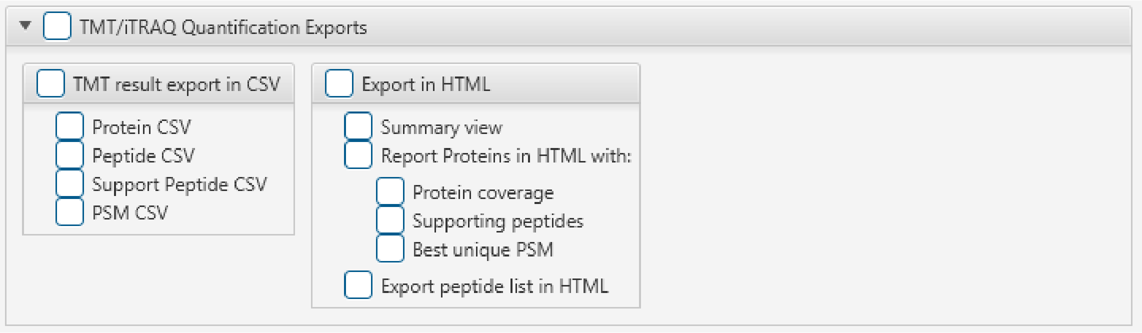

9.6.1 TMT/iTRAQ result - Exporting - comma separated values (CSV) format

There are several options for exporting the PEAKS Isobaric Labelling Quantification results in CSV format:

- **Proteins CSV:** The list of protein quantifications, filtered by the protein filters in the Summary view and will be saved to proteins.csv.
- **Peptide CSV:** A complete list of all quantified peptides that pass the peptide feature filters set on the "Summary" page and their associated details.
- **Support peptides CSV:** These are the supporting peptides of the quantified proteins and their associated details.
- **Feature Vector CSV:** All predicted peptide features and their associated quantification information. Both identified and unidentified peptide features are included. To include unidentified peptide features, set the Peptide Id Count equal to 0 in the Peptide feature filter.

9.6.2 TMT/iTRAQ result - Exporting - HTML format

Export in HTML: This will generate single or multiple HTML reports in the specified location.

The following exporting options are available:

- **Summary view:** The “Summary” view page will be saved as a summary.html file in HTML format in the specified location.
- **Protein coverage:** The coverage pane will be saved for each protein.
- **Supporting peptides:** A list of supporting peptides will be saved for each protein.
- **Best unique PSM:** The best unique PSM will be saved for each protein.
- **Export peptide list in html:** creates a separate HTML-containing peptide identifications.

An individual protein will have its own HTML output file, where the corresponding protein coverage, the supporting peptides, and the best unique PSM are gathered.

10. Stable Isotope Labeling by Amino acids in Cell culture (SILAC)

Precursor ion quantification with isotope labels at the MS level is one of the three quantification modes that are supported by the optional PEAKS Q module for PEAKS Studio. Typically, an unlabelled sample is mixed with a sample subjected to stable isotope labelling. The samples are then analyzed together in an LC-MS/MS experiment. The same peptide from different samples is recognized by a set of precursor ion peaks with similar retention time and mass differences equal to the expected difference caused by the stable isotope label. The ratio is calculated from the intensities of those peaks. PEAKS Q supports both user-defined labels and commercial quantification labels. PEAKS supports the analysis of Super-SILAC experiments that uses a mixture of SILAC-labeled cells as a spike-in standard for accurate quantification of other unlabeled or labeled samples.

10.1 Precursor Ion Quantification Analysis Workflow and Settings

Select the PEAKS Q workflow in the Workflow Selection step

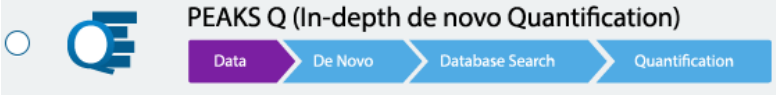

The following steps are the same as in the PEAKS DB workflow (as in section 6.1 Database Search Workflow and Parameter Settings) and is reiterated below.

Click on Data Refine to proceed through the workflow.

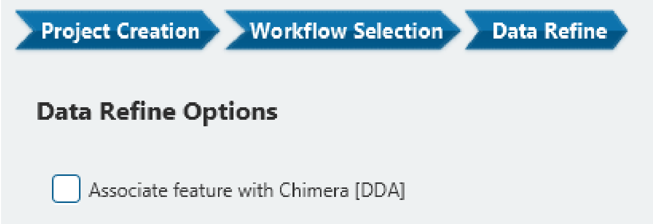

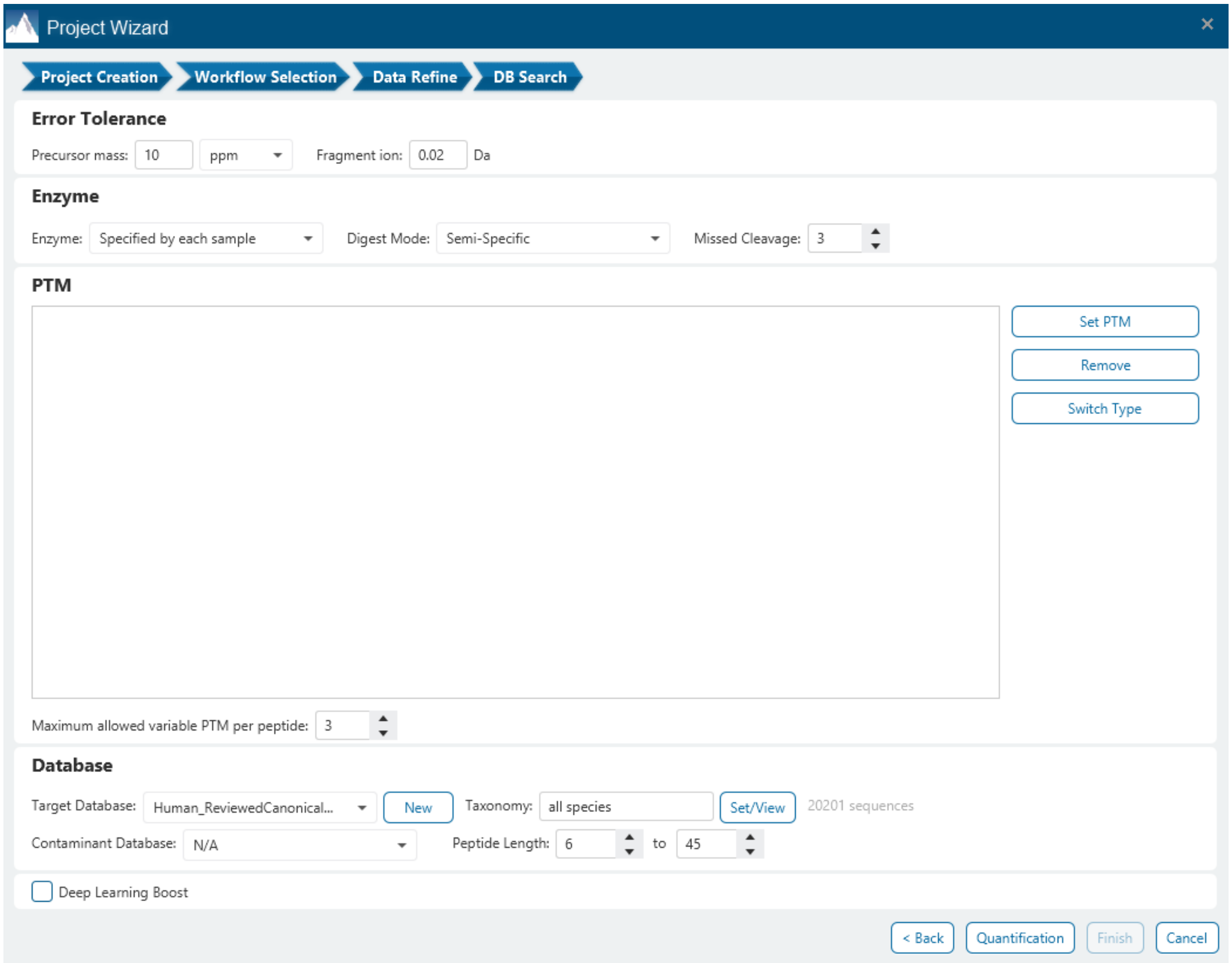

The following parameter settings are provided:

Precursor Mass Error Tolerance: The maximum mass difference between the peptide and the monoisotopic mass of the precursor ion.

Precursor Mass Tolerance unit: PEAKS generates peptides within the precursor mass error tolerance. Precursor mass error tolerance can be specified in either Daltons or ppm.

Fragment Mass Error Tolerance: PEAKS uses this value when scoring peptide sequences. PEAKS Studio considers a fragment ion to be matched if the calculated m/z is within the specified error tolerance. Units are Daltons.

Both precursor and fragment error tolerance parameters need to be set consistently with the mass accuracy of the instrument.

Enzyme Settings: PEAKS Studio digests the protein database *in silico* to generate peptide candidates. It is necessary to specify the enzyme for protein digestion from the Enzyme drop-down menu.

Note: When the selected dataset is digested with different enzymes, "Specified by each sample" allows samples to be analyzed separately using their respective enzymes specified during project creation.

Note: "None" is a special enzyme allowing non-specific cleavage at both ends of the peptide. It is the recommended option when no digestion enzyme was used or when the digestion enzyme exhibits a high degree of non-specificity.

Missed Cleavages: This specifies the number of missed cleavage sites allowed in a peptide.

Digest Mode: This specifies the type of in silico cleavages allowed at a peptide termini based on the method used for protein digestion. If "Specific" is selected, the specificity of the selected enzyme is strictly enforced at both termini of a peptide. "Semispecific" allows one terminus to disobey the enzyme specificity rules.

Note: If the enzyme is specified as "None", then no matter which mode is selected for "Digest Mode", the "None" enzyme digest rule will be applied.

PTM Settings: Click “Set PTM” to open the **PTM Options** dialog and specify the fixed PTMs and a few common variable PTMs expected in the experiment. Highlight PTMs and click on the button to place them as either Fixed or Variable PTM. From there, highlighted PTMs can be removed by clicking the “Remove” button. Clicking “Remove All” will reset the PTM options. Highlighting and clicking “Switch Type” will move the Fixed PTM in into the Variable PTM list and vice-verse. Click on “New” to create a new custom PTM. While a PTM is selected, click on “View” to see more details.

Note: Multiple PTMs can be highlighted by holding down the CTRL key or SHIFT key on the keyboard.

Note: For a specific residue, only one fixed modification is allowed.

Once the PTMs are selected, the maximum number of variable PTMs per peptide can be specified and a value of less than 4 is recommended.

Note: The use of variable modifications increases the size of the computational search space for the de novo sequencing algorithm. It is recommended not to select too many variable modifications in PEAKS de novo. See PEAKS PTM for alternate search options.

Target Database: Select a protein sequence database for the search. Select one from the list of databases that have been configured in PEAKS and set the taxonomy by clicking on Set/View, if applicable. To configure a new database, click on New next to the Target Database dropdown to open the Database Configuration dialog.

Contaminant Database: To search for contaminants, select a contaminant database from the list of databases that have been configured in PEAKS. Keep the selection as N/A if no contaminant database is required for the search.

Peptide Length: The length for a peptide can be set when creating an analysis, where the default peptide length range is between 6 and 45.

Deep Learning Boost: Check this option to use PEAKS enhanced deeplearning algorithm to better identify PSMs in the DB Search process.

Click on Quantification to proceed to the next step and select the Precursor Ion Quantification (eg. SILAC) option.

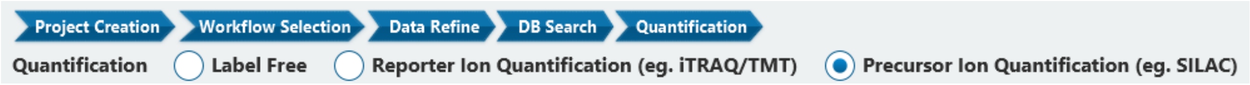

The following parameters are available:

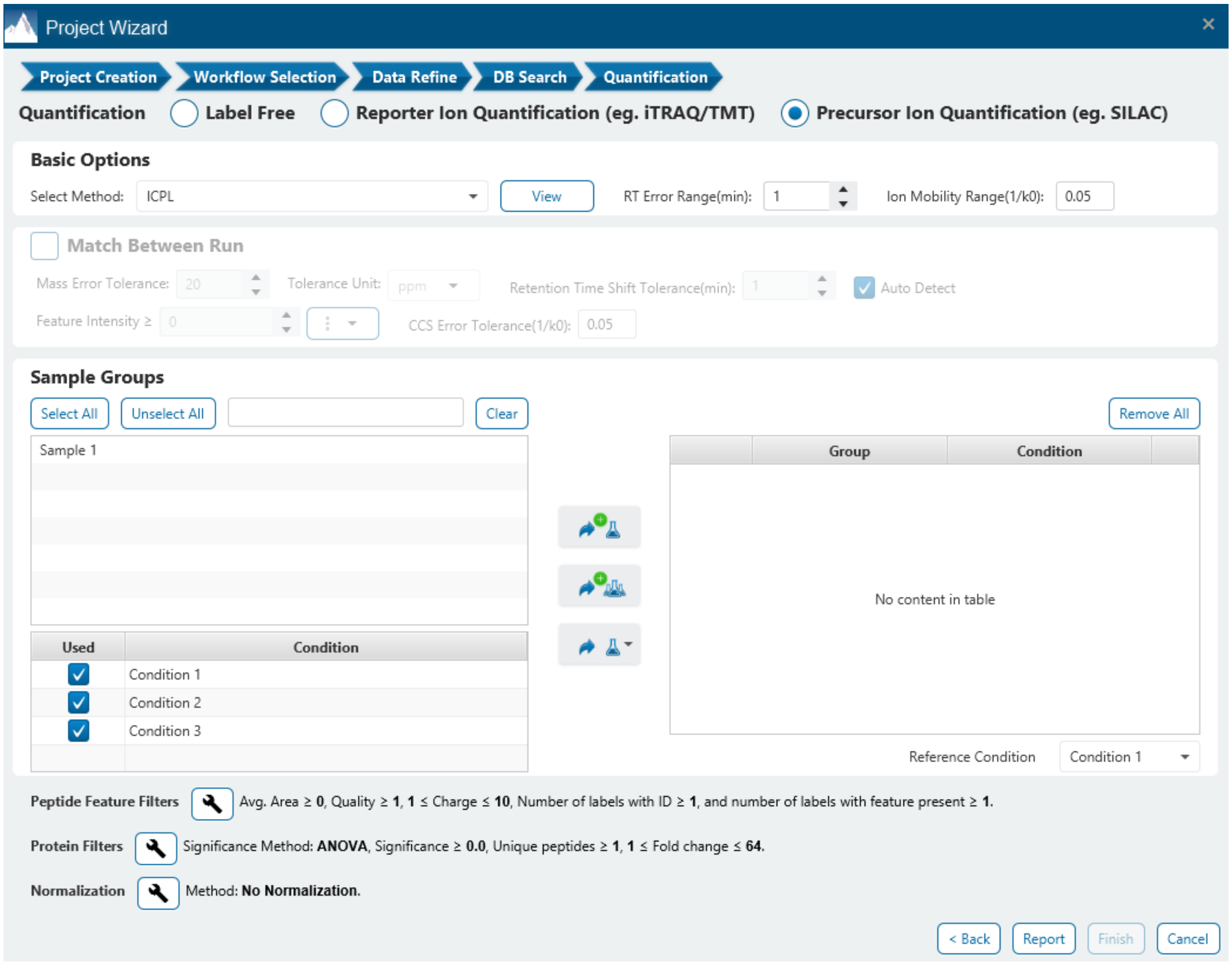

Select Methods: From the Select Methods drop-down menu, choose the appropriate quantification method used in the experiment, e.g. SILAC-2plex (R10,K8)

RT Error Range: The retention time shift tolerance is the maximum elution time difference that is considered for the quantification of an identified peptide after RT alignment.

Ion Mobility Range(1/k0): Only available for timsTOF data. The maximum 1/k0 difference that is considered for the quantification of an identified peptide after RT alignment.

Match Between Runs (MBR): Enabled for two or more samples. The options are described below.

Mass Error Tolerance: Controls the allowable difference in mass between the identified MS/MS scan in one sample versus features in other samples. This can be set similar to the precursor mass error tolerance used for database search.

Retention Time Shift: The maximum allowable difference between the retention time of the identified MS/ MS scan and the peptide feature considered for MBR.

Auto Detect: Let PEAKS Studio detect the optimal Retention Time Shift Tolerance for your data. An initial quantification run will be performed to determine the retention time shift trend. The optimal error tolerance will then be used for the full search.

Feature Intensity: Set a value for the minimum intensity a peptide feature must exhibit to be considered for MBR. By default, the integrated area under the curve of the peptide feature will be used to represent feature intensity.

CCS Error Tolerance (1/k0): This parameter is only enabled when the analysis contains timsTOF data. The maximum collisional cross section (CCS) differential between an identified MS/MS scan and peptide features in other samples to allow MBR.

Sample Groups: SILAC requires sample grouping. All available samples are listed in the left window. After selecting a sample, it can be added to a new group by clicking the button or to an existing group by clicking the button and selecting that option from the drop-down menu. If you select multiple channels, you can add each one individually to create multiple new groups by clicking the button. Samples and Groups can be removed by clicking the button next to each element. The names and colours for each group can be edited by clicking on the coloured square dropdown, respectively.

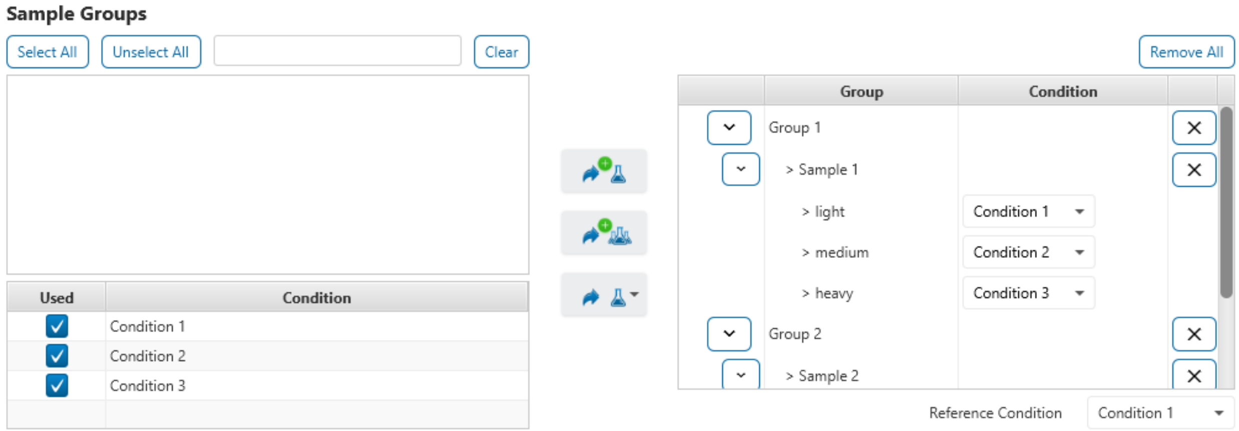

Conditions: The number of conditions depends on the quantification method selected. For example, SILAC-2plex(R10,K8) has 2 conditions and SILAC-3plex(R10,K8|R6,K4) has 3 conditions to study. Each label in a sample represents one unique condition. To remove a label from analysis, uncheck the corresponding condition. Condition names can be renamed.

Reference Condition: The condition which is the denominator of the calculated ratios. The reference condition can be chosen from the selected conditions only.

Click on the tool icon next to Peptide Feature Filters to open the **Peptide Feature Filters** dialog

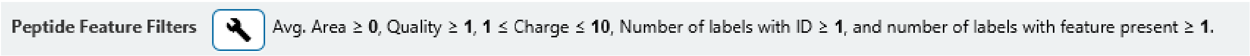

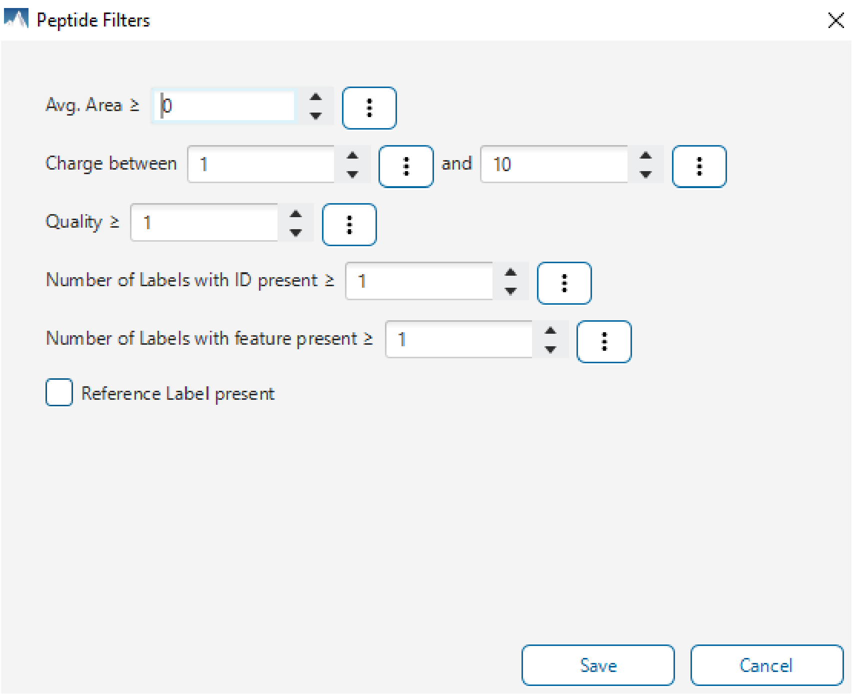

Average Area: The minimum average area of all peptide features associated with the peptide to be included in the result. Features with higher abundance have been shown to be more reproducible across replicates.

Charge between: Only peptides with feature vectors that all fall within this range will be used in protein quantification.

Quality: A quality value will be applied to each quantified peptide to represent the likely reproducibility of the quantification result. Set the minimum quality score cut-off. Factors that affect the quality score include m/z difference, RT difference, XIC shape similarity, and the feature intensities.

Number of labels with ID present: Only peptides with an identified MS/MS scan in the number of given labels will be included in the peptide table.

Number of labels with feature present: Only peptides with a predicted LC-MS peptide features in the number of given labels will be included in the peptide table.

Reference label present: Only quantifiable peptides in which the reference label peptide feature is present will be included in the peptide table if this is checked.

Click on the tool icon next to Protein Filters to open the Protein Filters dialog.

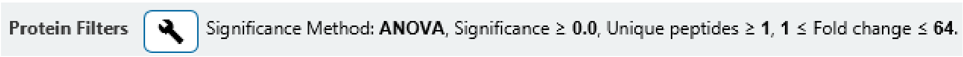

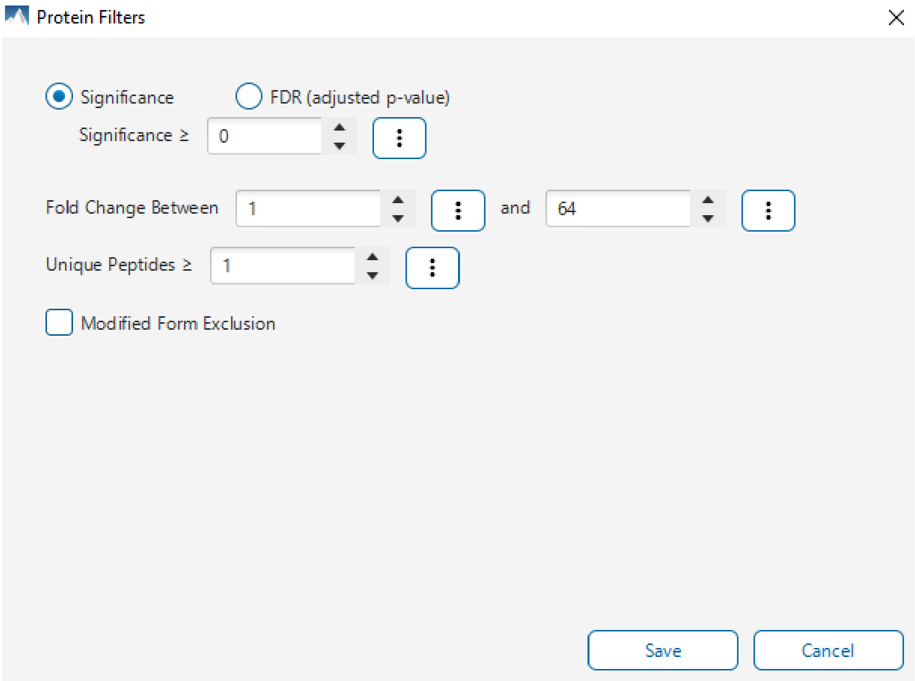

Significance: Only protein groups with a significance above this threshold will be listed in the “Protein” view. The significance score is calculated as the -10lg of the significance testing p-value. Significance is calculated using welch's ANOVA.

FDR% (adjusted p-value): Adopt the multiple hypothesis testing Benjamin-Hochberg method to adjust the p-value to the given false discovery rate. Only protein groups with significance scores passing the calculated FDR will be listed in the "Protein" view. Either this or "Significance" can be selected to set a significance threshold.

Fold Change: The required magnitude of change of abundance between conditions. The minimum value is one because the fold change is calculated as the magnitude of change where the lower value is always the denominator.

Unique Peptides: Only protein groups with unique peptides at or above this number will be listed in the "Protein" view. Peptides are considered unique if they are only matched to one protein group.

Modified Form Exclusion: When checked, peptides that are identified as both a modified and unmodified form will be excluded from the protein ration calculation. This does not apply to the quantification labels. When a peptide is split between an unmodified and modified form, the ratio of both forms may differ from the protein ratio. Therefore, including modified peptides in the protein ratio calculation might lead to incorrect results. By checking this box, if a peptide has modified and unmodified forms, both will not be used as candidates, if a peptide only has one modified form, it is still a candidate, but if a peptide has more than one modified form, all forms will not be used as candidates.

Click on the tool icon next to Normalization to open the Normalization dialog.

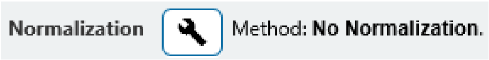

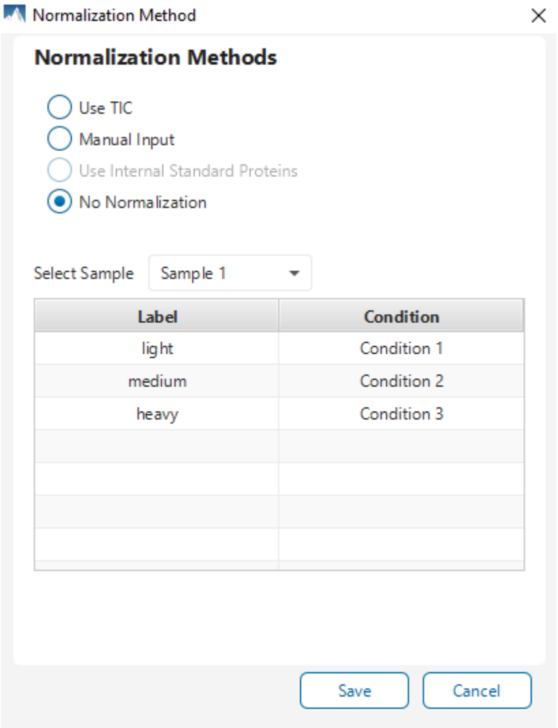

Use TIC: Using the total intensity of all quantifiable peptides from each condition, a global ratio is created. The global ratio is used as a normalization factor for all proteins and peptides.

Manual input: This option performs the same default calculation as auto normalization ("Use TIC") but also allows for the manual change of each sample's expected ratios. This should be used whenever the expected ratio between conditions isn't 1:1.

Use internal standard proteins: This option displays a list of identified proteins, which can be selected to create a normalization factor. A search bar is included to enable the quick location of select proteins.

No Normalization: All samples are automatically assigned Factors of 1.0.

Click on “Report” to proceed to the final step. Report filters can be set here which will filter the final result.

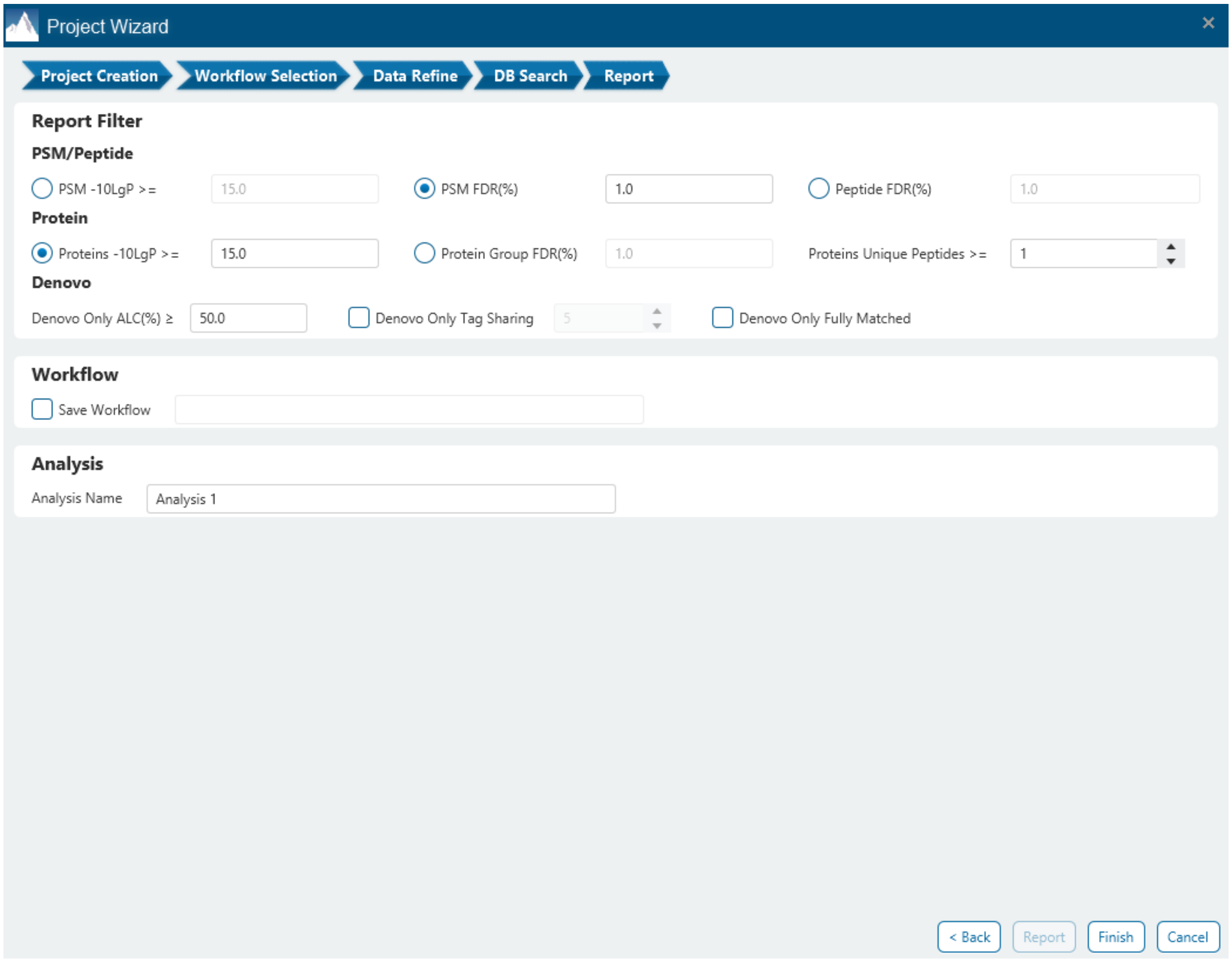

These filters are provided:

PSM/Peptide: this section is a mandatory section to filter out some results under the user-defined filter. Any changes to the filter will create a new analysis with old parameters and update the results and statistics.

PSM -10lgP: The PEAKS peptide score (-10lgP) is calculated for every peptide-spectrum match (PSM) reported by PEAKS DB, PEAKS PTM, and SPIDER. The score is derived from the p-value that indicates the statistical significance of the peptide-spectrum match. A peptide may be matched to many spectra, resulting in multiple PSMs. In that case, the peptide's score is calculated as the maximum among all PSM scores. For details of the scoring algorithm, please refer to the publication, *"PEAKS DB: De Novo sequencing assisted database search for sensitive and accurate peptide identification ", Mol Cell Proteomics, 2011 Dec 20.* A minimum requirement can be set and all identifications must pass this filter.

PSM FDR (%): The PSM filter can also be set to use the false discovery rate. FDR is estimated using a "decoy-fusion" approach.

Peptide FDR (%): The Peptides can also be set to use the false discovery rate. Peptides are scored based on their top PSM and using a “decoy-fusion” approach, the result is reported at that FDR.

Note: Decoy fusion is an enhanced target-decoy method for result validation with FDR. Decoy fusion appends a decoy sequence to each protein as the "negative control" for the search. See BSI's web tutorial (http://www.bioinfor.com/fdr-tutorial/) for more details.

De Novo ALC (%): This filter controls the minimum ALC score required for display in the de novo only result. A default of 50% is set.

Denovo Only Tag Sharing: The number set here corresponds to the length of de novo only tags to be shown in the result Protein coverage. For de novo only peptides that are not fully matched, they must share a tag length with of minimum of this parameter with the protein residue to be displayed.

Denovo Only Fully Matched: Checking this option will show the de novo only peptides in the Protein coverage regardless of what tag sharing parameter is set if the entire de novo only sequence matches the protein residue.

In the Report step, there also is the option to specify an Analysis Name (which can be later renamed) and/or decide to save the Workflow to quickly re-use in future analyses.

10.2 Understanding SILAC Results

Once completed, a SILAC result node will be added to the project tree. Double-click on this 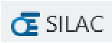

node to open the Precursor Ion Quantification result that contains three tabs as described below:

Summary: The outline of the isobaric labelling search results with statistics. This is the place to examine the overall performance of the experiment and adjust filters. If there are too many proteins, the heatmap will not show on the summary page.

Proteins: The quantified proteins with a list of supporting peptide features for each protein.

Peptides: The quantified peptides are listed in a table. Extracted ion chromatogram (XIC) and supporting peptide features are also displayed for each peptide.

10.3. SILAC - Summary View

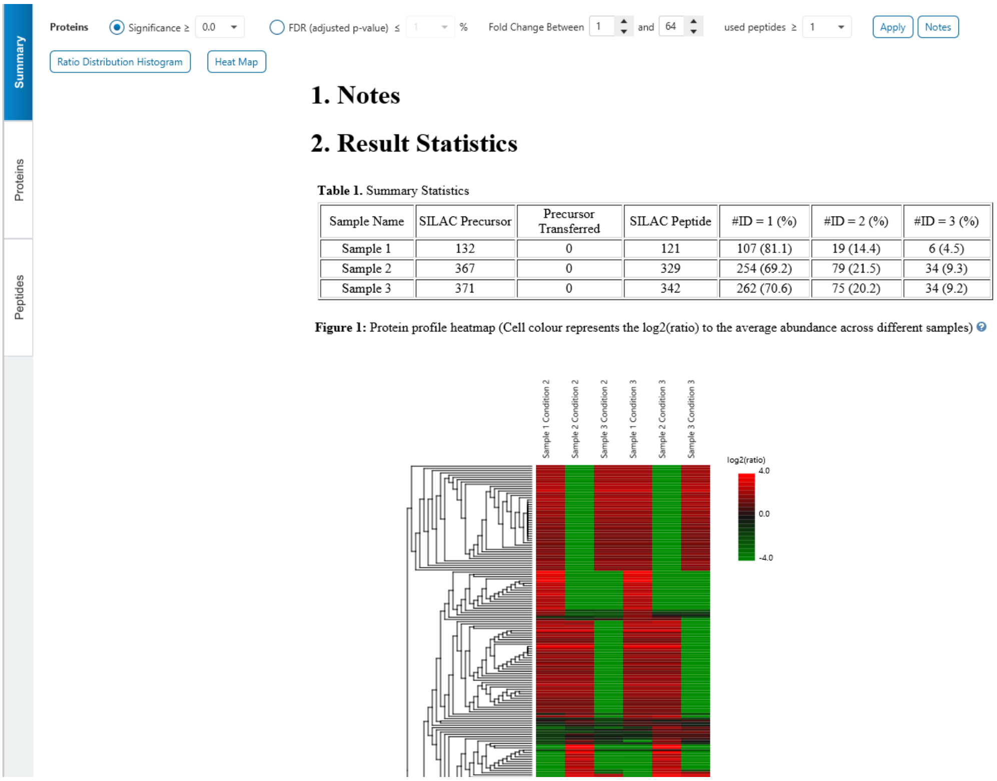

1. Notes

2. Result Statistics

Table 1. Summary Statistics

Sample Name	SILAC Precursor	Precursor Transferred	SILAC Peptide	#ID = 1 (%)	#ID = 2 (%)	#ID = 3 (%)
Sample 1	132	0	121	107 (81.1)	19 (14.4)	6 (4.5)
Sample 2	367	0	329	254 (69.2)	79 (21.5)	34 (9.3)
Sample 3	371	0	342	262 (70.6)	75 (20.2)	34 (9.2)

Figure 1: Protein profile heatmap (Cell colour represents the log2(ratio) to the average abundance across different samples)

10.3.1 Summary View - Protein Filters

Filter settings used in the screenshot above means that no protein filter is applied, thus giving all quantifiable protein.

Alternately, parameters can be adjusted for additional analysis by updating the protein filters above.

Significance: Only protein groups with a significance above this threshold will be listed in the **Protein View**. The significance score is calculated as the -10log10 of the significance testing p-value. PEAKS provides ANOVA significance testing methods A significance score threshold of 20 is recommended, which equals to a significance testing p value of 0.01. Either this or “FDR (adjusted p-value)” can be selected to set a significance threshold.

Fold Change: Only proteins at or above this fold change threshold will be listed in the Protein View.

Used Peptides: Only proteins with at least this many number of used peptides will be listed in the Protein View.

Click on [Apply] to apply these filters changes to the result. This will update charts on the Summary View and will update the Protein table accordingly.

10.3.2 Summary View - Notes

Click on [Notes] to open the Notes dialog and save information about the result and saved in section 1. Notes on the Summary page.

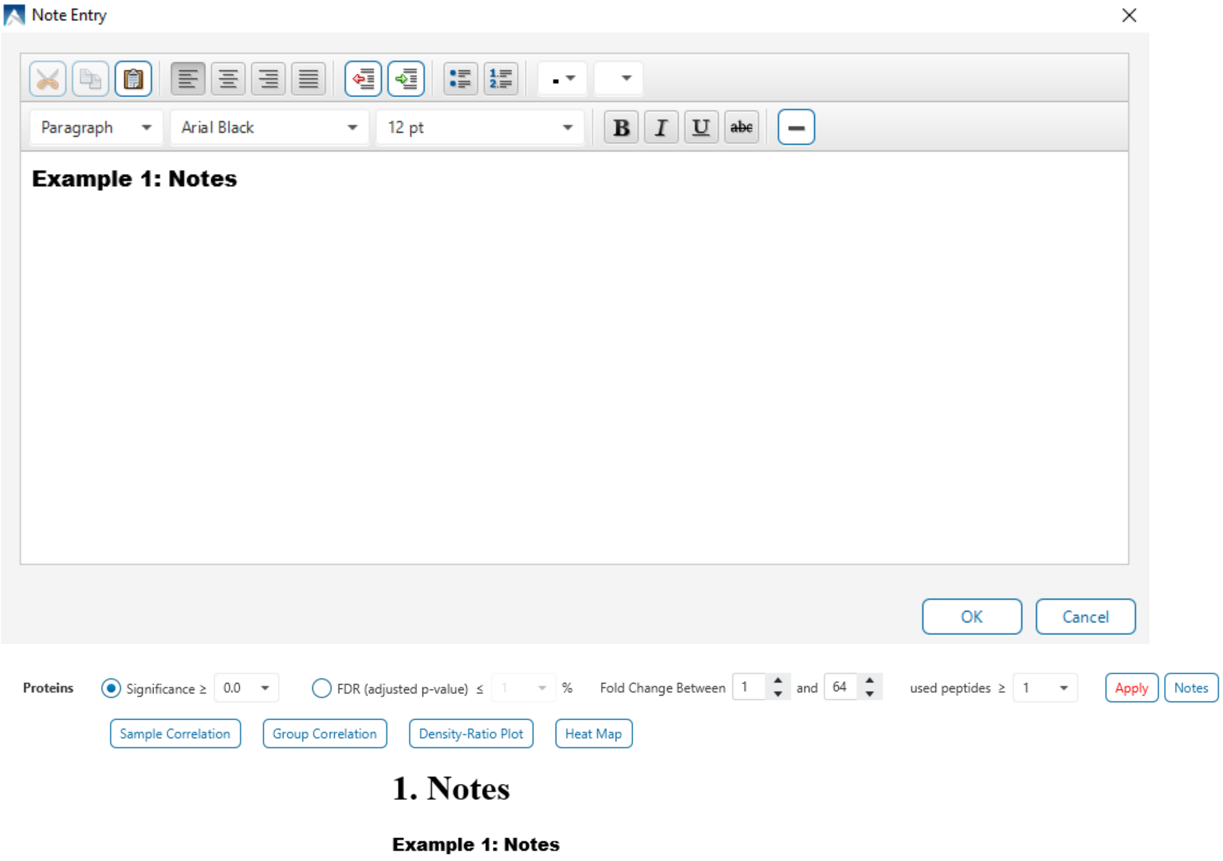
Note Entry
Paragraph
Arial Black
12 pt
Example 1: Notes
OK
Cancel
Proteins
Significance ≥
0.0
FDR (adjusted p-value) ≤
%
Fold Change Between
1
and
64
used peptides ≥
1
Apply
Notes
Sample Correlation
Group Correlation
Density-Ratio Plot
Heat Map
1. Notes
Example 1: Notes

10.3.3 Summary View - Heat Map

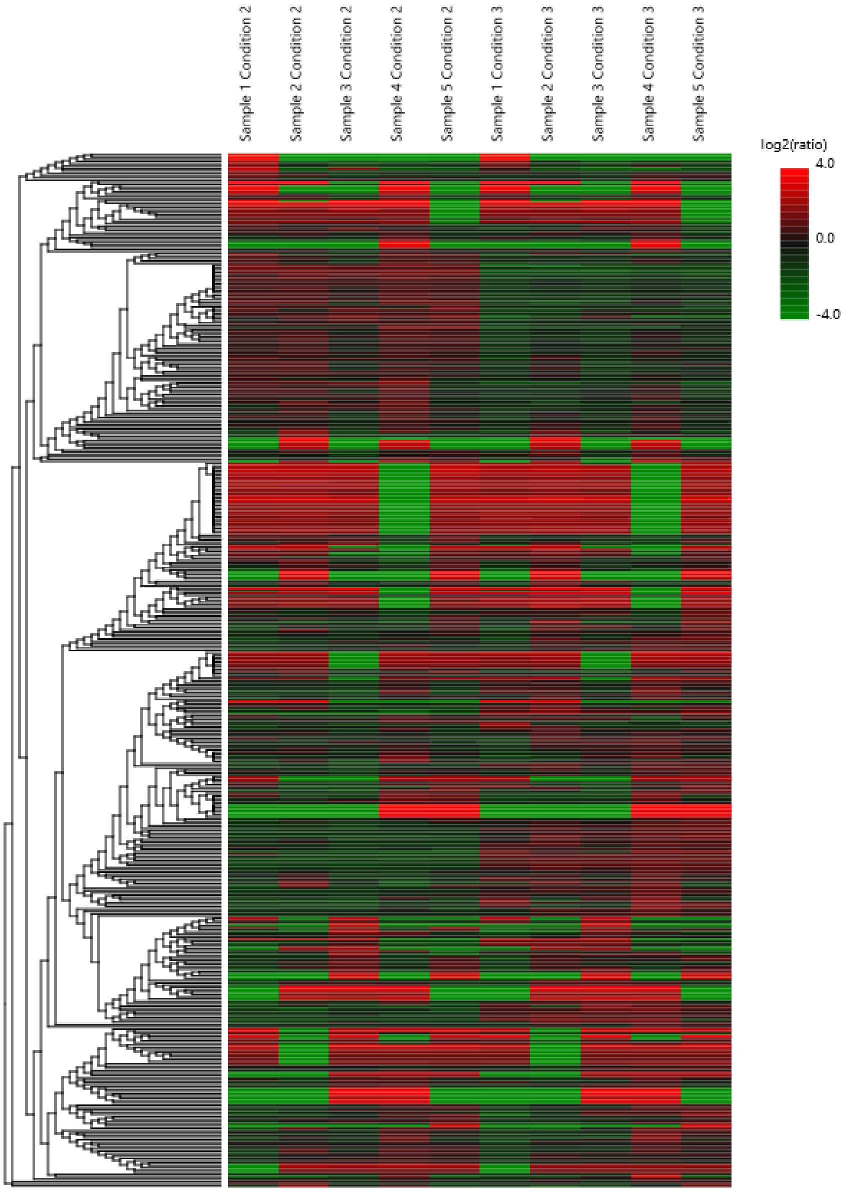

The heat map displays the protein groups that passed the filters. The relative protein abundance is represented as a heat map of the representative proteins of each protein group. These representative proteins are clustered if they exhibit a similar expression trend across the samples. The hierarchical clustering is generated using a neighbour-joining algorithm with a Euclidean distance similarity measurement of the log2 ratios of the abundance of each sample relative to the average abundance. Similarly, the conditions in different samples are clustered if they exhibit a similar expression trend across the protein groups. An interactive protein profile heat map is opened in a new window when the Heat Map button is clicked options.

Mouse over the dendogram in a section of the heatmap and click on it to open the Variation Trend chart.

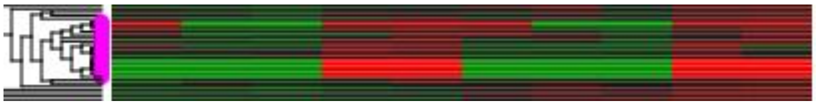

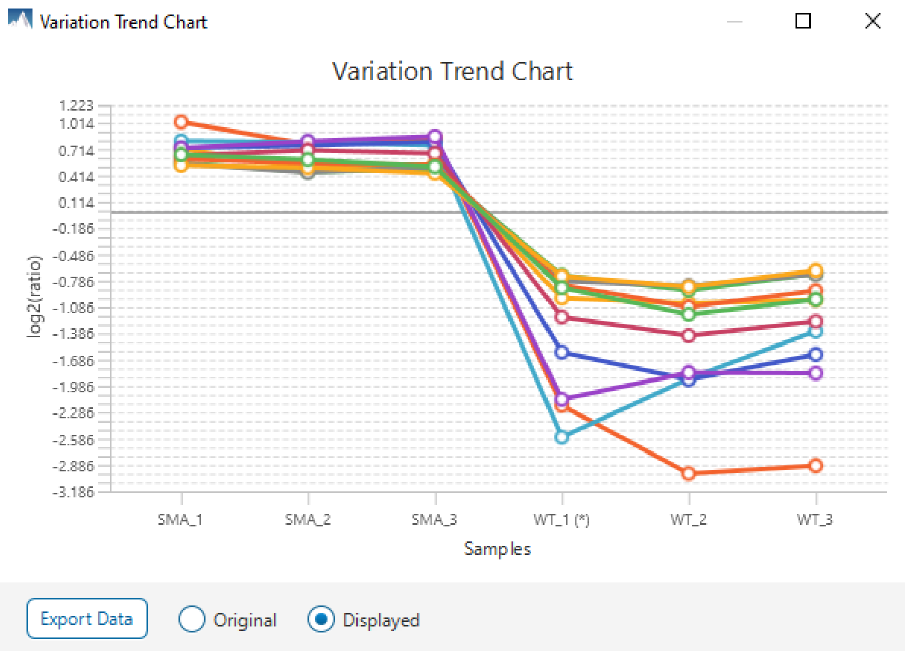

The Variation Trend chart displays the expression level of the selected proteins in the samples.

Mouse over points on the variation trend chart to see a tooltip of the protein, sample, and log2ratio.

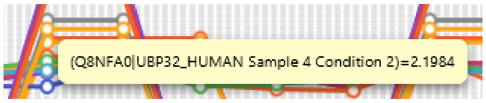

Click on the Export Data button to export the variation trend chart data in a text file format. When Displayed is selected, the log2 ratios will be in the export. When Original is selected, the original ratio values will be in the export instead.

10.3.4 Summary View - Volcano plot

Figure 2. volcano plot combines a statistical test with the magnitude of the change enabling quick visual identification of those data-points that display large-magnitude changes and that are also statistically significant. A separate volcano plot will be shown for each condition relative to the reference condition.

Horizontal broken grey line: The selected significance threshold

Vertical broken grey lines: The selected fold change thresholds

Plotting points in this way results in two regions of interest in the plot: those points that are found towards the top of the plot that are far to either the left or the right side. These represent values that display large magnitude fold changes (hence being left or right of center) as well as high statistical significance (hence being towards the top).

Figure 2. The volcano plot for proteins.

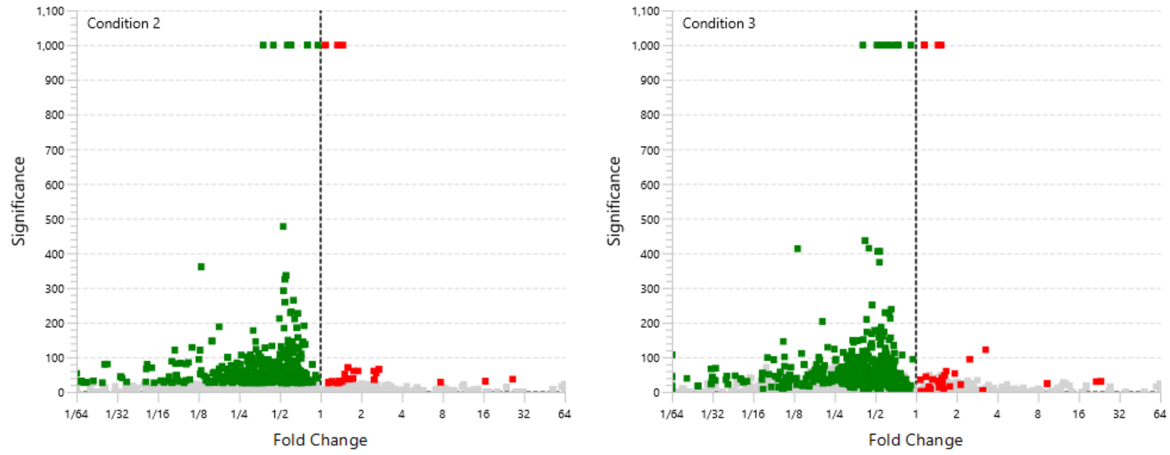

In this figure, the ratio is the group ratio set with respect to the base group. If more than two groups are present, the max ratio is used instead.

The Protein View contains an interactive volcano plot that can be hidden or displayed. Markers for the proteins that are above the set significance threshold will be displayed in colour. Scrolling the cursor over a marker will highlight it and, subsequently, clicking on this marker will highlight the protein in the interface.

10.3.5 Summary View - Ratio Distribution Histogram

Click on [Ratio Distribution Histogram] button in the Summary header to open the Ratio Distribution Histogram.

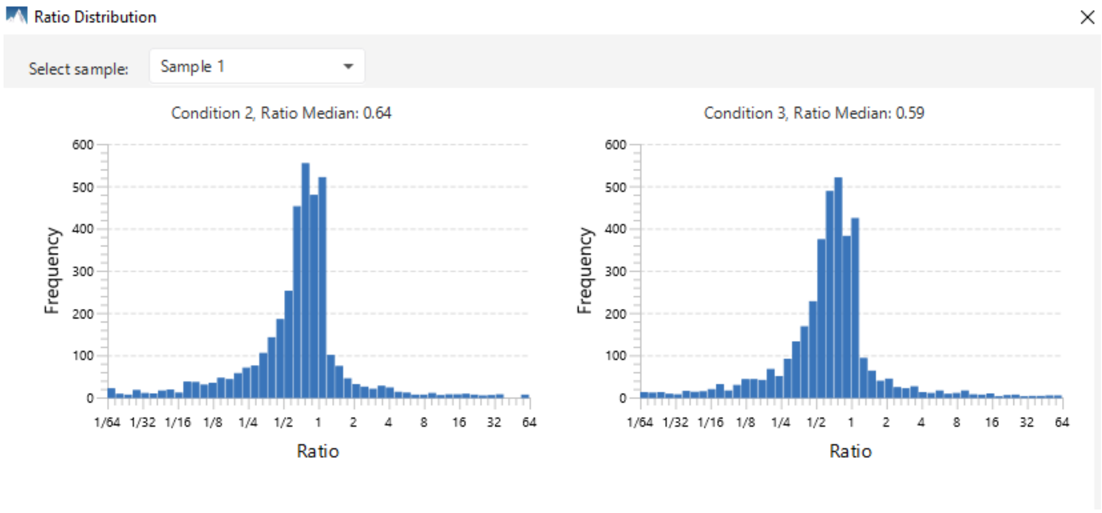

Select from the sample dropdown to view the ratio distribution of each sample.

For every condition, the distribution of ratios relative to the reference condition is plotted. Right-click on the image to export the chart.

10.3.6 Summary View - Filtration parameters and statistics

Tables 2 and 3 list the filtration parameters and filtered results, respectively.

Table 2. Result filtration parameters.

FDR(adjusted p-value)	≤ 1%
Min Fold Change	≥ 1
Max Fold Change	≤ 64
Used peptides	≥ 1

Table 3. Statistics of filtered result.

SILAC Peptide	6478
All Peptide sequences	10618
Features	52487
SILAC Precursor	17779
Protein groups	448

10.3.7 Summary View - Search parameters

Table 4 lists the parameters as set in the workflow.

Table 4. Search Parameters

Search Engine Name: PEAKS
Quantification type: ICAT/SILAC
Silac Method: SILAC-3plex (R10,K8|R6,K4)
Retention Time Tolerance: 1.00
Enable Match Between Run: No
5 samples in 1 group
Group 1:
  Sample 1:
    Light: Condition 1
    Medium: Condition 2
    Heavy: Condition 3
  Sample 2:
    Light: Condition 1
    Medium: Condition 2
    Heavy: Condition 3
  Sample 3:
    Light: Condition 1
    Medium: Condition 2
    Heavy: Condition 3
  Sample 4:
    Light: Condition 1
    Medium: Condition 2
    Heavy: Condition 3
  Sample 5:
    Light: Condition 1
    Medium: Condition 2
    Heavy: Condition 3
Conditions: Condition 1 Condition 2 Condition 3
Reference Condition: Condition 1
Peptide Feature Filter:
    Avg. area ≥ 0.0
    Quality ≥ 1.0
    Charge between: [1 - 10]
    Number of labels with ID present ≥ 1
    Number of labels with feature present ≥ 1
Protein Filter:
    Significance method ANOVA
    FDR ≤ 0.01
    Fold change between: [0.0 - 64.0]
    Unique peptides ≥ 1
Normalization method: No Normalization

10.4 SILAC - Protein View

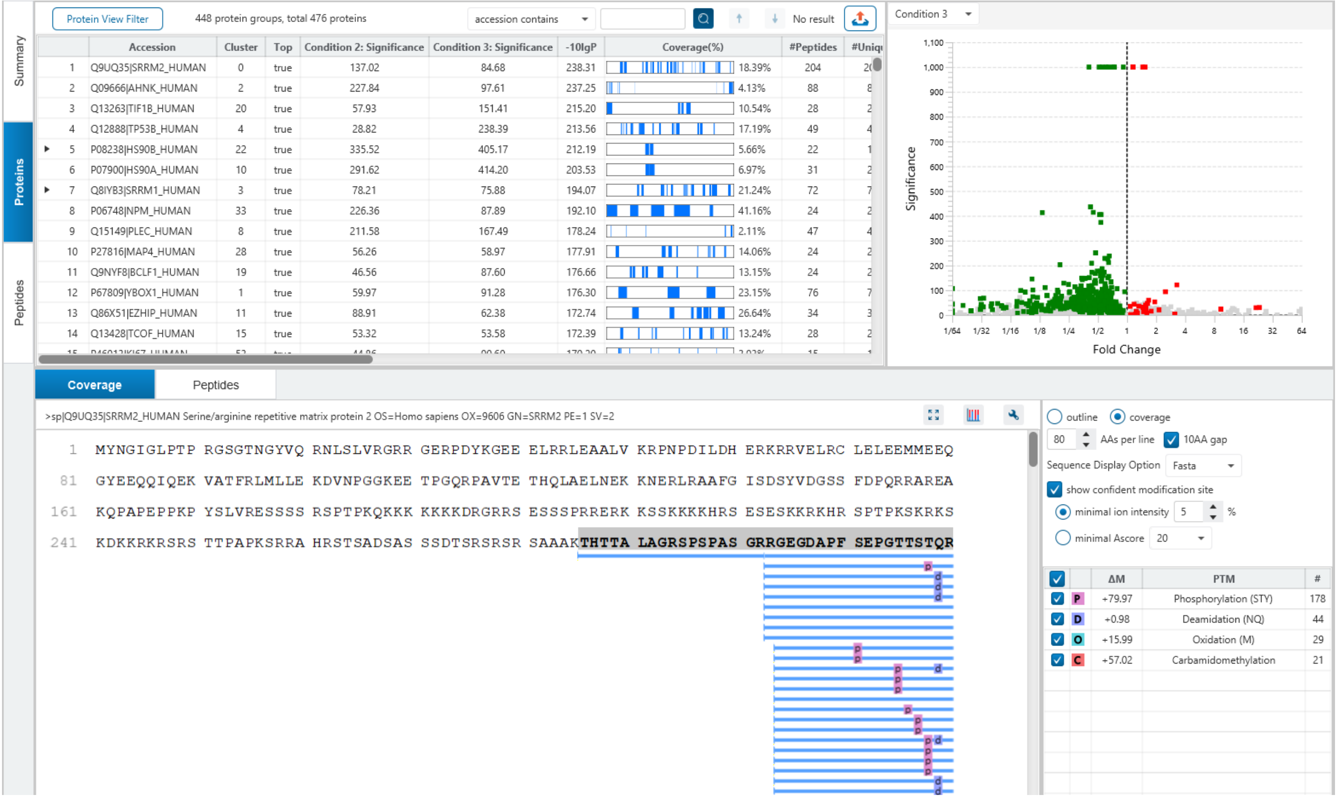

The **Proteins View** lists all the quantified proteins present in the sample, characterizes each protein at the amino acid level, and lists the supporting peptide features of each protein. It has six components:

Protein Table: Lists all the quantified proteins present in the sample.

Protein View Filters: Allows filtering the protein table to select specific proteins.

Protein Volcano Plot: Click on a marker that is above the significance threshold (the horizontal dashed line) set in the Summary View. Upon selection, the corresponding protein in the Protein Table will be highlighted.

PTM Profiling: Click on 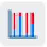 to open PTM profiling.

Coverage: Characterizes the protein sequence at the amino acid level. All the PTMs occurred on the protein sequence will be displayed together with the MS/MS data supporting the inference.

Peptides: Lists the supporting peptides identified from the selected proteins in the protein table.

10.4.1 Proteins View - Protein Table controls

At the top of the Proteins View, there are the options to filter, search, and export the protein table.

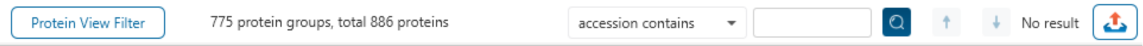

10.4.1.1 Protein Table controls - Protein View Filters

Click on the 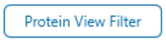 button to open the Protein View Filter.

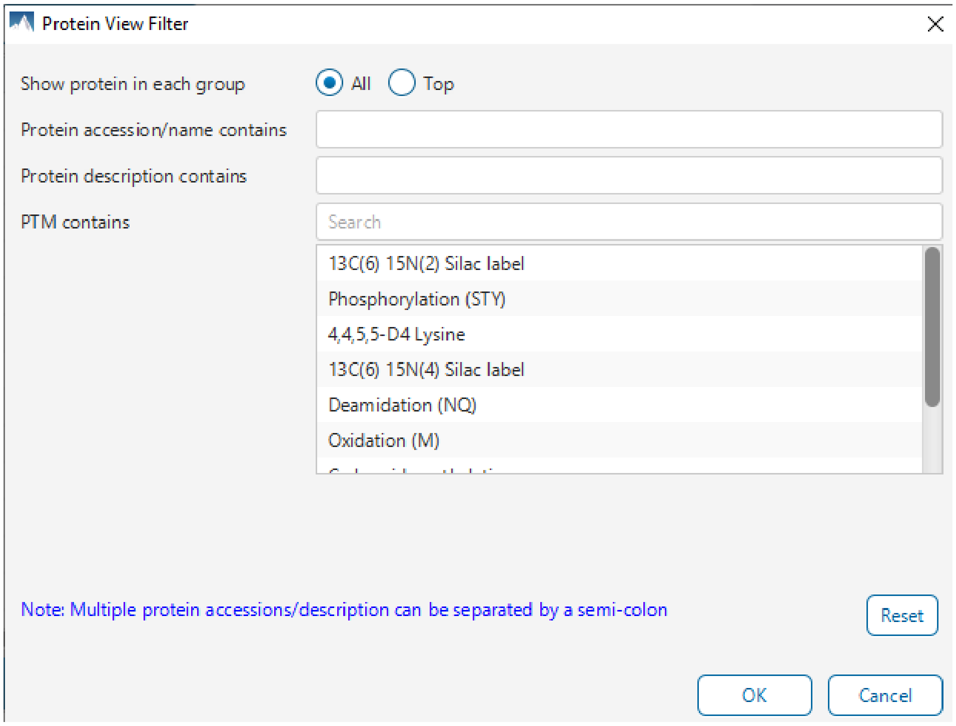

The following options are available for filtering the protein table:

Show All or Top proteins in each group: Adjust the protein list based on Protein grouping, top is selected by default. Proteins are grouped based on parsimony.

- **Top:** Show only the top proteins in each group. These proteins are supported by the most unique peptides in the group. Proteins in the group that share a subset of the unique peptides that support the top protein (subproteins) will not be displayed.
- **All:** Show all proteins in each group. Proteins are grouped together if they are supported by the same set or a sub-set of the top protein in the group. If a protein is supported by a peptide not supporting the top protein it will be added to a new group.

Protein accession/name contains: Restrict the protein table to only display protein IDs which contain the entered text in its accession/name.

Protein description contains: Only proteins with descriptions that contain the entered text will be included in the protein table if text is entered in this field.

Note: In these above two filters, multiple entries can be separated by a semi-colon.

PTM contains: Filter protein results by the presence of selected PTMs. Proteins that are supported by peptides containing modifications that match the selected PTMs will be included. If multiple PTMs match the modification name, they will all be included in the table.

Click on the 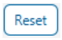 button to remove all filters.

Click on 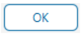 to apply the Protein View Filter to the Protein table.

Click on Cancel to discard changes and close the dialog.

10.4.1.2 Protein Table controls - Protein count

At the top of the protein table shows the count of protein groups and number of proteins in the table based on the current Protein View Filters.

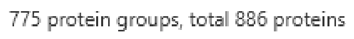

10.4.1.3 Protein Table controls - Protein Table Search Function

An easy-to-use search function is available for the protein table. The protein accession or description substring can be searched for, and all search results can be navigated through with the Up and Down arrows.

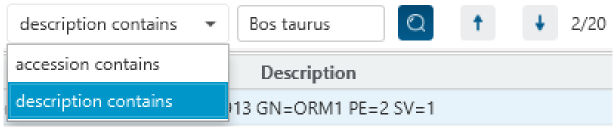

Protein accession/name contains: Restrict the protein table to only display protein IDs which contain the entered text in its accession/name.

Protein description contains: Only proteins with descriptions that contain the entered text will be included in the protein table if text is entered in this field.

10.4.1.4 Protein Table controls - Protein Table Export

Click on the button to export the contents of the current table. This considers the Protein View Filters, the sorting applied to the table and is consistent with the view. There is the option to export the Protein table and/or the Supporting peptide table in CSV format.

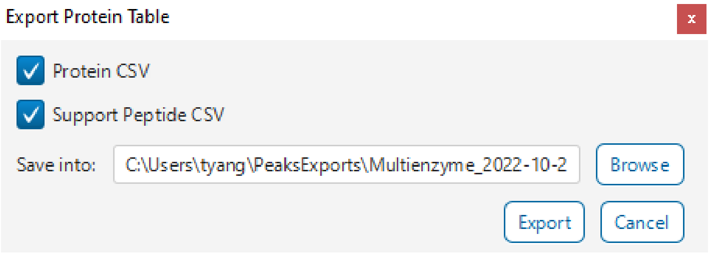

10.4.2 Proteins View - Protein Table

Each row in the table represents a group of proteins that are supported by a common set of peptides. To expand the group, click the right arrow button at the left.

	Accession	Cluster	Top	Condition 2: Significance	Condition 3: Significance	-10lgP	Coverage(%)	#Peptides	#Unique	PTM	Condition 2
1	Q9UQ35|SRRM2_HUMAN	0	true	137.02	84.68	238.31	18.39%	204	204	P D O C	
2	Q09666|AHNK_HUMAN	2	true	227.84	97.61	237.25	4.13%	88	88	P D O	
3	Q13263|TIF1B_HUMAN	20	true	57.93	151.41	215.20	10.54%	28	28	P D O A	
4	Q12888|TP53B_HUMAN	4	true	28.82	238.39	213.56	17.19%	49	49	P D O C	
▸ 5	P08238|HS90B_HUMAN	22	true	335.52	405.17	212.19	5.66%	22	15	P	
6	P07900|HS90A_HUMAN	10	true	291.62	414.20	203.53	6.97%	31	24	P	
▸ 7	Q8IYB3|SRRM1_HUMAN	3	true	78.21	75.88	194.07	21.24%	72	72	P D	
8	P06748|NPM_HUMAN	33	true	226.36	87.89	192.10	41.16%	24	24	P D O C A	
9	Q15149|PLEC_HUMAN	8	true	211.58	167.49	178.24	2.11%	47	47	P D O	
10	P27816|MAP4_HUMAN	28	true	56.26	58.97	177.91	14.06%	24	24	P D O C	
11	Q9NYF8|BCLF1_HUMAN	19	true	46.56	87.60	176.66	13.15%	24	23	P D	
12	P67809|YBOX1_HUMAN	1	true	59.97	91.28	176.30	23.15%	76	76	P D	
13	Q86X51|EZHIP_HUMAN	11	true	88.91	62.38	172.74	26.64%	34	34	P D C	
14	Q13428|TCOF_HUMAN	15	true	53.32	53.58	172.39	13.24%	28	28	P D O	
[illegible]	[illegible]	[illegible]	[illegible]	[illegible]	[illegible]	[illegible]	[illegible]	[illegible]	[illegible]	[illegible]	

The columns in the Protein Table are:

- **Accession:** The accession number of the protein as seen in the FASTA database.
- **Cluster:** A unique identifier that classifies which protein group the protein belongs to.
- **Top:** Indicates whether this protein is a top protein.
- **Condition Significance:** The significance for each condition
- **-10lgP:** The -10lgP score of the top PSM.
- **Coverage (%):** The percentage of the protein sequence that is covered by supporting peptides. The coverage is visualized by a colored bar. Light blue blocks indicate the parts of the sequence covered by low-confidence peptides. Dark blue blocks indicate the parts covered by high-confidence peptides. This is the total coverage including results from all samples.
- **#Peptides:** The number of high-confidence supporting peptides.
- **#Unique:** The number of high-confidence supporting peptides that are mapped to only one protein group. Unique peptides with same sequence but different modifications are only counted once in this number.
- **PTM:** The identified modifications displayed with color-coded icons.
- **Condition Profile:** The protein abundance among the samples is depicted as a heat map. Hold the cursor on a profile to view the sample channels, abundances, and ratios with respect to the base sample.
- **Condition Ratio:** The ratio columns show the peptide abundance in each condition for all groups. A peptide's abundance in each condition is the sum of all supporting feature vectors abundance within the filters. Then the peptide abundance in each condition is compared to the reference condition to obtain the peptide condition ratio in each sample separately. The peptide condition ratio of a group is computed by calculating the median from the peptide condition ratios in the samples in that group.
- **Avg. Mass:** The protein mass calculated using the average mass.
- **Description:** The protein's header information as seen in the FASTA database.

Note: For #Peptides and #Unique, two peptides with the same starting and ending positions in the protein are counted as one, regardless of their PTM forms. This is to follow the MCP (Molecular & Cellular Proteomics) guidelines.

10.4.3 Proteins View - Coverage Pane

The Protein Coverage view visually maps the supporting peptides and de novo tags to the protein selected in the Protein table. It also shows all identified sites with modifications or mutations to assist with protein characterization at the amino acid level. The "Protein Coverage" view is composed of three major components - Protein Sequence Coverage, Coverage Control Panel, and Protein Tools.

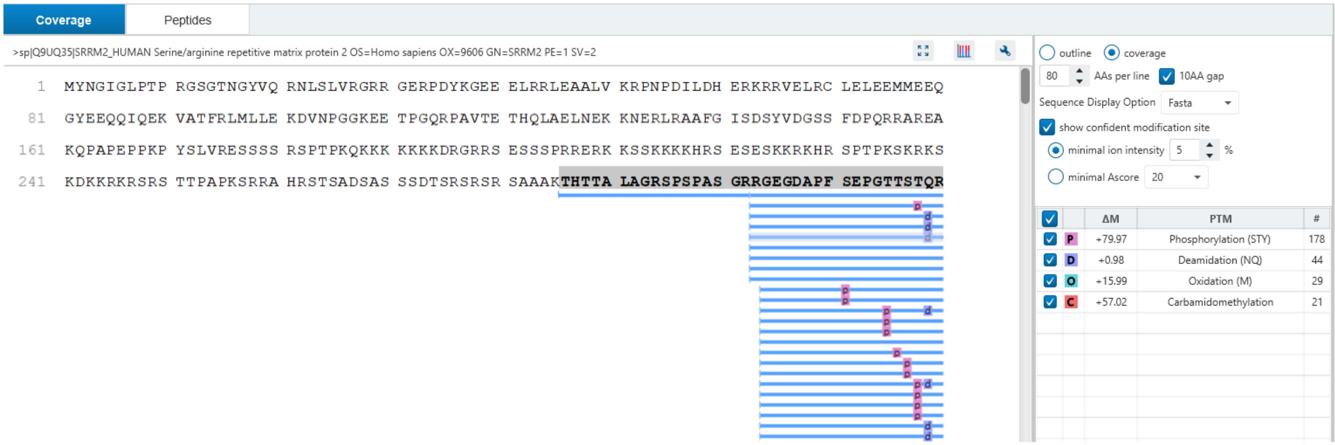

10.4.3.1 Protein Sequence Coverage

This area visualizes the coverage of the protein sequence.

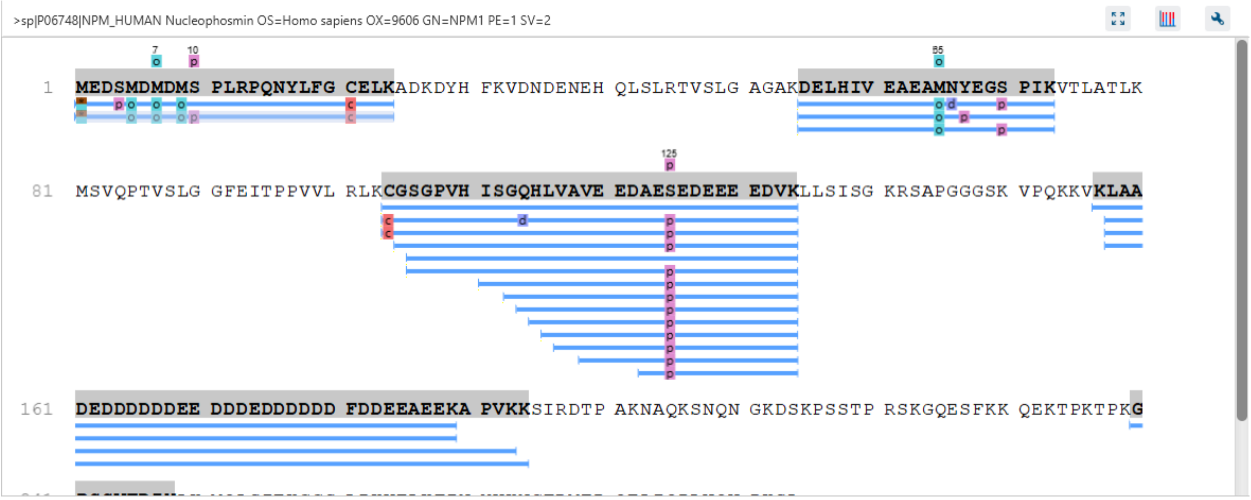

Regions in the protein sequence that are covered by supporting peptides are displayed in bold font with a grey background. Confident modifications and mutations identified in supporting peptides are displayed as icons above the protein sequence. Modifications are represented by colored icons with the first letter of its modification name. If a residue is modified by more than one modification in the same supporting peptide, "*" is used instead of a letter. The header information of the protein is shown on the top of the protein sequence.

The supporting peptides and matched de novo tags can be shown as colored bars under the protein sequence.

Placing the cursor over a bar shows a tooltip with detailed information of the peptide.

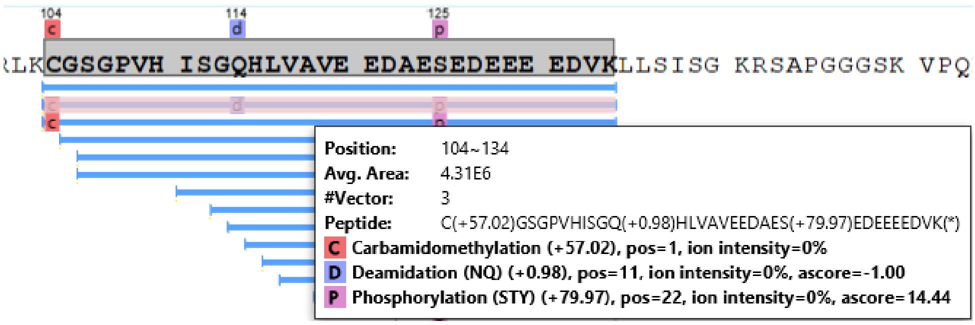

Left clicking on a bar shows the details from which the supporting peptide is identified.

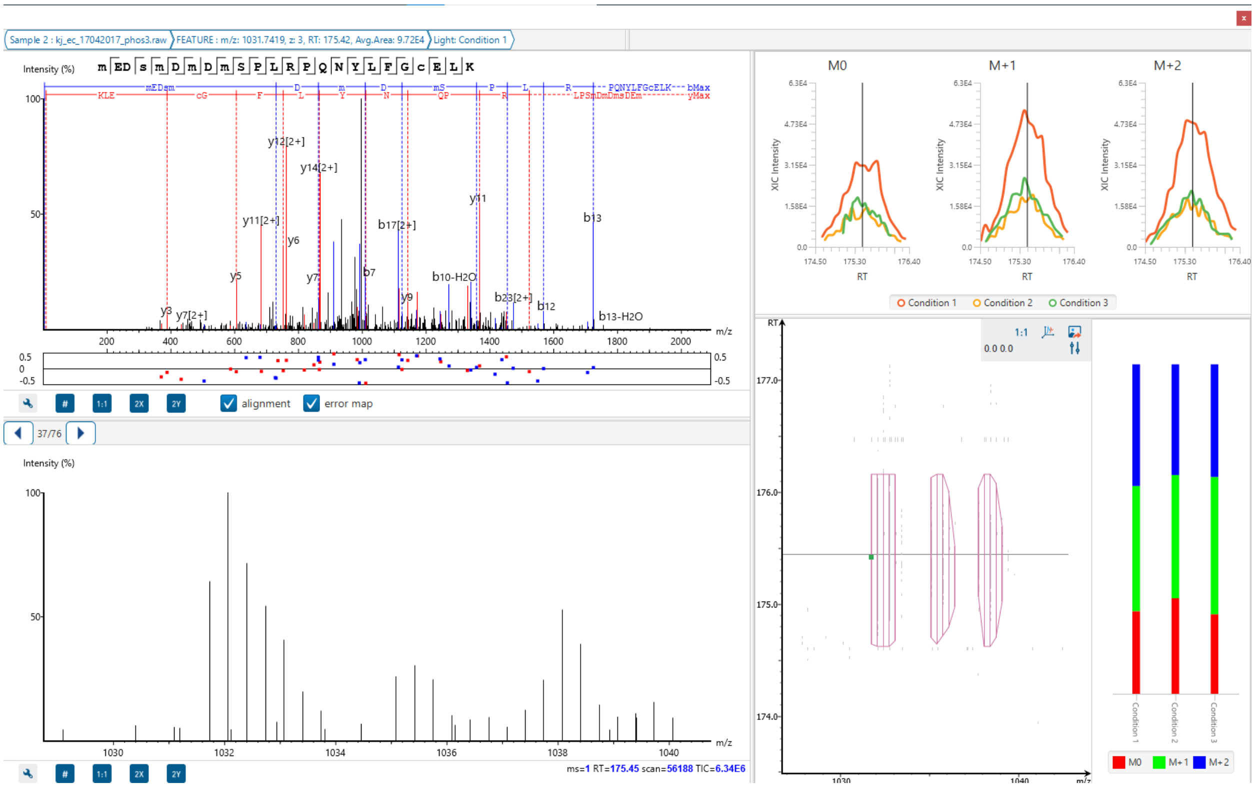

Right clicking on a peptide bar shows additional options. “Show in spectrum” has the same behaviour as left clicking on the peptide bar which is to open the annotated spectrum chart. “Show in table” has the function to jump to the supporting peptides table.

10.4.3.2 Coverage Control Panel

This panel controls what to display in the protein sequence coverage view.

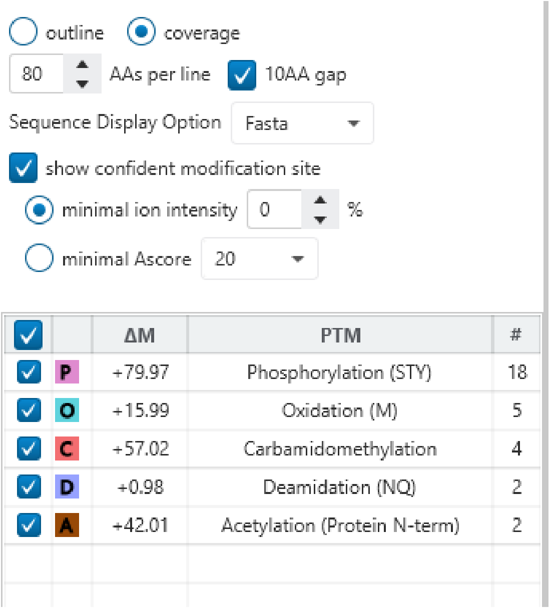

Sequence Display Mode: The protein sequence can be displayed in either "outline" or "coverage" mode.

Outline mode: In outline mode, the protein sequence is displayed without supporting peptides and matched de novo tags. However, the sequence coverage at a specific position can be examined by left-clicking a residue.

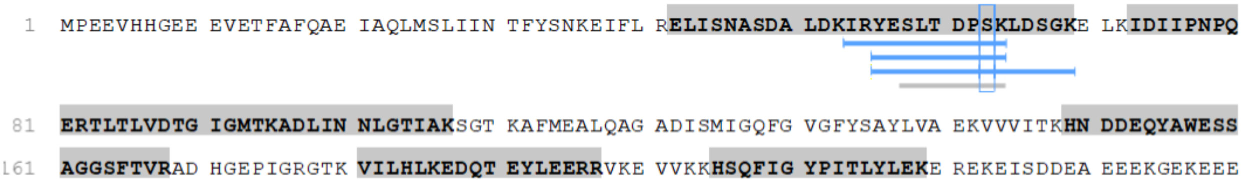

Coverage mode: In coverage mode, all the supporting peptides and matched de novo tags are shown.

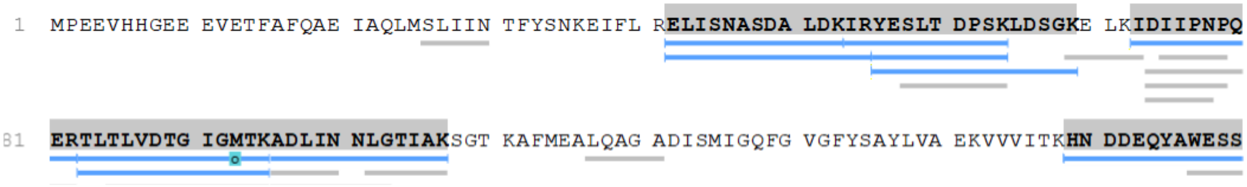

AAs per line and 10AA gap: Specify the number of amino acids per line and whether to show a gap for every 10 amino acids in the protein sequence.

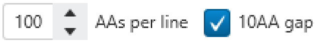

Sequence Display Option: Select whether to view the protein sequence as Fasta or a specific enzyme used in the project. When a particular enzyme is selected, the protein sequence will be highlighted to emphasize expected peptides generated by the selected enzyme's cleavage specificity.

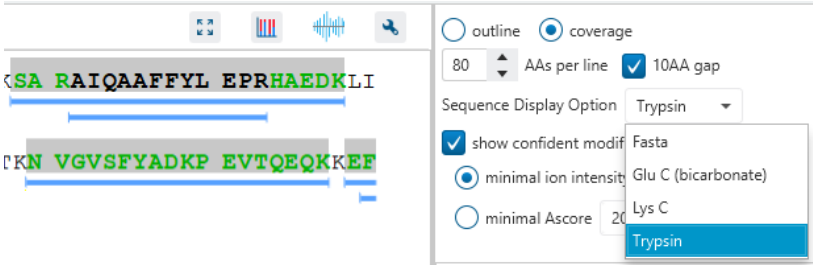

Modification minimal ion intensity / Ascore: A modification is considered confident if there are fragment ions supporting the modified residue with the minimal peak intensity above the specified threshold. Only confident modifications are displayed as icons above the protein sequence.

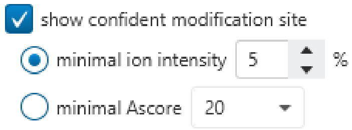

Modification table: The modification table shows the modifications identified in supporting peptides on the protein. For each modification, the number of supporting PSMs with this particular modification is shown. The checkbox on the left controls whether to indicate the modification in the protein coverage.

☑		ΔM	PTM	#
☑	T	+304.21	TMT16plex	27
☑	P	+79.97	Phosphorylation (STY)	27
☑	O	+15.99	Oxidation (M)	3

10.4.3.3 Proteins View - Protein Tools

The Proteins View toolbar is at the upper-right corner of the protein sequence coverage tab and contains helpful protein tools.

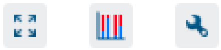

Full screen: When selected, the protein coverage window expands in full screen mode. Click to return from full screen mode.

Tool box: The tool box contains the following tools:

- Copy protein sequence. This copies the protein sequence of the current selected protein into the system clipboard.
- Save protein coverage as image. Different scale options are provided to increase the image size.
- Coverage statistics. This provides useful statistics based on the current selected protein.

PTM Profiling: Calculates the difference in abundance between peptides with confidently identified PTMs versus unmodified peptides. Peptide feature areas are used for this comparison.

The PTM Profiling tool provides quantitative information of modified peptides compared with unmodified peptides for the modification sites of the protein across all MS samples.

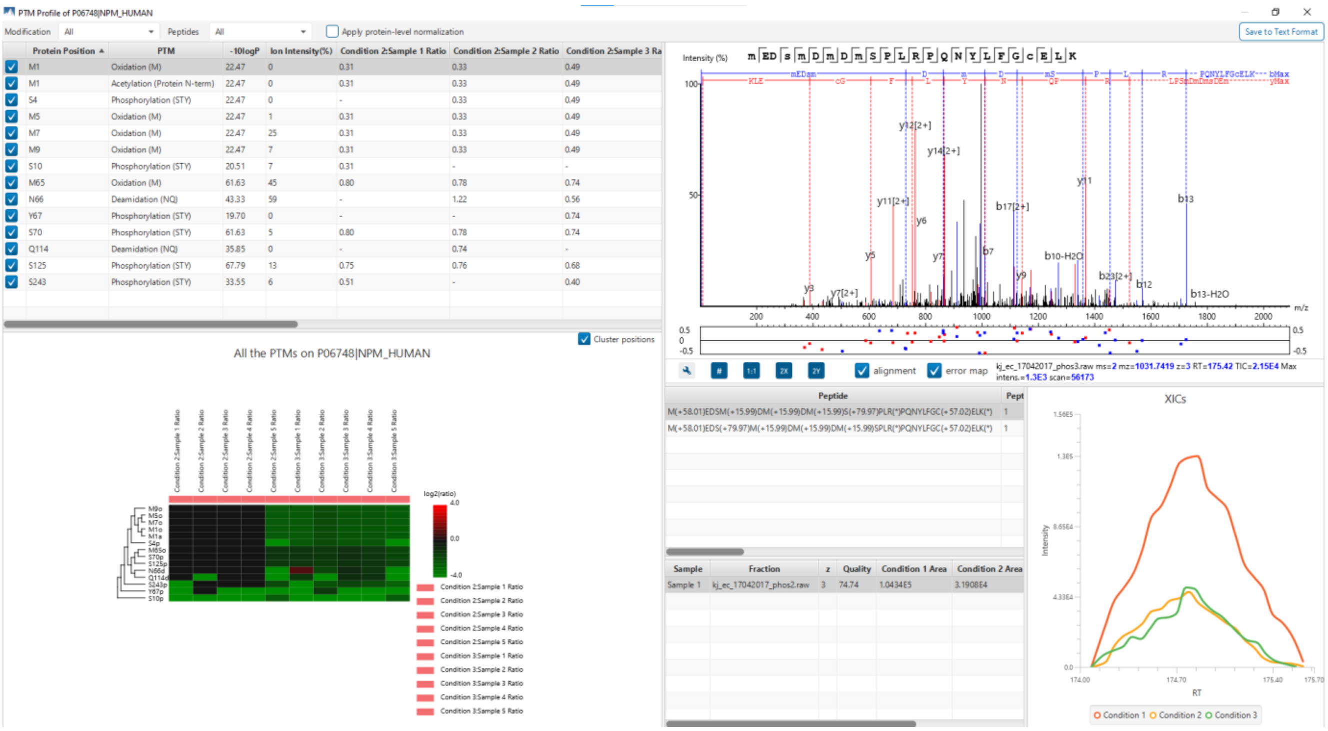

The inputs for the profiling can be set using the drop-down lists at the top-left corner of the dialog.

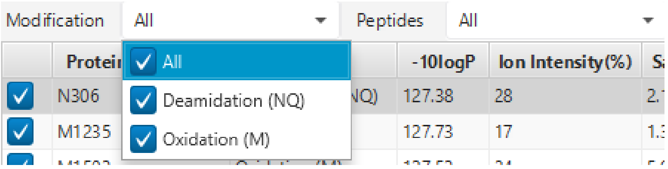

- **Proteins:** The accession ID of the selected protein to be analyzed for PTM profiling is shown in the title of the dialog. The selected protein can not be changed within the dialog. To run profiling on another protein, close the dialog, select the protein of interest from the protein table and run the profiling by clicking button.
- **Modification:** The drop-down list contains the detected and selected confident PTMs for the protein. Select the type of modification to study a specific PTM or choose "All the PTMs" to visualize the profiling information of all PTM sites at together.

  Note: The fixed PTMs are not used for profiling. In addition to this, the labeling modifications are not considered either.

- **Peptides:** Data source used for the analysis. "All" considers all the peptides for this protein.
- **Apply protein-level normalization:** By default, the protein-level normalization is not applied. If checked, the displayed modified/unmodified area is obtained by dividing the true modified/unmodified area by the corresponding ratio of the selected protein.

PTM Profile Table

The PTM Profile Table shows the detailed quantitative information for the modification sites. Each row in the table shows the modified and unmodified area for each sample of the selected PTM at each detected modification site. The table can be sorted by any column. The following list describes the contents in each column:

- **(Check box):** Allow the selection of each table row. Only the marked rows are used for the PTM Profile Chart.
- **Protein Position:** The detected modification sites in the protein. The modified amino acid symbol together with the position in the protein sequence is shown.
- **PTM:** The type of PTM being profiled.
- **-10logP:** The best -10logP identification score for the corresponding modified peptides.
- **AScore/ion intensity:** The best PTM AScore / ion intensity associated with the corresponding PTM at the specified site of the modified peptides.
- **Condition Sample Ratio:** Displays the ratio for each Condition versus the reference Condition for each sample.

	Protein Position ▲	PTM	-10logP	Ion Intensity(%)	Condition 2:Sample 1 Ratio	Condition 2:Sample 2 Ratio	Condition 2:Sample 3 Ra
✓	M1	Oxidation (M)	22.47	0	0.31	0.33	0.49
✓	M1	Acetylation (Protein N-term)	22.47	0	0.31	0.33	0.49
✓	S4	Phosphorylation (STY)	22.47	0	-	0.33	0.49
✓	M5	Oxidation (M)	22.47	1	0.31	0.33	0.49
✓	M7	Oxidation (M)	22.47	25	0.31	0.33	0.49
✓	M9	Oxidation (M)	22.47	7	0.31	0.33	0.49
✓	S10	Phosphorylation (STY)	20.51	7	0.31	-	-
✓	M65	Oxidation (M)	61.63	45	0.80	0.78	0.74
✓	N66	Deamidation (NQ)	43.33	59	-	1.22	0.56
✓	Y67	Phosphorylation (STY)	19.70	0	-	-	0.74
✓	S70	Phosphorylation (STY)	61.63	5	0.80	0.78	0.74
✓	Q114	Deamidation (NQ)	35.85	0	-	0.74	-
✓	S125	Phosphorylation (STY)	67.79	13	0.75	0.76	0.68
✓	S243	Phosphorylation (STY)	33.55	6	0.51	-	0.40

PTM Profile Heat Map

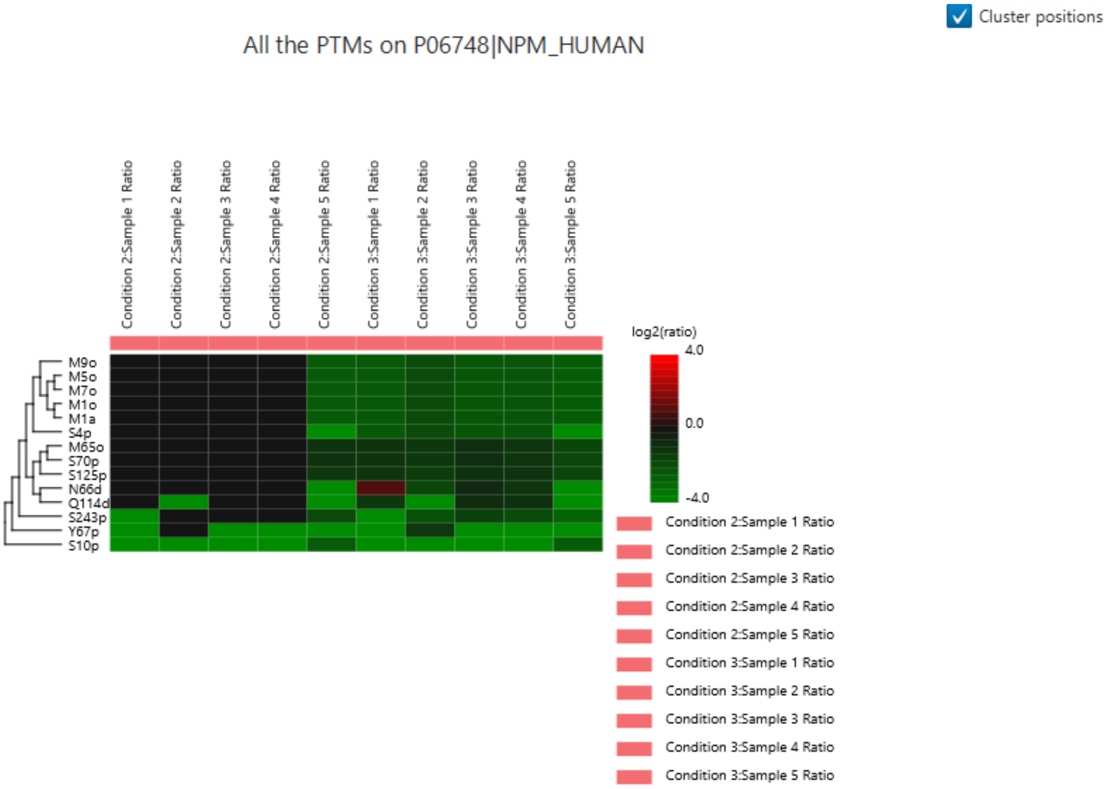

The PTM Profile Heat Map is located at the bottom left corner in the dialog. The relative abundance of modified peptides in all groups for the selected modification sites as a heat map. The color scheme is determined by the log2 ratios of the modified area of each group relative to the average abundance. For more details about the heat map, please refer to section 10.3.3 Summary View - Heat Map.

Spectrum Annotation View

The Spectrum Annotation View provides a graphical representation for the best modified peptide and the best unmodified peptide when “All” is selected from the “Peptides” drop-down list. The top spectrum corresponds to the best modified peptide, whereas the bottom spectrum shows the best unmodified peptide.

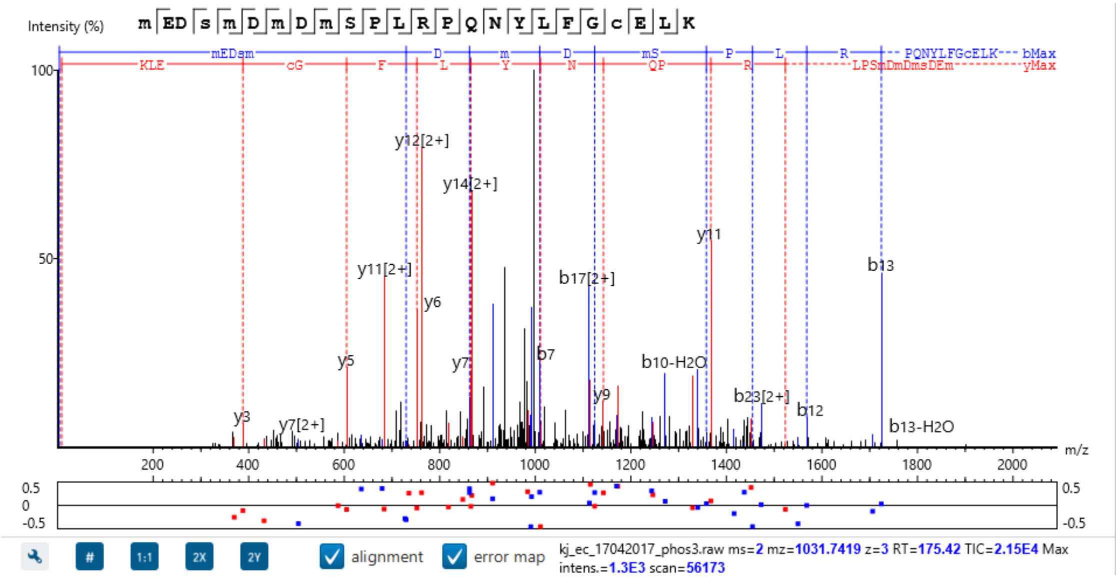

Peptides Table: Peptides table lists the modified peptides that contain the selected PTM at the specified modification site.

Peptide	Peptide Position	Ion Intensity(%)	Condition 2:Sample 1 Ratio	Condition 2:Sample 2 Ratio	Condition 2:
M(+58.01)EDSM(+15.99)DM(+15.99)...	1	0	0.31	-	-
M(+58.01)EDS(+79.97)M(+15.99)DM(...	1	0	-	0.33	0.49

All Matches Table: The supporting PSMs of the selected peptide in Peptides Table are listed in the All Matches Table. The headers are self-explanatory. The last column Area is the area of the feature that is associated with the corresponding PSM.

Sample	Fraction	z	Quality	Condition 1 Area	Condition 2 Area	Condition 3 Area	Ion Intensity(%)
Sample 1	kj_ec_17042017_phos2.raw	3	74.74	1.0434E5	3.1908E4	3.0446E4	0

XICs: It shows the XICs figure for the selected modified match in the All Matches Table. Each condition is plotted as a separate curve.

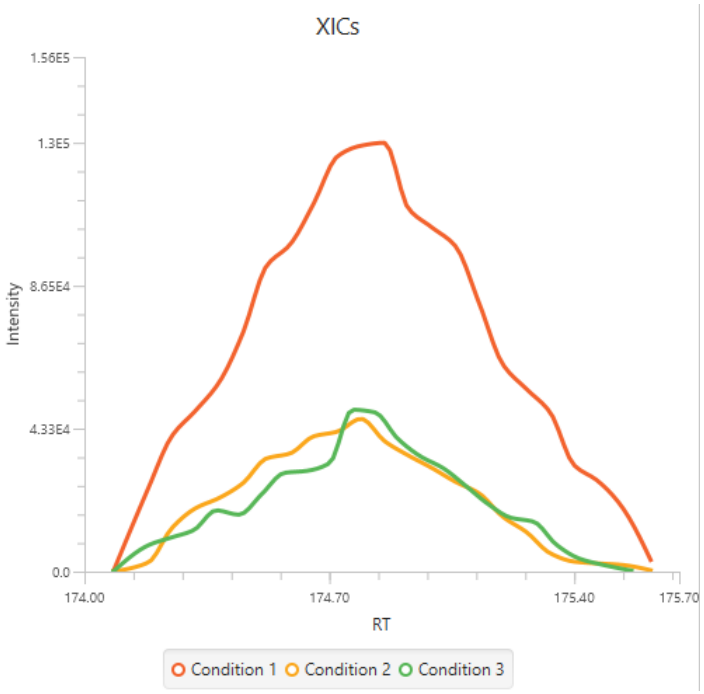

PTM Profile Export

The PTM profile can be exported as an image as well as in text format.

Image export: To save the profile chart, right click and choose Save to Image... menu item to save the displayed graph as a PNG file.

Text format export: Click [Save to Text Format] button at the top-right corner to export the calculated PTM Profile data to a text file in CSV format. The PTM Profile of the selected modification on the selected protein will be exported by default. In addition, profiles on all proteins for the selected modification may also be exported, if “Export ptm profile for all proteins” is checked. The exported file contains all necessary information to construct the PTM profile graph. The PTM profile data are grouped together by protein, then by modification type, and then by the modification site on the protein sequence. The headers of the CSV column are similar to the PTM Profile Table and also self-explanatory. The following items are explained for clarity:

- **Modified Peptide Sequence Window:** protein sequence containing 10 AA ahead of the modification site and 10 AA after the modification site with padding as necessary.
- **Sample Best AScore / Ion Intensity:** best AScore or Ion Intensity for each sample.

Note: When "Export PTM profile for all proteins" is not checked, only the profiling data that is marked in the PTM Profile Table will be exported.

10.4.3.4 Proteins View - Supporting Peptides

The supporting peptides assigned to the protein are shown in a separate tab beside the Protein Coverage view. The sequence can be clicked and a pop-up window will show up to display the feature details.

	Peptide	Used	-10lgP	Avg. Area	Condition 2: Ratio Profile	Condition 3: Ratio Profile	Group 1: Condition 2 Ratio	Group 1: Condition 3 Ratio	#Vector	Start	End	PTM
1	AES(+79.97)EDEEEEDVK(*)	√	27.77	1.24E4			0.59	0.32	2	123	134	P
2	C(+57.02)GS(+79.97)GPVHISGQHL...	√	17.82	3.55E5			0.85	0.70	1	104	134	
3	C(+57.02)GSGPVHISGQ(+0.98)HLV...	√	35.85	4.31E6			0.83	0.68	3	104	134	C D P
4	C(+57.02)GSGPVHISGQHLVAVEED...	√	29.23	1.96E6			0.86	0.65	3	104	134	C P
5	DELHIVEAEAM(+15.99)N(+0.98)YE...	√	43.33	4.09E4			0.83	0.54	6	55	73	O D P
6	DELHIVEAEAM(+15.99)NY(+79.97)...	√	19.69	6.29E5			0.74	0.59	1	55	73	O P
7	DELHIVEAEAM(+15.99)NYEGS(+79....	√	61.63	1.42E6			0.78	0.60	10	55	73	O P
8	GPSS(+79.97)VEDIK(*)	√	33.54	1.34E4			0.49	0.34	4	240	248	P
9	GQHLVAVEEDAES(+79.97)EDEEEED...	√	28.03	2.76E4			0.74	0.49	2	113	134	P
10	GSGPVHISGQHLVAVEEDAES(+79.9...	√	35.70	3.6E5			0.78	0.54	2	105	134	P
11	HLVAVEEDAES(+79.97)EDEEEEDVK(*)	√	58.11	2.5E5			0.72	0.56	10	115	134	P
12	ISGQHLVAVEEDAES(+79.97)EDEEE...	√	56.01	4.55E5			0.69	0.57	5	111	134	P
13	K(*)LAADEDDDDDDEEDDDEDDDD...	√	21.10	2.23E3			0.38	0.86	1	157	189	
14	LAADEDDDDDDEEDDDEDDDDDDF...	√	31.94	4.42E6			0.86	0.63	5	158	189	
15	LAADEDDDDDDEEDDDEDDDDDDF...	√	40.55	4.82E6			0.70	0.57	9	158	193	
16	LAADEDDDDDDEEDDDEDDDDDDF...	√	32.53	1.21E7			0.78	0.60	9	158	194	
17	LVAVEEDAES(+79.97)EDEEEEDVK(*)	√	56.57	1.21E5			0.75	0.41	1	116	134	P
18	M(+58.01)EDS(+79.97)M(+15.99)D...	√	22.47	1.24E5			0.36	0.41	5	1	24	[illegible] O P O O O [illegible]

This table contains the following information of the quantifiable support peptides for the selected protein:

- **Peptide:** The amino acid sequence of the peptide, as determined in PEAKS Database or Spectral Library search. A modified residue is followed by a pair of parentheses enclosing the modification mass. All non-unique peptides and those not passing the filter will be excluded automatically.
- **Used:** Shows whether this peptide is used to calculate the protein abundance. The top three peptides with highest abundance are used to calculate the protein abundance.
- **-10lgP:** The -10lg of a p-value represents the likelihood that the observed change between conditions is caused by random chance. The peptides are first separated into groups based on similar quality score. Significance is then calculated for each quality score group, or bin, using a two-tail T-test that assumes log normal distribution but does not assume equal variance. The -10log p-value is then calculated from this result and then displayed in the significance column.
- **Average Area:** Each peptide feature associated with the peptide has its own area under the XIC curve. The average area of is the average area of all the features associated with the peptide.
- **Condition Profile:** The relative peptide abundance for each condition compared to the reference condition among the samples is depicted as a heat map. Hold the cursor on a profile to view the samples, the areas, and the ratios with respect to the reference condition.
- **Condition Ratio:** The ratio columns show the relative peptide abundance for each condition with respect to the reference condition for all groups. A peptide's abundance in each condition is the sum of its supporting peptide feature abundances within the peptide filters. Then the peptide abundance in each condition is compared to the reference condition to obtain the peptide condition ratio in each sample separately. The peptide condition ratio of a group is computed by calculating the median from the peptide condition ratios of the samples in that group.
- **#Vector:** This shows the number of quantifiable feature vectors of a peptide. A feature vector is a full set of peptide features used to calculate a ratio.
- **Start:** This shows the protein position of the first residue of the peptide.
- **End:** This shows the protein position of the last residue (inclusive) of the peptide.
- **PTM:** The types and the numbers of modifications present in the peptide shown using color-coded icons. Scroll over each icon to see the modification name and mass.

10.5 SILAC result - Peptides View

Quantified proteins are displayed in the Peptide View along with their detailed information.

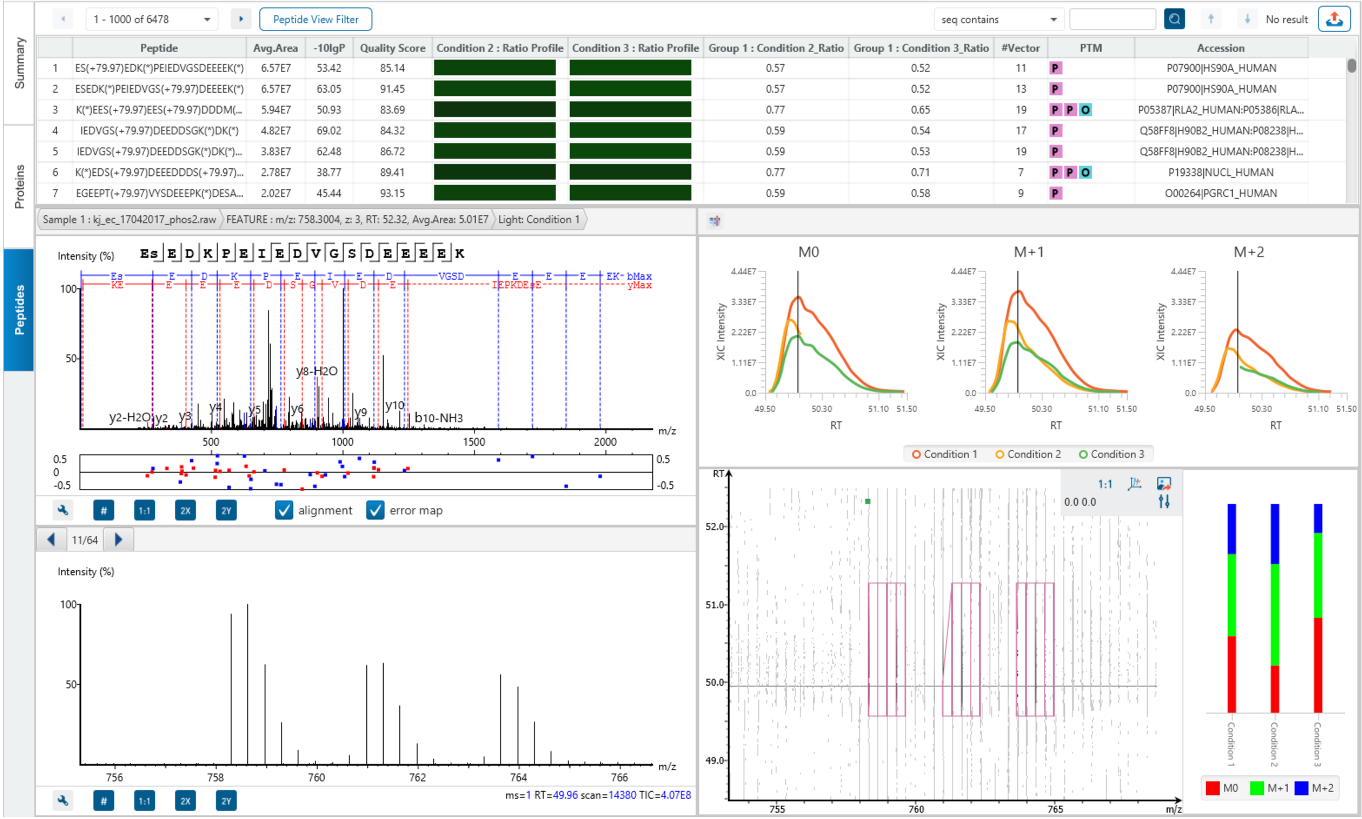

10.5.1 Peptides View - Peptide Table

The peptides are grouped together based on quantifiable feature vectors with the same sequence. When there are more than 1000 peptides, the table is split into multiple pages.

	Peptide	Avg.Area	-10lgP	Quality Score	Condition 2 : Ratio Profile	Condition 3 : Ratio Profile	Group 1 : Condition 2_Ratio	Group 1 : Condition 3_Ratio	#Vector	PTM	Accession
1	ES(+79.97)EDK(*)PEIEDVGSDEEEEK(*)	6.57E7	53.42	85.14			0.57	0.52	11	P	P07900|HS90A_HUMAN
2	ESEDK(*)PEIEDVGS(+79.97)DEEEEK(*)	6.57E7	63.05	91.45			0.57	0.52	13	P	P07900|HS90A_HUMAN
3	K(*)EES(+79.97)EES(+79.97)DDDM(...	5.94E7	50.93	83.69			0.77	0.65	19	P P O	P05387|RLA2_HUMAN:P05386|RLA...
4	IEDVGS(+79.97)DEEDDSGK(*)DK(*)	4.82E7	69.02	84.32			0.59	0.54	17	P	Q58FF8|H90B2_HUMAN:P08238|H...
5	IEDVGS(+79.97)DEEDDSGK(*)DK(*)...	3.83E7	62.48	86.72			0.59	0.53	19	P	Q58FF8|H90B2_HUMAN:P08238|H...
6	K(*)EDS(+79.97)DEEEDDDS(+79.97)...	2.78E7	38.77	89.41			0.77	0.71	7	P P O	P19338|NUCL_HUMAN
7	EGEEPT(+79.97)VYSDEEEPK(*)DESA...	2.02E7	45.44	93.15			0.59	0.58	9	P	O00264|PGRC1_HUMAN

The view is similar to the support peptide table described in the previous section Section 10.4.3.4 Proteins View - Supporting Peptides. The Peptide table entries may be filtered by using the Peptide View Filters on top of the peptide table. To reiterate, the columns are (with two extra columns Quality score and Accession):

- **Peptide:** The amino acid sequence of the peptide, as determined in PEAKS Database or Spectral Library search. A modified residue is followed by a pair of parentheses enclosing the modification mass. All non-unique peptides and those not passing the filter will be excluded automatically.
- **-10lgP:** The -10lg of a p-value represents the likelihood that the observed change between conditions is caused by random chance. The peptides are first separated into groups based on similar quality score. Significance is then calculated for each quality score group, or bin, using a two-tail T-test that assumes log normal distribution but does not assume equal variance. The -10log p-value is then calculated from this result and then displayed in the significance column.
- **Average Area:** Each peptide feature associated with the peptide has its own area under the XIC curve. The average area of is the average area of all the features associated with the peptide.

- **Quality Score:** The quality score represents the predicted reproducibility of the quantification result. Factors that affect the quality score include m/z difference, RT difference, XIC shape similarity, and the feature intensities.
- **Condition Profile:** The relative peptide abundance for each condition compared to the reference condition among the samples is depicted as a heat map. Hold the cursor on a profile to view the samples, the areas, and the ratios with respect to the reference condition.
- **Condition Ratio:** The ratio columns show the relative peptide abundance for each condition with respect to the reference condition for all groups. A peptide's abundance in each condition is the sum of its supporting peptide feature abundances within the peptide filters. Then the peptide abundance in each condition is compared to the reference condition to obtain the peptide condition ratio in each sample separately. The peptide condition ratio of a group is computed by calculating the median from the peptide condition ratios of the samples in that group.
- **#Vector:** This shows the number of quantifiable feature vectors of a peptide. A feature vector is a full set of peptide features used to calculate a ratio.
- **Start:** This shows the protein position of the first residue of the peptide.
- **End:** This shows the protein position of the last residue (inclusive) of the peptide.
- **PTM:** The types and the numbers of modifications present in the peptide shown using color-coded icons. Scroll over each icon to see the modification name and mass.
- **Accession:** The proteins supported by the peptide, separated by ',' if the peptide supports multiple proteins. If none of the proteins that the peptide supports pass the protein filters, the accession field will be empty.

To copy the sequence, right click on a row (or multiple rows) in the table and select “Copy selected peptide sequence(s)”. Another option is to mouse over any cell in the table and hitting Ctrl + C on your keyboard to copy the contents of those selected cells.

10.5.2 Peptide Table controls

The menu above the peptide table provides options to scroll through the table of peptides, filter the table, and search for specific entries.

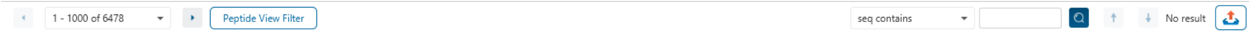

10.5.2.1 Peptide Table controls - Pagination

For easy viewing and to improve performance, the peptide table is limited to showing 1000 entries at once. Pagination is used to manage which set of 1000 entries will be viewed.

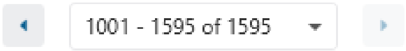

In the top left, there is a dropdown to select the rows for viewing. Click the left arrow or right arrow to select the previous/next 1000 rows. If the table is sorted or filtered, it may update the pagination set of rows that is currently in view.

10.5.2.2 Peptide Table controls - View Filter

Click on the 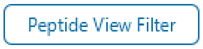

button to open the Peptide View Filter which is used to filter the Peptide table. The following are the filter options provided:

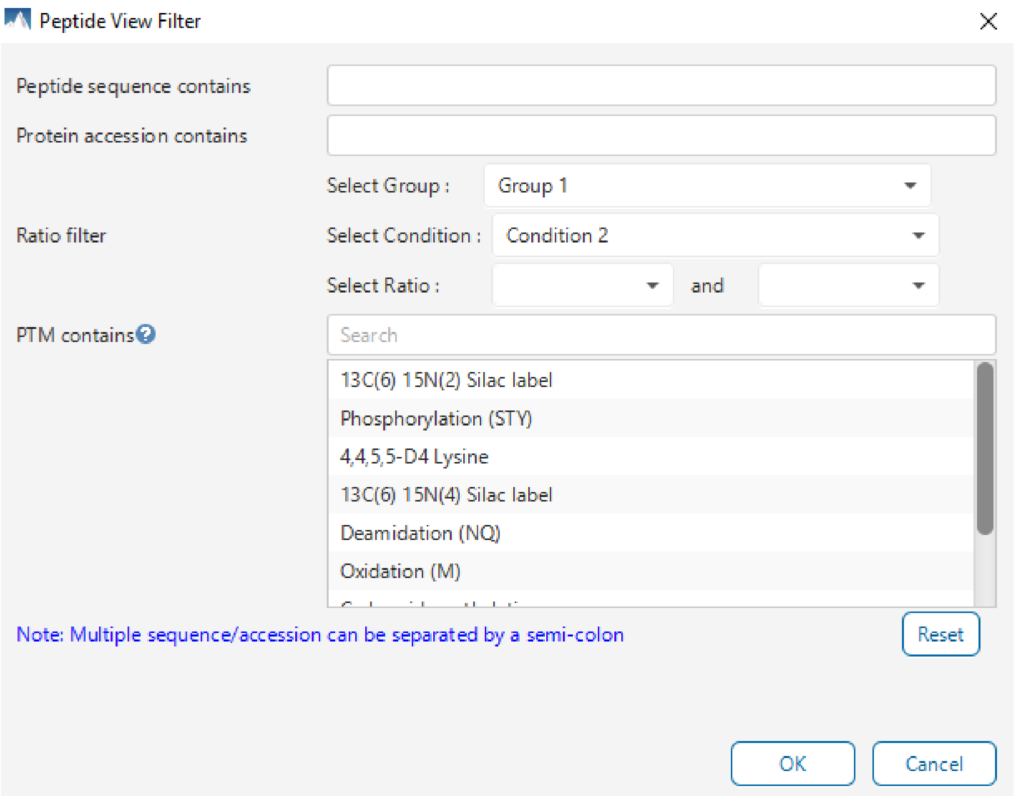

- **Peptide sequence contains:** Enter an amino acid sequence, only peptides that contain that sequence will be displayed in the peptide table. Adding PTMs in brackets in the sequence search is not handled, meaning only the sequence backbone is considered in the filter. Use the PTM contains for further filtering if necessary.

- **Protein Accession Contains:** Enter a peptide accession, only peptides that contain that accession will be displayed in the peptide table.

- **Show Transferred IDs Only:** Only display peptides where match between runs was used to include at least one peptide feature in the result.

- **Ratio Filter:** Only peptides where the ratio for the selected group and condition fall within the specified range will be displayed in the peptide table.

- **PTM contains:** Type the names of PTMs you would like to include. Peptides containing PTMs that match the text will be included. If multiple PTMs match the text they will all be included in the table.

Note: In all filters, multiple entries can be separated by a semi-colon.

Click on the Reset button to reset all filters to their default setting.

Click on OK to apply the filters to the Peptides table.

Click on Cancel to discard the changes in the View Filter.

10.5.2.3 Peptide Table controls - Search function

On the top right of menu, there is a search function with 1 option. Search results can be navigated between using the Up or Down arrows.

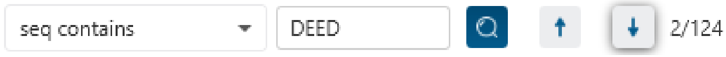

Options for searching include:

- **Seq contains:** Search for the sequence in the table. If specifying a sequence without PTMs, the results will include that sequence and any sequences with PTMs with the same backbone. If specifying a sequence with bracket and some mass value (like in the above image), then only sequence that have a substring with an exact match will be considered.
  Additionally, since the peptide table shows both I/L and they can not be distinguished, then searching for I in the sequence is equivalent to searching for L in the sequence. i.e. Searching for "LVLK" is the same as searching for "LVIK", "IVLK", and "IVIK"

10.5.3 Feature vector selection breadcrumb

There is a sample and feature selection breadcrumb which allows you to switch between features associated with the peptide. Click on the Sample in the breadcrumb to reveal other samples and the features in that sample related to the current peptide selection. From here you can select other samples, fractions, or features.

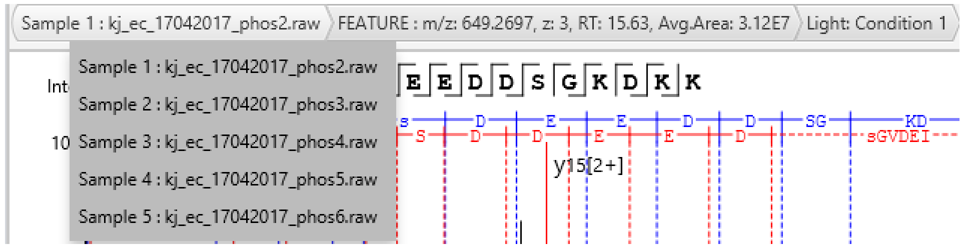

By clicking on the fraction level, all fractions in this sample are revealed as well as the features in each fraction associated with this peptide. From here you can select other fractions and features to view.

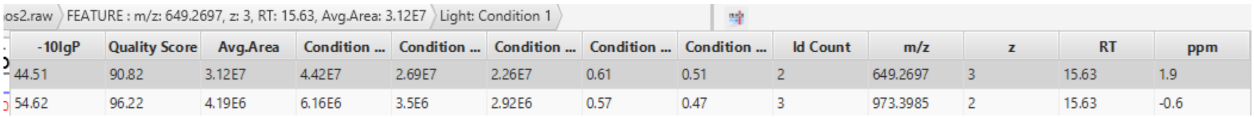

-10lgP	Quality Score	Avg.Area	Condition ...	Condition ...	Condition ...	Condition ...	Condition ...	Id Count	m/z	z	RT	ppm
44.51	90.82	3.12E7	4.42E7	2.69E7	2.26E7	0.61	0.51	2	649.2697	3	15.63	1.9
54.62	96.22	4.19E6	6.16E6	3.5E6	2.92E6	0.57	0.47	3	973.3985	2	15.63	-0.6

Clicking on the feature vector level will show all features vectors in the currently selected fraction.

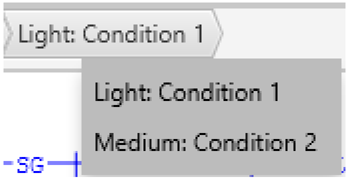

Switch between Conditions with the last portion of the breadcrumb.

All other components in the Peptide tab interface will update according to the currently selected Condition.

10.5.4 Protein jump button

Click on 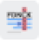 to jump to a protein that contains the currently selected PSM. If there are more than 1 protein, a dropdown is revealed which allows the user to select which protein to jump to. If a protein is filtered out due to a protein summary filter or summary page protein filter, then the option to jump will be grayed out.

10.5.5 Annotated Spectrum Chart

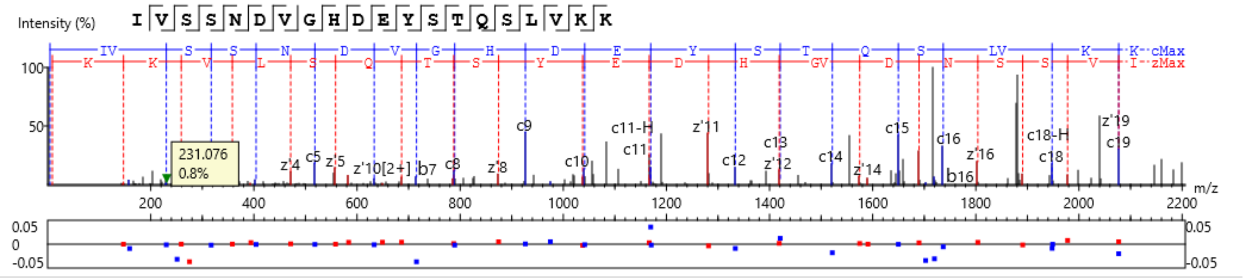

For the currently selected PSM, a graphical representation is shown in the form of an annotated spectrum chart. There are several controls provided for this view which will be explored in the following sections.

10.5.5.1 Annotated Spectrum Chart - Chart Navigation

Moving the cursor over the peptide sequence in the spectrum will show the mass transitions for a particular amino acid residue.

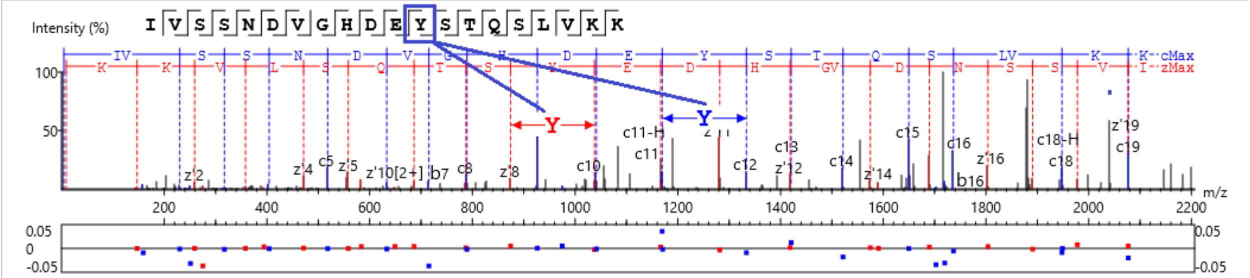

Zoom to a m/z region: Drag horizontally from the start m/z and to the end m/z with left mouse button.

Zoom in/out smoothly: Place the cursor at a particular m/z value (right below the x-axis), zoom in/out by scrolling the mouse wheel.

Increase/Decrease peak intensity: Place the cursor in the spectrum and scroll the mouse wheel.

See the whole spectrum: Double click in the spectrum or click the 1:1 button.

10.5.5.2 Annotated Spectrum Chart - Spectrum Annotation Settings

Click on the 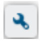 button to show the spectrum annotation settings.

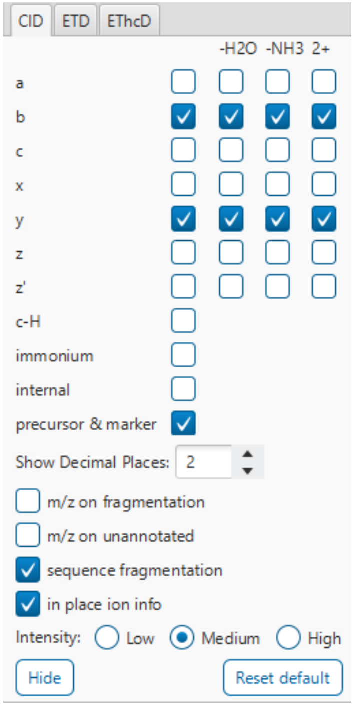

The spectra in the PEAKS results can be annotated by selecting ion types from the thorough collection of ions that PEAKS offers. The selected ion types will be displayed in the "Ion Match" table, as well. It is possible to annotate the spectrum with various ions for CID, EThcD and ETD. Each of these fragmentation modes can be set with different settings which will be shared with other results with the same fragmentation type.

To reset the defaults, click on the "Reset default" button.

Show Decimal Places: Select the number of decimal places that will appear in the ion table and the spectrum view. The default is set to two decimal places.

m/z on Fragmentation: Select this to display the m/z value on top of the annotated ions.

m/z on Unannotated: Select this to display the m/z value on top of the peaks without ion matches.

Sequence Fragmentation: Select this to display the sequence fragmentation on the top-left corner of the "Spectrum Annotation" view.

In Place Ion Info: Ion information, m/z value,and relative intensity are all displayed in a pop-up within the "Spectrum Annotation" view when this option is checked and the cursor is placed on a peak.

Intensity: Set the intensity threshold for spectrum annotation to low (2%), medium (5%), or high (10%).

10.5.5.3 Annotated Spectrum Chart - Additional Chart Controls

Click on 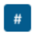 or 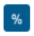 to toggle between absolute and percentage intensity values in the Y axis.

Click on 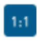 to reset the zoom level of the annotated spectrum chart to the default.

Click on 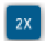 to zoom into the X axis by a factor of 2.

Click on 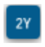 to zoom into the Y axis by a factor of 2.

The "alignment" check box ☑ alignment allows user to display/hide how the fragment ions generated from the peptide align with the spectrum. N-terminal ions are shown in blue and C-terminal ions are shown in red.

The “error map” check box ☑ error map is used to show/hide the error map underneath the chart.

10.5.6 MS1 Spectrum

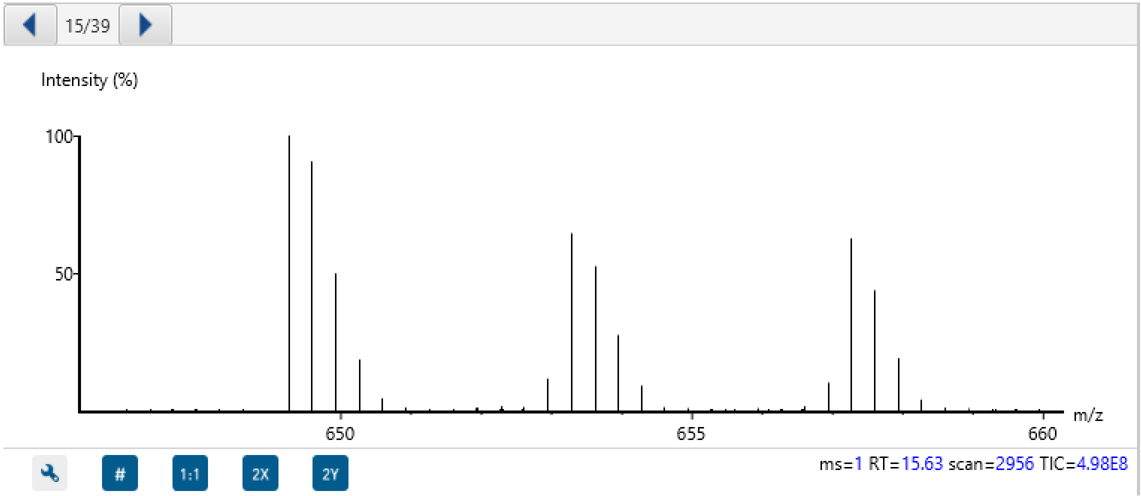

This view provides an MS1 spectrum zoomed in on the reporter ions in the experiment. This is an interactive spectrum, enabling zooming in and out using the scroll wheel of the mouse. Double-clicking will zoom in on an area of interest. Clicking and dragging will slide the spectrum to the left or right.

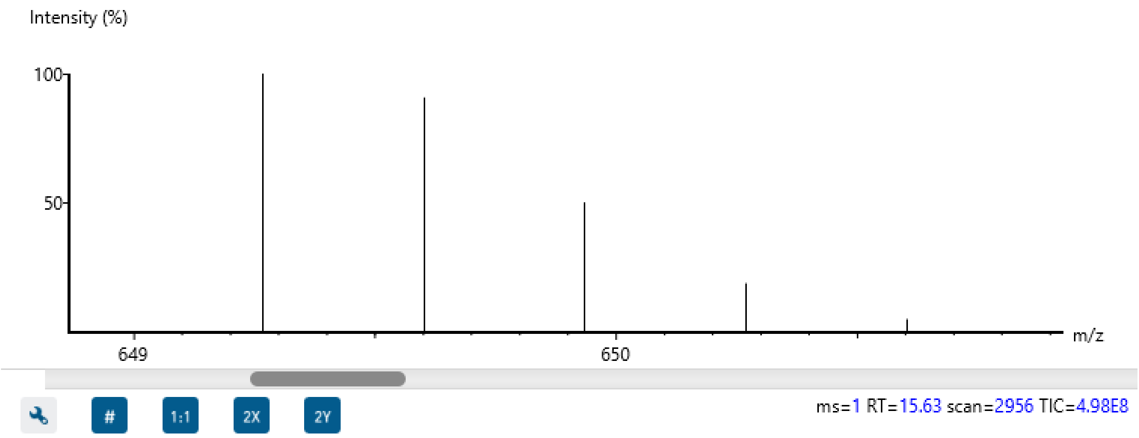

10.5.7 eXtracted Ion Chromatogram (XIC) chart

This map presents the changes in feature intensity over the retention time range of the peaks for all labels of the selected peptide. Additionally, the coloured isotopic distribution plot - M0, M+1, M+2, etc. - of each label is included, which shows the relative composition of each label.

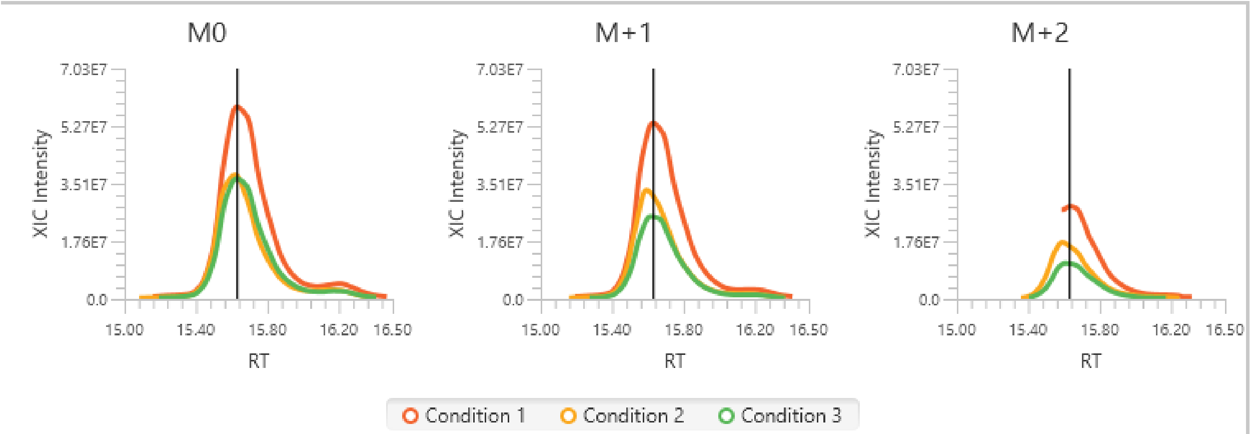

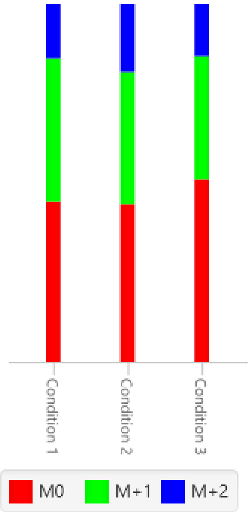

10.5.8 LC/MS Snapshot

This LC/MS screen is zoomed into the features that correspond to the isotopic labels, e.g. “Light” or “Heavy”. The m/z between the markers for these features should equate to the mass difference of the two labels in this sample.

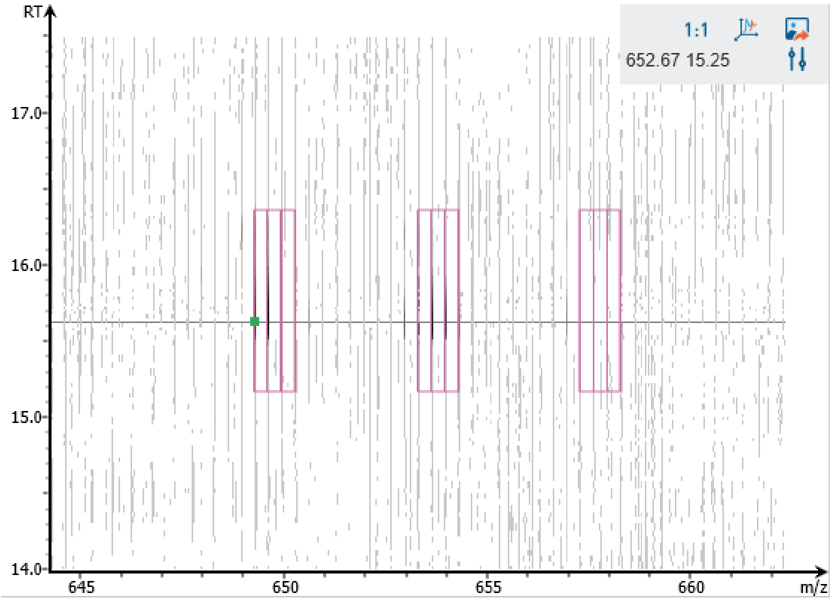

Users can change the view from the 2-D heat map to a 3-D intensity view by clicking the intensity view button in the upper right-hand corner of the LC-MS Snapshot. In addition, users can export the LC-MS heat map by clicking the export image button in the upper right-hand corner of the LC-MS Snapshot.

Expand the options dropdown by clicking to reveal a search option to specify a *m/z RT* (ensure there is a space between these two numbers) location to zoom into. There is also a Peak slider filter to filter out low intensity peaks and a feature area filter to filter out low area features.

10.6 SILAC Exporting

In Project View, double-click on Export to open the Export node.

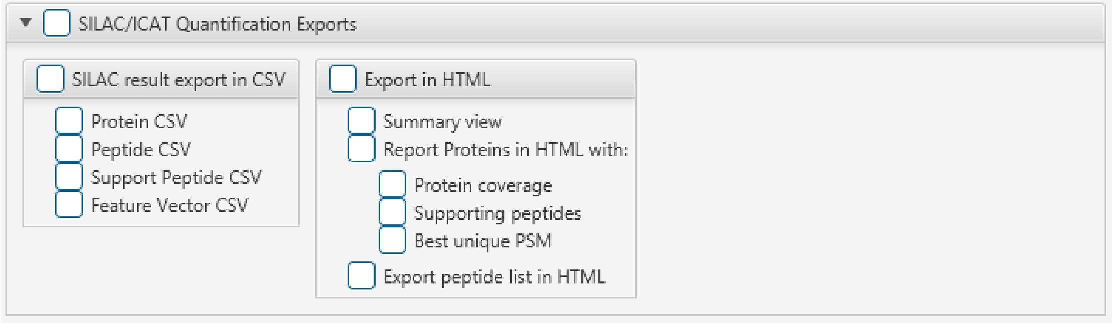

10.6.1 SILAC - Exporting - comma separated values (CSV) format

There are several options for exporting the PEAKS Precursor Ion Quantification results in CSV format:

- **Proteins CSV:** The list of protein quantifications, filtered by the protein filters in the Summary view and will be saved to proteins.csv.
- **Peptide CSV:** A complete list of all quantified peptides that pass the peptide feature filters set on the "Summary" page and their associated details.
- **Support peptides CSV:** These are the supporting peptides of the quantified proteins and their associated details.

- **Feature Vector CSV:** All predicted peptide features and their associated quantification information. Both identified and unidentified peptide features are included. To include unidentified peptide features, set the Peptide Id Count equal to 0 in the Peptide feature filter.

10.6.2 SILAC - Exporting - HTML format

Export in HTML: This will generate single or multiple HTML reports in the specified location.

The following exporting options are available:

- **Summary view:** The “Summary” view page will be saved as a summary.html file in HTML format in the specified location.
- **Protein coverage:** The coverage pane will be saved for each protein.
- **Supporting peptides:** A list of supporting peptides will be saved for each protein.
- **Best unique PSM:** The best unique PSM will be saved for each protein.
- **Export peptide list in html:** creates a separate HTML-containing peptide identifications.

An individual protein will have its own HTML output file, where the corresponding protein coverage, the supporting peptides, and the best unique PSM are gathered.

11. Glycan Search

PEAKS Glycan is an add-on module for PEAKS Studio that provides a unique solution for in-depth Glycoproteomic analysis. PEAKS Glycan module utilises a glycopeptide-based approach to profile the glycoproteins in your sample using LC-MS/MS data. As part of the PEAKS Studio platform, PEAKS Glycan module presents a refined database search and newly developed algorithms to facilitate the identification and characterisation of both N- and O-linked glycans. Glycan profiling is done at specific sites within a protein (positional profiling) and compares glycopeptide abundances across samples by label-free quantification. Currently, it supports ThermoFisher and Bruker instruments and includes an easy-to-use project wizard for workflow and analysis parameter selection that will be familiar to all PEAKS users.

Glycosylation, the attachment of sugar moieties or glycans to organic molecules, is one of the most common post-translation modifications (PTM) and plays a critical function in a wide range of biological processes. Glycans are the most complex and heterogeneous class of carbohydrate-based structures due to their non-template-driven biosynthetic process. They can be linear or branched, and they are attached to the side chain of amino acids within glycoproteins. The glycans in glycoproteins can be categorised as being N-linked, glycans bound to nitrogen atom of asparagine (N), or O-linked, glycans bound to the oxygen of serine (S) or threonine (T) side chains. Importantly, glycosylation can affect a proteins structure, stability, and function, and is important for physiological and pathological functions.

Glycoproteomics involves identifying glycans and the associated glycosylation sites within proteins across the proteome of a given cell or tissue. At the protein level, amino acid sequences, or individual amino acids, are used to predict glycosylation sites. Fragmentation data from MS/MS spectra are used to identify these sites and the attached N- or O-linked glycan structures. In the past, LC-MS/MS-based glycoproteomics was challenged by complexity of structures present in a sample, resulting in an extensive search space consisting of all potential peptide sequences, glycans' compositions and structures, in addition to other PTMs. PEAKS Glycan module was designed to handle these challenges of mass spectrometric glycoproteome analysis, and as a comprehensive data analysis tool it provides a highly sensitive and accurate glycoproteomics software solution.

11.1 Glycan Search Analysis Workflow and Setting

Select the PEAKS Search workflow in the Workflow Selection step

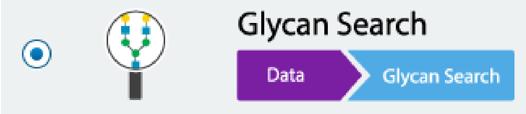

The following steps are similar to the PEAKS DB workflow (as in section 6.1 Database Search Workflow and Parameter Settings) and is reiterated below. There are additional Glycan Option parameters for Glycan Search.

Click on Data Refine to proceed through the workflow.

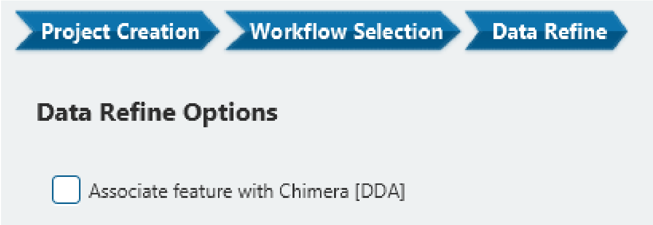

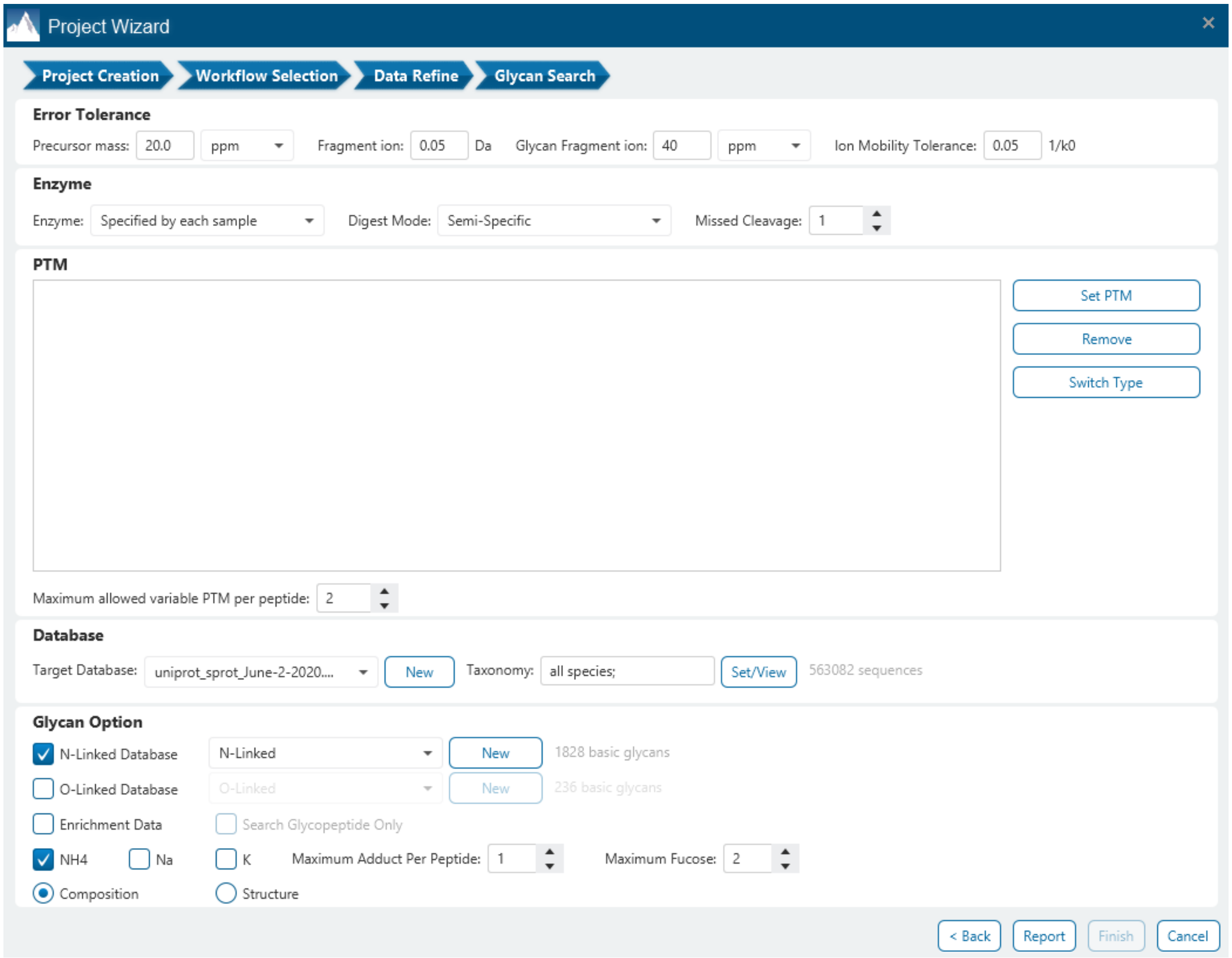

The following parameter settings are provided:

Precursor Mass Error Tolerance: The maximum mass difference between the peptide and the monoisotopic mass of the precursor ion.

Precursor Mass Tolerance unit: PEAKS generates peptides within the precursor mass error tolerance. Precursor mass error tolerance can be specified in either Daltons or ppm.

Fragment Mass Error Tolerance: PEAKS uses this value when scoring peptide sequences. PEAKS Studio considers a fragment ion to be matched if the calculated m/z is within the specified error tolerance. Units are Daltons.

Glycan Fragment ion: This enables the selection of a fragment ion m/z tolerance for Glycans. Glycan Search uses this value when scoring glycan peptides. PEAKS considers that a fragment ion is matched if the calculated m/z is within the tolerance. Glycan Fragment ion mass tolerance can be specified in either Da or ppm.

Ion Mobility Tolerance (1/k0): This tolerance can only be set for timsTOF instruments. This setting is the allowable maximum shift in mean 1/k0 of an identified peptide between different runs,

Both precursor and fragment error tolerance parameters need to be set consistently with the mass accuracy of the instrument.

Enzyme Settings: PEAKS Studio digests the protein database *in silico* to generate peptide candidates. It is necessary to specify the enzyme for protein digestion from the Enzyme drop-down menu.

Note: When the selected dataset is digested with different enzymes, "Specified by each sample" allows samples to be analyzed separately using their respective enzymes specified during project creation.

Note: "None" is a special enzyme allowing non-specific cleavage at both ends of the peptide. It is the recommended option when no digestion enzyme was used or when the digestion enzyme exhibits a high degree of non-specificity.

Missed Cleavages: This specifies the number of missed cleavage sites allowed in a peptide.

Digest Mode: This specifies the type of in silico cleavages allowed at peptide termini based on the method used for protein digestion. If "Specific" is selected, the specificity of the selected enzyme is strictly enforced at both termini of a peptide. "Semispecific" allows one terminus to disobey the enzyme specificity rules.

Note: If the enzyme is specified as "None", then no matter which mode is selected for "Digest Mode", the "None" enzyme digest rule will be applied.

PTM Settings: Click “Set PTM” to open the **PTM Options** dialog and specify the fixed PTMs and a few common variable PTMs expected in the experiment. Highlight PTMs and click on the ➡ button to place them as either Fixed or Variable PTM. From there, highlighted PTMs can be removed by clicking the “Remove” button. Clicking “Remove All” will reset the PTM options. Highlighting and clicking “Switch Type” will move the Fixed PTM in into the Variable PTM list and vice-verse. Click on “New” to create a new custom PTM. While a PTM is selected, click on “View” to see more details.

Note: Multiple PTMs can be highlighted by holding down the CTRL key or SHIFT key on the keyboard.

Note: For a specific residue, only one fixed modification is allowed.

Once the PTMs are selected, the maximum number of variable PTMs per peptide can be specified and a value of less than 4 is recommended.

Note: The use of variable modifications increases the size of the computational search space for the de novo sequencing algorithm. It is recommended not to select too many variable modifications in PEAKS de novo. See PEAKS PTM for alternate search options.

Target Database: Select a protein sequence database for the search. Select one from the list of databases that have been configured in PEAKS and set the taxonomy by clicking on [Set/View], if applicable. To configure a new database, click on [New] next to the Target Database dropdown to open the Database Configuration dialog.

Glycan Option:

Glycan Search can be selected based on the glycan linkage nature: N-Link, O-Link, or N-Link + O-Link Glycan Search. N-glycans are bound to Asparagine (N), while O-glycans are bound to Serine (S) / Threonine (T). To run N-link followed by O-link, check both checkboxes next to the Glycan Database selection.

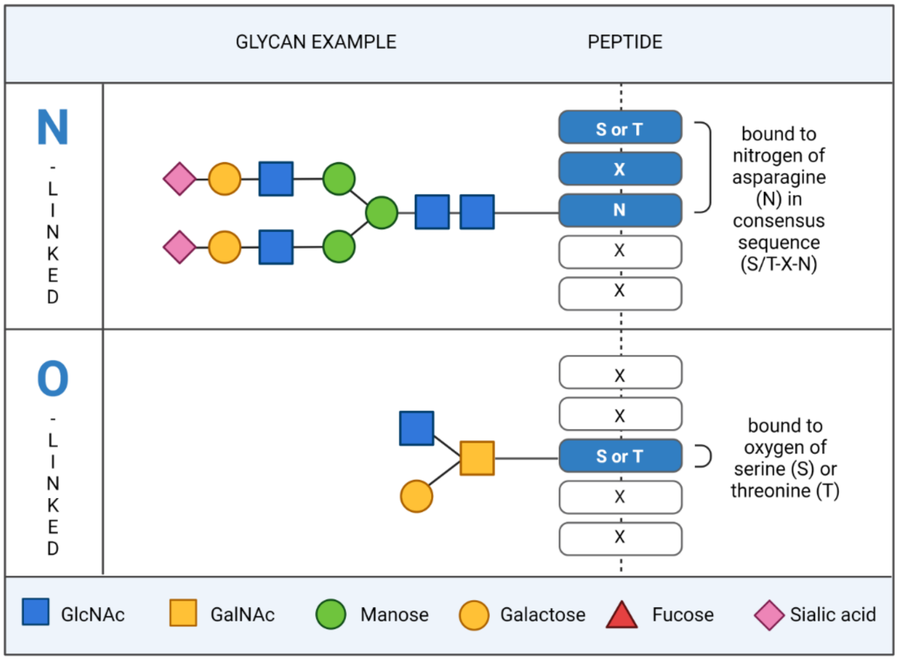

Enrichment Data: The Enrichment Data checkbox specifies whether the data selected in the project is considered to be enriched data. Enriched data will search the whole protein database regardless of the size of the database. Also, if there are <500 entries in the database, the whole database is used for searching glycan peptides as well. If Enrichment Data checkbox is unchecked and the database is greater than 500 entries, then the Database search result set of proteins is used for searching for glycan peptides. Setting this option may drastically increase computing time for large databases.

Search Glycopeptide Only: Reports only glycopeptides and does not report non-glycopeptide results from database search. This option is only available if the previous parameter “Enrichment Data” is selected.

NH4, Na, K: Select these to search for NH4, Na, or K adducts respectively.

Maximum Adduct Per Peptide: Sets the limit to the number of adducts on each glycopeptide.

Maximum Fucose: Sets the limit to the number of Fucose molecules for each glycan. O-link may have two glycans.

Composition or Structure: If selecting Composition based, Glycan PSMs with the same composition and different structures are merged and reported as one glycopeptide. If selecting Structure, Glycan PSMs with the same composition but different structures are reported as separate glycopeptides.

Click on “Report” to proceed to the final step. Report filters can be set here which will filter the final result.

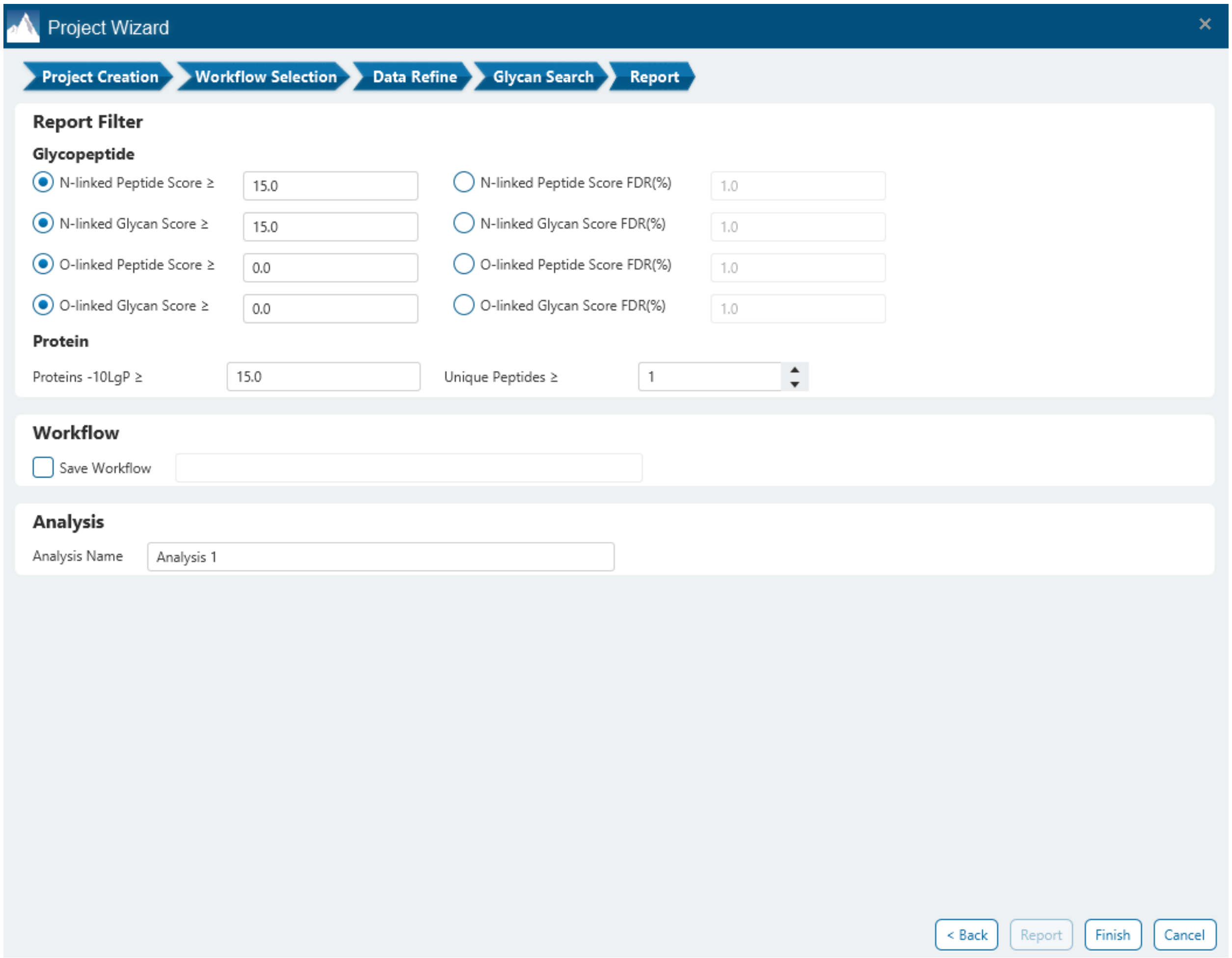

These filters are provided:

Score Filter: this section is a mandatory section to filter out some results under the user-defined filter. Any changes to the filter will create a new analysis with old parameters and update the results and statistics.

For each of N-link and O-link glycan search (if enabled), there is a Peptide Score and Glycan Score to be set.

Peptide score: The PEAKS peptide score (-10lgP) is calculated for every peptide-spectrum match (PSM) reported by PEAKS DB, PEAKS PTM, and SPIDER. The score is derived from the p-value that indicates the statistical significance of the peptide-spectrum match. A peptide may be matched to many spectra, resulting in multiple PSMs. In that case, the peptide's score is calculated as the maximum among all PSM scores. For details of the scoring algorithm, please refer to the publication, *"PEAKS DB: De Novo sequencing assisted database search for sensitive and accurate peptide identification ", Mol Cell Proteomics, 2011 Dec 20.* A minimum requirement can be set and all identifications must pass this filter.

Peptide FDR (%): The Peptides can also be set to use the false discovery rate. Peptides are scored based on their top PSM and using a “decoy-fusion” approach, the result is reported at that FDR.

Note: Decoy fusion is an enhanced target-decoy method for result validation with FDR. Decoy fusion appends a decoy sequence to each protein as the "negative control" for the search. See BSI's web tutorial (http://www.bioinfor.com/fdr-tutorial/) for more details.

Glycan Score: Glycopeptides above this score threshold will remain in the glycopeptide list.

Glycan Score FDR (%): Similar to Peptide FDR, the glycopeptides above the false discovery rate calculated for the whole set of glycopeptides will be reported.

Protein -10lgP: Proteins above the -10lgP (a combination of Peptide score and Glycan score) filter will be reported in the Protein table. This is an initial filter set that can be adjusted in the Summary page.

Unique Peptides: Proteins with this minimum # of unique peptides will be reported in the Protein table. This is an initial filter set that can be adjusted in the Summary page.

In the Report step, there also is the option to specify an Analysis Name (which can be later renamed) and/or decide to save the Workflow to quickly re-use in future analyses.

Click Finish to start running the project or analysis.

11.2 Understanding Glycan Search results

The Glycan Search result is the main result view. To open the Glycan Search result, double-click on the
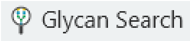

node in the Project View under any Analysis.

The Glycan Search view comes with 6 main tabs for result visualisation.

- Summary View
- Proteins View
- Glycopeptide View
- Peptide View
- Feature View
- Partial View

11.3 Glycan Search Summary View

The Summary View is selected by default when a Glycan Search result is opened. The 'Summary View' reports key statistics in table format as an overview of the result. Protein filters and Notes about the experiment can be set here. To select the Summary View, click on the 'Summary' tab.

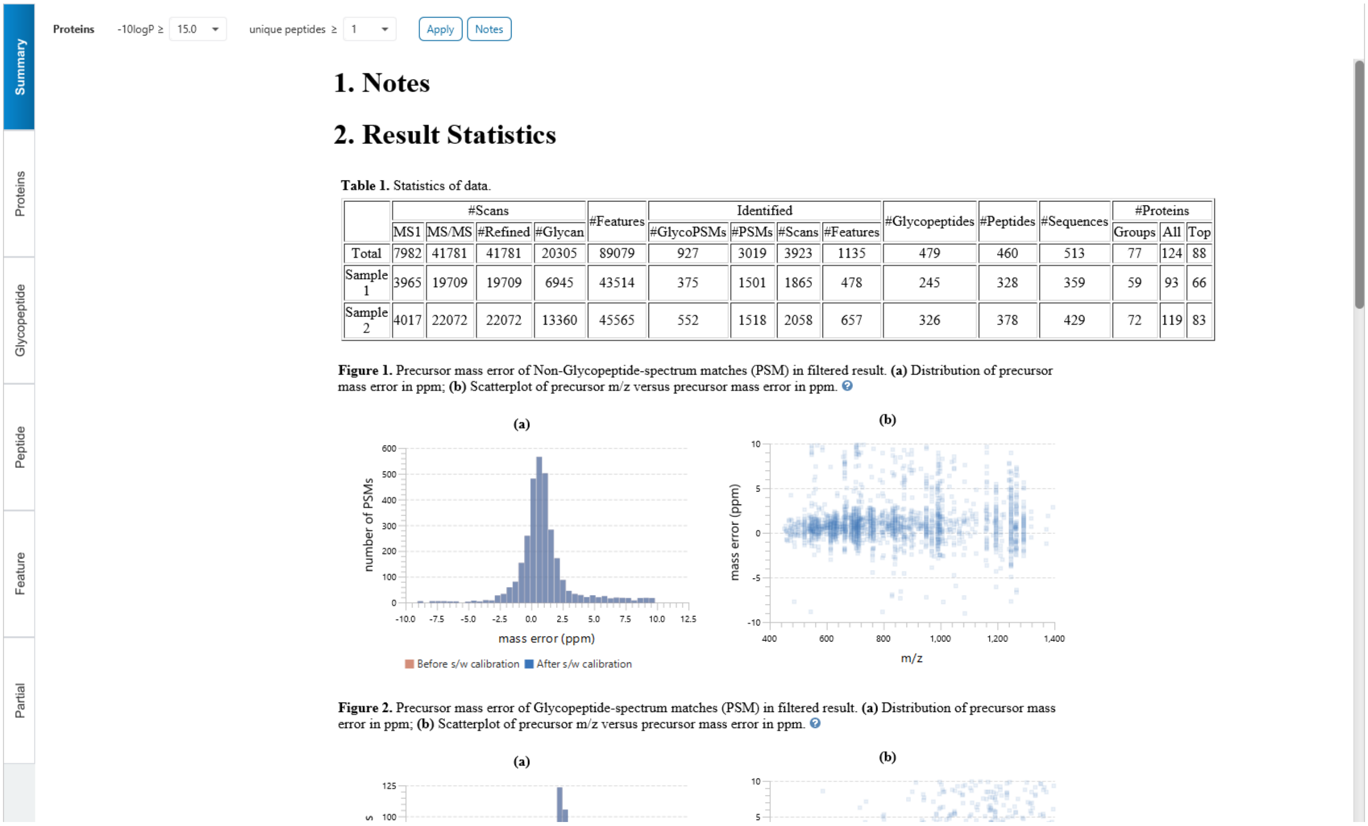

	#Scans				#Features	Identified				#Glycopeptides	#Peptides	#Sequences	#Proteins		
	MS1	MS/MS	#Refined	#Glycan		#GlycoPSMs	#PSMs	#Scans	#Features				Groups	All	Top
Total	7982	41781	41781	20305	89079	927	3019	3923	1135	479	460	513	77	124	88
Sample 1	3965	19709	19709	6945	43514	375	1501	1865	478	245	328	359	59	93	66
Sample 2	4017	22072	22072	13360	45565	552	1518	2058	657	326	378	429	72	119	83

11.3.1 Summary View - Notes

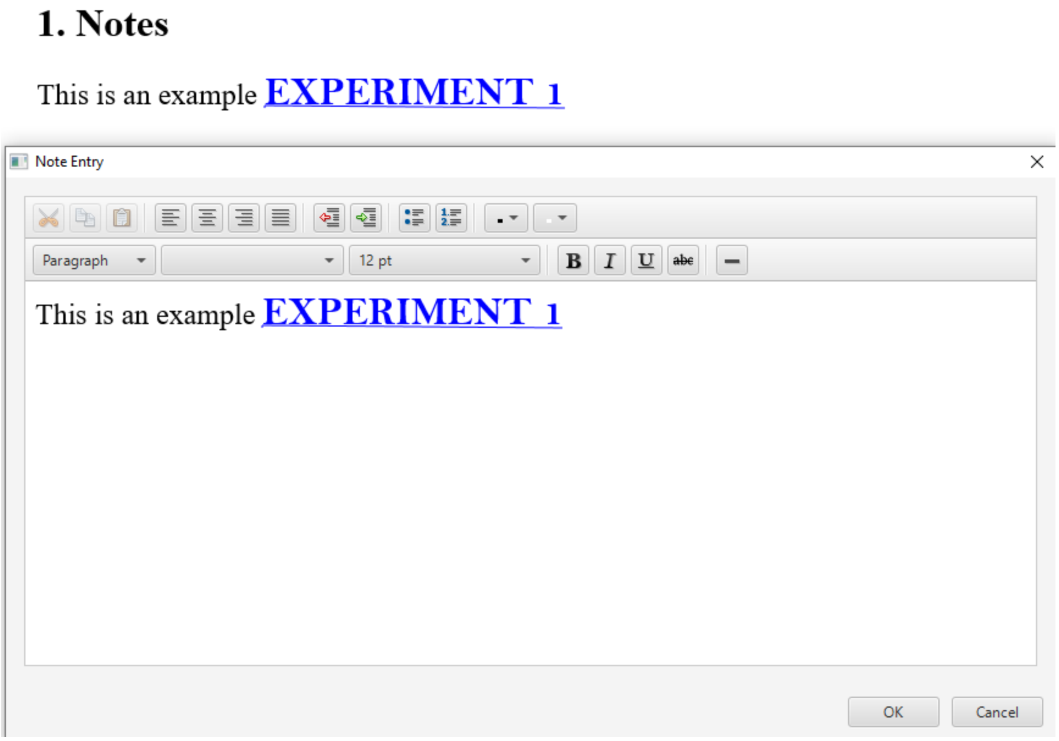

Click on the Notes button in the top bar to open the Note Entry editor. In the header of the report, information such as a description of the experiment can be entered. Lots of formatting options are provided to give the user flexibility on how they want to show this information. Clicking OK will save the notes into the result, which will remain with the project.

11.3.2 Summary View - Protein Filters

Protein Filters are provided in the Summary View.

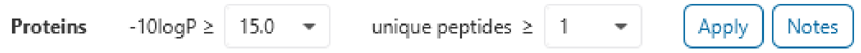

The Protein score (-10lgP) is calculated as the weighted sum of the -10lgP scores of the protein's supporting peptides. After removing any redundant peptides, the supporting peptides are sorted by -10lgP scores in descending order, and the k-th ranked peptide contributes to the weighted sum with a weight of 1/k.

The unique peptides filter is based on the number of unique supporting peptides for the protein.

Changing the -10lgP filter or unique peptides filter and clicking the red 'Apply' button will affect the Protein table in the Protein tab, and all proteins below the thresholds will be filtered out. This will also affect jumping functions from other tables (such as jumping from the Glycan table and Peptide table) as that protein is no longer accessible. The protein table exports will also be affected accordingly.

11.3.3 Summary View - Statistics of Data

2. Result Statistics

Table 1. Statistics of data.

	#Scans				#Features	Identified				#Glycopeptides	#Peptides	#Sequences	#Proteins		
	MS1	MS/MS	#Refined	#Glycan		#GlycoPSMs	#PSMs	#Scans	#Features				Groups	All	Top
Total	7982	41781	41781	20305	89079	927	3019	3923	1135	479	460	513	77	124	88
Sample 1	3965	19709	19709	6945	43514	375	1501	1865	478	245	328	359	59	93	66
Sample 2	4017	22072	22072	13360	45565	552	1518	2058	657	326	378	429	72	119	83

Table 1 Statistics of Data tabulates MS1 and MS2 information, as well as identification in the peptide and protein level. This table in the "Summary" view provides a condensed statistical information for the analysis.

For timsTOF Instruments, the statistics table has been modified to show key information about the #Frames and #Precursors.

11.3.4 Summary View - Non-glycopeptide Precursor mass error distribution

Figure 1. plots the distribution of non-glycopeptide PSMs in the filtered result. Figure 1. (a) shows the number of PSMs for each mass error in ppm. Figure 1. (b) shows the scatterplot of mass error in ppm across the m/z range

Figure 1. Precursor mass error of Non-Glycopeptide-spectrum matches (PSM) in filtered result. **(a)** Distribution of precursor mass error in ppm; **(b)** Scatterplot of precursor m/z versus precursor mass error in ppm.

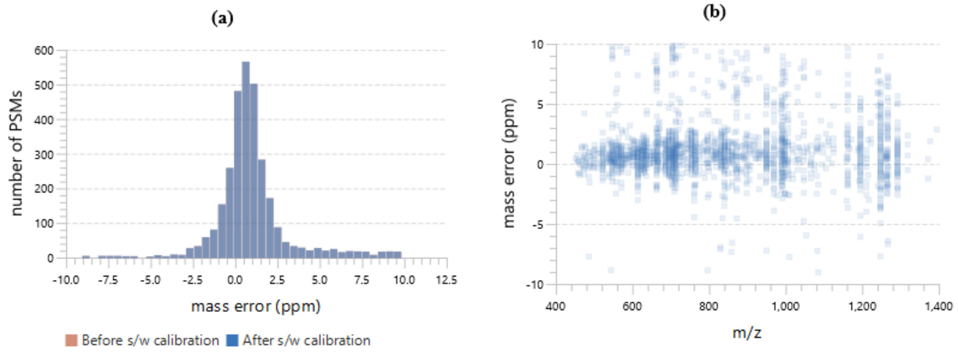

11.3.5 Summary View - Glycopeptide Precursor mass error distribution

Figure 2. plots the distribution of glycopeptide PSMs in the filtered result. Figure 2. (a) shows the number of PSMs for each mass error in ppm. Figure 2. (b) shows the scatterplot of mass error in ppm across the m/z range

Figure 2. Precursor mass error of Glycopeptide-spectrum matches (PSM) in filtered result. **(a)** Distribution of precursor mass error in ppm; **(b)** Scatterplot of precursor m/z versus precursor mass error in ppm.

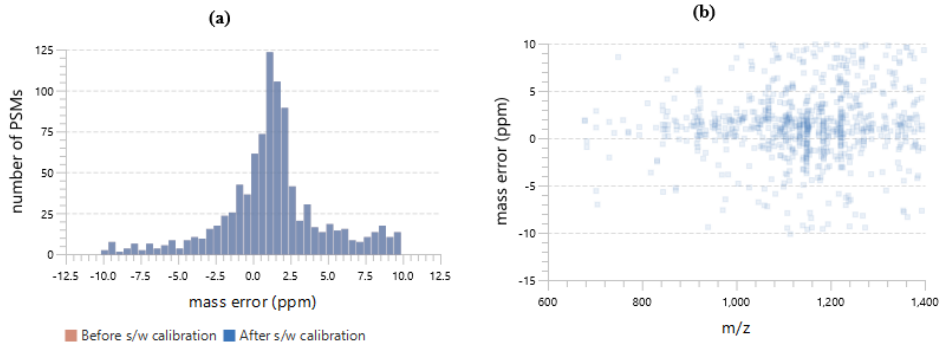

11.3.6 Summary View - Number of Glycosylation Sites (top 20)

Table 2. Number of glycosylation sites (top 20).

Glycan Moiety	Structure	Type	Sample 1	Sample 2
(HexNAc)4(Hex)5(NeuAc)2	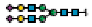	N-Link	32	39
(HexNAc)4(Hex)5(NeuAc)1	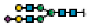	N-Link	10	16
(HexNAc)5(Hex)6(NeuAc)3	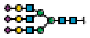	N-Link	10	14
(HexNAc)1		O-Link	6	12
(HexNAc)5(Hex)6(NeuAc)2	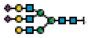	N-Link	9	9
(HexNAc)4(Hex)4(Fuc)2(NeuAc)2	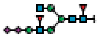	N-Link	3	10
(HexNAc)1(Hex)1(NeuAc)1		O-Link	4	8
(HexNAc)4(Hex)4(NeuAc)2	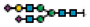	N-Link	9	2
(Hex)1		O-Link	6	5
(HexNAc)4(Hex)4	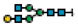	N-Link	4	6
(HexNAc)4(Hex)5	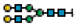	N-Link	6	4
(HexNAc)2(Hex)5	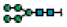	N-Link	5	5
(HexNAc)4(Hex)2(Fuc)2(NeuAc)1	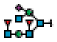	O-Link	3	7
(HexNAc)5(Hex)5(NeuAc)1	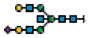	N-Link	3	6
(HexNAc)4(Hex)5(Fuc)1(NeuAc)2	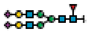	N-Link	3	5
(HexNAc)5(Hex)5(Fuc)1(NeuAc)1	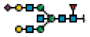	N-Link	3	5
(HexNAc)5(Hex)5(NeuAc)1		O-Link	3	5
(HexNAc)4(Hex)5(Fuc)1(NeuAc)1	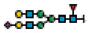	N-Link	2	6
(HexNAc)5(Hex)5(NeuAc)2	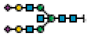	N-Link	3	5
(HexNAc)3(Hex)4(NeuAc)1	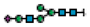	N-Link	2	6

Table 2 Number of Glycosylation Sites (top 20) shows the 20 most abundant glycans found in the result. It will list both N-linked and O-linked Glycans for combined N-link and O-linked Glycan searches. For every position in proteins in the protein table, if the glycan is found at that position it will be counted as a Glycosylation Site in this table. For the full Glycosylation Sites table, use the Glycosylation Sites CSV export from the Export node.

Note: If running with Glycan Composition only (instead of Structure based) in the Glycan Search workflow parameters, this table will not display the structure.

11.3.7 Summary View - Result Filtration Parameters.

Table 3 Result Filtration Parameters simply states the protein filters that are currently set in the Summary page. If the Summary page is used in a report, the protein filters set for that analysis report can be obtained here.

Table 3. Result filtration parameters.

Protein -10lgP	≥15
Proteins unique peptides	≥1

11.4 Glycan Proteins View

The ‘Proteins View’ lists protein identifications that have been filtered by the current settings in "Summary" view. It also visualizes the protein sequence coverage for identified proteins. The view has four main components:

- **Protein table:** This lists protein identifications that have been filtered by the current settings in the Summary View and the “Protein View Filters”.
- **Coverage:** This visualizes the protein sequence coverage of the selected protein, showing the mapping of support peptides and de novo tags to the protein sequence.
- **Peptides:** This lists supporting peptides mapped to the selected protein.
- **Glycopeptides:** This lists supporting glycan peptides mapped to the selected protein.

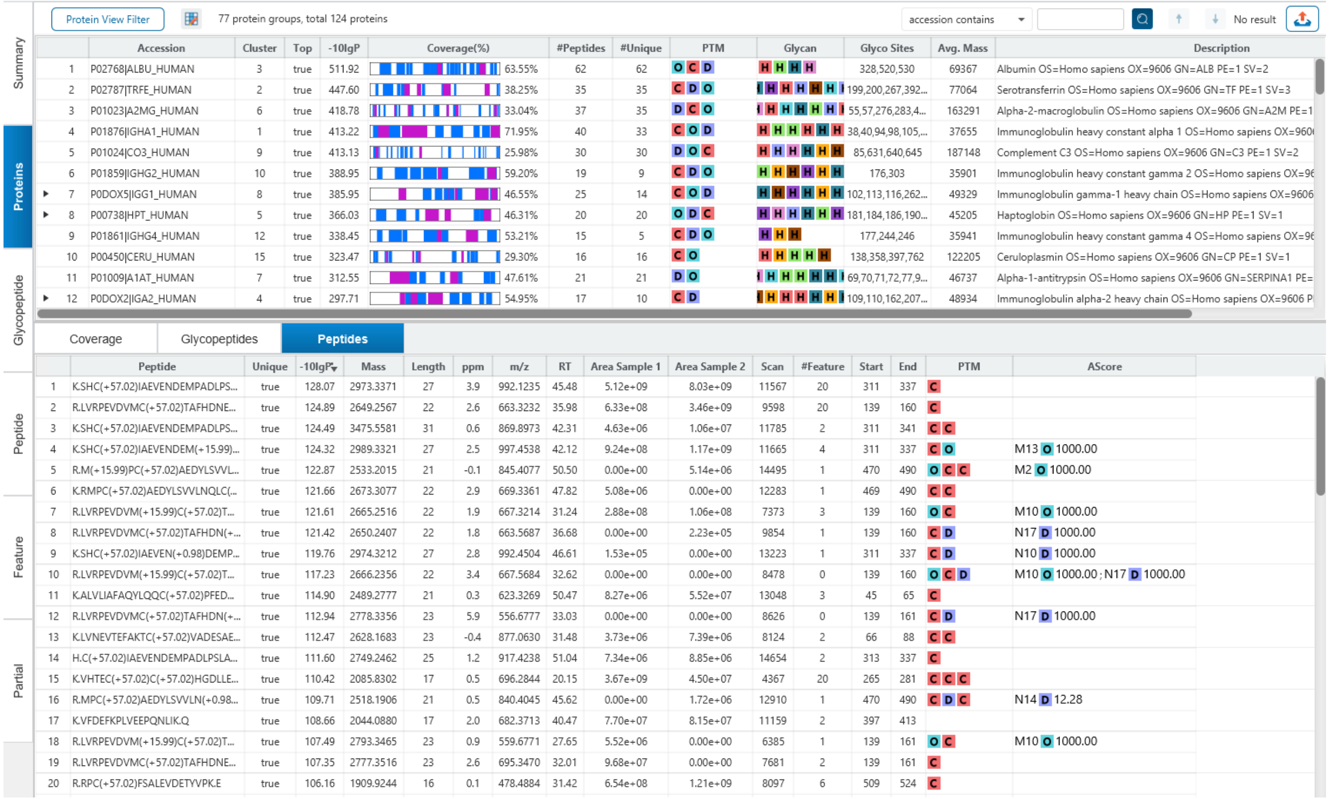

11.4.1 Protein Table

Each row in the table represents a group of proteins that are supported by a common set of peptides. A dark blue node indicates that the group has multiple proteins. To expand the group, click the " →" button at the left. To collapse the group, click the “↓” button.

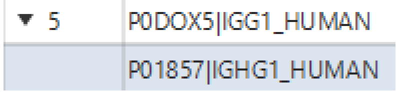

Note: A supporting peptide is considered highly confident if it has a peptide -10lgP score above the set threshold.

The columns in the "Protein" table are:

- **Accession:** The accession number of the protein as seen in the FASTA database.
- **Cluster:** This is a unique number to identify which protein group a protein belongs to. Those with the same number will be grouped together.
- **Top:** This column is provided to indicate if the protein is considered a top protein.
- **-10lgP:** The protein confidence score.
- **Coverage(%):** The percentage of the protein sequence that is covered by supporting peptides. The coverage is visualized by a colored bar. Purple blocks indicate the parts of the sequence covered by glycan peptides (either N-link or O-link). Blue blocks indicate the parts covered by high-confidence native peptides. This is the total coverage including results from all samples.
- **#Peptides:** The number of high-confidence supporting peptides (both glyco peptides and native peptides).
- **#Unique:** The number of high-confidence supporting peptides (both glyco peptides and native peptides) that are mapped to only one protein group. Unique peptides with same sequence but different modifications are only counted once in this number.
- **PTM:** The identified modifications displayed with color-coded icons. Mouse over the cell to see more details on which PTMs are included in the protein.
- **Glycan:** The identified glycans displayed with color-coded icons. Mouse over the cell to see more details on which glycans are included in the protein.
- **Glyco Sites:** The sites in the protein with glycans are listed here.
- **Avg. Mass:** The protein mass calculated using the average mass.
- **Description:** The protein's header information as seen in the FASTA database.

Note: For #Peptides and #Unique, two peptides with the same starting and ending positions in the protein are counted as one, regardless of their PTM forms. This is to follow the MCP (Molecular & Cellular Proteomics) guidelines.

Any columns in the protein table can be reorganized and shuffled. Most columns are able to be sorted.

11.4.2 Protein View Filters

Click on the 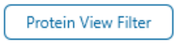

button to access the Protein View Filters.

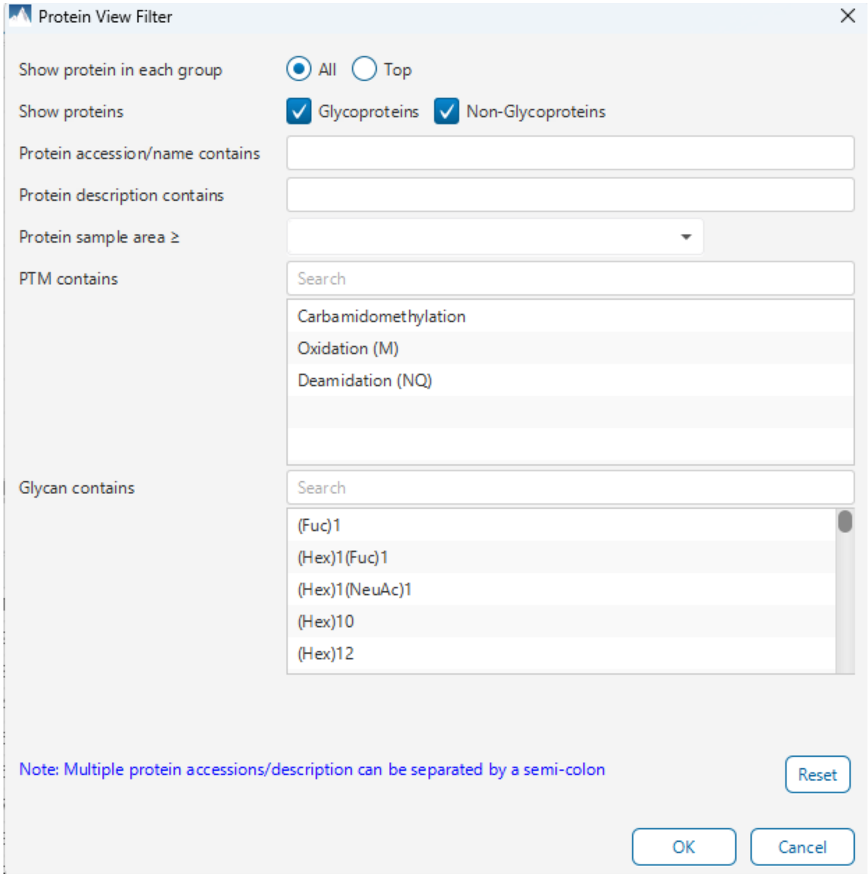

Show All or Top proteins in each group: Adjust the protein list based on Protein grouping, All is selected by default. Proteins are grouped based on parsimony.

- **All:** Show all proteins in each group. Proteins are grouped together if they are supported by the same set or a sub-set of the top protein in the group. If a protein is supported by a peptide not supporting the top protein it will be added to a new group.
- **Top:** Show only the top proteins in each group. These proteins are supported by the most unique peptides in the group. Proteins in the group that share a subset of the unique peptides that support the top protein (sub-proteins) will not be displayed.

Show Proteins checkboxes: Checkboxes for Glycoproteins and Non-Glycoproteins are provided. If Glycoproteins is checked, then proteins with supporting glycan peptides will show in the Protein table. If Non-Glycoproteins proteins is checked, then proteins with supporting native peptides will show in the Protein table. If neither are checked, the protein table would be empty.

Protein accession/name contains: Restrict the protein table to only display protein IDs which contain the entered text in its accession/name.

Protein description contains: Only proteins with descriptions that contain the entered text will be included in the protein table if text is entered in this field.

Protein sample area: Filter proteins based on total protein area found in each sample. Sample areas are calculated using the total of all peptide features from unique supporting peptides. If a protein is identified and has areas from multiple samples, only one of the samples must pass this filter to be included.

PTM contains: Filter protein results based on the presence of selected PTMs. Proteins that are supported by peptides containing modifications that match the selected PTMs will be included. If multiple PTMs match the modification name, they will all be included in the table.

Glycan contains: Filter protein results by the presence of selected Glycans. Proteins that are supported by glycan peptides containing the selected glycans will be included. If multiple glycans match the modification name, they will all be included in the table.

Note: In all filters, multiple entries can be separated by a semi-colon.

Click on the 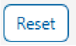

button to reset all filters to their defaults.

Click on the 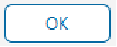

button to apply the changes.

Otherwise click on the 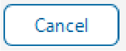

button to cancel the changes and keep the previous settings.

11.4.3 Protein Optional Columns

Click on the 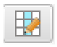 button to show the optional columns dropdown to show/hide additional columns in the protein table.

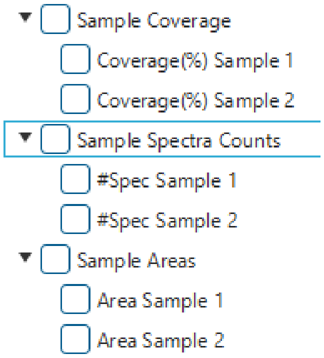

The options provided are sample level coverage, spectra counts, and areas.

Since there can be many samples in an analysis, all the sample level optional columns are not selected by default.

11.4.4 Protein Table Search Function

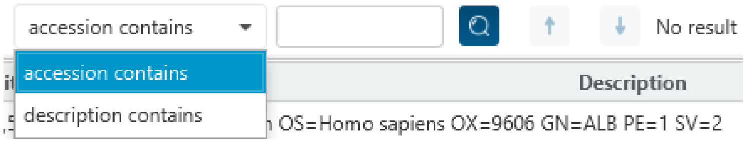

An easy to use search function is available for the protein table. The protein accession or description substring can be searched for and all search results can be navigated through with the Up and Down arrows.

11.4.5 Protein Coverage

The "Protein Coverage" view visually maps the supporting peptides and de novo tags to the protein selected in the Protein table. It also shows all identified sites with modifications or mutations to assist with protein characterization at the amino acid level. The "Protein Coverage" view is composed of three major components - Protein Sequence Coverage, Coverage Control Panel, and Protein Tools.

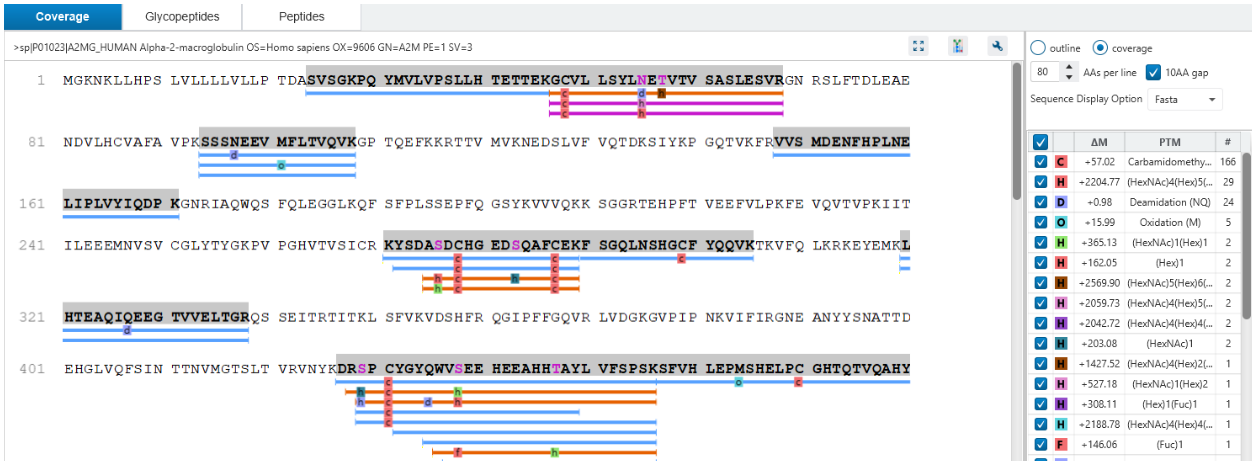

11.4.5.1 Protein Sequence Coverage

This area visualizes the coverage of the protein sequence.

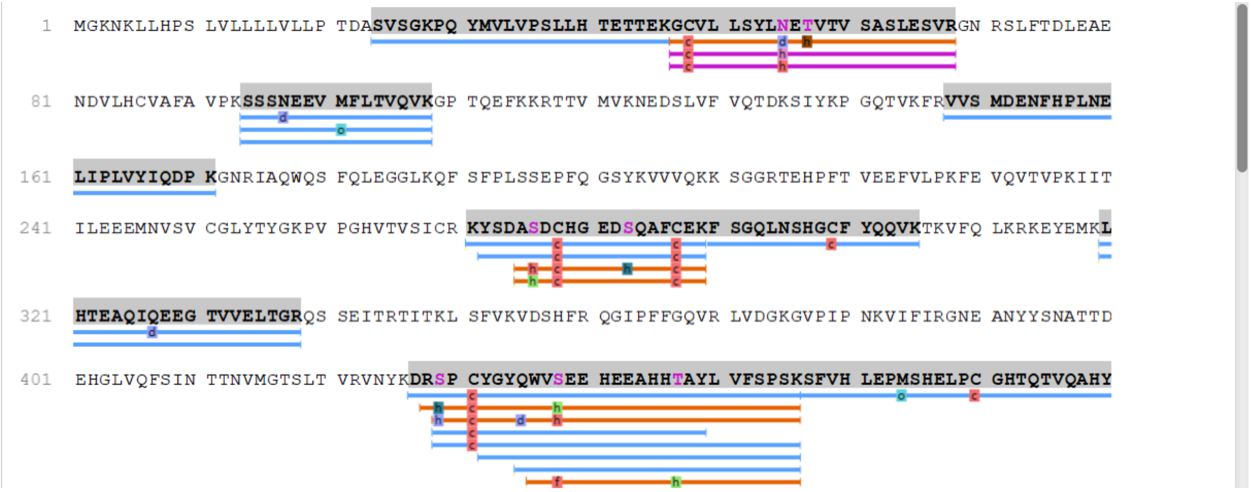

Regions in the protein sequence that are covered by supporting peptides are displayed in bold font with a grey background. Confident modifications identified in supporting peptides are displayed as icons above the protein sequence. Modifications are represented by colored icons with the first letter of its modification name.

The supporting native peptides and glycan peptides are shown as colored bars under the protein sequence. Non-glycopeptides are blue coloured, N-linked peptides are purple coloured, and O-linked peptides are orange coloured.

Placing the cursor over a bar shows a tooltip with detailed information of the peptide. Left clicking on a bar shows the annotated spectrum from which the supporting peptide is identified.

Right clicking on a peptide bar shows additional options. “Show in spectrum” has the same behaviour as left clicking on the peptide bar which is to open the annotated spectrum chart. “Show in table” has the function to jump to either the supporting glycopeptides table or supporting non-glycopeptides table.

Clicking on the 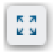 button will show the full screen view. Click on 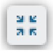 to return to the original view.

Click on the 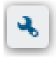 button reveals three more options for the protein coverage.

- Copy protein sequence: This will simply copy the protein sequence along with the header into the clipboard so that it can be easily pasted.
- Save image as: The image of the protein coverage can be exported with different sizes.
- Coverage statistics: Additional statistics about the coverage are shown here.

11.4.5.2 Glycan Profile

Clicking on 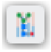 will open the Glycan Profile.

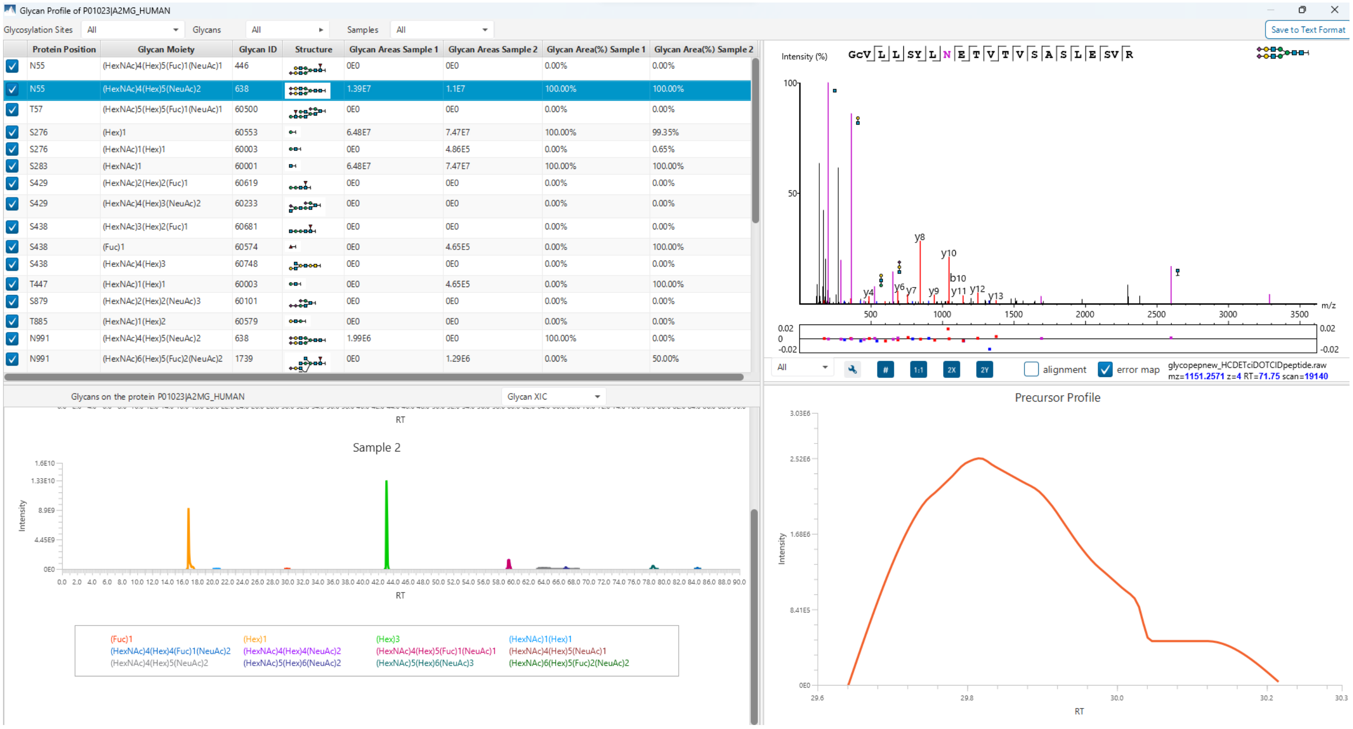

The Glycan Profile table will show all Glycan Moiety at every Protein Position. By default, all Glycosylation sites, Glycan Moiety, and Samples are selected. Click on the dropdowns above the table to select specific options to show in the table.

When selecting a row in the Glycan Profile table, the top right spectrum chart and the bottom right XIC chart is updated with the selection.

The bottom left chart has two options. The Glycan XIC chart shows the combined XIC of glycans for each of the selected samples. Mouse drag in the chart area to zoom in. The Glycan Site Profiles shows a bar chart of the area distribution between samples of each glycan moiety for this protein, pictured below.

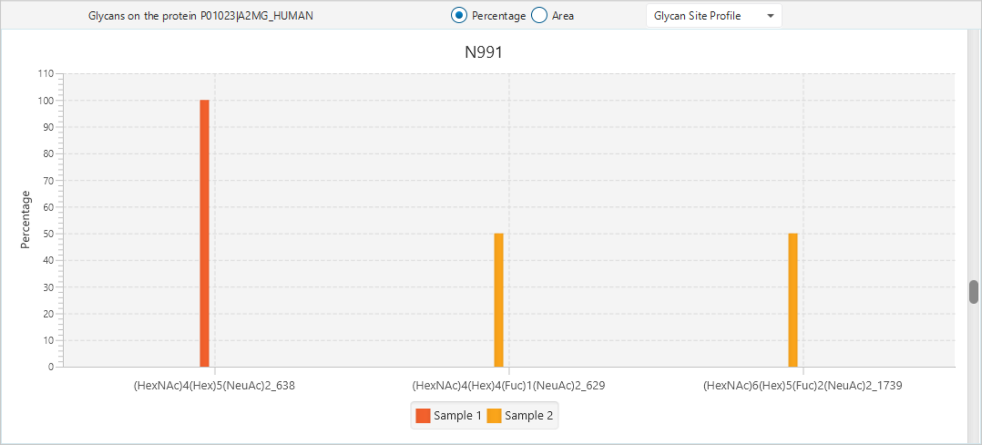

There is a radio button above the chart to switch between Percentage and Absolute area.

Right click on the chart area to export an image of the glycan profile chart.

On the top right there is the option to Save to Text Format. Click on the button 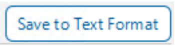 to export the Glycan Profile table in CSV format.

11.4.5.3 Glycan Pie Chart

In the protein coverage, a purple coloured amino acid in the residue indicates a glycosylation site. Click on it to open the Glycan Pie Chart.

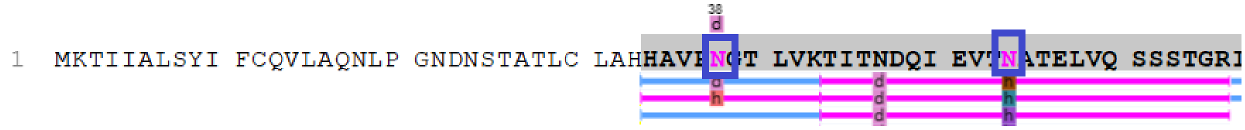

The Glycan Pie Chart shows the relative area of each glycan at that site. The top 7 glycans will be shown as slices in the pie chart and the rest will be categorized as 'Other'.

An accompanying table is provided for the actual area values detected for each sample at this protein site. Mouse over the Glycan moiety column to see the cartoon structure of that moiety. Mouse over the glycan profile or group profile to see a pop-up with the ratios for each sample compared to the base sample.

Some of the columns are sortable where necessary and can be rearranged if needed.

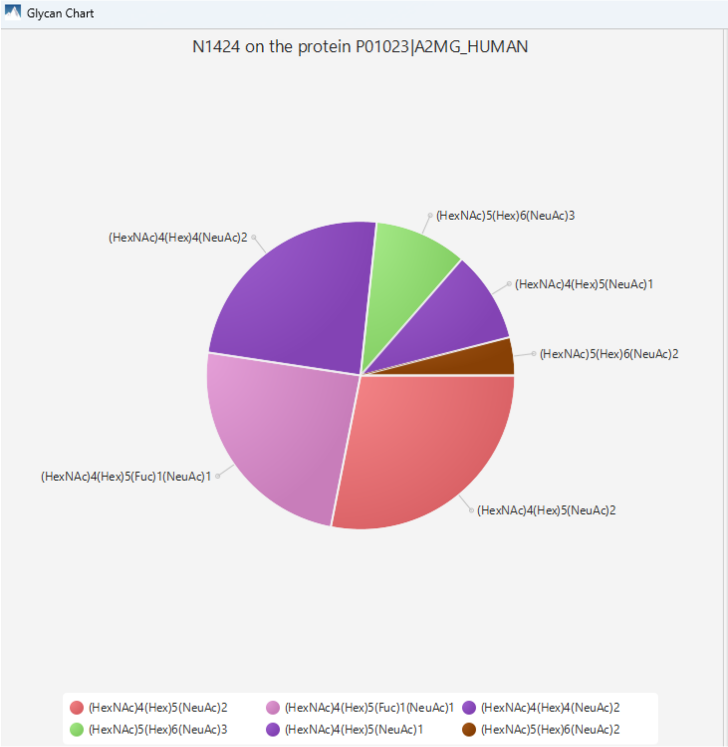

		Glycan moiety	Area(%)	#Feature
1	H	(HexNAc)4(Hex)5(NeuAc)2	28.08%	1
2	H	(HexNAc)4(Hex)5(Fuc)1(NeuAc)1	24.30%	1
3	H	(HexNAc)4(Hex)4(NeuAc)2	24.30%	1
4	H	(HexNAc)5(Hex)6(NeuAc)3	9.72%	1
5	H	(HexNAc)4(Hex)5(NeuAc)1	9.60%	1
6	H	(HexNAc)5(Hex)6(NeuAc)2	4.00%	1

11.4.5.4 Coverage Control Panel

This panel controls what to display in the protein sequence coverage view.

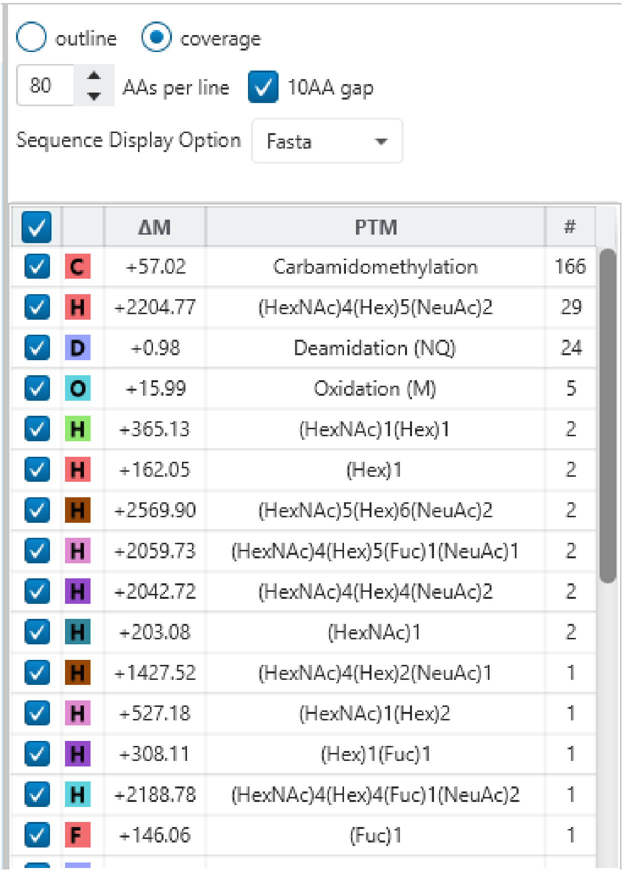

Sequence Display Mode: The protein sequence can be displayed in either "outline" or "coverage" mode.

“coverage” mode is the default mode to see the details of supporting glycan peptides and native peptides by viewing their coverage bars.

In “outline” mode, the protein sequence is displayed without supporting peptide bars. However, the sequence coverage at a specific position can be examined by left-clicking on a residue.

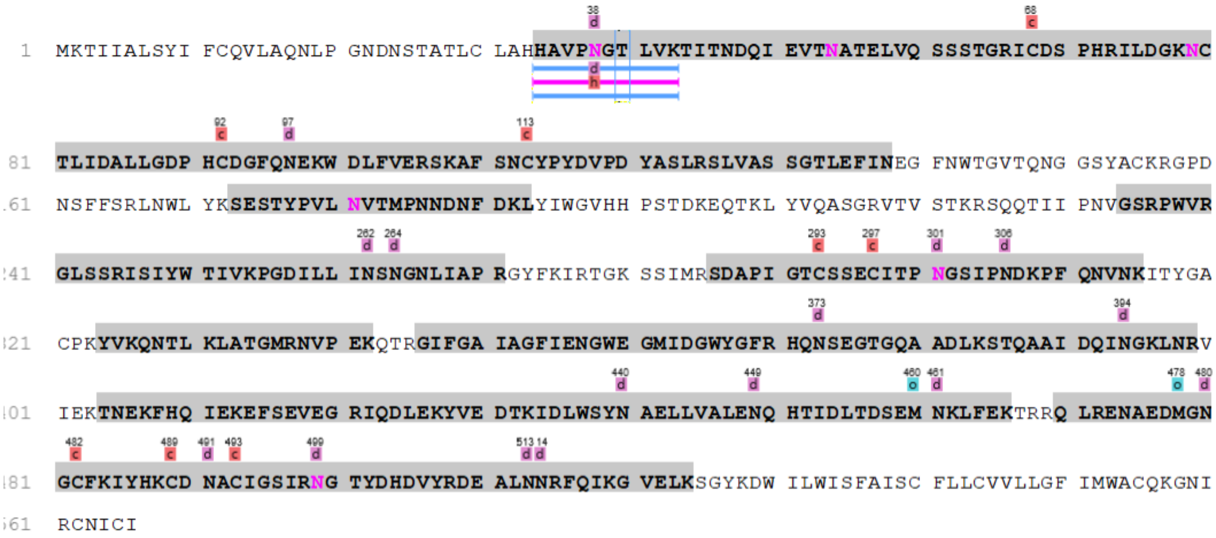

AAs per line and 10AA gap: Specify the number of amino acids per line and whether to show a gap for every 10 amino acids in the protein sequence.

Sequence Display Option: Select whether to view the protein sequence as Fasta or a specific enzyme used in the project. When a particular enzyme is selected, the protein sequence will be highlighted to emphasize expected peptides generated by the selected enzyme's cleavage specificity.

Modification Table: Also known as the PTM Table, specific glycans or PTMs can be deselected to hide from the protein coverage view. The # column indicates the number of instances that the modification is found in supporting peptides of this protein. Reminder that peptide bars are combined if they share the same start and end positions and have the same modifications.

11.4.6 Supporting Glycopeptides Table

The "Glyco Peptides" tab displays a table of supporting glycan peptides for the protein of interest. This table is similar to the Glycan table in the "Glycan" view, except that "Accession" is excluded and the columns "(Checkbox)", "Unique", "Start", and "End" are included. Descriptions of extra columns are given below. See 11.5.1 Glycan Table for all descriptions.

Coverage | **Glycopeptides** | Peptides

	Peptide	Unique	Glycan	Glycan Type	-10lgP	Glycan Score	Peptide Score	Mass	Length	ppm	m/z	RT	Area Sample 1	Area Sample 2	Scan	#Feature	Start	Enc
1	D.AS(+162.05)DC(+57.02)HGEDS(+...	true	(Hex)1;(HexNAc)1	OLink	43.99	58.67	29.30	2104.7834	15	1.1	702.6025	16.75	6.48e+07	7.47e+07	3215	2	275	2
2	K.VSN(+2204.77)QTLSLFFTVLQDVP...	true	(HexNAc)4(Hex)5(NeuAc)2	NLink	38.82	27.67	49.96	4366.9445	19	3.8	1092.7476	67.29	2.14e+07	0.00e+00	17836	1	1422	1
3	D.RS(+1880.67)PC(+57.02)YGYQW...	true	(HexNAc)4(Hex)3(NeuAc)2;(HexNA...	OLink	34.16	18.45	49.87	6672.7197	29	8.8	1113.1370	31.33	0.00e+00	0.00e+00	8064	0	428	4
4	K.GC(+57.02)VLLSYLN(+2204.77)ET...	true	(HexNAc)4(Hex)5(NeuAc)2	NLink	32.49	32.57	32.39	4600.9967	22	-0.1	1151.2571	71.75	1.39e+07	1.10e+07	19140	5	47	
5	K.VSN(+2042.72)QTLSLFFTVLQDVP...	true	(HexNAc)4(Hex)4(NeuAc)2	NLink	31.98	20.09	43.86	4204.8921	19	-1.0	1056.4860	59.34	0.00e+00	1.85e+07	17335	1	1422	1
6	K.VSN(+2861.00)QTLSLFFTVLQDVP...	true	(HexNAc)5(Hex)6(NeuAc)3	NLink	30.70	23.86	37.53	5023.1716	19	1.7	1256.8022	78.54	0.00e+00	7.39e+06	23399	1	1422	1
7	N.IYVLDYLN(+2204.77)ETQQLTPEIK.S	true	(HexNAc)4(Hex)5(NeuAc)2	NLink	27.83	23.86	31.79	4383.9122	18	1.2	1096.9874	54.95	1.99e+06	0.00e+00	14335	1	984	1
8	K.VSN(+2569.90)QTLSLFFTVLQDVP...	true	(HexNAc)5(Hex)6(NeuAc)2	NLink	26.79	21.33	32.25	4732.0761	19	1.2	1184.0277	66.88	0.00e+00	3.05e+06	19759	1	1422	1
9	Q.WVS(+146.06)EEHEEAHHT(+365....	true	(Fuc)1;(HexNAc)1(Hex)1	OLink	24.66	21.57	27.75	2993.3446	21	8.6	749.3499	29.81	0.00e+00	4.65e+05	7588	1	436	4
10	K.VSN(+1913.68)QTLSLFFTVLQDVP...	true	(HexNAc)4(Hex)5(NeuAc)1	NLink	24.40	22.23	26.56	4075.8491	19	1.0	1019.9705	59.44	0.00e+00	7.30e+06	17374	1	1422	1
11	E.QNMVLFAPNIYVLDYLN(+2188.78...	true	(HexNAc)4(Hex)4(Fuc)1(NeuAc)2	NLink	22.96	17.90	28.01	5382.4128	27	-3.2	1346.3544	84.42	0.00e+00	1.29e+06	25155	1	975	1
12	D.AS(+365.13)DC(+57.02)HGEDSQ...	true	(HexNAc)1(Hex)1	OLink	22.20	20.19	24.21	2104.7834	15	-5.6	702.5978	20.52	0.00e+00	4.86e+05	4511	1	275	2
13	F.APNIYVLDYLN(+2903.04)ETQQLT...	true	(HexNAc)6(Hex)5(Fuc)2(NeuAc)2	NLink	20.34	16.51	24.17	5364.3187	21	7.9	1346.3544	84.42	0.00e+00	1.29e+06	25150	1	981	1
14	K.VS(+1141.36)NQT(+1427.52)LSLF...	true	(HexNAc)1(Hex)2(NeuGc)2;(HexNA...	OLink	20.00	18.94	21.05	4731.0566	19	5.9	1183.7784	67.24	0.00e+00	0.00e+00	17803	0	1422	1
15	K.VSN(+2059.73)QTLSLFFTVLQDVP...	true	(HexNAc)4(Hex)5(Fuc)1(NeuAc)1	NLink	18.20	21.24	15.15	4221.9064	19	2.0	1056.4860	59.34	0.00e+00	1.85e+07	17340	1	1422	1
16	K.GC(+57.02)VLLSYLN(+2059.73)ET...	true	(HexNAc)4(Hex)5(Fuc)1(NeuAc)1	NLink	17.57	15.78	19.36	4455.9586	22	0.0	1114.9969	54.50	0.00e+00	0.00e+00	15786	0	47	
17	R.S(+876.32)PC(+57.02)YGYQ(+0.9...	true	(HexNAc)2(Hex)2(Fuc)1;(HexNAc)3...	OLink	17.34	15.17	19.51	5294.1833	28	7.1	1059.8514	28.96	0.00e+00	0.00e+00	7274	0	429	4
18	K.GC(+57.02)VLLSYLN(+0.98)ET(+2...	true	(HexNAc)5(Hex)5(Fuc)1(NeuAc)1	OLink	16.82	19.04	14.59	4660.0220	22	5.3	1166.0189	56.36	0.00e+00	0.00e+00	16373	0	47	
19	K.VS(+308.11)NQT(+1751.62)LSLFF...	true	(Hex)1(Fuc)1;(HexNAc)4(Hex)4(Ne...	OLink	16.36	15.70	17.02	4221.9064	19	3.8	1056.4879	59.98	0.00e+00	0.00e+00	15736	0	1422	1
20	F.APN(+0.98)IYVLDYLNET(+486.16)...	true	(Hex)3;(HexNAc)2(Fuc)1	OLink	15.61	15.70	15.51	3715.7587	23	-2.2	929.9449	43.09	0.00e+00	1.10e+08	12082	1	981	1

Unique: This shows whether the glycol peptide is a unique supporting glyco peptide to the protein group.

Start: This shows the peptide's starting position in the protein.

End: This shows the peptide's ending position (inclusive) in the protein.

Most columns are sortable and can be arranged where necessary.

11.4.7 Supporting Native Peptides Table

The Supporting Native Peptides table is also known as the “Peptides” tab and it displays a table of supporting native peptides for the protein of interest. This table is similar to the Peptide table in the “Peptide” view, except that "Accession" is excluded and the columns "(Checkbox)", "Unique", "Start", and "End" are included. Descriptions of extra columns are given below. See 11.6.1 Peptide Table for all descriptions.

Coverage | Glyco Peptides | **Peptides**

	Peptide	Unique	-10lgP	Mass	Length	ppm	m/z	RT	Scan	#Feature	Start	End	PTM	AScore
1	K.VFPLSLC(+57.02)STQPDGNVVIAC...	true	120.66	4777.3519	44	0.9	1195.3463	65.44	19261	6	8	51	C C	
2	R.NFPPSQDASGDLYTTSSQLTLPAT...	true	100.61	3167.5081	30	1.2	1056.8446	41.40	11666	5	52	81	C	
3	K.KGDTFSC(+57.02)MVGHEALPLAF...	true	99.83	2336.1293	21	1.5	585.0405	33.88	8901	2	307	327	C	
4	L.SLC(+57.02)STQPDGNVVIAC(+57...	true	98.49	4321.0783	40	-1.0	1081.2758	61.75	18077	4	12	51	C C	
5	C.STQPDGNVVIAC(+57.02)LVQGFF...	true	97.60	3960.9316	37	-0.7	991.2394	59.27	17303	4	15	51	C	
6	L.C(+57.02)STQPDGNVVIAC(+57.0...	true	93.98	4120.9622	38	-0.5	1031.2473	59.09	17282	2	14	51	C C	
7	L.C(+57.02)STQPDGNVVIAC(+57.0...	true	89.77	4121.9462	38	5.2	1031.4991	61.09	17878	1	14	51	C C D	Q16 D 10.53
8	N.VVIAC(+57.02)LVQGFFPQEPLSVT...	true	86.86	3261.6492	30	-0.7	1088.2229	55.04	15946	1	22	51	C	
9	K.GDTFSC(+57.02)MVGHEALPLAFT...	true	85.01	2208.0343	20	1.0	737.0195	38.61	9606	1	308	327	C	
10	K.SGNTFRPEVHLLPPPSEELALNELVT...	false	84.14	3572.8660	32	1.6	894.2252	50.25	14384	2	222	253	C	
11	R.QEPSQGTTTFAVTSILR.V	true	83.12	1834.9425	17	-0.1	612.6547	37.61	10188	4	283	299		
12	K.VFPLSLC(+57.02)STQPDGN(+0.9...	true	81.61	4779.3200	44	9.6	1195.8488	85.24	25433	1	8	51	C D C D	N14 D 0.00 ; Q22 D 0.00
13	C.LVQGFFPQEPLSVTWSESGQGVTA...	true	79.81	2719.3605	25	-0.2	907.4606	46.47	13177	0	27	51		
14	K.VFPLSLC(+57.02)STQPDGNVVIAC...	true	73.68	4778.3360	44	5.1	1195.5974	79.71	21493	0	8	51	C C D	Q22 D 7.38
15	H.LLPPPSEELALNELVTLTC(+57.02)L...	false	71.64	2448.3297	22	1.3	817.1183	57.35	16711	1	232	253	C	
16	K.VFPLSLC(+57.02)STQ(+0.98)PDG...	true	70.98	4779.3200	44	9.6	1195.8488	85.24	25402	1	8	51	C D C D	Q10 D 0.00 ; Q22 D 0.00
17	K.KGDTFSC(+57.02)MVGHEALPLAF...	true	70.09	2337.1133	21	9.3	585.2910	33.89	8162	0	307	327	C D	Q20 D 1001.00
18	K.SGN(+0.98)TFRPEVHLLPPPSEELA...	false	68.59	3573.8501	32	0.5	894.4702	52.96	15284	0	222	253	D C	N3 D 71.95
19	N.FPPSQDASGDLYTTSSQLTLPATQ...	true	68.22	3053.4651	29	1.5	1018.8306	41.53	11511	0	53	81	C	
20	K.VFPLSLC(+57.02)STQPDGNVVIAC...	true	59.14	4779.3200	44	9.6	1195.8488	85.24	25232	1	8	51	C C D D	Q22 D 8.28 ; Q27 D 2.33

Unique: This shows whether the native peptide is a unique supporting native peptide to the protein group.

Start: This shows the peptide's starting position in the protein.

End: This shows the peptide's ending position (inclusive) in the protein.

Most columns are sortable and can be arranged where necessary.

11.5 Glycan Search Glycopeptide View

The "Glycopeptide View" displays the resulting glycan peptide identifications. The table on the top shows the details of each peptide identification. The bottom section provides additional information about the glycan peptide-spectrum matches for the peptide selected in the table.

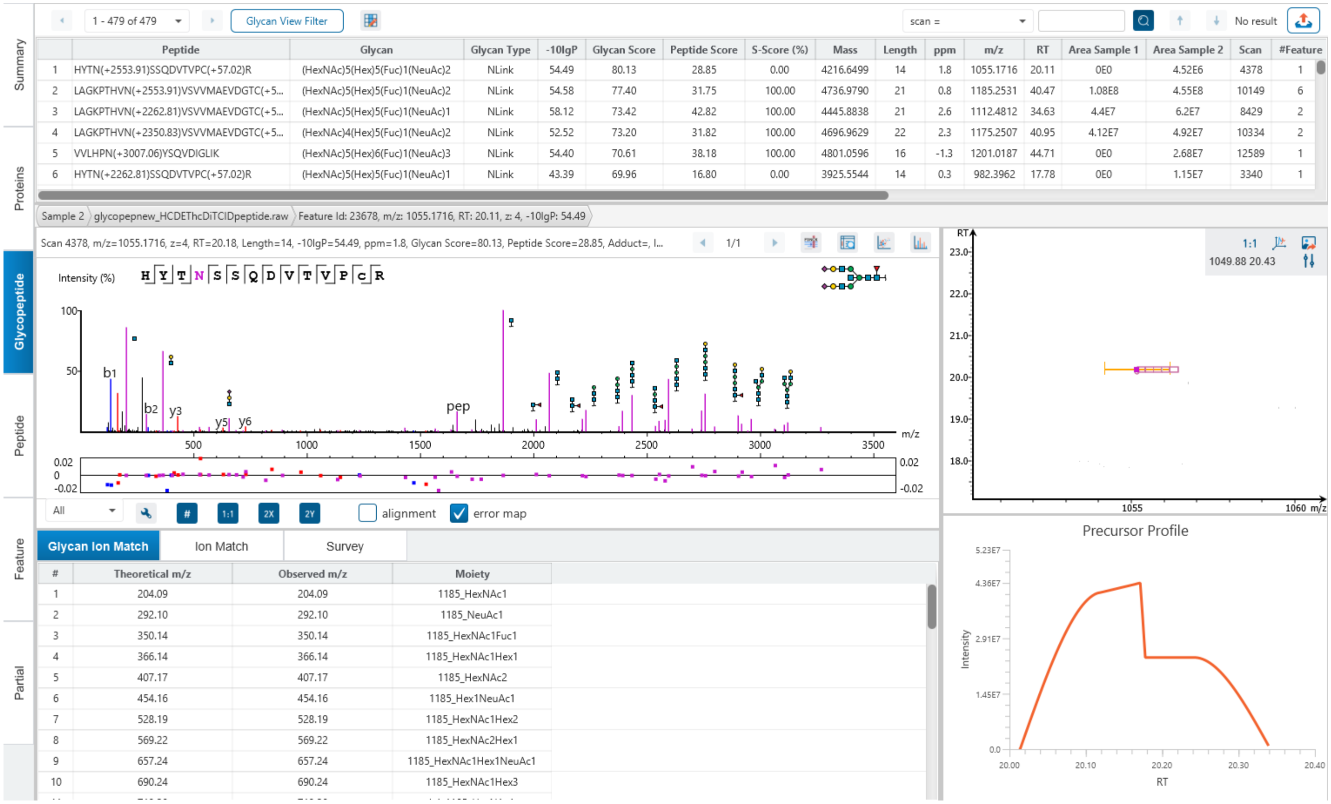

11.5.1 Glycan Table

The Glycan Table shows the filtered glycan peptide identification results. Each row in the table is a glycopeptide identification represented by its highest-scoring PSM. When there are more than 1000 peptides, the table is split into multiple pages.

Note: If there are multiple PSMs matched to a peptide, the table displays the information for the top-scoring PSM. Peptides with the same sequence but different modifications are considered different and shown in different rows.

	Peptide	Glycan	Glycan Type	-10lgP	Glycan Score	Peptide Score	S-Score (%)	Mass	Length	ppm	m/z	RT	Area Sample 1	Area Sample 2	Scan	#Feature
1	HYTN(+2553.91)SSQDVTVPC(+57.02)R	(HexNAc)5(Hex)5(Fuc)1(NeuAc)2	NLink	54.49	80.13	28.85	0.00	4216.6499	14	1.8	1055.1716	20.11	0E0	4.52E6	4378	1
2	LAGKPTHVN(+2553.91)VSVVMAEVDGTC(+5...	(HexNAc)5(Hex)5(Fuc)1(NeuAc)2	NLink	54.58	77.40	31.75	100.00	4736.9790	21	0.8	1185.2531	40.47	1.08E8	4.55E8	10149	6
3	LAGKPTHVN(+2262.81)VSVVMAEVDGTC(+5...	(HexNAc)5(Hex)5(Fuc)1(NeuAc)1	NLink	58.12	73.42	42.82	100.00	4445.8838	21	2.6	1112.4812	34.63	4.4E7	6.2E7	8429	2
4	LAGKPTHVN(+2350.83)VSVVMAEVDGTC(+5...	(HexNAc)4(Hex)5(Fuc)1(NeuAc)2	NLink	52.52	73.20	31.82	100.00	4696.9629	22	2.3	1175.2507	40.95	4.12E7	4.92E7	10334	2
5	VVLHPN(+3007.06)YSQVDIGLIK	(HexNAc)5(Hex)6(Fuc)1(NeuAc)3	NLink	54.40	70.61	38.18	100.00	4801.0596	16	-1.3	1201.0187	44.71	0E0	2.68E7	12589	1
6	HYTN(+2262.81)SSQDVTVPC(+57.02)R	(HexNAc)5(Hex)5(Fuc)1(NeuAc)1	NLink	43.39	69.96	16.80	0.00	3925.5544	14	0.3	982.3962	17.78	0E0	1.15E7	3340	1

To copy the sequence, right click on a row (or multiple rows) in the table and select “Copy selected peptide sequence(s)”. Another option is to mouse over any cell in the table and hitting Ctrl + C on your keyboard to copy the contents of those selected cells.

The following list describes the contents in each of the default columns:

- **Peptide:** The amino acid sequence of the glycan peptide, as determined by the Glycan Search workflow. A modified residue is followed by a pair of parentheses enclosing the modification or glycan mass.
- **Glycan:** The Glycan moiety on this glycopeptide. For N-link results, there will always be 1. For O-link results, there could be more than 1.

- **Glycan Type:** Simply states whether the glycan moiety is N-link or O-link. This column is particularly useful for N-Link + O-link search workflows.
- **-10lgP:** The peptide -10lgP score. The score indicates the scoring significance of a peptide-precursor spectrum match.
- **Glycan Score:** The normalized sum of ions log intensity, based on glycan fragment ion, peptide ion, and glycopeptide ion (Y ion).
- **Peptide Score:** Classifies glycan peptides with the normalized sum of ions log intensity, based on peptide fragment ions, and those peptide fragment ions with glycan. Different fragmentation type considers different peptide fragment ion:
    - CID and HCD considers b,y ion without glycan and b,y ion with at most 1 glycan node from root
    - ETD considers b,y,c,z,z' ion without glycan and b,y,c,z,z' ion with whole glycan from root
    - ETHCD considers b,y,c,z,z' ion without glycan, b,y,c,z,z' ion with 1 glycan from root, and b,y,c,z,z' ion with whole glycan from root
- **S-Score (%):** Measures the confidence of the top glycopeptide candidate as opposed to other candidates for the spectrum. For glycan candidates with the same composition and same peptide, the candidate is sorted by matched glycan y-ion count. *S-Score = (most y-ion count - 2nd most y-ion count)/(most y-ion count).* The higher the score the better. 100% indicates only 1 result, and it is the best match. 0% indicates that the top1 and top2 result are very similar, and we cannot confidently say the result is the best match.
- **Mass:** The monoisotopic mass of the glycan peptide.
- **Length:** The number of amino acids in the sequence backbone.
- **ppm:** The precursor mass error, calculated as 10^6 × (precursor mass - theoretical peptide mass)/(theoretical peptide mass).
- **m/z:** The precursor mass-to-charge ratio.
- **RT:** The retention time (elution time) of the spectrum as recorded in the data.
- **Area per Sample:** The combined area for all features in that sample associated with this peptide.
- **Scan:** The scan number of the spectrum that matches the peptide sequence with the highest -10lgP.
- **#Feature:** For single sample analysis, shows number of features assigned to the peptide sequence from identification. For multiple samples, if peptide has feature vectors, it will show number of features used (area > 0) in all feature vectors. If not, it shows the number of features from identification.
- **Id Count:** Number of identifications in total.
- **Adduct:** Indicates whether this glycopeptide has an adduct and lists which adduct.
- **Isotopic Shift:** Indicates the isotopic mass shift.
- **Accession:** The accessions of proteins that contain this glycan peptide.
- **PTM:** The types and the numbers of modifications present in the peptide shown in color-coded icons.

- **AScore:** Localization score assigned to modifications on the peptide. It is the -10 log of a p-value. In this case, the p-value is the probability that the modification occurs at the reported position compared to other possible positions. A -10lgP of 20 is equal to a p-value of 0.01. Scores are listed in the format AminoAcid, peptide position, modification, AScore. For example, serine at amino acid 3 is phosphorylated with an AScore of 54.01 will be reported as: S3 P 54.01.

11.5.2 Glycan Table controls

The menu above the peptide table provides options to scroll through the table of glycan peptides, filter the table, show optional columns and search for specific entries.

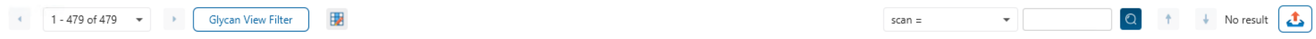

11.5.2.1 Glycan Table controls - Pagination

For easy viewing and to improve performance, the glycan table is limited to showing 1000 entries at once. Pagination is used to manage which set of 1000 entries will be viewed.

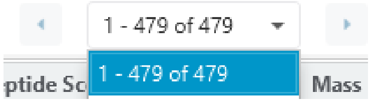

In the top left, there is a dropdown to select the rows for viewing. Click the left arrow or right arrow to select the previous/next 1000 rows. If the table is sorted or filtered, it may update the pagination set of rows that is currently in view.

11.5.2.2 Glycan Table controls - View Filter

Click on the 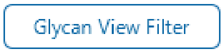 button to open the Glycan View Filter which is used to filter the Glycan table. The following are the filter options provided:

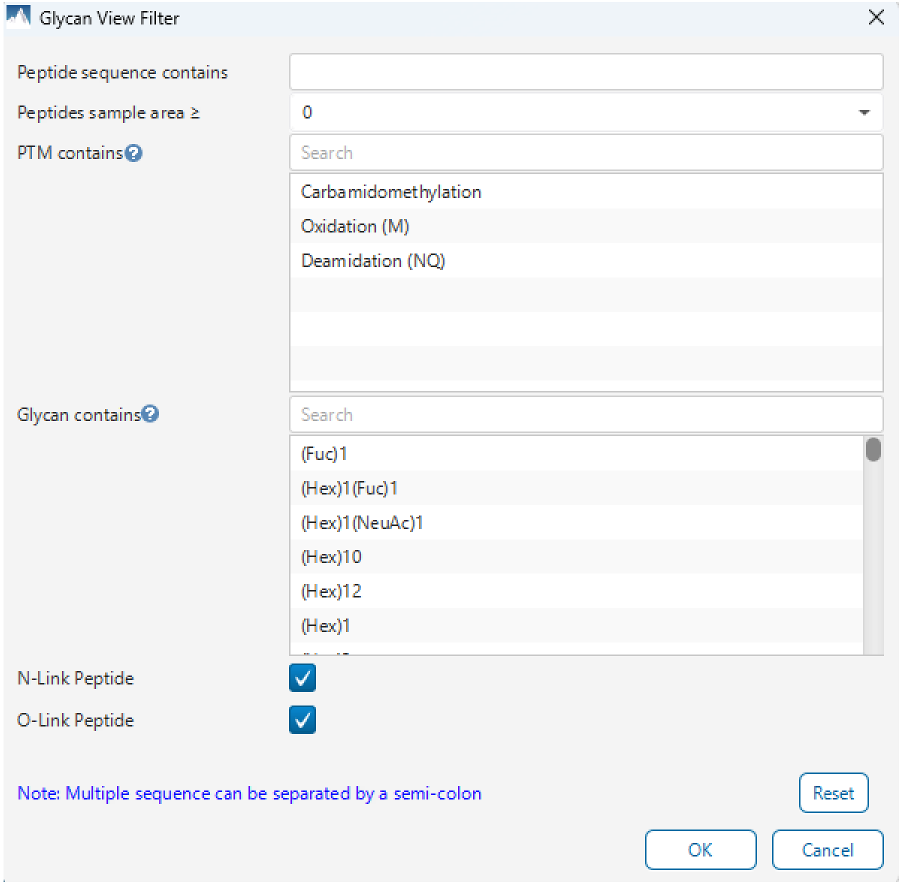

- **Peptide sequence contains:** Enter an amino acid sequence, only peptides that contain that sequence will be displayed in the peptide table. Adding PTMs or Glycans in brackets in the sequence search is not handled, meaning only the sequence backbone is considered in the filter. Use the PTM contains or Glycan contains for further filtering if necessary.
- **Peptide sample area:** Filters based on total peptide area found in each sample. Sample areas are calculated using the total of all peptide features from all spectra that identified the glycan peptide. If the peptide is found in multiple samples, only one sample must pass this filter for the peptide to be included.
- **PTM contains:** Type the names of PTMs you would like to include. Peptides containing PTMs that match the text will be included. If multiple PTMs match the text, they will all be included in the table.
- **Glycan contains:** Type the names of Glycans you would like to include. Peptides containing Glycans that match the text will be included. If multiple Glycans match the text, they will all be included in the table.
- **N-link Peptide and O-link Peptide checkboxes:** These checkboxes are only provided for N-link + O-link Glycan Search workflow. This is to select/deselect showing either type of Glycan in the table.
- **Note:** In all filters, multiple entries can be separated by a semi-colon.

Click on the Reset button to reset all filters to their default setting.

Click on OK to apply the filters to the Glycan table.

Click on Cancel to discard the changes in the View Filter.

11.5.2.3 Glycan Table controls - Optional Columns

Click on the 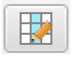 button to open the optional columns dropdown. By default the sample area columns are displayed. Sample Feature count columns can also be shown. For projects with many samples, it might be useful to hide these optional columns and reduce the width of the glycan table.

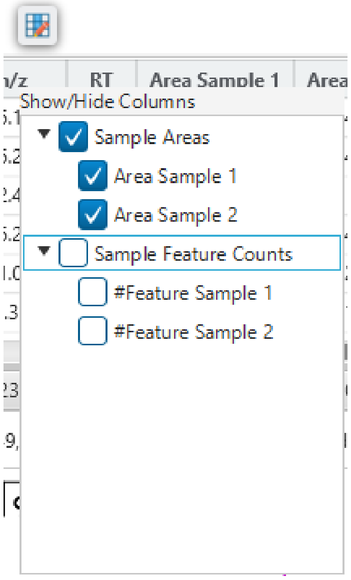

11.5.2.4 Glycan Table controls - Search function

On the top right of menu, there is a search function with 4 options. Search results can be navigated between using the Up or Down arrows.

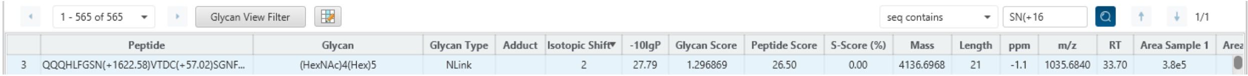

Options for searching include:

- **Scan =:** Search for the Scan number associated with each peptide. One scan can be associated to multiple peptides. For projects with multiple data files, the scan number is searched on every fraction.

- **m/z ≈:** Search for the approximate m/z. The search will consider a range of m/z equal to the search value +/- 1 of the last significant digit.

For example, searching for m/z ≈ 580.8 will find all m/z between [580.7, 580.9]

- **RT ≈:** Search for the approximate RT. Likewise, the search will consider a range of RT equal to the search value +/- 1 of the last significant digit set.

For example, searching for RT ≈ 64 will find all m/z between [63, 65]

- **Seq contains:** Search for the sequence in the table. If specifying a sequence without glycans or PTMs, the results will include that sequence and any sequences with glycans or PTMs with the same backbone. If specifying a sequence with bracket and some mass value (like in the above image), then only sequence that have a substring with an exact match will be considered.

Additionally, since the glycan peptide table shows both I/L and they can not be distinguished, then searching for I in the sequence is equivalent to searching for L in the sequence. i.e. Searching for “LVLK” is the same as searching for “LVIK”, “IVLK”, and “IVIK”

11.5.3 Feature selection breadcrumb

There is a sample and feature selection breadcrumb which allows you to switch between features associated with the peptide. Click on the Sample in the breadcrumb to reveal other samples and the features in that sample related to the current glycan peptide selection. From here you can select other samples, fractions, or features.

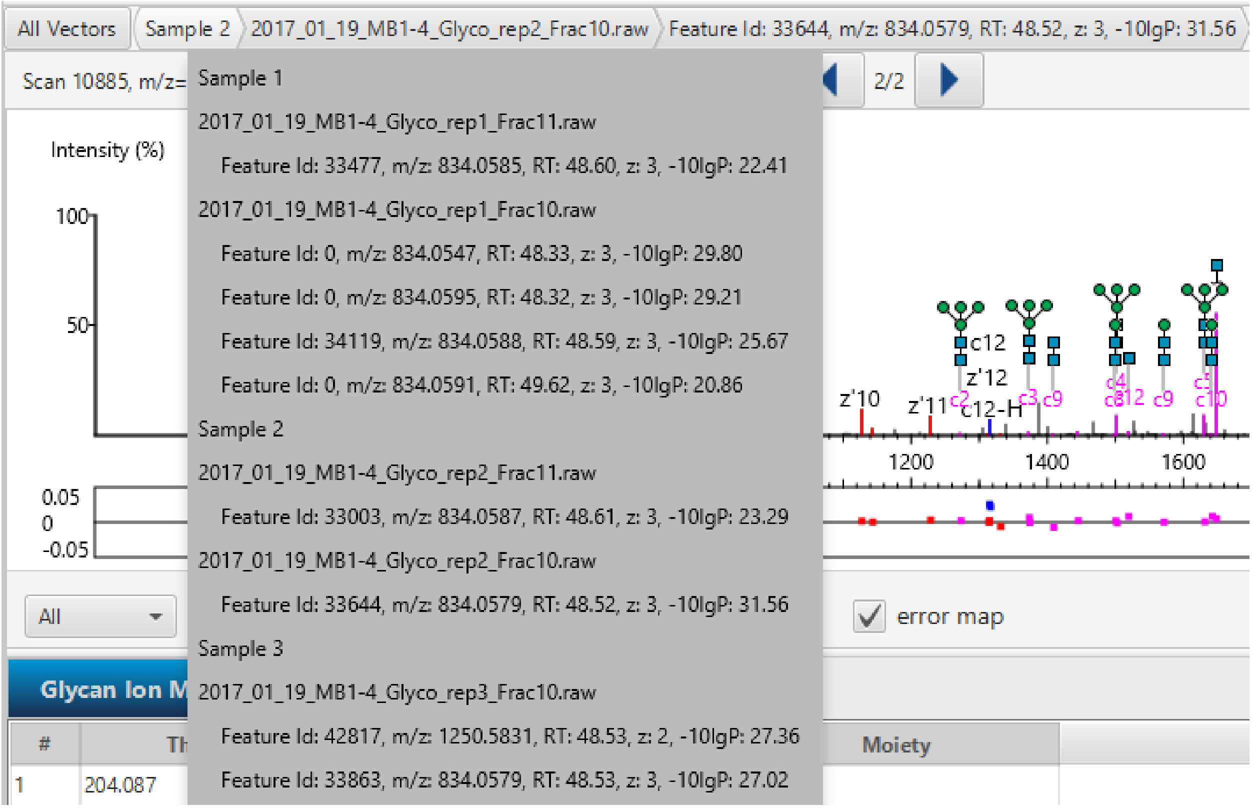

By clicking on the fraction level, all fractions in this sample are revealed as well as the features in each fraction associated with this peptide. From here you can select other fractions and features to view.

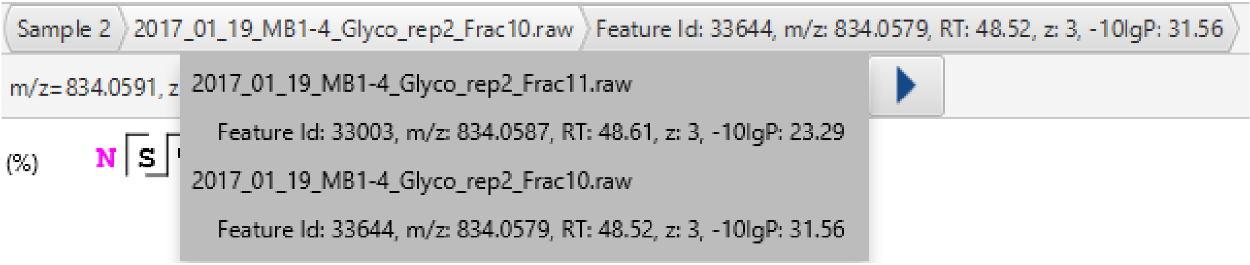

Lastly, clicking on the feature level will show all features in the currently selected fraction. Features with Feature Id = 0 are known as fake features and are constructed to enhance identification.

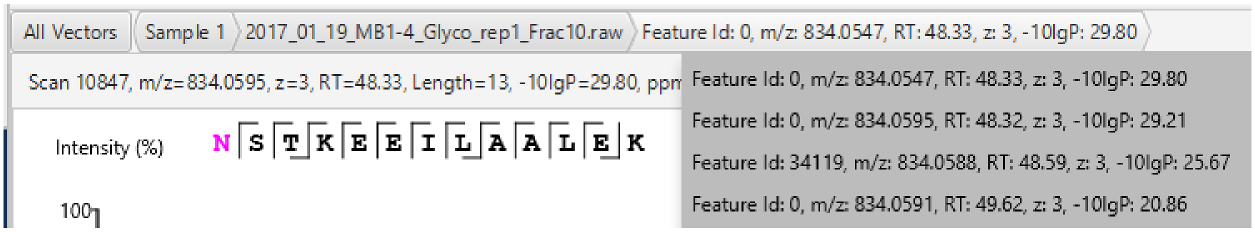

11.5.4 PSM selection

Underneath the feature breadcrumb will shown the currently selected PSM. Click on the Left and Right arrow buttons to switch between PSMs related to the currently selected feature.

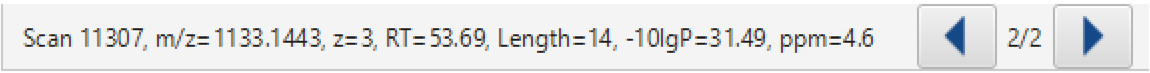

All other components in the Glycan tab interface will update according to the currently selected PSM.

For timsTOF instruments, Precursor ID is used rather than Scan number.

11.5.4.1 Protein jump button

Click on [icon] to jump to a protein that contains the currently selected glycan PSM. If there are more than 1 protein, a dropdown is revealed which allows the user to select which protein to jump to. If a protein is filtered out due to a protein summary filter or summary page protein filter, then the option to jump will be grayed out.

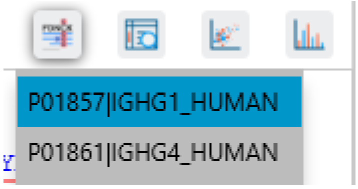

11.5.4.2 Show spectrum in Data View

Click on [icon] to open the Data Refine MS/MS tab (if not already opened) and jump to the corresponding selected Scan.

11.5.4.3 Show spectrum in LC/MS View

Click on [icon] to open the Data Refine LC/MS tab (if not already opened) and jump to the corresponding selected Scan, highlighting the Glycopeptide MS/MS marker and the feature if present.

11.5.4.4 Show Raw Spectrum View

Click on [icon] to show the raw spectrum chart of the currently selected Scan.

11.5.5 Annotated Spectrum Chart

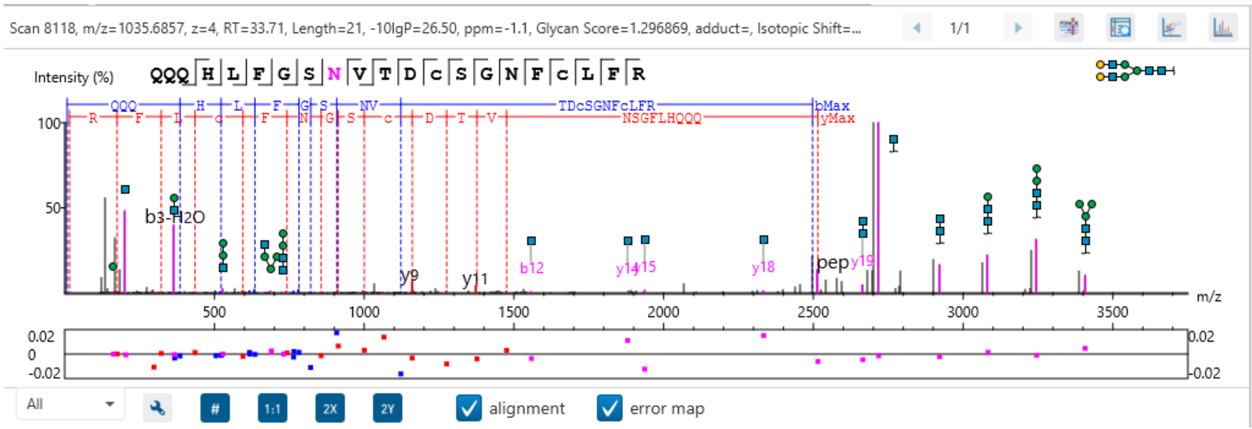

For the currently selected PSM, a graphical representation is shown in the form of an annotated spectrum chart. There are several controls provided for this view which will be explored in the following sections.

11.5.5.1 Annotated Spectrum Chart - Chart Navigation

Moving the cursor over the peptide sequence in the spectrum will show the mass transitions for a particular amino acid residue.

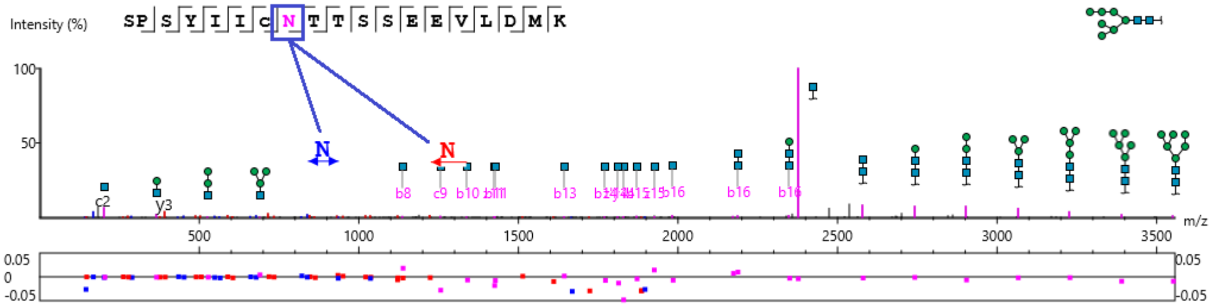

Zoom to a m/z region: Drag horizontally from the start m/z and to the end m/z with left mouse button.

Zoom in/out smoothly: Place the cursor at a particular m/z value (right below the x-axis), zoom in/out by scrolling the mouse wheel.

Increase/Decrease peak intensity: Place the cursor in the spectrum and scroll the mouse wheel.

See the whole spectrum: Double click in the spectrum or click the 1:1 button.

11.5.5.2 Annotated Spectrum Chart - Spectrum Annotation Settings

Click on the 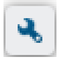 button to show the spectrum annotation settings.

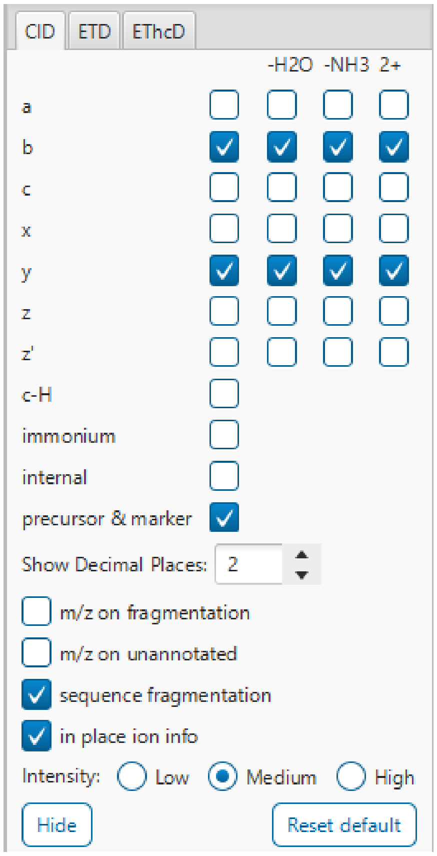

The spectra in the PEAKS results can be annotated by selecting ion types from the thorough collection of ions that PEAKS offers. The selected ion types will be displayed in the “Ion Match” table, as well. It is possible to annotate the spectrum with various ions for CID, EThcD and ETD. Each of these fragmentation modes can be set with different settings which will be shared with other results with the same fragmentation type.

To reset the defaults, click on the “Reset default” button.

Show Decimal Places: Select the number of decimal places that will appear in the ion table and the spectrum view. The default is set to two decimal places.

m/z on Fragmentation: Select this to display the m/z value on top of the annotated ions.

m/z on Unannotated: Select this to display the m/z value on top of the peaks without ion matches.

Sequence Fragmentation: Select this to display the sequence fragmentation on the top-left corner of the “Spectrum Annotation” view.

In Place Ion Info: Ion information, m/z value,and relative intensity are all displayed in a pop-up within the “Spectrum Annotation” view when this option is checked and the cursor is placed on a peak.

Intensity: Set the intensity threshold for spectrum annotation to low (2%), medium (5%), or high (10%).

11.5.5.3 Annotated Spectrum Chart - Additional Chart Controls

Click on the dropdown to show annotation on Glycan ions, Peptide ions, or both.

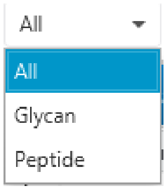

Click on 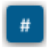 or 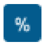 to toggle between absolute and percentage intensity values in the Y axis.

Click on 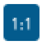 to reset the zoom level of the annotated spectrum chart to the default.

Click on 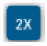 to zoom into the X axis by a factor of 2.

Click on 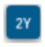 to zoom into the Y axis by a factor of 2.

The "alignment" check box 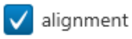 allows user to display/hide how the fragment ions generated from the peptide align with the spectrum. N-terminal ions are shown in blue and C-terminal ions are shown in red.

The “error map” check box 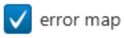 is used to show/hide the error map underneath the chart.

11.5.6 Glycan Ion Match Table

The Glycan Ion Match Table shows the theoretical m/z and observed m/z for each moiety of glycan ions matched in the spectrum. These columns are sortable and can be rearranged.

Glycan Ion Match | Ion Match | Survey

#	Theoretical m/z	Observed m/z	Moiety
1	163.060	163.060	10099_Hex1
2	204.087	204.087	10099_HexNAc1
3	366.140	366.139	10099_HexNAc1Hex1
4	528.192	528.192	10099_HexNAc1Hex2
5	690.245	690.247	10099_HexNAc1Hex3
6	690.245	690.247	10099_HexNAc1Hex3
7	731.272	731.272	10099_HexNAc2Hex2
8	1558.714	1558.710	b12_10099_HexNAc1
9	1879.795	1879.806	y14_10099_HexNAc1
10	1936.817	1936.804	y15_10099_HexNAc1
[illegible]	[illegible]	[illegible]	[illegible]

11.5.7 Ion Match Table

The “Ion Match” tab in the bottom pane contains a table with possible fragment ions for the selected MS/MS scan. Each ion in the table shows the calculated mass, where if the fragment ion is present in the spectrum, its mass value is displayed in color. N-terminal ions are shown in blue and C-terminal ions are shown in red. A fragment ion is found when there is a matching peak within the mass error tolerance, as defined in the de novo sequencing parameters, and when relative intensity of the matching peak is at least 2%. The ion types displayed in the table can be configured in "Spectrum Annotation Settings" (see 11.5.5.2 Annotated Spectrum Chart - Spectrum Annotation Settings)

Glycan Ion Match | **Ion Match** | Survey

#	b	b-H2O	b-NH3	b(2+)	Seq	y	y-H2O	y-NH3	y(2+)	#
1	129.066	111.056	112.039	65.033	Q					21
2	257.125	239.114	240.098	129.062	Q	2387.065	2369.055	2370.039	1194.033	20
3	385.182	367.170	368.157	193.092	Q	2259.007	2240.996	2241.980	1130.004	19
4	522.241	504.232	505.214	261.621	H	2130.948	2112.938	2113.921	1065.988	18
5	635.326	617.317	618.299	318.163	L	1993.890	1975.879	1976.862	997.445	17
6	782.396	764.382	765.370	391.697	F	1880.805	1862.795	1863.778	940.903	16
7	839.416	821.395	822.389	420.208	G	1733.737	1715.726	1716.710	867.369	15
8	926.448	908.455	909.421	463.724	S	1676.716	1658.705	1659.689	838.858	14
9	1040.491	1022.481	1023.464	520.746	N(+1622.58)	1589.683	1571.673	1572.656	795.342	13
10	1139.560	1121.549	1122.516	570.280	V	1475.644	1457.630	1458.614	738.320	12
[illegible]	[illegible]	[illegible]	[illegible]	[illegible]	[illegible]	[illegible]	[illegible]	[illegible]	[illegible]	[illegible]

Note: Uncheck the alignment checkbox in the spectrum annotation view to see the highlighted fragment ions more easily.

11.5.8 Survey Chart

The “Survey” tab displays the precursor ion spectrum. The buttons that appear in this section are identical to those explained above in 11.5.5.3 Annotated Spectrum Chart - Additional Chart Controls

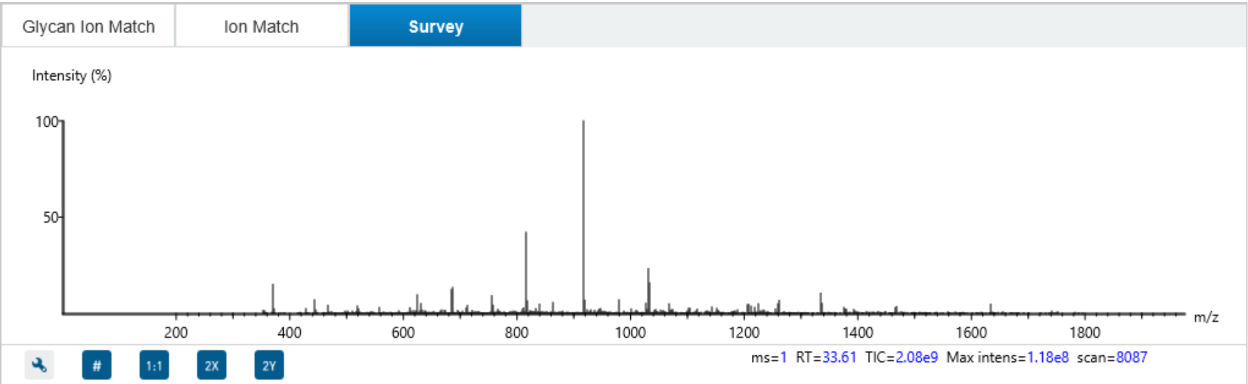

11.5.9 LC/MS Snapshot

The LC-MS Snapshot displays a zoomed-in view of the precursor scan in the LC-MS heat map 2-D view. The glycan peptide feature is highlighted in purple and the selected PSM marker is yellow for O-linked and purple for N-linked. The span of the orange line in the LC-MS Snapshot designates the isolation window.

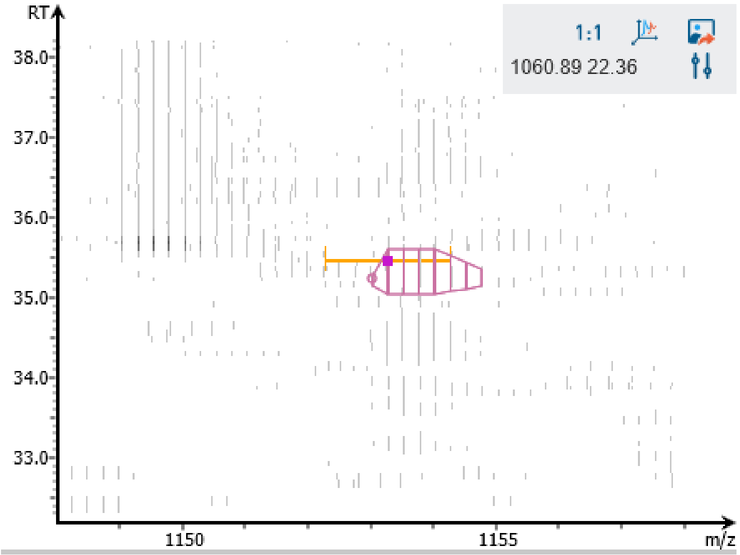

Users can change the view from the 2-D heat map to a 3-D intensity view by clicking the intensity view button 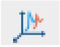 in the upper right-hand corner of the LC-MS Snapshot. In addition, users can export the LC-MS heat map by clicking the export image button 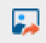 in the upper right-hand corner of the LC-MS Snapshot.

Expand the options dropdown by clicking 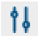 to reveal a search option to specify a *m/z RT* (ensure there is a space between these two numbers) location to zoom into. There is also a Peak slider filter to filter out low intensity peaks and a feature area filter to filter out low area features.

11.5.10 Precursor Profile

The Precursor Profile, or eXtracted Ion Chromatogram (XIC) of the precursor ion, graphs the elution profile of the selected peptide feature vector over the retention time range where it was identified. The graph is plotted with

the retention time along the x-axis and the intensity along the y-axis.

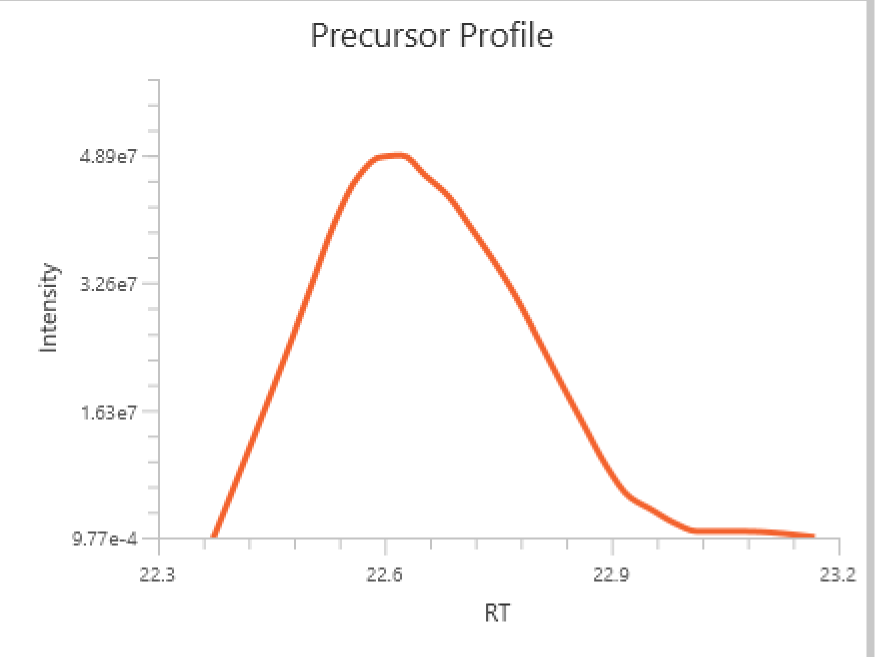

11.6 Glycan Search Peptide View

The "Peptide View" is very similar to the Glycopeptide View and it displays the resulting non-glycopeptide identifications. The table on the top shows the details of each peptide identification. The bottom section provides additional information about the peptide-spectrum matches for the native peptide selected in the table.

Note: If you are already familiar with the Glycopeptide Tab Section 11.5, you can skip this section as the interface is largely the same.

11.6.1 Peptide Table

The Peptide Table shows the filtered native peptide identification results. Each row in the table is a native peptide identification represented by its highest-scoring PSM. When there are more than 1000 peptides, the table is split into multiple pages.

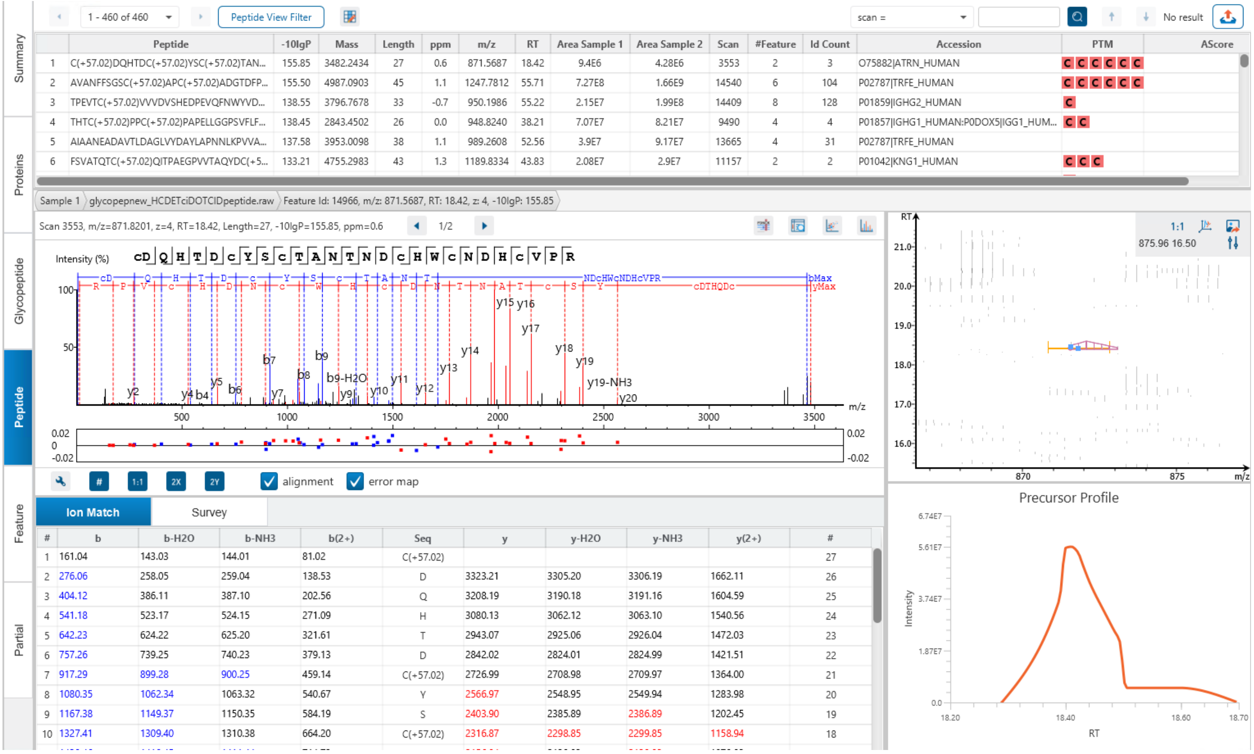

Note: If there are multiple PSMs matched to a peptide, the table displays the information for the top-scoring PSM. Peptides with the same sequence but different modifications are considered different and shown in different rows.

To copy the sequence, right click on a row (or multiple rows) in the table and select “Copy selected peptide sequence(s)”. Another option is to mouse over any cell in the table and hitting Ctrl + C on your keyboard to copy the contents of those selected cells.

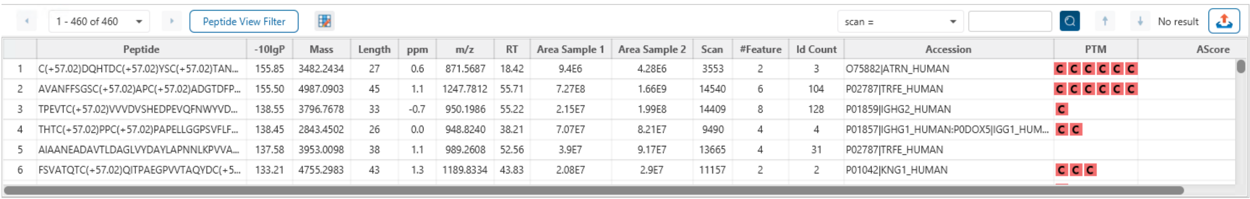

The following list describes the contents in each of the default columns:

- **Peptide:** The amino acid sequence of the peptide, as determined by the Glycan Search workflow. A modified residue is followed by a pair of parentheses enclosing the modification.
- **-10lgP:** The peptide -10lgP score. The score indicates the scoring significance of a peptide-precursor spectrum match.
- **Mass:** The monoisotopic mass of the peptide.
- **Length:** The number of amino acids in the sequence backbone.
- **ppm:** The precursor mass error, calculated as $10^6 \times$ (precursor mass - peptide mass) / peptide mass.
- **m/z:** The precursor mass-to-charge ratio.
- **RT:** The retention time (elution time) of the spectrum as recorded in the data.

- **Area per Sample:** The combined area for all features in that sample associated with this peptide.
- **Sample Profile:** A column only shown for Glycan Search results with two or more samples. Shows a profile view of the ratio between areas in each sample.
- **Group Profile:** A column only shown for Glycan Search results with two or more samples. Shows a profile view of the ratio between areas in each group.
- **#Vector:** A column only shown for Glycan Search results with two or more samples. Counts the number of feature vectors associated with this peptide.
- **Scan:** The scan number of the spectrum that matches the peptide sequence with the highest -10lgP.
- **#Feature:** For single sample analysis, shows number of features assigned to the peptide sequence from identification. For multiple samples, if peptide has feature vectors, it will show number of features used (area > 0) in all feature vectors. If not, it shows the number of features from identification.
- **Id Count:** Number of identifications in total.
- **Accession:** The accessions of proteins that contain this native peptide.
- **PTM:** The types and the numbers of modifications present in the peptide shown in color-coded icons.
- **AScore:** Localization score assigned to modifications on the peptide. It is the -10 log of a p-value. In this case, the p-value is the probability that the modification occurs at the reported position compared to other possible positions. A -10lgP of 20 is equal to a p-value of 0.01. Scores are listed in the format AminoAcid, peptide position, modification, AScore. For example, serine at amino acid 3 is phosphorylated with an AScore of 54.01 will be reported as: S3 P 54.01.

11.6.2 Peptide Table controls

The menu above the peptide table provides options to scroll through the table of native peptides, filter the table, show optional columns and search for specific entries.

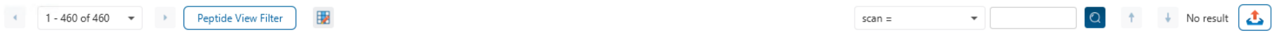

11.6.2.1 Peptide Table controls - Pagination

For easy viewing and to improve performance, the peptide table is limited to showing 1000 entries at once. Pagination is used to manage which set of 1000 entries will be viewed.

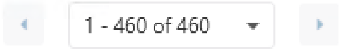

In the top left, there is a dropdown to select the rows for viewing. Click the left arrow or right arrow to select the previous/next 1000 rows. If the table is sorted or filtered, it may update the pagination set of rows that is currently in view.

11.6.2.2 Glycan Table controls - View Filter

Click on the 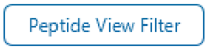

button to open the Peptide View Filter which is used to filter the Peptide table. The following are the filter options provided:

- Peptide sequence contains: Enter an amino acid sequence, only peptides that contain that sequence will be displayed in the peptide table. Adding PTMs in brackets in the sequence search is not handled, meaning only the sequence backbone is considered in the filter. Use the PTM contains for further filtering if necessary.

- Peptide sample area: Filters based on total peptide area found in each sample. Sample areas are calculated using the total of all peptide features from all spectra that identified the native peptide. If the peptide is found in multiple samples, only one sample must pass this filter for the peptide to be included.

- PTM contains: Type the names of PTMs you would like to include. Peptides containing PTMs that match the text will be included. If multiple PTMs match the text, they will all be included in the table.

Note: In all filters, multiple entries can be separated by a semi-colon.

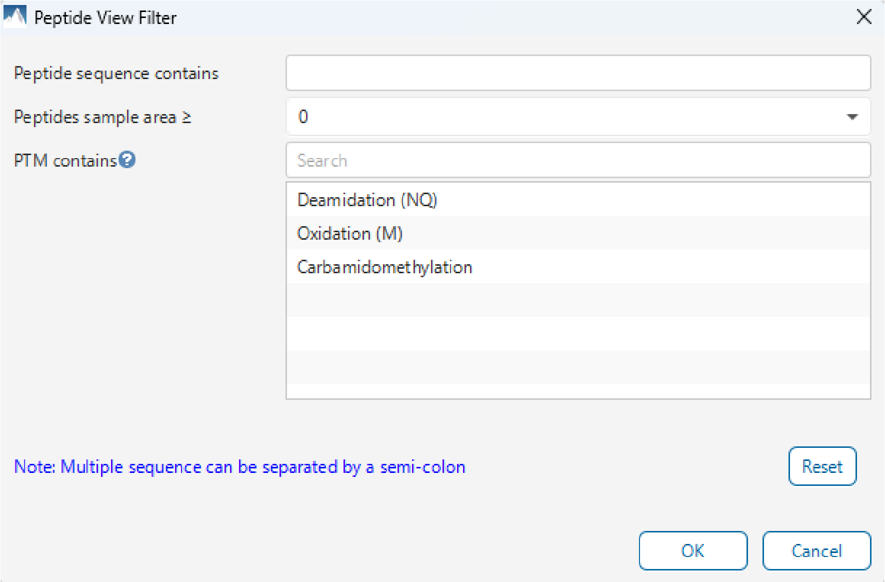

Click on the Reset button to reset all filters to their default setting.

Click on OK to apply the filters to the Peptide table.

Click on Cancel to discard the changes in the View Filter.

11.6.2.3 Peptide Table controls - Optional Columns

Click on the 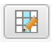 button to open the optional columns dropdown. By default the sample area columns are displayed. Sample Feature count columns can also be shown. For projects with many samples, it might be useful to hide these optional columns and reduce the width of the peptide table.

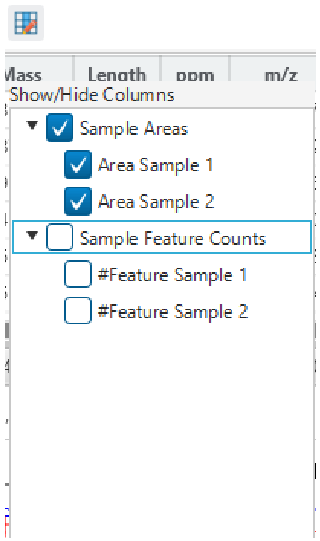

11.6.2.4 Glycan Table controls - Search function

On the top right of menu, there is a search function with 4 options. Search results can be navigated between using the Up or Down arrows.

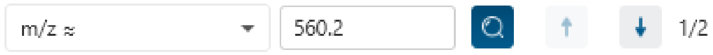

Options for searching include:

- **Scan =:** Search for the Scan number associated with each peptide. One scan can be associated to multiple peptides. For projects with multiple data files, the scan number is searched on every fraction.
- **m/z ≈:** Search for the approximate m/z. The search will consider a range of m/z equal to the search value +/- 1 of the last significant digit. For example, searching for m/z ≈ 560.21 will find all m/z between [560.20, 560.22]
- **RT ≈:** Search for the approximate RT. Likewise, the search will consider a range of RT equal to the search value +/- 1 of the last significant digit set. For example, searching for RT ≈ 55.5 will find all m/z between [55.4, 55.6]
- **Seq contains:** Search for the sequence in the table. If specifying a sequence without PTMs, the results will include that sequence and any sequences with PTMs with the same backbone. If specifying a sequence with bracket and some mass value (like in the above image), then only sequences that have a substring with an exact match will be considered.
  Additionally, since the peptide table shows both I/L and they can not be distinguished, then searching for I in the sequence is equivalent to searching for L in the sequence. i.e. searching for "LVLK" is the same as searching for "LVIK", "IVLK", and "IVIK"

11.6.3 Feature selection breadcrumb

There is a sample and feature selection breadcrumb which allows you to switch between features associated with the peptide. Click on the Sample in the breadcrumb to reveal other samples and the features in that sample related to the current peptide selection. From here you can select other samples, fractions, or features.

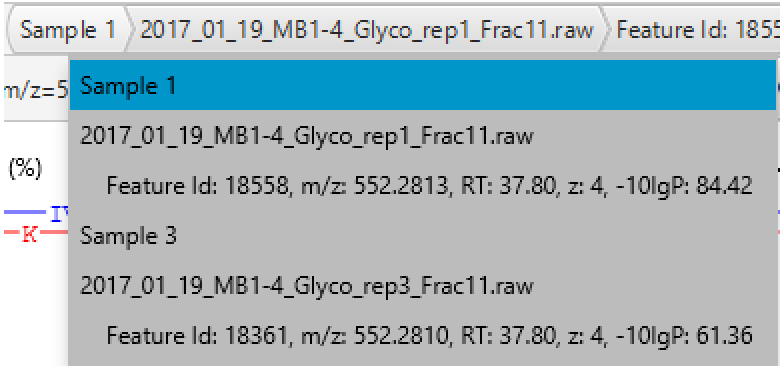

By clicking on the fraction level, all fractions in this sample are revealed as well as the features in each fraction associated with this peptide. From here you can select other fractions and features to view.

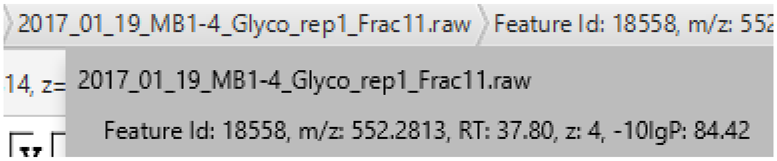

Lastly, clicking on the feature level will show all features in the currently selected fraction. Features with Feature Id = 0 are known as fake features and are constructed to enhance identification.

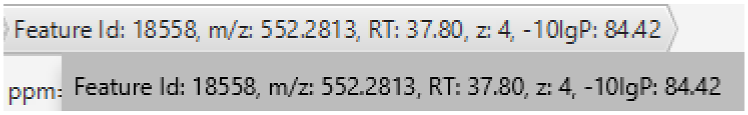

11.6.4 PSM selection

Underneath the feature breadcrumb will shown the currently selected PSM. Click on the Left and Right arrow buttons to switch between PSMs related to the currently selected feature.

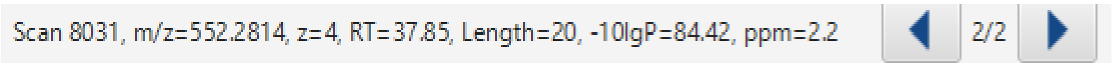

All other components in the Peptide tab interface will update according to the currently selected PSM.

For timsTOF instruments, Precursor ID is used rather than Scan number.

11.6.4.1 Protein jump button

Click on 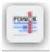 to jump to a protein that contains the currently selected PSM. If there are more than 1 protein, a dropdown is revealed which allows the user to select which protein to jump to. If a protein is filtered out due to a protein summary filter or summary page protein filter, then the option to jump will be grayed out.

11.6.4.2 Show spectrum in Data View

Click on to open the Data Refine MS/MS tab (if not already opened) and jump to the corresponding selected Scan.

11.6.4.3 Show spectrum in LC/MS View

Click on to open the Data Refine LC/MS tab (if not already opened) and jump to the corresponding selected Scan, highlighting the Glycopeptide MS/MS marker and the feature if present.

11.6.4.4 Show Raw Spectrum View

Click on to show the raw spectrum chart of the currently selected Scan.

11.6.5 Annotated Spectrum Chart

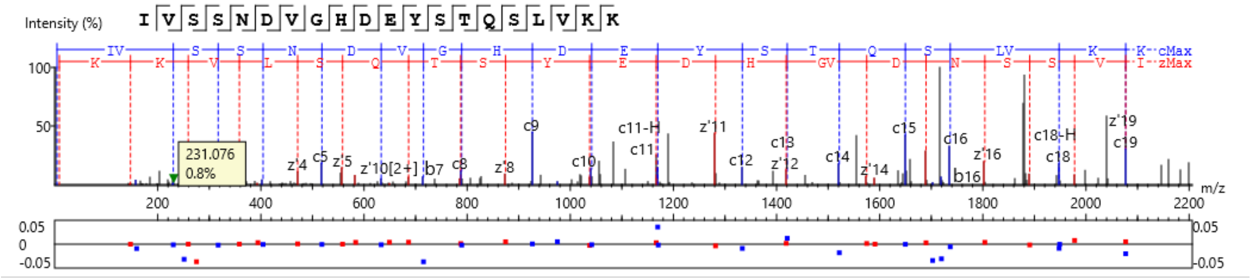

For the currently selected PSM, a graphical representation is shown in the form of an annotated spectrum chart. There are several controls provided for this view which will be explored in the following sections.

11.6.5.1 Annotated Spectrum Chart - Chart Navigation

Moving the cursor over the peptide sequence in the spectrum will show the mass transitions for a particular amino acid residue.

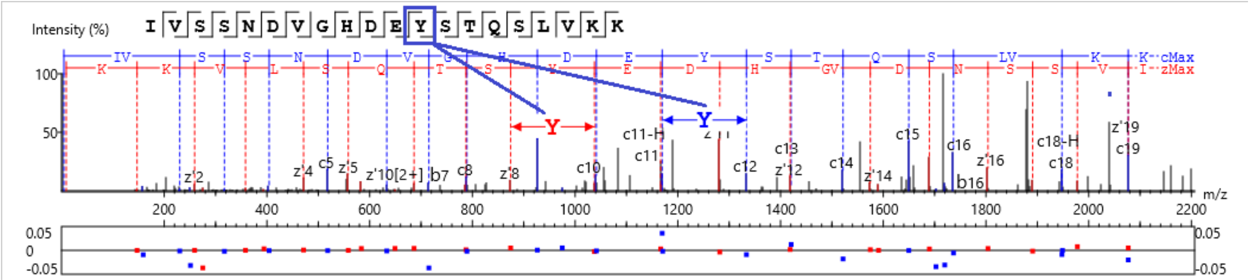

Zoom to a m/z region: Drag horizontally from the start m/z and to the end m/z with left mouse button.

Zoom in/out smoothly: Place the cursor at a particular m/z value (right below the x-axis), zoom in/out by scrolling the mouse wheel.

Increase/Decrease peak intensity: Place the cursor in the spectrum and scroll the mouse wheel.

See the whole spectrum: Double click in the spectrum or click the 1:1 button.

11.6.5.2 Annotated Spectrum Chart - Spectrum Annotation Settings

Click on the 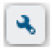 button to show the spectrum annotation settings.

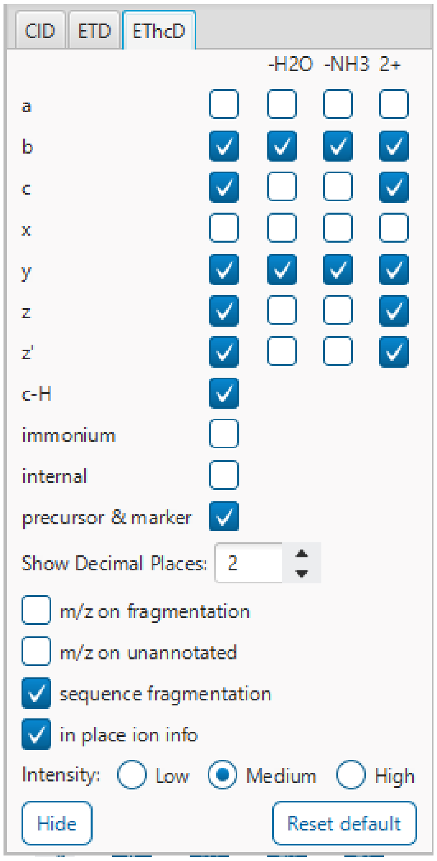

The spectra in the PEAKS results can be annotated by selecting ion types from the thorough collection of ions that PEAKS offers. The selected ion types will be displayed in the “Ion Match” table, as well. It is possible to annotate the spectrum with various ions for CID, EThcD and ETD. Each of these fragmentation modes can be set with different settings which will be shared with other results with the same fragmentation type.

To reset the defaults, click on the “Reset default” button.

Show Decimal Places: Select the number of decimal places that will appear in the ion table and the spectrum view. The default is set to two decimal places.

m/z on Fragmentation: Select this to display the m/z value on top of the annotated ions.

m/z on Unannotated: Select this to display the m/z value on top of the peaks without ion matches.

Sequence Fragmentation: Select this to display the sequence fragmentation on the top-left corner of the “Spectrum Annotation” view.

In Place Ion Info: Ion information, m/z value, and relative intensity are all displayed in a pop-up within the "Spectrum Annotation" view when this option is checked and the cursor is placed on a peak.

Intensity: Set the intensity threshold for spectrum annotation to low (2%), medium (5%), or high (10%).

11.6.5.3 Annotated Spectrum Chart - Additional Chart Controls

Click on # or % to toggle between absolute and percentage intensity values in the Y axis.

Click on 1:1 to reset the zoom level of the annotated spectrum chart to the default.

Click on 2X to zoom into the X axis by a factor of 2.

Click on 2Y to zoom into the Y axis by a factor of 2.

The "alignment" check box allows user to display/hide how the fragment ions generated from the peptide align with the spectrum. N-terminal ions are shown in blue and C-terminal ions are shown in red.

The "error map" check box is used to show/hide the error map underneath the chart.

11.6.6 Ion Match Table

The "Ion Match" tab in the bottom pane contains a table with possible fragment ions for the selected MS/MS scan. Each ion in the table shows the calculated mass, where if the fragment ion is present in the spectrum, its mass value is displayed in color. N-terminal ions are shown in blue and C-terminal ions are shown in red. A fragment ion is found when there is a matching peak within the mass error tolerance, as defined in the de novo sequencing parameters, and when relative intensity of the matching peak is at least 2%. The ion types displayed in the table can be configured in "Spectrum Annotation Settings".

Ion Match | Survey

#	b	b-H2O	b-NH3	b(2+)	Seq	y	y-H2O	y-NH3	y(2+)	#
10	1079.503	1061.488	1062.498	540.248	E	1596.643	1578.636	1579.610	798.818	13
11	1265.576	1247.569	1248.557	633.292	W	1467.601	1449.588	1450.585	734.297	12
12	1394.626	1376.616	1377.600	697.813	E	1281.521	1263.512	1264.488	641.266	11
13	1481.659	1463.648	1464.632	741.329	S	1152.480	1134.469	1135.461	576.736	10
14	1596.682	1578.671	1579.655	798.841	N(+0.98)	1065.449	1047.440	1048.427	533.228	9
15	1653.703	1635.693	1636.676	827.352	G	950.422	932.407	933.390	475.709	8
16	1781.773	1763.751	1764.735	891.381	Q	893.397	875.388	876.370	447.198	7
17	1878.815	1860.804	1861.788	939.907	P	765.341	747.326	748.315	383.169	6
18	2007.857	1989.847	1990.830	1004.429	E	668.288	650.274	651.258	334.642	5
19	2122.880	2104.870	2105.853	1061.940	N(+0.98)	539.245	521.231	522.222	270.124	4
[illegible]	[illegible]	[illegible]	[illegible]	[illegible]	[illegible]	[illegible]	[illegible]	[illegible]	[illegible]	[illegible]

Note: Uncheck the alignment checkbox in the spectrum annotation view to see the highlighted fragment ions more easily.

11.6.7 Survey Chart

The "Survey" tab displays the precursor ion spectrum. The buttons that appear in this section are identical to those explained above in Section 11.6.5.3 Annotated Spectrum Chart - Additional Chart Controls.

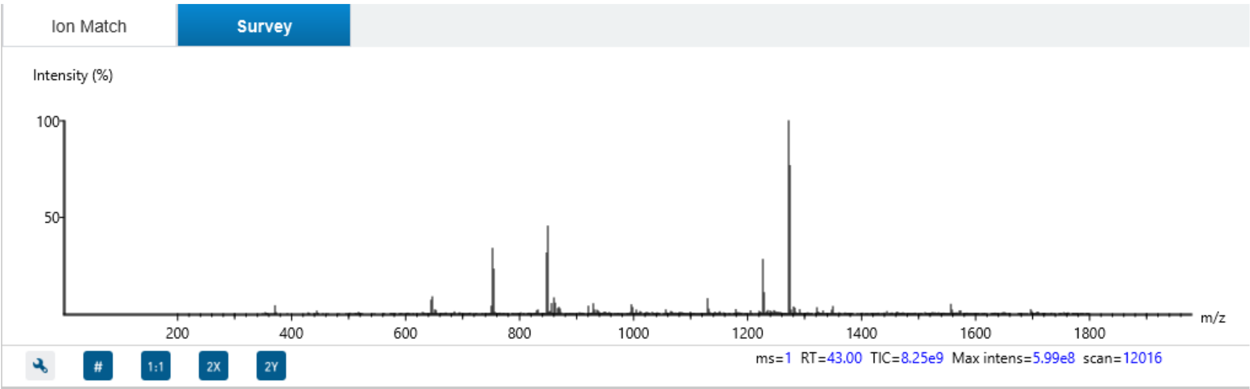

11.6.8 LC/MS Snapshot

The LC-MS Snapshot displays a zoomed-in view of the precursor scan in the LC-MS heat map 2-D view. The peptide feature is highlighted in purple and the selected PSM marker is blue. The span of the orange line in the LC-MS Snapshot designates the isolation window.

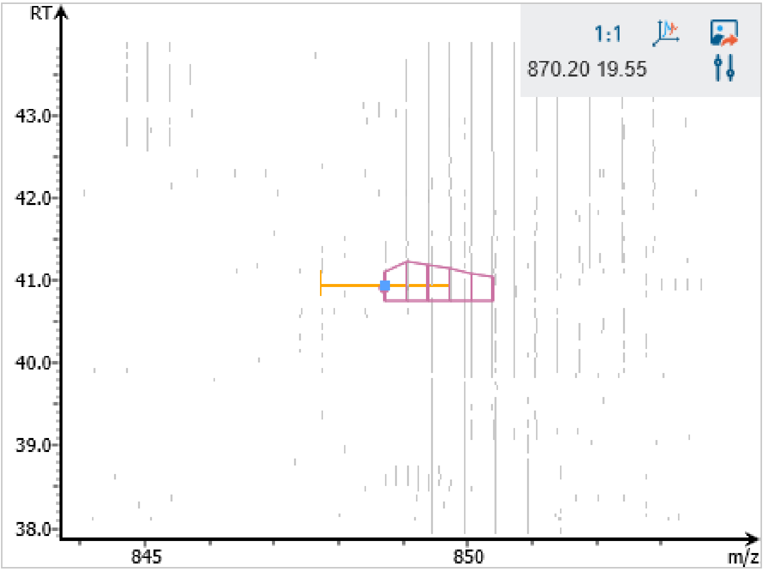

Users can change the view from the 2-D heat map to a 3-D intensity view by clicking the intensity view button in the upper right-hand corner of the LC-MS Snapshot. In addition, users can export the LC-MS heat map by clicking the export image button in the upper right-hand corner of the LC-MS Snapshot.

Expand the options dropdown by clicking to reveal a search option to specify a m/z RT (ensure there is a space between these two numbers) location to zoom into. There is also a Peak slider filter to filter out low intensity peaks and a feature area filter to filter out low area features.

11.6.9 Precursor Profile

The Precursor Profile, or eXtracted Ion Chromatogram (XIC) of the precursor ion, graphs the elution profile of the selected peptide feature vector over the retention time range where it was identified. The graph is plotted with the retention time along the x-axis and the intensity along the y-axis.

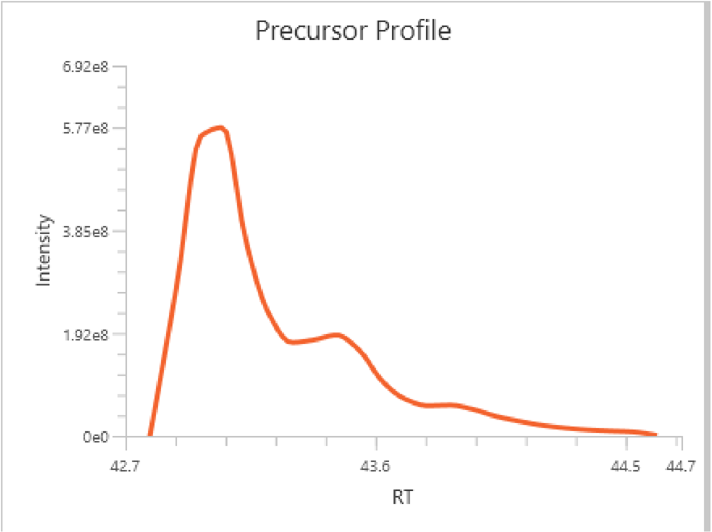

11.7 Glycan Search Feature View

The Glycan Search Feature View shows all real features associated with peptides.

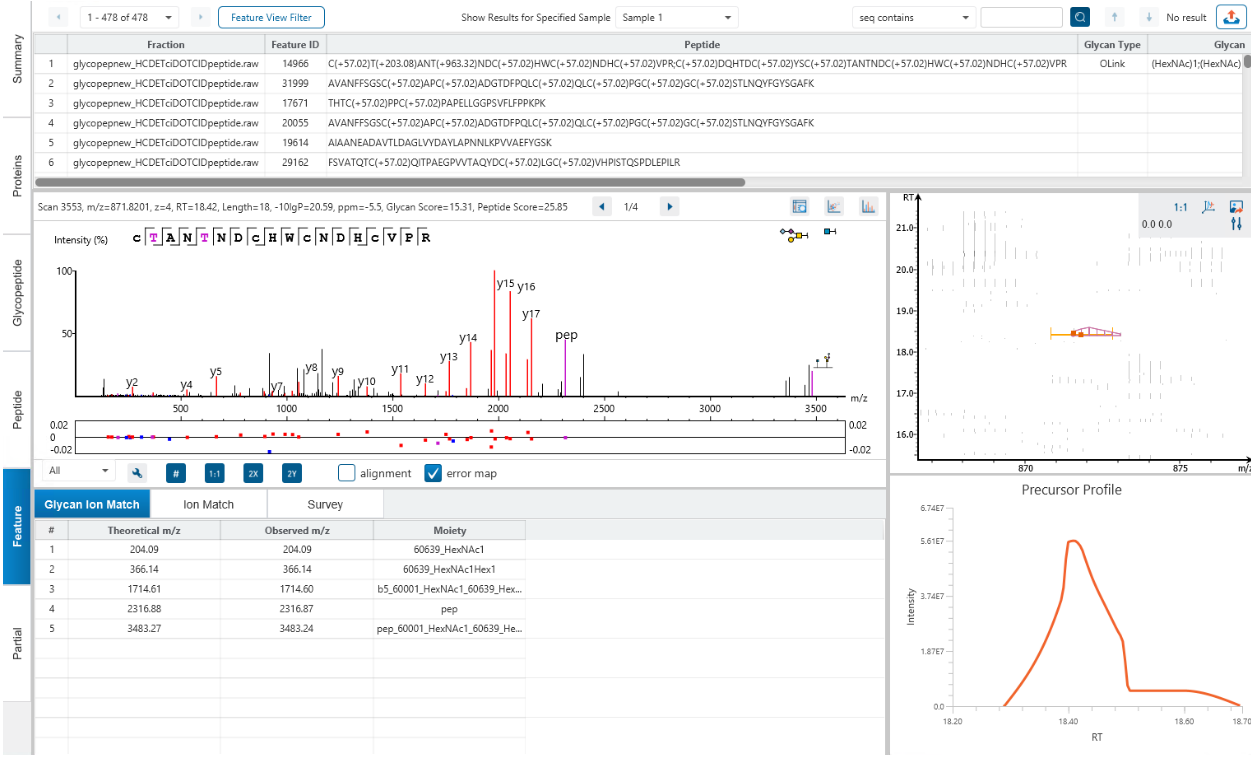

11.7.1 Feature Table

The Feature Table consists of the following columns.

- **Fraction:** The data file from which this feature is detected.
- **Feature ID:** A unique identifier of the feature from the data file.
- **Peptide:** The peptide sequence including PTMs and Glycans
- **Glycan Type:** For glycan peptides, classifies the glycan as an N-link or O-link glycan.
- **Glycan:** Contains the glycan moiety information for the glycan peptide if applicable.
- **m/z:** Each feature in the feature vector has its own precursor mass-to-charge ratio. This column displays the average precursor mass-to-charge ratio of all of the features in the feature vector.
- **z:** The precursor charge of the feature or feature vector.
- **RT:** Simple the middle point of the retention time range of the feature or features in the feature vector. It is equal to (RT Begin + RT End) / 2
- **RT Begin:** The lower bound of the retention time range of the feature or features in the feature vectors.
- **RT End:** The higher bound of the retention time range of the feature or features in the feature vectors.
- **1/k0:** Column shown for timsTOF instruments only, measuring the 1/k0 ion mobility range for the feature or feature vector.
- **Area:** Each feature in the feature vector has its own intensity area under the XIC curve. This column displays the average area of all the features in the feature vector.
- **ppm:** The mass error of a feature. For multiple samples, the mass error of the feature vector is calculated from the mass error of each feature in that vector.
- **Id Count:** The total number of identifications found for the peptide in fractions.
- **PTM:** Any PTMs in the peptide are shown here. Mouse over for a description of the PTM.
- **Accession:** A list of proteins that contain this peptide.

11.7.2 Feature Table controls

The menu above the feature table provides options to scroll through the table of features or feature vectors, filter the table, show optional columns and search for specific entries.

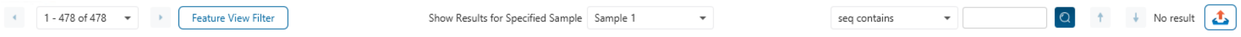

11.7.2.1 Feature Table controls - Pagination

For easy viewing and to improve performance, the glycan table is limited to showing 1000 entries at once. Pagination is used to manage which set of 1000 entries will be viewed.

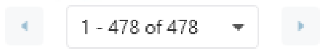

In the top left, there is a dropdown to select the rows for viewing. Click the left arrow or right arrow to select the previous/next 1000 rows. If the table is sorted or filtered, it may update the pagination set of rows that is currently in view.

11.7.2.2 Feature Table controls - Sample selection

The feature table only shows results from one sample at a time. Use the dropdown to switch between samples and update the Feature table accordingly.

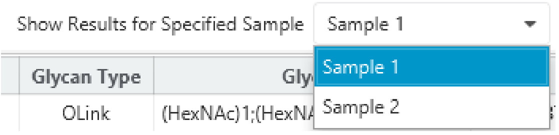

11.7.2.3 Feature Table controls - Search function

On the top right of menu, there is a search function with 2 options. Search results can be navigated between using the Up or Down arrows.

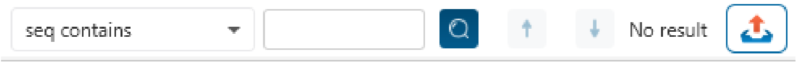

Options for searching include:

- **m/z ≈:** Search for the approximate m/z. The search will consider a range of m/z equal to the search value +/- 1 of the last significant digit.

For example, searching for m/z ≈ 650 will find all m/z between [649, 651]

- **Seq contains:** Search for the sequence in the table. If specifying a sequence without glycans or PTMs, the results will include that sequence and any sequences with glycans or PTMs with the same backbone. If specifying a sequence with bracket and some mass value (like in the above image), then only sequence that have a substring with an exact match will be considered.

Additionally, since the glycan peptide table shows both I/L and they can not be distinguished, then searching for I in the sequence is equivalent to searching for L in the sequence. i.e. Searching for “LVLK” is the same as searching for “LVIK”, “IVLK”, and “IVIK

11.7.3 Feature Details

The Feature Details tab contains the eXtracted Ion Chromatogram (XIC) chart that displays the shape of the selected peptide feature vector over the retention time range where they were identified. The table at the top-left corner displays both the area and the ratio of the features in the feature vector. This table also works as a control to select the features that will be used to draw the XIC plot and other plots in the “Sample Features” and “RT Alignment” tabs.

11.7.4 PSM selection

Underneath the feature breadcrumb will shown the currently selected PSM. Click on the Left and Right arrow buttons to switch between PSMs related to the currently selected feature.

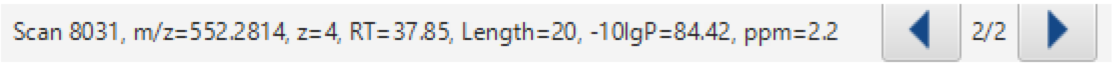

All other components in the Peptide tab interface will update according to the currently selected PSM.

For timsTOF instruments, Precursor ID is used rather than Scan number.

11.7.4.1 Show spectrum in Data View

Click on to open the Data Refine MS/MS tab (if not already opened) and jump to the corresponding selected Scan.

11.7.4.2 Show spectrum in LC/MS View

Click on to open the Data Refine LC/MS tab (if not already opened) and jump to the corresponding selected Scan, highlighting the Glycopeptide MS/MS marker and the feature if present.

11.7.4.3 Show Raw Spectrum View

Click on to show the raw spectrum chart of the currently selected Scan.

11.7.5 Annotated Spectrum Chart

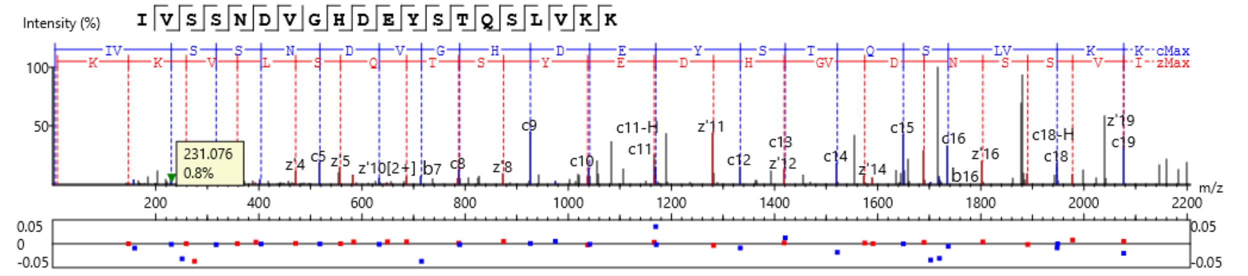

For the currently selected PSM, a graphical representation is shown in the form of an annotated spectrum chart. There are several controls provided for this view which will be explored in the following sections.

11.7.5.1 Annotated Spectrum Chart - Chart Navigation

Moving the cursor over the peptide sequence in the spectrum will show the mass transitions for a particular amino acid residue.

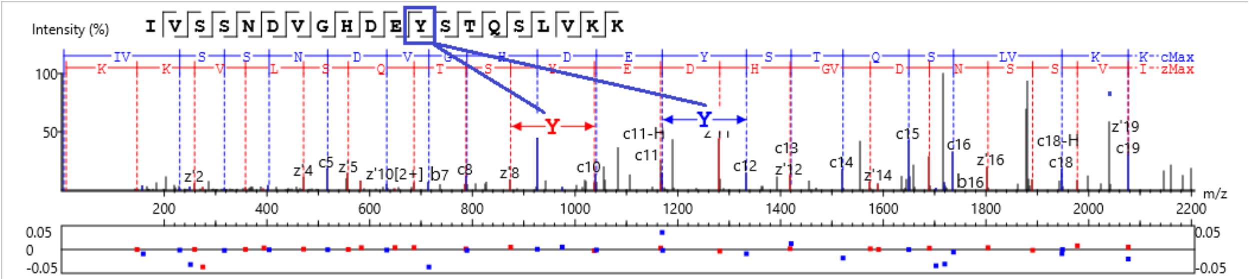

Zoom to a m/z region: Drag horizontally from the start m/z and to the end m/z with left mouse button.

Zoom in/out smoothly: Place the cursor at a particular m/z value (right below the x-axis), zoom in/out by scrolling the mouse wheel.

Increase/Decrease peak intensity: Place the cursor in the spectrum and scroll the mouse wheel.

See the whole spectrum: Double click in the spectrum or click the 1:1 button.

11.7.5.2 Annotated Spectrum Chart - Spectrum Annotation Settings

Click on the button to show the spectrum annotation settings.

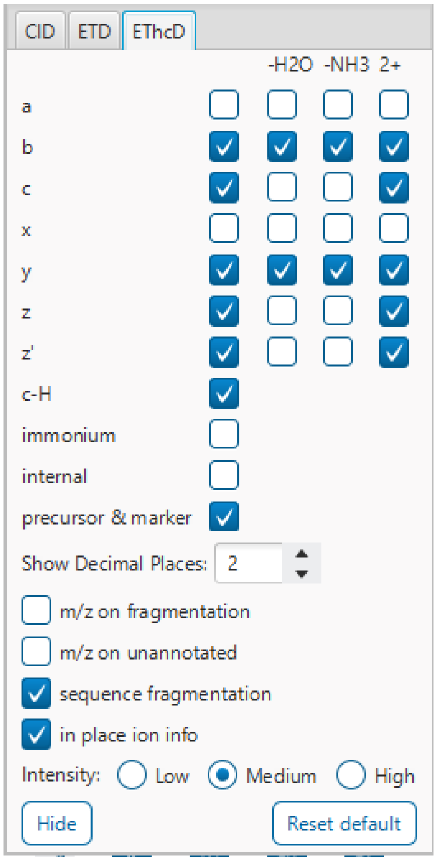

The spectra in the PEAKS results can be annotated by selecting ion types from the thorough collection of ions that PEAKS offers. The selected ion types will be displayed in the “Ion Match” table, as well. It is possible to annotate

the spectrum with various ions for CID, EThcD and ETD. Each of these fragmentation modes can be set with different settings which will be shared with other results with the same fragmentation type.

To reset the defaults, click on the “Reset default” button.

Show Decimal Places: Select the number of decimal places that will appear in the ion table and the spectrum view. The default is set to two decimal places.

m/z on Fragmentation: Select this to display the m/z value on top of the annotated ions.

m/z on Unannotated: Select this to display the m/z value on top of the peaks without ion matches.

Sequence Fragmentation: Select this to display the sequence fragmentation on the top-left corner of the “Spectrum Annotation” view.

In Place Ion Info: Ion information, m/z value, and relative intensity are all displayed in a pop-up within the “Spectrum Annotation” view when this option is checked and the cursor is placed on a peak.

Intensity: Set the intensity threshold for spectrum annotation to low (2%), medium (5%), or high (10%).

11.7.5.3 Annotated Spectrum Chart - Additional Chart Controls

Click on 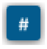

or 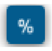

to toggle between absolute and percentage intensity values in the Y axis.

Click on 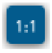

to reset the zoom level of the annotated spectrum chart to the default.

Click on 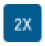

to zoom into the X axis by a factor of 2.

Click on 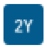

to zoom into the Y axis by a factor of 2.

The "alignment" check box allows user to display/hide how the fragment ions generated from the peptide align with the spectrum. N-terminal ions are shown in blue and C-terminal ions are shown in red.

The “error map” check box is used to show/hide the error map underneath the chart.

11.7.6 Ion Match Table

The “Ion Match” tab in the bottom pane contains a table with possible fragment ions for the selected MS/MS scan. Each ion in the table shows the calculated mass, where if the fragment ion is present in the spectrum, its mass value is displayed in color. N-terminal ions are shown in blue and C-terminal ions are shown in red. A fragment ion is found when there is a matching peak within the mass error tolerance, as defined in the de novo sequencing parameters, and when relative intensity of the matching peak is at least 2%. The ion types displayed in the table can be configured in "Spectrum Annotation Settings”.

#	b	b-H2O	b-NH3	b(2+)	Seq	y	y-H2O	y-NH3	y(2+)	#
10	1079.503	1061.488	1062.498	540.248	E	1596.643	1578.636	1579.610	798.818	13
11	1265.576	1247.569	1248.557	633.292	W	1467.601	1449.588	1450.585	734.297	12
12	1394.626	1376.616	1377.600	697.813	E	1281.521	1263.512	1264.488	641.266	11
13	1481.659	1463.648	1464.632	741.329	S	1152.480	1134.469	1135.461	576.736	10
14	1596.682	1578.671	1579.655	798.841	N(+0.98)	1065.449	1047.440	1048.427	533.228	9
15	1653.703	1635.693	1636.676	827.352	G	950.422	932.407	933.390	475.709	8
16	1781.773	1763.751	1764.735	891.381	Q	893.397	875.388	876.370	447.198	7
17	1878.815	1860.804	1861.788	939.907	P	765.341	747.326	748.315	383.169	6
18	2007.857	1989.847	1990.830	1004.429	E	668.288	650.274	651.258	334.642	5
19	2122.880	2104.870	2105.853	1061.940	N(+0.98)	539.245	521.231	522.222	270.124	4

Note: Uncheck the alignment checkbox in the spectrum annotation view to see the highlighted fragment ions more easily.

11.7.7 Survey Chart

The “Survey” tab displays the precursor ion spectrum. The buttons that appear in this section are identical to those explained above in Section 11.7.5.3 Annotated Spectrum Chart - Additional Chart Controls.

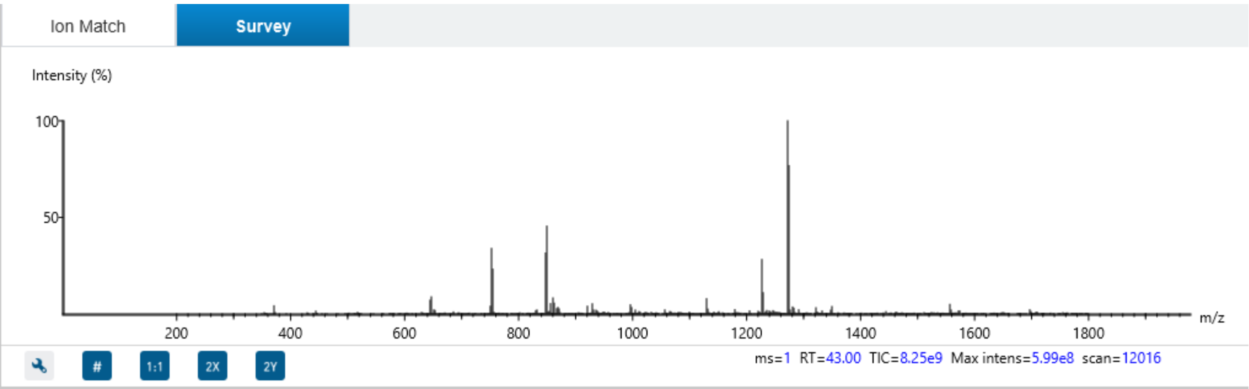

11.7.8 LC/MS Snapshot

The LC-MS Snapshot displays a zoomed-in view of the precursor scan in the LC-MS heat map 2-D view. The peptide feature is highlighted in purple and the selected PSM marker is blue. The span of the orange line in the LC-MS Snapshot designates the isolation window.

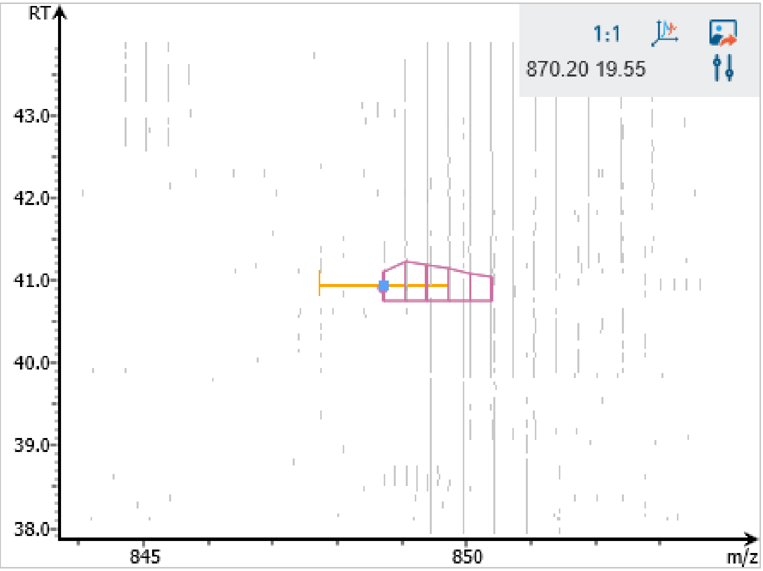

Users can change the view from the 2-D heat map to a 3-D intensity view by clicking the intensity view button in the upper right-hand corner of the LC-MS Snapshot. In addition, users can export the LC-MS heat map by clicking the export image button in the upper right-hand corner of the LC-MS Snapshot.

Expand the options dropdown by clicking to reveal a search option to specify a m/z RT (ensure there is a space between these two numbers) location to zoom into. There is also a Peak slider filter to filter out low intensity peaks and a feature area filter to filter out low area features.

11.7.9 Precursor Profile

The Precursor Profile, or eXtracted Ion Chromatogram (XIC) of the precursor ion, graphs the elution profile of the selected peptide feature vector over the retention time range where it was identified. The graph is plotted with the retention time along the x-axis and the intensity along the y-axis.

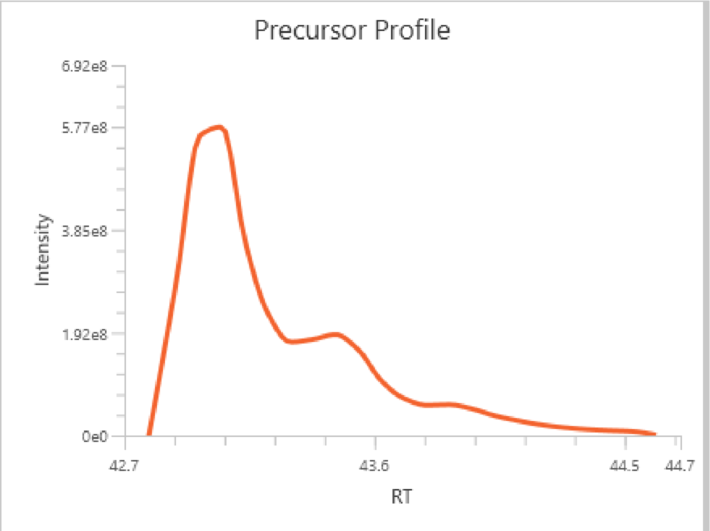

11.8 Glycan Search Partial View

The "Partial View" displays glycopeptides that either have no identified glycan or no identified peptide (if it has both it will show in the Glycan table). For those with no identified glycan, only the spectrum information is saved. For those with no identified peptide but with identified glycan, both spectrum information and glycan information are saved.

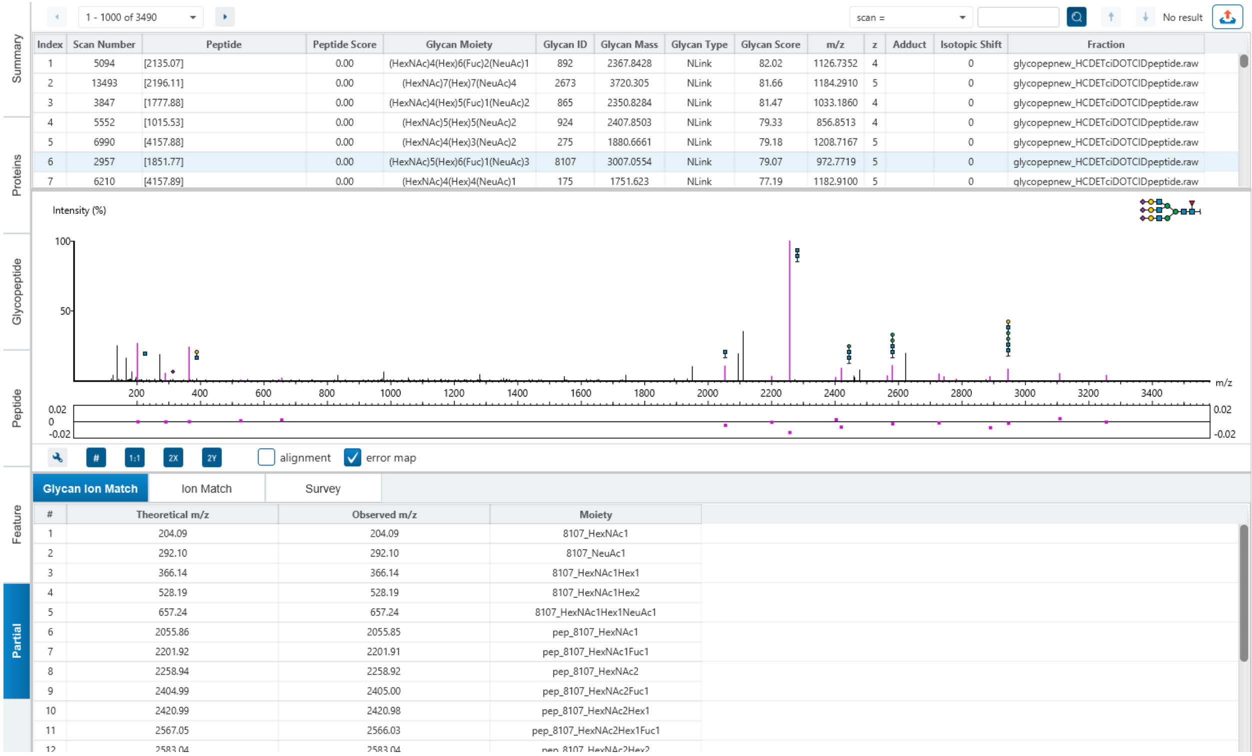

11.8.1 Partial Table

The Partial Table shows the filtered glycan peptide identification results. Each row in the table is a glycopeptide identification represented by its highest-scoring PSM. When there are more than 1000 peptides, the table is split into multiple pages.

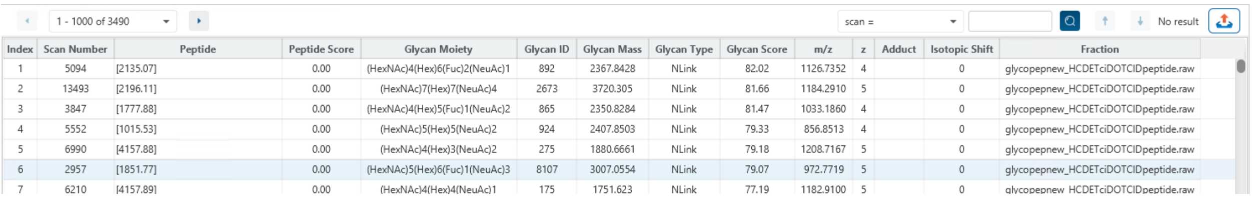

1 - 1000 of 3490   scan =   No result

Index	Scan Number	Peptide	Peptide Score	Glycan Moiety	Glycan ID	Glycan Mass	Glycan Type	Glycan Score	m/z	z	Adduct	Isotopic Shift	Fraction
1	5094	[2135.07]	0.00	(HexNAc)4(Hex)6(Fuc)2(NeuAc)1	892	2367.8428	NLink	82.02	1126.7352	4		0	glycopepnew_HCDETciDOTCIDpeptide.raw
2	13493	[2196.11]	0.00	(HexNAc)7(Hex)7(NeuAc)4	2673	3720.305	NLink	81.66	1184.2910	5		0	glycopepnew_HCDETciDOTCIDpeptide.raw
3	3847	[1777.88]	0.00	(HexNAc)4(Hex)5(Fuc)1(NeuAc)2	865	2350.8284	NLink	81.47	1033.1860	4		0	glycopepnew_HCDETciDOTCIDpeptide.raw
4	5552	[1015.53]	0.00	(HexNAc)5(Hex)5(NeuAc)2	924	2407.8503	NLink	79.33	856.8513	4		0	glycopepnew_HCDETciDOTCIDpeptide.raw
5	6990	[4157.88]	0.00	(HexNAc)4(Hex)3(NeuAc)2	275	1880.6661	NLink	79.18	1208.7167	5		0	glycopepnew_HCDETciDOTCIDpeptide.raw
6	2957	[1851.77]	0.00	(HexNAc)5(Hex)6(Fuc)1(NeuAc)3	8107	3007.0554	NLink	79.07	972.7719	5		0	glycopepnew_HCDETciDOTCIDpeptide.raw
7	6210	[4157.89]	0.00	(HexNAc)4(Hex)4(NeuAc)1	175	1751.623	NLink	77.19	1182.9100	5		0	glycopepnew_HCDETciDOTCIDpeptide.raw

To copy cell contents, mouse over any cell in the table and hitting Ctrl + C on your keyboard to copy the contents of those selected cells. This also works when selecting multiple rows and hitting Ctrl+C.

The following list describes the contents in each of the columns in the Unidentified table:

- **Index:** Simply the row count of the table. If the table is sorted, the Index remains the same order while the other columns sort.
- **Scan Number:** The scan number of the spectrum.

- **Peptide:** The peptide sequence or mass with partial identification.
- **Peptide Score:** The score assigned to identification of native peptide.
- **Glycan Moiety:** The best glycan moiety on this spectrum. For N-link results, there will always be 1. For O-link results, there could be more than 1.
- **Glycan ID:** The unique ID number we assign to that Glycan structure.
- **Glycan Mass:** The mass of the Glycan.
- **Glycan Type:** Identified whether the glycan is N-link or O-link.
- **Glycan Score:** The normalized sum of ions matched and unmatched. The score can be negative if the sum of unmatched ions intensity exceeds the matched ions.
- **m/z:** The precursor mass-to-charge ratio.
- **z:** The charge of this spectrum.
- **Adduct:** Indicates if an adduct is present and which adducts
- **Isotopic Shift:** Indicates if there is an isotopic shift
- **Fraction:** The fraction that this spectrum belongs to.

11.8.2 Partial Table controls

The menu above the peptide table provides options to scroll through the table of glycan peptides and search for specific entries.

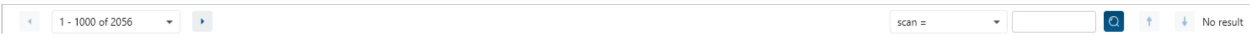

11.8.2.1 Partial Table controls - Pagination

For easy viewing and to improve performance, the glycan table is limited to showing 1000 entries at once. Pagination is used to manage which set of 1000 entries will be viewed.

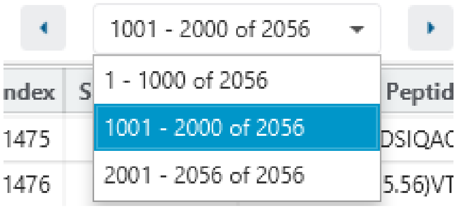

In the top left, there is a dropdown to select the rows for viewing. Click the left arrow or right arrow to select the previous/next 1000 rows. If the table is sorted or filtered, it may update the pagination set of rows that is currently in view.

11.8.2.2 Partial Table controls - Search function

On the top right of menu, there is a search function with 3 options. Search results can be navigated between using the Up or Down arrows.

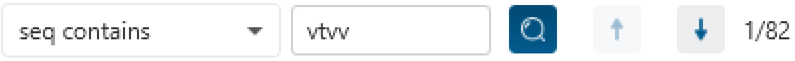

Options for searching include:

- **Scan = or Precursor Id =:** Search for the Scan number (or Precursor Id for timsTOF instruments) for the unidentified spectrum. For projects with multiple data files, the scan number is searched on every fraction.

- **m/z ≈:** Search for the approximate m/z. The search will consider a range of m/z equal to the search value +/- 1 of the last significant digit.

  For example, searching for m/z ≈ 800.80 will find all m/z between [800.79, 800.81]

- **Seq contains:** Search for the sequence in the table. If specifying a sequence without glycans or PTMs, the results will include that sequence and any sequences with glycans or PTMs with the same backbone. If specifying a sequence with bracket and some mass value, then only sequence that have a substring with an exact match will be considered.

11.8.3 Partial Spectrum Chart

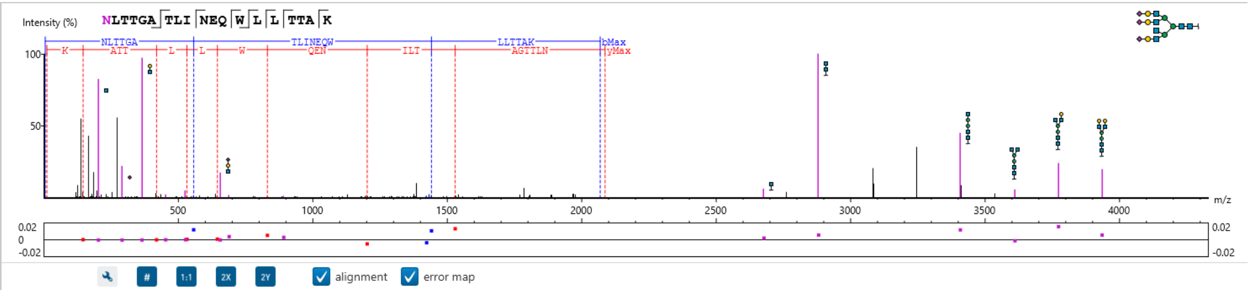

For the currently selected spectrum, a graphical representation is shown in the form of an annotated spectrum chart. Similar to the other annotated spectrum charts, there are several controls provided for this view which will be explored in the following sections. Since there are no peptide identification, several of the provided options that are shared have no use in this chart.

11.8.3.1 Partial Spectrum Chart - Chart Navigation

Zoom to a m/z region: Drag horizontally from the start m/z and to the end m/z with left mouse button.

Zoom in/out smoothly: Place the cursor at a particular m/z value (right below the x-axis), zoom in/out by scrolling the mouse wheel.

Increase/Decrease peak intensity: Place the cursor in the spectrum and scroll the mouse wheel.

See the whole spectrum: Double click in the spectrum or click the 1:1 button.

11.8.3.2 Partial Spectrum Chart - Spectrum Annotation Settings

Click on the 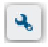 button to show the spectrum annotation settings.

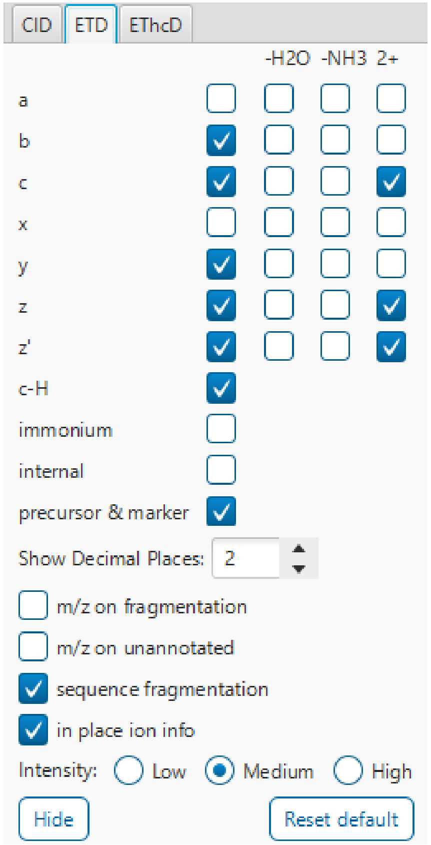

It is possible to annotate the spectrum with various ions for CID, EThcD and ETD. Each of these fragmentation modes can be set with different settings which will be shared with other results with the same fragmentation type.

To reset the defaults, click on the "Reset default" button.

Show Decimal Places: Select the number of decimal places that will appear in the ion table and the spectrum view. The default is set to two decimal places.

m/z on Fragmentation: This option has no use in this chart.

m/z on Unannotated: Select this to display the m/z value on top of the peaks without ion matches.

Sequence Fragmentation: This option has no use in this chart.

In Place Ion Info: Ion information, m/z value,and relative intensity are all displayed in a pop-up within the "Spectrum Annotation" view when this option is checked and the cursor is placed on a peak.

Intensity: Set the intensity threshold for spectrum annotation to low (2%), medium (5%), or high (10%).

11.8.3.3 Partial Spectrum Chart - Additional Chart Controls

Click on 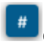 or 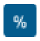 to toggle between absolute and percentage intensity values in the Y axis.

Click on 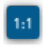 to reset the zoom level of the annotated spectrum chart to the default.

Click on 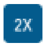 to zoom into the X axis by a factor of 2.

Click on 2Y to zoom into the Y axis by a factor of 2.

The "alignment" check box alignment is used to show the peptide alignment.

The “error map” check box error map is used to show/hide the error map underneath the chart.

11.8.4 Partial Glycan Ion Match Table

The Glycan Ion Match Table shows the theoretical m/z and observed m/z for each moiety of glycan ions matched in the spectrum. These columns are sortable and can be rearranged.

Glycan Ion Match | Ion Match | Survey

#	Theoretical m/z	Observed m/z	Moiety
1	163.060	163.060	75_Hex1
2	204.087	204.087	75_HexNAc1
3	325.113	325.114	75_Hex2
4	366.140	366.139	75_HexNAc1Hex1
5	528.192	528.192	75_HexNAc1Hex2
6	690.245	690.250	75_HexNAc1Hex3
7	1135.458	1135.460	b9_75_HexNAc1
8	1517.658	1517.659	b12_75_HexNAc1
9	1620.769	1620.750	y12_75_HexNAc1
10	1734.812	1734.815	y13_75_HexNAc1

11.8.5 Partial Ion Match Table

The “Ion Match” tab in the bottom pane contains a table with possible fragment ions for the selected MS/MS scan. Each ion in the table shows the calculated mass, where if the fragment ion is present in the spectrum, its mass value is displayed in color. N-terminal ions are shown in blue and C-terminal ions are shown in red. A fragment ion is found when there is a matching peak within the mass error tolerance, as defined in the de novo sequencing parameters, and when relative intensity of the matching peak is at least 2%. The ion types displayed in the table can be configured in "Spectrum Annotation Settings".

Glycan Ion Match | Ion Match | Survey

#	b	c	b-H2O	b-NH3	b(2+)	c(2+)	c-H	Seq	y	z	z'	y-H2O	y-NH3	y(2+)	z(2+)	z'(2+)	#
1	58.029	75.056	40.019	41.002	29.515	38.028	74.048	G									18
2	155.082	172.109	137.071	138.055	78.041	86.555	171.101	P	1943.928	1926.901	1927.909	1925.917	1926.901	972.464	963.951	964.454	17
3	270.109	287.136	252.098	253.082	135.554	144.066	286.128	D	1846.875	1829.848	1830.856	1828.865	1829.848	923.938	915.424	915.928	16
4	383.192	400.220	365.182	366.166	192.097	200.610	399.212	L	1731.848	1714.821	1715.829	1713.838	1714.821	866.424	857.911	858.415	15
5	470.227	487.252	452.214	453.198	235.613	244.126	486.244	S	1618.769	1601.737	1602.745	1600.754	1601.737	809.882	801.369	801.872	14
6	584.268	601.295	566.257	567.242	292.634	301.147	600.287	N	1531.727	1514.705	1515.713	1513.722	1514.705	766.366	757.853	758.357	13
7	744.299	761.326	726.288	727.274	372.649	381.163	760.318	C(+57.02)	1417.680	1400.662	1401.670	1399.679	1400.662	709.345	700.831	701.335	12
8	845.346	862.373	827.336	828.322	423.173	431.687	861.366	T	1257.662	1240.632	1241.639	1239.648	1240.632	629.329	620.816	621.320	11
9	932.377	949.405	914.370	915.360	466.689	475.203	948.398	S	1156.611	1139.584	1140.592	1138.600	1139.584	578.805	570.292	570.796	10
10	1029.431	1046.458	1011.421	1012.404	515.216	523.729	1045.450	P	1069.579	1052.553	1053.560	1051.568	1052.553	535.289	526.776	527.280	9

11.8.6 Partial Survey Chart

The “Survey” tab displays the precursor ion spectrum. For Orbitrap instruments, the controls are the same that appear in this section are identical to those explained above in 11.8.3.3 Partial Spectrum Chart - Additional Chart Controls.

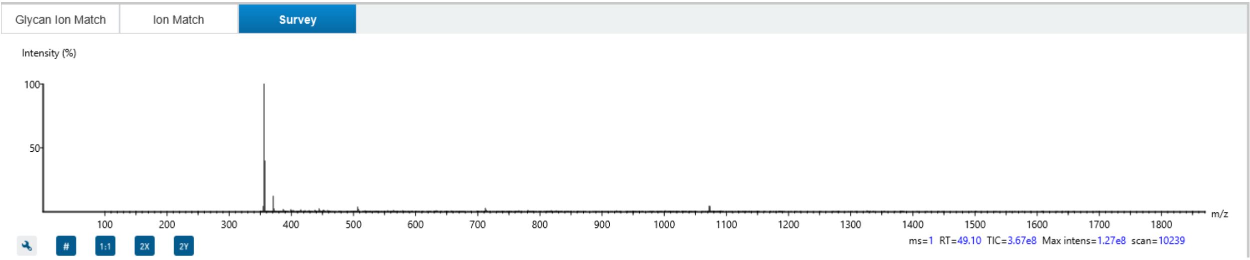

11.9 Glycan Search Exporting

In each of the Glycan Search tables, there is an export button. Clicking on will open the Export dialog which will contain options to export the table in CSV format. This export considers all table view filters and sorting applied to the table. This export is meant to be the same as what is shown in the table.

For the total exporting options, the Export node can be opened by double-clicking on the Export Node Export in the Project View. The Export node contains all the text and HTML exports for the relevant tables provided in the Glycan Search interface.

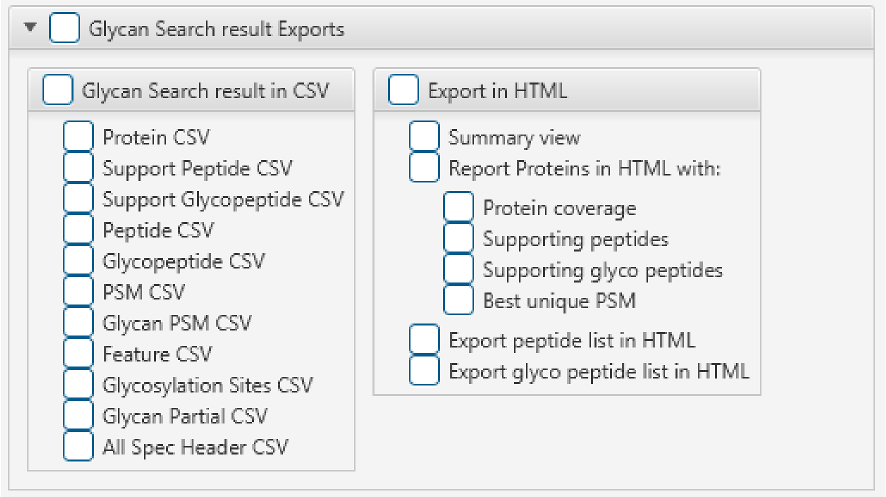

Click on Select All to select all export options. Click on Clear All to deselect all export options.

Click on any individual checkbox to enable that file for exporting or click on a parent checkbox to select all the exports underneath that group. For example, selecting "Glycan Search result Exports" will check all the CSV exports underneath it.

Click on the Browse button to select a location to save the exports into.

Click on Export to begin exporting all the files. Once the export is complete, a pop-up window will show.

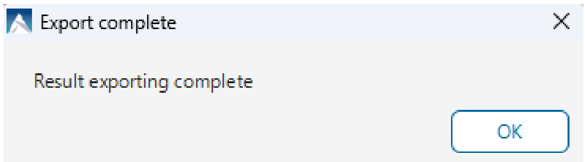

In the following sections we will go over each of the export contents.

Selecting “Glycan Search table exports in CSV” will select all exports that are in .csv format and the exports that are formed from the various tables provided in the Glycan Search result.

Note: Unless otherwise specified, the exports consider the summary page filters as set in the analysis. The View Filters are not considered in the Exports as those filters are intended to only filter the view for easy interpretation.

11.9.1 Export - Glycan Search Protein CSV

Exports the Protein table into *Glycan Search.proteins.csv* file. The columns in the export consists of those that are explained in the Protein table with optional columns checked and includes a few additional columns and differences:

- **Protein Group:** This is the same as the Cluster column in the proteins table and it is the protein cluster ID.
- **#Glycans:** The count of the unique glycan types identified in this protein.
- **Glycan ID:** A unique ID assigned to each Glycan structure to distinguish between them.
- **Glycan Found:** A TRUE/FALSE flag for easy filtering the .csv which indicates whether there are glycopeptides in this protein.
- **#Glycan Sites:** Indicates the number of sites in the protein residue that has glycosylation.

11.9.2 Export - Glycan Search Support Peptide CSV

Exports the Supporting Native Peptides table into *Glycan Search.protein-peptides.csv* file. The columns in the export consists of those that are explained in the Protein supporting glycopeptides table with some useful additional columns:

- **Protein Group:** Since the protein table is not present, a protein group number is provided.
- **Accession:** Likewise, to know which protein this supporting native peptide belongs to, the protein accession is saved in the export.
- **Top:** Indicates whether this is protein is a top protein in the protein group.
- **z:** The charge of the native peptide. Selected based on the top PSM.
- **Area per sample:** The area for each sample associated with this supporting native peptide.

11.9.3 Export - Glycan Search Support Glycan Peptide CSV

Exports the Supporting Glycan Peptides table into *Glycan Search.glycoprotein-peptides.csv* file. The columns in the export consists of those that are explained in the Protein supporting non-glycopeptides tabke with some useful additional columns:

- **Protein Group:** Since the protein table is not present, a protein group number is provided.
- **Accession:** Likewise, to know which protein this supporting native peptide belongs to, the protein accession is saved in the export.
- **Top:** Indicates whether this is protein is a top protein in the protein group.

- **Glycan ID:** A unique ID assigned to each Glycan structure to distinguish between them.
- **Glycan Mass:** The mass of the glycan(s) in this glycan peptide. Provided for easy sorting.
- **Area per sample:** The area for each sample associated with this supporting glycopeptide.
- **Source File:** The source file for which this Scan belongs to. The Scan and Source File of the highest -10lgP score PSM is the one shown.
- **#Feature per sample:** The number of features in each sample associated with this supporting glycopeptide.
- **Glycosylation position in Peptide:** Indicates which amino acid in the peptide chain has the glycosylation.
- **Glycosylation position in Protein:** Indicates which site in the protein residue has the glycosylation that has been identified in this glycopeptide.

11.9.4 Export - Glycan Search Peptide CSV

Exports the Native Peptides table into *Glycan Search.peptides.csv* file.

The columns in the export consists of those that are explained in the Peptide table section11.6.1 Peptide Table.

11.9.5 Export - Glycan Search Glycan Peptide CSV

Exports the Glycan Peptides table into *Glycan Search.glycopeptides.csv* file.

The columns in the export consists of those that are explained in Glycopeptide table section with some useful additional columns:

- **Glycan ID:** A unique ID assigned to each Glycan structure to distinguish between them.
- **Glycan Mass:** The mass of the glycan(s) in this glycan peptide. Provided for easy sorting.
- **z:** The charge of the glycan peptide. Selected based on the top PSM.
- **Glycosylation position in peptide:** Indicates which amino acid in the peptide chain has the glycosylation.

11.9.6 Export - Glycan Search PSM CSV

This Peptide Spectrum Match export is separated for each sample and is named in the format *Glycan Search.psms.[sample name].csv*.

The following columns are exported:

- **Peptide:** The amino acid sequence of the peptide, as determined by the Glycan Search search workflow. A modified residue is followed by a pair of parentheses enclosing the modification.
- **-10lgP:** The PSM -10lgP score. The score indicates the scoring significance of a peptide-precursor spectrum match.
- **Mass:** The monoisotopic mass of the peptide.

- **Length:** The number of amino acids in the sequence backbone.
- **ppm:** The precursor mass error, calculated as 10^6 × (precursor mass - peptide mass) / peptide mass.
- **m/z:** The precursor mass-to-charge ratio.
- **z:** The charge of the PSM.
- **RT:** The retention time (elution time) of the spectrum as recorded in the data.
- **Scan:** The scan number of the PSM.
- **Area:** The area of the feature associated with this PSM.
- **Source File:** The fraction/data file that this spectrum belongs to.
- **Accession:** The accessions of proteins that contain this native PSM.
- **PTM:** The types and the numbers of modifications present in the peptide shown in color-coded icons.
- **AScore:** Localization score assigned to modifications on the peptide. It is the -10 log of a p-value. In this case, the p-value is the probability that the modification occurs at the reported position compared to other possible positions. A -10lgP of 20 is equal to a p-value of 0.01. Scores are listed in the format AminoAcid, peptide position, modification, Ascore. For example, serine at amino acid 3 is phosphorylated with an AScore of 54.01 will be reported as: S3 P 54.01.

11.9.7 Export - Glycan Search Glycan PSM CSV

This Glycan Peptide Spectrum Match export is separated for each sample and is named in the format *Glycan Search.glycopsms.[sample name].csv*.

The following columns are exported:

- **Peptide:** The amino acid sequence of the glycan peptide, as determined by the Glycan Search search workflow. A modified residue is followed by a pair of parentheses enclosing the modification or glycan mass.
- **Glycan:** The Glycan moiety on this glycopeptide. For N-link results, there will always be 1. For O-link results, there could be more than 1.
- **Glycan ID:** A unique ID assigned to each Glycan structure to distinguish between them. A negative Glycan ID indicates a decoy glycan.
- **Glycan Type:** Simply states whether the glycan moiety is N-link or O-link. This column is particularly useful for N-Link + O-link search workflows.
- **-10lgP:** The peptide -10lgP score. The score indicates the scoring significance of a peptide-precursor spectrum match.
- **Glycan Score:** The normalized sum of ions log intensity, based on glycan fragment ion, peptide ion, and glycopeptide ion (Y ion).
- **Peptide Score:** Classifies glycan peptides with the normalized sum of ions log intensity, based on peptide fragment ions, and those peptide fragment ions with glycan. Different fragmentation type considers different peptide fragment ion:

- CID and HCD considers b,y ion without glycan and b,y ion with at most 1 glycan node from root
- ETD considers b,y,c,z,z' ion without glycan and b,y,c,z,z' ion with whole glycan from root
- ETHCD considers b,y,c,z,z' ion without glycan, b,y,c,z,z' ion with 1 glycan from root, and b,y,c,z,z' ion with whole glycan from root

- **Glycan Rank:** Each spectrum has up to 10 glycan candidates. The candidates are ordered by their glycan score and the rank means the rank of the glycan score which has the best final score. e.g. Rank 3 means the candidate has the 3rd best glycan score but has the best final score.
- **S-Score (%):** Measures the confidence of the top glycanpeptide candidate as opposed to other candidates for the spectrum. *S-Score = (finalScore top1candidate - finalScore top2candidate)/ finalScore top1candidate*. The higher the score the better. 100% indicates only 1 result, and it is the best match. 0% indicates that the top1 and top2 result are very similar, and we cannot confidently say the result is the best match.
- **Mass:** The monoisotopic mass of the glycan peptide.
- **Glycan Mass:** The mass of the glycan(s) in this glycan peptide. Provided for easy sorting.
- **Length:** The number of amino acids in the sequence backbone.
- **ppm:** The precursor mass error, calculated as $10^6 \times$ (precursor mass - peptide mass) / peptide mass.
- **m/z:** The precursor mass-to-charge ratio.
- **z:** The charge.
- **RT:** The retention time (elution time) of the spectrum as recorded in the data.
- **Area:** The area of the feature associated with this glycan PSM.
- **Scan:** The scan number of the glycan PSM.
- **Source File:** The fraction/data file that the spectrum belongs to.
- **Accession:** The accessions of proteins that contain this glycan PSM.
- **PTM:** The types and the numbers of modifications present in the peptide shown in color-coded icons.
- **AScore:** Localization score assigned to modifications on the peptide. It is the -10 log of a p-value. In this case, the p-value is the probability that the modification occurs at the reported position compared to other possible positions. A -10lgP of 20 is equal to a p-value of 0.01. Scores are listed in the format AminoAcid, peptide position, modification, Ascore. For example, serine at amino acid 3 is phosphorylated with an AScore of 54.01 will be reported as: S3 P 54.01.

11.9.8 Export - Glycan Search Feature CSV

The feature export is named in the format *Glycan Search.features.csv*

The columns shown in Features CSV export are consistent with the Feature with the columns rearranged and a few additional columns:

- **Glycan ID:** A unique ID assigned to each Glycan structure to distinguish between them.
- **-10lgP:** The top PSM -10lgP score. The score indicates the scoring significance of a peptide-precursor spectrum match.
- **Glycan Scores:** The normalized sum of ions log intensity, based on glycan fragment ion, peptide ion, and glycopeptide ion (Y ion).
- **Peptide Scores:** Classifies glycan peptides with the normalized sum of ions log intensity, based on peptide fragment ions, and those peptide fragment ions with glycan. Different fragmentation type considers different peptide fragment ion:
    - CID and HCD considers b,y ion without glycan and b,y ion with at most 1 glycan node from root
    - ETD considers b,y,c,z,z' ion without glycan and b,y,c,z,z' ion with whole glycan from root
    - ETHCD considers b,y,c,z,z' ion without glycan, b,y,c,z,z' ion with 1 glycan from root, and b,y,c,z,z' ion with whole glycan from root
- **Mass:** The monoisotopic mass.
- **Glycan Mass:** The mass of the glycan if present.
- **Length:** The length of the sequence backbone.
- **Glycosylation position in peptide:** Indicates which amino acid in the peptide chain has the glycosylation if it is present.

The Feature Vectors CSV export resembles the Features export except the m/z, RT mean, and Area is provided for each sample in separate columns.

11.9.9 Export - Glycan Search Glycosylation Sites CSV

This export is the full glycosylation sites statistics table as shown in the Summary page

The export is named in the format *Glycan Search.glycosylationSites.csv*

For all samples it will list both N-linked and O-linked Glycans for combined N-link and O-linked Glycan searches. For every position in proteins in the protein table, if the glycan is found at that position it will be counted as a Glycosylation Site in this table.

11.9.10 Export - Glycan Search Glycan Partial Spec CSV

This export is for the Partial table and is consistent with the columns in section 11.8.1 Partial Table. There is a separate export for each sample in the result and the exports are named in the format *Glycan Search. glycoPartialIdentifiedSpec.[sample name].csv*

A few extra columns are provided:

- **Glycan Mass:** The mass of the glycan if present.
- **RT:** The Retention Time of the spectrum.
- **PepMass:** The mass of the peptide portion of the unidentified spectrum.

11.9.11 Export - Glycan Search All Spec CSV

This export is for all spectrum in the result. It includes glycopeptides, native peptides, and the unidentified. There is a separate export for each sample in the result and the exports are named in the format *Glycan Search.spec.[sample name].csv*

The columns included in the export are as follows:

- **raw m/z**
- **raw z**
- **m/z:** The refined m/z.
- **z:** The charge after refinement (charge correction).
- **Scan:** The scan number of the spectrum.
- **Glycan:** Y or N flag indicating whether there is a glycan match.
- **Native:** Y or N flag indicating whether there is a native peptide match.
- **Source File:** The fraction or data file this spectrum belongs to.

11.9.12 Export - Glycan Search HTML

Export in HTML: This will generate single or multiple HTML reports in the specified location.

The following exporting options are available:

- **Summary view:** The "Summary" view page will be saved as a summary.html file in HTML format in the specified location.
- **Protein coverage:** The coverage pane will be saved for each protein.
- **Supporting peptides:** A list of supporting peptides will be saved for each protein.
- **Supporting glycol peptides:** A list of supporting glycopeptides will be saved for each protein.
- **Best unique PSM:** The best unique PSM will be saved for each protein.
- **Export peptide list in html:** creates a separate HTML-containing peptide identifications.
- **Export glycol peptide list in html:** creates a separate HTML-containing glycopeptide identifications.

An individual protein will have its own HTML output file, where the corresponding protein coverage, the supporting peptides, and the best unique PSM are gathered.

12. Tools

PEAKS Studio 11 comes with a few tools to help the user in analyzing results. These are the mass calculator, spectral library viewer, and compare result. There are two other useful tools in setting and monitoring performance which are the performance configuration tool and worker monitor tool.

12.1 Mass Calculator

The Mass Calculator is a handy tool used to quickly calculate mass or m/z based on sequence, modification, b/y-ion, and charge. Click on in the Tools tab to open the calculator.

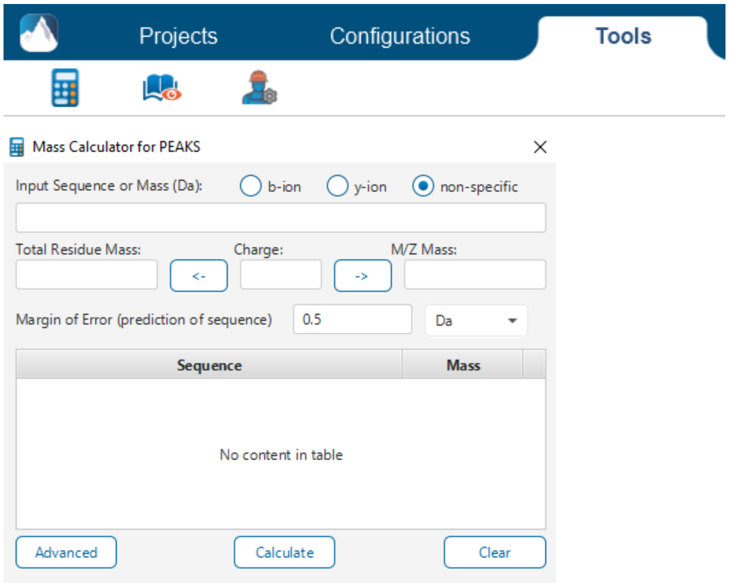

Copy/Type in a sequence (with modifications) and click on the 'Calculate' button to quickly find out the total residue mass and m/z mass.

12.2 Spectral Library Viewer

Click on in the Tools menu to open the Spectral Library Viewer.

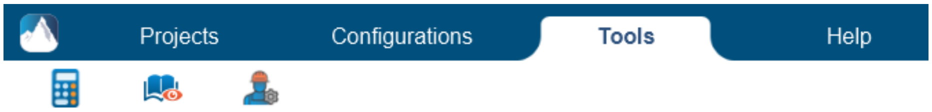

The Spectral Library Viewer is very useful in analyzing the contents of PEAKS Studio compatible libraries. It shows library info, distribution charts, library table content, and a spectrum chart of the top 20 ions.

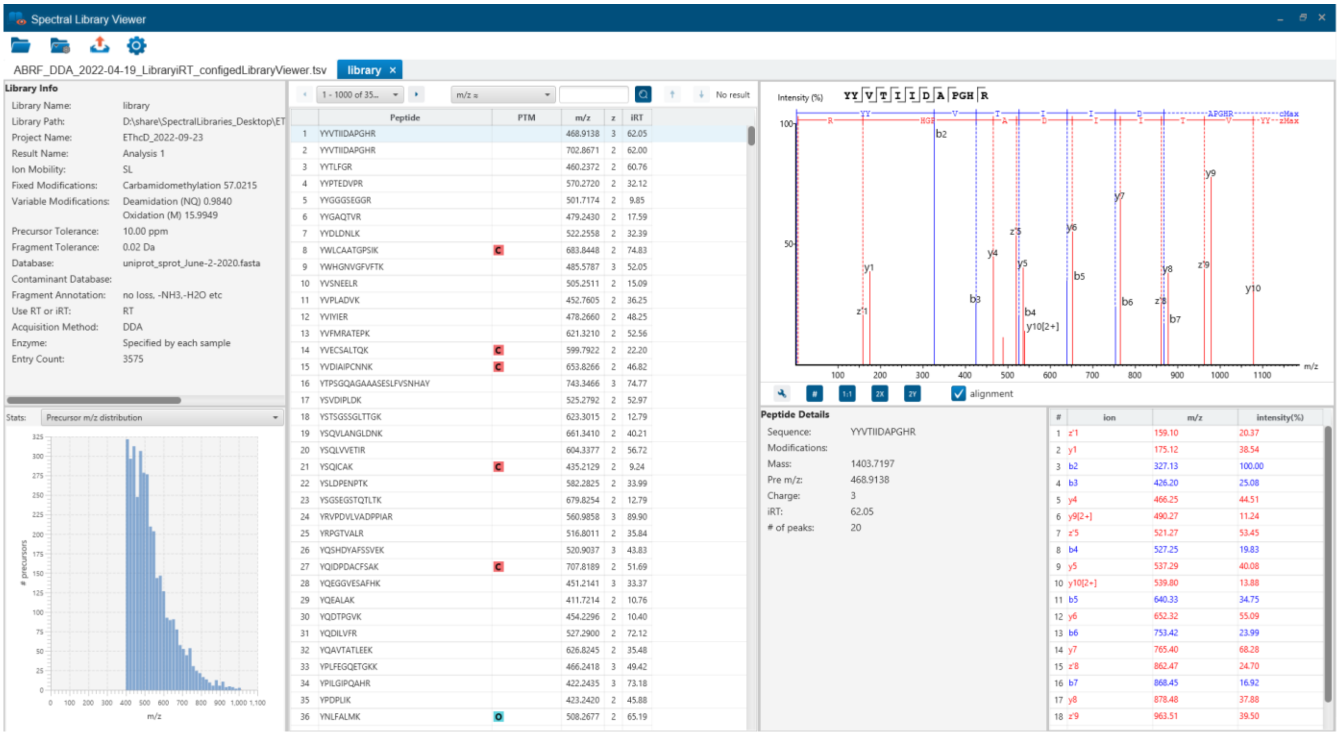

To open a library, click on 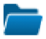 and select a PEAKS compatible library. Spectral Library Viewer supports PEAKS text format libraries generated by PEAKS Studio, PEAKS Online, and even PEAKS Studio Xpro (the older version of PEAKS Studio). In the Open dialog box, select the .tsv file (for Online and PEAKS Studio 11 spectral libraries) or the .lib file (for PEAKS Studio Xpro spectral libraries). The viewer will detect the library file format and also search for the .info (metainfo) file automatically. The library information and details will be shown in a separate tab in the main window of the viewer. Several libraries can be opened at once as tabs and can be switched between.

Already configured libraries can also be opened by clicking on the icon below for convenience. All configured libraries will be listed in a dropdown.

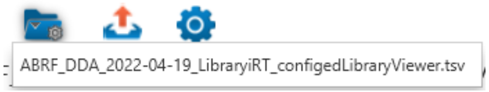

Click on 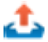 to export a copy of this library. This library will be saved in PEAKS Studio 11 format. This means that this will convert PEAKS Studio Xpro libraries into the PEAKS Studio 11 format.

Click on 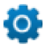 to directly configure this library into PEAKS Studio to be used in Library Search.

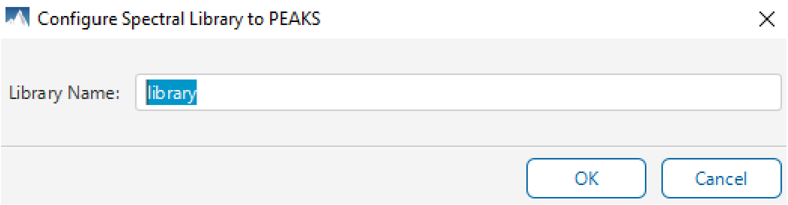

12.2.1 Library Viewer - Meta Information

Libraries generated by PEAKS products will come with a .info file which displays various information relevant to spectral library and its generation procedure. This file is not required and will not be necessary to configure the library or view in library viewer, but is recommended to accompany each library file.

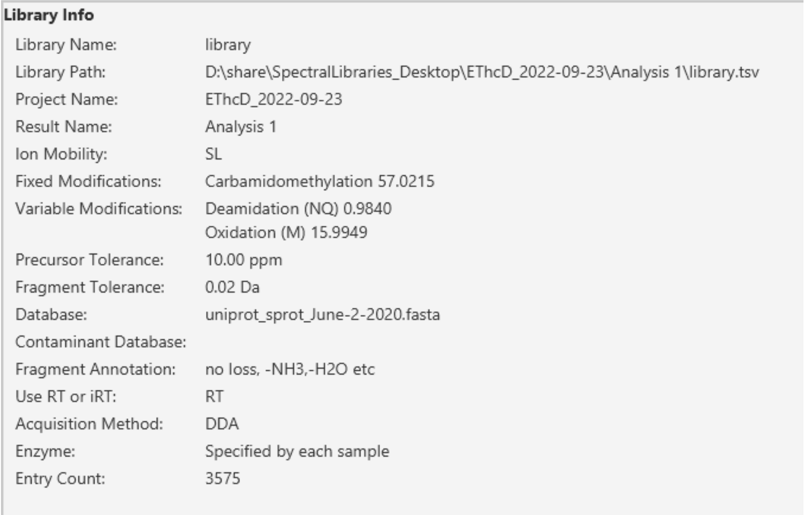

In the top left of the Library Viewer, the Library Info is extracted from the .info file. The following fields are displayed:

- Library Name: The name given to the library file. By default this would be library.tsv.
- Library Path: The location of the library file.
- Project Name: The name of the project used to generate the library.
- Result Name: The name of the analysis which the Database Search result used to generate the library.
- Ion Mobility: Shows as SL if there is no ion mobility in the library. Otherwise it may indicate that there is timsTOF 1/k0 or FAIMS CV values in the library.
- Fixed modifications: Fixed modifications used in the identification search used to generate the spectral library.
- Variable modifications: Variable modifications used in the identification search used to generate the spectral library.
- Precursor mass error tolerance: Precursor mass error tolerance used in the identification search used to generate the spectral library.
- Fragment mass error tolerance: Fragment ion mass error tolerance used in the identification search used to generate the spectral library.
- Database: Sequence database used for the database search used to generate the library.
- Contaminant Database: Contaminant database used for the database search used to generate the library.
- Fragmentation Annotation: Fragmentation annotation of the raw data.
- Use RT or iRT: Libraries can be saved with original RT or normalized iRT values. This indicates which of those options was used in generating this library.
- Acquisition Method: Shows if the library came from DDA or DIA data.
- Enzyme: Enzyme digest selected in the identification search used to generate the spectral library.
- Entry Count: The total number of entries in the library.

Note: A spectral library might not include all of the meta information listed above.

12.2.2 Library Viewer - Statistical Distribution

The spectral Library Viewer is equipped with several statistical analysis of the library for quality assessment and validation. Different statistical plots can be selected from a drop-down list and the corresponding graph is displayed.

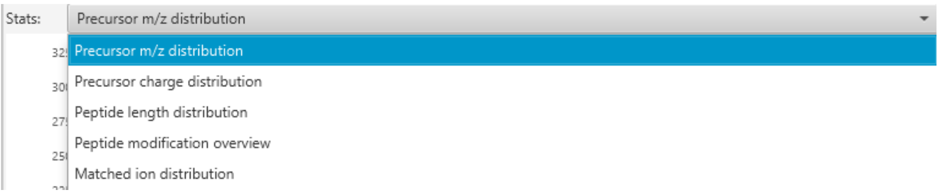

The following statistical plots are available for validation purposes:

Precursor m/z distribution

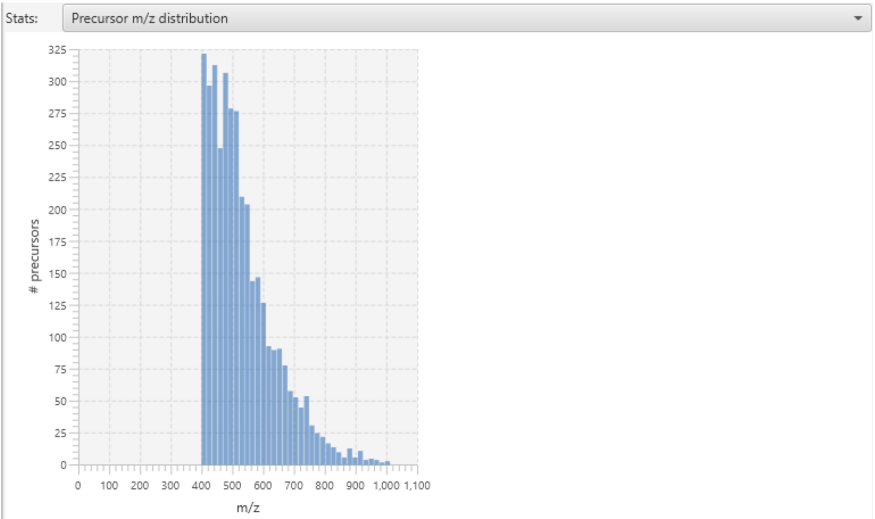

Precursor charge distribution

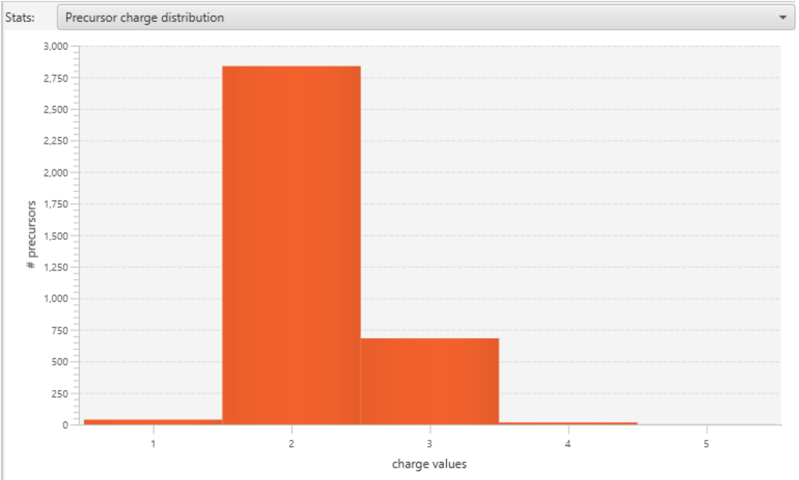

Peptide length distribution

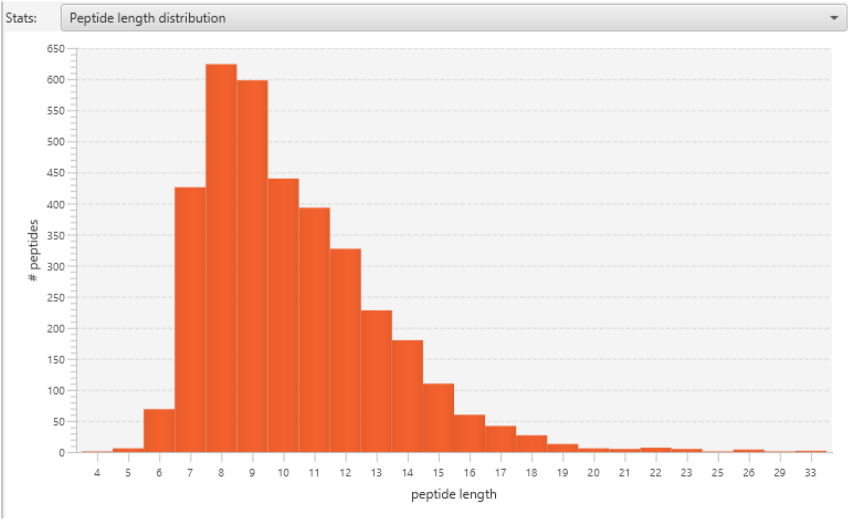

Peptide modification overview

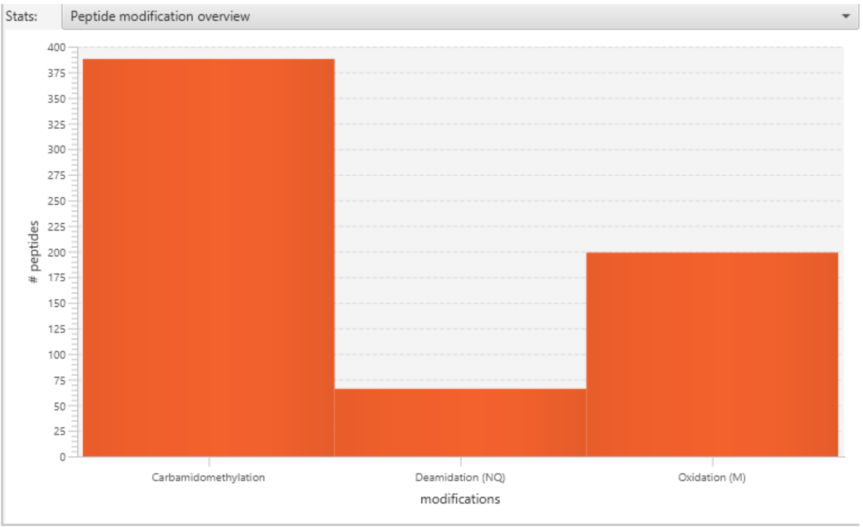

Matched ion distribution

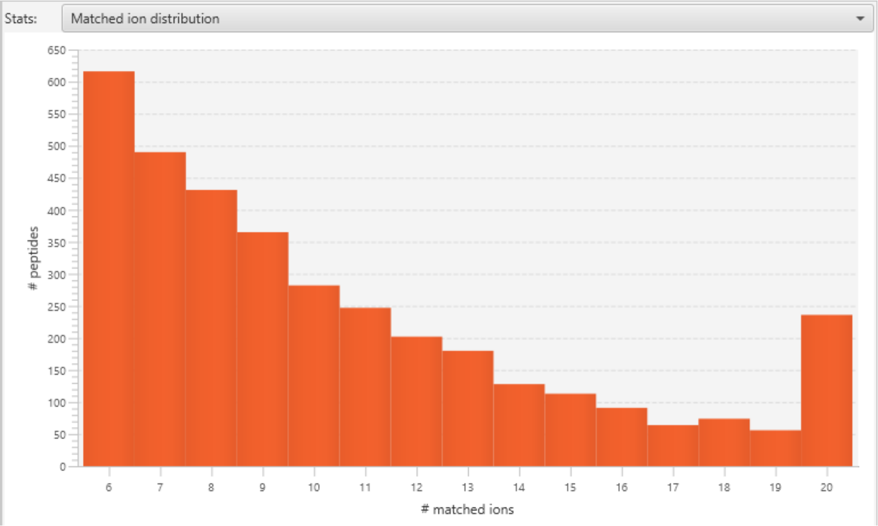

Right-click on any of the charts to export the image.

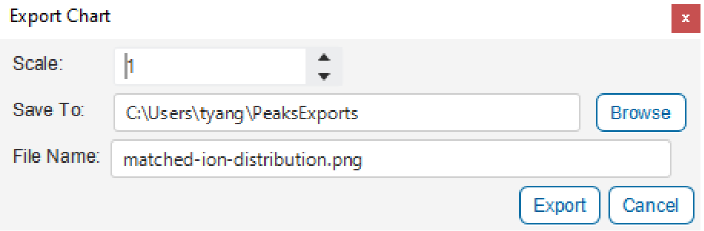

12.2.3 Library Viewer - Library Table

The spectral library entries are listed in the library table. Each row of the table corresponds to a ionized peptide entry in the library.

1 - 1000 of 3575 m/z ≈ No result

	Peptide	PTM	m/z	z	iRT
1	YYVTIIDAPGHR		468.9138	3	62.05
2	YYVTIIDAPGHR		702.8671	2	62.00
3	YYTLFGR		460.2372	2	60.76
4	YYPTEDVPR		570.2720	2	32.12
5	YYGGGSEGGR		501.7174	2	9.85
6	YYGAQTVR		479.2430	2	17.59
7	YYDLDNLK		522.2558	2	32.39
8	YWLCAATGPSIK	C	683.8448	2	74.83
9	YWHGNVGFVFTK		485.5787	3	52.05
10	YVSNEELR		505.2511	2	15.09
11	YVPLADVK		452.7605	2	36.25
12	YVIYIER		478.2660	2	48.25
13	YVFMRATEPK		621.3210	2	52.56
14	YVECSALTQK	C	599.7922	2	22.20
15	YVDIAIPCNNK	C	653.8266	2	46.82
16	YTPSGQAGAAASESLFVSNHAY		743.3466	3	74.77
17	YSVDIPLDK		525.2792	2	52.97
18	YSTSGSSGLTTGK		623.3015	2	12.79
19	YSQVLANGLDNK		661.3410	2	40.21
20	YSQLVVETIR		604.3377	2	56.72
21	YSQICAK	C	435.2129	2	9.24
22	YSLDPENPTK		582.2825	2	33.99
23	YSGSEGSTQTLTK		679.8254	2	12.79
24	YRVPDVLVADPPIAR		560.9858	3	89.90
25	YRPGTVALR		516.8011	2	35.84
26	YQSHDYAFSSVEK		520.9037	3	43.83
27	YQIDPDACFSAK	C	707.8189	2	51.69
28	YQEGGVESAFHK		451.2141	3	33.37
29	YQEALAK		411.7214	2	10.76
30	YQDTPGVK		454.2296	2	10.40
31	YQDILVFR		527.2900	2	72.12
32	YQAVTATLEEK		626.8245	2	35.48
33	YPLFEGQETGKK		466.2418	3	49.42
34	YPILGIPQAHR		422.2435	3	73.18
35	YPDPLIK		423.2420	2	45.88
36	YNLFALMK	O	508.2677	2	65.19

The library table contains the following columns:

- **Peptide:** Peptide base sequence with no modifications
- **PTM:** Modifications in the peptide, color coded for different modifications. Hovering over a PTM icon shows the detailed modification information such as the modified residue, modification site and modification mass.
- **m/z:** Theoretical mass-over-charge of the peptide.
- **z:** The charge of the peptide.
- **iRT:** Normalized retention time of the peptide if library was generated using iRT. This column will display un-normalized values if the library was general with original RT.

- **timsTOF 1/k0:** If the library is from timsTOF data. There will be 1/k0 values saved in the library which will be used in library search.
- **FAIMS CV:** If the library is from FAIMS data. The CV (compensation voltage) values are saved and will be used in library search.

Note: Even if a library does not have timsTOF 1/k0 or FAIMS CV, it can still be used to run library search for those respective data. If the library does have timsTOF 1/k0 or FAIMS CV, it can also be used to run library search for standard data and those column values will be disregarded.

All columns are sortable and can be rearranged.

12.2.3.1 Library Table - Pagination

By default, 1000 entries are shown in the table at any one time. This is to ensure performance so that only 1000 entries will be displayed and refreshed at one time. Click on the dropdown or the left/right arrows to switch between the 1000 entry segments.

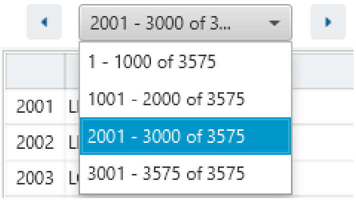

12.2.3.2 Library Table - Search function

On the top menu, there is a search function with 3 options. Search results can be navigated between using the Up or Down arrows.

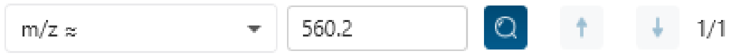

Options for searching include:

- **m/z ≈:** Search for the approximate m/z. The search will consider a range of m/z equal to the search value +/- 1 of the last significant digit. For example, searching for m/z ≈ 560.21 will find all m/z between [560.20, 560.22]
- **RT ≈:** Search for the approximate RT. Likewise, the search will consider a range of RT equal to the search value +/- 1 of the last significant digit set. For example, searching for RT ≈ 55.5 will find all m/z between [55.4, 55.6]
- **Seq contains:** Search for the sequence in the table. If specifying a sequence without PTMs, the results will include that sequence and any sequences with PTMs with the same backbone. If specifying a sequence with bracket and some mass value (like in the above image), then only sequence that have a substring with an exact match will be considered.
  Additionally, since the peptide table shows both I/L and they can not be distinguished, then searching for I in the sequence is equivalent to searching for L in the sequence. i.e. Searching for “LVLK” is the same as searching for “LVIK”, “IVLK”, and “IVIK”

12.2.3.3 Library Table - Spectrum Chart

Each library peptide will have a spectrum chart which displays the top 20 ions saved in the spectral library.

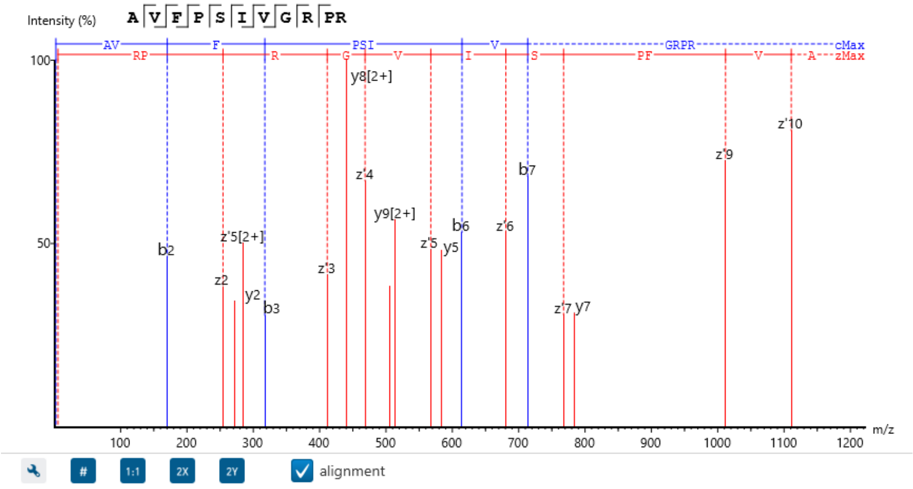

Moving the cursor over the peptide sequence in the spectrum will show the mass transitions for a particular amino acid residue.

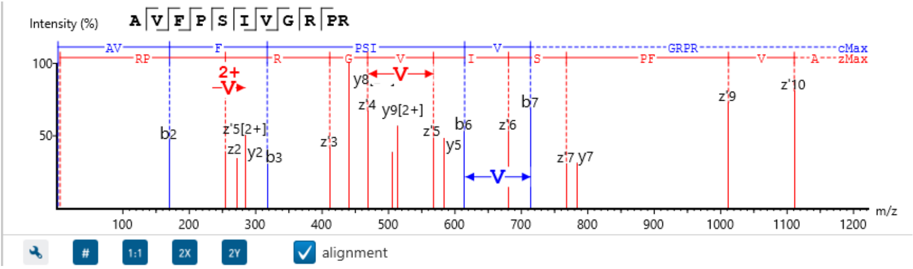

Zoom to a m/z region: Drag horizontally from the start m/z and to the end m/z with left mouse button.

Zoom in/out smoothly: Place the cursor at a particular m/z value (right below the x-axis), zoom in/out by scrolling the mouse wheel.

Increase/Decrease peak intensity: Place the cursor in the spectrum and scroll the mouse wheel.

See the whole spectrum: Double click in the spectrum or click the 1:1 button.

Click on the 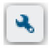 button to show the spectrum annotation settings.

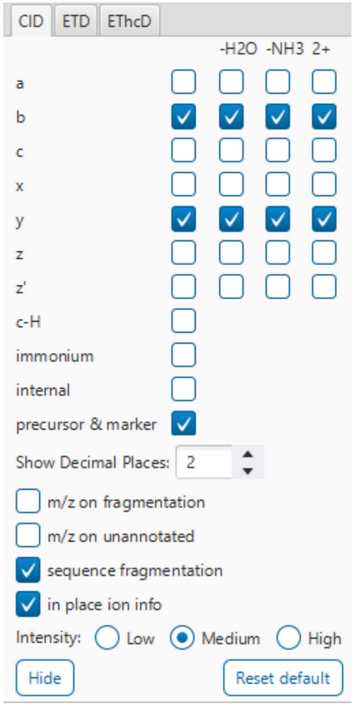

The spectra in the PEAKS results can be annotated by selecting ion types from the thorough collection of ions that PEAKS offers. It is possible to annotate the spectrum with various ions for CID, EThcD and ETD. Each of these fragmentation modes can be set with different settings which will be shared with other results with the same fragmentation type.

To reset the defaults, click on the “Reset default” button.

Show Decimal Places: Select the number of decimal places that will appear in the ion table and the spectrum view. The default is set to two decimal places.

m/z on Fragmentation: Select this to display the m/z value on top of the annotated ions.

m/z on Unannotated: Select this to display the m/z value on top of the peaks without ion matches.

Sequence Fragmentation: Select this to display the sequence fragmentation on the top-left corner of the “Spectrum Annotation” view.

In Place Ion Info: Ion information, m/z value, and relative intensity are all displayed in a pop-up within the “Spectrum Annotation” view when this option is checked and the cursor is placed on a peak.

Intensity: Set the intensity threshold for spectrum annotation to low (2%), medium (5%), or high (10%).

Click on # or % to toggle between absolute and percentage intensity values in the Y axis.

Click on 1:1 to reset the zoom level of the annotated spectrum chart to the default.

Click on 2X to zoom into the X axis by a factor of 2.

Click on 2Y to zoom into the Y axis by a factor of 2.

The "alignment" check box ☑ alignment allows user to display/hide how the fragment ions generated from the peptide align with the spectrum. N-terminal ions are shown in blue and C-terminal ions are shown in red.

12.2.3.4 Library Table - Peptide Details

For each selected peptide, more details are displayed.

Peptide Details

Sequence:	HTMMQARWK
Modifications:	3 Oxidation (M) 15.9949
Mass:	1203.5641
Pre m/z:	402.1953
Charge:	3
iRT:	16.93
# of peaks:	8

- **Sequence:** The peptide backbone sequence without modifications
- **Modifications:** Lists the modifications for this peptide, the mass value and the position in peptide
- **Mass:** The peptide mass
- **Pre m/z:** The precursor m/z
- **Charge:** Charge of the peptide
- **iRT:** This could be original RT or normalized iRT.
- **# of peaks:** The number of peaks in this library entry. Equal to the number of ions in the spectrum chart.

12.2.3.5 Library Table - Ion Table

List of fragment ions. The ion type, m/z and relative intensity of each fragment ion are listed in the table.

#	ion	m/z	intensity(%)
1	z1	158.09	30.86
2	y1	175.12	100.00
3	b4[2+]	181.08	3.83
4	y3[2+]	200.62	3.11
5	c3-H2O	229.13	6.00
6	c3-H	246.13	11.65
7	c4-H	377.17	4.80
8	y4	528.33	21.65
9	y5	643.35	15.63
10	y6	730.38	18.77
11	c8-H2O	805.35	19.77
12	b8	806.34	9.25
13	y7	845.41	53.26
14	b9	903.39	4.13
15	z'8	960.43	15.46
16	c10-H	1047.47	30.37
17	z10	1117.49	4.24

12.3 Compare Result

Click on 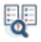 in the **Projects** menu to open the Compare Result dialog.

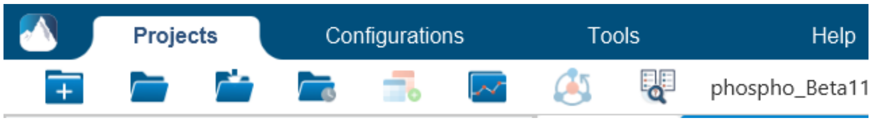

In compare result, 2 to 4 identification results can be selected to do a comparison. Important to note that only results in the same category can be used for comparison. There are four categories (which are split based on which workflow they are from):

- PEAKS DB search results: DB Search, PEAKS PTM, SPIDER
- DeepNovo Peptidome results: Peptide Search
- Glycan Search results: Glycan Search
- DIA Identification results: Spectral Library, DB Search

This means that if a DB Search result is selected for comparison, a Peptide Search or Glycan Search result cannot be selected, but another DB Search, a PEAKS PTM, or SPIDER result can be selected.

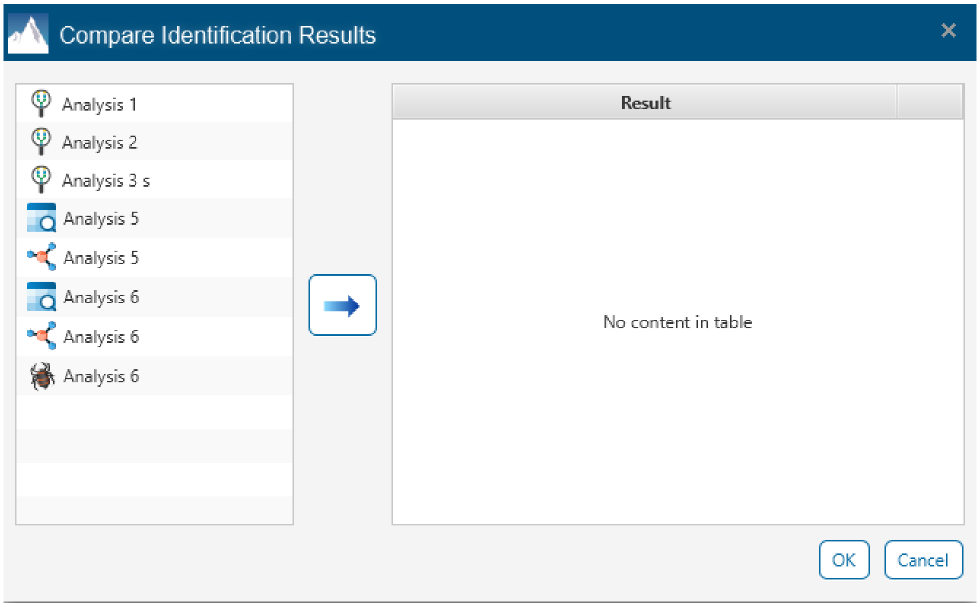

After selecting the results, a compare result dialog is shown. It has a Proteins tab and Peptide tab with table comparison, venn diagram, and score distribution. There is an additional Glycan tab for Glycan comparisons only.

12.3.1 Compare Result - Protein Compare Table

For results with Proteins, the set of proteins are compared between the different results. The key used for comparison is the Protein ID, which is the protein accession.

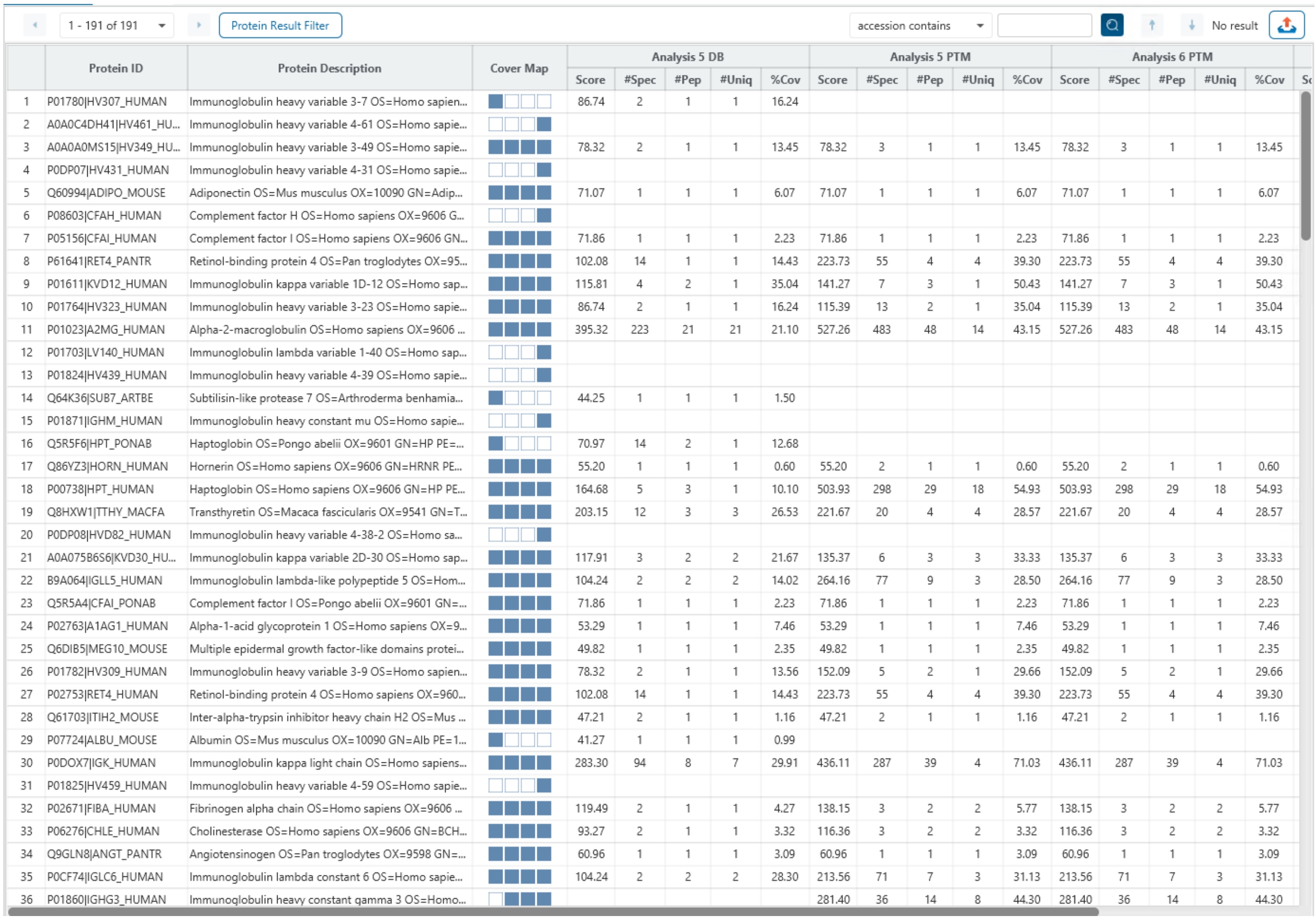

	Protein ID	Protein Description	Cover Map	Analysis 5 DB					Analysis 5 PTM					Analysis 6 PTM					
				Score	#Spec	#Pep	#Uniq	%Cov	Score	#Spec	#Pep	#Uniq	%Cov	Score	#Spec	#Pep	#Uniq	%Cov	Sc
1	P01780|HV307_HUMAN	Immunoglobulin heavy variable 3-7 OS=Homo sapien...	■□□□	86.74	2	1	1	16.24											
2	A0A0C4DH41|HV461_HU...	Immunoglobulin heavy variable 4-61 OS=Homo sapie...	□□□■																
3	A0A0A0MS15|HV349_HU...	Immunoglobulin heavy variable 3-49 OS=Homo sapie...	■■■■	78.32	2	1	1	13.45	78.32	3	1	1	13.45	78.32	3	1	1	13.45	
4	P0DP07|HV431_HUMAN	Immunoglobulin heavy variable 4-31 OS=Homo sapie...	□□□■																
5	Q60994|ADIPO_MOUSE	Adiponectin OS=Mus musculus OX=10090 GN=Adip...	■■■■	71.07	1	1	1	6.07	71.07	1	1	1	6.07	71.07	1	1	1	6.07	
6	P08603|CFAH_HUMAN	Complement factor H OS=Homo sapiens OX=9606 G...	□□□■																
7	P05156|CFAI_HUMAN	Complement factor I OS=Homo sapiens OX=9606 GN...	■■■■	71.86	1	1	1	2.23	71.86	1	1	1	2.23	71.86	1	1	1	2.23	
8	P61641|RET4_PANTR	Retinol-binding protein 4 OS=Pan troglodytes OX=95...	■■■■	102.08	14	1	1	14.43	223.73	55	4	4	39.30	223.73	55	4	4	39.30	
9	P01611|KVD12_HUMAN	Immunoglobulin kappa variable 1D-12 OS=Homo sap...	■■■■	115.81	4	2	1	35.04	141.27	7	3	1	50.43	141.27	7	3	1	50.43	
10	P01764|HV323_HUMAN	Immunoglobulin heavy variable 3-23 OS=Homo sapie...	■■■■	86.74	2	1	1	16.24	115.39	13	2	1	35.04	115.39	13	2	1	35.04	
11	P01023|A2MG_HUMAN	Alpha-2-macroglobulin OS=Homo sapiens OX=9606 ...	■■■■	395.32	223	21	21	21.10	527.26	483	48	14	43.15	527.26	483	48	14	43.15	
12	P01703|LV140_HUMAN	Immunoglobulin lambda variable 1-40 OS=Homo sap...	□□□■																
13	P01824|HV439_HUMAN	Immunoglobulin heavy variable 4-39 OS=Homo sapie...	□□□■																
14	Q64K36|SUB7_ARTBE	Subtilisin-like protease 7 OS=Arthroderma benhamia...	■□□□	44.25	1	1	1	1.50											
15	P01871|IGHM_HUMAN	Immunoglobulin heavy constant mu OS=Homo sapie...	□□□■																
16	Q5R5F6|HPT_PONAB	Haptoglobin OS=Pongo abelii OX=9601 GN=HP PE=...	■□□□	70.97	14	2	1	12.68											
17	Q86YZ3|HORN_HUMAN	Hornerin OS=Homo sapiens OX=9606 GN=HRNR PE...	■■■■	55.20	1	1	1	0.60	55.20	2	1	1	0.60	55.20	2	1	1	0.60	
18	P00738|HPT_HUMAN	Haptoglobin OS=Homo sapiens OX=9606 GN=HP PE...	■■■■	164.68	5	3	1	10.10	503.93	298	29	18	54.93	503.93	298	29	18	54.93	
19	Q8HXW1|TTHY_MACFA	Transthyretin OS=Macaca fascicularis OX=9541 GN=T...	■■■■	203.15	12	3	3	26.53	221.67	20	4	4	28.57	221.67	20	4	4	28.57	
20	P0DP08|HVD82_HUMAN	Immunoglobulin heavy variable 4-38-2 OS=Homo sa...	□□□■																
21	A0A075B6S6|KVD30_HU...	Immunoglobulin kappa variable 2D-30 OS=Homo sap...	■■■■	117.91	3	2	2	21.67	135.37	6	3	3	33.33	135.37	6	3	3	33.33	
22	B9A064|IGLL5_HUMAN	Immunoglobulin lambda-like polypeptide 5 OS=Hom...	■■■■	104.24	2	2	2	14.02	264.16	77	9	3	28.50	264.16	77	9	3	28.50	
23	Q5R5A4|CFAI_PONAB	Complement factor I OS=Pongo abelii OX=9601 GN=...	■■■■	71.86	1	1	1	2.23	71.86	1	1	1	2.23	71.86	1	1	1	2.23	
24	P02763|A1AG1_HUMAN	Alpha-1-acid glycoprotein 1 OS=Homo sapiens OX=9...	■■■■	53.29	1	1	1	7.46	53.29	1	1	1	7.46	53.29	1	1	1	7.46	
25	Q6DIB5|MEG10_MOUSE	Multiple epidermal growth factor-like domains protei...	■■■■	49.82	1	1	1	2.35	49.82	1	1	1	2.35	49.82	1	1	1	2.35	
26	P01782|HV309_HUMAN	Immunoglobulin heavy variable 3-9 OS=Homo sapien...	■■■■	78.32	2	1	1	13.56	152.09	5	2	1	29.66	152.09	5	2	1	29.66	
27	P02753|RET4_HUMAN	Retinol-binding protein 4 OS=Homo sapiens OX=960...	■■■■	102.08	14	1	1	14.43	223.73	55	4	4	39.30	223.73	55	4	4	39.30	
28	Q61703|ITIH2_MOUSE	Inter-alpha-trypsin inhibitor heavy chain H2 OS=Mus ...	■■■■	47.21	2	1	1	1.16	47.21	2	1	1	1.16	47.21	2	1	1	1.16	
29	P07724|ALBU_MOUSE	Albumin OS=Mus musculus OX=10090 GN=Alb PE=1...	■□□□	41.27	1	1	1	0.99											
30	P0DOX7|IGK_HUMAN	Immunoglobulin kappa light chain OS=Homo sapiens...	■■■■	283.30	94	8	7	29.91	436.11	287	39	4	71.03	436.11	287	39	4	71.03	
31	P01825|HV459_HUMAN	Immunoglobulin heavy variable 4-59 OS=Homo sapie...	□□□■																
32	P02671|FIBA_HUMAN	Fibrinogen alpha chain OS=Homo sapiens OX=9606 ...	■■■■	119.49	2	1	1	4.27	138.15	3	2	2	5.77	138.15	3	2	2	5.77	
33	P06276|CHLE_HUMAN	Cholinesterase OS=Homo sapiens OX=9606 GN=BCH...	■■■■	93.27	2	1	1	3.32	116.36	3	2	2	3.32	116.36	3	2	2	3.32	
34	Q9GLN8|ANGT_PANTR	Angiotensinogen OS=Pan troglodytes OX=9598 GN=...	■■■■	60.96	1	1	1	3.09	60.96	1	1	1	3.09	60.96	1	1	1	3.09	
35	P0CF74|IGLC6_HUMAN	Immunoglobulin lambda constant 6 OS=Homo sapie...	■■■■	104.24	2	2	2	28.30	213.56	71	7	3	31.13	213.56	71	7	3	31.13	
36	P01860|IGHG3_HUMAN	Immunoglobulin heavy constant gamma 3 OS=Homo...	□■■■						281.40	36	14	8	44.30	281.40	36	14	8	44.30	

The following columns are in the Protein compare table:

- **Protein Description:** The description of the protein.
- **Cover Map:** Indicates which results contain this Protein ID. The cover map order is the same as the other of the results in the table.
- **Score:** PEAKS protein -10lgP score.
- **#Spec**: The number of PSMs matched to supporting peptides for the protein.
- **#Pep:** The number of supporting peptides for the protein.
- **#Uniq:** The number of unique supporting peptides for the protein.
- **%Cov:** The sequence coverage of the protein by supporting peptides.

12.3.1.1 Protein Compare Table - Pagination

By default, 1000 entries are shown in the table at any one time. This is to ensure performance so that only 1000 entries will be displayed and refreshed at one time. Click on the dropdown or the left/right arrows to switch between the 1000 entry segments.

12.3.1.2 Protein Compare Table - Protein Result Filter

Click on the [Protein Result Filter] button to open the Protein Result Filter.

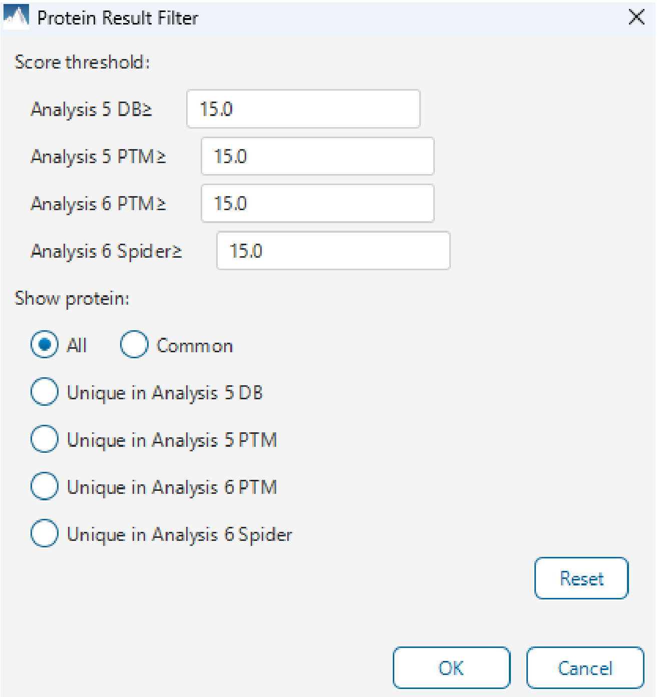

Score threshold: Sets a protein score threshold for each of the up to four results in the comparison.

Show protein: Only displays proteins that meet the criteria of the cover map below:

- All: All proteins are listed.
- Common: Only proteins found in every result are listed.
- Unique in X: Only proteins found in the selected result but not other results are listed.

Click on the [Reset] button to remove all filters.

Click on [OK] to apply the Protein View Filter to the Protein table.

Click on [Cancel] to discard changes and close the dialog.

12.3.1.3 Protein Compare Table - Search Function

An easy-to-use search function is available for the protein table. The protein accession or description substring can be searched for, and all search results can be navigated through with the Up and Down arrows.

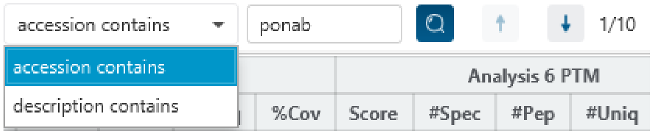

Protein accession/name contains: Restrict the protein table to only display protein IDs which contain the entered text in its accession/name.

Protein description contains: Only proteins with descriptions that contain the entered text will be included in the protein table if text is entered in this field.

12.3.1.4 Protein Compare Table - Exporting

Click on to open the Export pop-up and export the contents of the Protein table.

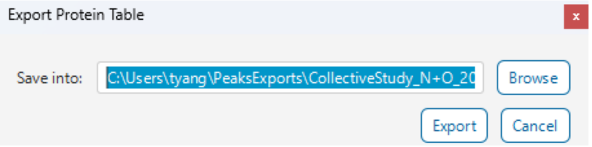

The export content is the same as what is displayed in the table, which consider the result filters.

12.3.1.5. Protein Compare Table - Venn Diagram

A Protein Number Venn Diagram is generated which shows the overlap of Proteins between the selected results. The type of venn diagram depends on the number of results nodes selected. As an example, the venn diagram below is comparing 4 result nodes. Different venn diagrams are also available for two sample and three sample comparisons.

The venn diagram considers the result filter and will update accordingly. Right-click on the venn diagram to get the option to export the image.

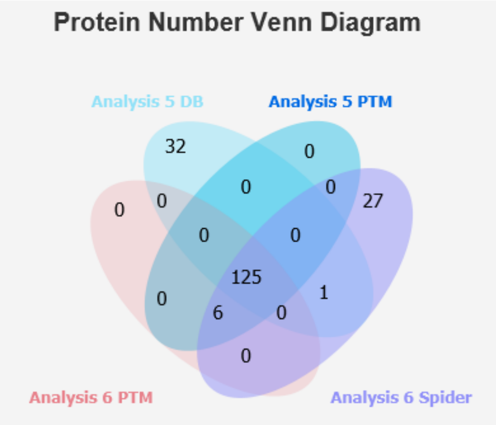

12.3.1.6. Protein Compare Table - Score Distribution

The Protein Score Distribution plot shows the number of proteins at a certain score. The higher the rank, the higher the protein score. This gives a quick overview of how many proteins are at certain score thresholds.

The score distribution chart considers the result filter and will update accordingly. Right-click on the score distribution chart to get the option to export the image.

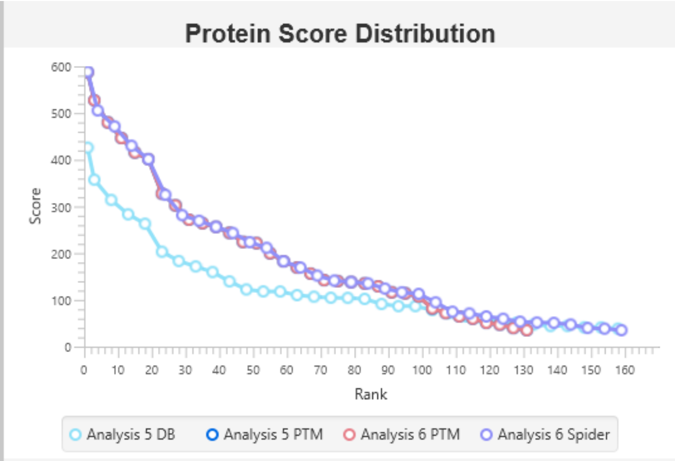

12.3.2 Compare Result - Peptide Compare Table

The set of peptides are compared between the different results. The key used for comparison is the Peptide sequence.

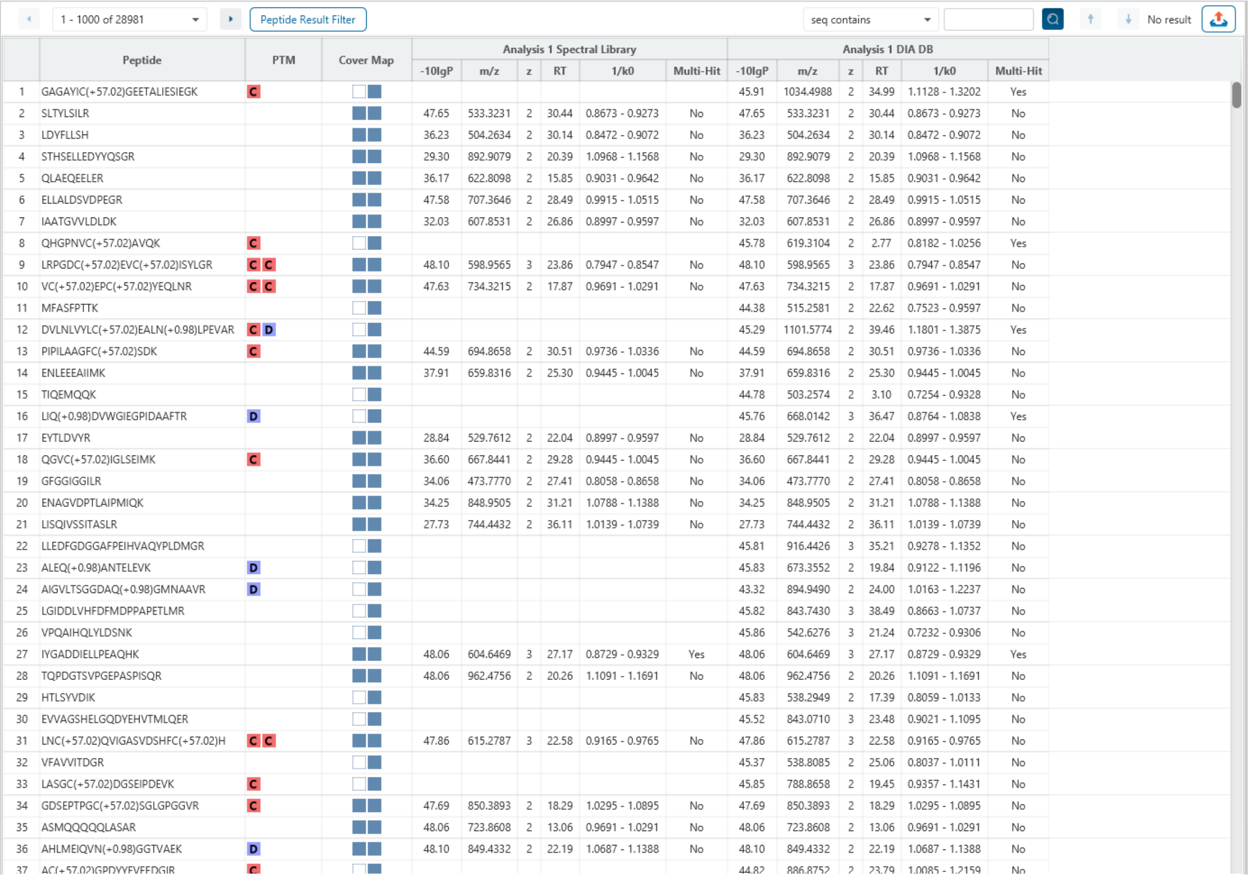

	Peptide	PTM	Cover Map	Analysis 1 Spectral Library						Analysis 1 DIA DB					
				-10lgP	m/z	z	RT	1/k0	Multi-Hit	-10lgP	m/z	z	RT	1/k0	Multi-Hit
1	GAGAYIC(+57.02)GEETALIESIEGK	C								45.91	1034.4988	2	34.99	1.1128 - 1.3202	Yes
2	SLTYLSILR			47.65	533.3231	2	30.44	0.8673 - 0.9273	No	47.65	533.3231	2	30.44	0.8673 - 0.9273	No
3	LDYFLLSH			36.23	504.2634	2	30.14	0.8472 - 0.9072	No	36.23	504.2634	2	30.14	0.8472 - 0.9072	No
4	STHSELLEDYYQSGR			29.30	892.9079	2	20.39	1.0968 - 1.1568	No	29.30	892.9079	2	20.39	1.0968 - 1.1568	No
5	QLAEQEELER			36.17	622.8098	2	15.85	0.9031 - 0.9642	No	36.17	622.8098	2	15.85	0.9031 - 0.9642	No
6	ELLALDSVDPEGR			47.58	707.3646	2	28.49	0.9915 - 1.0515	No	47.58	707.3646	2	28.49	0.9915 - 1.0515	No
7	IAATGVVLDLDK			32.03	607.8531	2	26.86	0.8997 - 0.9597	No	32.03	607.8531	2	26.86	0.8997 - 0.9597	No
8	QHGPNVC(+57.02)AVQK	C								45.78	619.3104	2	2.77	0.8182 - 1.0256	Yes
9	LRPGDC(+57.02)EVC(+57.02)ISYLGR	C C		48.10	598.9565	3	23.86	0.7947 - 0.8547	No	48.10	598.9565	3	23.86	0.7947 - 0.8547	No
10	VC(+57.02)EPC(+57.02)YEQLNR	C C		47.63	734.3215	2	17.87	0.9691 - 1.0291	No	47.63	734.3215	2	17.87	0.9691 - 1.0291	No
11	MFASFPTTK									44.38	515.2581	2	22.62	0.7523 - 0.9597	No
12	DVLNLVYLC(+57.02)EALN(+0.98)LPEVAR	C D								45.29	1101.5774	2	39.46	1.1801 - 1.3875	Yes
13	PIPILAAGFC(+57.02)SDK	C		44.59	694.8658	2	30.51	0.9736 - 1.0336	No	44.59	694.8658	2	30.51	0.9736 - 1.0336	No
14	ENLEEEAIIMK			37.91	659.8316	2	25.30	0.9445 - 1.0045	No	37.91	659.8316	2	25.30	0.9445 - 1.0045	No
15	TIQEMQQK									44.78	503.2574	2	3.10	0.7254 - 0.9328	No
16	LIQ(+0.98)DVWGIEGPIDAAFTR	D								45.76	668.0142	3	36.47	0.8764 - 1.0838	Yes
17	EYTLDVYR			28.84	529.7612	2	22.04	0.8997 - 0.9597	No	28.84	529.7612	2	22.04	0.8997 - 0.9597	No
18	QGVC(+57.02)IGLSEIMK	C		36.60	667.8441	2	29.28	0.9445 - 1.0045	No	36.60	667.8441	2	29.28	0.9445 - 1.0045	No
19	GFGGIGGILR			34.06	473.7770	2	27.41	0.8058 - 0.8658	No	34.06	473.7770	2	27.41	0.8058 - 0.8658	No
20	ENAGVDPTLAIPMIQK			34.25	848.9505	2	31.21	1.0788 - 1.1388	No	34.25	848.9505	2	31.21	1.0788 - 1.1388	No
21	LISQIVSSITASLR			27.73	744.4432	2	36.11	1.0139 - 1.0739	No	27.73	744.4432	2	36.11	1.0139 - 1.0739	No
22	LLEDFGDGGAFPEIHVAQYPLDMGR									45.81	916.4426	3	35.21	0.9278 - 1.1352	No
23	ALEQ(+0.98)ANTELEVK	D								45.83	673.3552	2	19.84	0.9122 - 1.1196	No
24	AIGVLTSGGDAQ(+0.98)GMNAAVR	D								43.32	894.9490	2	24.00	1.0163 - 1.2237	No
25	LGIDDLVHFDFMDPPAPETLMR									45.82	843.7430	3	38.49	0.8663 - 1.0737	No
26	VPQAIHQLYLDSNK									45.86	542.6276	3	21.24	0.7232 - 0.9306	No
27	IYGADDIELLPEAQHK			48.06	604.6469	3	27.17	0.8729 - 0.9329	Yes	48.06	604.6469	3	27.17	0.8729 - 0.9329	Yes
28	TQPDGTSVPGEPASPISQR			48.06	962.4756	2	20.26	1.1091 - 1.1691	No	48.06	962.4756	2	20.26	1.1091 - 1.1691	No
29	HTLSYVDIK									45.83	538.2949	2	17.39	0.8059 - 1.0133	No
30	EVVAGSHELGQDYEHVTMLQER									45.52	843.0710	3	23.48	0.9021 - 1.1095	No
31	LNC(+57.02)QVIGASVDSHFC(+57.02)H	C C		47.86	615.2787	3	22.58	0.9165 - 0.9765	No	47.86	615.2787	3	22.58	0.9165 - 0.9765	No
32	VFAVVITDGR									45.37	538.8085	2	25.06	0.8037 - 1.0111	No
33	LASGC(+57.02)DGSEIPDEVK	C								45.85	788.8658	2	19.45	0.9357 - 1.1431	No
34	GDSEPTPGC(+57.02)SGLGPGGVR	C		47.69	850.3893	2	18.29	1.0295 - 1.0895	No	47.69	850.3893	2	18.29	1.0295 - 1.0895	No
35	ASMQQQQQLASAR			48.06	723.8608	2	13.06	0.9691 - 1.0291	No	48.06	723.8608	2	13.06	0.9691 - 1.0291	No
36	AHLMEIQVN(+0.98)GGTVAEK	D		48.10	849.4332	2	22.19	1.0687 - 1.1388	No	48.10	849.4332	2	22.19	1.0687 - 1.1388	No
37	AC(+57.02)GPDYYEVEFDGIR	C								44.82	886.8752	2	23.79	1.0085 - 1.2159	No

The following columns are in the Protein compare table:

- **Peptide:** The peptide sequence. Used as the key for comparison.
- **PTM:** The modifications in the peptide. Mouse-over the PTM to see more details in a tooltip.

- **Cover Map:** Indicates which results contain this peptide. The cover map order is the same as the other of the results in the table.
- **-10lgP:** Top PSM -10lgP score.
- **m/z:** The m/z of this peptide in this result.
- **z:** The charge of this peptide in this result.
- **RT:** The retention of this peptide in this result.
- **1/k0:** For timsTOF data only. Shows the 1/k0 value of the result.
- **CV:** For FAIMS data only. Shows the CV (compensation voltage) of the result.
- **Multi-Hit:** Indicates if there are multiple PSM hits.

12.3.2.1 Peptide Compare Table - Pagination

By default, 1000 entries are shown in the table at any one time. This is to ensure performance so that only 1000 entries will be displayed and refreshed at one time. Click on the dropdown or the left/right arrows to switch between the 1000 entry segments.

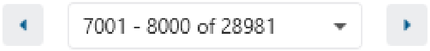

12.3.2.2 Peptide Compare Table - Peptide Result Filter

Click on the 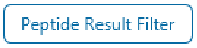

button to open the Peptide Result Filter.

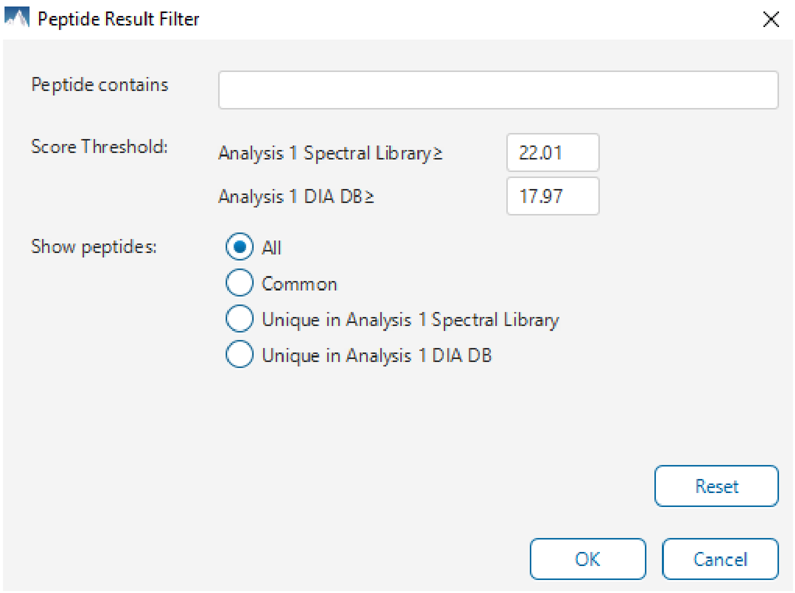

Peptide contains: Only keeps the peptides that contain the sequence. Multiple sequences can be listed here if separated by semicolon. E.g. search for "LSS;GV".

Score Threshold: Sets a peptide -10lgP threshold for each of the up to four results in the comparison.

Show peptides: Only displays proteins that meet the criteria of the cover map below:

- All: All peptides are listed.
- Common: Only peptides found in every result are listed.
- Unique in X: Only proteins found in the selected result but not other results are listed.

Click on the Reset button to remove all filters.

Click on OK to apply the Protein View Filter to the Protein table.

Click on Cancel to discard changes and close the dialog.

12.3.2.3 Peptide Compare Table - Search Function

An easy-to-use search function is available for the peptide compare table. Use this to search for certain sequences of interest.

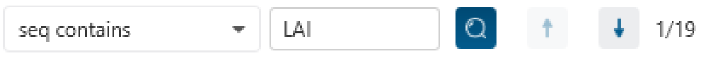

12.3.2.4 Peptide Compare Table - Exporting

Click on 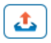 to open the Export pop-up and export the contents of the Peptide table.

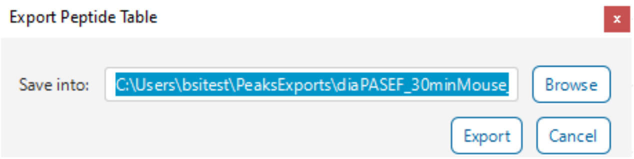

The export content is the same as what is displayed in the table, which consider the result filters.

12.3.2.5. Peptide Compare Table - Venn Diagram

A Peptide Number Venn Diagram is generated which shows the overlap of Peptides between the selected results. The type of venn diagram depends on the number of results nodes selected. As an example, the venn diagram below is comparing 2 result nodes. Different venn diagrams are also available for four sample and three sample comparisons.

The venn diagram considers the result filter and will update accordingly. Right-click on the venn diagram to get the option to export the image.

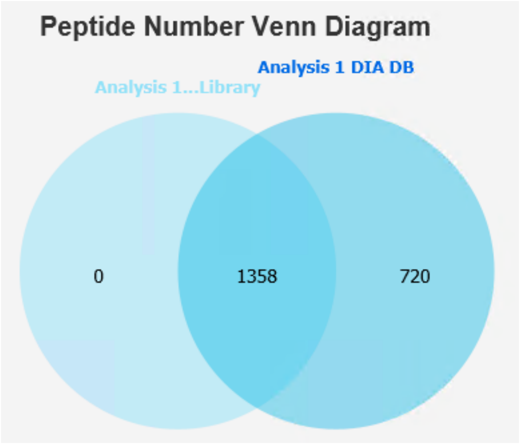

12.3.2.6. Peptide Compare Table - Score Distribution

The Peptide Score Distribution plot shows the number of peptides at a certain score. The higher the rank, the higher the peptide score. This gives a quick overview of how many proteins are at certain score thresholds.

The score distribution chart considers the result filter and will update accordingly. Right-click on the score distribution chart to get the option to export the image.

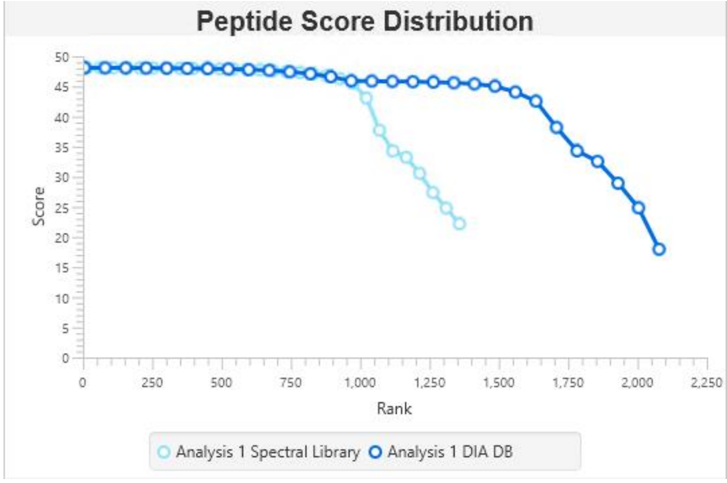

12.3.3 Compare Result - Glycan Compare Table

The set of glucan peptides are compared between the different results. The key used for comparison is the Peptide sequence.

	Peptide	Glycan	Glycan Type	PTM	Cover Map	Analysis 1						
						-10lgP	Glycan Score	Peptide Score	S-score	m/z	z	RT
1	QLAHQS(+1037.35)NS(+1150.40)TNIFFSPVSIATAFAM	(HexNAc)2(Hex)2(NeuGc)1	OLink	H H		19.17	15.31	23.03	0.00	1193.2616	4	84.31
2	LSLHRPALEDLLLGSEAN(+1872.65)LTC(+57.02)TLTGLR	(HexNAc)3(Hex)6(NeuAc)1	NLink	H C		20.86	22.30	19.43	1.00	1032.7094	4	34.37
3	HYTNPSQDVTVPC(+57.02)PVPS(+1955.72)TPPT(+1752.64)PSPSTPPT	(HexNAc)5(Hex)4(Fuc)2	OLink	C H H		23.93	16.30	31.55	0.00	1354.1702	5	32.63
4	HYTNPSQDVTVPC(+57.02)PVPSTPPT(+1005.36)PS(+2740.99)	(HexNAc)2(Hex)1(Fuc)1(NeuAc)1	OLink	C H H								
5	HRPALEDLLLGSEAN(+1501.55)LTC(+57.02)TLTGLR	(HexNAc)5(Hex)3	NLink	H C		32.15	23.31	40.99	0.00	965.7057	4	52.33
6	AKIMNGEADAMS(+1987.71)LDGGFVYIAGK	(HexNAc)5(Hex)6	OLink	H		19.47	17.88	21.06	1.00	1087.2271	4	53.46
7	VVLHPN(+2366.82)YSQVDIGLIKLK	(HexNAc)4(Hex)6(NeuAc)2	NLink	H		25.08	20.87	29.29	1.00	1101.5093	4	38.78
8	HYTNPSQDVTVPC(+57.02)PVPSTPPTPS(+1022.38)PS(+1913.68)TPPTP	(HexNAc)2(Hex)2(Fuc)2	OLink	C H H		25.34	15.75	34.94	0.00	1090.9467	4	47.76
9	AN(+2188.78)LSSQALQM(+15.99)SLDYGFVTPLTSMSIR	(HexNAc)4(Hex)4(Fuc)1(NeuAc)2	NLink	H O		53.79	26.11	81.47	1.00	1176.2284	4	37.70
10	GC(+57.02)VLLSYLN(+0.98)ET(+2262.81)VTVSASLESVR	(HexNAc)5(Hex)5(Fuc)1(NeuAc)1	OLink	C D H								
11	MVSHHN(+1897.68)LTTGATLINEQWLLTTAK	(HexNAc)4(Hex)4(Fuc)1(NeuAc)1	NLink	H		29.28	19.38	39.19	1.00	1108.4564	4	37.91
12	LAGKPTHVN(+2555.92)VSVVMAEVDGTC(+57.02)	(HexNAc)7(Hex)7	NLink	H C								
13	EEQYN(+1809.66)STYR	(HexNAc)5(Hex)4(Fuc)1	NLink	H		17.80	16.95	18.66	0.00	1000.3973	3	15.40
14	N(+2674.93)FSMIIDGMTYPGIIK	(HexNAc)4(Hex)7(Fuc)1(NeuAc)2	NLink	H		17.02	15.99	18.05	1.00	1119.4716	4	66.11
15	C(+57.02)GLVPVLAENYN(+2163.74)K	(HexNAc)3(Hex)6(NeuAc)2	NLink	C H		41.68	34.48	48.89	1.00	989.9480	4	34.62
16	PWQAKMVSHHNLT(+1913.68)TGAT(+1110.39)LINEQWLLTTAK	(HexNAc)4(Hex)5(NeuAc)1	OLink	H H		20.11	18.11	22.10	0.00	1263.5662	5	41.56
17	APNIYVLDYLN(+2903.04)ETQQLTPEIK	(HexNAc)6(Hex)5(Fuc)2(NeuAc)2	NLink	H		20.34	16.51	24.17	1.00	1346.3544	4	84.42
18	EEQYN(+1768.64)STYR	(HexNAc)4(Hex)5(Fuc)1	NLink	H		19.60	18.61	20.59	0.00	986.7230	3	15.14
19	T(+1150.40)T(+1150.40)EDC(+57.02)IAKIM(+15.99)NGEADAMSLDGGFVYIAGK	(HexNAc)2(Hex)1(NeuAc)2	OLink	H H C O		19.53	18.51	20.56	0.00	1349.3110	4	53.85
20	GLEWVSGISWN(+0.98)SGS(+2157.78)IGYADS(+1136.42)VK	(HexNAc)6(Hex)4(NeuAc)1	OLink	D H H								
21	LSLHRPALEDLLLGSEAN(+2075.73)LTC(+57.02)TLTGLR	(HexNAc)4(Hex)6(NeuAc)1	NLink	H C		36.04	30.66	41.41	1.00	972.1956	4	37.46
22	QSN(+2383.84)STNIFFSPVSIATAFAMLSLGTK	(HexNAc)4(Hex)7(Fuc)1(NeuAc)1	NLink	H		15.94	16.22	15.66	1.00	1279.8158	4	70.82
23	IEETTMTT(+656.23)QT(+656.23)PAPIQAPSAILPLPGQSVER	(HexNAc)1(Hex)1(NeuAc)1	OLink	H H		22.84	16.24	29.44	0.00	1031.5006	4	59.38
24	QDQC(+57.02)IYN(+3204.13)TTYLNVQR	(HexNAc)7(Hex)11	NLink	C H								
25	SLPGESEEMMEEVDQVTLYSYKVQS(+1540.53)TITS(+2278.81)R	(HexNAc)2(Hex)7	OLink	H H								
26	HYTNPSQDVTVPC(+57.02)PVPS(+2449.90)T(+1631.62)PPTPSP	(HexNAc)6(Hex)4(Fuc)2(NeuAc)1	OLink	C H H		21.10	18.99	23.20	0.00	1332.1603	5	26.11
27	PGS(+1095.40)GKDYAGVFS(+972.31)DAGLTFTSSSGQQTAQR	(HexNAc)3(Hex)3	OLink	H H		21.31	15.70	26.93	0.00	1247.7798	4	62.50
28	T(+1969.70)HDEILEGLNFNLT(+1944.63)EIPEAQIHEGFQELLR	(HexNAc)3(Hex)3(Fuc)2(NeuAc)2	OLink	H H		20.64	18.99	22.28	0.00	1237.5137	6	69.58
29	MVSHHN(+2862.02)LTTGATLINEQWLLTTAK	(HexNAc)5(Hex)6(Fuc)2(NeuAc)2	NLink	H		19.98	22.41	17.54	1.00	1269.7710	4	61.03
30	C(+57.02)T(+203.08)ANT(+963.32)NDC(+57.02)HWC(+57.02)NDHC(+57.02)VPR	(HexNAc)1	OLink	H H C C		45.27	39.57	50.98	0.00	770.8693	4	18.31
31	HYTNPSQDVTVPC(+57.02)PVPSTPPT(+146.06)PS(+1630.60)PSTPPTP	(Fuc)1	OLink	C F H		24.66	21.57	27.75	0.00	749.3499	4	29.81
32	C(+57.02)GLVPVLAENYN(+3260.16)K	(HexNAc)6(Hex)9(Fuc)2(NeuAc)1	NLink	C H		35.31	17.57	53.05	1.00	1190.4977	4	39.47
33	QQQHLFGSN(+3753.31)V	(HexNAc)7(Hex)9(NeuAc)3	NLink	H								
34	LS(+2141.79)LHRPALEDLLLGS(+1793.67)EAN(+0.98)LTC(+57.02)TLTGLR	(HexNAc)6(Hex)3(Fuc)1(NeuAc)1	OLink	H H D C								
35	HYTNPSQDVTVPC(+57.02)PVPS(+2448.88)T(+1971.72)PPTP	(HexNAc)6(Hex)4(NeuAc)2	OLink	C H H								
36	HYTNPSQDVTVPC(+57.02)PVPS(+1897.68)TPPTPS(+1529.51)PSTPPTP	(HexNAc)4(Hex)4(Fuc)1(NeuAc)1	OLink	C H H								

The following columns are in the Protein compare table:

- **Peptide:** The peptide sequence. Used as the key for comparison.
- **Glycan:** Lists the Glycan moiety for this peptide. For Olink
- **Glycan Type:** Indicates if it is an N-linked or O-linked glycopeptide.
- **PTM:** The modifications in the peptide. Mouse-over the PTM to see more details in a tooltip.
- **Cover Map:** Indicates which results contain this peptide. The cover map order is the same as the other of the results in the table.
- **-10lgP:** Top PSM -10lgP score.
- **Glycan Score:** The glycan score of each result.
- **Peptide Score:** The peptide score of each result.
- **S-score:** The S-score of each result.
- **m/z:** The m/z of this peptide in this result.
- **z:** The charge of this peptide in this result.
- **RT:** The retention of this peptide in this result.
- **1/k0:** For timsTOF data only. Shows the 1/k0 value of the result.
- **CV:** For FAIMS data only. Shows the CV (compensation voltage) of the result.
- **Multi-Hit:** Indicates if there are multiple PSM hits.

12.3.3.1 Glycan Compare Table - Pagination

By default, 1000 entries are shown in the table at any one time. This is to ensure performance so that only 1000 entries will be displayed and refreshed at one time. Click on the dropdown or the left/right arrows to switch between the 1000 entry segments.

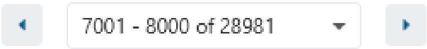

12.3.3.2 Glycan Compare Table - Peptide Result Filter

Click on the Glycan Result Filter button to open the Peptide Result Filter.

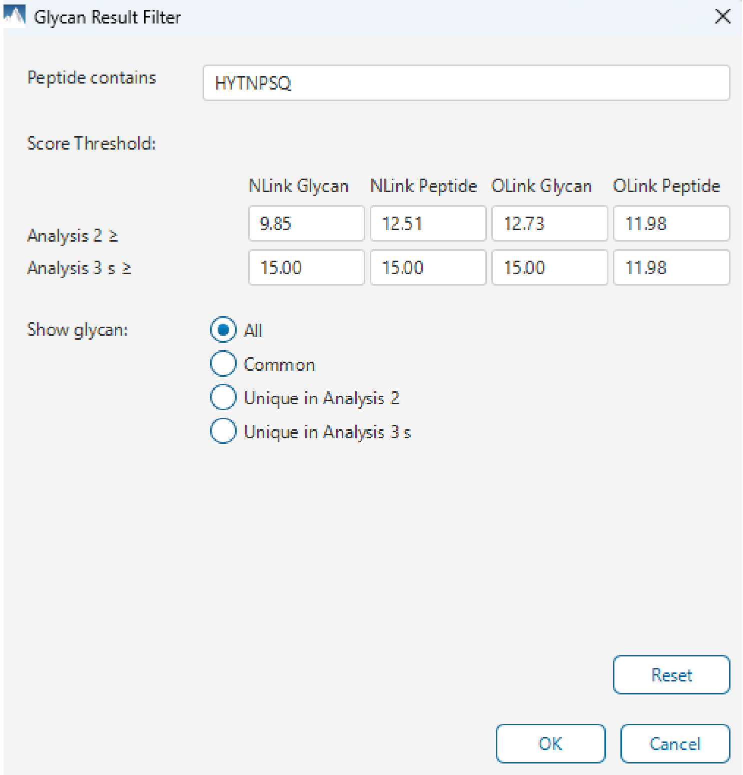

Peptide contains: Only keeps the peptides that contain the sequence. Multiple sequences can be listed here if separated by semicolon. E.g. search for “LSS;GV”.

Score Threshold: Sets a peptide -10lgP threshold for each of the up to four results in the comparison. There is a separate filter for Glycan score and Peptide score. There is also a sperate filter for N-linked glycopeptides and O-linked glycopeptides.

Show glycan: Only displays proteins that meet the criteria of the cover map below:

- All: All peptides are listed.
- Common: Only peptides found in every result are listed.
- Unique in X: Only proteins found in the selected result, but not other results are listed.

Click on the Reset button to remove all filters.

Click on OK to apply the Protein View Filter to the Protein table.

Click on Cancel to discard changes and close the dialog.

12.3.3.3 Glycan Compare Table - Search Function

An easy-to-use search function is available for the glycan compare table. Use this to search for certain sequences of interest.

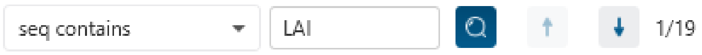

12.3.3.4 Glycan Compare Table - Exporting

Click on 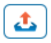 to open the Export pop-up and export the contents of the Peptide table.

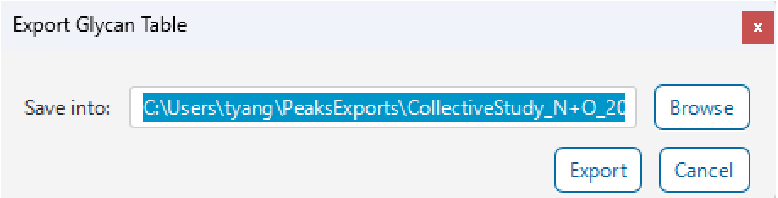

The export content is the same as what is displayed in the table, which consider the result filters.

12.3.3.5. Glycan Compare Table - Venn Diagram

A Peptide Number Venn Diagram is generated which shows the overlap of Glycopeptides between the selected results. The type of venn diagram depends on the number of results nodes selected. As an example, the venn diagram below is comparing 2 result nodes. Different venn diagrams are also available for four sample and three sample comparisons.

The venn diagram considers the result filter and will update accordingly. Right-click on the venn diagram to get the option to export the image.

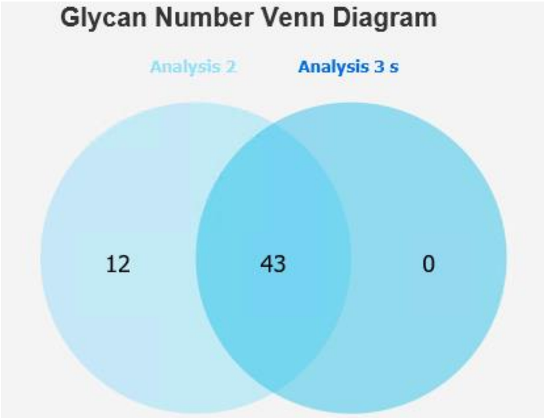

12.3.3.6. Glycan Compare Table - Glycan Score Distribution

The Glycan Score Distribution plot shows the number of glycan peptides at a certain glycan score. The higher the rank, the higher the glycan score. This gives a quick overview of how many proteins are at certain score thresholds.

The score distribution chart considers the result filter and will update accordingly. Right-click on the score distribution chart to get the option to export the image.

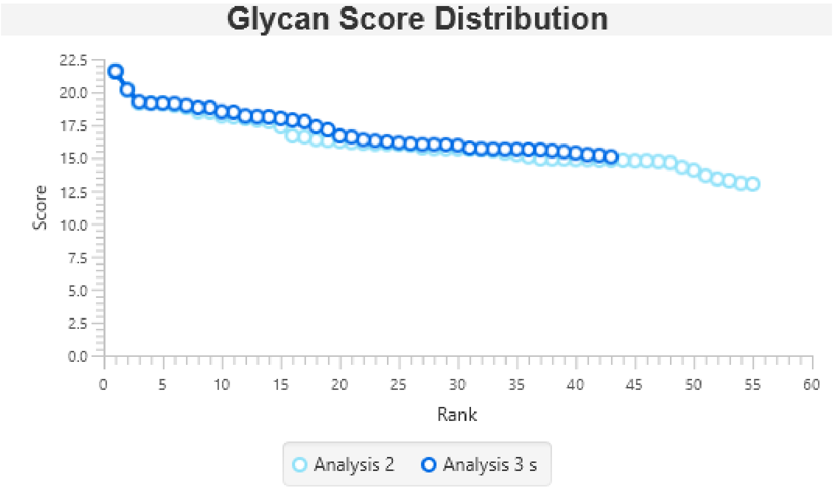

12.3.3.7. Glycan Compare Table - Peptide Score Distribution

The Peptide Score Distribution plot shows the number of glycan peptides at a certain peptide score. The higher the rank, the higher the peptide score. This gives a quick overview of how many proteins are at certain score thresholds.

The score distribution chart considers the result filter and will update accordingly. Right-click on the score distribution chart to get the option to export the image.

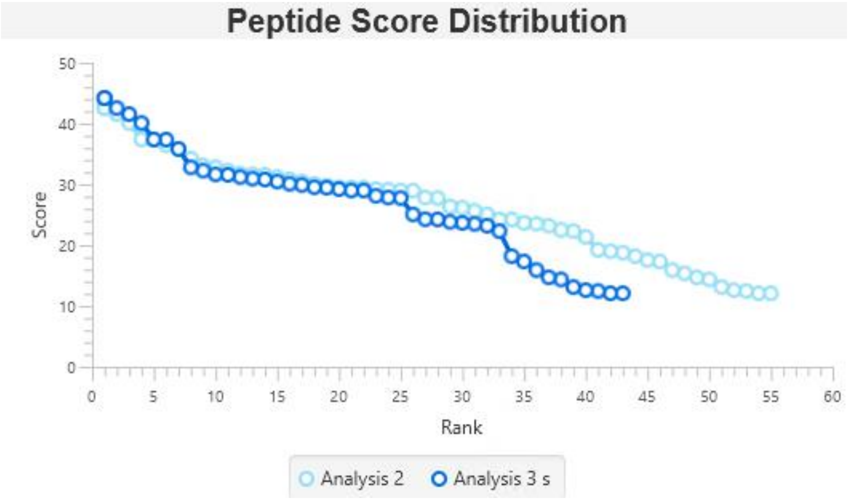

12.4 Performance Configuration

In the Configurations tab, click on the 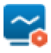 icon to open the Performance Configuration dialog.

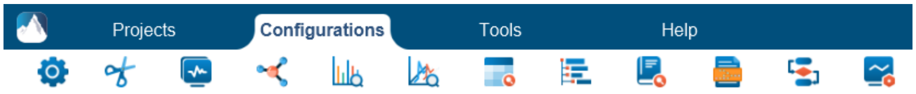

The Computer Resources shows the detected memory and number of processors of the computer.

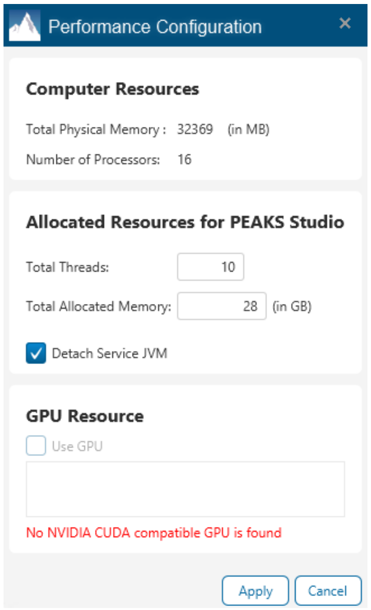

By default, performance configuration tries to set a reasonable total thread and allocated memory count to make use of the computer resources while leaving some for other programs.

You can manually change these default values and click ‘Apply’, but only do so if you understand the system requirements for other programs.

The Detach Service JVM is recommended to be checked. 2 threads and 8 GB memory will be reserved for processing user interface updates. If this is unchecked, the user interface processing and project/analysis processing tasks will be shared and PEAKS Studio may freeze more often if no resources are available for refreshing the user interface.

GPU resource checkbox will only be enabled if the computer has an NVIDIA CUDA compatible GPU. The GPU should have an updated driver and at least 8 GB memory to be considered usable. The GPU is only used for DIA Database step, and can be disregarded if not running DIA Database Search.

Click on ‘Cancel’ to discard any changes.

12.5 Worker Monitor

Click on 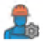 in the Tools tab to open the Worker Monitor.

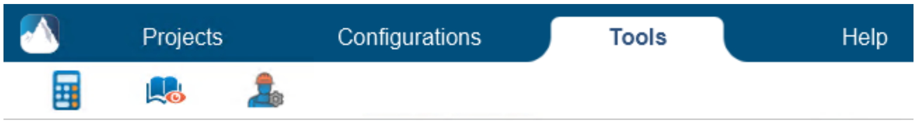

The Worker Monitor shows which tasks are currently running in PEAKS Studio. In addition, it indicates how many threads and how much memory is being used for each task. This is useful in checking the utilization of PEAKS Studio, and also for debugging purposes.

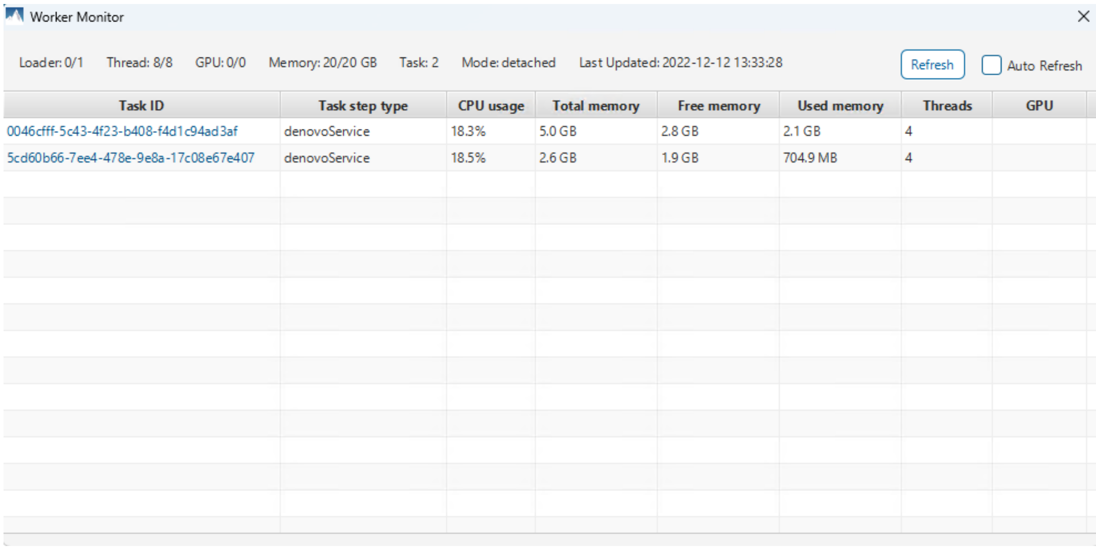

Click on the ‘Refresh’ button to update the worker monitor. Check ‘Auto Refresh’ to continuously update the worker monitor information every 2 seconds.
